# Supplementary material for: Reaction scope and mechanistic insights of nickel-catalyzed migratory Suzuki–Miyaura cross-coupling
Source: Nat Commun. 2020 Jan 21;11:417. doi: 10.1038/s41467-019-14016-1 (PMC6972863; doi:10.1038/s41467-019-14016-1)
Supplement: Supplementary file 2 — Supplementary Information [file 41467_2019_14016_MOESM2_ESM.pdf]

Reaction Scope and Mechanistic Insights of Nickel-Catalyzed Migratory Suzuki-Miyaura Cross-Coupling

Li et al.

Supplementary Methods

All reactions were run under a dry argon atmosphere fitted on a glass tube or vial. All glassware was oven dried at 120 ^{o}C for 2 h and cooled down under vacuum. Thin layer chromatography (TLC) employed glass 0.25 mm silica gel plates. Flash chromatography columns were packed with 300-400 mesh silica gel in petroleum (bp. 60-90 ^{o}C). GC-MS spectra were recorded on a Varian GC-MS 3900-2100T. All new compounds were characterized by ^{1}H NMR, ^{13}C NMR, ^{19}F NMR and HRMS. The known compounds were characterized by ^{1}H NMR, ^{13}C NMR. ^{1}H ^{13}C and ^{19}F NMR data were recorded with Bruker 400 MHz with tetramethylsilane as an internal standard. Data for ^{1}H ^{13}C and ^{19}F NMR are reported as follows: chemical shift (δ ppm), multiplicity (s = singlet, d = doublet, t = triplet, q = quartet, qui = quintet, dd = doublet of doublet, dt = doublet of triplet, dq = doublet of quartet, m = multiplet), integration, and coupling constant (Hz). All chemical shifts (δ) were reported in ppm and coupling constants (*J*) in Hz. All chemical shifts were reported relative to tetramethylsilane (0 ppm for ^{1}H), Chloroform-*d* (77.16 ppm for ^{13}C), respectively. GC analyses were performed on an Agilent 7890B gas chromatograph with an FID detector using a *J* & W DB-1 column (10 m, 0.1 mm I.D.). High resolution mass spectra (HRMS) were measured with a Waters Micromass GCT instrument.

NiI_2 (CAS Nu: 13462-90-3) was purchased from sigma-aldrich. Bathocuproine (BC, CAS Nu: 4733-39-5) and anhydrous DMA were purchased from Adamas-beta®. LiOH, LiOMe and Et_3SiH were purchased from Energy Chemical. KI and *n*-Bu_4NBr (TBAB, CAS Nu:1643-19-2) were purchased from Tokyo Chemical Industry. $LiAlD_4$ (95% D) was purchased from AMEKO. Unless otherwise noted, alkyl acids, alkyl bromides and aryl boric acids were obtained from commercial suppliers (Energy Chemical, Adamas-beta®, *J*&K and so on) and used without further purification.

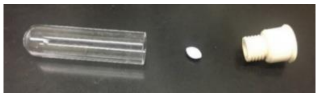

Supplementary Figure 1. Reaction Tube

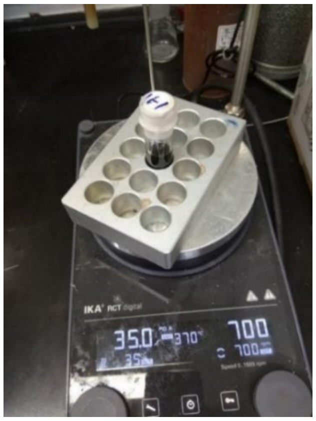

Supplementary Figure 2. Reaction on Process

Supplementary Table 1. Reaction Optimization of Alkyl Tosylates with 2a [a]

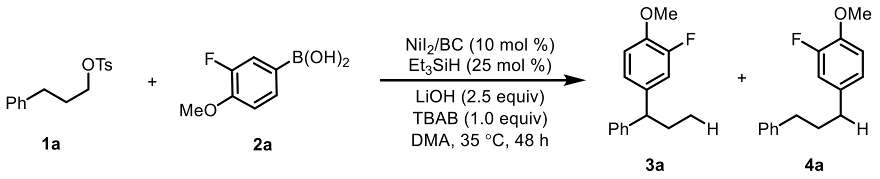

entry	deviation from standard conditions	yield, %	**3a**:**4a**
1	No	89 (82 [b])	27:1
2	no Et_3SiH	6	4:1
3	Zn instand of Et_3SiH	35	12:1
4	Mn instand of Et_3SiH	trace	-
5	$ZnMe_2$ instand of Et_3SiH	trace	-
6	$NaBH_4$ instand of Et_3SiH	trace	-
7	MeMgBr instand of Et_3SiH	trace	-
8	KF instead of LiOH	trace	-
9	Na_2CO_3 instead of LiOH	trace	-
10	Cs_2CO_3 instead of LiOH	trace	-
11	NaOH instead of LiOH	trace	-
12	LiOMe instead of LiOH	trace	-
13	no TBAB	8	2:1
14	$NiCl_2$ instead of NiI_2	trace	-
15	$NiBr_2$ instead of NiI_2	46	21:1
16	$Ni(cod)_2$, no Et_3SiH	12	2:1
17	**L1**	28	2:1
18	**L2**	81	51:1
19	**L3**	78	35:1
20	**L4**	76	1:77
21	*i*-PrOH instead of DMA	0	0
22	THF instead of DMA	0	0
23	Toluene instead of DMA	0	0
24	NMP instead of DMA	67	20:1
25	1.0 equiv H_2O was added	trace	-
26	1.0 equiv *i*-PrOH was added	trace	-
27	LiOH (1.5 equiv)	46	24:1
28	NiI_2(BC) (5 mol %)	72	19:1
29	60 °C, 24 h	71	8:1
30	60 °C, no Et_3SiH	10	6:1

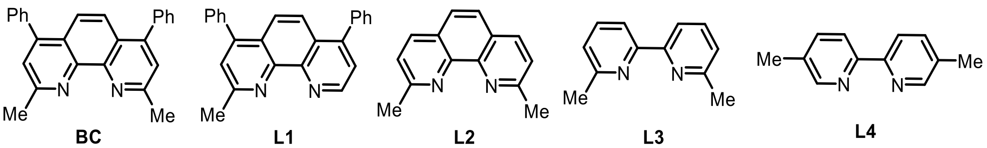

[a] **Standard conditions**: NiI_2 (15.6 mg, 0.05 mmol, 10 mol %), **BC** (18.0 mg, 0.05 mmol, 10 mol %), Et_3SiH (20 μL, 0.13 mmol, 25 mol %), **1a** (145.0 mg, 0.5 mmol, 1.0 equiv), **2a** (127.5 mg, 0.75 mmol, 1.5 equiv), LiOH (29.9 mg, 1.25 mmol, 2.5 equiv), TBAB (161.2 mg, 0.5 mmol, 1.0 equiv), DMA (4 mL). Yields were determined by GC with 1,3,5-trimethoxybenzene as the internal standard. [b] Isolated yield.

Supplementary Table 2. Reaction Optimization of Alkyl Chlorides [a]

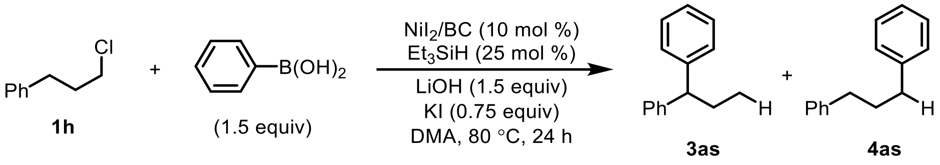

entry	deviation from standard conditions	yield, %	**3as**:**4as**
1	No	78 (70 [b])	13:1
2	no KI	13	20:1
3	KI (50 mol%)	62	15:1
4	KI (100 mol%)	71	10:1
5	LiI instead of KI	42	15:1
6	NaI instead of KI	73	11:1
7	*n*-Bu_4NI instead of KI	33	19:1
8	*n*-Bu_4NBr instead of KI	31	24:1
9	LiOH (2.5 equiv)	53	9:1
10	35 °C for 72 h	10	15:1

[a] **Standard conditions**: NiI_2 (15.6 mg, 0.05 mmol, 10 mol %), **BC** (18.0 mg, 0.05 mmol, 10 mol %), Et_3SiH (20 μL, 0.13 mmol, 25 mol %), alkyl chloride (72 μL, 0.5 mmol, 1.0 equiv), $PhB(OH)_2$ (91.5 mg, 0.75 mmol, 1.5 equiv), LiOH (17.9 mg, 0.75 mmol, 1.5 equiv), DMA (4 mL). Yields were determined by GC with 1,3,5-trimethoxybenzene as the internal standard. [b] Isolated yield.

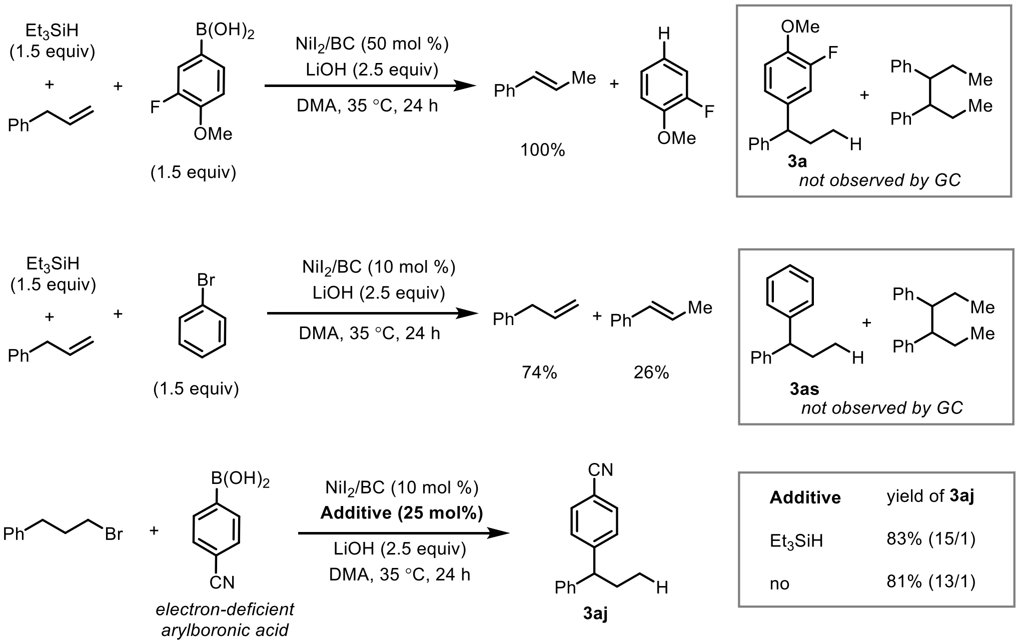

Supplementary Figure 3. Investigation the Role of Et_3SiH

Conclusion: Et_3SiH only play the role of generating the active nickel catalyst from the Ni(II) salt.

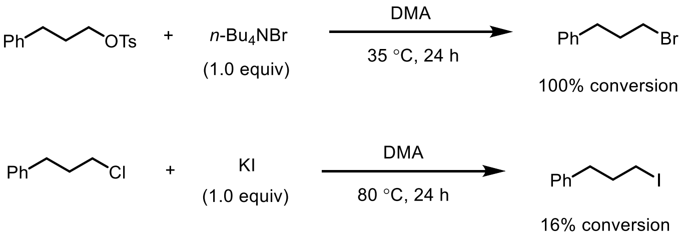

Supplementary Figure 4. Investigation the *in situ* Conversion

Procedure: the two reaction was setup at 0.5 mmol scale in 4.0 mL anhydrous DMA. The conversions were determined by GC analysis. **Notice**: The formation of alkyl-Br and alkyl-I can also be observed in the standard reaction by GC analysis.

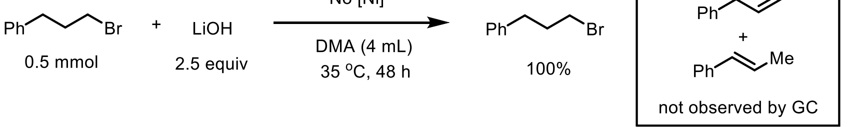

Supplementary Figure 5. Results of without Nickel Catalyst

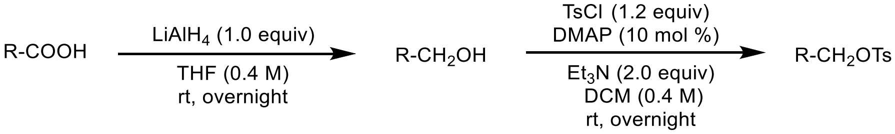

General procedure for the reduction of carboxylic acid: [1] To a stirred solution of $LiAlH_4$ (1.0 equiv) in THF (0.4 M) was added a solution of carboxylic acid (1.0 equiv) in THF dropwise at 0 °C. The mixture was stirred at 0 °C for another 1 h and then allowed to warm to room temperature for 12 h. The reaction progress was monitored by thin-layer chromatography (TLC). The reaction was quenched with 10% NaOH, then the mixture was extracted with EtOAc (3 × 40 mL), and the organic layers were combined and concentrated in vacuo to give a crude material of alcohol, which was used directly in the next step without further purification.

General procedure for the alcohol tosylation: [2] To a solution of corresponding starting alcohol (1.0 equiv) in DCM (0.4 M), TsCl (1.2 equiv), DMAP (10 mol %) and Et_3N (2 equiv) were added. The reaction mixture was stirred rapidly at room temperature for 12 h. The reaction progress was monitored by thin-layer chromatography (TLC). After completion of the reaction, the mixture was extracted with DCM (3 × 40 mL), and the organic layers were combined and concentrated in vacuo to give a crude material of alkyl tosylate. The crude product was purified via flash chromatography over silica gel.

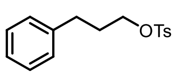

3-Phenylpropyl 4-methylbenzenesulfonate (1a) [3] **(**12.5 g, 86%, a viscous oil.**):** ^{1}H NMR (400 MHz, Chloroform-*d*) δ 7.83 (d, *J* = 8.3 Hz, 2 H), 7.38 (d, *J* = 8.0 Hz, 2 H), 7.29 - 7.19 (m, 3 H), 7.11 - 7.09 (m, 2 H), 4.06 (t, *J* = 6.2 Hz, 2 H), 2.68 (t, *J* = 7.6 Hz, 2 H), 2.49 (s, 3 H), 2.01 - 1.97 (m, 2 H).

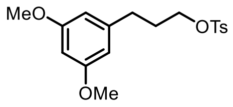

3-(3,5-Dimethoxyphenyl)propyl 4-methylbenzenesulfonate (1j) (4.6 g, 76%, a viscous oil.**):** ^{1}H NMR (400 MHz, Chloroform-*d*) δ 7.79 (d, *J* = 8.3 Hz, 2 H), 7.34 (d, *J* = 8.0 Hz, 2 H), 6.29 (t, *J* = 2.2 Hz, 1 H), 6.26 (d, *J* = 2.2 Hz, 2 H), 4.03 (t, *J* = 6.2 Hz, 2 H), 3.76 (s, 6 H), 2.59 (t, *J* = 7.3 Hz, 2 H), 2.45 (s, 3 H), 1.99 - 1.87 (m, 2 H). ^{13}C NMR (101 MHz, Chloroform-*d*) δ 160.9, 144.9, 142.9, 133.1, 130.0, 128.0, 106.6, 98.2, 69.8, 55.4, 31.9, 30.4, 21.8. HRMS (ESI) Calculated for $C_{18}H_{22}NaO_5S$ ($[M+Na]^+$): 373.1086, measured: 373.1088.

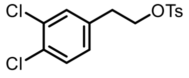

3,4-Dichlorophenethyl 4-methylbenzenesulfonate (1k) (5.4 g, 79%, a white solid.**):** ^{1}H NMR (400 MHz, Chloroform-*d*) δ 7.63 (d, *J* = 8.3 Hz, 2 H), 7.28 - 7.26 (m, 3 H), 7.12 (d, J = 2.0 Hz, 1 H), 6.94 (dd, *J* = 8.2, 2.1 Hz, 1 H), 4.21 (t, *J* = 6.4 Hz, 2 H), 2.90 (t, *J* = 6.4 Hz, 2 H), 2.45 (s, 3 H). ^{13}C NMR (101 MHz, Chloroform-*d*) δ 145.0, 136.7, 132.5, 132.5, 131.0, 130.7, 130.4, 129.8, 128.4, 127.7, 69.9, 34.4, 21.7. HRMS (ESI) Calculated for $C_{15}H_{14}Cl_2NaO_3S$ ($[M+Na]^+$): 366.9938, measured: 366.9942.

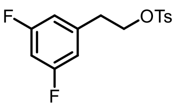

3,5-Difluorophenethyl 4-methylbenzenesulfonate (1l) (4.2 g, 68%, a white solid.**):** ^{1}H NMR (400 MHz, Chloroform-*d*) δ 7.68 (d, *J* = 8.3 Hz, 2 H), 7.30 (d, *J* = 8.1 Hz, 2 H), 6.68 - 6.58 (m, 3 H), 4.21 (t, *J* = 6.5 Hz, 2 H), 2.92 (t, *J* = 6.5 Hz, 2 H), 2.44 (s, 3 H). ^{13}C NMR (101 MHz, Chloroform-*d*) δ163.1 (dd, J = 248.7, 12.8 Hz), 145.1, 140.3 (t, *J* = 9.2 Hz), 132.7, 130.0, 127.9, 110.8 (dd, *J* = 18.4, 6.7 Hz), 102.5 (t, *J* = 25.2 Hz), 69.7, 35.1, 21.7. ^{19}F NMR (377 MHz, Chloroform-*d*) δ -109.81. HRMS (ESI) Calculated for $C_{15}H_{14}F_2NaO_3S$ ($[M+Na]^+$): 335.0529, measured: 335.0532.

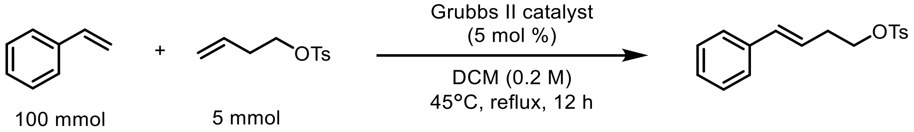

(*E*)-4-phenylbut-3-en-1-yl 4-methylbenzenesulfonate (1m) [2] **(**1.1 g, 73%, a viscous oil.**):** Into a dry 100 mL round-bottom flask equipped with a stirbar were added homoallyl tosylate (1.2 g, 5 mmol, 1.0 equiv), styrene (11.5 mL, 100 mmol, 20 equiv), and Grubbs's 2nd generation catalyst (332.4 mg, 1 mmol, 5 mol %) in 25 mL of DCM. The flask was equipped with a condenser and refluxed for 12 h at 45 °C under N_2. The reaction progress was monitored by thin-layer chromatography (TLC). After completion of the reaction, the mixture was cooled to ambient temperature and filtered by diatomite to remove Grubbs's 2nd generation catalyst. Then the organic layer was concentrated in vacuo to give a crude product, which was purified via flash chromatography over silica gel. ^{1}H NMR (400 MHz, Chloroform-*d*) δ 7.78 (d, *J* = 7.8 Hz, 2 H), 7.32 - 7.28 (m, 4 H), 7.27 (s, 2 H), 7.22 (t, *J* = 7.1 Hz, 1 H), 6.39 (d, *J* = 15.9 Hz, 1 H), 6.00 (dd, *J* = 15.2, 7.6 Hz, 1 H), 4.14 (t, *J* = 6.6 Hz, 2 H), 2.55 (q, *J* = 6.8 Hz, 2 H), 2.42 (s, 3 H).

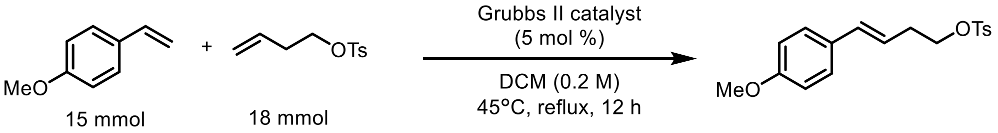

(*E*)-4-(4-methoxyphenyl)but-3-en-1-yl 4-methylbenzenesulfonate (1n) [2] **(**1.2 g, 25%, a viscous oil.**):** Into a dry 200 mL round-bottom flask equipped with a stirbar were added homoallyl tosylate (4.1 g, 18 mmol, 1.2 equiv), 1-methoxy-4-vinylbenzene (2.0 mL, 15 mmol, 1.0 equiv), and Grubbs's 2nd generation catalyst (636.8 mg, 0.75 mmol, 5 mol %) in 75 mL of DCM. The flask was equipped with a condenser and refluxed for 12 h at 45 °C under N_2. The reaction progress was monitored by thin-layer chromatography (TLC). After completion of the reaction, the mixture was cooled to ambient temperature and filtered by diatomite to remove Grubbs's 2nd generation catalyst. Then the organic layer was concentrated in vacuo to give a crude product, which was purified via flash chromatography over silica gel. ^{1}H NMR (400 MHz, Chloroform-*d*) δ 7.78 (d, *J* = 7.8 Hz, 2 H), 7.30 (d, *J* = 7.9

Hz, 2 H), 7.20 (d, J = 8.2 Hz, 2 H), 6.83 (d, J = 8.2 Hz, 2 H), 6.33 (d, J = 15.9 Hz, 1 H), 5.85 (dt, J = 15.1, 7.0 Hz, 1 H), 4.12 (t, J = 6.7 Hz, 2 H), 3.80 (s, 3 H), 2.53 (q, J = 6.9 Hz,2 H), 2.42 (s, 3 H).

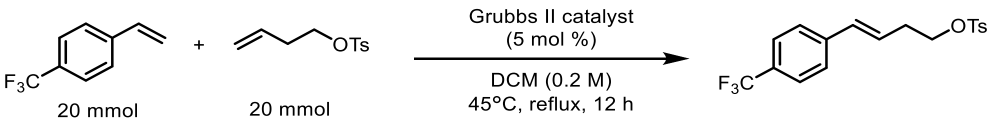

(*E*)-4-(4-(trifluoromethyl)phenyl)but-3-en-1-yl 4-methylbenzenesulfonate (1o) [2] **(**1.7 g, 23%, a viscous oil.**):** Into a dry 200 mL round-bottom flask equipped with a stirbar were added homoallyl tosylate (1.0 equiv, 20 mmol, 4.5 g), 1-methoxy-4-vinylbenzene (1.0 equiv, 20 mmol, 3.0 mL), and Grubbs's 2nd generation catalyst (849.0 mg, 1 mmol, 5 mol %) in 100 mL of DCM. The flask was equipped with a condenser and refluxed for 12 h at 45 °C under N_2. The reaction progress was monitored by thin-layer chromatography (TLC). After completion of the reaction, the mixture was cooled to ambient temperature and filtered by diatomite to remove Grubbs's 2nd generation catalyst. Then the organic layer was concentrated in vacuo to give a crude product, which was purified via flash chromatography over silica gel. ^{1}H NMR (400 MHz, Chloroform-*d*) δ 7.78 (d, J = 7.9 Hz, 2 H), 7.54 (d, J = 8.0 Hz, 2 H), 7.36 (d, J = 8.0 Hz, 2 H), 7.30 (d, J = 8.0 Hz, 2 H), 6.43 (d, J = 15.9 Hz, 1 H), 6.12 (dt, J = 15.1, 7.0 Hz, 1 H), 4.16 (t, J = 6.4 Hz, 2 H), 2.59 (q, J = 6.6 Hz, 2 H), 2.42 (s, 3 H).

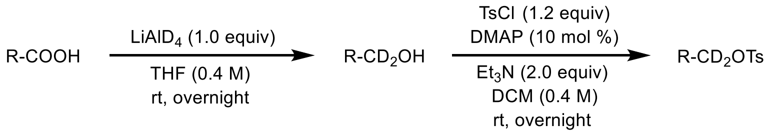

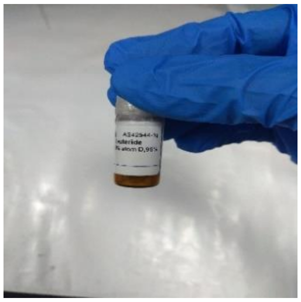

Supplementary Figure 6. Source of $LiAlD_4$

General procedure for the reduction of carboxylic acid: [1] Under N_2 condition, to a stirred solution of $LiAlD_4$ (1.0 equiv, 95% D) in anhydrous THF (0.4 M) was added a solution of carboxylic acid (1.0 equiv) in THF dropwise at 0 °C. The mixture was stirred at 0 °C for another 1h and then allowed to warm to room temperature for 12 h. The reaction progress was monitored by thin-layer chromatography (TLC).The reaction was quenched with 10% NaOH, then the mixture was extracted with EtOAc (3 × 40 mL), and the organic layers were combined and concentrated in vacuo to give a crude material of alcohol, which was used without further purification.

General procedure for the alcohol tosylation: [2] To a solution of corresponding starting alcohol (1.0 equiv) in DCM (0.4 M), TsCl (1.2 equiv), DMAP (10 mol %) and Et_3N (2 equiv) were added. The reaction mixture was stirred rapidly at room temperature for 12 h. The reaction progress was monitored by thin-layer chromatography (TLC). After completion of the reaction, the mixture was extracted with DCM (3 × 40 mL), and the organic layers were combined and concentrated in vacuo to give a crude material of alkyl tosylate. The crude product was purified via flash chromatography over silica gel.

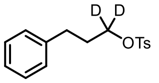

3-Phenylpropyl-1, 1-*d*₂ 4-methylbenzenesulfonate (1a-D₂') [1] **(**0.6 g, 67%, 94% D**):** A viscous oil. ^{1}H NMR (400 MHz, Chloroform-*d*) δ 7.80 - 7.78 (m, 2 H), 7.35 (d, *J* = 8.0 Hz, 2 H), 7.26 - 7.22 (m, 2 H), 7.19 - 7.15 (m, 1 H), 7.08 - 7.06 (m, 2 H), 2.64 (t, *J* = 7.6 Hz, 2 H), 2.46 (s, 3 H), 1.94 (t, *J* = 7.6 Hz, 2 H). ^{13}C NMR (101 MHz, Chloroform-*d*) δ 144.9, 140.5, 133.2, 130.0, 128.6, 128.5, 128.0, 126.3, 31.5, 30.4, 21.8. HRMS (ESI) Calculated for $C_{16}H_{16}D_2NaO_3S$ ($[M+Na]^+$): 315.1000, measured: 315.1004.

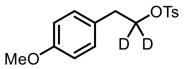

2-(4-Methoxyphenyl)ethyl-1, 1-*d*₂ 4-methylbenzenesulfonate (1o-D₂') (2.2 g, 72%, 95% D**):** A viscous. ^{1}H NMR (400 MHz, Chloroform-*d*) δ 7.70 - 7.67 (m, 2 H), 7.29 - 7.27 (m, 2 H), 7.04 - 7.00 (m, 2 H), 6.80 - 6.76 (m, 2 H), 3.78 (s, 3 H), 2.88 (s, 2 H), 2.43 (s, 3 H). ^{13}C NMR (101 MHz, Chloroform-*d*) δ 158.6, 144.8, 133.1, 130.0, 129.9, 128.2, 127.9, 114.1, 55.4, 34.4, 21.8. HRMS (ESI) Calculated for $C_{16}H_{16}D_2NaO_4S$ ($[M+Na]^+$): 331.0949, measured: 331.0953.

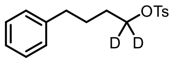

4-Phenylbutyl-1,1-d₂ 4-methylbenzenesulfonate (1p-D₂')(0.86 g, 56%, 94% D**):** A white oil. ^{1}H NMR (400 MHz, Chloroform-*d*) δ 7.79 - 7.76 (m, 2 H), 7.33 (d, *J* = 8.1 Hz, 2 H), 7.28 - 7.24 (m, 2 H), 7.20 - 7.15 (m, 1 H), 7.11 - 7.09 (m, 2 H), 2.57 - 2.54 (m, 2 H), 2.44 (s, 3 H), 1.67 - 1.62 (m, 4 H). ^{13}C NMR (101 MHz, Chloroform-*d*) δ 144.8, 141.7, 133.2, 129.9, 128.46, 128.45, 128.0, 126.0, 35.2, 28.2, 27.1, 21.8. HRMS (ESI) Calculated for $C_{17}H_{18}D_2NaO_3S$ ($[M+Na]^+$): 329.1156, measured: 329.1146.

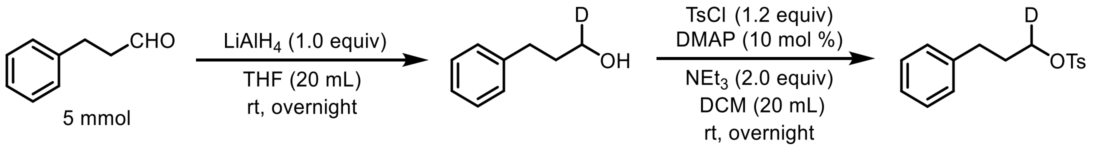

Procedure for the reduction of 3-phenylpropanal: [1] To a stirred solution of $LiAlD_4$ (210 mg, 5 mmol, 1.0 equiv) in THF (20 mL) was added a solution of 3-phenylpropanal (0.7 mL, 5 mmol, 1.0 equiv) in THF dropwise at 0 °C. The mixture was stirred at 0 °C for another 1 h and then allowed to warm to room temperature for 12 h. The reaction progress was monitored by thin-layer chromatography (TLC).The reaction was quenched with 10% NaOH, then the mixture was extracted with EtOAc (3 × 40 mL), and the organic layers were combined and

concentrated in vacuo to give a crude material of 3-phenylpropan-1-*d*-1-ol, which was used without further purification.

Procedure for 3-phenylpropan-1-*d*-1-ol tosylation : [2] To a solution of 3-phenylpropan-1-d-1-ol (0.7 mL, 5 mmol, 1.0 equiv) in DCM (20 mL), TsCl (1.2 g, 6 mmol, 1.2 equiv), DMAP (61 mg, 0.5 mmol, 10 mol %) and Et_3N (1.4 mL, 10 mmol, 2 equiv) were added. The reaction mixture was stirred rapidly at room temperature for 12 h. The reaction progress was monitored by thin-layer chromatography (TLC). After completion of the reaction, the mixture was extracted with DCM (3 × 40 mL), and the organic layers were combined and concentrated in vacuo to give a crude material. The crude product was purified via flash chromatography over silica gel.

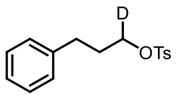

3-Phenylpropyl-1-*d* 4-methylbenzenesulfonate (1a-D$_1$)(1.1 g, 76%, 95% D)**:** A viscous oil. ^{1}H NMR (400 MHz, Chloroform-*d*) δ 7.80 - 7.77 (m, 2 H), 7.36 - 7.32 (m, 2 H), 7.26 - 7.21 (m, 2 H), 7.19 - 7.15 (m, 1 H), 7.08 - 7.05 (m, 2 H), 4.02 (q, *J* = 6.3 Hz, 1 H), 2.64 (t, *J* = 7.6 Hz, 2 H), 2.45 (s, 3 H), 1.97 - 1.92 (m, 2 H). ^{13}C NMR (101 MHz, Chloroform-*d*) δ 144.9, 140.5, 133.2, 130.0, 128.6, 128.5, 128.0, 126.2, 69.4 (t, *J* = 23.0 Hz), 31.5, 30.4, 21.8. HRMS (ESI) Calculated for $C_{16}H_{17}DNaO_3S$ ($[M+Na]^+$): 314.0937, measured: 314.0939.

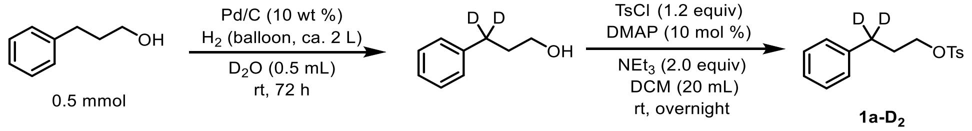

1a-D$_2$

Synthesis of 3-phenylpropan-3, 3-*d*$_2$-1-ol : [4] To a stirred solution of 10% Pd/C (10 wt % of the substrate) in D_2O (2.5 mL) was added a solution of 3-phenylpropan-1-ol (0.7 mL, 5 mmol, 1.0 equiv) dropwise at room temperature in a sealed test tube filled with hydrogen gas (2 L). The mixture was stirred at room temperature for 72 h. The reaction progress was monitored by thin-layer chromatography (TLC). The mixture was diluted with EtOAc (10 mL), then filtered using a membrane filter to remove the catalyst. The filtrate was partitioned between EtOAc and aqueous layers.Then the mixture was extracted with EtOAc (3 × 40 mL), and the organic layers were combined and concentrated in vacuo to give a crude material of 3-phenylpropan-3, 3-*d*$_2$-1-ol, which was used without further purification.

Synthesis of 3-phenylpropyl-3, 3-*d*$_2$ 4-methylbenzenesulfonate （**1a-D**$_2$, 83% D）**:** To a solution of 3-phenylpropan-3, 3-*d*$_2$-1-ol (1.0 equiv, 5 mmol) in DCM (20 mL), TsCl (1.2 g, 6 mmol, 1.2 equiv), DMAP (61 mg, 0.5 mmol, 10 mol %) and Et_3N (1.4 mL, 10 mmol, 2 equiv) were added. The reaction mixture was stirred rapidly at room temperature for 12 h. The reaction progress was monitored by thin-layer chromatography (TLC). After completion of the reaction, the mixture was extracted with DCM (3 × 40 mL), and the organic layers were

combined and concentrated in vacuo to give a crude material of 3-phenylpropyl-3, 3-d_2 4-methylbenzenesulfonate. The crude product was purified via flash chromatography over silica gel offer 1.2 g **1a-D₂** as a viscous oil, 79% yield. ^{1}H NMR (400 MHz, Chloroform-*d*) δ 7.79 (d, *J* = 8.3 Hz, 2 H), 7.35 (d, *J* = 8.0 Hz, 2 H), 7.27 - 7.21 (m, 2 H), 7.20 - 7.14 (m, 1 H), 7.09 - 7.03 (m, 2 H), 4.02 (t, *J* = 6.2 Hz, 2 H), 2.46 (s, 3 H), 1.94 (t, *J* = 6.3 Hz, 2 H). ^{13}C NMR (101 MHz, Chloroform-*d*) δ 144.9, 140.4, 133.1, 130.0, 128.6, 128.5, 128.0, 126.3, 69.7, 30.5, 30.4, 21.8. HRMS (ESI) Calculated for $C_{16}H_{16}D_2NaO_3S$ ([M+Na]$^+$): 315.1000, measured: 315.1004.

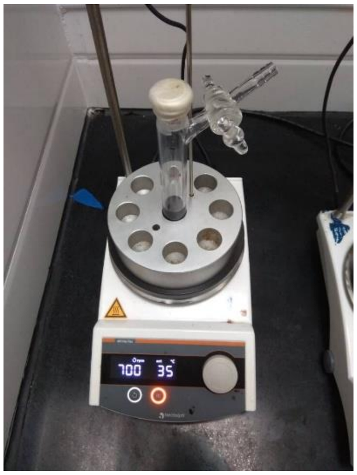

Supplementary Figure 7. Reactiom on process

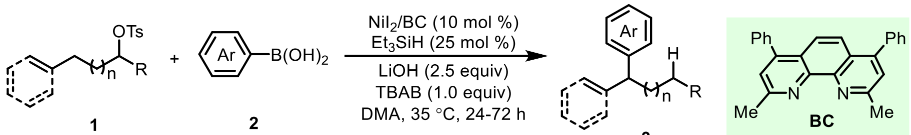

1 2 3

Procedure A: Under N_2 atmosphere, into an oven-dried 10 mL reaction tube equipped with a magnetic stir bar and sealed with a rubber stopper sequentially added NiI_2 (15.6 mg, 0.05 mmol, 10 mol %), BC (18.0 mg, 0.05 mmol, 10 mol %), anhydrous DMA (4 mL) and Et_3SiH (20 µL, 0.13 mmol, 25 mol %). The mixture was stirred at 35 ℃ for 30 min, then TBAB (161.2 mg, 0.5 mmol, 1.0 equiv), LiOH (29.9 mg, 1.25 mmol, 2.5 equiv), alkyl tosylate (0.5 mmol, 1 equiv) and aryl boronic acid (0.75 mmol, 1.5 equiv) were added in this order. The resulting mixture was stirred at 35 ℃ (if aryl boronic pinacol ester was used, stirred at 60 ^{o}C) and monitored by GC until the alkyl tosylate disappeared. After the reaction was complete, the mixture was quenched by saturated brine and extracted with ethyl acetate (20 mL × 3). The combined organic layers were dried over Na_2SO_4 and concentrated under reduced pressure. The resulting crude product was separated on a silica gel column affording the cross-coupling product.

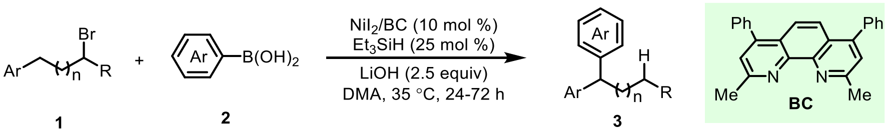

Procedure B: Without TBAB additive, otherwise are same as procedure **A**.

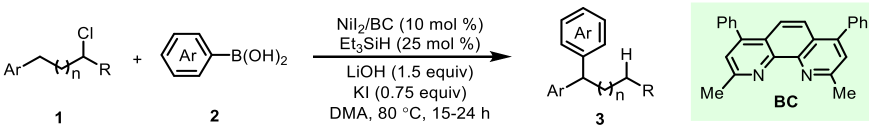

Procedure C: KI (62.3 mg, 0.38 mmol, 0.75 equiv), LiOH (17.9 mg, 0.75 mmol, 1.5 equiv), the resulting mixture was stirred at 80 ℃. Otherwise are same as procedure **A**.

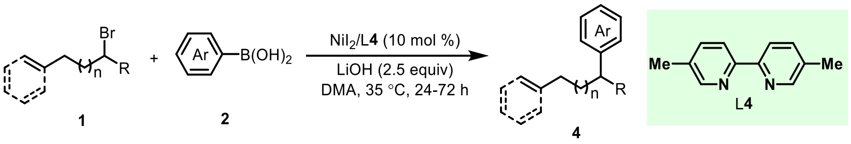

Procedure D: Without TBAB and Et_3SiH additive, L**4** instead of of BC, otherwise are same as procedure **A**.

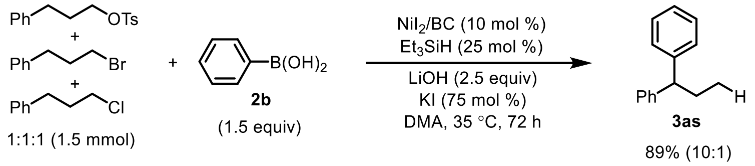

Supplementary Figure 8. Convergent Synthesis

Under N_2 atmosphere, into an oven-dried 20 mL reaction tube equipped with a magnetic stir bar and sealed with a rubber stopper sequentially added NiI_2 (46.8 mg, 0.15 mmol, 10 mol %), BC (54.0 mg, 0.15 mmol, 10 mol %), Et_3SiH (60 μL, 0.38 mmol, 25 mol %) and anhydrous DMA (4 mL). The mixture was stirred at 35 ℃ for 30 min, then KI (186.8 mg, 1.13 mmol, 0.75 equiv), LiOH (89.7 mg, 3.75 mmol, 2.5 equiv), alkyl tosylate (145.0 mg, 0.5 mmol), alkyl bromide (76 μL, 0.5 mmol), alkyl chloride (72 μL, 0.5 mmol) and aryl acid 2b (274.5 mg, 2.25 mmol, 1.5 equiv) were added to the resulting mixture in this order. The resulting mixture was stirred at 35 ℃ and monitored by GC until the alkyl chloride disappeared. After the reaction was complete, the mixture was quenched by saturated brine and extracted with ethyl acetate (40 mL × 3). The combined organic layers were dried over Na_2SO_4 and concentrated under reduced pressure. The resulting crude product was separated on a silica gel column, affording the cross-coupling product.

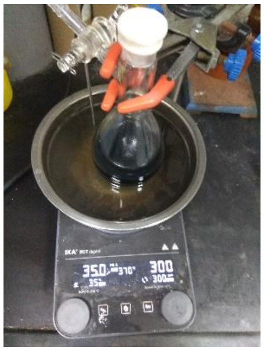

Supplementary Figure 9. Reaction on process

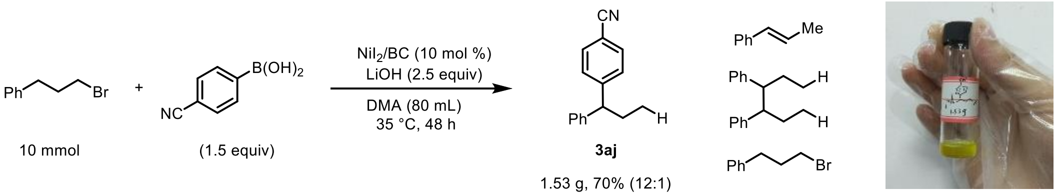

Supplementary Figure 10. Gram Scale

Under N_2 atmosphere, into an oven-dried 200 mL schlenk flask equipped with a magnetic stir bar and sealed with a rubber stopper sequentially added NiI_2 (312 mg, 1 mmol, 10 mol %), BC (360 mg, 1 mmol, 10 mol %), LiOH (600 mg, 25 mmol, 2.5 equiv), (3-bromopropyl)benzene (1.52 mL, 10 mmol), (4-cyanophenyl)boronic acid (2.21 g, 15 mmol, 1.5 equiv) and DMA (80 mL) were added to the resulting mixture in this order. The resulting mixture was stirred at 35 ℃ and monitored by GC until the alkyl bromoride disappeared. After the reaction was complete, the mixture was quenched by saturated brine and extracted with ethyl acetate (40 mL × 3). The combined organic layers were dried over Na_2SO_4 and concentrated under reduced pressure. The resulting crude product was separated on a silica gel column, affording the cross-coupling product (1.53 g, 70%).

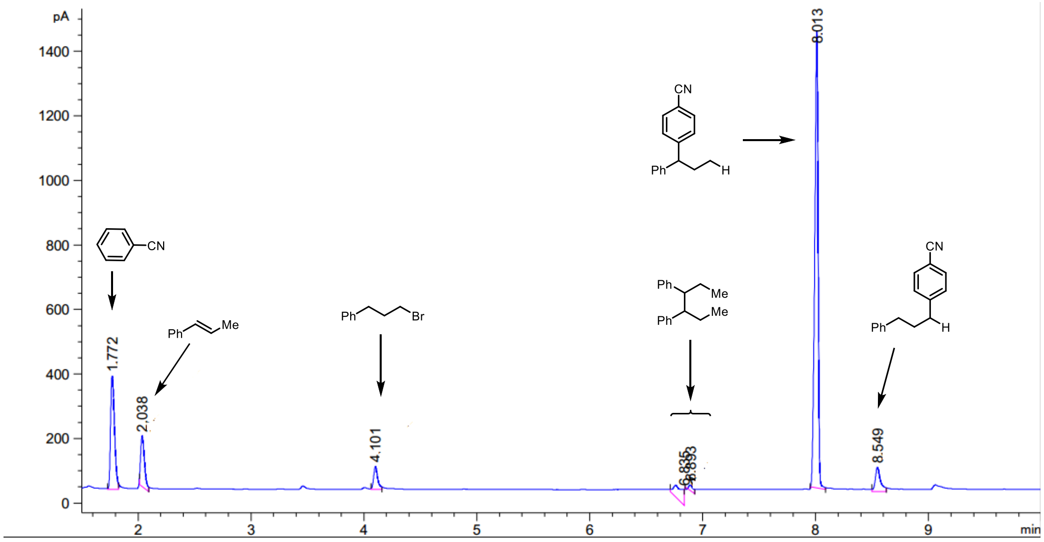

Supplementary Figure 11. Crude GC Spectrum

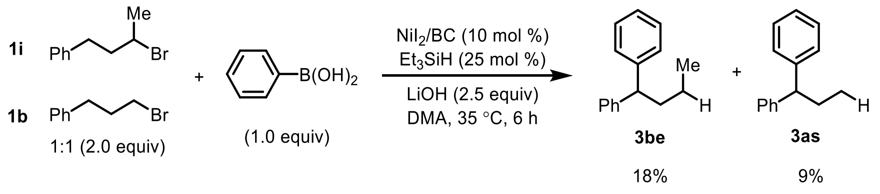

Supplementary Figure 12. Competition Experiment

In glovebox, into an oven-dried 10 mL reaction tube equipped with a magnetic stir bar and sealed with a rubber stopper sequentially added NiI_2 (15.6 mg, 0.05 mmol, 10 mol %), BC (18.0 mg, 0.05 mmol, 10 mol %), anhydrous DMA (4 mL) and Et_3SiH (20 μL, 0.13 mmol, 25 mol %). The mixture was stirred at 35 °C for 30 min. Then LiOH (29.9 mg, 1.25 mmol, 2.5 equiv), (3-bromopropyl)benzene **1b** (76 μL, 0.5 mmol, 1 equiv), (3-bromobutyl)benzene **1i** (86 μL,0.5 mmol, 1 equiv) and phenylboronic acid (61.0 mg, 0.5 mmol, 1.0 equiv) were added to the resulting mixture in this order. The tube was sealed with a rubber stopper, stirred at 35 °C. The reaction progress was monitored by GC with 1, 3, 5-trimethoxybenzene as the internal standard.

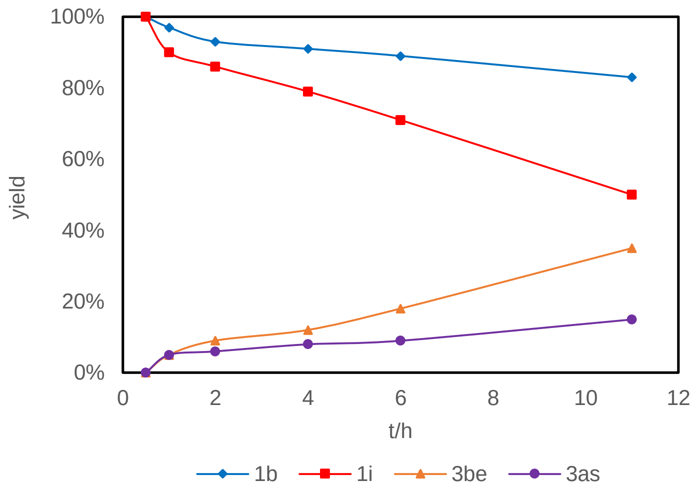

Supplementary Figure 13. Time Course of Figure 12

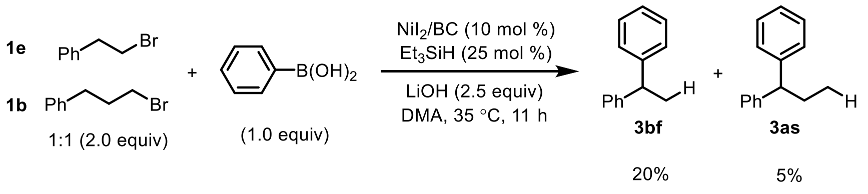

Supplementary Figure 14. Competition Experiment

In glovebox, into an oven-dried 10 mL reaction tube equipped with a magnetic stir bar and sealed with a rubber stopper sequentially added NiI_2 (15.6 mg, 0.05 mmol, 10 mol %), BC (18.0 mg, 0.05 mmol, 10 mol %), anhydrous DMA (4 mL) and Et_3SiH (20 μL, 0.13 mmol, 25 mol %). The mixture was stirred at 35 °C for 30 min. Then LiOH

(29.9 mg, 1.25 mmol, 2.5 equiv), (2-bromoethyl)benzene **1e** (68 μL, 0.5 mmol, 1 equiv), (3-bromopropyl)benzene **1b** (76 μL,0.5 mmol, 1 equiv) and phenylboronic acid (61.0 mg, 0.5 mmol, 1.0 equiv) were added to the resulting mixture in this order. The tube was sealed with a rubber stopper, stirred at 35 °C. The reaction progress was monitored by GC with 1, 3, 5-trimethoxybenzene as the internal standard.

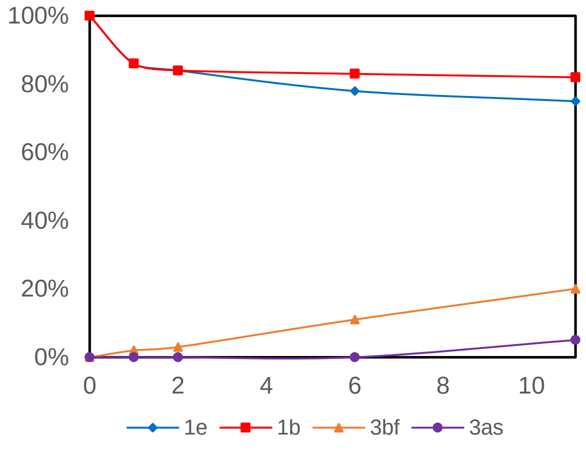

Supplementary Figure 15. Time Course of Figure 14

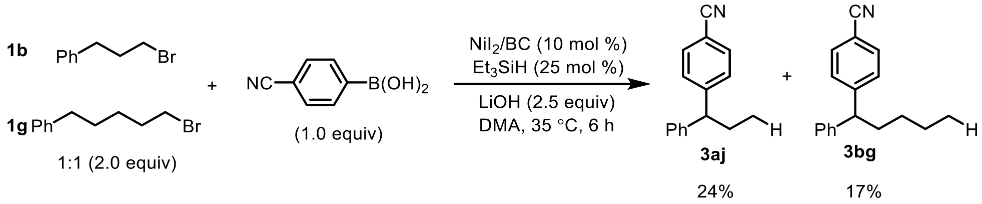

Supplementary Figure 16. Competition Experiment

Competition Experiments: In glovebox, NiI_2 (15.6 mg, 0.05 mmol, 10 mol %), BC (18.0 mg, 0.05 mmol, 10 mol %), and Et_3SiH (20 μL, 0.13 mmol, 25 mol %), anhydrous DMA (4 mL) were added into an oven-dried 10 mL reaction tube equipped with a magnetic stir bar, and the mixture was stirred at 35 °C for 30 min. Then LiOH (29.9 mg, 1.25 mmol, 2.5 equiv), alkyl bromide **1b** (76 μL, 0.5 mmol, 1 equiv), alkyl bromide **1g** (92 μL, 0.5 mmol, 1 equiv) and 4-cyanophenylboronic acid (73.5 mg, 0.5 mmol, 1.0 equiv) were added to the resulting mixture in this order. The tube was sealed with a rubber stopper, stirred at 35 °C. The reaction progress was monitored by GC with 1,3,5-trimethoxybenzene as the internal standard.

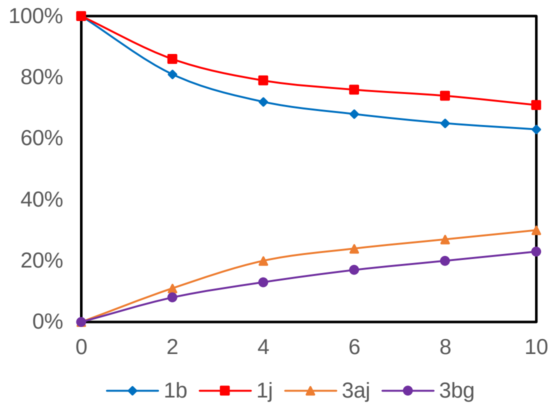

Supplementary Figure 17. Time Course of Figure 16

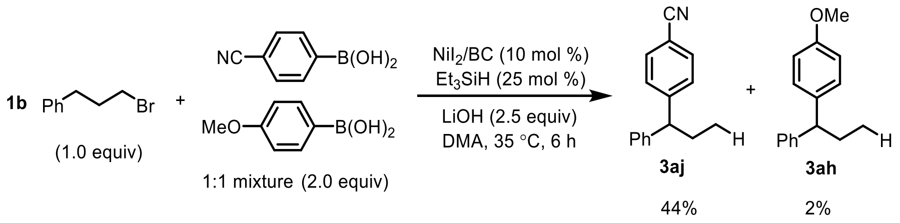

Supplementary Figure 18. Competition Experiment

In glovebox, into an oven-dried 10 mL reaction tube equipped with a magnetic stir bar and sealed with a rubber stopper sequentially added NiI_2 (15.6 mg, 0.05 mmol, 10 mol %), BC (18.0 mg, 0.05 mmol, 10 mol %), anhydrous DMA (4 mL) and Et_3SiH (20 μL, 0.13 mmol, 25 mol %). The mixture was stirred at 35 °C for 30 min. Then LiOH (29.9 mg, 1.25 mmol, 2.5 equiv), (3-bromopropyl)benzene (76 μL,0.5 mmol, 1 equiv), (4-methoxyphenyl)boronic acid (76.0 mg, 0.5 mmol, 1.0 equiv) and (4-cyanophenyl)boronic acid (73.5 mg, 0.5 mmol, 1.0 equiv) were added to the resulting mixture in this order. The tube was sealed with a rubber stopper, stirred at 35 °C. The reaction progress was monitored by GC with 1, 3, 5-trimethoxybenzene as the internal standard.

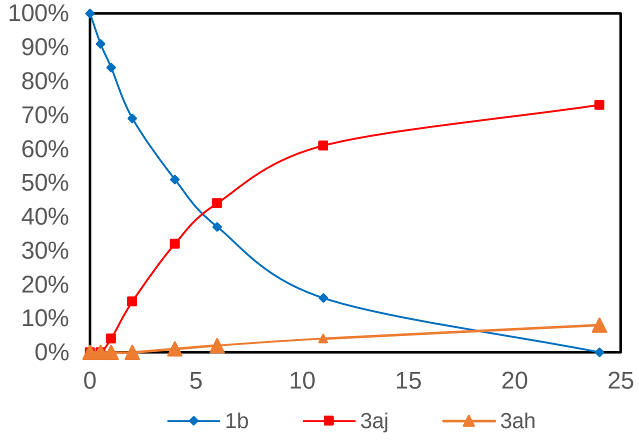

Supplementary Figure 19. Time Course of Figure 18

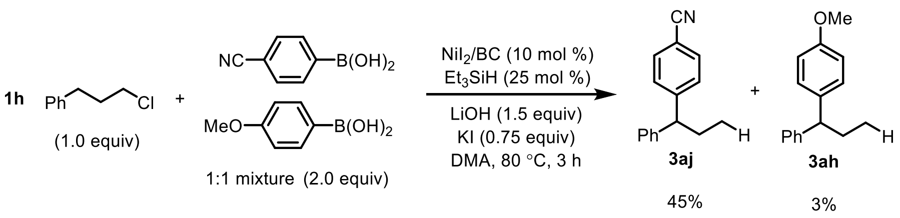

Supplementary Figure 20. Competition Experiment

Under N_2 atmosphere, into an oven-dried 10 mL reaction tube equipped with a magnetic stir bar and sealed with a rubber stopper sequentially added NiI_2 (15.6 mg, 0.05 mmol, 10 mol %), BC (18.0 mg, 0.05 mmol, 10 mol %), anhydrous DMA (4 mL) and Et_3SiH (20 μL, 0.13 mmol, 25 mol %). The mixture was stirred at 35 °C for 30 min. Then KI (62.3 mg, 0.38 mmol, 0.75 equiv), LiOH (18.0 mg, 0.75 mmol, 1.5 equiv), (3-chloropropyl)benzene **1h** (72 μL, 0.5 mmol, 1 equiv), (4-methoxyphenyl)boronic acid (76.0 mg, 0.5 mmol, 1.0 equiv) and (4-cyanophenyl)boronic acid (73.5 mg, 0.5 mmol, 1.0 equiv) were added to the resulting mixture in this order. The tube was sealed with a rubber stopper, stirred at 80 °C. The reaction progress was monitored by GC with 1, 3, 5-trimethoxybenzene as the internal standard.

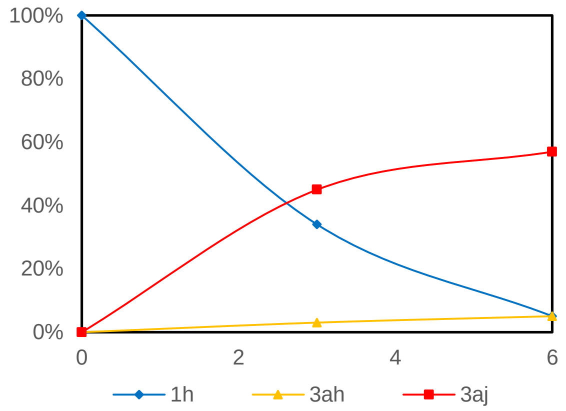

Supplementary Figure 21. Time Course of Figure 20

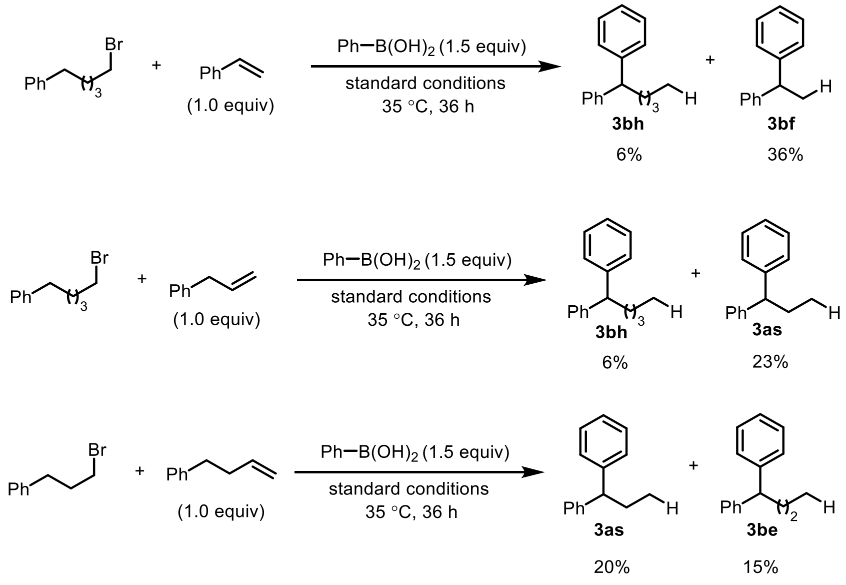

Supplementary Figure 22. Competition experiments of olefin additives

Competition Experiments of Olefin Additives: In glovebox, into an oven-dried 10 mL reaction tube equipped with a magnetic stir bar and sealed with a rubber stopper sequentially added NiI_2 (15.6 mg, 0.05 mmol, 10 mol %), BC (18.0 mg, 0.05 mmol, 10 mol %), anhydrous DMA (4 mL) and Et_3SiH (20 μL, 0.13 mmol, 25 mol %). The mixture was stirred at 35 °C for 30 min. Then LiOH (29.9 mg, 1.25 mmol, 2.5 equiv), alkyl bromide (0.5 mmol, 1 equiv), alkene (0.5 mmol, 1 equiv) and phenylboronic acid (91.5 mg, 0.75 mmol, 1.5 equiv) were added to the resulting mixture in this order. The tube was sealed with a rubber stopper, stirred at 35 °C. The tube was sealed with a rubber stopper, stirred at 35 °C. The reaction progress was monitored by GC with 1, 3, 5-trimethoxybenzene as the internal standard.

Conclusion: The substrates with shorter carbon chain react faster than longer ones, and olefin can dissociate from the nickel intermediate during chain-walking.

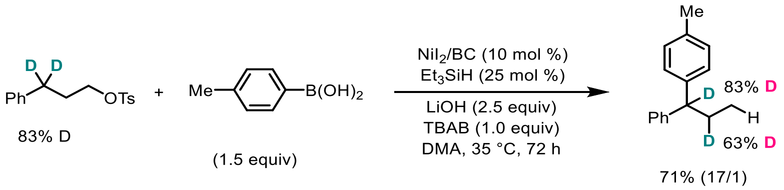

Supplementary Figure 23. D-labelled experiment

1-Methyl-4-(1-phenylpropyl-1, 2-d$_2$)benzene (3bb): Under N_2 atmosphere, into an oven-dried 20 mL reaction tube equipped with a magnetic stir bar and sealed with a rubber stopper sequentially added NiI_2 (15.6 mg, 0.05 mmol, 10 mol %), BC (18.0 mg, 0.05 mmol, 10 mol %), and Et_3SiH (20 μL, 0.13 mmol, 25 mol %), anhydrous DMA (4 mL). The mixture was stirred at 35 °C for 30 min. Then TBAB (161.2 mg, 0.5 mmol, 1.0 equiv), LiOH

(29.9 mg, 1.25 mmol, 2.5 equiv), D-labeled alkyl tosylate (146.0 mg, 0.5 mmol, 1 equiv) and aryl boric acid (102.1 mg, 0.75 mmol, 1.5 equiv) were added to the resulting mixture in this order. The resulting mixture was stirred at 35 ℃ and monitored by GC. After the reaction was complete, the mixture was quenched by saturated brine and extracted with ethyl acetate (40 mL × 3). The combined organic layers were dried over Na_2SO_4 and concentrated under reduced pressure. The resulting crude product was separated on a silica gel column affording the cross-coupling product 75.3 mg as a colorless oil, 71%, *rr* = 17:1. ^{1}H NMR (400 MHz, Chloroform-*d*) δ 7.28 - 7.21 (m, 4 H), 7.17 - 7.07 (m, 5 H), 2.29 (s, 3 H), 2.07 - 2.00 (m, 1 H), 0.89 (t, *J* = 7.2 Hz, 3 H). ^{13}C NMR (101 MHz, Chloroform-*d*) δ 145.5, 142.2, 135.6, 129.2, 128.5, 128.0, 127.9, 126.0, 53.0 - 52.9 (m), 28.8 - 28.3 (m), 21.1, 12.9 - 12.8 (m). HRMS (ESI) Calculated for $C_{16}H_{16}D_2Na$ ($[M+Na]^+$): 235.1432, measured: 235.1434.

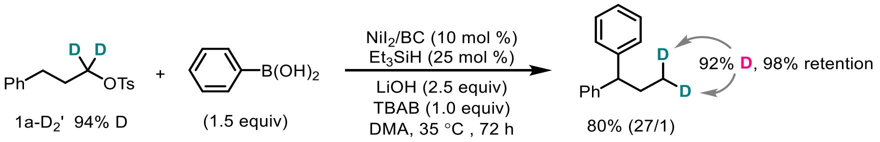

Supplementary Figure 24. D-labelled Experiment

(Propane-1, 1-diyl-3, 3-d$_2$)dibenzene (3as-D$_2$'): Under N_2 atmosphere, into an oven-dried 20 mL reaction tube equipped with a magnetic stir bar and sealed with a rubber stopper sequentially added NiI_2 (15.6 mg, 0.05 mmol, 10 mol %), **BC** (18.0 mg, 0.05 mmol, 10 mol %), and Et_3SiH (20 μL, 0.13 mmol, 25 mol %), anhydrous DMA (4 mL). The mixture was stirred at 35 °C for 30 min. Then TBAB (161.2 mg, 0.5 mmol, 1.0 equiv), LiOH (29.9 mg, 1.25 mmol, 2.5 equiv), D-labeled alkyl tosylate (146.0 mg, 0.5 mmol, 1 equiv) and phenylboronic acid (91.5 mg, 0.75 mmol, 1.5 equiv) were added to the resulting mixture in this order. The resulting mixture was stirred at 35 ℃ and monitored by GC. After the reaction was complete, the mixture was quenched by saturated brine and extracted with ethyl acetate (40 mL × 3). The combined organic layers were dried over Na_2SO_4 and concentrated under reduced pressure. The resulting crude product was separated on a silica gel column affording the cross-coupling product **3as-D$_2$'** 72.9 mg as a colorless oil, 80% yield, *rr* = 27:1. ^{1}H NMR (400 MHz, Chloroform-*d*) δ 7.29 - 7.22 (m, 8 H), 7.18 - 7.14 (m, 2 H), 3.78 (t, *J* = 7.8 Hz, 1 H), 3.78 (dd, *J* = 7.8 Hz, 2 H), 0.91 - 0.84 (m, 1 H).^{13}C NMR (101 MHz, Chloroform-*d*) δ 145.3, 128.5, 128.0, 126.1, 53.3, 28.6, 12.4 (qui, *J* = 19.3 Hz). HRMS (ESI) Calculated for $C_{15}H_{14}D_2Na$ ($[M+Na]^+$): 221.1270, measured: 221.1270.

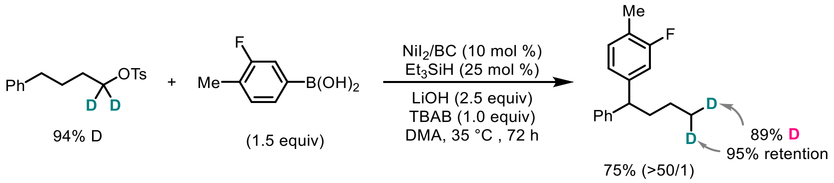

Supplementary Figure 25. D-labelled Experiment

2-Fluoro-1-methyl-4-(1-phenylbutyl-4,4-d$_2$)benzene (3bi-D$_2$): Under N_2 atmosphere, into an oven-dried 20 mL reaction tube equipped with a magnetic stir bar and sealed with a rubber stopper sequentially added NiI_2 (15.6 mg, 0.05 mmol, 10 mol %), **BC** (18.0 mg, 0.05 mmol, 10 mol %), and Et_3SiH (20 μL, 0.13 mmol, 25 mol %), anhydrous DMA (4 mL). The mixture was stirred at 35 °C for 30 min. Then TBAB (161.2 mg, 0.5 mmol, 1.0 equiv), LiOH (29.9 mg, 1.25 mmol, 2.5 equiv), D-labeled alkyl tosylate (153.1 mg, 0.5 mmol, 1 equiv) and aryl boric acid (151.5 mg, 0.75 mmol, 1.5 equiv) were added to the resulting mixture in this order. The resulting mixture was stirred at 35 ℃ and monitored by GC. After the reaction was complete, the mixture was quenched by saturated brine and extracted with ethyl acetate (40 mL × 3). The combined organic layers were dried over Na_2SO_4 and concentrated under reduced pressure. The resulting crude product was separated on a silica gel column affording the cross-coupling product 91.6 mg as a colorless oil, 75% yield, *rr* > 50:1. ^1H NMR (400 MHz, Chloroform-*d*) δ 7.29 - 7.24 (m, 2 H), 7.22 - 7.14 (m, 3 H), 7.08 - 7.03 (m, 1 H), 6.91 - 6.86 (m, 2 H), 3.85 (t, J = 7.8 Hz, 1 H), 2.20 (d, J = 1.9 Hz, 3 H), 2.00 - 1.95 (m, 2 H), 1.28 - 1.22 (m, 2 H), 0.92 - 0.85 (m, 1 H). ^{13}C NMR (151 MHz, Chloroform-d) δ 161.4 (d, J = 244.3 Hz), 145.3 (d, J = 7.0 Hz), 145.1, 131.3 (d, J = 5.4 Hz), 128.6, 127.9, 126.3, 123.4 (d, J = 2.9 Hz), 122.3 (d, J = 17.3 Hz), 114.4 (d, J = 22.2 Hz), 50.6, 37.9, 21.0, 14.3 (d, J = 3.6 Hz), 13.6 (qui, J = 19.1 Hz). ^{19}F NMR (376 MHz, Chloroform-*d*) δ -117.64. HRMS (ESI) Calculated for $C_{17}H_{17}D_2Na$ ($[M+Na]^+$): 267.1494, measured: 267.1487.

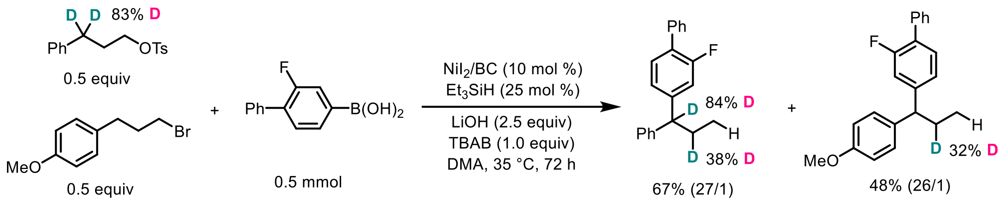

Supplementary Figure 26. D-labelled Experiment

Procedure: Under N_2 atmosphere, into an oven-dried 20 mL reaction tube equipped with a magnetic stir bar and sealed with a rubber stopper sequentially added NiI_2 (15.6 mg, 0.05 mmol, 10 mol %), BC (18.0 mg, 0.05 mmol, 10 mol %), and Et_3SiH (20 μL, 0.13 mmol, 25 mol %), anhydrous DMA (4 mL). The mixture was stirred at 35 °C for 30 min. Then TBAB (161.2 mg, 0.5 mmol, 1.0 equiv), LiOH (29.9 mg, 1.25 mmol, 2.5 equiv), D-labeled alkyl tosylate (73.0 mg, 0.25 mmol, 0.5 equiv), alkyl bromide (42 μL, 0.25 mmol, 0.5 equiv) and aryl boric acid (108.0 mg, 0.5 mmol, 1.0 equiv) were added to the resulting mixture in this order. The resulting mixture was stirred at 35 ℃ and monitored by GC. After the reaction was complete, the mixture was quenched by saturated brine and extracted with ethyl acetate (40 mL × 3). The combined organic layers were dried over Na_2SO_4 and concentrated under reduced pressure. The resulting crude product was separated on a silica gel column affording the cross-coupling product.

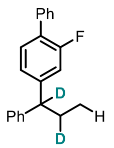

2-Fluoro-4-(1-phenylpropyl-1,2-d$_2$)-1,1'-biphenyl (3bj-D$_2$): ^{1}H NMR (400 MHz, Chloroform-*d*) δ 7.52 - 7.49 (m, 2 H), 7.42 – 7.38 (m, 2 H), 7.34 – 7.24 (m, 6 H), 7.21 – 7.17 (m, 1 H), 7.09 – 7.01 (m, 2 H), 2.10 – 2.04 (m, 1 H), 0.92 (t, J = 7.3 Hz, 3 H). ^{19}F NMR (376 MHz, Chloroform-*d*) δ -118.06. ^{13}C NMR (151 MHz, Chloroform-*d*) δ 159.9 (d, J = 247.7 Hz), 147.0 (d, J = 7.1 Hz), 144.5, 135.9, 130.6 (d, J = 4.1 Hz), 129.1 (d, J = 2.9 Hz), 128.7, 128.5, 128.01, 127.98, 127.6, 126.7 (d, J = 13.5 Hz), 126.5, 124.0 (d, J = 3.3 Hz), 115.5 (d, J = 22.9 Hz), 52.9 – 52.0 (m), 28.6 – 28.0 (m), 12.9 – 12.0 (m). ^{19}F NMR (376 MHz, Chloroform-*d*) δ -118.06. HRMS (ESI) Calculated for $C_{21}H_{17}D_2FNa$ ($[M+Na]^+$): 315.1494, measured: 315.1490.

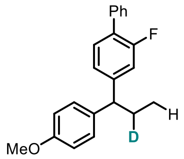

2-Fluoro-4-(1-(4-methoxyphenyl)propyl-2-d)-1,1'-biphenyl (3bk-D$_1$'): ^{1}H NMR (400 MHz, Chloroform-*d*) δ 7.52 - 7.49 (m, 2 H), 7.43 - 7.38 (m, 2 H), 7.34 - 7.30 (m, 2 H), 7.18 - 7.15 (m, 2 H), 7.07 - 6.99 (m, 2 H), 6.86 - 6.83 (m, 2 H), 3.78 (q, J = 7.3 Hz, 1 H), 3.77 (s, 3 H), 2.11 - 1.99 (m, 1 H), 0.92 (t, J = 7.2 Hz, 3 H). ^{13}C NMR (151 MHz, Chloroform-*d*) δ 159.8 (d, J = 247.6 Hz), 158.2, 147.5 (d, J = 7.0 Hz), 136.6, 135.9, 130.6 (d, J = 4.1 Hz), 129.1 (d, J = 2.8 Hz), 128.9, 128.5, 127.5, 126.6 (d, J = 13.6 Hz), 123.9 (d, J = 3.2 Hz), 115.4 (d, J = 22.9 Hz), 114.0, 55.3, 52.0, 28.7, 12.9. ^{19}F NMR (376 MHz, Chloroform-*d*) δ -118.12. HRMS (ESI) Calculated for $C_{22}H_{20}DFNa$ ($[M+Na]^+$): 344.1537, measured: 344.1535.

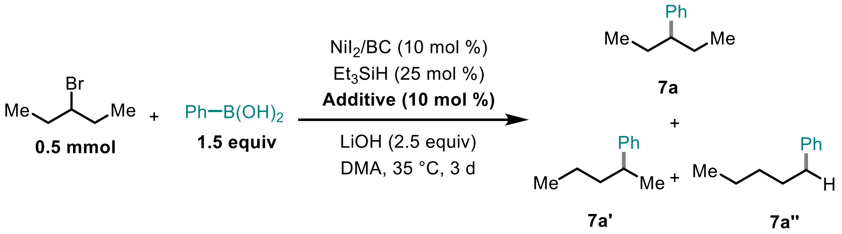

L5:

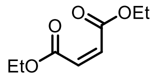

Additive	Yield (7a/7a'/7a'')
no	< 5%
**L5** (10 mol %)	30% (83:52:1)

Supplementary Figure 27. Investigation the Role of Aryl Group

In glovebox, into an oven-dried 10 mL reaction tube equipped with a magnetic stir bar and sealed with a rubber stopper sequentially added NiI_2 (15.6 mg, 0.05 mmol, 10 mol %), BC (18.0 mg, 0.05 mmol, 10 mol %), anhydrous DMA (4 mL) and Et_3SiH (20 μL, 0.13 mmol, 25 mol %). The mixture was stirred at 35 °C for 30 min. Then LiOH (29.9 mg, 1.25 mmol, 2.5 equiv), phenylboronic acid (91.5 mg, 0.5 mmol, 1.5 equiv), 3-bromopentane (62 μL, 0.5 mmol, 1 equiv) and L5 (8 μL, 0.05 mmol, 10 mol %) were added to the resulting mixture in this order. The tube was sealed with a rubber stopper, stirred at 35 °C and monitored by GC. After the reaction was complete, the mixture was quenched by saturated brine and extracted with ethyl acetate (20 mL × 3). The combined organic layers were dried over Na_2SO_4 and concentrated under reduced pressure. The resulting crude product was separated on a silica gel column affording the cross-coupling product.

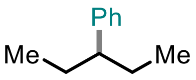

Pentan-3-ylbenzene (7a): [5] ^{1}H NMR (400 MHz, Chloroform-*d*) δ = 7.30 - 7.26 (m, 2 H), 7.19 - 7.12 (m, 3 H), 2.4 - 2.27 (m, 1 H)), 1.59 - 1.51 (m, 4 H), 0.77 (t, *J* = 7.4 Hz, 6 H). ^{13}C NMR (100 MHz, Chloroform-*d*) δ = 146.0, 128.3, 128.0, 125.9, 49.8, 29.4, 12.3.

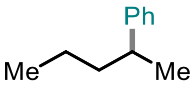

Pentan-2-ylbenzene (7a'): [6] ^{1}H NMR (400 MHz, Chloroform-*d*) δ = 7.30 - 7.26 (m, 2 H), 7.19 - 7.12 (m, 3 H), 2.72 - 2.66 (m, 1 H)), 1.74 - 1.63 (m, 3 H), 1.28 - 1.21 (m, 5 H), 0.86 (t, J = 7.0 Hz, 3 H). ^{13}C NMR (100 MHz, Chloroform-*d*) δ = 148.1, 128.4, 127.2, 125.9, 40.8, 39.8, 22.4, 21.0, 14.3.

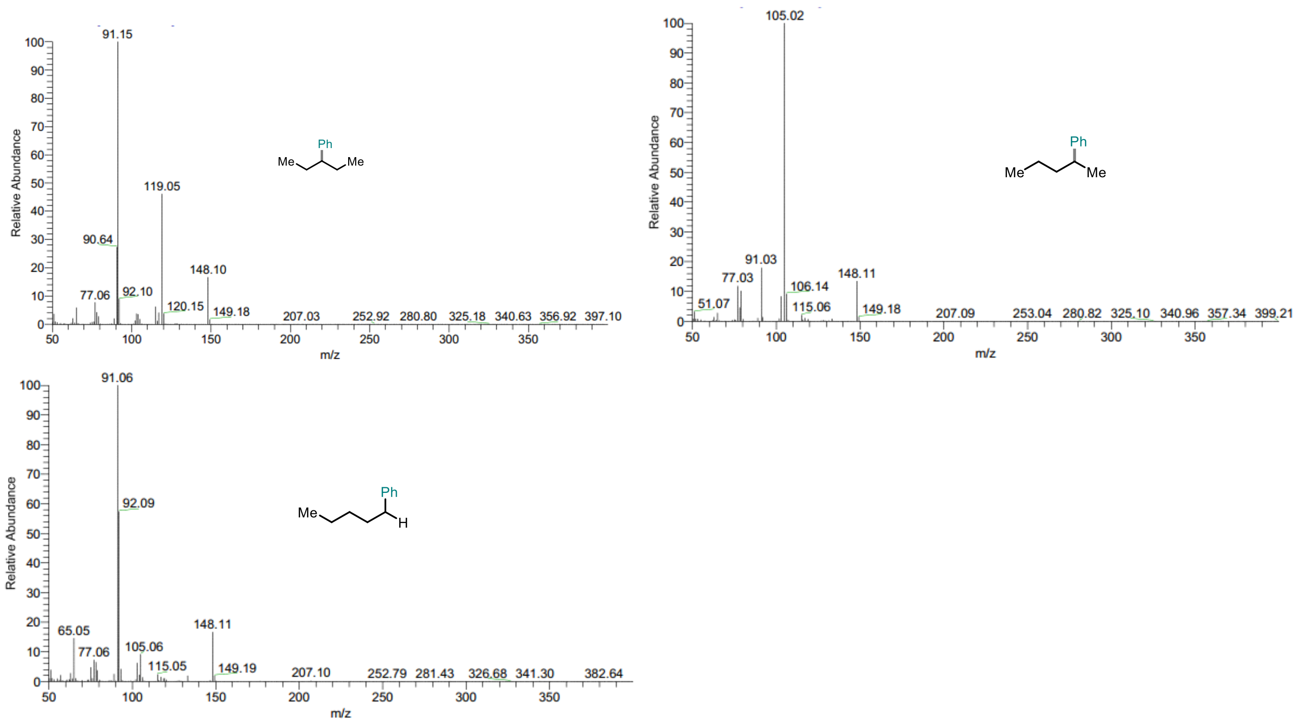

Supplementary Figure 28. Mass spectra for 7a, 7a' and 7a''

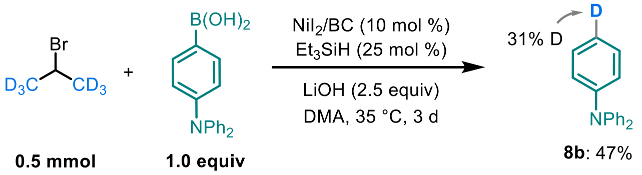

Supplementary Figure 29. D-labelled experiment

In glovebox, into an oven-dried 10 mL reaction tube equipped with a magnetic stir bar and sealed with a rubber stopper sequentially added NiI_2 (15.6 mg, 0.05 mmol, 10 mol %), BC (18.0 mg, 0.05 mmol, 10 mol %), anhydrous DMA (4 mL) and Et_3SiH (20 µL, 0.13 mmol, 25 mol %). The mixture was stirred at 35 °C for 30 min. Then LiOH (29.9 mg, 1.25 mmol, 2.5 equiv), (4-(diphenylamino)phenyl)boronic acid (216.8 mg, 0.5 mmol, 1.5 equiv) and 2-bromopropane-1,1,1,3,3,3-D6 (47 µL, 0.5 mmol, 1 equiv) were added to the resulting mixture in this order. The

tube was sealed with a rubber stopper, stirred at 35 °C and monitored by GC. After the reaction was complete, the mixture was quenched by saturated brine and extracted with ethyl acetate (20 mL × 3). The combined organic layers were dried over Na_2SO_4 and concentrated under reduced pressure. The resulting crude product was separated on a silica gel column affording the cross-coupling product.

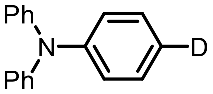

N,N-diphenylaniline-4-D: [7] ^{1}H NMR (400 MHz, Chloroform-*d*) δ 7.27 - 7.22 (m, 6 H), 7.11 - 7.07 (m, 6 H), 7.03 - 6.99 (m, 2.69 H). ^{13}C NMR (101 MHz, Chloroform-*d*) δ 148.0, 129.3, 124.3, 122.8.

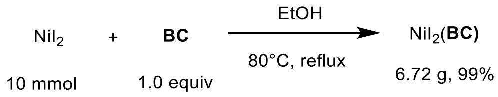

Preparation of NiI_2(BC): To a round bottom flask was added NiI_2 (10 mmol, 1.0 equiv, 3.12 g) and BC (10 mmol, 1.0 equiv, 3.60 g). Absolute EtOH and a magnetic stir bar were added to the flask and a reflux condenser was attached. The mixture was heated to 80°C for the length of time indicated, after which the flask was cooled to ambient temperature, the magnetic stir bar was removed, and the solvent was evaporated to provide NiI_2(**BC**) (6.72 g, 99% yield) by rotary evaporation.

Preventative Time Course of the Reactions: In glovebox, into an oven-dried 10 mL reaction tube equipped with a magnetic stir bar and sealed with a rubber stopper sequentially added NiI_2(**BC**) (33.6 mg, 0.05 mmol, 10 mol %), LiOH (29.9 mg, 1.25 mmol, 2.5 equiv), (3-bromopropyl)benzene (76 μL, 0.5 smmol, 1 equiv), (4-cyanophenyl)boronic acid (110.2 mg, 0.75 mmol, 1.5 equiv), trimethoxybenzene (42.0 mg, 0.25 mmol, 0.5 equiv) and DMA (4 mL) were added to the resulting mixture in this order. The resulting mixture was stirred at 35 °C and monitored by GC until the alkyl bromoride disappeared. The reaction solution (10 μL) was taken out by syringe at certain time and immediately quenched by water. Each data point represents the result of the sample which was analyzed by gas chromatography. The representative time course reveals a monotonic decrease in [(3-bromopropyl)benzene] and increase in [**3aj**] (Supplementary Figure 30).

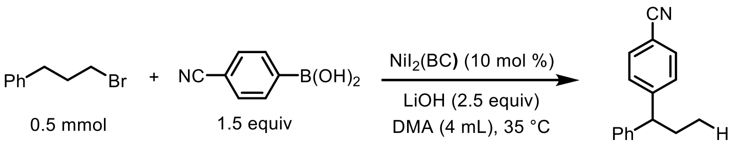

3aj

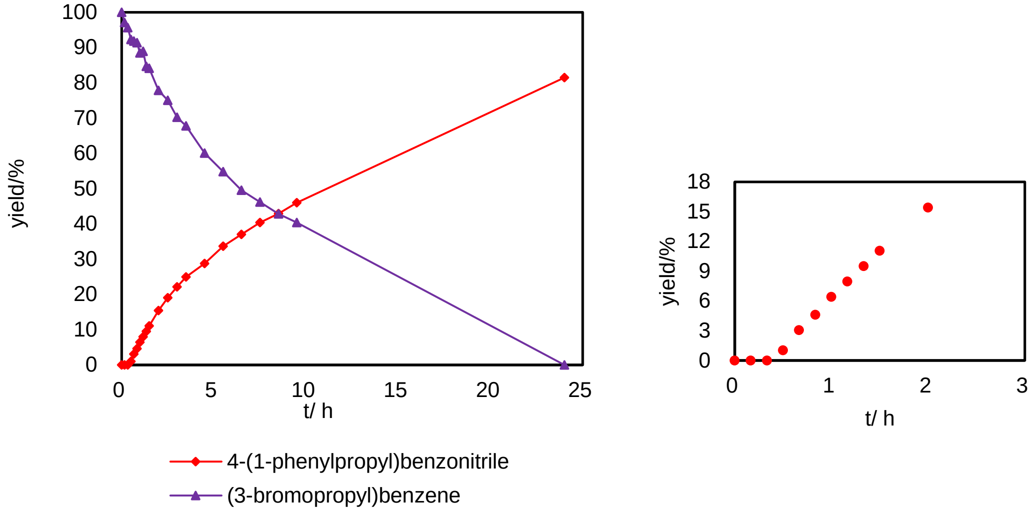

Supplementary Figure 30. Representive time course of the reaction. Reaction condition: [NiI_2(**BC**)] = 12.5 mM, [(3-bromopropyl)benzene] = 0.125 M, [(4-cyanophenyl)boronic acid] = 0.188 M, [LiOH] = 0.312 M, [Trimethoxybenzene] = 0.0625 M at 35 °C in DMA.

Kinetic Studies: With the optimized reaction condition, the kinetics of the reaction was monitored by gas chromatography with 1,3,5-trimethoxybenzene as internal standard. Each reaction was monitored to 0-20% conversion, and rate constants were calculated for each reaction using the initial rates method. Error analysis was conducted using standard equations and calculations.

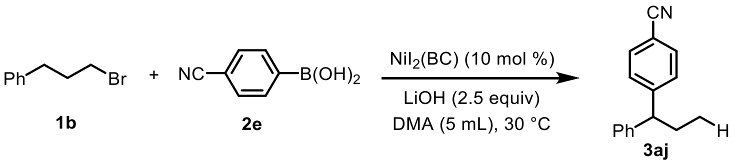

General procedure to determine the initial rate on the concentration of NiI_2(BC): In glovebox, into an oven-dried 10 mL reaction tube equipped with a magnetic stir bar and sealed with a rubber stopper sequentially added NiI_2(BC) (2 mol %, 3 mol %, 4 mol %, 5 mol %, 7.5 mol %, 10 mol %, 12.5 mol %, 15 mol %, respectively), LiOH (29.9 mg, 1.25 mmol, 2.5 equiv), **1b** (76 μL, 0.5 mmol, 1 equiv), **2e** (73.5 mg, 0.50 mmol, 1.0 equiv), trimethoxybenzene (42.0 mg, 0.25 mmol, 0.5 equiv) and DMA (5 mL) were added to the resulting mixture in this order. The resulting mixture was stirred at 30 °C. The reaction solution (20 μL) was taken out by syringe at every 0.5 h and immediately quenched by water. Each data point represents the result of the sample which was analyzed by gas chromatography.

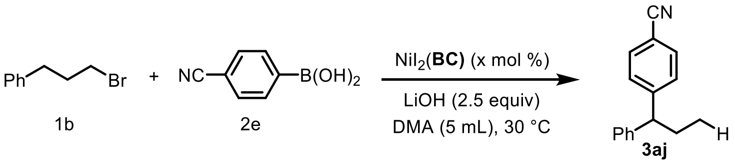

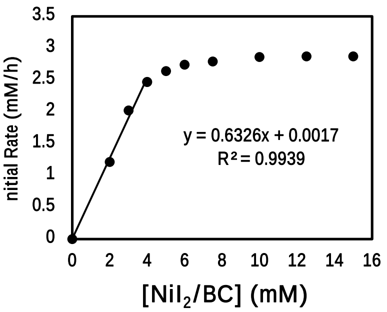

Supplementary Figure 31. Initial rate on the concentration of NiI_2(**BC**) from the reaction of **1b** (0.1 M), LiOH (0.25 M), **2e** (0.10 M) with 2 mM, 3 mM, 4 mM, 5 mM, 7.5 mM, 10 mM, 12.5 mM, 15 mM) of NiI_2(**BC**).

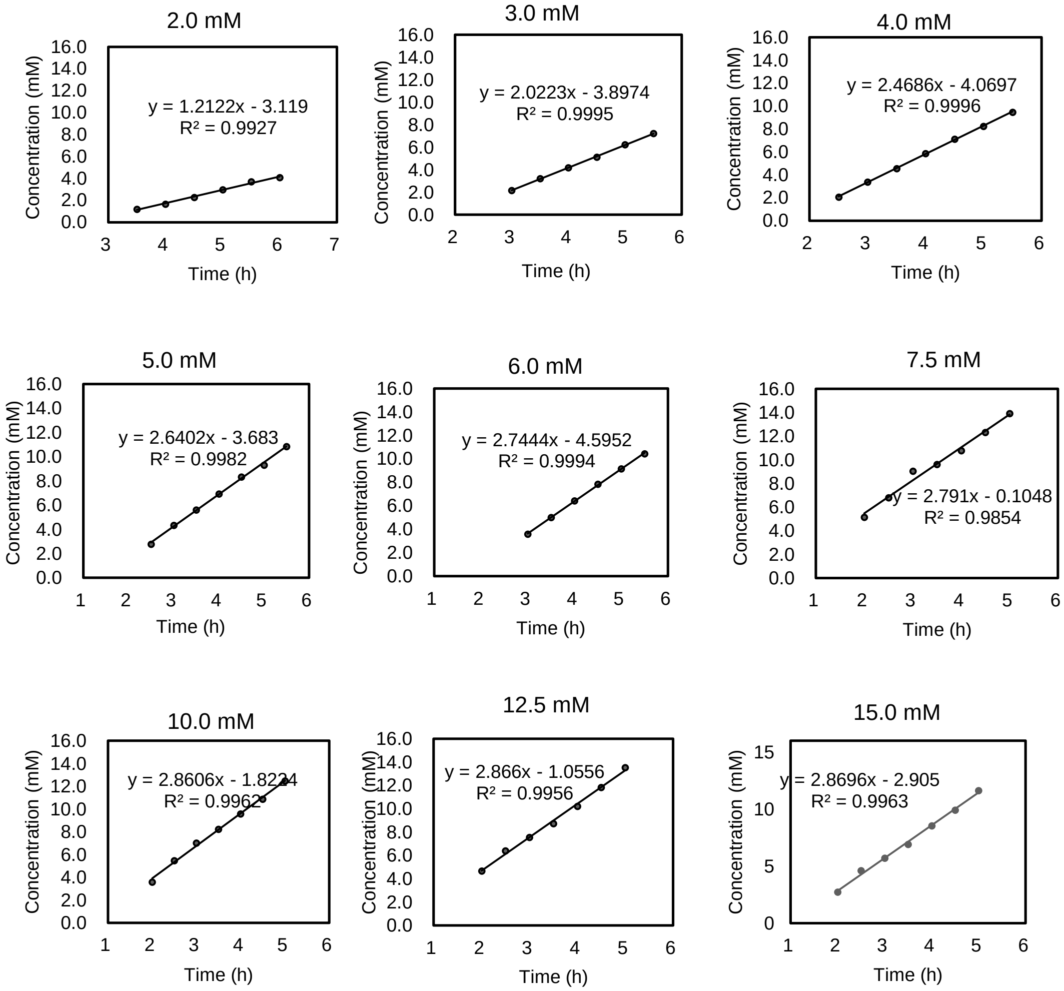

Supplementary Figure 32. Plot of the rate of product from the reaction of **1b** (0.10 M), LiOH (0.25 M), **2e** (0.10 M) with 2 mM, 3 mM, 4 mM, 5 mM, 7.5 mM, 10 mM, 12.5 mM, 15 mM) of NiI_2(BC).

General procedure to determine the initial rate on the concentration of 1-Bromo-3-phenylpropane (1b): In glovebox, into an oven-dried 10 mL reaction tube equipped with a magnetic stir bar and sealed with a rubber stopper sequentially added NiI_2(**BC**) (33.6 mg, 0.05 mmol, 10 mol %), LiOH (29.9 mg, 1.25 mmol, 2.5 equiv), **1b** (0.5 equiv, 1.0 equiv, 1.5 equiv, 2.0 equiv, 2.5 equiv, respectively), **2e** (73.5 mg, 0.50 mmol, 1.0 equiv),

trimethoxybenzene (42.0 mg, 0.25 mmol, 0.5 equiv) and DMA (5 mL) were added to the resulting mixture in this order. The resulting mixture was stirred at 30 °C. The reaction solution (20 μL) was taken out by syringe at every 0.5 h and immediately quenched by water. Each data point represents the result of the sample which was analyzed by gas chromatography.

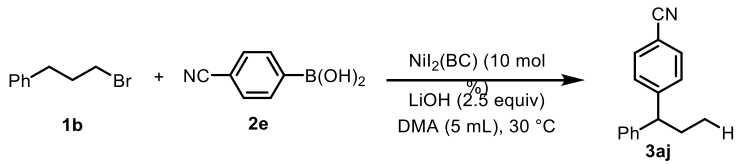

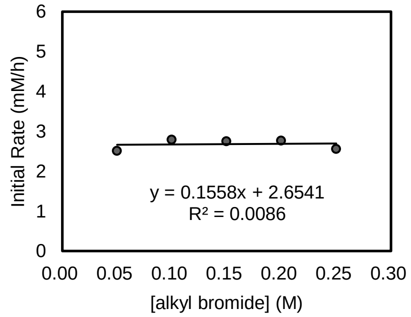

Supplementary Figure 33. Initial rate on the concentration of **1b** from the reaction of NiI_2(BC) (0.01 M), LiOH (0.25 M), **2e** (0.10 M) with 0.05 M, 0.10 M, 0.15 M, 0.20, 0.25 M of **1b**.

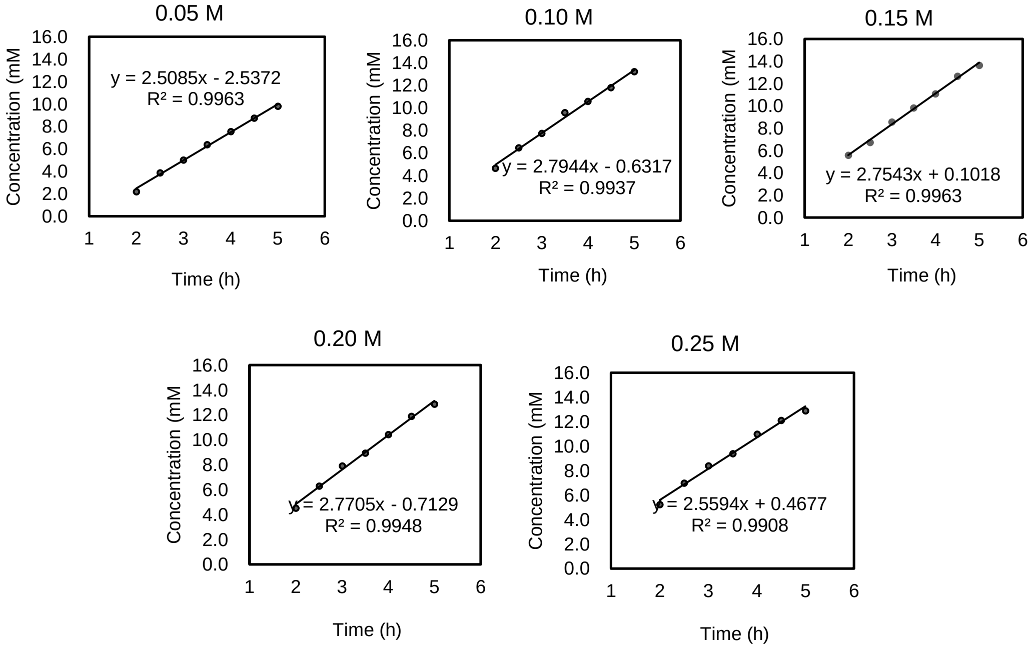

Supplementary Figure 34. Plot of the rate of product from the reaction of NiI_2(BC) (0.01 M), LiOH (0.25 M), **2e** (0.10 M) with 0.05 M, 0.10 M, 0.15 M, 0.20, 0.25 M of **1b**.

General procedure to determine the initial rate on the concentration of LiOH: In glovebox, into an oven-dried 10 mL reaction tube equipped with a magnetic stir bar and sealed with a rubber stopper sequentially added

NiI_2(**BC**) (33.6 mg, 0.05 mmol, 10 mol %), LiOH (2.0 equiv, 2.5 equiv, 3.0 equiv, 3.5 equiv, respectively), (3-bromopropyl)benzene (76 μL, 0.5 mmol, 1 equiv), **2e** (73.5 mg, 0.50 mmol, 1.0 equiv), trimethoxybenzene (42.0 mg, 0.25 mmol, 0.5 equiv) and DMA (5 mL) were added to the resulting mixture in this order. The resulting mixture was stirred at 30 °C. The reaction solution (20 μL) was taken out by syringe at every 0.5 h and immediately quenched by water. Each data point represents the result of the sample which was analyzed by gas chromatography.

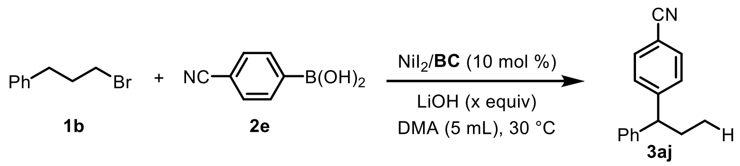

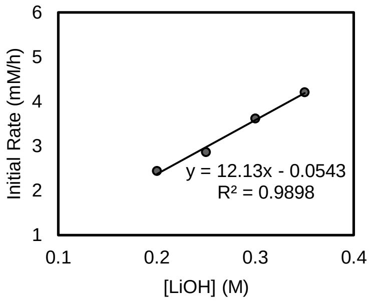

Supplementary Figure 35. Initial rate on the concentration of LiOH from the reaction of NiI_2(BC) (0.01 M), **1b** (0.1 M), **2e** (0.10 M) with 0.20 M, 0.25 M, 0.30 M, 0.35 M of LiOH.

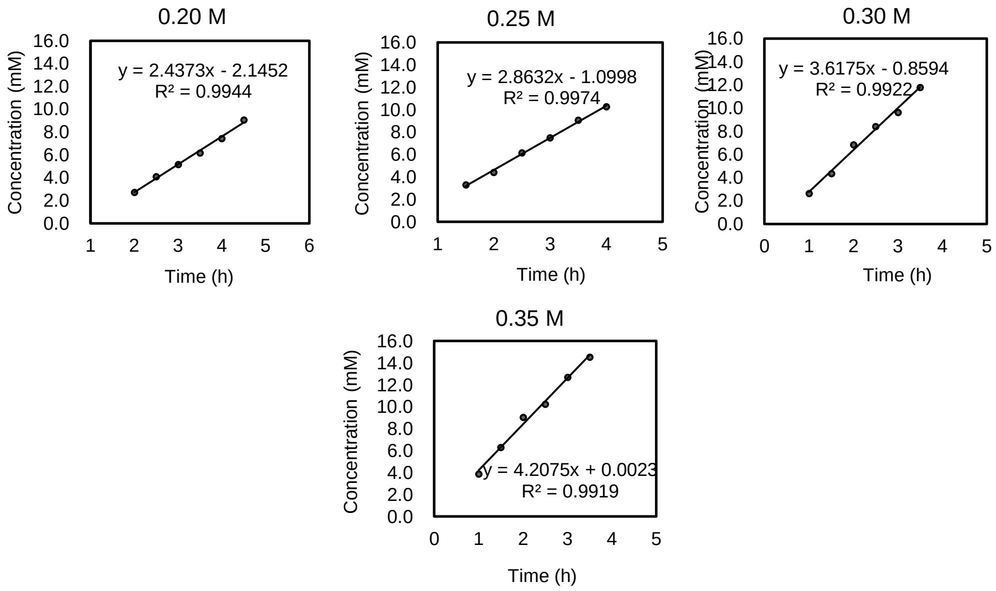

Supplementary Figure 36. Plot of the rise of product from the reaction of NiI_2(BC) (0.01 M), **1b** (0.1 M), **2e** (0.10 M) with 0.20 M, 0.25 M, 0.30 M, 0.35 M of LiOH.

General procedure to determine the initial rate on the concentration of (4-cyanophenyl)boronic acid (2e): In glovebox, into an oven-dried 10 mL reaction tube equipped with a magnetic stir bar and sealed with a rubber stopper sequentially added NiI_2(**BC**) (33.6 mg, 0.05 mmol, 10 mol %), LiOH (29.9 mg, 1.25 mmol, 2.5 equiv), (3-

bromopropyl)benzene (76 μL, 0.5 mmol, 1 equiv), **2e** (1.0 equiv, 1.5 equiv, 2.0 equiv, 2.5 equiv, 3.0 equiv, respectively), trimethoxybenzene (42.0 mg, 0.25 mmol, 0.5 equiv) and DMA (5 mL) were added to the resulting mixture in this order. The resulting mixture was stirred at 30 °C. The reaction solution (20 μL) was taken out by syringe at every 0.5 h and immediately quenched by water. Each data point represents the result of the sample which was analyzed by gas chromatography.

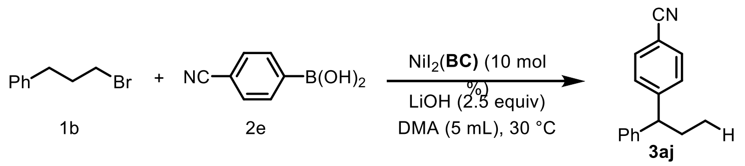

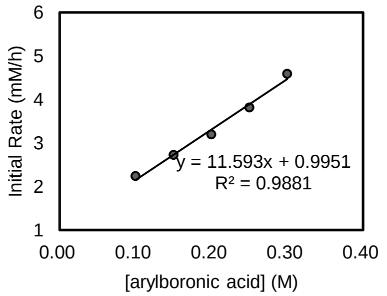

Supplementary Figure 37. Initial rate on the concentration of **2e** from the reaction of NiI_2(BC) (0.01 M), **1b** (0.10 M), LiOH (0.25 M) with 0.10 M, 0.15 M, 0.20 M, 0.25 M, 0.30 M of **2e**.

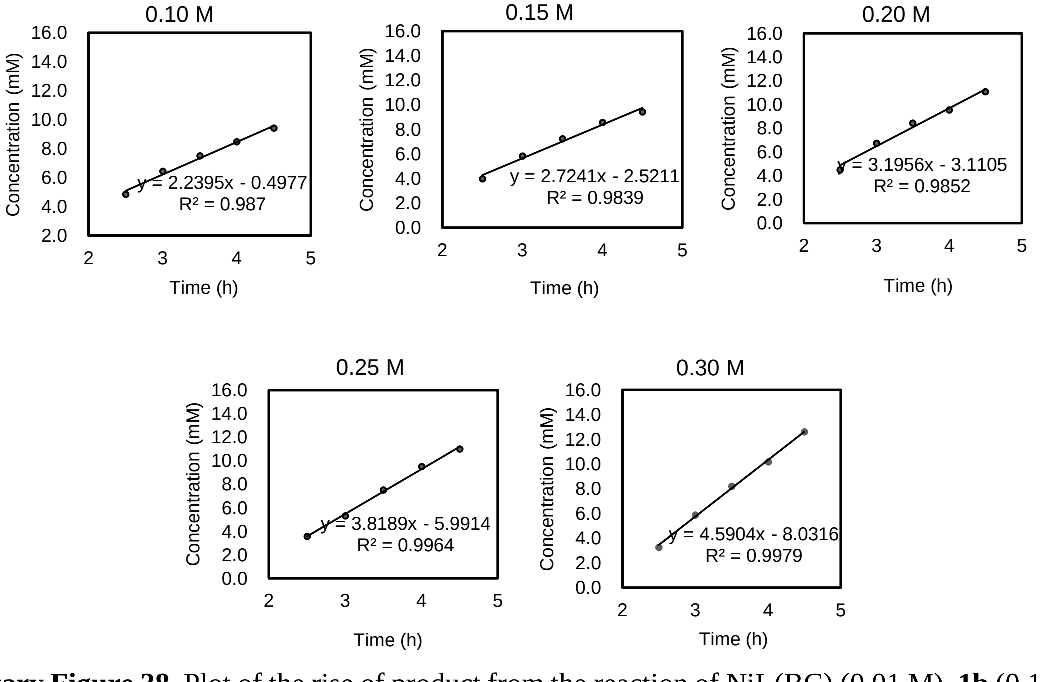

Supplementary Figure 38. Plot of the rise of product from the reaction of NiI_2(BC) (0.01 M), **1b** (0.10 M), LiOH (0.25 M) with 0.10 M, 0.15 M, 0.20 M, 0.25 M, 0.30 M of **2e**.

Hammett Plot. Relative rate constants were determined for the reaction of **2** with different (p-RC_6H_4)$(CH_2)_3$Br

(R = MeO, H, Cl, and CF_3).

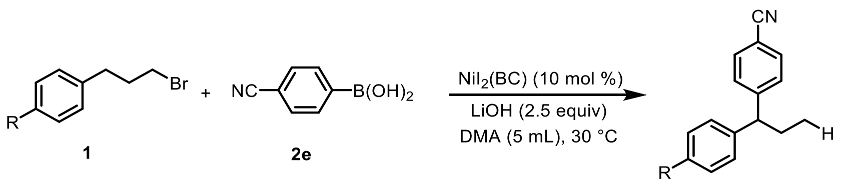

General procedure to determine the initiate rates of different (p-RC_6H_4)(CH_2)$_3$Br: In glovebox, into an oven-dried 10 mL reaction tube equipped with a magnetic stir bar and sealed with a rubber stopper sequentially added NiI_2(**BC**) (33.6 mg, 0.05 mmol, 10 mol %), LiOH (29.9 mg, 1.25 mmol, 2.5 equiv), alkyl bromide (0.5 mmol, 1 equiv), **2e** (73.5 mg,0.5 mmol, 1.0 equiv), trimethoxybenzene (42.0 mg, 0.25 mmol, 0.5 equiv) and DMA (5 mL) were added to the resulting mixture in this order. The resulting mixture was stirred at 30 °C. The reaction solution (20 μL) was taken out by syringe at every 20 min and immediately quenched by water. Each data point represents the result of the sample which was analyzed by gas chromatography.

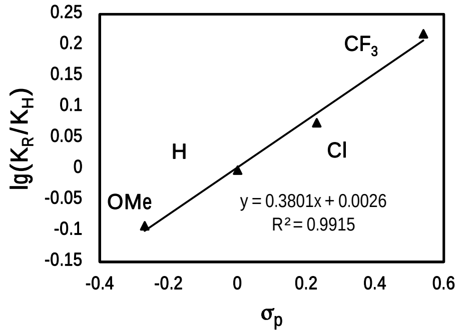

Supplementary Figure 39. Hammett plots of alkyl bromides from the reaction of NiI_2(BC) (0.01 M), alkyl bromides (0.10 M), LiOH (0.25 M) with **2e** (0.1M).

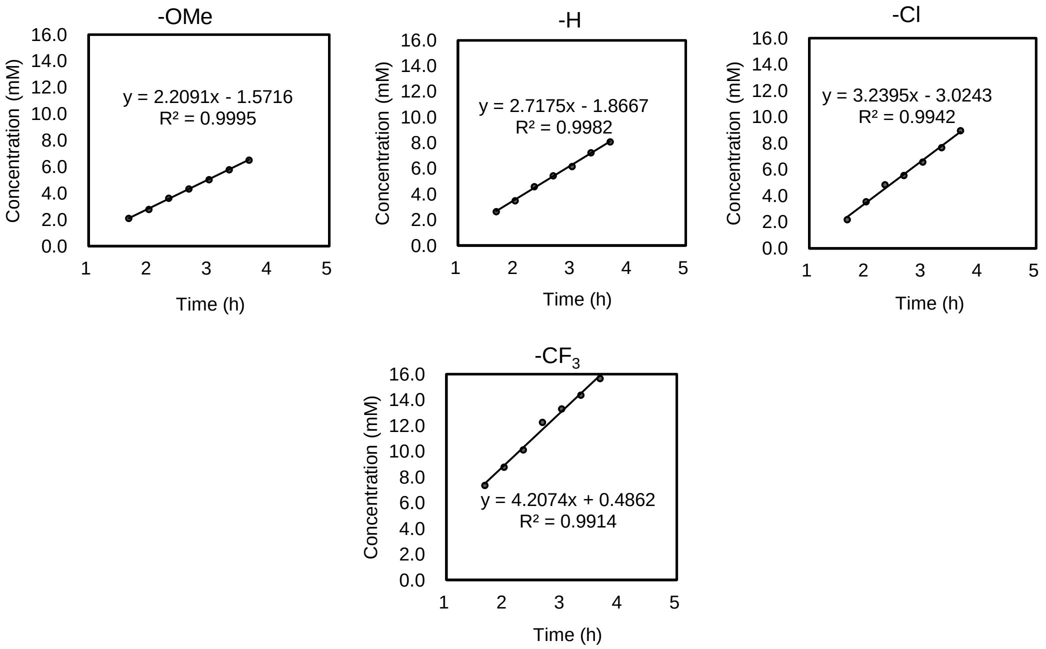

Supplementary Figure 40. Relative rate constants were determined for the reaction of **2e** with different alkyl bromides (R = MeO, H, Cl, CF_3).

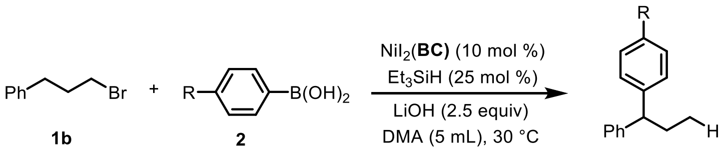

General procedure to determine the initial rate on different p-$RC_6H_4B(OH)_2$ (R = MeO, H, F, CF_3, CN): In glovebox, into an oven-dried 10 mL reaction tube equipped with a magnetic stir bar and sealed with a rubber stopper sequentially added NiI_2(**BC**) (33.6 mg, 0.05 mmol, 10 mol %), Et_3SiH (20 μL, 0.125 mmol, 25 mol %), LiOH (29.9 mg, 1.25 mmol, 2.5 equiv), **1b** (76 μL, 0.5 mmol, 1 equiv), different p-$RC_6H_4B(OH)_2$ (xx mg, 0.5 mmol, 1.0 equiv) Trimethoxybenzene (42.0 mg, 0.25 mmol, 0.5 equiv) and DMA (5 mL) were added to the resulting mixture in this order. The resulting mixture was stirred at 30 °C. The reaction solution (20 μL) was taken out by syringe at every 0.5 h and immediately quenched by water. Each data point represents the result of the sample which was analyzed by gas chromatography.

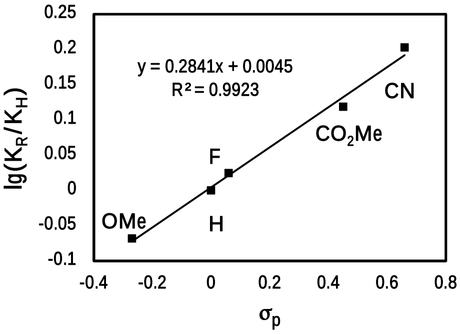

Supplementary Figure 41. Hammett plots of p-$RC_6H_4B(OH)_2$ from the reaction of NiI_2(BC) (0.01 M), Et_3SiH (0.025 M), **1b** (0.10 M), LiOH (0.25 M) with p-$RC_6H_4B(OH)_2$ (0.10 M).

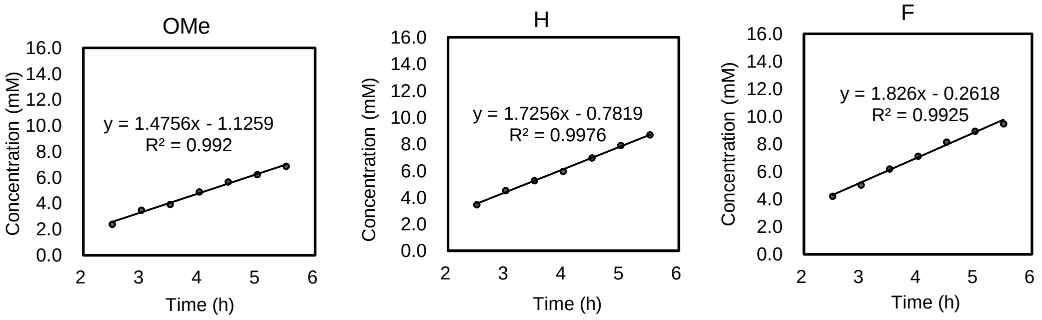

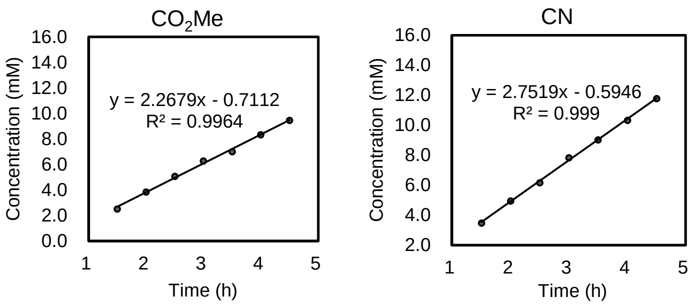

Supplementary Figure 42. Relative rate constants were determined for the reaction of **1b** with different p-$RC_6H_4B(OH)_2$ (R = MeO, H, F, CO_2Me, CN).

General procedure to determine the initiate rates of different alkyl bromides: In glovebox, into an oven-dried 10 mL reaction tube equipped with a magnetic stir bar and sealed with a rubber stopper sequentially added NiI_2(**BC**) (33.6 mg, 0.05 mmol, 10 mol %), LiOH (29.9 mg, 1.25 mmol, 2.5 equiv), alkyl bromide (0.5 mmol, 1 equiv), **2e** (73.5 mg,0.5 mmol, 1.0 equiv), trimethoxybenzene (42.0 mg, 0.25 mmol, 0.5 equiv) and DMA (5 mL) were added to the resulting mixture in this order. The resulting mixture was stirred at 30 °C. The reaction solution (20 μL) was taken out by syringe at every 0.5 h and immediately quenched by water. Each data point represents the result of the sample which was analyzed by gas chromatography.

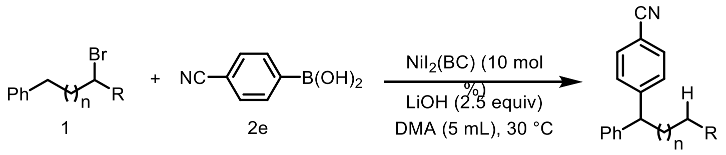

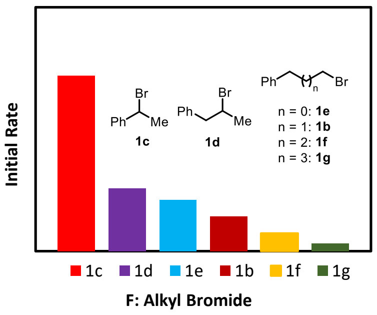

Supplementary Figure 43. Initial rate on the concentration of different alkyl bromides from the reaction of NiI_2(**BC**) (0.01 M), alkyl bromides (0.10 M), LiOH (0.25 M) with **2e** (0.1M).

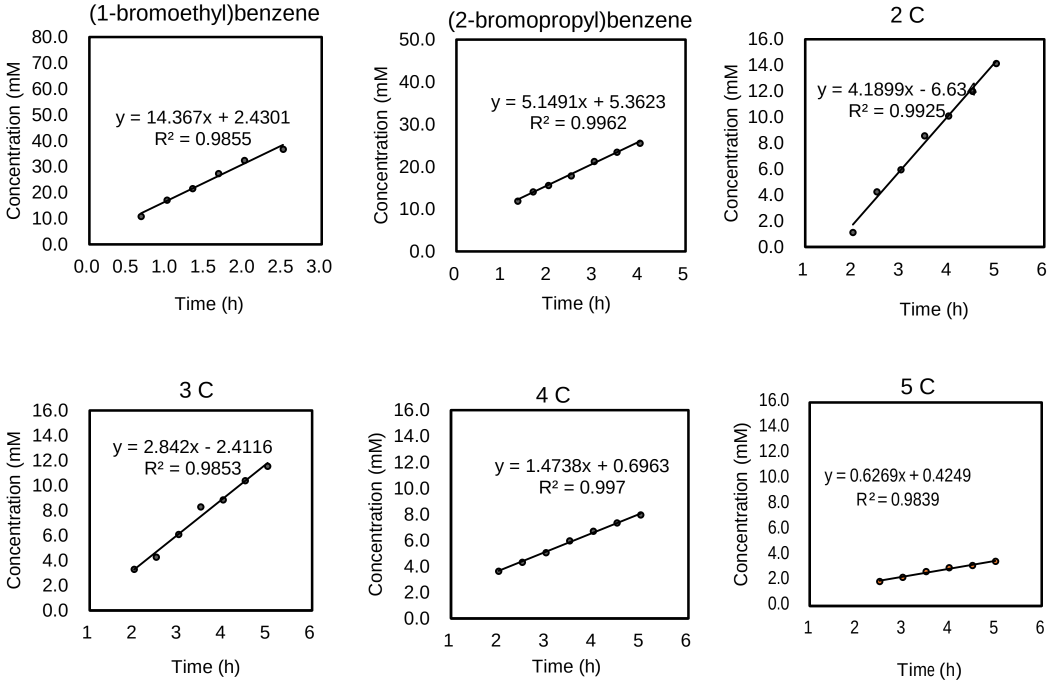

Supplementary Figure 44. Relative rate constants were determined for the reaction of different alkyl bromides with **2e**.

Radical Clock Experiment: In glovebox, into an oven-dried 10 mL reaction tube equipped with a magnetic stir bar and sealed with a rubber stopper sequentially added NiI_2/L4 (2 mol %, 4 mol %, 6 mol %, 8 mol %, 10 mol %, 15 mol %, 20 mol %, 25 mol %, respectively), LiOH (29.9 mg, 1.25 mmol, 2.5 equiv), 6-bromohex-1-ene (67 μL, 0.5 mmol, 1 equiv), (4-cyanophenyl)boronic acid (110.0 mg, 0.75 mmol, 1.5 equiv) and DMA (5 mL) were added to the resulting mixture in this order. The resulting mixture was stirred at 35 °C for 48 h. After the reaction was complete, the mixture was quenched by saturated brine and extracted with ethyl acetate (20 mL × 3). The combined organic layers were dried over Na_2SO_4 and concentrated under reduced pressure. The resulting crude product was separated on a silica gel column affording the cross-coupling product.

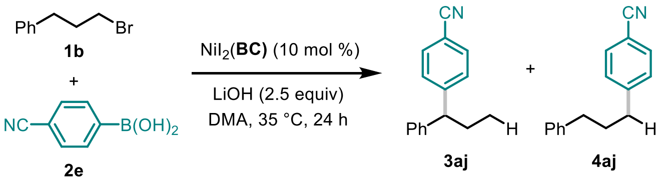

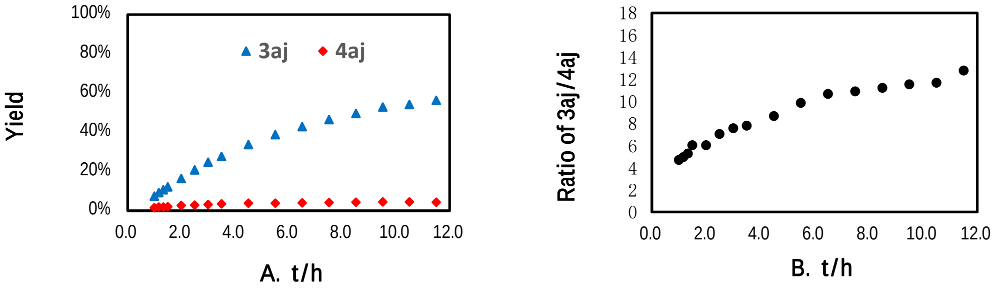

Supplementary Figure 45. Time Course of Product 3aj and 4aj

Radical Clock Experiment: In glovebox, into an oven-dried 10 mL reaction tube equipped with a magnetic stir bar and sealed with a rubber stopper sequentially added NiI_2/L4 (2 mol %, 4 mol %, 6 mol %, 8 mol %, 10 mol %, 15 mol %, 20 mol %, 25 mol %, respectively), LiOH (29.9 mg, 1.25 mmol, 2.5 equiv), 6-bromohex-1-ene (67 μL, 0.5 mmol, 1 equiv), (4-cyanophenyl)boronic acid (110.0 mg, 0.75 mmol, 1.5 equiv) and DMA (5 mL) were added to the resulting mixture in this order. The resulting mixture was stirred at 35 °C for 48 h. After the reaction was complete, the mixture was quenched by saturated brine and extracted with ethyl acetate (20 mL × 3). The combined organic layers were dried over Na_2SO_4 and concentrated under reduced pressure. The resulting crude product was separated on a silica gel column affording the cross-coupling product.

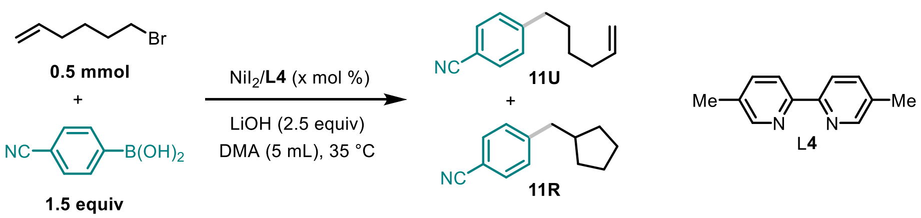

Supplementary Figure 46. Radical Clock Experiment

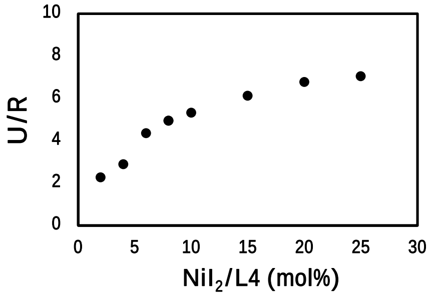

Supplementary Figure 47. Effect of Catalyst Loading on the Ratio of 11U/11R

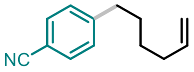

4-(hex-5-en-1-yl)benzonitrile: [8] ^{1}H NMR (400 MHz, Chloroform-*d*) δ 7.57 - 7.55 (m, 2 H), 7.28 - 7.26 (m, 2 H), 5.83 - 5.73 (m, 2 H), 5.18 - 4.81 (m, 1H), 2.69 (t, *J* = 8.0 Hz, 2 H), 2.11 - 2.05 (m, 2 H), 1.68 - 1.61 (m, 2 H), 1.47 - 1.39 (m, 2 H). ^{13}C NMR (101 MHz, Chloroform-*d*) δ 148.5, 138.6, 132.3, 129.4, 129.3, 119.3, 114.8, 109.7, 36.0, 33.6, 30.5, 28.5.

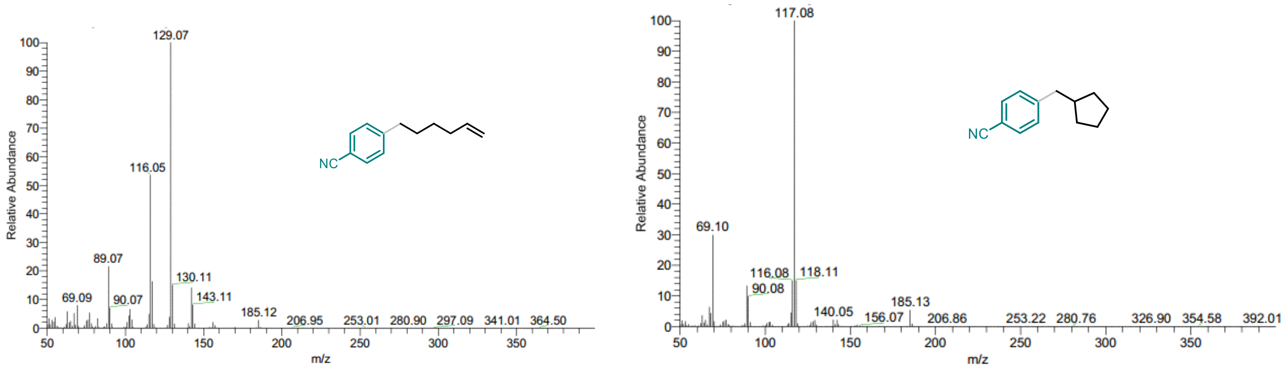

Supplementary Figure 48. Mass spectra for 11U and 11R

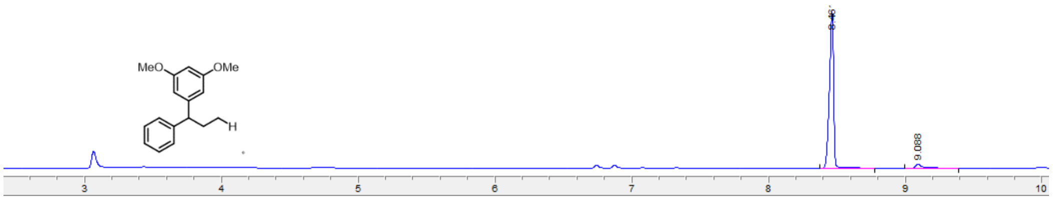

Supplementary Figure 49. The crude GC spectrum of **3d**

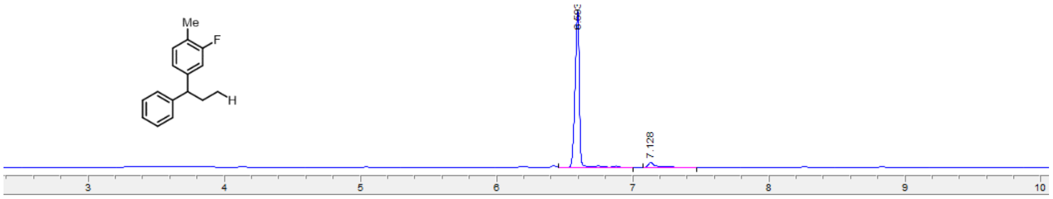

Supplementary Figure 50. The crude GC spectrum of **3g**

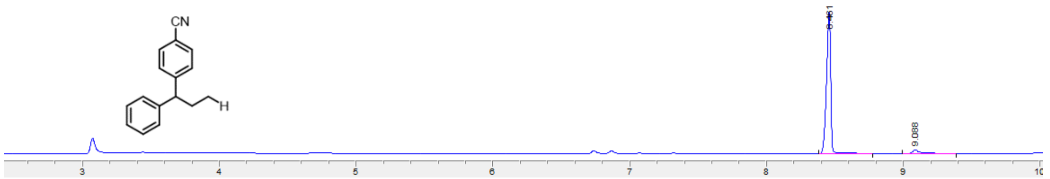

Supplementary Figure 51. The crude GC spectrum of **3aj**

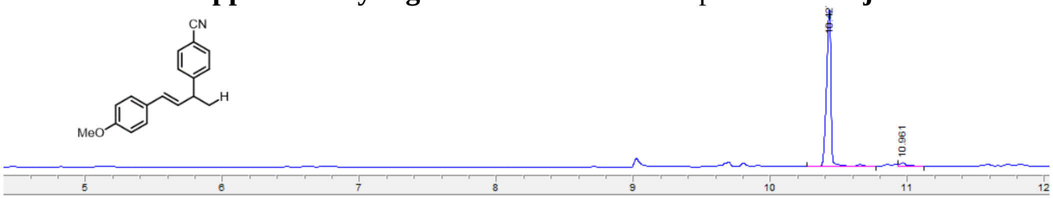

Supplementary Figure 52. The crude GC spectrum of **3ad**

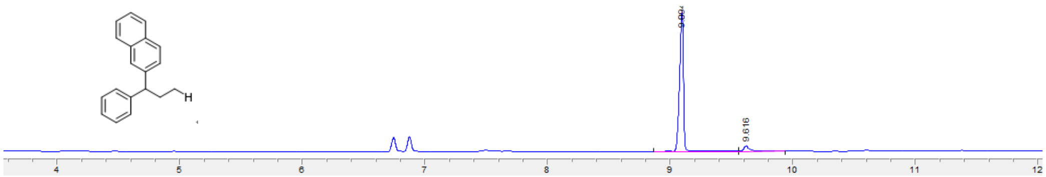

Supplementary Figure 53. The crude GC spectrum of **3au**

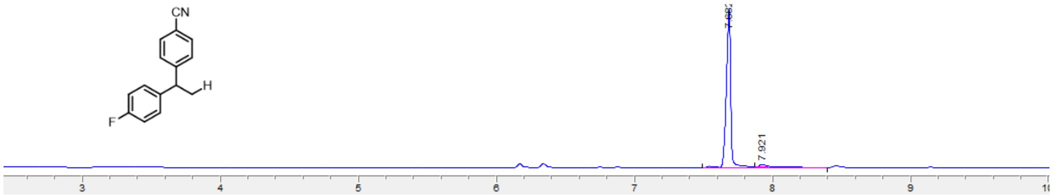

Supplementary Figure 54. The crude GC spectrum of **3aw**

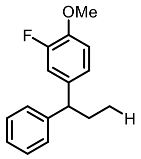

2-Fluoro-1-methoxy-4-(1-phenylpropyl)benzene (3a): The reaction was conducted following the general procedure **A** in a 0.5 mmol scale. The residue was purified by column chromatography on silica gel to afford the product **3a** (100.1 mg, 82 % yield, *rr* = 27:1) as a colorless oil. ^{1}H NMR (400 MHz, Chloroform-*d*) δ 7.30 - 7.25 (2 H), 7.21 - 7.15 (m, 3 H), 6.97 - 6.92 (m, 2 H), 6.88 - 6.83 (m, 1 H), 3.84 (s, 3 H), 3.71 (t, *J* = 7.8 Hz, 1 H), 2.02 (dq, *J* = 7.3, 7.3 Hz, 2 H), 0.88 (t, *J* = 7.3 Hz, 3 H). ^{13}C NMR (101 MHz, Chloroform-*d*) δ 152.4 (d, *J* = 245.1 Hz), 145.8 (d, *J* = 10.7 Hz), 145.0, 138.5 (d, *J* = 5.5 Hz), 128.6, 127.9, 126.3, 123.5 (d, *J* = 3.3 Hz), 115.6 (d, *J* = 18.1 Hz), 113.3 (d, *J* = 2.2 Hz), 56.4, 52.4 (d, *J* = 1.3 Hz), 28.7, 12.8. ^{19}F NMR (377 MHz, Chloroform-*d*) δ -135.40. HRMS (ESI) Calculated for $C_{16}H_{17}FNaO$ ([M+Na]$^+$): 267.1156, measured: 267.1155.

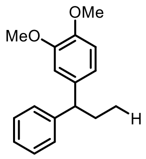

1, 2-Dimethoxy-4-(1-phenylpropyl)benzene (3b): The reaction was conducted following the general procedure **A** in a 0.5 mmol scale. The residue was purified by column chromatography on silica gel to afford the product **3b** (80.7 mg, 63 % yield, *rr* = 27:1) as a colorless oil. ^{1}H NMR (400 MHz, Chloroform-*d*) δ7.30 - 7.20 (m, 4 H), 7.19 - 7.14 (m, 1 H), 6.79 (s, 2 H), 6.73 (s, 1 H), 3.84 (s, 3 H), 3.83 (s, 3 H), 3.73 (t, *J* = 7.8 Hz, 1 H), 2.04 (dq, *J* = 7.4, 7.4 Hz, 2 H), 0.90 (t, *J* = 7.3 Hz, 3 H). ^{13}C NMR (101 MHz, Chloroform-*d*) δ 148.8, 147.3, 145.5, 137.9, 128.5, 127.9, 126.1, 119.7, 111.4, 111.1, 55.9, 55.9, 52.9, 28.9, 13.0. HRMS (ESI) Calculated for $C_{17}H_{20}NaO_2$ ([M+Na]$^+$): 279.1356, measured: 279.1358.

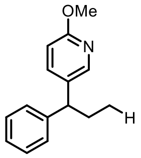

2-Methoxy-5-(1-phenylpropyl)pyridine (3c): The reaction was conducted following the general procedure **A** in a 0.5 mmol scale. The residue was purified by column chromatography on silica gel to afford the product **3c** (77.2 mg, 68% yield, *rr* = 46:1) as a colorless oil. ^{1}H NMR (400 MHz, Chloroform-*d*) δ 8.06 (d, *J* = 2.5 Hz, 1 H), 7.40 (dd, *J* = 8.6, 2.5 Hz, 1 H), 7.30 - 7.25 (m, 2H), 7.22 - 7.15 (m, 3 H), 6.66 (dd, *J* = 8.6, 0.7 Hz, 1 H), 3.90 (s, 3 H), 3.73 (t, *J* = 7.8 Hz, 1 H), 2.12 - 1.96 (m, 2 H), 0.90 (t, *J* = 7.3 Hz, 3 H). ^{13}C NMR (101 MHz, Chloroform-*d*) δ 162.8, 145.8, 144.6, 138.4, 133.3, 128.6, 127.9, 126.4, 110.8, 53.4, 50.0, 28.5, 12.8. HRMS (ESI) Calculated for $C_{15}H_{17}NNaO$ ([M+Na]$^+$): 250.1202, measured: 250.1205.

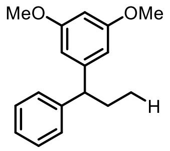

1, 3-Dimethoxy-5-(1-phenylpropyl)benzene (3d): The reaction was conducted following the general procedure **A** in a 0.5 mmol scale. The residue was purified by column chromatography on silica gel to afford the product **3d** (111.4 mg, 87 % yield, *rr* = 28:1) as a colorless oil. ^{1}H NMR (400 MHz, Chloroform-*d*) δ 7.30 - 7.22 (m, 4 H), 7.20 - 7.14 (m, 1 H), 6.41 (d, *J* = 2.2 Hz, 2 H), 6.28 (t, *J* = 2.3 Hz, 1 H), 3.75 (s, 6 H), 3.71 (t, *J* = 7.8 Hz, 1 H), 2.08 - 2.00 (m, 2 H), 0.90 (t, *J* = 7.3 Hz, 3 H). ^{13}C NMR (101 MHz, Chloroform-*d*) δ 160.8, 147.7, 145.0, 128.5, 127.9, 126.2, 106.4, 97.6, 55.3, 53.6, 28.6, 12.9. HRMS (ESI) Calculated for $C_{17}H_{20}NaO_2$ ([M+Na]$^+$): 279.1356, measured: 279.1358.

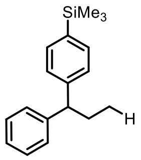

Trimethyl(4-(1-phenylpropyl)phenyl)silane (3e): The reaction was conducted following the general procedure **A** in a 0.5 mmol scale. The residue was purified by column chromatography on silica gel to afford the product **3e** (105.9 mg, 79% yield, *rr* = 12:1) as a colorless oil. ^{1}H NMR (400 MHz, Chloroform-*d*) δ 7.43 - 7.41 (m, 2 H), 7.29 - 7.21 (m, 6 H), 7.18 - 7.14 (m, 1 H), 3.77 (t, *J* = 7.8 Hz, 1 H), 2.07 (dq, *J* = 7.4 Hz, *J* = 7.4 Hz, 2 H), 0.89 (t, *J* = 7.3 Hz, 3 H), 0.23 (s, 9 H). ^{13}C NMR (101 MHz, Chloroform-*d*) δ 145.9, 145.1, 137.7, 133.6, 128.5, 128.1, 127.4, 126.2, 53.4, 28.6, 13.0, -0.9. HRMS (ESI) Calculated for $C_{18}H_{24}NaSi$ ($[M+Na]^+$): 291.1539, measured: 291.1539.

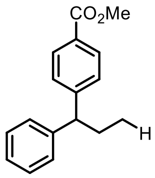

Methyl 4-(1-phenylpropyl)benzoate (3f): [9] The reaction was conducted following the general procedure **A** in a 0.5 mmol scale. The residue was purified by column chromatography on silica gel to afford the product **3f** (99.1 mg, 78% yield, *rr* = 29:1) as a colorless oil. ^{1}H NMR (400 MHz, Chloroform-*d*) δ 7.97 - 7.93 (m, 2 H), 7.32 - 7.16 (m, 7 H), 3.88 (s, 3 H), 3.84 (t, *J* = 7.8 Hz, 1 H), 2.08 (dq, *J* = 7.3, 7.3 Hz, 2 H), 0.89 (t, *J* = 7.3 Hz, 3 H). ^{13}C NMR (101 MHz, Chloroform-*d*) δ 167.2, 150.7, 144.3, 129.9, 128.6, 128.1, 128.1, 128.0, 126.4, 53.3, 52.1, 28.5, 12.8.

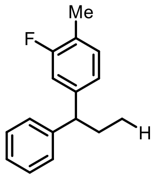

2-Fluoro-1-methyl-4-(1-phenylpropyl)benzene (3g): The reaction was conducted following the general procedure **A** in a 0.5 mmol scale. The residue was purified by column chromatography on silica gel to afford the product **3g** (93.5 mg, 82% yield, *rr* = 21:1) as a colorless oil. ^{1}H NMR (400 MHz, Chloroform-*d*) δ 7.29 - 7.27 (m, 2 H), 7.22 - 7.15 (m, 3 H), 7.06 (t, *J* = 7.9 Hz, 1 H), 6.91 - 6.86 (m, 2 H), 3.74 (t, *J* = 7.8 Hz, 1 H), 2.21 (d, *J* = 1.8 Hz, 3 H), 2.03 (dq, *J* = 7.4 Hz, 7.4 Hz, 2 H), 0.89 (t, *J* = 7.3 Hz, 3 H). ^{13}C NMR (101 MHz, Chloroform-*d*) δ 161.4 (d, *J* = 244.2 Hz), 145.1 (d, *J* = 6.7 Hz), 144.9, 131.3 (d, *J* = 5.5 Hz), 128.6, 127.9, 126.3, 123.4 (d, *J* = 3.2 Hz), 122.3 (d, *J* = 17.2 Hz), 114.4 (d, *J* = 22.1 Hz), 52.8 (d, *J* = 1.5 Hz), 28.6, 14.3 (d, *J* = 3.4 Hz), 12.8. ^{19}F NMR (377 MHz, Chloroform-*d*) δ -117.78. HRMS (ESI) Calculated for $C_{16}H_{18}F$ ($[M+H]^+$): 229.1398, measured: 229.1387.

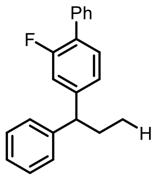

2-Fluoro-4-(1-phenylpropyl)-1,1'-biphenyl (3h): The reaction was conducted following the general procedure **A** in a 0.5 mmol scale. The residue was purified by column chromatography on silica gel to afford the product **3h** (123.3 mg, 85% yield, *rr* = 20:1) as a colorless oil. ^{1}H NMR (400 MHz, Chloroform-*d*) δ 7.52 - 7.49 (m, 2 H), 7.44 - 7.38 (m, 2 H), 7.35 - 7.24 (m, 6 H), 7.22 - 7.17 (m, 1 H), 7.11 - 6.98 (m, 2 H), 3.80 (t, *J* = 7.8 Hz, 1 H), 2.12 - 2.03 (m, 2 H), 0.92 (t, *J* = 7.3 Hz, 3 H). ^{13}C NMR (101 MHz, Chloroform-*d*) δ 159.8 (d, *J* = 247.7 Hz), 147.1 (d, *J* = 7.2 Hz), 144.5, 135.9 (d, *J* = 1.3 Hz), 130.6 (d, *J* = 4.0 Hz), 129.0 (d, *J* = 3.0 Hz), 128.7, 128.5, 128.0, 127.6, 126.7 (d, *J* = 13.6 Hz), 126.5, 124.0 (d, *J* = 3.1 Hz), 115.5 (d, *J* = 23.0 Hz), 52.9 (d, *J* = 1.4 Hz), 28.6, 12.9. ^{19}F NMR (377 MHz, Chloroform-*d*) δ -118.21. HRMS (ESI) Calculated for $C_{21}H_{19}FNa$ ($[M+Na]^+$): 313.1363, measured: 313.1367.

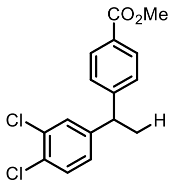

Methyl 4-(1-(3, 4-dichlorophenyl)ethyl)benzoate (3i): The reaction was conducted following the general procedure **A** in a 0.5 mmol scale. The residue was purified by column chromatography on silica gel to afford the product **3i** (117.5 mg, 76% yield, *rr* = 26:1) as a colorless oil. ^{1}H NMR (400 MHz, Chloroform-*d*) δ 7.98-7.96 (m, 2 H), 7.35 (d, *J* = 8.3 Hz, 1 H), 7.28 - 7.24 (m, 3 H), 7.02 (dd, *J* = 8.3, 2.1 Hz, 1 H), 4.15 (q, *J* = 7.2 Hz, 1 H), 3.90 (s, 3 H), 1.62 (d, *J* = 7.2 Hz, 3 H). ^{13}C NMR (101 MHz, Chloroform-*d*) δ 167.0, 150.4, 145.8, 132.6, 130.5, 130.4, 130.1, 129.7, 128.6, 127.7, 127.2, 52.2, 44.2, 21.5. HRMS (ESI) Calculated for $C_{16}H_{14}Cl_2NaO_2$ ($[M+Na]^+$): 331.0263, measured: 331.0264.

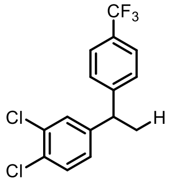

1,2-Dichloro-4-(1-(4-(trifluoromethyl)phenyl)ethyl)benzene (3j): The reaction was conducted following the general procedure **A** in a 0.5 mmol scale. The residue was purified by column chromatography on silica gel to afford the product **3j** (108.5 mg, 68% yield, *rr* = 17:1) as a colorless oil.^{1}H NMR (400 MHz, Chloroform-*d*) δ 7.55 (d, *J* = 8.3 Hz, 2 H), 7.36 - 7.28 (m, 4 H), 7.03 - 7.00 (m, 1 H), 4.16 (q, *J* = 7.2 Hz, 1 H), 1.62 (d, *J* = 7.2 Hz, 3 H). ^{13}C NMR (101 MHz, Chloroform-*d*) δ 149.1, 145.5, 132.6, 130.5, 130.5, 129.6, 127.9, 128.8 (q, *J* = 32.5 Hz), 127.1, 126.8 (q, *J* = 273.3 Hz), 125.6 (q, *J* = 3.9 Hz), 43.9, 21.4. ^{19}F NMR (377 MHz, Chloroform-*d*) δ -62.37. HRMS (ESI) Calculated for $C_{15}H_{11}Cl_2F_3Na$ ($[M+Na]^+$): 341.0088, measured: 341.0082.

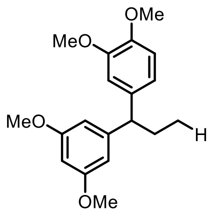

4-(1-(3,5-Dimethoxyphenyl)propyl)-1,2-dimethoxybenzene (3k): The reaction was conducted following the general procedure **A** in a 0.5 mmol scale. The residue was purified by column chromatography on silica gel to afford the product **3k** (128.0 mg, 81% yield, *rr* = 16:1) as a colorless oil. ^{1}H NMR (400 MHz, Chloroform-*d*) δ 6.82 - 6.77 (m, 2 H), 6.74 (d, *J* = 1.6 Hz, 1 H), 6.40 (d, *J* = 2.3 Hz, 2 H), 6.28 (t, *J* = 2.3 Hz, 1 H), 3.84 (s, 6 H), 3.75 (s, 6 H), 3.65 (t, *J* = 7.8 Hz, 1 H), 2.06 - 1.95 (m, 2 H), 0.90 (t, *J* = 7.3 Hz, 3 H). ^{13}C NMR (101 MHz, Chloroform-*d*) δ 160.7, 148.8, 148.0, 147.4, 137.5, 119.6, 111.3, 111.1, 106.2, 97.5, 55.9, 55.9, 55.3, 53.1, 28.8, 12.9. HRMS (ESI) Calculated for $C_{19}H_{24}NaO_4$ ($[M+Na]^+$): 339.1567, measured: 339.1569.

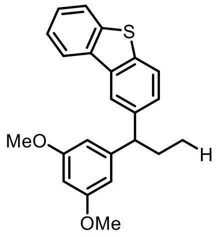

2-(1-(3,5-Dimethoxyphenyl)propyl)dibenzo[b,d]thiophene (3l): The reaction was conducted following the general procedure **A** in a 0.5 mmol scale. The residue was purified by column chromatography on silica gel to afford the product **3l** (154.0 mg, 85% yield, *rr* = 15:1) as a colorless oil. ^{1}H NMR (400 MHz, Chloroform-*d*) δ 8.16 - 8.11 (m, 1 H), 8.02 (d, *J* = 1.7 Hz, 1H) 7.84 - 7.80 (m, 1 H), 7.74 (d, *J* = 8.3 Hz, 1 H), 7.45 - 7.40 (m, 2 H), 7.34 (dd, *J* = 8.3, 1.8 Hz, 1 H), 6.47 (d, *J* = 2.2 Hz, 2 H), 6.30 (t, *J* = 2.3 Hz, 1 H), 3.90 (t, *J* = 7.8 Hz, 1 H), 3.75 (s, 6 H), 2.19 - 2.11 (m, 2 H), 0.95 (t, *J* = 7.3 Hz, 3 H).^{13}C NMR (101 MHz, Chloroform-*d*) δ 160.8, 147.8, 141.4, 139.9, 137.3, 135.8, 135.6, 127.1, 126.7, 124.3, 123.0, 122.8, 121.7, 120.7, 106.4, 97.7, 55.4, 53.5, 28.8, 13.0. HRMS (ESI) Calculated for $C_{23}H_{22}NaO_2S$ ($[M+Na]^+$): 385.1232, measured: 385.1239.

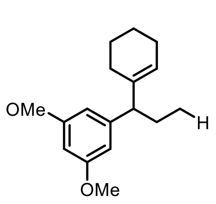

1-(1-(cyclohex-1-en-1-yl)propyl)-3,5-dimethoxybenzene (3m): The reaction was conducted following the general procedure **A** in a 0.5 mmol scale. The residue was purified by column chromatography on silica gel to afford the product **3m** (84.5 mg, 65 % yield, rr > 20:1) as a colorless oil. ^{1}H NMR (400 MHz, Chloroform-*d*) δ 6.37 (d, J = 2.3 Hz, 2 H), 6.30 (t, J = 2.3 Hz, 1 H), 5.60 (td, J = 3.8, 2.3 Hz, 1 H), 3.77 (s, 6 H), 2.91 (t, J = 7.6 Hz, 1 H), 2.05 - 2.0 (m, 2 H), 1.82 - 1.63 (m, 4 H), 1.52 (t, J = 3.2 Hz, 4 H), 0.84 (t, J = 7.3 Hz, 3 H). ^{13}C NMR (101 MHz, Chloroform-*d*) δ 160.5, 147.4, 139.8, 121.5, 106.3, 97.5, 55.3, 55.2, 26.7, 25.6, 25.5, 23.2, 22.8, 12.7. HRMS (ESI) Calculated for $C_{17}H_{25}O_2$ ([M+H]$^+$): 261.1849, measured: 261.1849.

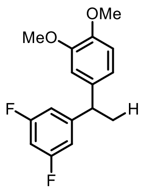

4-(1-(3, 5-Difluorophenyl)ethyl)-1, 2-dimethoxybenzene (3n): The reaction was conducted following the general procedure **A** in a 0.5 mmol scale. The residue was purified by column chromatography on silica gel to afford the product **3n** (90.2 mg, 65% yield, *rr* = 35:1) as a colorless oil. ^{1}H NMR (400 MHz, Chloroform-*d*) δ 6.85 - 6.80 (m, 1 H), 6.80 - 6.68 (m, 3 H), 6.68 (d, *J* = 2.0 Hz, 1 H), 6.61 (tt, *J* = 8.9, 2.3 Hz, 1 H), 4.06 (q, *J* = 7.2 Hz, 1 H), 3.86 (s, 3 H), 3.84 (s, 3 H), 1.59 (d, *J* = 7.2 Hz, 3 H). ^{13}C NMR (101 MHz, Chloroform-*d*) δ 163.0 (dd, *J* = 247.7, 12.9 Hz), 150.8 (t, *J* = 8.3 Hz), 149.0, 147.8, 137.5, 119.3, 111.2, 111.0, 110.4 (dd, *J* = 18.0, 6.7 Hz), 101.5 (t, *J* = 25.4 Hz), 55.9, 55.9, 44.2 (t, *J* = 1.8 Hz), 21.8. ^{19}F NMR (377 MHz, Chloroform-*d*) δ -110.24. HRMS (ESI) Calculated for $C_{16}H_{16}F_2NaO_2$ ([M+Na]$^+$): 301.1016, measured: 301.1013.

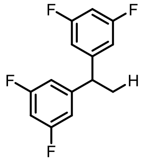

5, 5'-(Ethane-1, 1-diyl)bis(1, 3-difluorobenzene) (3o): The reaction was conducted following the general procedure **A** in a 0.5 mmol scale. The residue was purified by column chromatography on silica gel to afford the product **3o** (96.5 mg, 76% yield, *rr* = 24:1) as a colorless oil. ^{1}H NMR (400 MHz, Chloroform-*d*) δ 6.74 - 6.64 (m, 6 H), 4.08 (q, *J* = 7.2 Hz, 1 H), 1.59 (d, *J* = 7.3 Hz, 3 H). ^{13}C NMR (101 MHz, Chloroform-*d*) δ 163.2 (dd, *J* = 248.6, 12.8 Hz), 148.9 (t, *J* = 8.5 Hz), 110.6 (dd, *J* = 18.2, 6.1 Hz), 102.2 (t, *J* = 25.3 Hz), 44.3, 21.3. ^{19}F NMR (377 MHz, Chloroform-*d*) δ -109.57. HRMS (ESI) Calculated for $C_{14}H_{10}F_4Na$ ([M+Na]$^+$): 277.2174, measured: 277.2177.

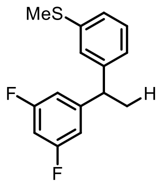

(3-(1-(3, 5-Difluorophenyl)ethyl)phenyl)(methyl)sulfane (3p): The reaction was conducted following the general procedure **A** in a 0.5 mmol scale. The residue was purified by column chromatography on silica gel to afford the product **3p** (118.8 mg, 90% yield, *rr* = 31:1) as a colorless oil. ^{1}H NMR (400 MHz, Chloroform-*d*) δ 7.22 (td, *J* = 7.6, 0.8 Hz, 1 H), 7.11 - 7.08 (m, 2 H), 6.96 - 6.93 (m, 1 H), 6.75 - 6.69 (m, 2 H), 6.62 (tt, *J* = 8.9, 2.3 Hz, 1 H), 4.06 (q, *J* = 7.2 Hz, 1 H), 2.46 (s, 3 H), 1.59 (d, *J* = 7.2 Hz, 3 H). ^{13}C NMR (101 MHz, Chloroform-*d*) δ 163.1 (dd, *J* = 248.0, 13.0 Hz), 150.1 (t, *J* = 8.4 Hz), 145.6, 138.9, 129.2, 125.8, 124.6, 124.4, 110.6 (dd, *J* = 18.5, 6.6 Hz), 101.7 (t, *J* = 25.4 Hz), 44.6 (t, *J* =

1.9 Hz), 21.5, 15.8. ^{19}F NMR (377 MHz, Chloroform-*d*) δ -110.07. HRMS (ESI) Calculated for $C_{15}H_{14}F_2NaS$ ([M+Na]$^+$): 265.0857, measured: 265.0846.

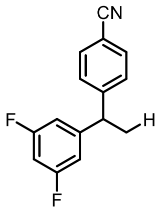

4-(1-(3, 5-Difluorophenyl)ethyl)benzonitrile (3q): The reaction was conducted following the general procedure **A** in a 0.5 mmol scale. The residue was purified by column chromatography on silica gel to afford the product **3q** (103.3 mg, 85% yield, *rr* = 24:1) as a colorless oil. ^{1}H NMR (400 MHz, Chloroform-*d*) δ 7.62 -7.59 (m, 2 H), 7.31 -7.28 (m, 2 H), 6.73 - 6.64 (m, 3 H), 4.17 (q, *J* = 7.2 Hz, 1 H), 1.63 (d, *J* = 7.2 Hz, 3 H). ^{13}C NMR (101 MHz, Chloroform-*d*) δ 163.2 (dd, *J* = 248.8, 12.8 Hz), 150.4, 148.7 (t, *J* = 8.5 Hz), 132.6, 128.4, 118.9, 110.6 (dd, *J* = 18.3, 6.9 Hz), 102.3 (t, *J* = 25.3 Hz), 44.7 (t, *J* = 1.9 Hz), 21.2. ^{19}F NMR (377 MHz, Chloroform-*d*) δ -109.38. HRMS (ESI) Calculated for $C_{15}H_{11}F_2NNa$ ([M+Na]$^+$): 266.0757, measured: 266.0756.

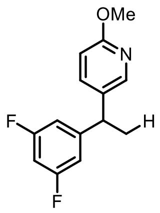

5-(1-(3, 5-Difluorophenyl)ethyl)-2-methoxypyridine (3r): The reaction was conducted following the general procedure **A** in a 0.5 mmol scale. The residue was purified by column chromatography on silica gel with petroleum ether to afford the product **3r** (92.1 mg, 74% yield, *rr* = 27:1) as a colorless oil. ^{1}H NMR (400 MHz, Chloroform-*d*) δ 8.03 (d, *J* = 2.5 Hz, 1 H), 7.35 (dd, *J* = 8.6, 2.6 Hz, 1 H), 6.74 - 6.61 (m, 4 H), 4.07 (q, *J* = 7.2 Hz, 1 H), 3.92 (s, 3 H), 1.60 (d, *J* = 7.2 Hz, 3 H). ^{13}C NMR (101 MHz, Chloroform-*d*) δ 163.2 (dd, *J* = 248.3, 12.8 Hz), 163.2, 149.8 (t, *J* = 8.4 Hz), 145.4, 138.1, 133.0, 111.1, 110.5 (dd, *J* = 19.0, 5.6 Hz), 101.9 (t, *J* = 25.4 Hz), 53.6, 41.5 (t, *J* = 1.6 Hz), 21.5. ^{19}F NMR (377 MHz, Chloroform-*d*) δ -109.84. HRMS (ESI) Calculated for $C_{14}H_{13}F_2NNaO$ ([M+Na]$^+$): 272.0857, measured: 272.0860.

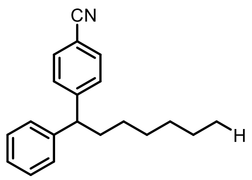

4-(1-phenylheptyl)benzonitrile (3s): The reaction was conducted following the general procedure **A** in a 0.5 mmol scale. The residue was purified by column chromatography on silica gel to afford the product **3s** (71.5 mg, 52 % yield, *rr* = 14:1) as a colorless oil.^{1}H NMR (400 MHz, Chloroform-*d*) δ 7.48 - 7.45 (m, 2 H), 7.26 - 7.19 (m, 4 H), 7.13 - 7.10 (m, 3 H), 3.85 (t, *J* = 7.8 Hz, 1 H), 2.02 - 1.88 (m, 2 H), 1.26 - 1.11 (m, 8 H), 0.77 (t, *J* = 6.9 Hz, 3 H). ^{13}C NMR (101 MHz, Chloroform-*d*) δ 151.1, 143.7, 132.4, 128.7, 128.8, 127.9, 126.7, 119.1, 110.0, 51.6, 35.4, 31.8, 29.3, 27.9, 22.7, 14.2. HRMS (ESI) Calculated for $C_{20}H_{23}NNa$ ([M+Na]$^+$): 300.1723, measured: 300.1727.

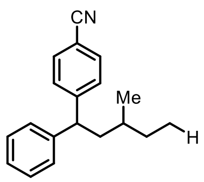

4-(3-methyl-1-phenylpentyl)benzonitrile (3t): The reaction was conducted following the general procedure **A** in a 0.5 mmol scale. The residue was purified by column chromatography on silica gel to afford the product **3t** (19.7 mg, 15 % yield, dr = 1:1, rr = 1:1) as a colorless oil.1H NMR (400 MHz, Chloroform-*d*) δ 7.49 (dd, J = 8.3, 6.4 Hz, 2 H), 7.27 (dd, J = 8.4, 4.6 Hz, 2 H), 7.22 (dd, J = 7.4, 5.7 Hz, 2 H), 7.16 - 7.12 (m, 3 H), 4.01 (t, J = 8.3 Hz, 1 H), 2.07 - 1.96 (m, 1 H), 1.77 - 1.63 (m, 1 H), 1.34 - 1.28 (m, 1 H), 1.22 - 1.18 (m, 1 H), 1.16 - 1.08 (m, 2 H), 0.82 (d, J = 6.1 Hz, 3 H), 0.76 (t,

J = 7.1 Hz, 3 H). ^{13}C NMR (101 MHz, Chloroform-*d*) δ 151.5, 150.8, 144.1, 143.3, 132.4, 132.4, 128.9, 128.8, 128.8, 128.7, 128.1, 127.8, 126.8, 126.7, 119.2, 119.2, 110.0, 110.0, 48.9, 42.4, 31.8, 29.7, 29.5, 19.2, 19.1, 11.2, 11.2. HRMS (ESI) Calculated for $C_{19}H_{21}NNa$ ([M+Na]$^+$): 286.1566, measured: 286.1575.

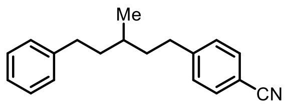

4-(3-methyl-5-phenylpentyl)benzonitrile (4t): ^{1}H NMR (400 MHz, Chloroform-*d*) δ 7.48 (d, J = 8.3 Hz, 2 H), 7.25 - 7.13 (m, 4 H), 7.16 - 7.05 (m, 3 H), 2.70 - 2.39 (m, 4 H), 1.68 - 1.51 (m, 2 H), 1.48 - 1.33 (m, 3 H), 0.93 (d, J = 5.8 Hz, 3 H). ^{13}C NMR (101 MHz, Chloroform-*d*) δ 148.8, 142.8, 132.3, 129.3, 128.5, 125.8, 119.3, 109.6, 38.8, 38.4, 33.7, 33.5, 32.1, 19.6. HRMS (ESI) Calculated for $C_{19}H_{21}NNa$ ([M+Na]$^+$): 286.1566, measured: 286.1575

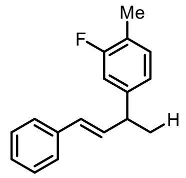

(*E*)-2-Fluoro-1-methyl-4-(4-phenylbut-3-en-2-yl)benzene (3u): The reaction was conducted following the general procedure A in a 0.5 mmol scale. The residue was purified by column chromatography on silica gel with petroleum ether to afford the product **3u** (88.8 mg, 74% yield, *rr* > 20:1) as a colorless oil. ^{1}H NMR (400 MHz, Chloroform-*d*) δ 7.38 - 7.32 (m, 2 H), 7.32 - 7.25 (m, 2 H), 7.22 - 7.16 (m, 1 H), 7.15 - 7.07 (m, 1 H), 6.97 - 6.88 (m, 2 H), 6.40 (d, *J* = 16.0 Hz, 1 H), 6.32 (dd, *J* = 15.9, 6.4 Hz, 1 H), 3.62 - 3.56 (m, 1 H), 2.24 (d, *J* = 1.8 Hz, 3 H), 1.43 (d, *J* = 7.0 Hz, 3 H). ^{13}C NMR (101 MHz, Chloroform-*d*) δ 161.5 (d, *J* = 244.5 Hz), 145.5 (d, *J* = 6.7 Hz), 137.5, 134.8, 131.4 (d, *J* = 5.5 Hz), 128.9, 128.6, 127.3, 126.3, 122.7 (d, *J* = 3.1 Hz), 122.5 (d, *J* = 17.3 Hz), 113.9 (d, *J* = 22.2 Hz), 42.1 (d, *J* = 1.7 Hz), 21.2, 14.3 (d, *J* = 3.5 Hz). ^{19}F NMR (377 MHz, Chloroform-*d*) δ -117.57. HRMS (ESI) Calculated for $C_{17}H_{17}FNa$ ([M+Na]$^+$): 263.1212, measured: 263.1217.

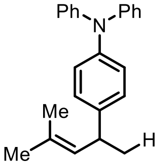

4-(4-methylpent-3-en-2-yl)-N, N-diphenylaniline (3v): The reaction was conducted following the general procedure **A** in a 0.5 mmol scale. The residue was purified by column chromatography on silica gel to afford the product **3v** (58.8 mg, 37 % yield, rr = 5:1) as a colorless oil. ^{1}H NMR (400 MHz, Chloroform-*d*) δ 7.25 - 7.19 (m, 4 H), 7.11 - 7.06 (m, 6 H), 7.02 - 6.95 (m, 4 H), 5.27 - 5.24 (m, 1 H), 3.65 - 3.58 (m, 1 H), 1.70 (dd, J = 11.0, 1.4 Hz, 6 H), 1.28 (d, J = 7.0 Hz, 3 H). ^{13}C NMR (101 MHz, Chloroform-*d*) δ 148.1, 145.4, 142.0, 130.4, 129.2, 127.7, 124.7, 123.9, 123.8, 122.4, 37.6, 26.0, 22.5, 18.1. HRMS (ESI) Calculated for $C_{24}H_{25}NNa$ ([M+Na]$^+$): 350.1885, measured: 350.1884.

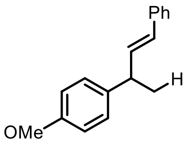

(*E*)-1-methoxy-4-(4-phenylbut-3-en-2-yl)benzene (3w): The reaction was conducted following the general procedure **A** in a 0.5 mmol scale. The residue was purified by column chromatography on silica gel to afford the product **3w** (xx mg, 89 % yield, rr = 49:1) as a colorless oil. ^{1}H NMR (400 MHz, Chloroform-*d*) δ 7.36 - 7.34 (m, 2 H), 7.28 (dd, J = 8.5, 6.8 Hz, 2 H), 7.20 - 7.17 (m, 3 H), 6.88 - 6.84 (m, 2 H), 6.37 (d, J = 4.7 Hz, 2 H), 3.78 (s, 3 H), 3.62 - 3.54 (m, 1 H), 1.44 (d, J = 7.0 Hz, 3 H). ^{13}C NMR (101 MHz, Chloroform-*d*) δ 158.1, 137.8, 137.7, 135.7, 128.6, 128.3, 128.3, 127.1, 126.2, 114.0, 55.4, 41.8, 21.4. HRMS (ESI) Calculated for $C_{17}H_{19}O$ ([M+H]$^+$): 261.1250, measured: 261.1250.

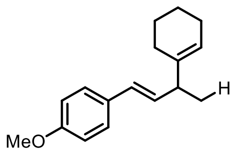

(*E*)-1-(3-(cyclohex-1-en-1-yl)but-1-en-1-yl)-4-methoxybenzene (3x): The reaction was conducted following the general procedure **A** in a 0.5 mmol scale. The residue was purified by column chromatography on silica gel to afford the product **3x** (72.0 mg, 60 % yield, *rr* = 71:1) as a colorless oil. ^{1}H NMR (400 MHz, Chloroform-*d*) δ 7.22 (d, J = 8.8 Hz, 2 H), 6.76 (d, J = 8.7 Hz,2 H), 6.22 (d, J = 15.9 Hz, 1 H), 5.95 (dd, J = 15.9, 7.4 Hz, 1 H), 5.43 (d, J = 1.1 Hz, 1 H), 3.73 (s, 3 H), 2.79 - 2.72 (m, 1 H), 1.95 - 1.86 (m, 4 H), 1.55 - 1.45 (m, 4 H), 1.10 (d, J = 6.9 Hz, 3 H). ^{13}C NMR (101 MHz, Chloroform-*d*) δ 158.7, 141.2, 133.2, 130.8, 127.8, 127.2, 120.5, 114.0, 55.4, 44.3, 26.7, 25.5, 23.2, 22.8, 18.7. HRMS (ESI) Calculated for $C_{17}H_{23}O$ ($[M+H]^+$): 243.1749, measured: 243.1749.

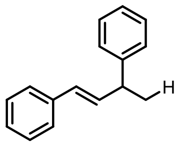

(*E*)-But-1-ene-1, 3-diyldibenzene (3y): [10] The reaction was conducted following the general procedure **A** in a 0.5 mmol scale. The residue was purified by column chromatography on silica gel to afford the product **3y** (83.3 mg, 80% yield, *rr* > 20:1) as a colorless oil. ^{1}H NMR (400 MHz, Chloroform-*d*) δ 7.36 - 7.25 (m, 8 H), 7.22 - 7.16 (m, 2 H), 6.44 - 6.34 (m, 2H), 3.66 - 3.60 (m, 1H), 1.46 (d, *J* = 7.0 Hz, 3H). ^{13}C NMR (101 MHz, Chloroform-*d*) δ 145.7, 137.6, 135.3, 128.6, 127.4, 127.2, 126.4, 126.3, 126.3, 42.7, 21.4.

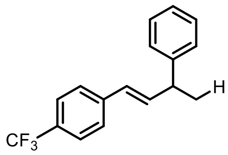

(*E*)-1-(3-Phenylbut-1-en-1-yl)-4-(trifluoromethyl)benzene (3z) : [10] The reaction was conducted following the general procedure **A** in a 0.5 mmol scale. The residue was purified by column chromatography on silica gel to afford the product **3z** (92.5 mg, 67% yield, *rr* > 20:1) as a colorless oil. ^{1}H NMR (400 MHz, Chloroform-*d*) δ 7.53 (d, *J* = 8.2 Hz, 2 H), 7.43 (d, *J* = 8.2 Hz, 2 H), 7.35 - 7.31 (m, 2 H), 7.28 - 7.21 (m, 3 H), 6.52 - 6.40 (m, 2 H), 3.69 - 3.60 (m, 1 H), 1.48 (d, *J* = 7.1 Hz, 3 H). ^{13}C NMR (101 MHz, Chloroform-*d*) δ 145.1, 141.2, 138.1, 129.0 (q, *J* = 32 Hz), 128.7, 127.4, 127.4, 126.6, 126.4, 125.6 (q, *J* = 3.8 Hz), 124.4 (q, *J* = 271.9 Hz), 42.8, 21.2. ^{19}F NMR (377 MHz, Chloroform-*d*) δ -62.35.

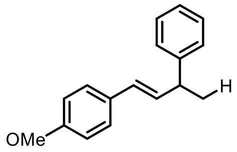

(*E*)-1-Methoxy-4-(3-phenylbut-1-en-1-yl)benzene (3aa): [10] The reaction was conducted following the general procedure **A** in a 0.5 mmol scale. The residue was purified by column chromatography on silica gel to afford the product **3aa** (91.6 mg, 77% yield, *rr* > 20:1) as a colorless oil. 1H NMR (400 MHz, Chloroform-*d*) δ 7.33 - 7.25 (m, 6 H), 7.22 - 7.17 (m, 1 H), 6.84 - 6.80 (m, 2 H), 6.35 (dd, *J* = 15.9, 1.2 Hz, 1 H), 6.23 (dd, *J* = 15.9, 6.7 Hz, 1 H), 3.78 (s, 3 H), 3.64 - 3.57 (m, 1 H), 1.44 (d, *J* = 7.0 Hz, 3 H). 13C NMR (101 MHz, Chloroform-*d*) δ 158.9, 146.0, 133.2, 130.5, 128.6, 128.0, 127.4, 127.4, 126.3, 114.0, 55.4, 42.7, 21.5.

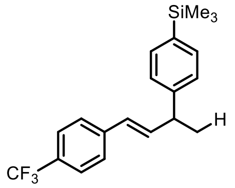

(*E*)-Trimethyl(4-(4-(4-(trifluoromethyl)phenyl)but-3-en-2-yl)phenyl)silane (3ab): The reaction was conducted following the general procedure **A** in a 0.5 mmol scale. The residue was purified by column chromatography on silica gel to afford the product **3ab** (130.1 mg, 75% yield, *rr* = 37:1) as a colorless oil. ^{1}H NMR (400 MHz, Chloroform-*d*) δ 7.53 - 7.48 (m, 4 H), 7.42 (d, *J* = 8.1 Hz, 2 H), 7.26 (d, *J* = 8.0 Hz, 2 H), 6.52 - 6.39 (m, 2 H), 3.68 - 3.62 (m, 1 H), 1.48 (d, *J* = 7.0 Hz, 3 H), 0.26 (s, 9 H). ^{13}C NMR (101 MHz, Chloroform-*d*) δ 145.8, 141.2, 138.4, 138.0, 133.8, 129.0 (q, *J* = 32.4 Hz), 127.5, 126.9, 126.4, 125.6 (q, *J* = 3.8 Hz), 124.4 (q, *J* = 273 Hz), 42.8, 21.0, -0.9. ^{19}F NMR (377 MHz, Chloroform-*d*) δ -62.31. HRMS (ESI) Calculated for $C_{20}H_{23}F_3NaSi$ ([M+Na]$^+$): 371.1419, measured: 371.1422.

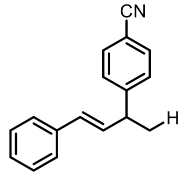

(*E*)-4-(4-phenylbut-3-en-2-yl)benzonitrile (3ac): [11] The reaction was conducted following the general procedure **A** in a 0.5 mmol scale. The residue was purified by column chromatography on silica gel to afford the product **3ac** (78.1 mg, 67% yield, *rr* > 20:1) as a colorless oil. ^{1}H NMR (400 MHz, Chloroform-*d*) δ 7.61 - 7.58 (m, 2 H), 7.39 - 7.28 (m, 6 H), 7.25 - 7.20 (m, 1 H), 6.42 (dd, *J* = 15.9, 1.1 Hz, 1 H), 6.32 (d, *J* = 6.7 Hz, 1 H), 3.73 - 3.66 (m, 1 H), 1.47 (d, *J* = 7.1 Hz, 3 H). ^{13}C NMR (101 MHz, Chloroform-*d*) δ 151.2, 137.0, 133.4, 132.5, 129.8, 128.7, 128.2, 127.6, 126.3, 119.2, 110.1, 42.8, 21.0.

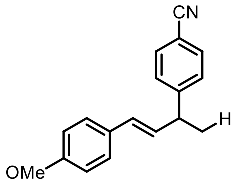

(*E*)-4-(4-(4-methoxyphenyl)but-3-en-2-yl)benzonitrile (3ad): The reaction was conducted following the general procedure **A** in a 0.5 mmol scale. The residue was purified by column chromatography on silica gel to afford the product **3ad** (80.2 mg, 61% yield, *rr* = 31:1) as a colorless oil. ^{1}H NMR (400 MHz, Chloroform-*d*) δ 7.59 - 7.57 (m, 2 H), 7.37 - 7.35 (m, 2 H), 7.29 - 7.35 (m, 2 H), 6.85 - 6.82 (m, 2 H), 6.36 (dd, *J* = 15.9, 1.2 Hz, 1 H), 6.16 (dd, *J* = 15.9, 6.9 Hz, 1 H), 3.78 (s, 3 H), 3.69 - 3.63 (m, 1 H), 1.45 (d, *J* = 7.0 Hz, 3 H). ^{13}C NMR (101 MHz, Chloroform-*d*) δ 159.1, 151.5, 132.4, 131.2, 129.8, 129.1, 128.2, 127.4, 119.2, 114.0, 110.0, 55.4, 42.7, 21.1. HRMS (ESI) Calculated for $C_{18}H_{17}NNaO$ ([M+Na]$^+$): 286.1202, measured: 286.1208.

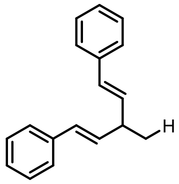

((1*E*, 4*E*)-3-methylpenta-1, 4-diene-1,5-diyl)dibenzene (3ae) : [10] The reaction was conducted following the general procedure **A** in a 0.5 mmol scale. The residue was purified by column chromatography on silica gel to afford the product **3e** (105.3 mg, 90% yield, *rr* > 20:1) as a colorless oil. ^{1}H NMR (400 MHz, Chloroform-*d*) δ 7.39 - 7.18 (m, 10 H), 6.43 (dd, *J* = 16.1, 1.2 Hz, 2 H), 6.24 (dd, *J* = 15.9, 6.9 Hz, 2 H), 3.26 - 3.16 (m, 1 H), 1.30 (d, *J* = 6.9 Hz, 3 H). ^{13}C NMR (101 MHz, Chloroform-*d*) δ 137.7, 134.4, 128.9, 128.6, 127.2, 126.2, 40.2, 20.4.

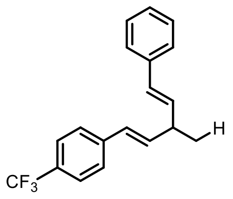

1-((1*E*, 4*E*)-3-methyl-5-phenylpenta-1, 4-dien-1-yl)-4-(trifluoromethyl) benzene (3af): The reaction was conducted following the general procedure **A** in a 0.5 mmol scale. The residue was purified by column chromatography on silica gel to afford the product **3af** (90.6 mg, 66% yield, *rr* > 20:1) as a colorless oil. ^{1}H NMR (400 MHz, Chloroform-*d*) δ 7.54 (d, *J* = 8.1 Hz, 2 H), 7.45 (d, *J* = 8.1 Hz, 2 H), 7.39 - 7.37 (m, 2 H), 7.33 - 7.29 (m, 2 H), 7.24 - 7.20 (m, 1 H), 6.44 (dd, *J* = 16.0, 6.8 Hz, 2 H), 6.34 (dd, *J* = 16.0, 6.6 Hz, 1 H), 6.27 - 6.19 (m, 1 H), 3.28 - 3.19 (m, 1 H), 1.31 (d, *J* = 6.9 Hz, 3 H). ^{13}C NMR (101 MHz, Chloroform-*d*) δ 141.2, 137.5, 137.2, 133.7, 129.4, 128.7 (q, *J* = 20.2 Hz), 128.7, 127.8, 127.4, 126.4, 126.3, 125.8 (q, *J* = 272.9 Hz), 125.6 (q, *J* = 3.9 Hz), 40.2, 20.2. ^{19}F NMR (377 MHz, Chloroform-*d*) δ -62.36. HRMS (ESI) Calculated for $C_{19}H_{17}F_3Na$ ([M+Na]$^+$): 325.3300, measured: 325.3305.

.

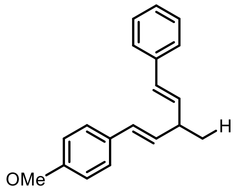

1-Methoxy-4-((1*E*, 4*E*)-3-methyl-5-phenylpenta-1,4-dien-1-yl)benzene (3ag): The reaction was conducted following the general procedure **A** in a 0.5 mmol scale. The residue was purified by column chromatography on silica gel to afford the product **3ag** (99.0 mg, 75% yield, *rr* = 35:1) as a colorless oil. ^{1}H NMR (400 MHz, Chloroform-*d*) δ 7.38 - 7.36 (m, 2 H), 7.32 - 7.28 (m, 4 H), 7.22 - 7.18 (m, 1 H), 6.84 (d, *J* = 8.7 Hz, 2 H), 6.44 - 6.35 (m, 2 H), 6.24 (dd, *J* = 15.9, 6.8 Hz, 1 H), 6.10 (dd, *J* = 15.9, 7.0 Hz, 1 H), 3.79 (s, 3 H), 3.22 - 3.14 (m, 1 H), 1.28 (d, *J* = 6.9 Hz, 3 H). ^{13}C NMR (101 MHz, Chloroform-*d*) δ 158.9, 137.8, 134.6, 132.2, 130.5, 128.7, 128.6, 128.3, 127.3, 127.1, 126.2, 114.0, 55.4, 40.2, 20.5. HRMS (ESI) Calculated for $C_{19}H_{20}NNaO$ ([M+Na]$^+$): 287.1412, measured: 287.1414.

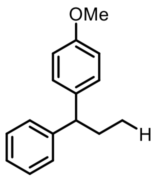

1-Methoxy-4-(1-phenylpropyl)benzene (3ah): [12] The reaction was conducted following the general procedure B in a 0.5 mmol scale. The residue was purified by column chromatography on silica gel to afford the product **3ah** (88.1 mg, 78 % yield, *rr* = 14:1) as a colorless oil. ^{1}H NMR (400 MHz, Chloroform-*d*) δ 7.28 -7.20 (m, 4 H), 7.17 - 7.12 (m, 3 H), 6.83 - 6.80 (m, 2 H), 3.76 (s, 3 H), 3.75 (t, *J* = 8.0 Hz, 1 H), 2.03 (dq, *J* = 7.4, 7.4 Hz, 2 H), 0.88 (t, *J* = 7.3 Hz, 3 H). ^{13}C NMR (101 MHz, Chloroform-*d*) δ 157.9, 145.7, 137.4, 128.9, 128.5, 127.9, 126.0, 113.8, 55.3, 52.5, 28.9, 13.0.

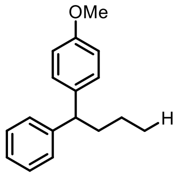

1-Methoxy-4-(1-phenylbutyl)benzene (3ai): The reaction was conducted following the general procedure B in a 0.5 mmol scale. The residue was purified by column chromatography on silica gel to afford the product **3ai** (87.6 mg, 73 % yield, *rr* = 16:1) as a colorless oil. ^{1}H NMR (400 MHz, Chloroform-*d*) δ 7.28 - 7.20 (m, 4 H), 7.17 - 7.12 (m, 3 H), 6.83 - 6.79 (m, 2 H), 3.85 (t, *J* = 7.9 Hz, 1 H), 3.75 (s, 3 H), 2.01 - 1.95 (m, 2 H), 1.32 - 1.22 (m, 2 H), 0.91 (t, *J* = 7.3 Hz, 3 H). ^{13}C NMR (101 MHz, Chloroform-*d*) δ 157.9, 145.9, 137.6, 128.9, 128.5, 127.9, 126.0, 113.8, 55.3, 50.3, 38.2, 21.3, 14.2. HRMS (ESI) Calculated for $C_{17}H_{20}NaO$ ([M+Na]$^+$): 263.1406, measured: 263.1403.

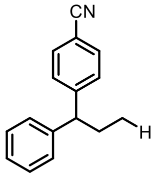

4-(1-Phenylpropyl)benzonitrile (3aj): The reaction was conducted following the general procedure B in a 0.5 mmol scale. The residue was purified by column chromatography on silica gel to afford the product **3aj** (91.7 mg, 83% yield, *rr* = 15:1) as a colorless oil. ^{1}H NMR (400 MHz, Chloroform-*d*) δ 7.56 - 7.54 (m, 2 H), 7.34 - 7.27 (m, 4 H), 7.22 - 7.18 (m, 3 H), 3.84 (t, *J* = 7.8 Hz, 1 H), 2.13 - 2.01 (m, 2 H), 0.89 (t, *J* = 7.3 Hz, 3 H). ^{13}C NMR (101 MHz, Chloroform-*d*) δ 150.9, 143.5, 132.3, 128.8, 128.8, 127.9, 126.7, 119.1, 110.0, 53.3, 28.3, 12.7. HRMS (ESI) Calculated for $C_{17}H_{20}NaO$ ($[M+Na]^+$): 244.1097, measured: 244.1100.

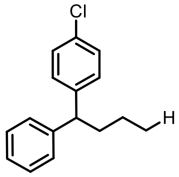

1-Chloro-4-(1-phenylbutyl)benzene (3ak): The reaction was conducted following the general procedure **B** in a 0.5 mmol scale. The residue was purified by column chromatography on silica gel to afford the product **3ak** (83.0 mg, 68% yield, *rr* = 15:1) as a colorless oil. ^{1}H NMR (400 MHz, Chloroform-*d*) δ 7.29 - 7.15 (m, 9 H), 3.88 (t, *J* = 7.8 Hz, 1 H), 2.05 - 1.92 (m, 2 H), 1.31 - 1.22 (m, 2 H), 0.91 (t, *J* = 7.3 Hz, 3 H). ^{13}C NMR (101 MHz, Chloroform-*d*) δ 144.9, 143.9, 131.8, 129.3, 128.6, 127.9, 126.3, 50.5, 37.9, 21.2, 14.2. HRMS (ESI) Calculated for $C_{16}H_{17}ClNa$ ($[M+Na]^+$): 267.0916, measured: 267.0918.

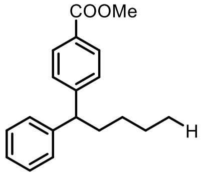

Methyl 4-(1-phenylpentyl)benzoate (3al): The reaction was conducted following the general procedure B in a 0.5 mmol scale. The residue was purified by column chromatography on silica gel to afford the product **3al** (93.1 mg, 66% yield, *rr* = 13:1) as a colorless oil. ^{1}H NMR (400 MHz, Chloroform-*d*) δ 7.99 - 7.90 (m, 2 H), 7.31 - 7.25 (m, 4 H), 7.23 - 7.15 (m, 3 H), 3.93 (t, *J* = 7.8 Hz, 1 H), 3.87 (s, 3 H), 2.08 - 2.02 (m, 2 H), 1.37 - 1.29 (m, 2 H), 1.26 - 1.18 (m, 2 H), 0.86 (t, *J* = 7.2 Hz, 3 H). ^{13}C NMR (101 MHz, Chloroform-*d*) δ 167.2, 150.9, 144.5, 129.9, 128.62, 128.0, 128.0, 127.9, 126.4, 52.1, 51.5, 35.3, 30.3, 22.8, 14.1. HRMS (ESI) Calculated for $C_{19}H_{22}NaO_2$ ($[M+Na]^+$): 305.1512, measured: 305.1518.

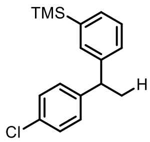

(3-(1-(4-Chlorophenyl)ethyl)phenyl)trimethylsilane (3am): The reaction was conducted following the general procedure **B** in a 0.5 mmol scale. The residue was purified by column chromatography on silica gel with petroleum ether to afford the product **3am** (110.9 mg, 77% yield, *rr* = 12:1) as a colorless oil.^{1}H NMR (400 MHz, Chloroform-*d*) δ 7.37 - 7.35 (m, 2 H), 7.29 - 7.23 (m, 3 H), 7.17 - 7.13 (m, 3 H), 4.12 (q, *J* = 7.2 Hz, 1 H), 1.62 (d, *J* = 7.2 Hz, 3 H), 0.24 (s, 9 H).^{13}C NMR (101 MHz, Chloroform-*d*) δ 145.0, 140.8, 132.6, 131.8, 131.4, 129.1, 128.6, 128.0, 128.0, 44.4, 22.1, -0.9. HRMS (ESI) Calculated for $C_{17}H_{21}ClNaSi$ ($[M+Na]^+$): 311.0999, measured: 312.0002.

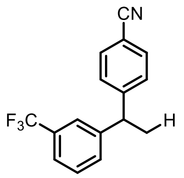

4-(1-(3-(trifluoromethyl)phenyl)ethyl)benzonitrile (3an): The reaction was conducted following the general procedure B in a 0.5 mmol scale. The residue was purified by column chromatography on silica gel to afford the product **3an** (119.6 mg, 87% yield, *rr* = 24:1) as a colorless oil. ^{1}H NMR (400 MHz, Chloroform-*d*) δ 7.61 -7.58 (m, 2 H), 7.50 - 7.41 (m, 3 H), 7.37 - 7.29 (m, 3 H), 4.27 (q, *J* = 7.2 Hz, 1 H), 1.68 (d, *J* = 7.3 Hz, 3 H). ^{13}C NMR (101 MHz, Chloroform-*d*) δ 150.9, 145.7, 132.6, 131.2 (d, *J* = 1.1 Hz), 131.0 (q, *J* = 32.1 Hz), 129.3, 128.5, 124.2 (q, *J* = 272.4 Hz), 123.7 (q, *J* = 3.8 Hz), 124.3 (q, *J* = 3.8 Hz), 119.0, 110.5, 44.8, 21.5. ^{19}F NMR (377 MHz, Chloroform-*d*) δ -62.50. HRMS (ESI) Calculated for $C_{16}H_{12}F_3NNa$ ([M+Na]$^+$): 298.0814, measured: 298.0817.

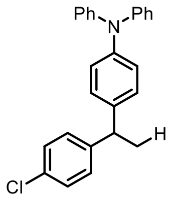

4-(1-(4-Chlorophenyl)ethyl)-*N*,*N*-diphenylaniline (3ao): The reaction was conducted following the general procedure **B** in a 0.5 mmol scale. The residue was purified by column chromatography on silica gel to afford the product **3ao** (176.2 mg, 92% yield, *rr* = 13:1) as a colorless oil. ^{1}H NMR (400 MHz, Chloroform-*d*) δ 7.26 - 7.19 (m, 6 H), 7.17 - 7.15 (m, 2 H), 7.07 - 7.04 (m, 6 H), 7.00 - 6.96 (m, 4 H), 4.06 (q, *J* = 7.2 Hz, 1 H), 1.59 (d, *J* = 7.2 Hz, 3 H). ^{13}C NMR (101 MHz, Chloroform-*d*) δ 147.9, 146.0, 145.2, 140.2, 131.8, 129.3, 129.1, 128.6, 128.3, 124.2, 124.2, 122.7, 43.8, 22.0. HRMS (ESI) Calculated for $C_{26}H_{22}ClNNa$ ([M+H]$^+$): 384.1514, measured: 384.1518.

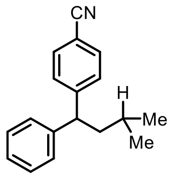

4-(3-Methyl-1-phenylbutyl)benzonitrile (3ap): The reaction was conducted following the general procedure **B** in a 6.0 mmol scale. The residue was purified by column chromatography on silica gel to afford the product **3ap** (23.7 mg, 19% yield, *rr* = 7:1) as a colorless oil. ^{1}H NMR (400 MHz, Chloroform-*d*) δ 7.56 (d, *J* = 8.0 Hz, 2 H), 7.35 - 7.28 (m, 4 H), 7.22 - 7.19 (m, 3 H), 4.07 (t, *J* = 8.0 Hz, 1 H), 2.01 - 1.84 (m, 2 H), 1.45 - 1.38 (m, 1 H), 0.93 (s, 3 H), 0.91 (s, 3 H). ^{13}C NMR (101 MHz, Chloroform-*d*) δ 151.10, 143.6, 132.4, 128.8, 128.8, 128.0, 126.8, 119.2, 110.0, 49.1, 44.6, 25.6, 22.8, 22.6. HRMS (ESI) Calculated for $C_{18}H_{19}NNa$ ([M+Na]$^+$): 272.1410, measured: 272.1414.

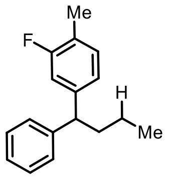

2-Fluoro-1-methyl-4-(1-phenylbutyl)benzene (3aq): The reaction was conducted following the general procedure **B** in a 0.5 mmol scale. The residue was purified by column chromatography on silica gel to afford the product **3aq** (72.6 mg, 60% yield, *rr* = 13:1) as a colorless oil. ^{1}H NMR (400 MHz, Chloroform-*d*) δ 7.28 - 7.24 (m, 2 H), 7.22 - 7.14 (m, 3 H), 7.07- 7.03 (m, 1 H), 6.91- 6.85 (m, 2 H), 3.85 (t, *J* = 7.8 Hz, 1 H), 2.20 (d, *J* = 1.8 Hz, 3 H), 2.00 - 1.95 (m, 2 H), 1.31 - 1.22 (m, 2 H), 0.91 (t, *J* = 7.3 Hz, 3 H). ^{13}C NMR (101 MHz, Chloroform-*d*) δ 161.4 (d, *J* = 244.2 Hz), 145.3 (d, *J* = 6.8 Hz), 145.0, 131.31 (d, *J* = 5.5 Hz), 128.6, 127.9, 126.3, 123.3 (d, *J* = 3.1 Hz), 122.3 (d, *J* = 17.2 Hz), 114.4 (d, *J* = 22.2 Hz), 50.6 (d, *J* = 1.7 Hz), 37.9, 21.2, 14.3 (d, *J* = 3.5 Hz), 14.2. ^{19}F NMR (377 MHz, Chloroform-*d*) δ -117.67. HRMS (ESI) Calculated for $C_{17}H_{19}FNa$ ([M+Na]$^+$): 265.1368, measured: 265.1371.

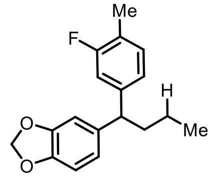

5-(1-(3-Fluoro-4-methylphenyl)butyl)benzo[d][1,3]dioxole (3ar): The reaction was conducted following the general procedure **B** in a 0.5 mmol scale. The residue was purified by column chromatography on silica gel to afford the product **3ar** (120.1 mg, 84% yield, *rr* = 10:1) as a colorless oil. ^{1}H NMR (400 MHz, Chloroform-*d*) δ 7.10 (t, *J* = 7.7 Hz, 1 H), 6.94 - 6.88 (m, 2 H), 6.77 - 6.71 (m, 3 H), 5.94 - 5.93 (m, 2 H), 3.81 (t, *J* = 7.8 Hz, 1 H), 2.25 (d, *J* = 1.8 Hz, 3 H), 1.99 - 1.93 (m, 2 H), 1.34 - 1.25 (m, 2 H), 0.95 (t, *J* = 7.3 Hz, 3 H).^{13}C NMR (101 MHz, Chloroform-*d*) δ 161.4 (d, *J* = 244.3 Hz), 147.8, 145.9, 145.4 (d, *J* = 6.7 Hz), 139.1, 131.3 (d, *J* = 5.5 Hz), 123.2, 123.1, 122.3 (d, *J* = 17.1 Hz), 120.8, 114.2 (d, *J* = 22.2 Hz), 108.2 (d, *J* = 1.8 Hz), 101.0, 50.2 (d, *J* = 1.6 Hz), 38.0, 21.2, 14.3 (d, *J* = 3.5 Hz), 14.2. ^{19}F NMR (377 MHz, Chloroform-*d*) δ -117.67. HRMS (ESI) Calculated for $C_{18}H_{19}FNaO_2$ ([M+Na]$^+$): 309.1261, measured: 309.1270.

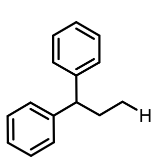

Propane-1, 1-diyldibenzene (3as) : [9] The reaction was conducted following the general procedure **C** in a 0.5 mmol scale. The residue was purified by column chromatography on silica gel to afford the product **3as** (79.4 mg, 81 % yield, *rr* = 14:1) as a colorless oil. ^{1}H NMR (400 MHz, Chloroform-*d*) δ 7.29 - 7.22 (m, 8 H), 7.18 - 7.14 (m, 2 H), 3.79 (t, *J* = 7.8 Hz, 1 H), 2.07 (dq, *J* = 7.4, *J* = 7.4 Hz, 2 H), 0.90 (t, *J* = 7.3 Hz, 3 H). ^{13}C NMR (101 MHz, Chloroform-*d*) δ 145.3, 128.5, 128.0, 126.1, 53.4, 28.7, 13.0.

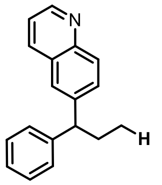

6-(1-Phenylpropyl)quinoline (3at): The reaction was conducted following the general procedure **C** in a 0.5 mmol scale. The residue was purified by column chromatography on silica gel to afford the product **3at** (59.3 mg, 48% yield, *rr* = 27:1) as a colorless oil. ^{1}H NMR (400 MHz, Chloroform-*d*) δ 8.84 (dd, *J* = 4.2, 1.7 Hz, 1 H), 8.11 - 8.09 (m, 1 H), 8.01 (d, *J* = 8.7 Hz, 1 H), 7.67 (d, *J* = 2.0 Hz, 1 H), 7.59 (dd, *J* = 8.8, 2.1 Hz, 1 H), 7.36 (dd, *J* = 8.3, 4.2 Hz, 1 H), 7.32 - 7.25 (m, 4 H), 7.22 - 7.17 (m, 1 H), 3.99 (t, *J* = 7.7 Hz, 1 H), 2.22 - 2.14 (m, 2 H), 0.94 (t, *J* = 7.3 Hz, 3 H). ^{13}C NMR (101 MHz, Chloroform-*d*) δ 150.0, 147.3, 144.6, 143.6, 136.0, 130.6, 129.5, 128.6, 128.4, 128.1, 126.4, 125.8, 121.2, 53.2, 28.5, 12.9. HRMS (ESI) Calculated for $C_{18}H_{18}N$ ([M+H]$^+$): 248.1434, measured: 248.1435.

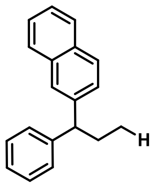

2-(1-Phenylpropyl)naphthalene (3au) : [9] The reaction was conducted following the general procedure **C** in a 0.5 mmol scale. The residue was purified by column chromatography on silica gel to afford the product **3au** (92.3 mg, 75% yield, *rr* = 16:1) as a colorless oil. ^{1}H NMR (400 MHz, Chloroform-*d*) δ 7.81 - 7.71 (m, 4 H), 7.46 - 7.39 (m, 2 H), 7.34 - 7.24 (m, 5 H), 7.20 - 7.15 (m, 1 H), 3.96 (t, *J* = 7.7 Hz, 1 H), 2.23 - 7.12 (m, 2 H), 0.94 (t, *J* = 7.3 Hz, 3 H).^{13}C NMR (101 MHz, Chloroform-*d*) δ 145.1, 142.7, 133.6, 132.2, 128.5, 128.2, 128.1, 127.8, 127.7, 127.0, 126.2, 126.0, 126.0, 125.4, 53.4, 28.5, 13.0.

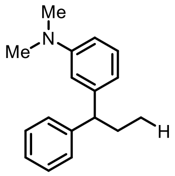

***N, N*-Dimethyl-3-(1-phenylpropyl)aniline (3av):** The reaction was conducted following the general procedure **C** in a 0.5 mmol scale. The residue was purified by column chromatography on silica gel to afford the product **3av** (72.9 mg, 61% yield, *rr* = 15:1) as a colorless oil. ^{1}H NMR (400 MHz, Chloroform-*d*) δ 7.27 - 7.23 (m, 4 H), 7.16 - 7.12 (m, 2 H), 6.63 - 6.61 (m, 2 H), 6.57 - 6.54 (m, 1 H), 3.73 (t, *J* = 7.8 Hz, 1 H), 2.89 (s, 6 H), 2.10 - 2.02 (m, 2 H), 0.90 (t, *J* = 7.3 Hz, 3 H). ^{13}C NMR (101 MHz, Chloroform-*d*) δ 150.7, 146.0, 145.5, 129.0, 128.3, 128.0, 125.9, 116.4, 112.7, 110.5, 53.8, 40.8, 28.8, 13.0. HRMS (ESI) Calculated for $C_{17}H_{21}NNa$ ([M+Na]$^{+}$): 262.1566, measured: 262.1565.

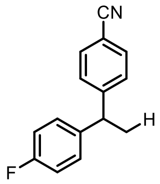

4-(1-(4-Fluorophenyl)ethyl)benzonitrile (3aw): The reaction was conducted following the general procedure **C** in a 0.5 mmol scale. The residue was purified by column chromatography on silica gel to afford the product **3aw** (103.5 mg, 92% yield, *rr* = 21:1) as a colorless oil. ^{1}H NMR (400 MHz, Chloroform-*d*) δ 7.59 - 7.57 (m, 2 H), 7.30 - 7.28 (m, 2 H), 7.16 - 7.12 (m, 2 H), 7.01 - 6.97 (m, 2 H), 4.19 (q, *J* = 7.2 Hz, 1 H), 1.63 (d, *J* = 7.2 Hz, 3 H). ^{13}C NMR (101 MHz, Chloroform-*d*) δ 161.6 (d, *J* = 245.1 Hz), 151.8, 140.5 (d, *J* = 3.3 Hz), 132.4, 129.1 (d, *J* = 7.9 Hz), 128.4, 119.1, 115.6 (d, *J* = 21.3 Hz), 110.2, 44.3, 21.7. ^{19}F NMR (377 MHz, Chloroform-*d*) δ -116.31. HRMS (ESI) Calculated for $C_{15}H_{12}FNNa$ ([M+Na]$^{+}$): 248.0851, measured: 248.0846.

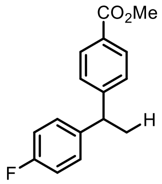

Methyl 4-(1-(4-fluorophenyl)ethyl)benzoate (3ax): The reaction was conducted following the general procedure **C** in a 0.5 mmol scale. The residue was purified by column chromatography on silica gel to afford the product **3ax** (109.7 mg, 85% yield, *rr* = 24:1) as a colorless oil. ^{1}H NMR (400 MHz, Chloroform-*d*) δ 7.98 - 7.93 (m, 2 H), 7.27 - 7.24 (m, 2 H), 7.17 - 7.12 (m, 2 H), 7.02 - 6.91 (m, 2 H), 4.17 (q, *J* = 7.2 Hz, 1 H), 3.88 (s, 3 H), 1.62 (d, *J* = 7.2 Hz, 3 H). ^{13}C NMR (101 MHz, Chloroform-*d*) δ 167.1, 161.5 (d, *J* = 244.5 Hz), 151.6, 141.2 (d, *J* = 3.3 Hz), 129.9, 129.1 (d, *J* = 7.8 Hz), 128.2, 127.7, 115.3 (d, *J* = 21.2 Hz), 52.1, 44.2, 21.8. 19F NMR (377 MHz, Chloroform-*d*) δ -116.83. HRMS (ESI) Calculated for $C_{16}H_{15}FNaO_2$ ([M+Na]$^{+}$): 259.1218, measured: 259.1129.

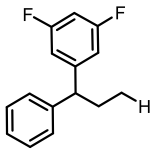

1, 3-Difluoro-5-(1-phenylpropyl)benzene (3ay): The reaction was conducted following the general procedure **A** (60 °C, 24 h)in a 0.5 mmol scale. The residue was purified by column chromatography on silica gel with petroleum ether to afford the product **3ay** (70.8 mg, 61% yield, *rr* = 11:1) as a colorless oil. ^{1}H NMR (400 MHz, Chloroform-*d*) δ 7.32 - 7.28 (m, 2 H), 7.22 - 7.18 (m, 3 H), 6.78 - 6.72 (m, 2 H), 6.60 (tt, *J* = 8.9, 2.3 Hz, 1 H), 3.75 (t, *J* = 7.8 Hz, 1 H), 2.03 (dq, *J* = 7.4, 7.4 Hz, 2 H), 0.89 (t, *J* = 7.3 Hz, 3 H). ^{13}C NMR (101 MHz, Chloroform-*d*) δ 163.1 (dd, *J* = 247.8, 12.9 Hz), 149.4 (t, *J* = 8.4 Hz), 143.8, 128.7, 127.9, 126.7, 110.8 (dd, *J* = 18.0, 6.4 Hz), 101.6 (t, *J* = 25.4 Hz), 53.1 (d, *J* = 1.9 Hz), 28.4, 12.7. ^{19}F NMR (377 MHz, Chloroform-*d*) δ -110.32. HRMS (ESI) Calculated for $C_{15}H_{15}F_2$ ([M+H]$^{+}$): 233.1136, measured: 233.1142.

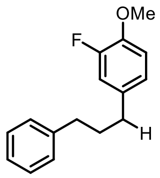

2-fluoro-1-methoxy-4-(3-phenylpropyl)benzene (4a): The reaction was conducted following the general procedure **D** in a 0.5 mmol scale. The residue was purified by column chromatography on silica gel with petroleum ether to afford the product **4a** (101.3 mg, 83% yield, rr = 45:1) as a colorless oil.^{1}H NMR (400 MHz, Chloroform-*d*) δ 7.30 - 7.26 (m, 2 H), 7.20 - 7.16 (m, 3 H), 6.92 - 6.89 (m, 1 H), 6.89 - 6.83 (m, 2 H), 3.85 (s, 3 H), 2.62 (t, *J* = 8.1 Hz, 2 H), 2.57 (t, *J* = 7.6 Hz, 2 H), 1.96 - 1.87 (m, 2 H). ^{13}C NMR (101 MHz, Chloroform-*d*) δ 152.4 (d, *J* = 244.9 Hz), 145.7 (d, *J* = 10.7 Hz), 142.2, 135.5 (d, *J* = 6.0 Hz), 128.5, 128.4, 125.9, 123.9 (d, *J* = 3.5 Hz), 116.1 (d, *J* = 17.9 Hz), 113.4 (d, *J* = 2.2 Hz), 56.4, 35.4, 34.5 (d, *J* = 1.4 Hz), 33.0. ^{19}F NMR (377 MHz, Chloroform-*d*) δ -135.8. HRMS (ESI) Calculated for $C_{15}H_{15}F_2$ ($[M+H]^+$): 233.1136, measured: 233.1142.

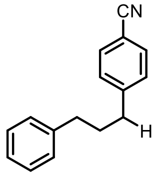

4-(3-phenylpropyl)benzonitrile (4aj): The reaction was conducted following the general procedure **D** in a 0.5 mmol scale. The residue was purified by column chromatography on silica gel with petroleum ether to afford the product **4aj** (92.8 mg, 84% yield, *rr* = 34:1) as a colorless oil.^{1}H NMR (400 MHz, Chloroform-*d*) δ 7.52 (d, *J* = 7.8 Hz, 2 H), 7.28 - 7.21 (m, 4 H), 7.17 - 7.12 (m, 3 H), 2.65 (t, *J* = 7.8 Hz, 2 H), 2.61 (t, *J* = 7.7 Hz, 2 H), 1.94 - 1.89 (m, 2 H). ^{13}C NMR (101 MHz, Chloroform-d) δ 148.1, 141.7, 132.3, 129.3, 128.5, 128.5, 126.1, 119.3, 109.7, 35.6, 35.4, 32.5. HRMS (ESI) Calculated for $C_{17}H_{20}NaO$ ($[M+Na]^+$): 244.1097, measured: 244.1102.

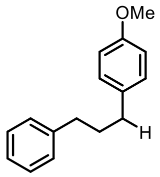

1-methoxy-4-(3-phenylpropyl)benzene (4ah): [13] The reaction was conducted following the general procedure **D** in a 0.5 mmol scale. The residue was purified by column chromatography on silica gel with petroleum ether to afford the product **4ah** (81.4 mg, 72% yield, rr > 100:1) as a colorless oil.^{1}H NMR (400 MHz, Chloroform-*d*) δ 7.31 - 7.28 (m, 2 H), 7.21 - 7.18 (m, 3 H), 7.13 - 7.10 (m, 2 H), 6.86 - 6.83 (m, 2 H), 3.79 (s, 3 H), 2.65 (t, *J* = 7.5 Hz, 2 H), 2.61 (t, *J* = 7.7 Hz, 2 H), 1.98 - 1.90 (m, 2 H).^{13}C NMR (101 MHz, Chloroform-*d*) δ 157.8, 142.5, 134.4, 129.4, 128.5, 128.4, 125.8, 113.8, 55.3, 35.5, 34.6, 33.3.

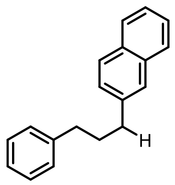

2-(3-phenylpropyl)naphthalene (4au): [13] The reaction was conducted following the general procedure **D** in a 0.5 mmol scale. The residue was purified by column chromatography on silica gel with petroleum ether to afford the product **4au** (83.6 mg, 68% yield, rr = 47:1) as a colorless oil.^{1}H NMR (400 MHz, Chloroform-*d*) δ 7.89 - 7.83 (m, 3 H), 7.70 - 7.69 (m, 1 H), 7.54 - 7.47 (m, 2 H), 7.42 - 7.36 (m, 3 H), 7.29 - 7.26 (m, 3 H), 2.89 (d, *J* = 7.7 Hz, 2 H), 2.78 (d, *J* = 7.7 Hz, 2 H), 2.17 - 2.09 (m, 2 H).^{13}C NMR (101 MHz, Chloroform-*d*) δ 142.4, 139.9, 133.7, 132.1, 128.6, 128.5, 128.0, 127.7, 127.5, 127.5, 126.5, 126.0, 125.9, 125.2, 35.7, 35.6, 33.0. HRMS (ESI) Calculated for $C_{19}H_{18}Na$ ($[M+Na]^+$): 269.1301, measured: 269.1298.

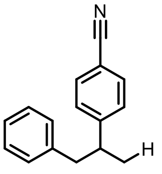

4-(1-phenylpropan-2-yl)benzonitrile (4az): The reaction was conducted following the general procedure **D** in a 0.5 mmol scale. The residue was purified by column chromatography on silica gel with petroleum ether to afford the product **4az** (81.8 mg, 74% yield, *rr* = 12:1) as a colorless oil.^{1}H NMR (400 MHz, Chloroform-*d*) δ 7.55 - 7.52 (m, 2 H), 7.24 - 7.19 (m, 4 H), 7.02 - 7.00 (m, 3 H), 3.11 - 3.02 (m, 1 H), 2.90 - 2.79 (m, 2 H), 1.27 (d, *J* = 6.9 Hz, 3 H). ^{13}C NMR (101 MHz, Chloroform-*d*) δ 152.4, 139.8, 132.3, 129.1, 128.4, 128.1, 126.3, 119.2, 110.0, 44.7, 42.3, 21.0. HRMS (ESI) Calculated for $C_{17}H_{20}NaO$ ($[M+Na]^+$): 244.1097, measured: 244.1101.

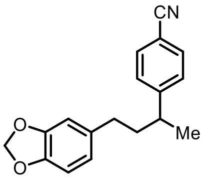

4-(4-(benzo[d][1,3]dioxol-5-yl)butan-2-yl)benzonitrile (4ba): The reaction was conducted following the general procedure **D** in a 0.5 mmol scale. The residue was purified by column chromatography on silica gel with petroleum ether to afford the product **4ba** (72.5 mg, 52% yield, *rr* > 100:1) as a colorless oil.^{1}H NMR (400 MHz, Chloroform-*d*) δ 7.61 - 7.58 (m, 2 H), 7.29 - 7.27 (m, 2 H), 6.70 (d, *J* 7.9 Hz, 1 H), 6.59 (d, *J* = 1.7 Hz, 1 H), 6.53 (dd, *J* = 7.9, 1.7 Hz, 1 H), 5.91 (s, 2 H), 2.81 - 2.72 (m, 1 H), 2.47 - 2.36 (m, 2 H), 1.90 - 1.84 (m, 2 H), 1.27 (d, *J* = 6.9 Hz, 3 H).^{13}C NMR (101 MHz, Chloroform-*d*) δ 153.0, 147.7, 145.8, 135.7, 132.4, 128.0, 121.1, 119.2, 110.0, 108.8, 108.3, 100.9, 39.8, 39.6, 33.5, 22.1. HRMS (ESI) Calculated for $C_{18}H_{17}NNaO_2$ ($[M+Na]^+$): 302.1151, measured: 302.1144.

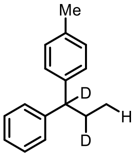

1-Methyl-4-(1-phenylpropyl-1, 2-d$_2$)benzene (3bb-D$_2$): The reaction was conducted following the general procedure **A** in a 0.5 mmol scale. The residue was purified by column chromatography on silica gel to afford the product **3bb-D$_2$** (75.3 mg, 71% yield, *rr* = 17:1) as a colorless oil. ^{1}H NMR (400 MHz, Chloroform-*d*) δ 7.28 - 7.21 (m, 4 H), 7.17 - 7.07 (m, 5 H), 2.29 (s, 3 H), 2.07 - 2.00 (m, 1 H), 0.89 (t, *J* = 7.2 Hz, 3 H). ^{13}C NMR (101 MHz, Chloroform-*d*) δ 145.5, 142.2, 135.6, 129.2, 128.5, 128.0, 127.9, 126.0, 53.0 - 52.9 (m), 28.7, 21.1, 12.9 - 12.8 (m). HRMS (ESI) Calculated for $C_{16}H_{16}D_2Na$ ($[M+Na]^+$): 235.1432, measured: 235.1434.

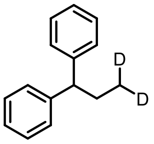

(Propane-1, 1-diyl-3, 3-d$_2$)dibenzene (3as-D$_2$'): The reaction was conducted following the general procedure **A** in a 0.5 mmol scale. The residue was purified by column chromatography on silica gel to afford the product **3as-D$_2$'** (79.2 mg, 80% yield, *rr* = 27:1, 92% D$_2$, 98% retention) as a colorless oil. ^{1}H NMR (400 MHz, Chloroform-*d*) δ 7.29 - 7.22 (m, 8 H), 7.18 - 7.14 (m, 2 H), 3.78 (t, *J* = 7.8 Hz, 1 H), 3.78 (dd, *J* = 7.8 Hz, 2 H), 0.91 - 0.84 (m, 1 H).^{13}C NMR (101 MHz, Chloroform-*d*) δ 145.3, 128.5, 128.0, 126.1, 53.3, 28.6, 12.4 (qui, *J* = 19.3 Hz). HRMS (ESI) Calculated for $C_{15}H_{14}D_2Na$ ($[M+Na]^+$): 221.1270, measured: 221.1270.

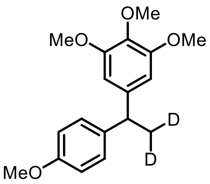

1,2,3-Trimethoxy-5-(1-(4-methoxyphenyl)ethyl-2, 2-d$_2$)benzene (3bc-D$_2$'): The reaction was conducted following the general procedure **A** in a 0.5 mmol scale. The residue was purified by column chromatography on silica gel to afford the product **3bc-D$_2$** (114.0 mg, 74% yield, *rr* > 20:1, 92% D$_2$, 97% retention) as a colorless oil. ^{1}H NMR (400 MHz, Chloroform-*d*) δ 7.14 (d, *J* = 8.6 Hz, 2 H), 6.84 (d, *J* = 8.7 Hz, 2 H), 6.42 (s, 2 H), 4.03 (d, *J* = 7.1 Hz, 1 H), 3.82 (s, 3 H), 3.81 (s, 6 H), 3.77 (s, 3 H), 1.59 - 1.55 (m, 1 H). ^{13}C NMR (101 MHz, Chloroform-*d*) δ 157.9, 153.1, 142.6, 138.3, 136.1, 128.4, 113.8, 104.6, 60.9, 56.1, 55.3, 44.1, 21.7 (qui, *J* = 20.2 Hz). HRMS (ESI) Calculated for $C_{18}H_{20}D_2NaO_4$ ([M+Na]$^+$): 327.1536, measured: 327.1537.

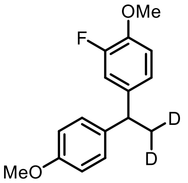

2-Fluoro-1-methoxy-4-(1-(4-methoxyphenyl)ethyl-2, 2-d$_2$)benzene (3bd-D$_2$'): The reaction was conducted following the general procedure **A** in a 0.5 mmol scale. The residue was purified by column chromatography on silica gel to afford the product **3bd-D$_2$** (104.8 mg, 80% yield, *rr* = 25:1, 92% D$_2$, 97% retention) as a colorless oil. ^{1}H NMR (400 MHz, Chloroform-*d*) δ 7.12 - 7.09 (m, 2 H), 6.93 - 6.81 (m, 5 H), 4.02 (d, *J* = 7.1 Hz, 1 H), 3.84 (s, 3 H), 3.77 (s, 3 H), 1.56 - 1.52 (m, 1 H). ^{13}C NMR (101 MHz, Chloroform-*d*) δ 158.0, 152.35 (d, *J* = 245.1 Hz), 145.74 (d, *J* = 10.8 Hz), 140.17 (d, *J* = 5.4 Hz), 138.3, 128.5, 122.98 (d, *J* = 3.4 Hz), 115.36 (d, *J* = 18.2 Hz), 113.9, 113.32 (d, *J* = 2.0 Hz), 56.4, 55.4, 43.0, 21.7 (qui, *J* = 19.2 Hz). ^{19}F NMR (377 MHz, Chloroform-*d*) δ -135.37. HRMS (ESI) Calculated for $C_{16}H_{15}D_2FNaO_2$ ([M+Na]$^+$): 285.1235, measured: 285.1230.

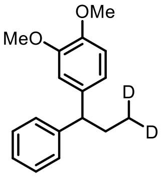

1, 2-Dimethoxy-4-(1-phenylpropyl-3, 3-d$_2$)benzene (3b-D$_2$'): The reaction was conducted following the general procedure **A** in a 0.5 mmol scale. The residue was purified by column chromatography on silica gel with to afford the product **3b-D$_2$** (96.8 mg, 75% yield, *rr* = 32:1, 93% D$_2$, 99% retention) as a colorless oil. ^{1}H NMR (400 MHz, Chloroform-*d*) δ 7.29 - 7.22 (m, 4 H), 7.18 - 7.14 (m, 1 H), 6.79 (s, 2 H), 6.73 (s, 1 H), 3.83 (s, 3 H), 3.83 (s, 3 H), 3.73 (t, *J* = 7.8 Hz, 1 H), 2.03 (dd, *J* = 7.4, 7.4 Hz, 2 H), 0.90 - 0.84 (m, 1 H). ^{13}C NMR (101 MHz, Chloroform-*d*) δ 148.8, 147.2, 145.4, 137.8, 128.4, 127.8, 126.0, 119.6, 111.3, 111.0, 55.9, 55.8, 52.8, 28.6, 12.4 (qui, *J* = 19.3 Hz). HRMS (ESI) Calculated for $C_{17}H_{18}D_2NaO_2$ ([M+Na]$^+$): 281.1487, measured: 281.1481.

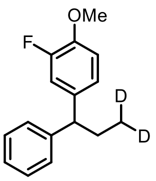

2-Fluoro-1-methoxy-4-(1-phenylpropyl-3, 3-d$_2$)benzene (3a-D$_2$'): The reaction was conducted following the general procedure **A** in a 0.5 mmol scale. The residue was purified by column chromatography on silica gel to afford the product **3a-D$_2$** (88.6 mg, 72% yield, *rr* = 18:1, 90% D$_2$, 96% retention) as a colorless oil. ^{1}H NMR (400 MHz, Chloroform-*d*) δ 7.29 - 7.25 (m, 2 H), 7.20 - 7.15 (m, 3 H), 6.97 - 6.92 (m, 2 H), 6.85 (t, *J* = 8.7 Hz, 1 H), 3.83 (s, 3 H), 3.71 (t, *J* = 7.8 Hz, 1 H), 2.00 (dd, *J* = 7.4, 7.4 Hz, 2 H), 0.86 - 0.83 (m, 1 H). ^{13}C NMR (101 MHz, Chloroform-*d*) δ 152.4 (d, *J* = 245.1 Hz), 145.8 (d, *J* = 10.8 Hz), 145.0, 138.6 (d, *J* = 5.5 Hz), 128.6, 123.5 (d, *J* = 3.4 Hz), 127.8, 126.3, 115.6 (d, *J* = 18.2 Hz), 113.3

(d, J = 2.1 Hz), 56.4, 52.3, 28.5, 12.2 (qui, J = 19.4 Hz). ^{19}F NMR (377 MHz, Chloroform-d) δ -135.36. HRMS (ESI) Calculated for $C_{16}H_{15}D_2FNaO$ ([M+Na]$^+$): 269.1287, measured: 269.1288.

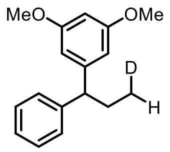

1,3-Dimethoxy-5-(1-phenylpropyl-3-d)benzene (3d-D$_1$'): The reaction was conducted following the general procedure **A** in a 0.5 mmol scale. The residue was purified by column chromatography on silica gel to afford the product **3d-D$_1$** (100.2 mg, 76% yield, *rr* > 20:1, 86% D$_1$, 91% retention) as a colorless oil. ^{1}H NMR (400 MHz, Chloroform-d) δ 7.28 - 7.21 (m, 4 H), 7.17 - 7.13 (m, 1 H), 6.41 (d, J = 2.3 Hz, 2 H), 6.28 (t, J = 2.3 Hz, 1 H), 3.73 (s, 6 H), 3.72 (t, J = 7.8 Hz, 1 H), 2.03 (dd, J = 7.3, 7.3 Hz, 2 H), 0.91 - 0.85 (m, 2 H). ^{13}C NMR (101 MHz, Chloroform-d) δ 160.8, 147.7, 144.9, 128.5, 127.9, 126.2, 106.3, 97.4, 55.3, 53.6, 28.5, 12.6 (t, J = 19.4 Hz). HRMS (ESI) Calculated for $C_{17}H_{19}DNaO_2$ ([M+Na]$^+$): 280.1420, measured: 280.1418.

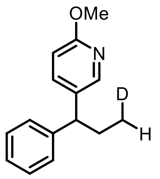

2-Methoxy-5-(1-phenylpropyl-3-d)pyridine (3c-D$_1$'): The reaction was conducted following the general procedure **A** in a 0.5 mmol scale. The residue was purified by column chromatography on silica gel to afford the product **3c-D$_1$** (68.4 mg, 65% yield, *rr* > 20:1, 85% D$_1$, 89% retention) as a colorless oil. ^{1}H NMR (400 MHz, Chloroform-d) δ 8.06 (d, J = 2.4 Hz, 1 H), 7.40 (dd, J = 8.6, 2.5 Hz, 1 H), 7.30 - 7.26 (m, 2 H), 7.22 - 7.16 (m, 3 H), 6.66 (d, J = 8.6 Hz, 1 H), 3.90 (s, 3 H), 3.73 (t, J = 7.7 Hz, 1 H), 2.11 - 1.96 (m, 2 H), 0.91 - 0.86 (m, 2 H).^{13}C NMR (101 MHz, Chloroform-d) δ 162.8, 145.8, 144.6, 138.4, 133.3, 128.6, 127.8, 126.4, 110.8, 53.4, 50.0, 28.4, 12.5 (t, J = 19.4 Hz). HRMS (ESI) Calculated for $C_{15}H_{16}DNNaO$ ([M+Na]$^+$): 251.1268, measured: 251.1265.

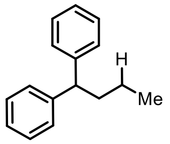

Butane-1, 1-diyldibenzene (3be)[9]: ^{1}H NMR (400 MHz, Chloroform-d) δ 7.28 - 7.22 (m, 8 H), 7.17 - 7.13 (m, 2 H), 3.90 (t, J = 7.8 Hz, 1 H), 2.05 - 1.99 (m, 2 H), 1.33 - 1.23 (m, 2 H), 0.92 (t, J = 7.3 Hz, 3 H). ^{13}C NMR (101 MHz, Chloroform-d) δ 145.4, 128.5, 128.0, 126.1, 51.2, 38.0, 21.3, 14.2.

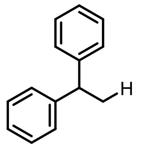

Ethane-1,1-diyldibenzene (3bf): [9] ^{1}H NMR (400 MHz, Chloroform-d) δ 7.29 - 7.15 (m, 10 H), 4.14 (q, J = 7.2 Hz, 1 H), 1.63 (d, J = 7.2 Hz, 3 H). ^{13}C NMR (101 MHz, Chloroform-d) δ 146.5, 128.5, 127.7, 126.1, 44.9, 22.0.

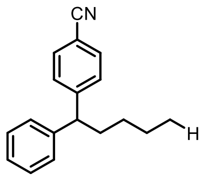

4-(1-phenylpentyl)benzonitrile (3bg): ^{1}H NMR (400 MHz, Chloroform-d) δ 7.57 - 7.55 (m, 2 H), 7.35 - 7.27 (m, 4 H), 7.22 - 7.18 (m, 3 H), 3.93 (t, J = 7.8 Hz, 1 H), 2.07 - 2.00 (m, 2 H), 1.37 - 1.31 (m, 2 H), 1.27 - 1.19 (m, 2 H), 0.86 (t, J = 7.3 Hz, 3 H). ^{13}C NMR (101 MHz,

Chloroform-*d*) δ 151.1, 143.8, 132.4, 128.81, 128.82, 127.9, 126.7, 119.2, 110.0, 51.6, 35.1, 30.2, 22.7, 14.1. HRMS (ESI) Calculated for $C_{18}H_{19}NNa$ ($[M+Na]^+$): 2721410, measured: 2721381.

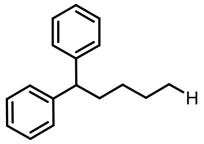

Pentane-1,1-diyldibenzene (3bh)[12]: ^1H NMR (400 MHz, Chloroform-*d*) δ 7.29 - 7.22 (m, 8 H), 7.18 - 7.14 (m, 2 H), 3.88 (t, *J* = 7.8 Hz, 1 H), 2.06 - 2.01 (m, 2 H), 1.38 - 1.29 (m, 2 H), 1.25 - 1.19 (m, 2 H), 0.86 (t, *J* = 7.2 Hz, 3 H). ^{13}C NMR (101 MHz, Chloroform-*d*) δ 145.5, 128.5, 128.0, 126.1, 51.5, 35.6, 30.4, 22.9, 14.2.

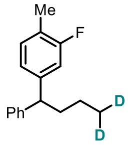

2-Fluoro-1-methyl-4-(1-phenylbutyl-4,4-d$_2$)benzene (3bi-D$_2$'): ^1H NMR (400 MHz, Chloroform-*d*) δ 7.29 - 7.24 (m, 2 H), 7.22 - 7.14 (m, 3 H), 7.08 - 7.03 (m, 1 H), 6.91 - 6.86 (m, 2 H), 3.85 (t, *J* = 7.8 Hz, 1 H), 2.20 (d, *J* = 1.9 Hz, 3 H), 2.00 - 1.95 (m, 2 H), 1.28 - 1.22 (m, 2 H), 0.92 - 0.85 (m, 1 H). ^{13}C NMR (151 MHz, Chloroform-d) δ 161.4 (d, *J* = 244.3 Hz), 145.3 (d, *J* = 7.0 Hz), 145.1, 131.3 (d, *J* = 5.4 Hz), 128.6, 127.9, 126.3, 123.4 (d, *J* = 2.9 Hz), 122.3 (d, *J* = 17.3 Hz), 114.4 (d, *J* = 22.2 Hz), 50.6, 37.9, 21.0, 14.3 (d, *J* = 3.6 Hz), 13.6 (qui, *J* = 19.1 Hz). ^{19}F NMR (376 MHz, Chloroform-*d*) δ -117.64. HRMS (ESI) Calculated for $C_{17}H_{17}D_2Na$ ($[M+Na]^+$): 267.1494, measured: 267.1487.

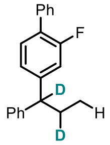

2-Fluoro-4-(1-phenylpropyl-1,2-d$_2$)-1,1'-biphenyl (3bj-D$_2$): ^1H NMR (400 MHz, Chloroform-*d*) δ 7.52 - 7.49 (m, 2 H), 7.42 – 7.38 (m, 2 H), 7.34 – 7.24 (m, 6 H), 7.21 – 7.17 (m, 1 H), 7.09 – 7.01 (m, 2 H), 2.10 – 2.04 (m, 1 H), 0.92 (t, *J* = 7.3 Hz, 3 H). ^{19}F NMR (376 MHz, Chloroform-*d*) δ -118.06. ^{13}C NMR (151 MHz, Chloroform-*d*) δ 159.9 (d, *J* = 247.7 Hz), 147.0 (d, *J* = 7.1 Hz), 144.5, 135.9, 130.6 (d, *J* = 4.1 Hz), 129.1 (d, *J* = 2.9 Hz), 128.7, 128.5, 128.01, 127.98, 127.6, 126.7 (d, *J* = 13.5 Hz), 126.5, 124.0 (d, *J* = 3.3 Hz), 115.5 (d, *J* = 22.9 Hz), 52.9 – 52.0 (m), 28.6 – 28.0 (m), 12.9 – 12.0 (m). ^{19}F NMR (376 MHz, Chloroform-*d*) δ -118.06. HRMS (ESI) Calculated for $C_{21}H_{17}D_2FNa$ ($[M+Na]^+$): 315.1494, measured: 315.1490.

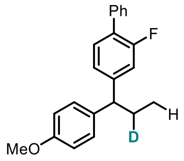

2-Fluoro-4-(1-(4-methoxyphenyl)propyl-2-d)-1,1'-biphenyl (3bk-D$_1$'): ^1H NMR (400 MHz, Chloroform-*d*) δ 7.52 - 7.49 (m, 2 H), 7.43 - 7.38 (m, 2 H), 7.34 - 7.30 (m, 2 H), 7.18 - 7.15 (m, 2 H), 7.07 - 6.99 (m, 2 H), 6.86 - 6.83 (m, 2 H), 3.78 (q, *J* = 7.3 Hz, 1 H), 3.77 (s, 3 H), 2.11 - 1.99 (m, 1 H), 0.92 (t, *J* = 7.2 Hz, 3 H). ^{13}C NMR (151 MHz, Chloroform-*d*) δ 159.8 (d, *J* = 247.6 Hz), 158.2, 147.5 (d, *J* = 7.0 Hz), 136.6, 135.9, 130.6 (d, *J* = 4.1 Hz), 129.1 (d, *J* = 2.8 Hz), 128.9, 128.5, 127.5, 126.6 (d, *J* = 13.6 Hz), 123.9 (d, *J* = 3.2 Hz), 115.4 (d, *J* = 22.9 Hz), 114.0, 55.3, 52.0, 28.7, 12.9. ^{19}F NMR (376 MHz, Chloroform-*d*) δ -118.12. HRMS (ESI) Calculated for $C_{22}H_{20}DFNa$ ($[M+Na]^+$): 344.1537, measured: 344.1535.

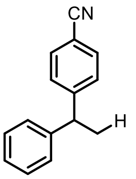

4-(1-phenylethyl)benzonitrile (3bl): [14] ^{1}H NMR (400 MHz, Chloroform-*d*) δ 7.57 - 7.54 (m, 2 H), 7.33 - 7.28 (m, 4 H), 7.24 - 7.16 (m, 3 H), 4.19 (q, *J* = 7.2 Hz, 1 H), 1.64 (d, *J* = 7.2 Hz, 3 H). ^{13}C NMR (101 MHz, Chloroform-*d*) δ 152.0, 144.8, 132.4, 128.8, 128.5, 127.7, 126.7, 119.1, 110.1, 45.0, 21.5.

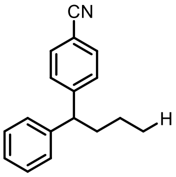

4-(1-phenylbutyl)benzonitrile (3bm): ^{1}H NMR (400 MHz, Chloroform-*d*) δ 7.57 - 7.54 (m, 2 H), 7.35 - 7.27 (m, 4 H), 7.22 - 7.18 (m, 3 H), 3.96 (t, *J* = 7.8 Hz, 1 H), 2.07 - 1.95 (m, 2 H), 1.32 - 1.22 (m, 2 H), 0.93 (t, *J* = 7.3 Hz, 3 H). ^{13}C NMR (101 MHz, Chloroform-*d*) δ 151.1, 143.7, 132.4, 128.80,128.81, 127.9, 126.7, 119.2, 110.0, 51.3, 37.6, 21.1, 14.1. HRMS (ESI) Calculated for $C_{17}H_{17}NNa$ ([M+Na]$^{+}$): 258.1253, measured: 258.1239.

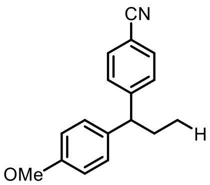

4-(1-(4-methoxyphenyl)propyl)benzonitrile (3bn): ^{1}H NMR (400 MHz, Chloroform-*d*) δ 7.57 - 7.54 (m, 2 H), 7.32 - 7.29 (m, 2 H), 7.12 - 7.08 (m, 2 H), 6.85 - 6.82 (m, 2 H), 3.79 (t, *J* = 8.3 Hz, 1 H), 3.77 (s, 3 H), 2.09 - 2.18 (m, 2 H), 0.89 (t, *J* = 7.3 Hz, 3 H). ^{13}C NMR (101 MHz, Chloroform-*d*) δ 158.4, 151.4, 135.7, 132.4, 128.9, 128.7, 119.2, 114.2, 109.9, 55.4, 52.6, 28.5, 12.7. HRMS (ESI) Calculated for $C_{17}H_{18}NO$ ([M+H]$^{+}$): 252.1383, measured: 252.1390.

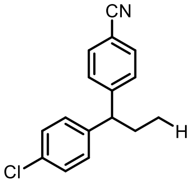

4-(1-(4-chlorophenyl)propyl)benzonitrile (3bo): ^{1}H NMR (400 MHz, Chloroform-*d*) δ 7.57 - 7.54 (m, 2 H), 7.31 - 7.29 (m, 2 H), 7.27 - 7.24 (m, 2 H), 7.14 - 7.10 (m, 2 H), 3.82 (t, *J* = 7.8 Hz, 1 H), 2.08 - 2.01 (m, 2 H), 0.89 (t, *J* = 7.3 Hz, 3 H).^{13}C NMR (101 MHz, Chloroform-*d*) δ 150.3, 142.0, 132.5, 132.4, 129.3, 128.9, 128.7, 119.0, 110.3, 52.6, 28.2, 12.6. HRMS (ESI) Calculated for $C_{16}H_{14}ClNNa$ ([M+Na]$^{+}$): 278.0707, measured: 278.0708.

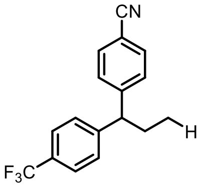

4-(1-(4-(trifluoromethyl)phenyl)propyl)benzonitrile (3bp): ^{1}H NMR (400 MHz, Chloroform-*d*) δ 7.60 - 7.55 (m, 4 H), 7.34 - 7.30 (m, 4 H), 3.92 (t, *J* = 7.8 Hz, 1 H), 2.14 - 2.06 (m, 2 H), 0.91 (t, *J* = 7.3 Hz, 3 H). ^{13}C NMR (101 MHz, Chloroform-*d*) δ 149.7, 147.60 (d, *J* = 1.1 Hz), 132.6, 129.1 (q, J = 32.5 Hz), 128.8, 128.3, 125.8 (q, *J* = 3.7 Hz), 124.2 (q, *J* = 271.9 Hz), 118.9, 110.5, 53.1, 28.2, 12.6. ^{19}F NMR (377 MHz, Chloroform-*d*) δ -62.4. HRMS (ESI) Calculated for $C_{17}H_{14}F_3NNa$ ([M+Na]$^{+}$): 312.0976, measured: 312.0979.

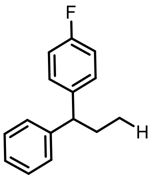

1-fluoro-4-(1-phenylpropyl)benzene (3bq): [9] ^{1}H NMR (400 MHz, Chloroform-*d*) δ 7.30 - 7.25 (m, 2 H), 7.22 - 7.15 (m, 5 H), 6.98 - 6.93 (m, 2 H), 3.77 (t, *J* = 7.8 Hz, 1 H), 2.06 - 2.02 (m, 2 H), 0.89 (t, *J* = 7.3 Hz, 3 H). ^{13}C NMR (101 MHz, Chloroform-*d*) δ 161.4 (d, *J* = 243.9 Hz), 145.1, 141.0 (d, *J* = 3.2 Hz), 129.4 (d, *J* = 7.8 Hz), 128.6, 127.9, 126.3, 115.2 (d, *J* = 21.0 Hz), 52.6, 28.8, 12.9.^{19}F NMR (377 MHz, Chloroform-*d*) δ -117.5.

Computational Methods: All density functional theory calculations were carried out with the Gaussian 09 programs.[15] Density functional B3-LYP[16-17] with a standard 6-31G(d) basis set (LANL2DZ basis set for Ni) was used for geometry optimizations. Harmonic frequency calculations were performed for all stationary points to confirm them as local minima or transition structures and to derive the thermochemical corrections for the enthalpies and free energies. M06 functional[18-19] was used to calculate the single point energies and provide highly accurate energy information. The solvent effects were considered by single point calculations on the gas-phase stationary points with a continuum solvation model SMD.[20] The larger basis set 6-311+G(d,p) (LANL2DZ basis set for Ni) was used in the solvation single point calculations. The energies given in this report are the M06 calculated Gibbs free energies and enthalpies in DMA solvent. Two explicit DMA molecules were added to each Li atom to make the Li four-coordinated. Outer-shell solvent molecules were treated using the implicit solvation model (SMD).

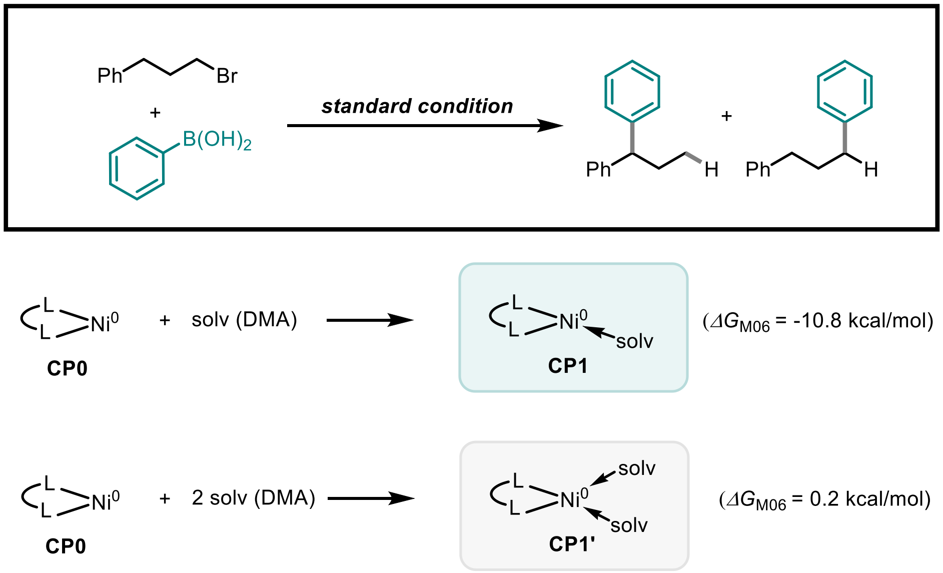

Supplementary Figure 55. Corresponding calculation of thermodynamic stability comparison between active catalyst CP1 and CP1'.

Usually, the energy profile starts with the most stable catalyst species added/generated in the reaction system. In the experimental report, in the presence of BC ligand and reductive Et_3SiH species, the catalyst precursor NiI_2 would generate $BCNi^0$ species by ligand exchange and reduction. As shown in Scheme S4, in the presence of solvent molecules (*N*, *N*-dimethylacetamide, DMA), solvent molecular

coordination occurs to generate three or four-coordinated nickel species **CP1** or **CP1'**. The free energy of three-coordinated $Ni^0(BC)(DMA)$ **CP1** is 10.8 kcal/mol lower than four-coordinated $Ni^0(BC)(DMA)_2$ **CP1'**. Therefore, **CP1** is considered to be the active catalyst species and set to relative zero point in the calculated reaction energy profile.

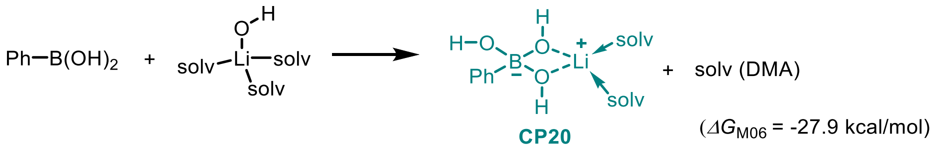

Supplementary Figure 56. Calculation of the combine process of Ph-B$(OH)_2$ and LiOH•3DMA to generate CP20.

In our experimental part, LiOH acts as an additive to active benzene boric acid. Considering charge neutralization and coordination number, we calculated the combine process of PhB$(OH)_2$ and LiOH·3DMA to generate **CP20** and find it's 27.9 kcal/mol exoergicity, which indicates the existence and stability of lithium phenyl boronate compounds **CP20**.

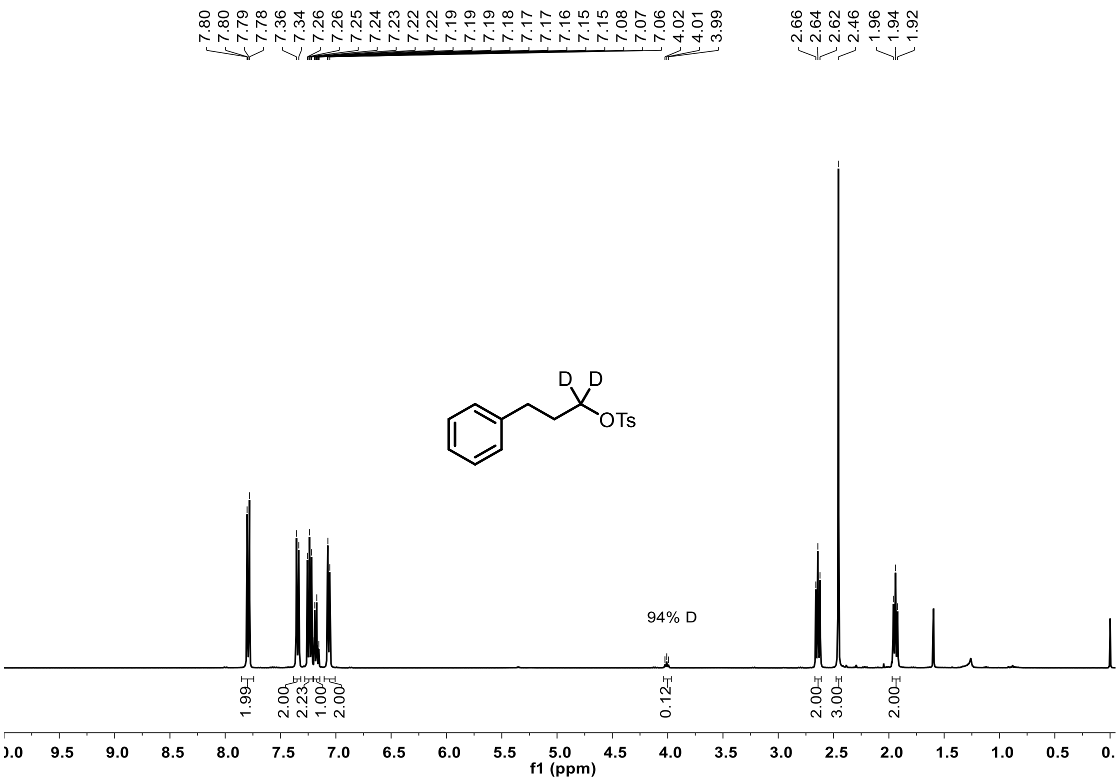

Supplementary Figure 57. ^{1}H NMR spectra for 1a-D$_2$'

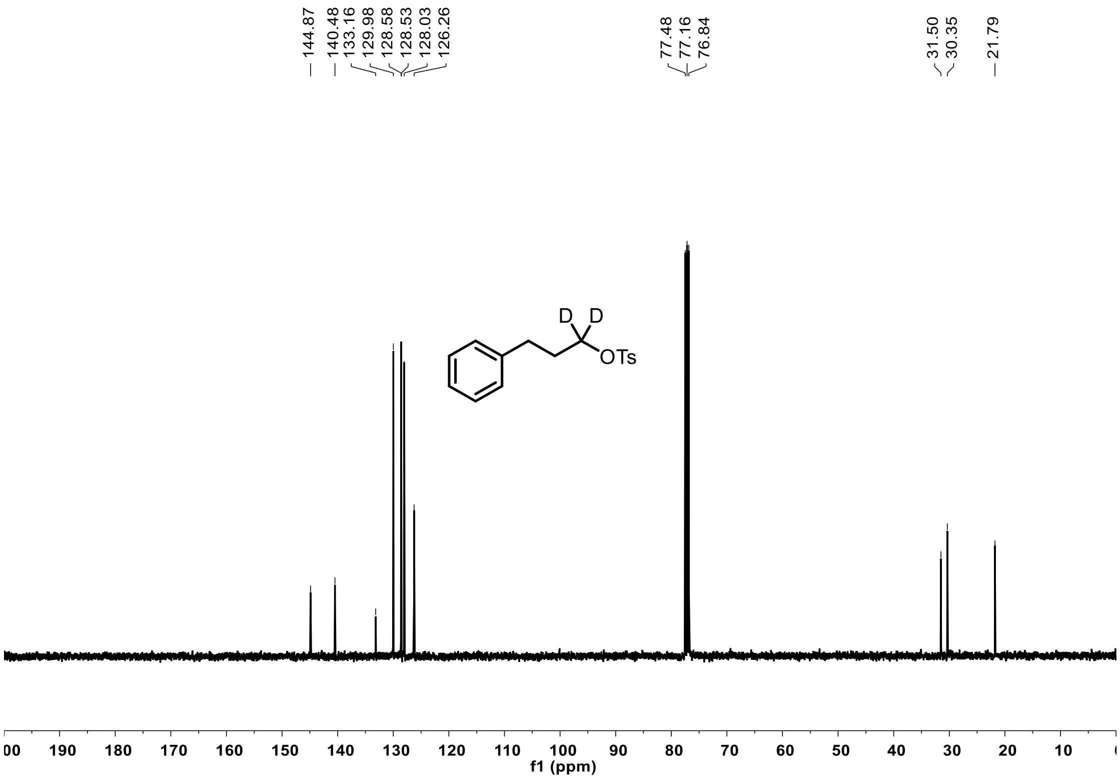

Supplementary Figure 58. ^{13}C NMR spectra for 1a-D$_2$'

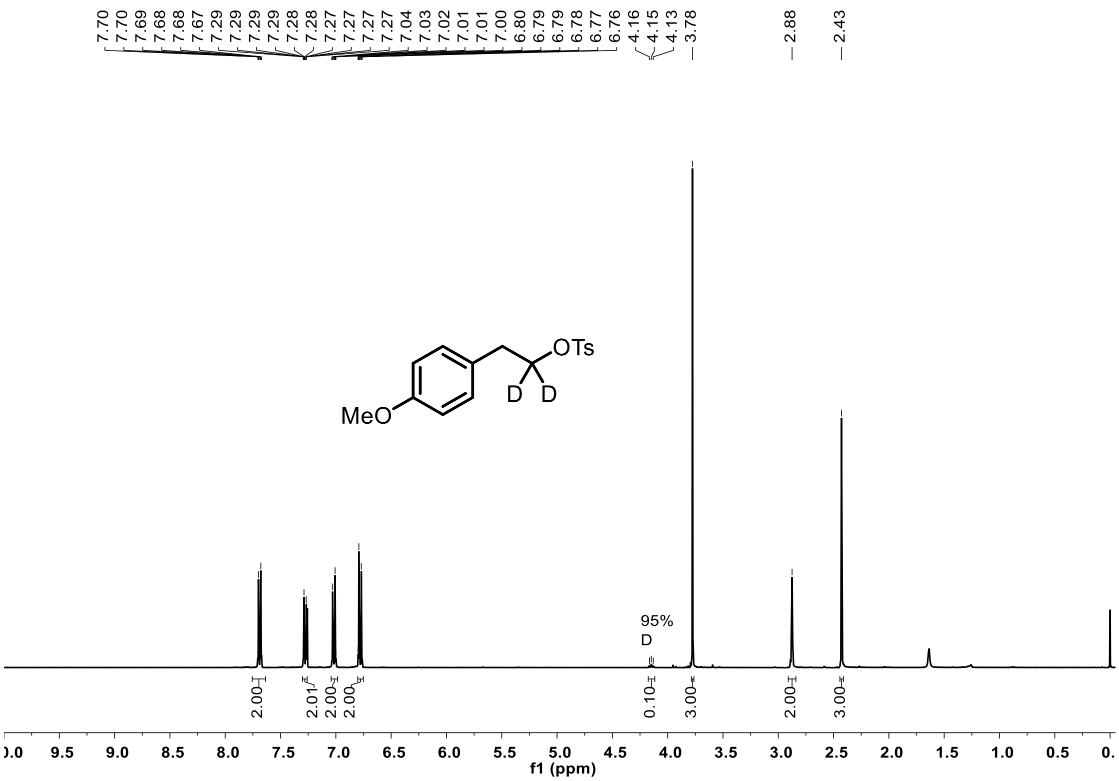

Supplementary Figure 59. ^{1}H NMR spectra of 1o-D$_2$'

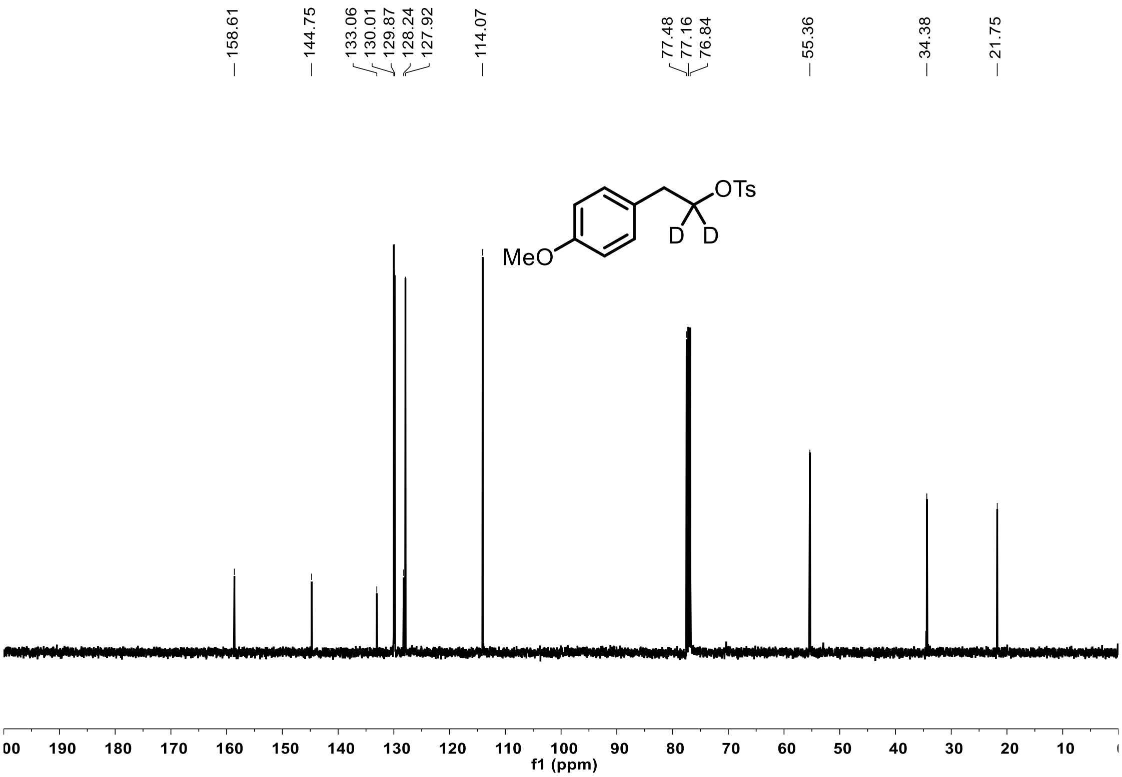

Supplementary Figure 60. ^{13}C NMR spectra of 1o-D$_2$'

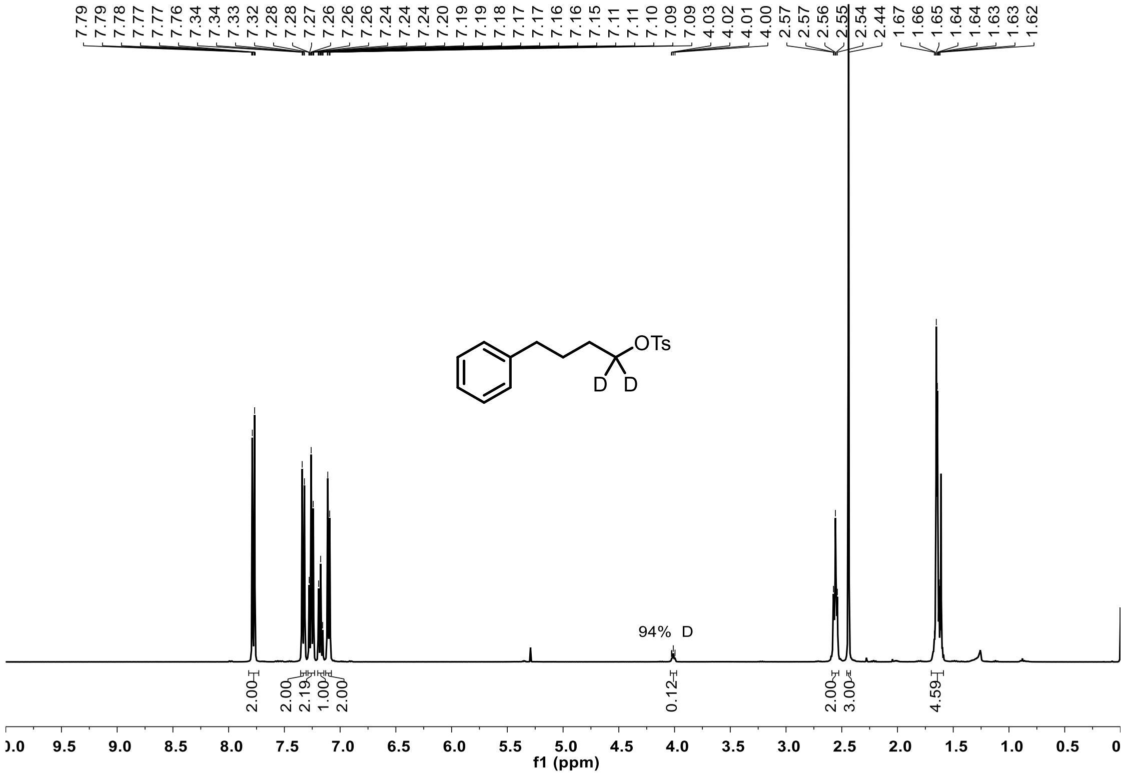

Supplementary Figure 61. ^{1}H NMR spectra of 1p-D$_2$‘

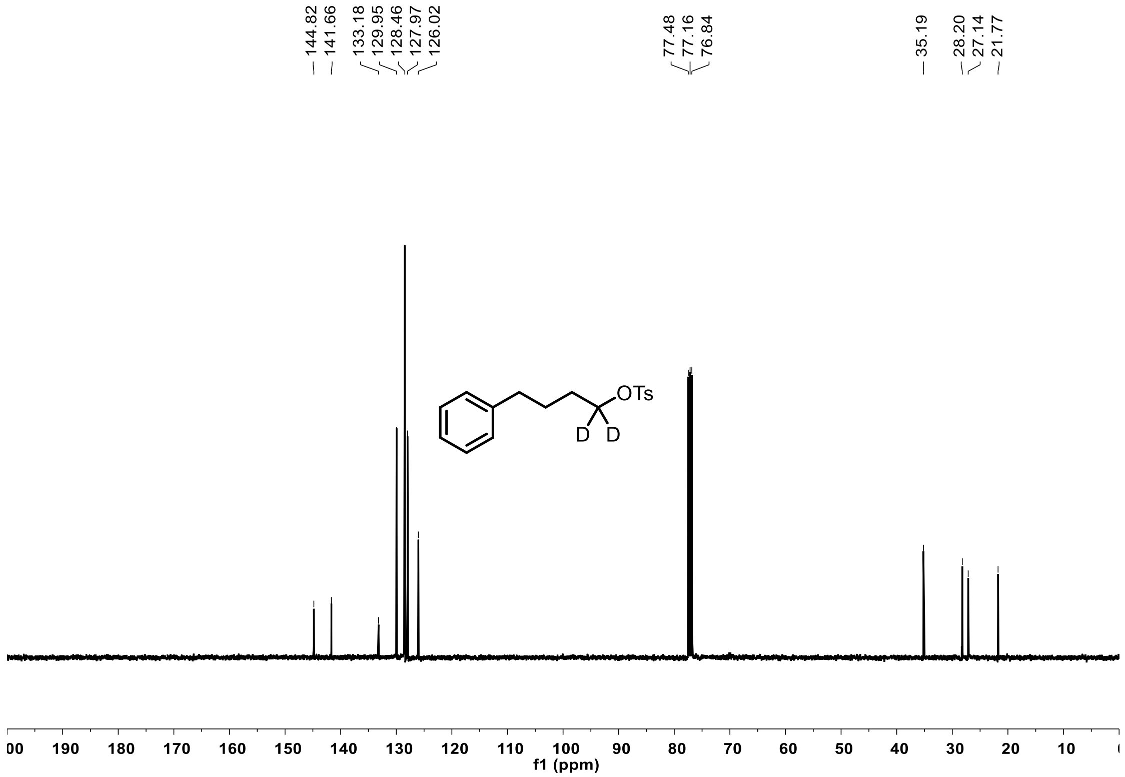

Supplementary Figure 62. ^{13}C NMR spectra 1p-D$_2$‘

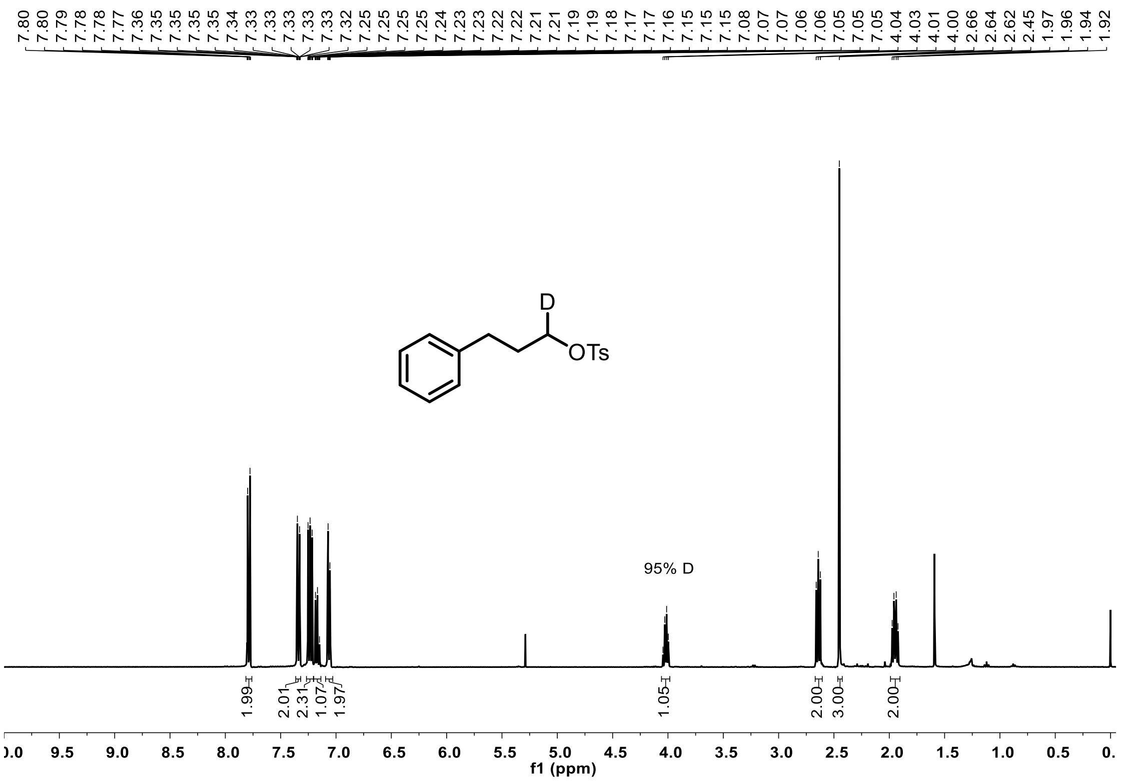

Supplementary Figure 63. ^{1}H NMR spectra for 1a-D_1

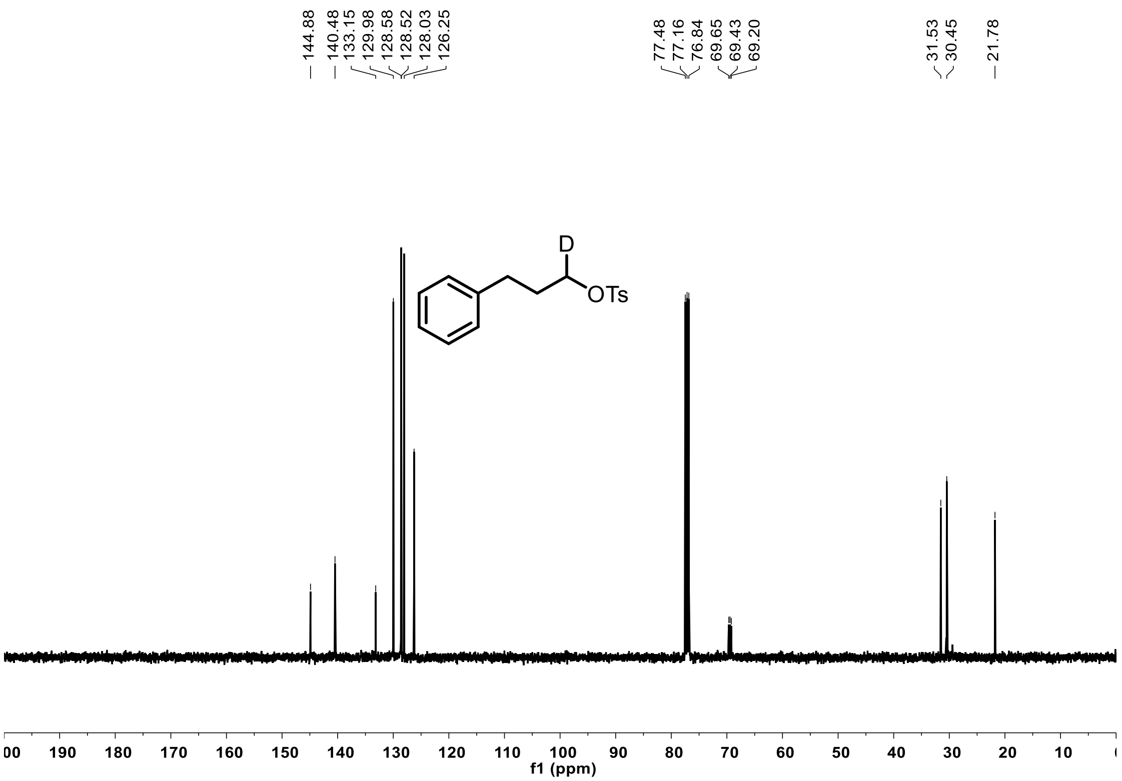

Supplementary Figure 64. ^{13}C NMR spectra for 1a-D_1

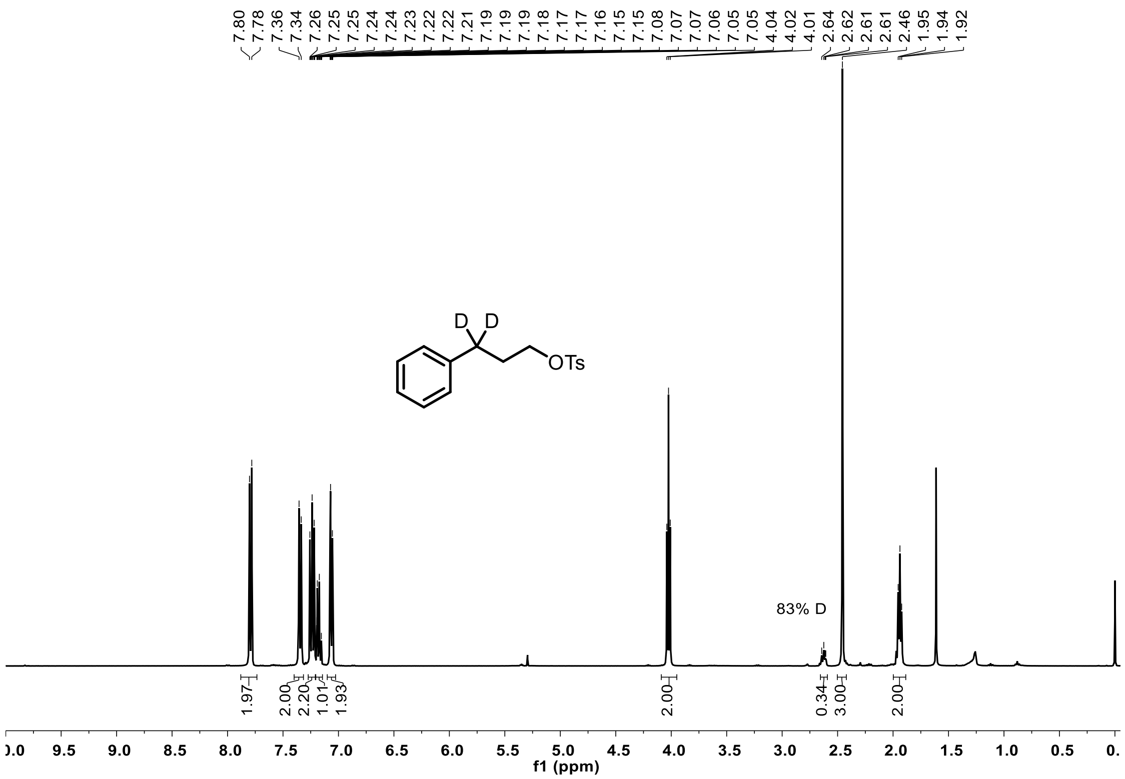

Supplementary Figure 65. ^{1}H NMR spectra for 1a-D_2

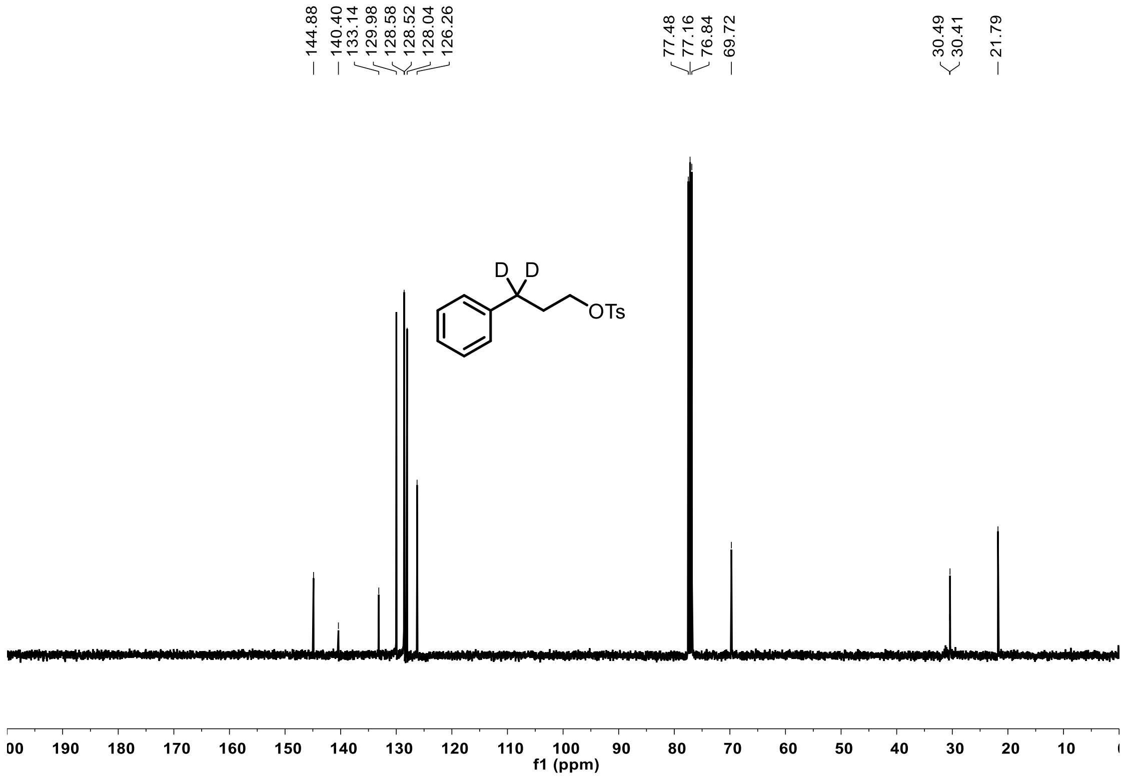

Supplementary Figure 66. ^{13}C NMR spectra for 1a-D_2

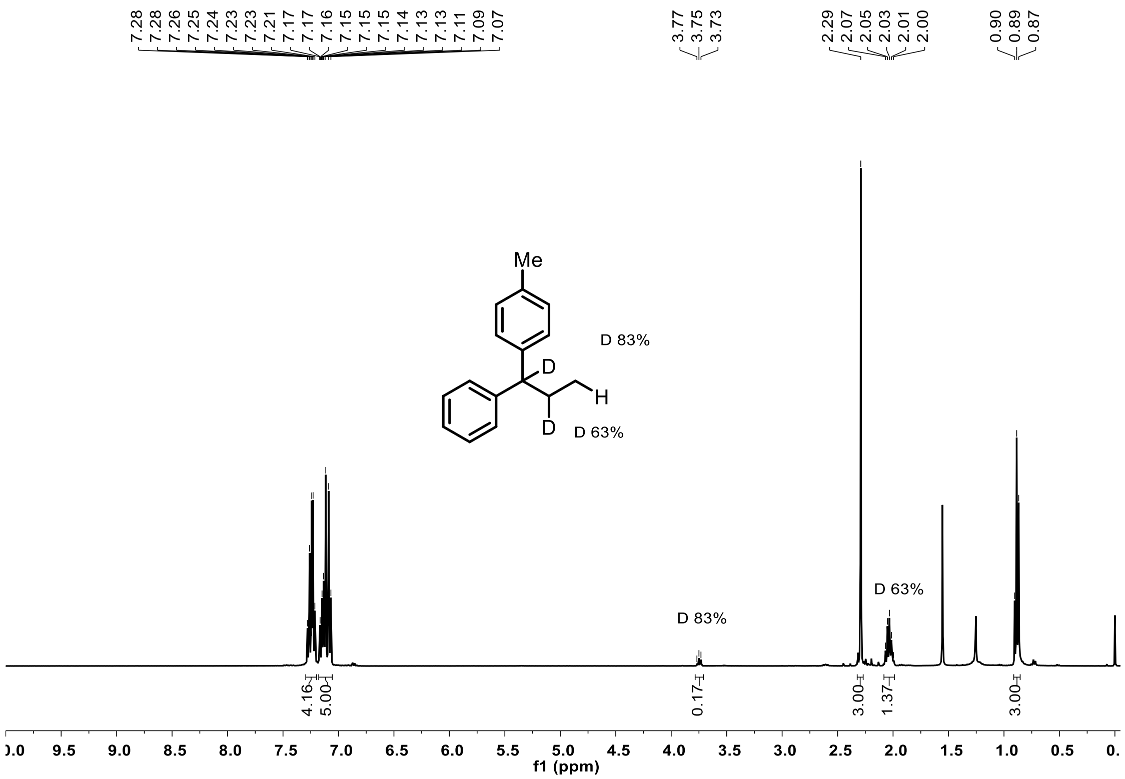

Supplementary Figure 67. ^{1}H NMR spectra for 3bb-D_2'

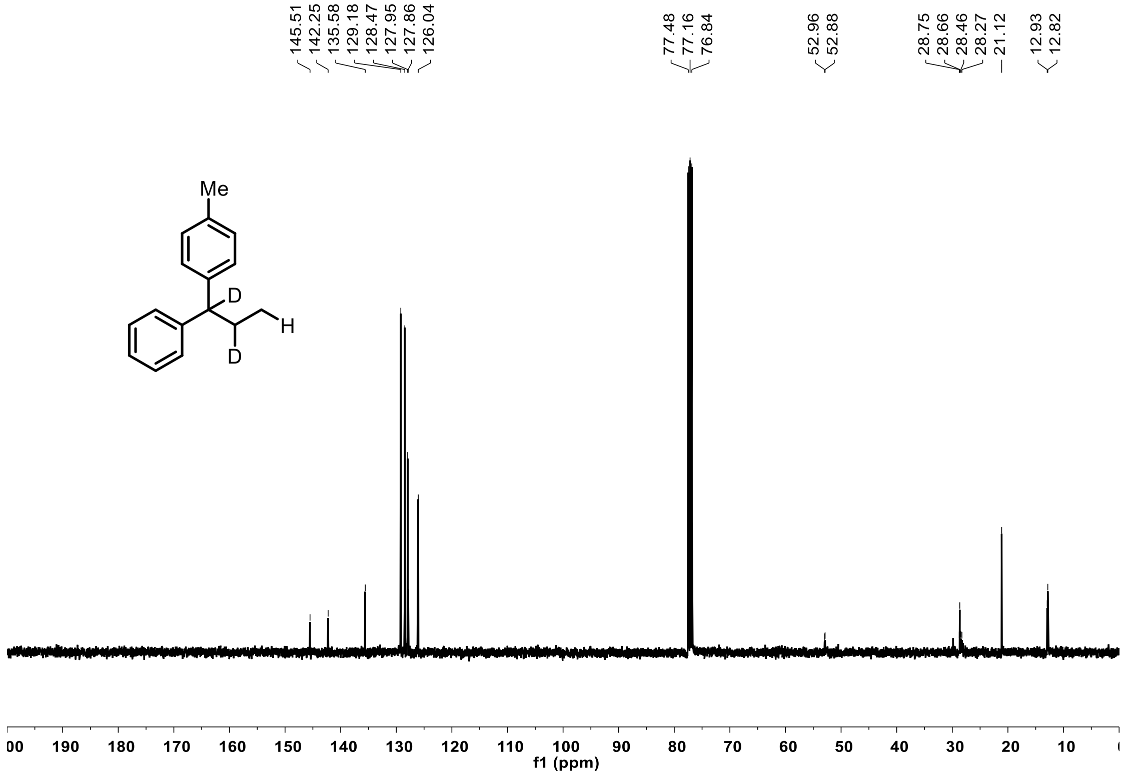

Supplementary Figure 68. ^{13}C NMR spectra for 3bb-D_2'

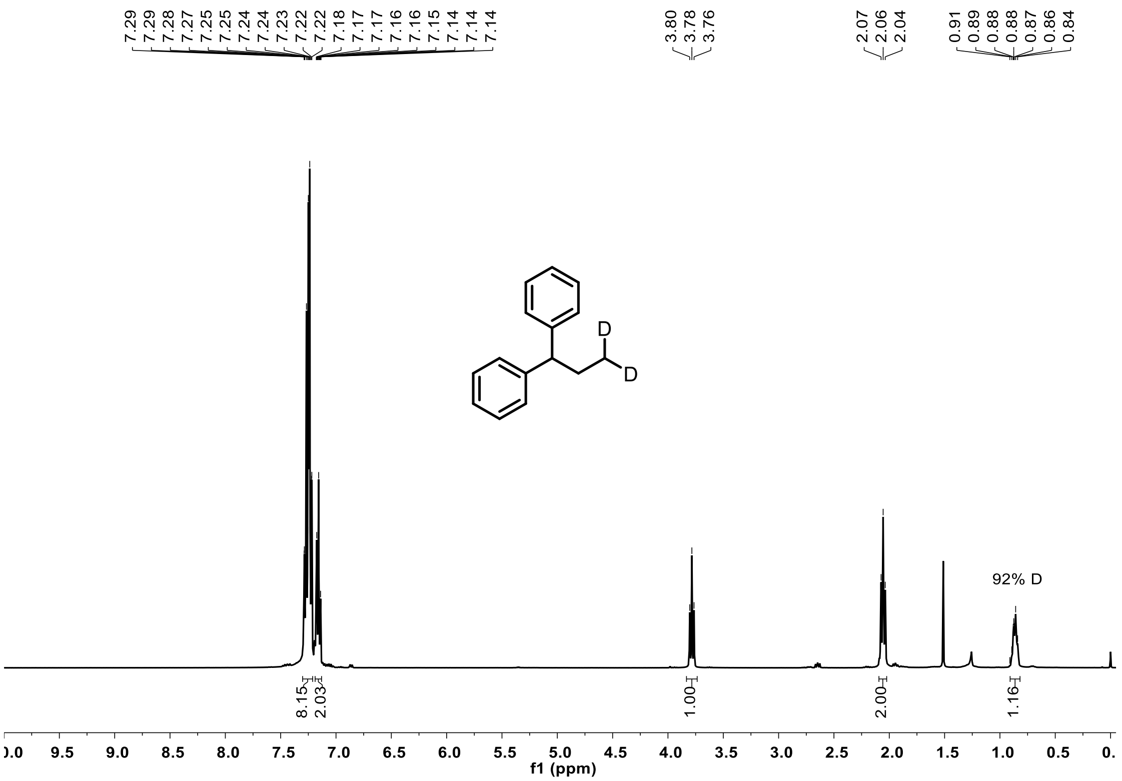

Supplementary Figure 69. ^{1}H NMR spectra for 3as-D_2’

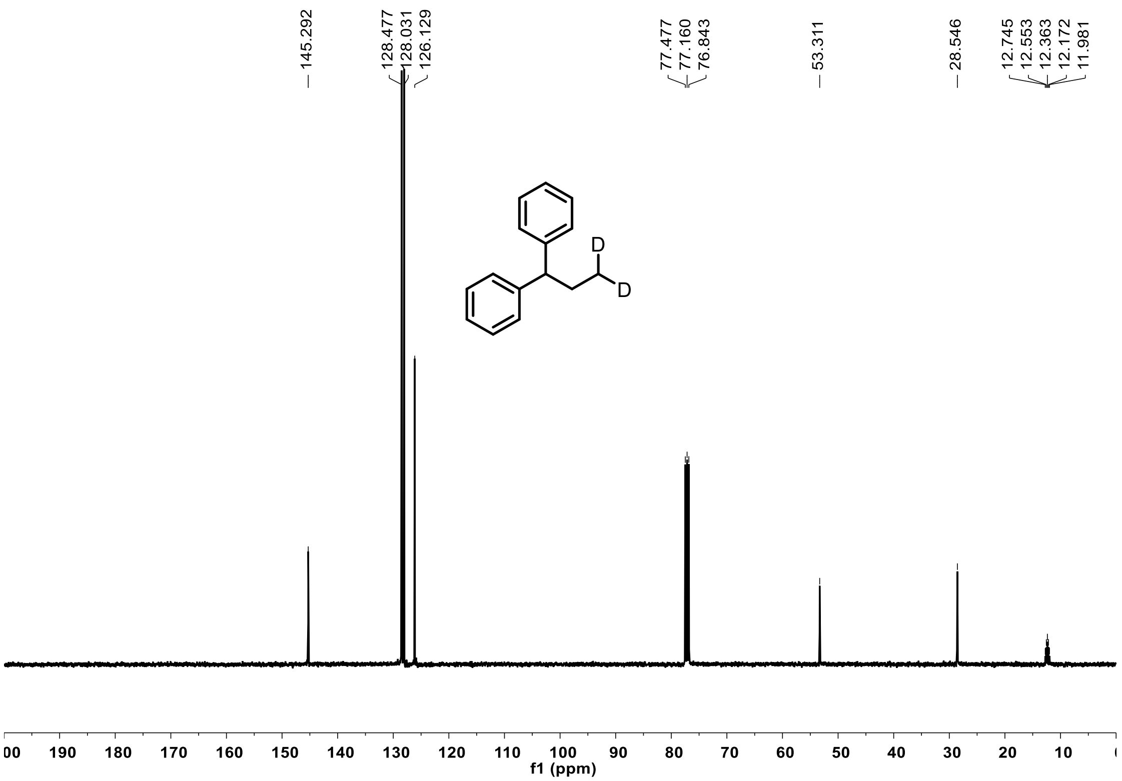

Supplementary Figure 70. ^{1}H NMR spectra for 3as-D_2’

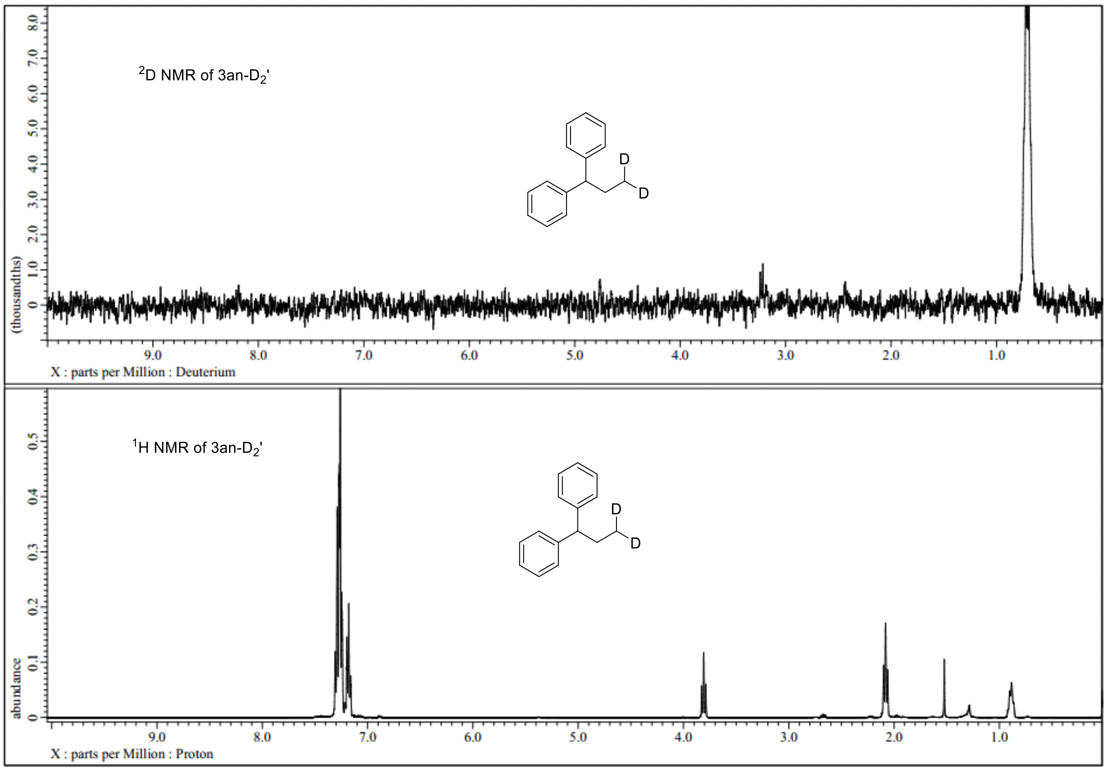

Supplementary Figure 71. ²D NMR spectra for 3as-D₂’

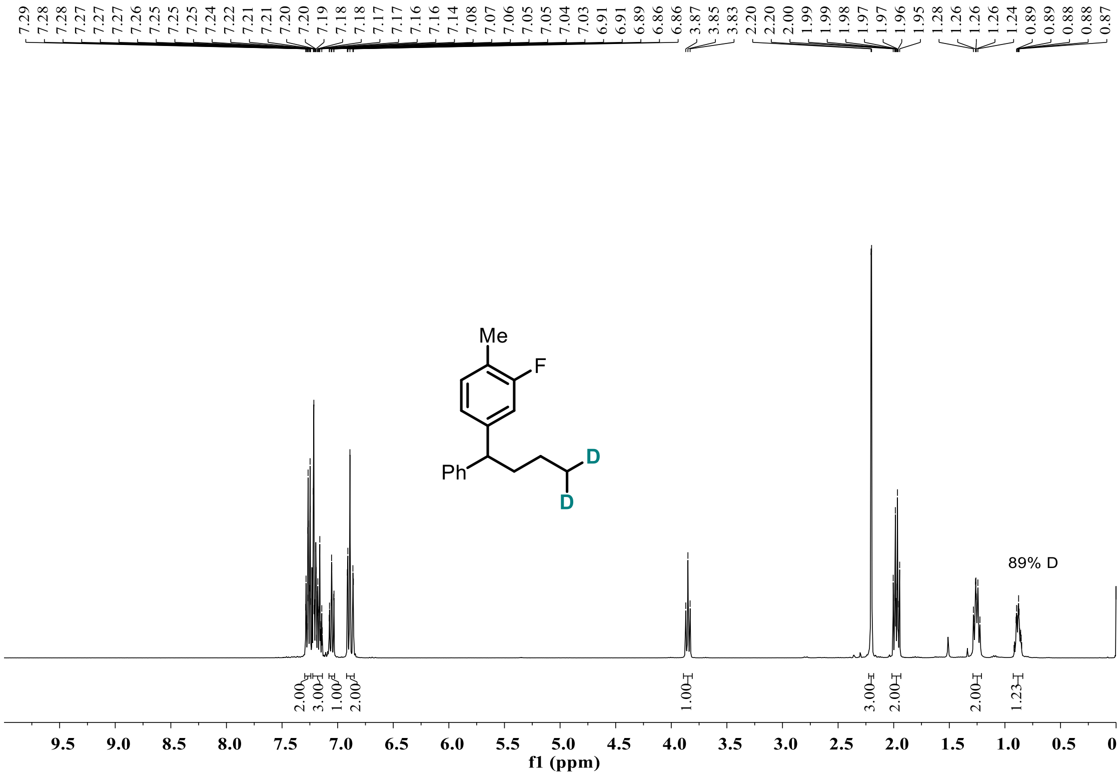

Supplementary Figure 72. ¹H NMR spectra for 3bi-D₂’

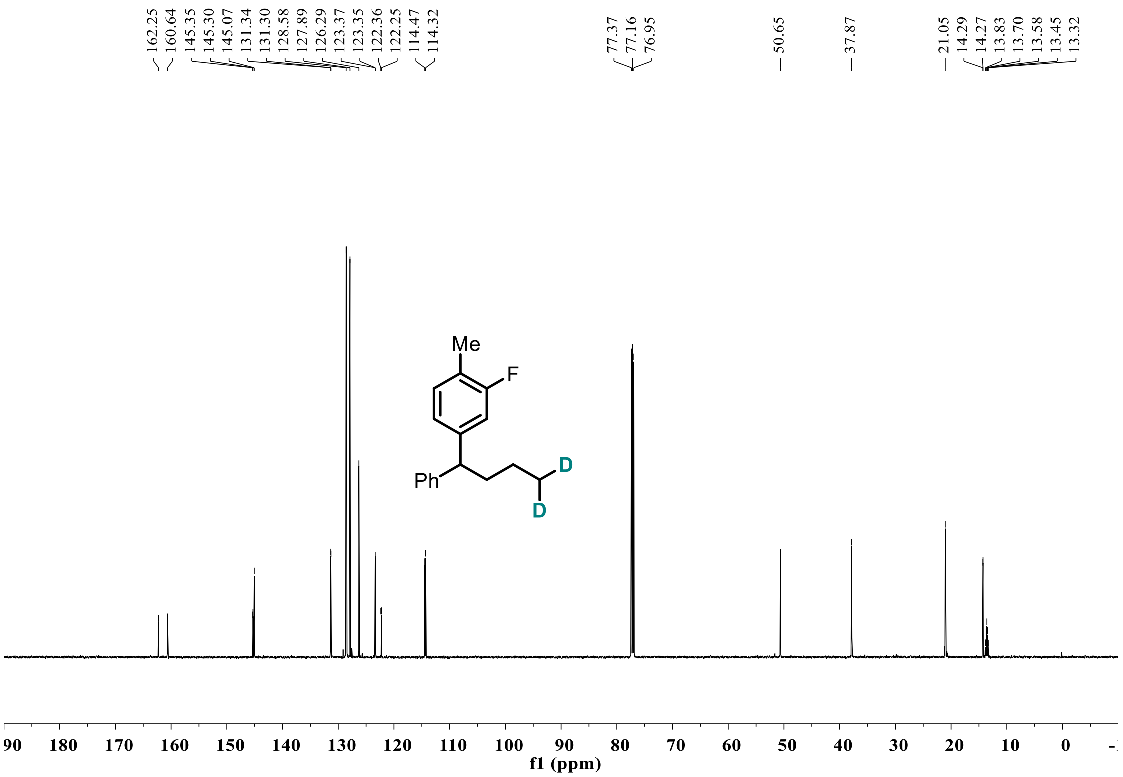

Supplementary Figure 73. ^{13}C NMR spectra for 3bi-D$_2$’

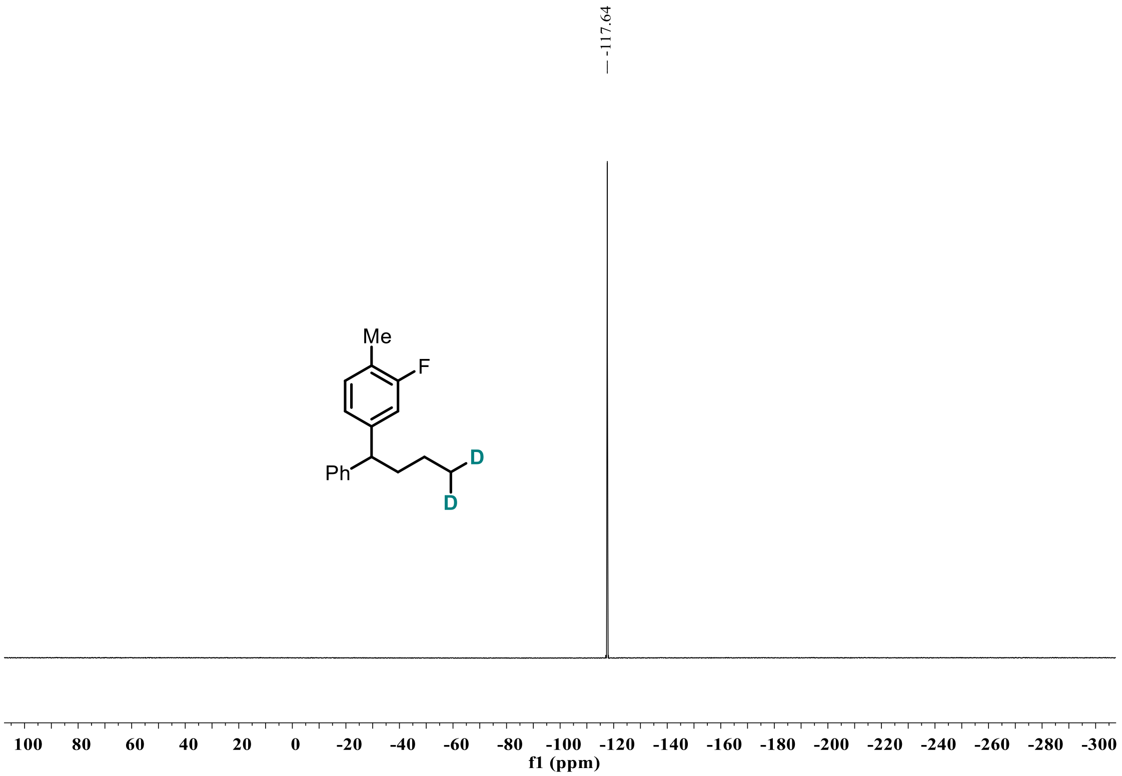

Supplementary Figure 74. ^{19}F NMR spectra

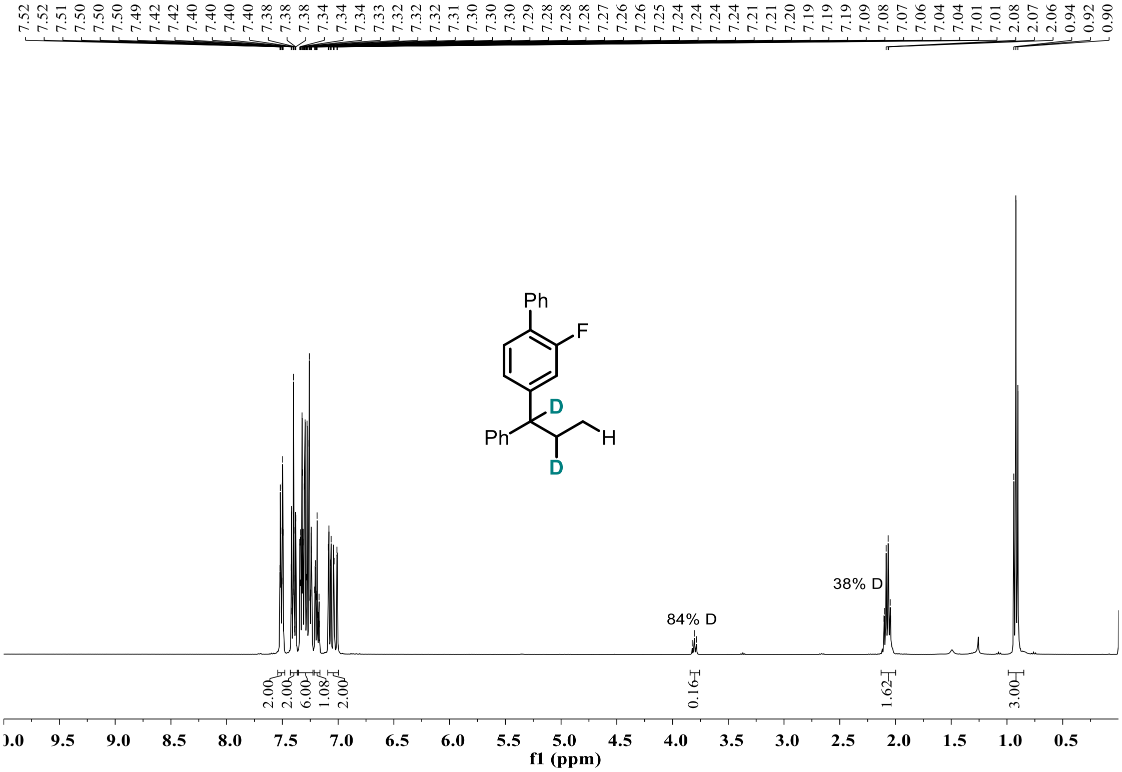

Supplementary Figure 75. ^{1}H NMR spectra for 3bj-D_2

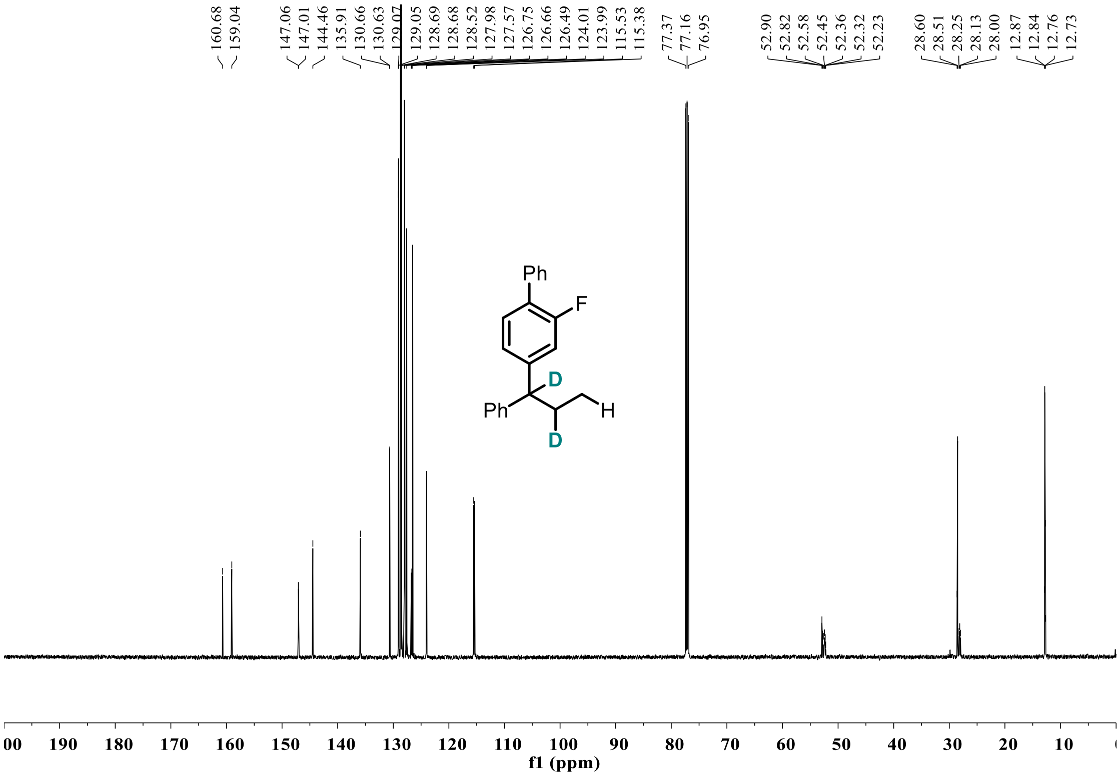

Supplementary Figure 76. ^{13}C NMR spectra for 3bj-D_2

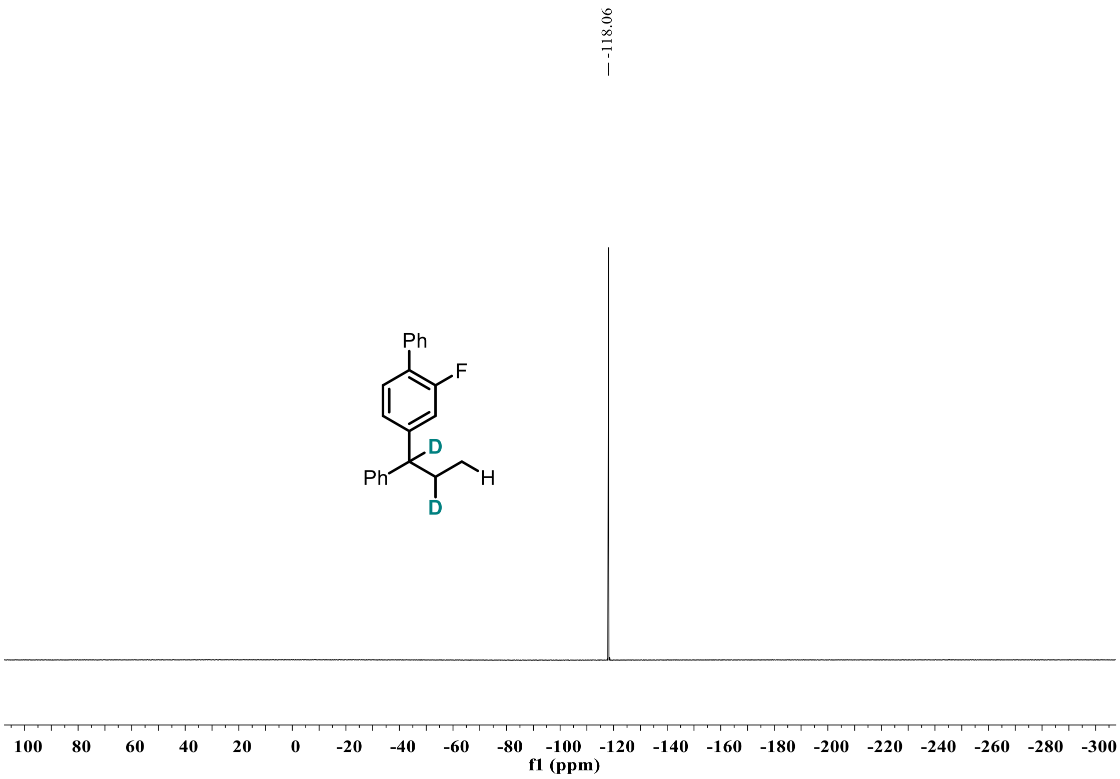

Supplementary Figure 77. ^{19}F NMR spectra for 3bj-D_2

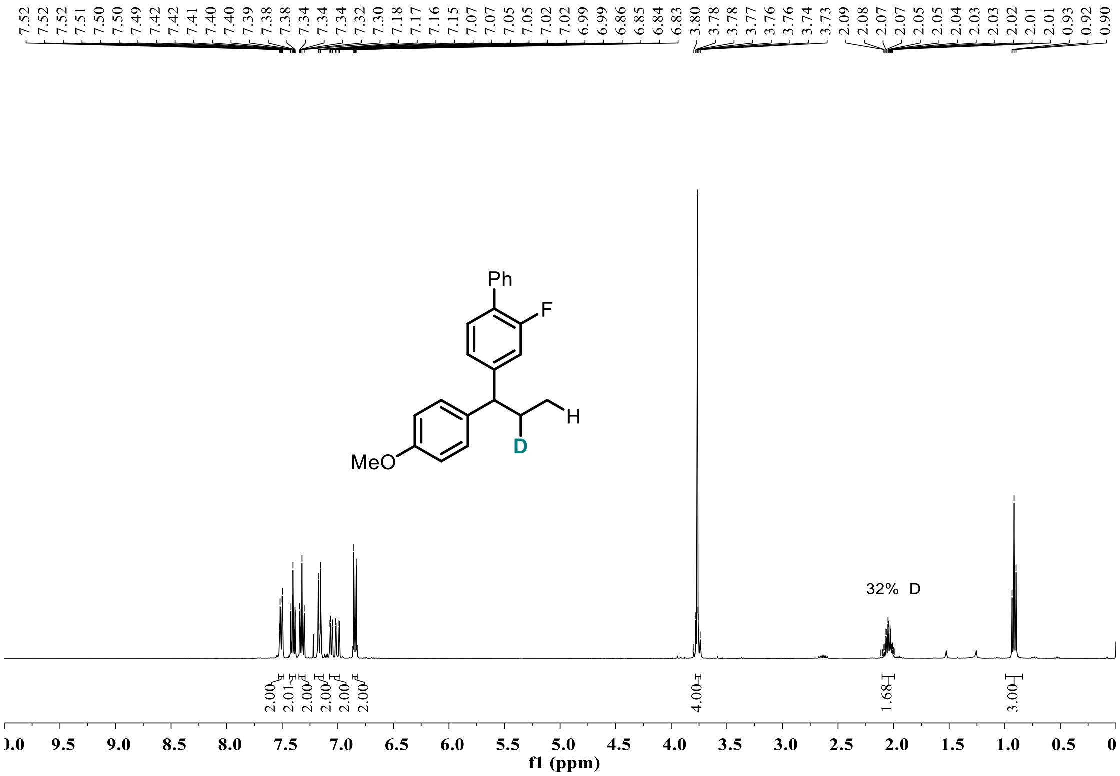

Supplementary Figure 78. ^{1}H NMR spectra for 3bk-D_2'

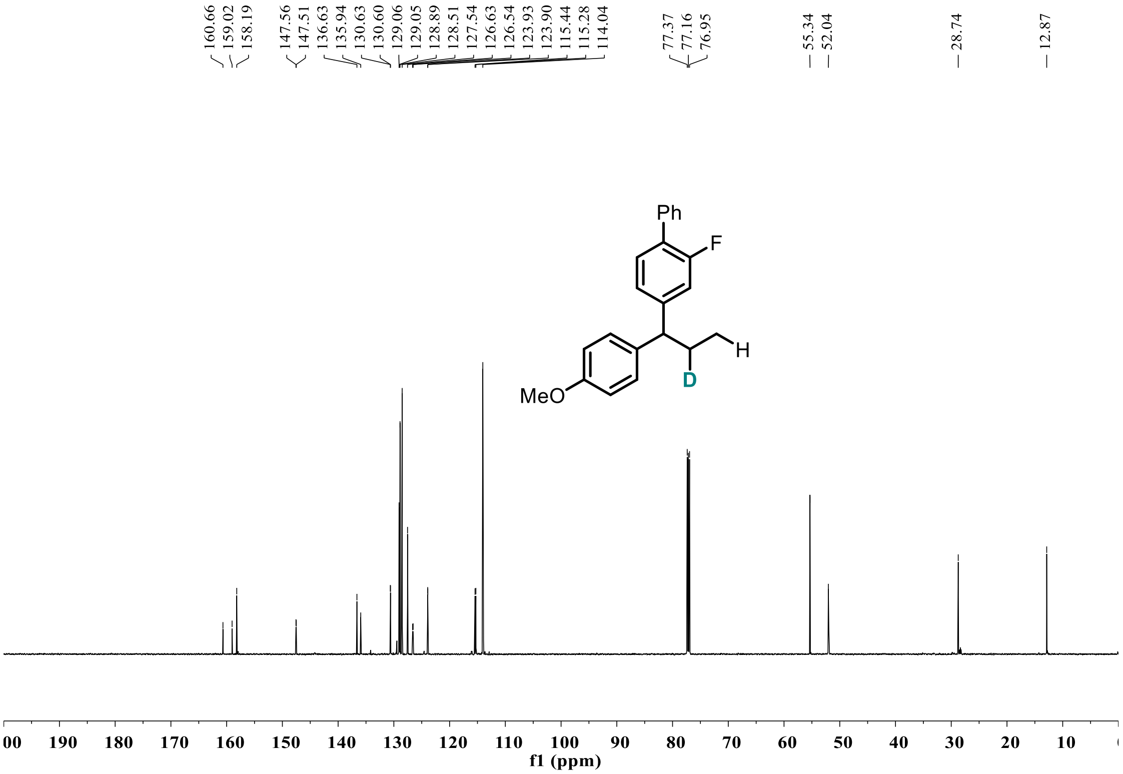

Supplementary Figure 79. ^{13}C NMR spectra 3bk-D_2’

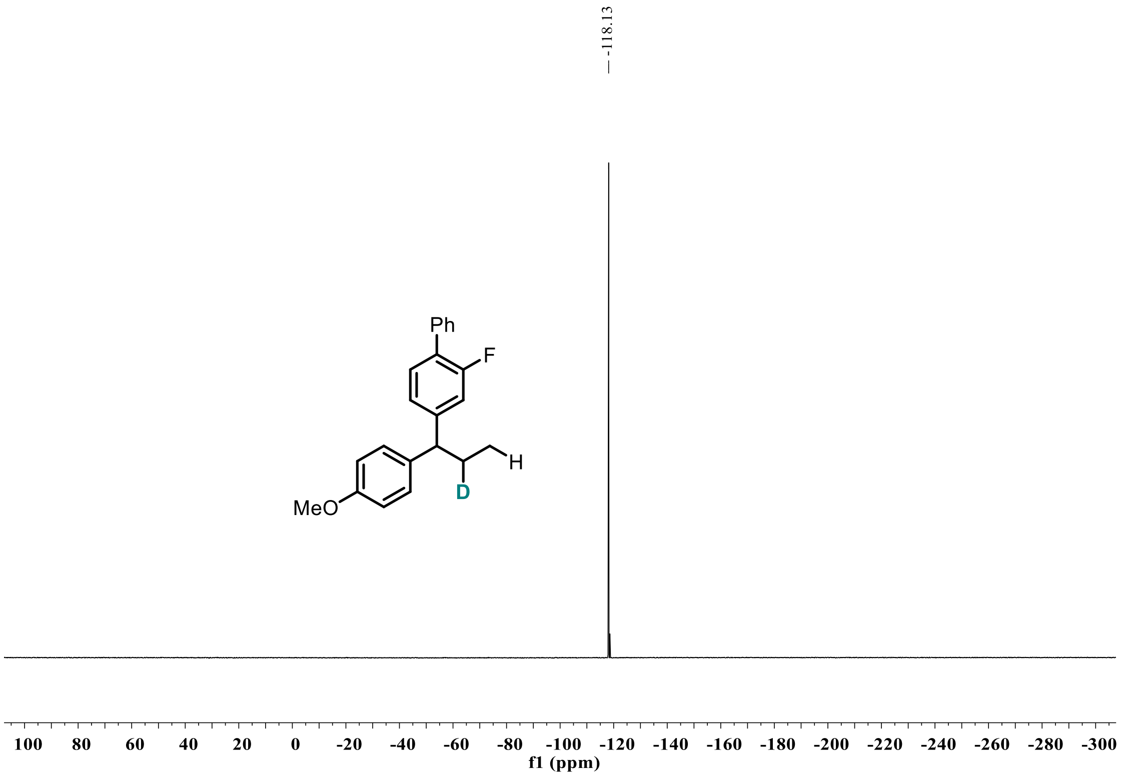

Supplementary Figure 80. ^{19}F NMR spectra for 3bk-D_2’

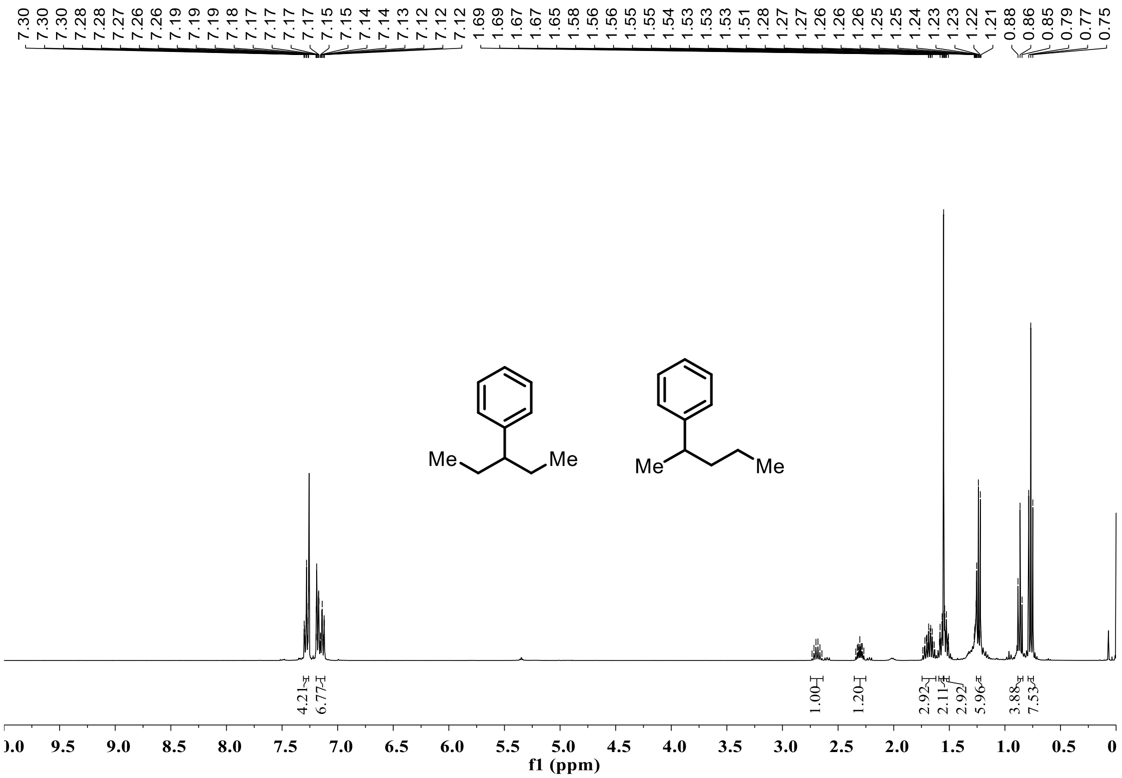

Supplementary Figure 81. ^{1}H NMR spectra for 7a and 7a’

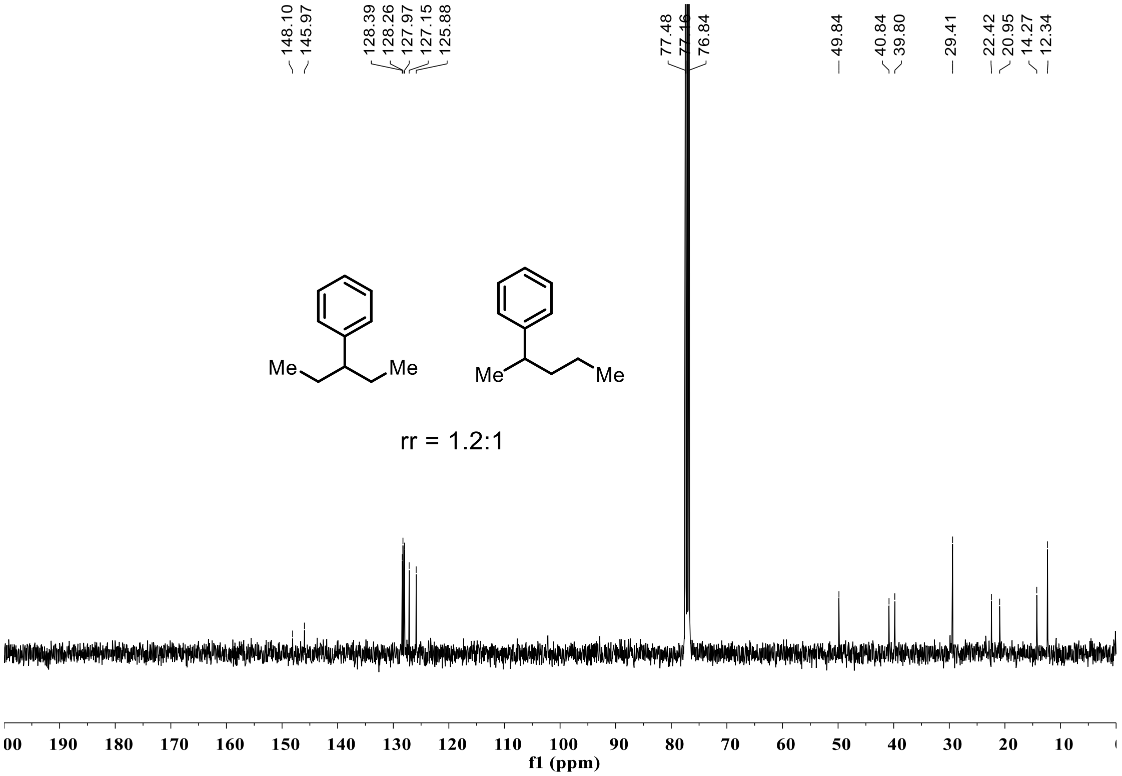

Supplementary Figure 82. ^{13}C NMR spectra for 7a and 7a’

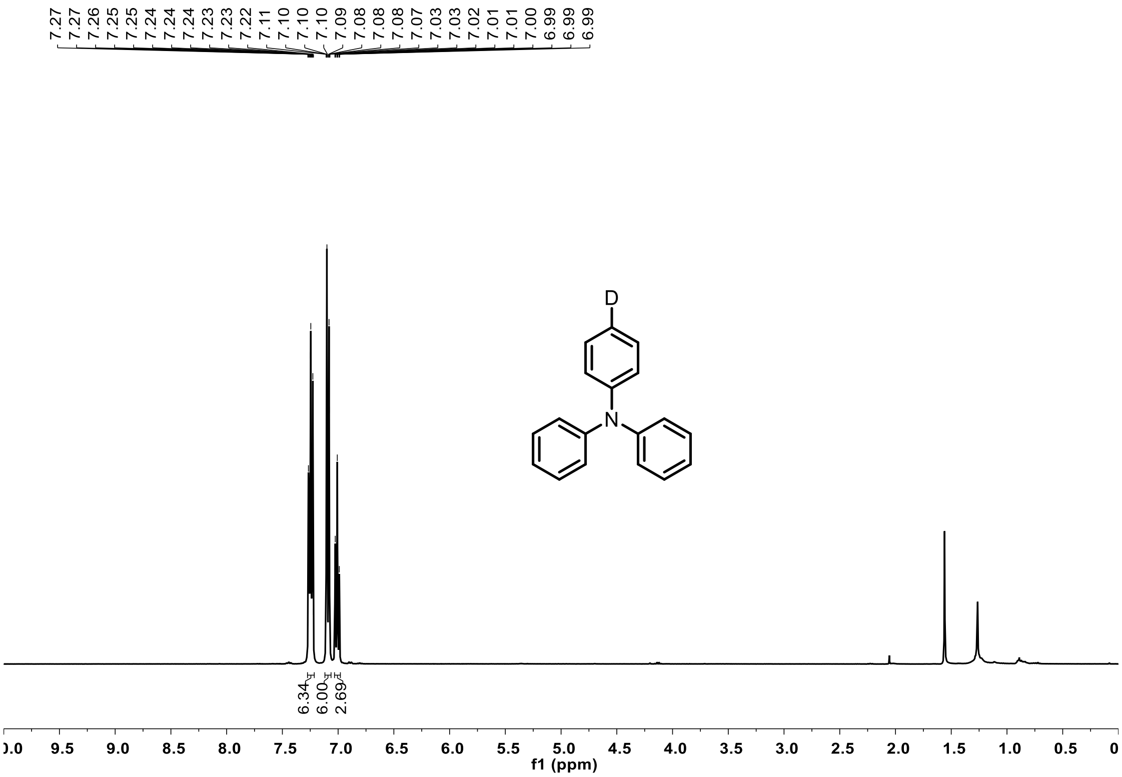

Supplementary Figure 83. ^{1}H NMR spectra for 8b

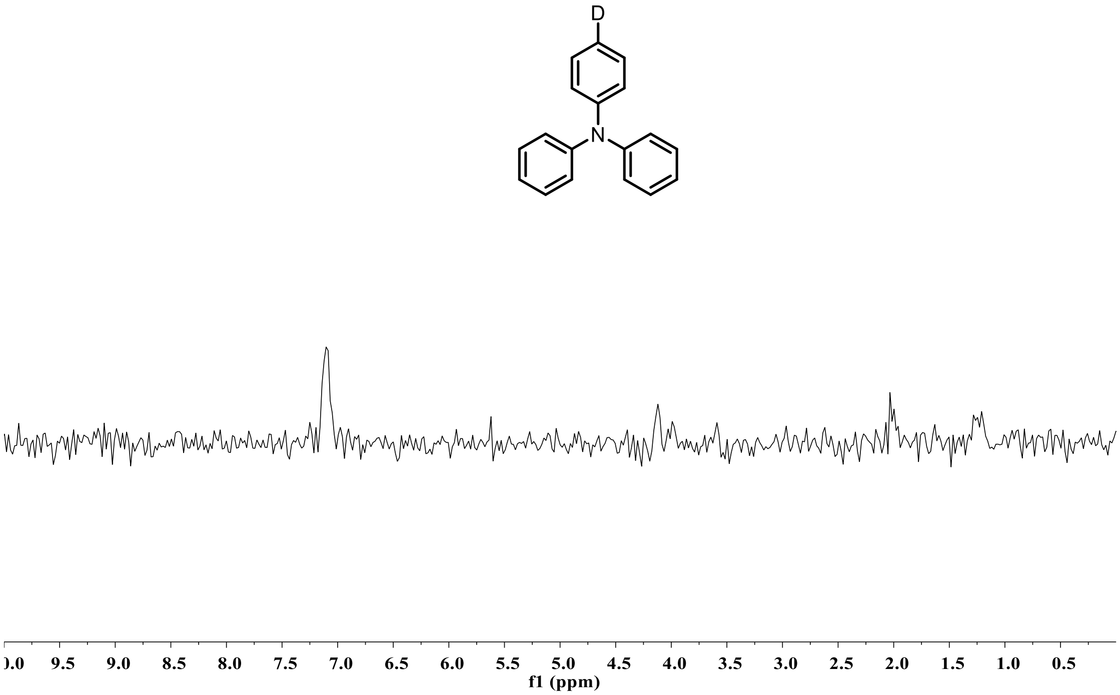

Supplementary Figure 84. ^{2}D NMR spectra for 8b

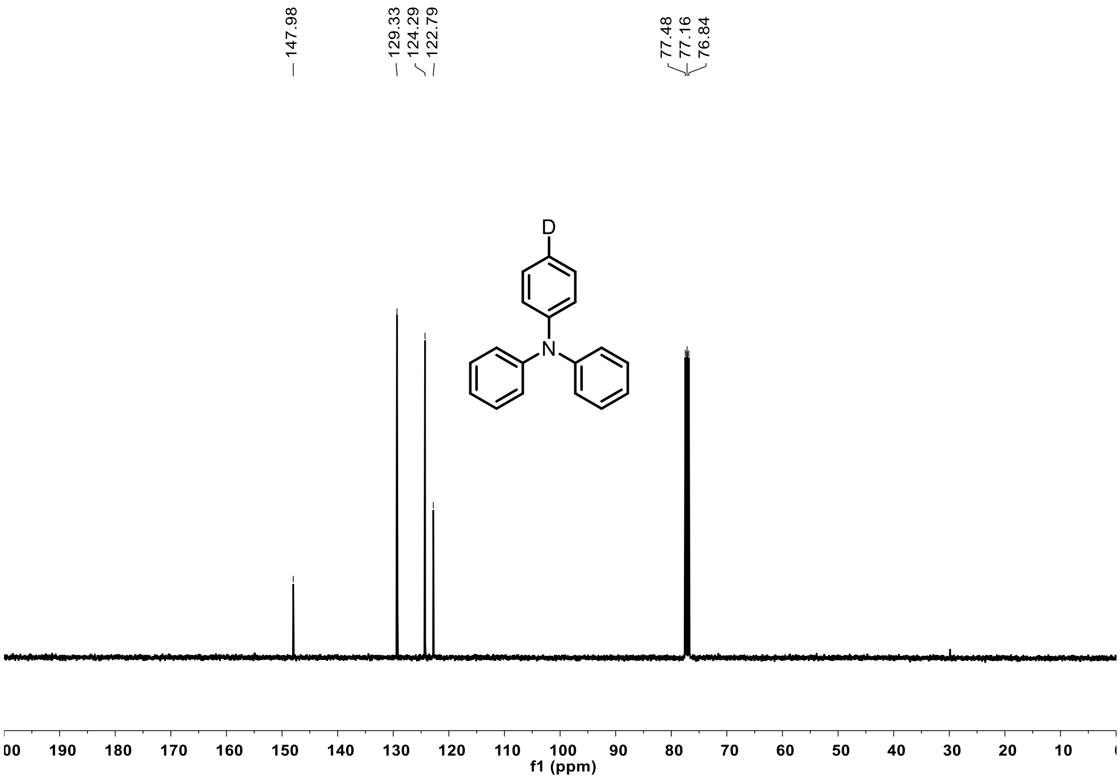

Supplementary Figure 85. ^{13}C NMR spectra for 8b

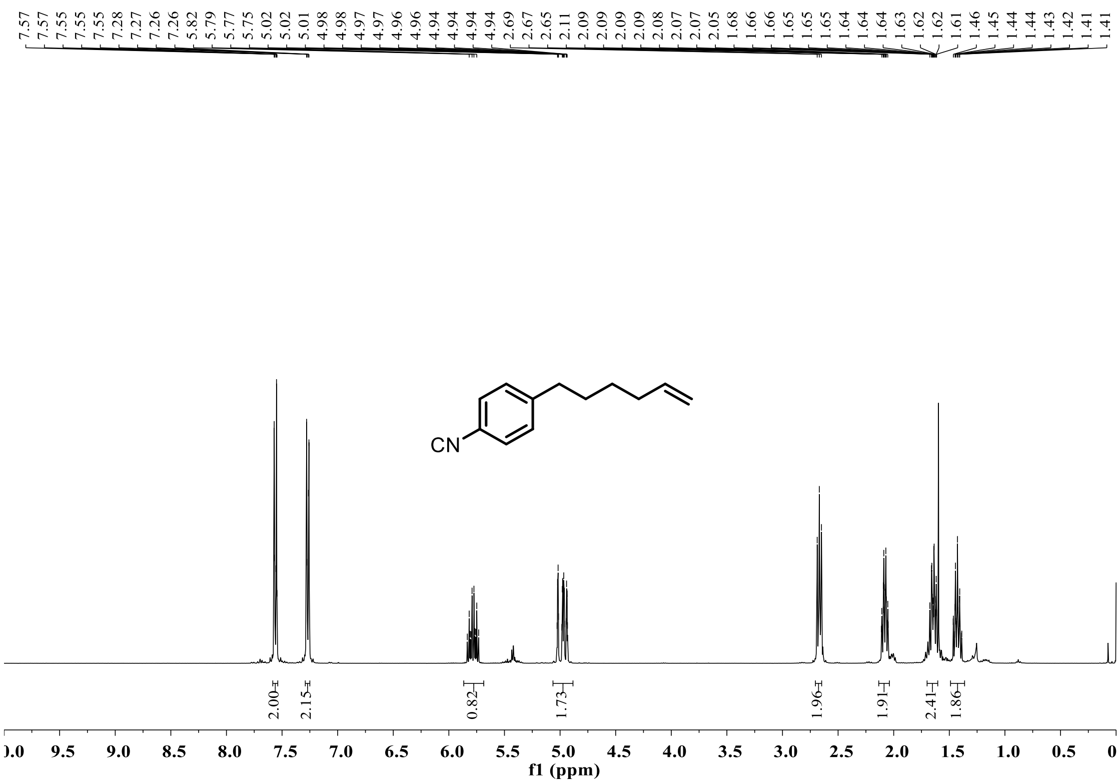

Supplementary Figure 86. ^{1}H NMR spectra for 11U

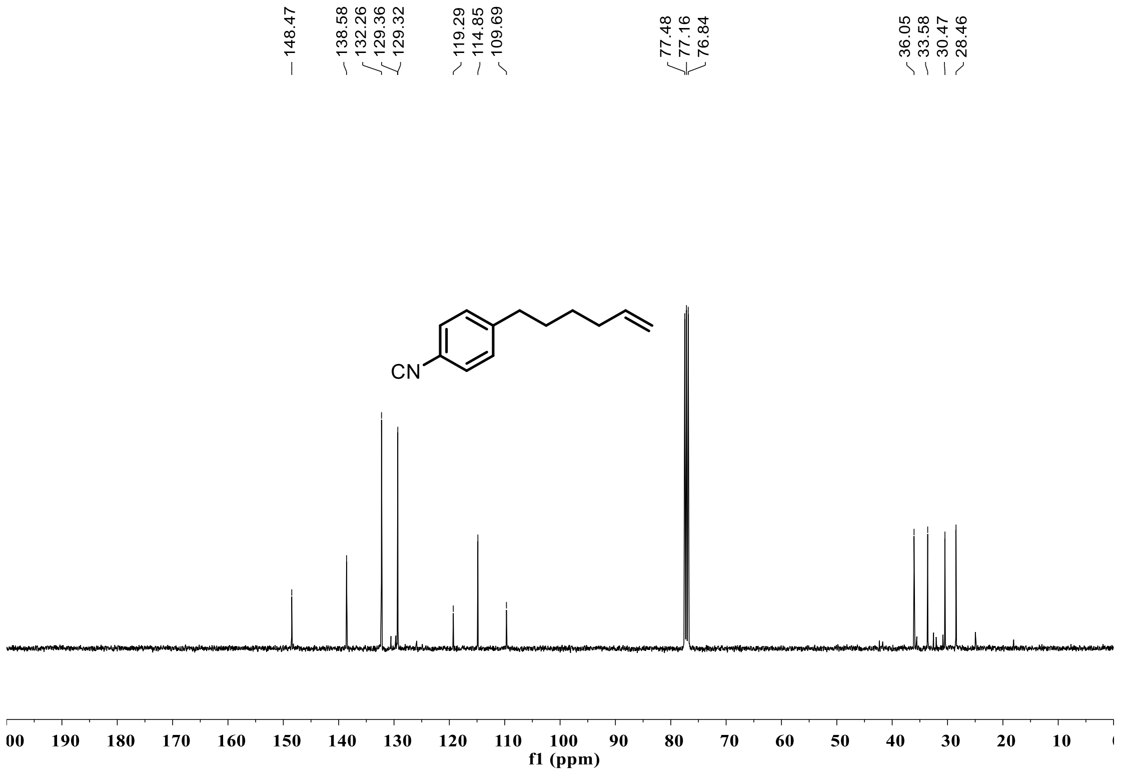

Supplementary Figure 87. ^{13}C NMR spectra for 11U

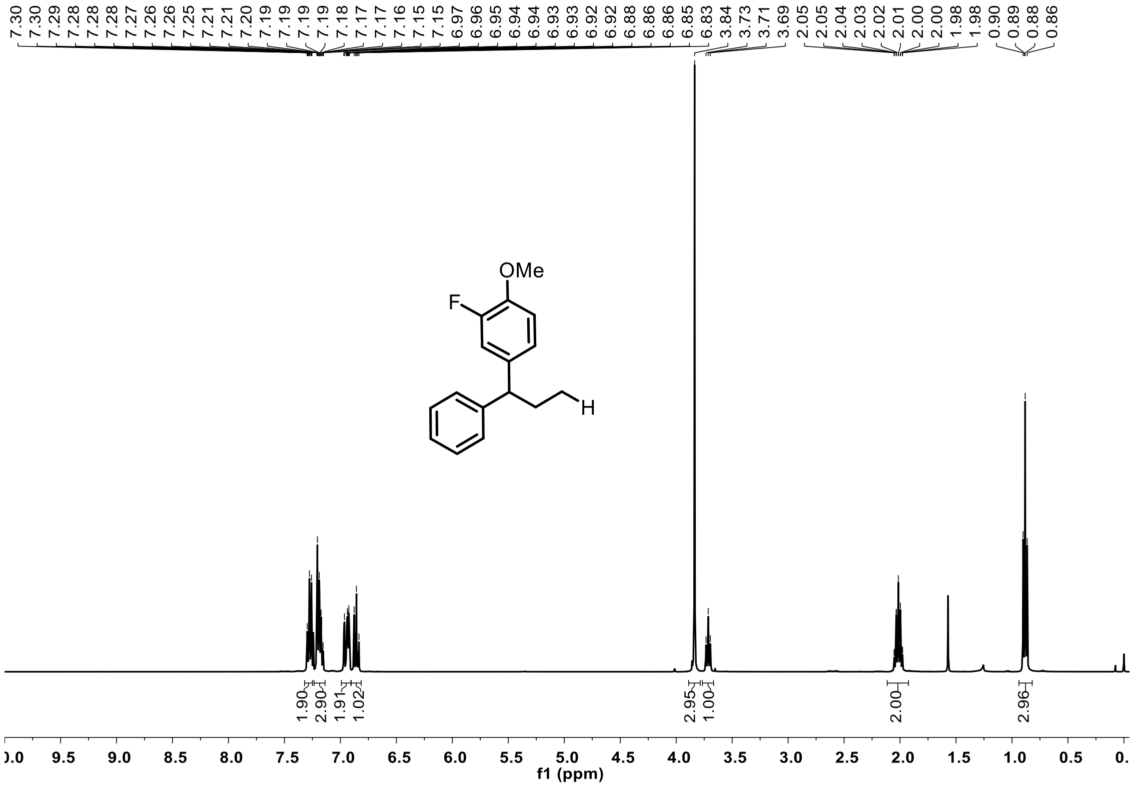

Supplementary Figure 88. ^{1}H NMR Spectrum of 3a

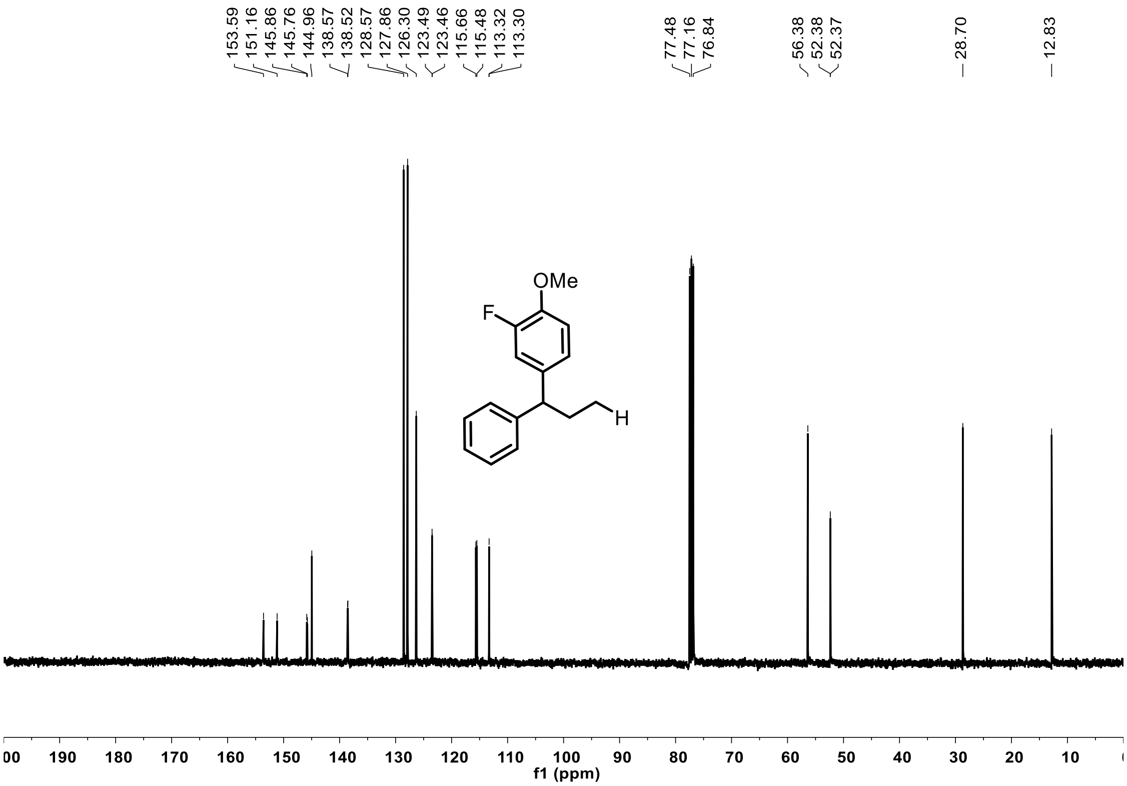

Supplementary Figure 89. ^{13}C NMR Spectrum of 3a

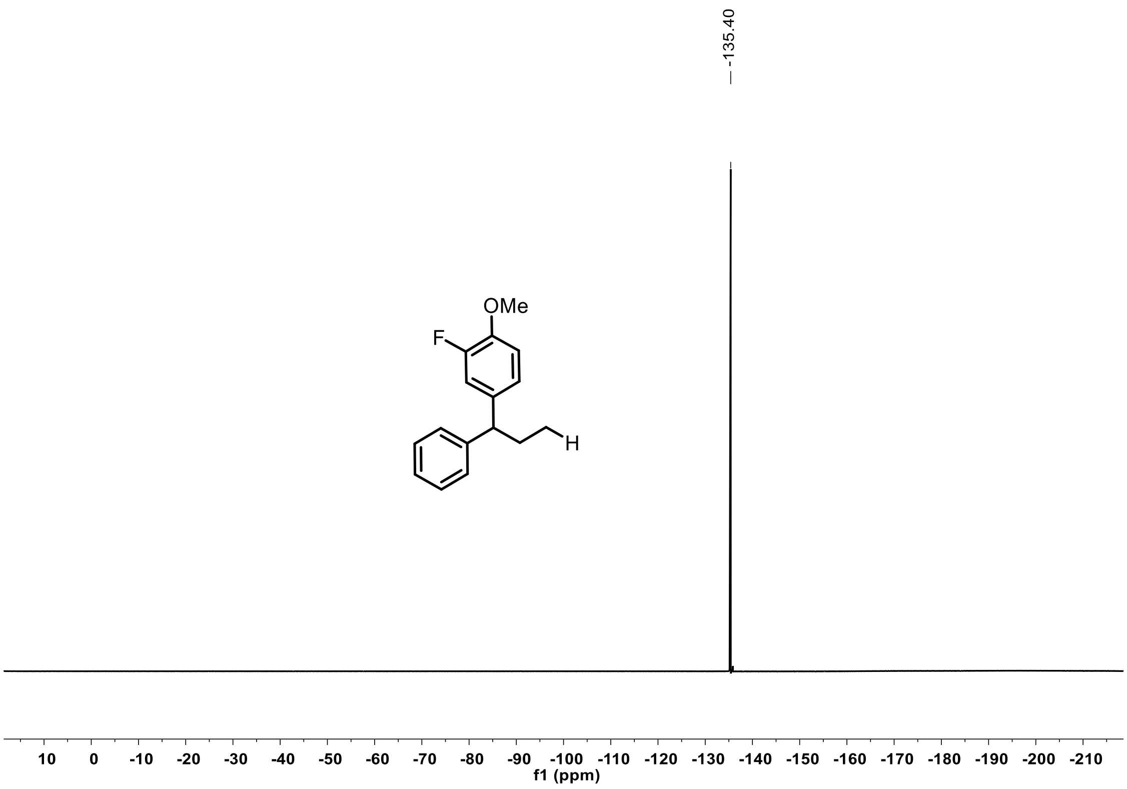

Supplementary Figure 90. ^{19}F NMR Spectrum of 3a

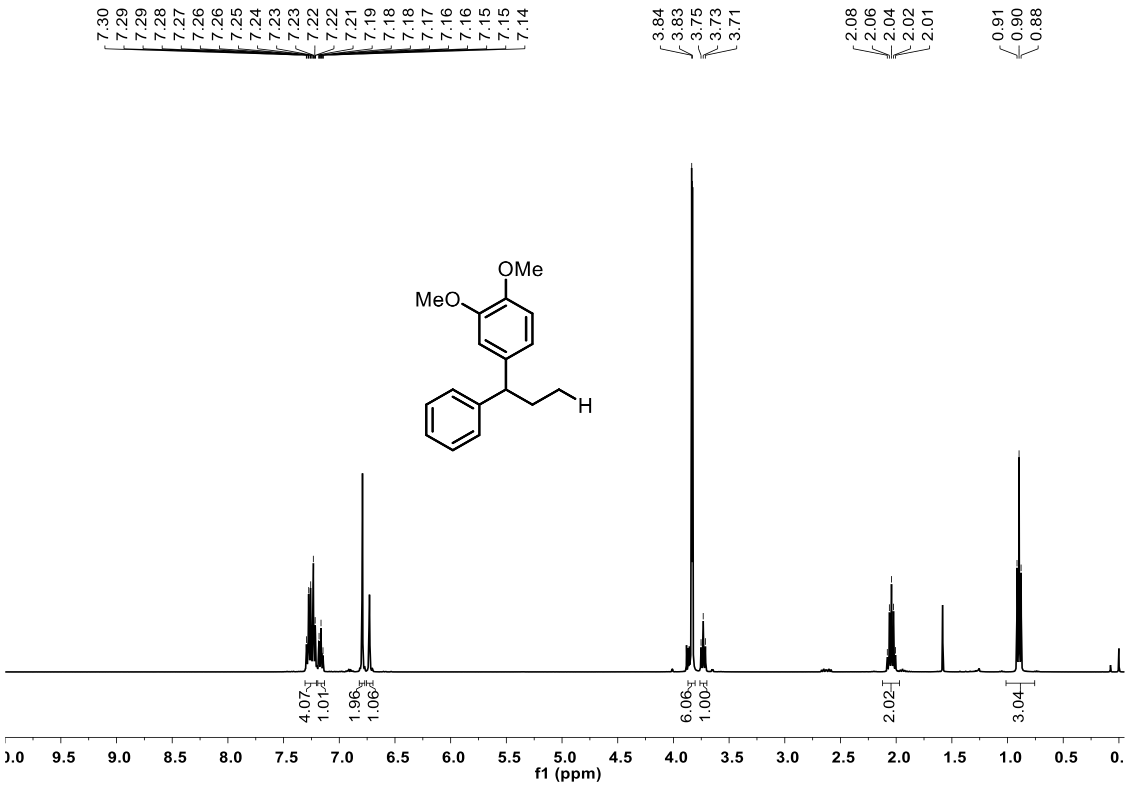

Supplementary Figure 91. ^{1}H NMR Spectrum of 3b

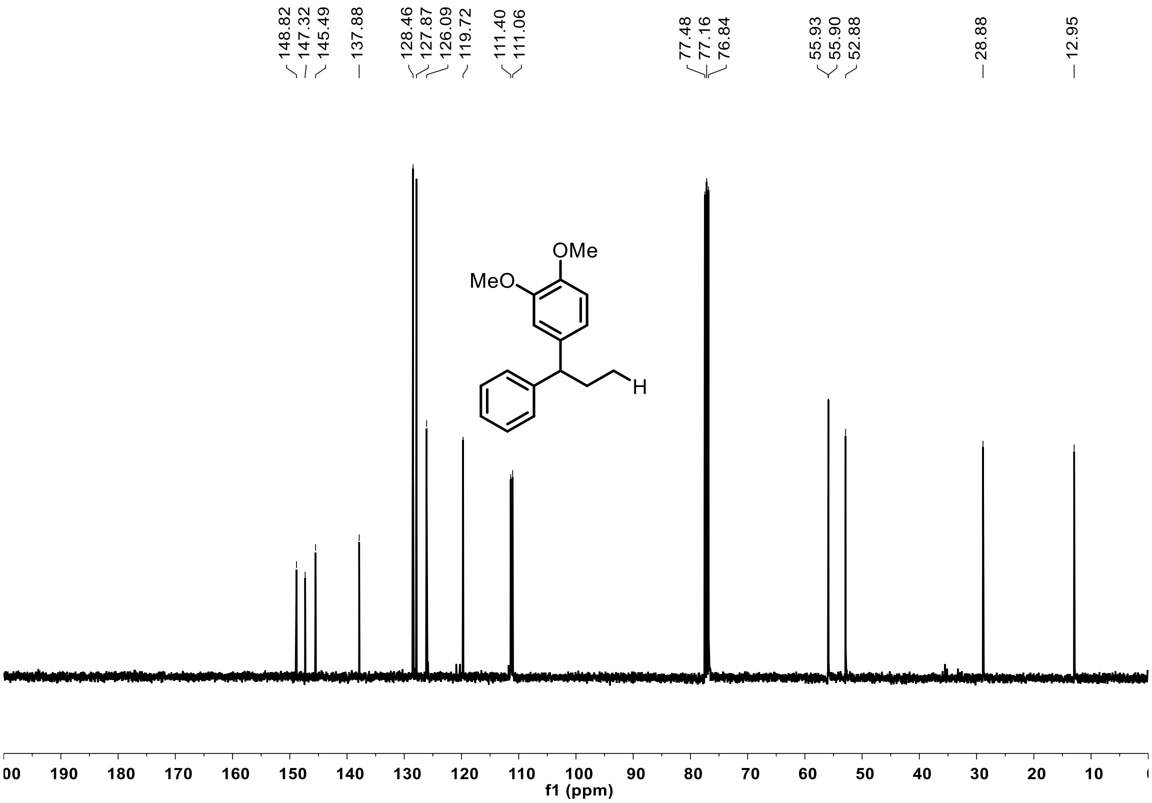

Supplementary Figure 92. ^{13}C NMR Spectrum of 3b

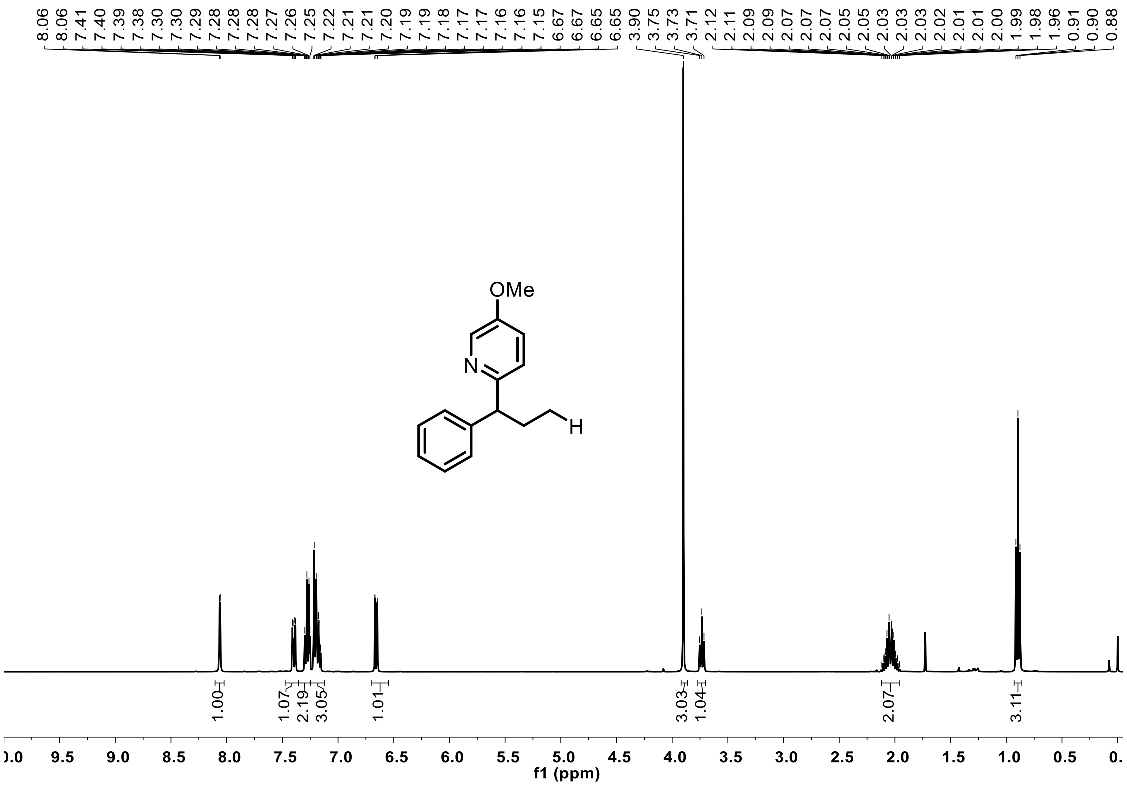

Supplementary Figure 93. ^{1}H NMR Spectrum of 3c

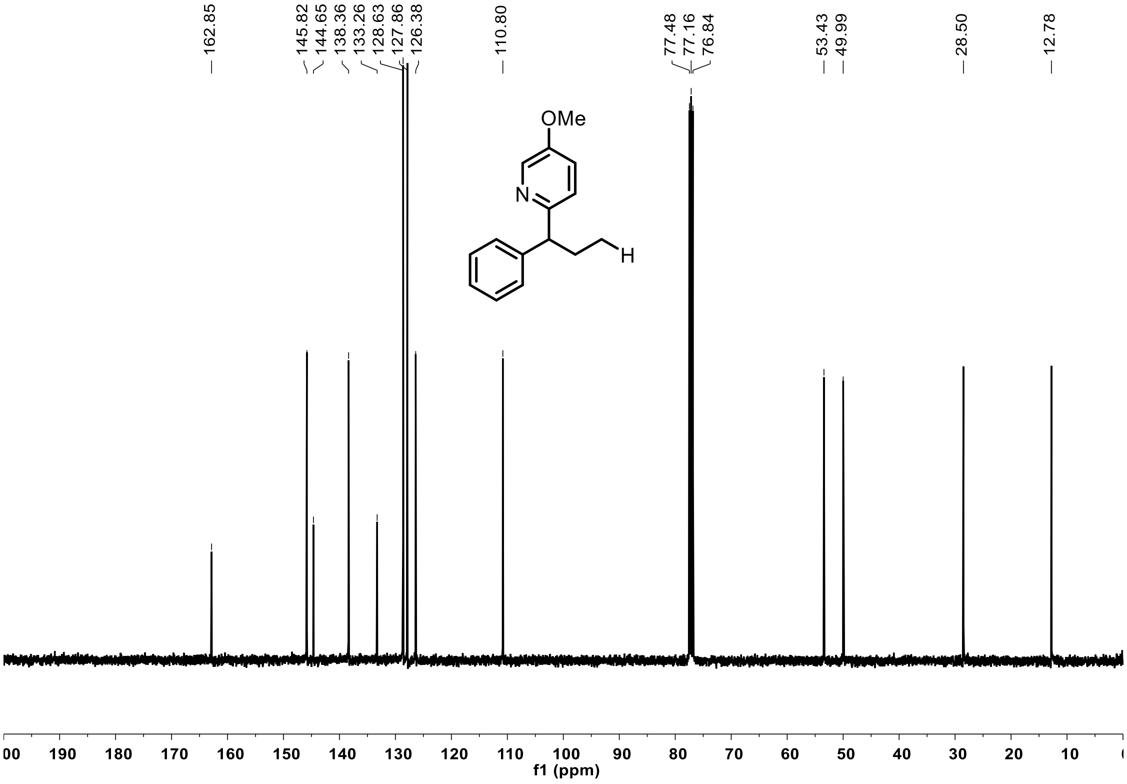

Supplementary Figure 94. ^{13}C NMR Spectrum of 3c

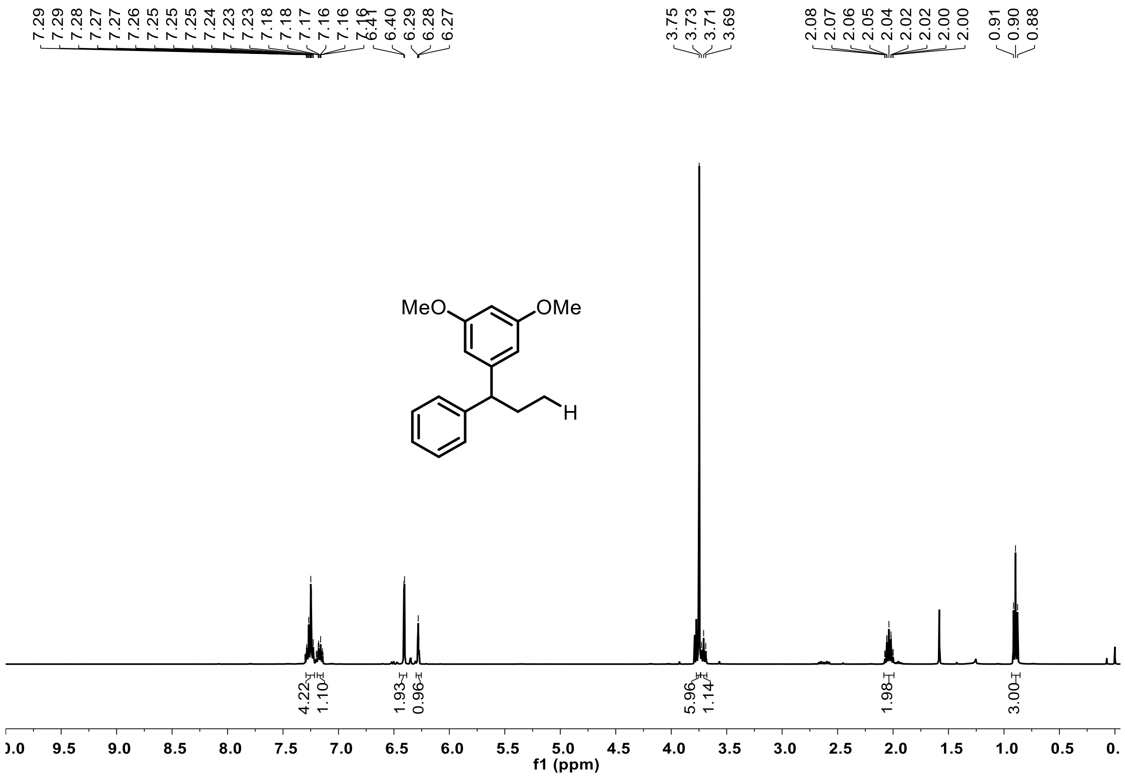

Supplementary Figure 95. ^{1}H NMR Spectrum of 3d

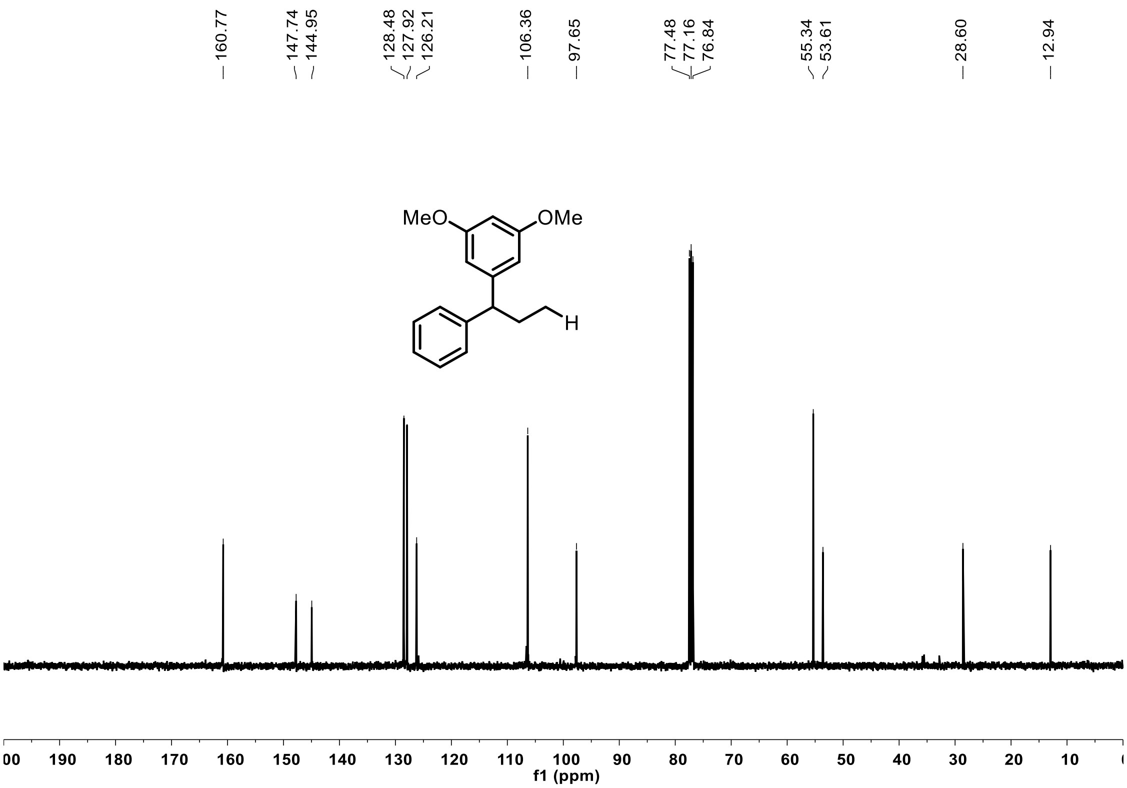

Supplementary Figure 96. ^{13}C NMR Spectrum of 3d

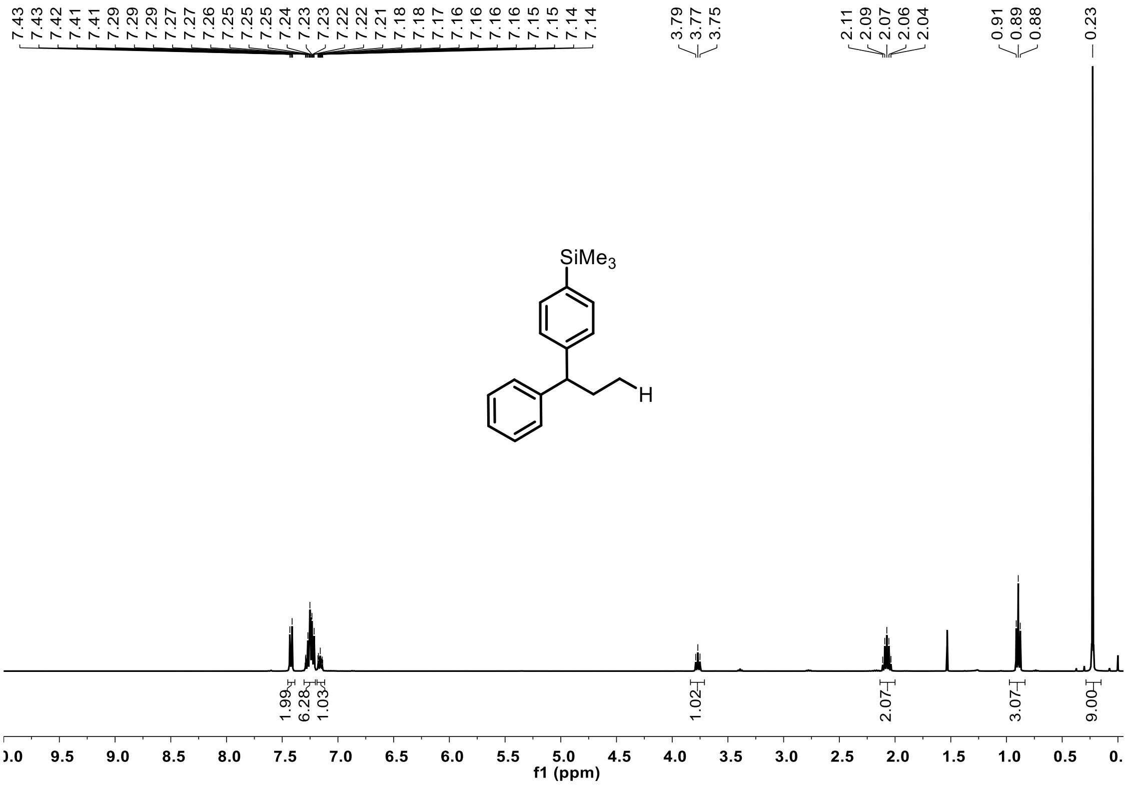

Supplementary Figure 97. ^{1}H NMR Spectrum of 3e

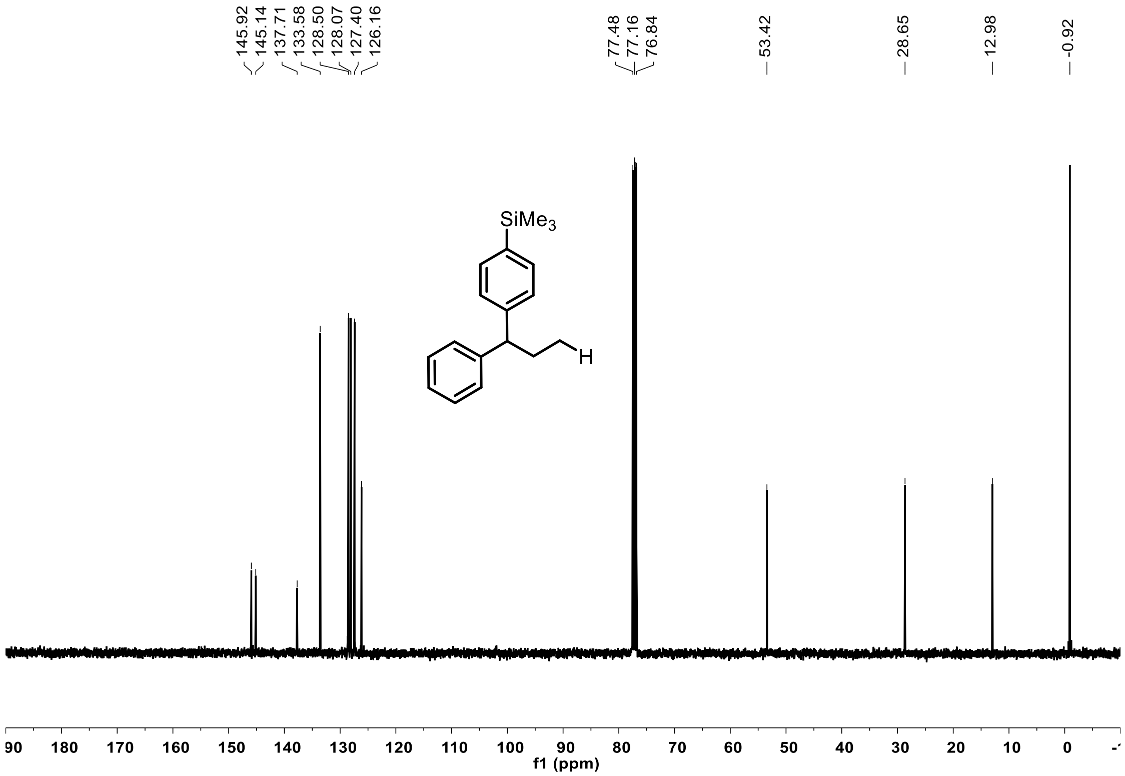

Supplementary Figure 98. ^{13}C NMR Spectrum of 3e

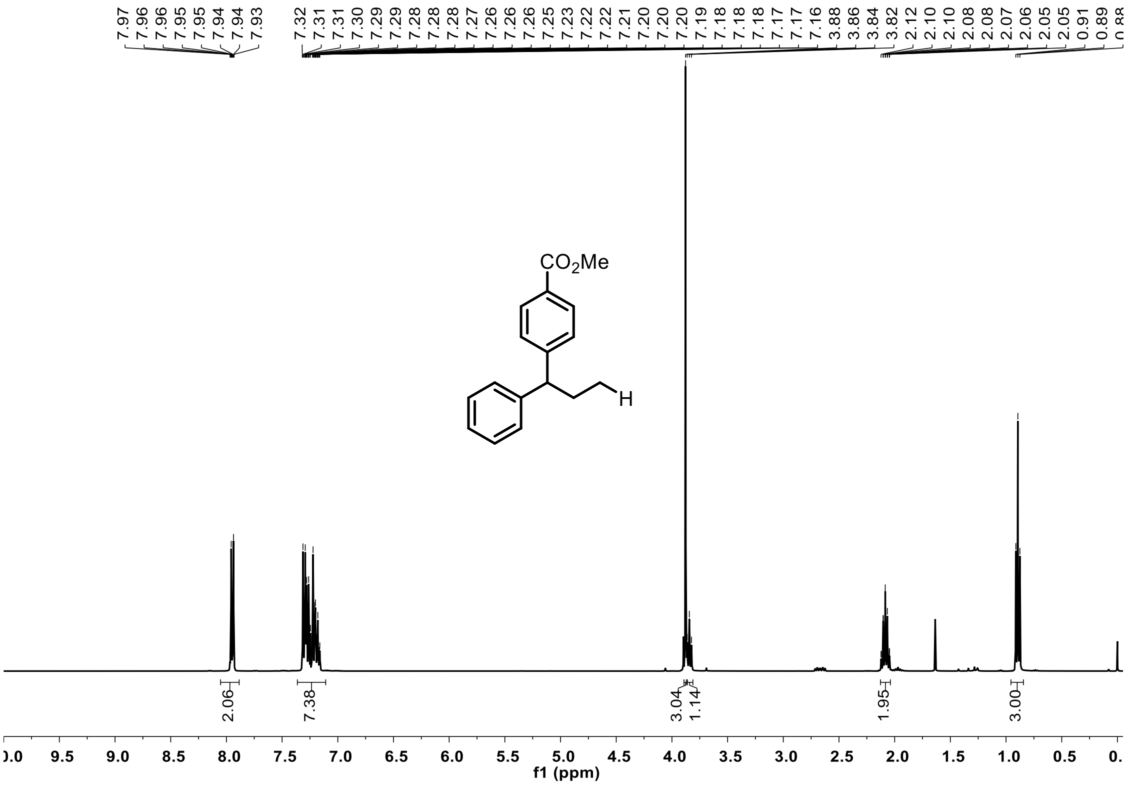

Supplementary Figure 99. ^{1}H NMR Spectrum of 3f

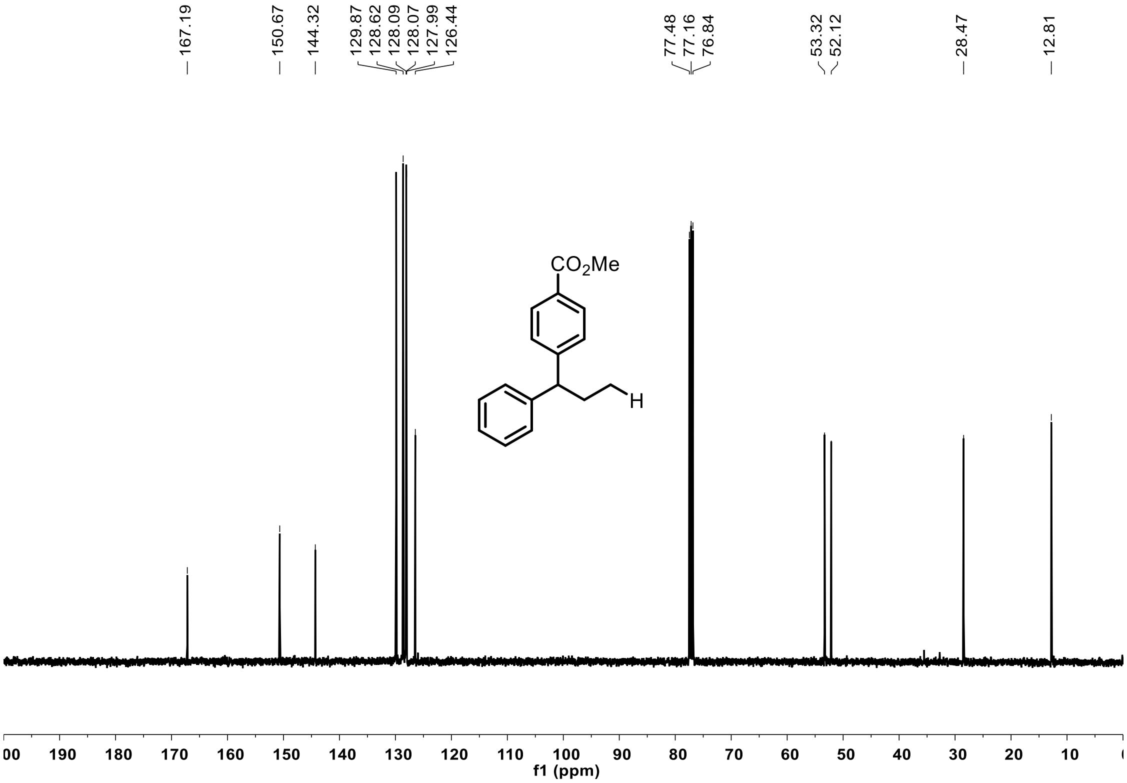

Supplementary Figure 100. ^{13}C NMR Spectrum of 3f

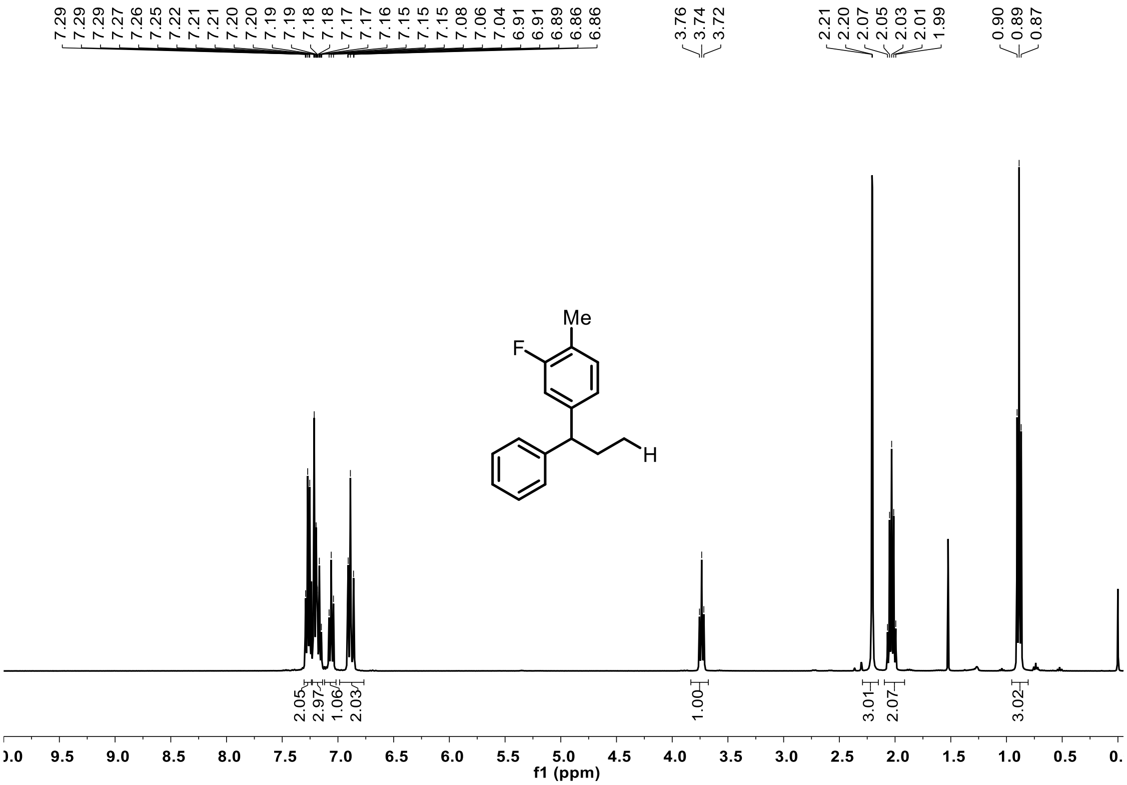

Supplementary Figure 101. ^{1}H NMR Spectrum of 3g

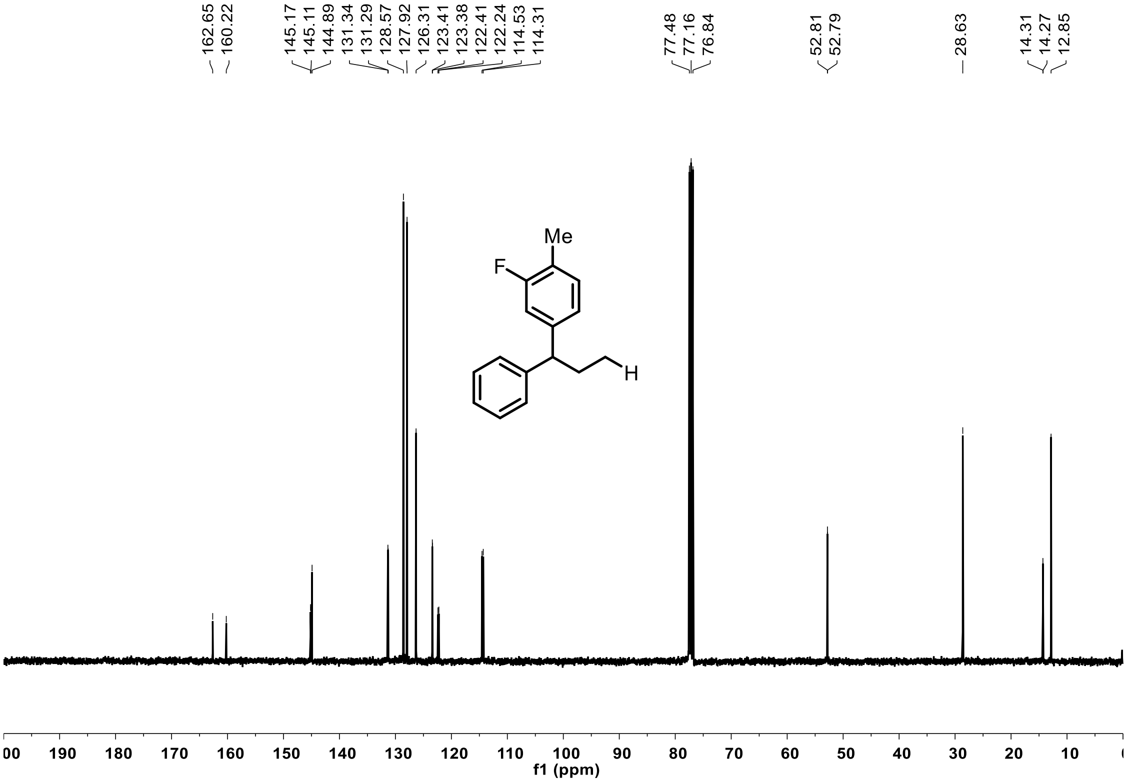

Supplementary Figure 102. ^{13}C NMR Spectrum of 3g

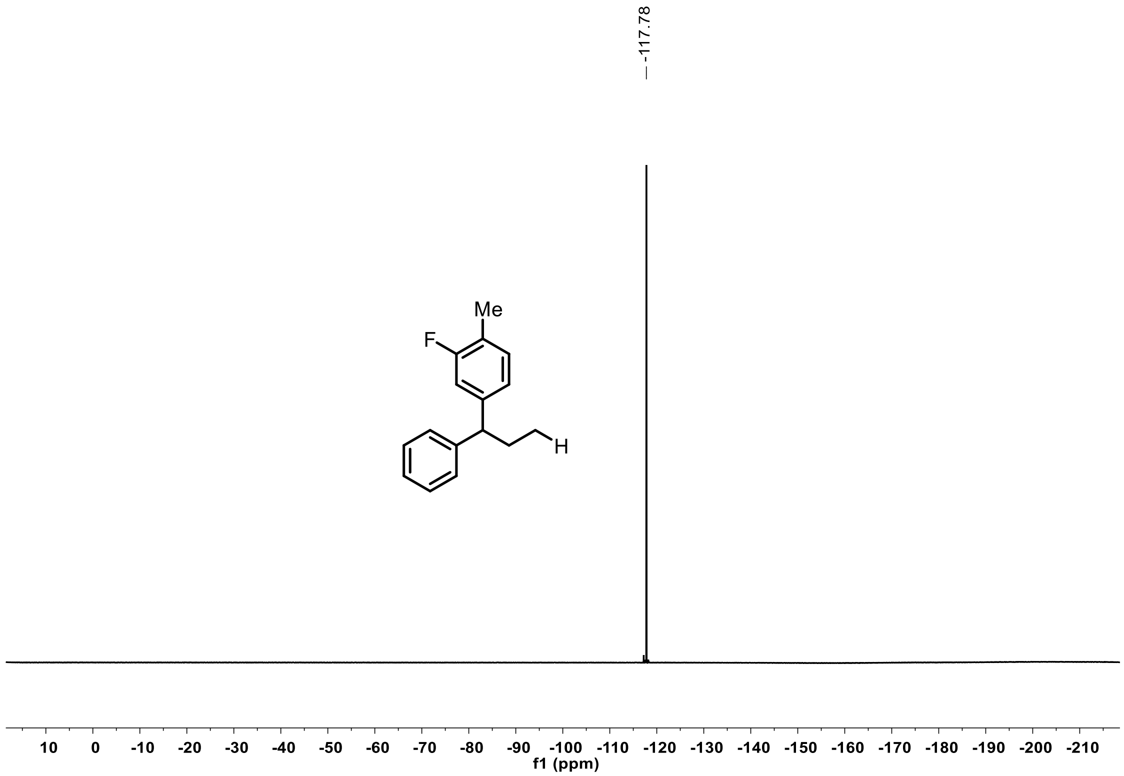

Supplementary Figure 103. ^{19}F NMR Spectrum of 3g

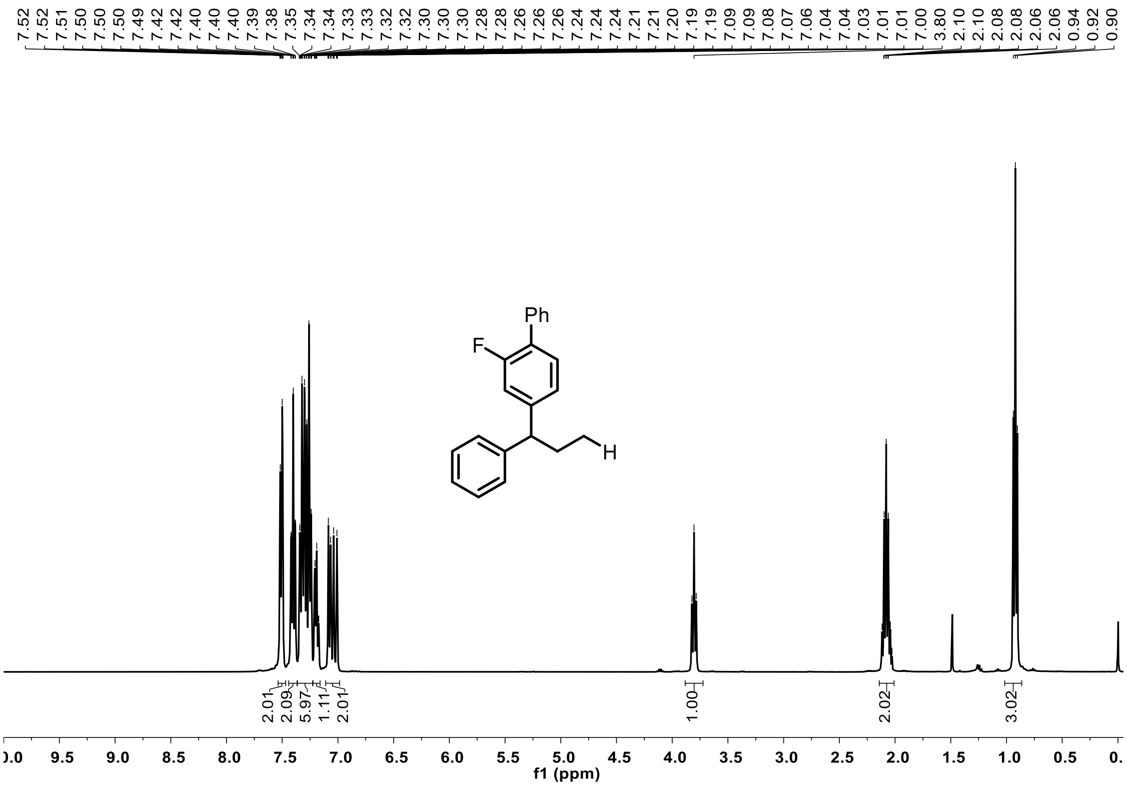

Supplementary Figure 104. ^{1}H NMR Spectrum of 3h

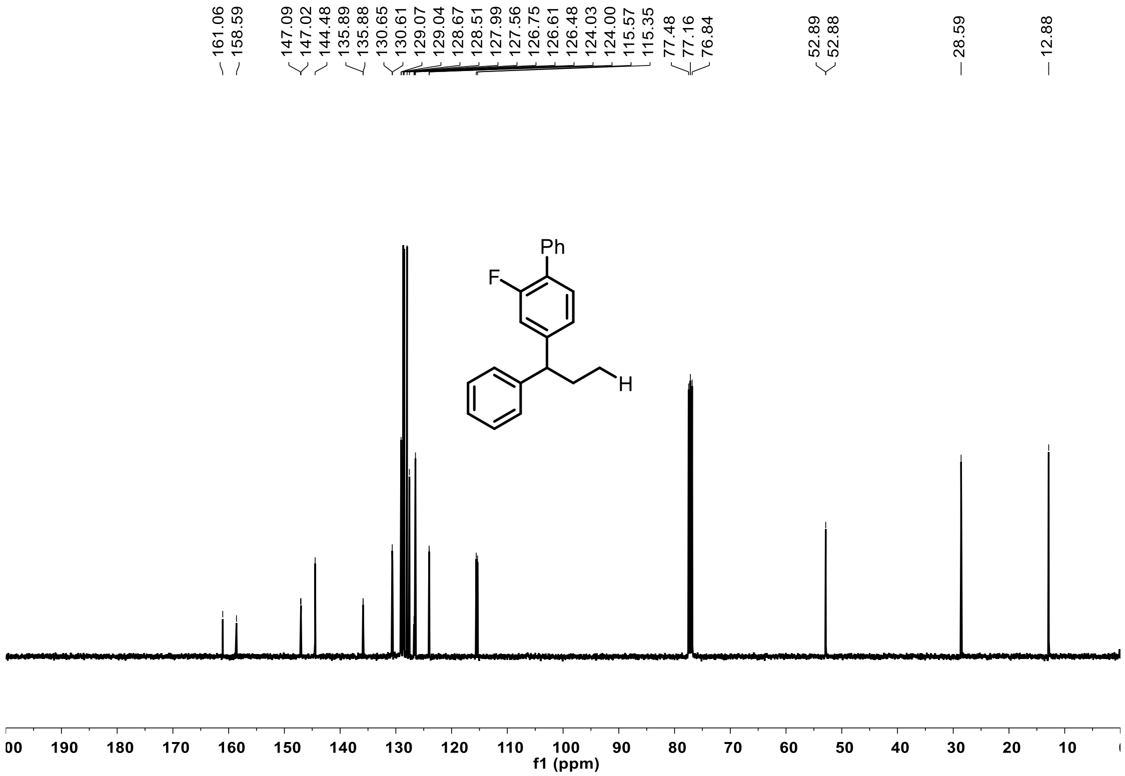

Supplementary Figure 105. ^{13}C NMR Spectrum of 3h

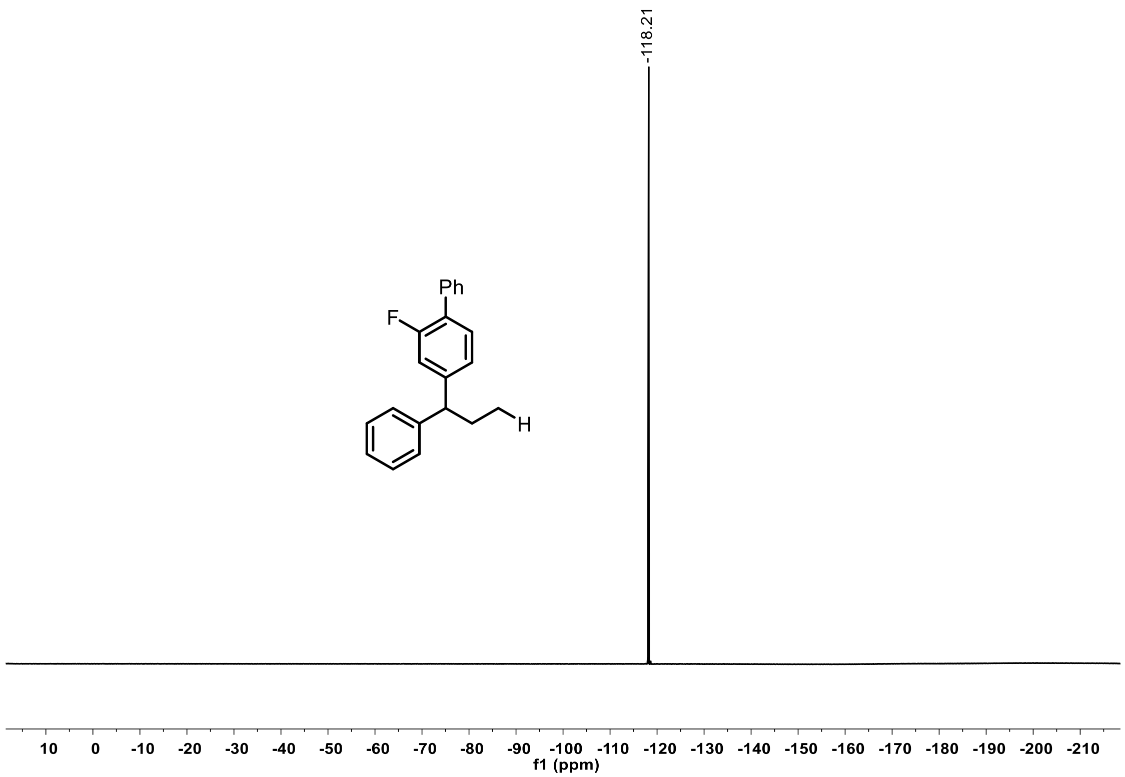

Supplementary Figure 106. ^{19}F NMR Spectrum of 3h

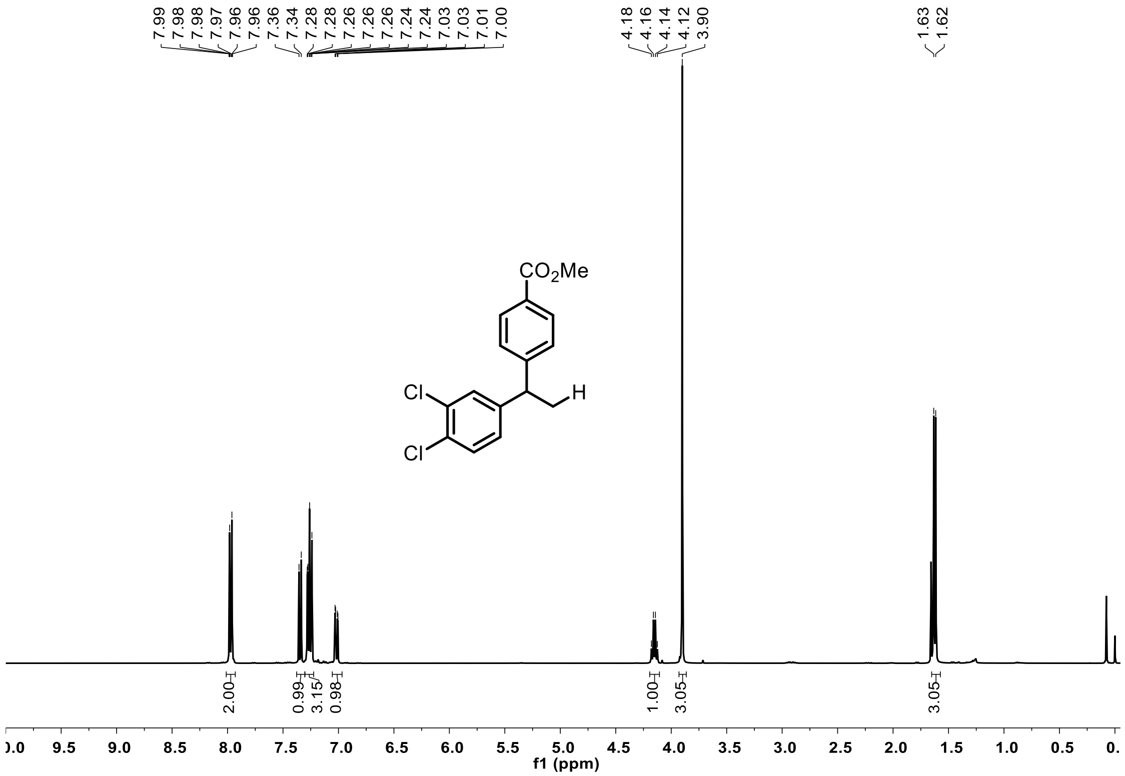

Supplementary Figure 107. ^{1}H NMR Spectrum of 3i

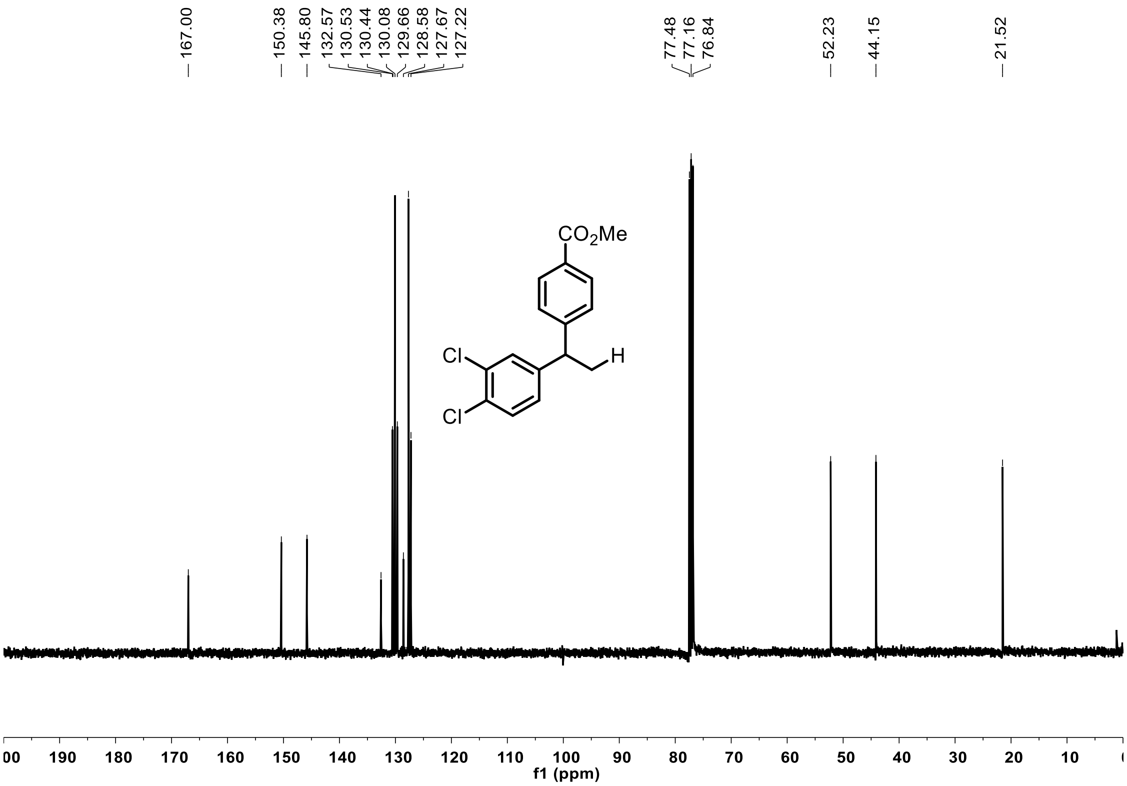

Supplementary Figure 108. ^{13}C NMR Spectrum of 3i

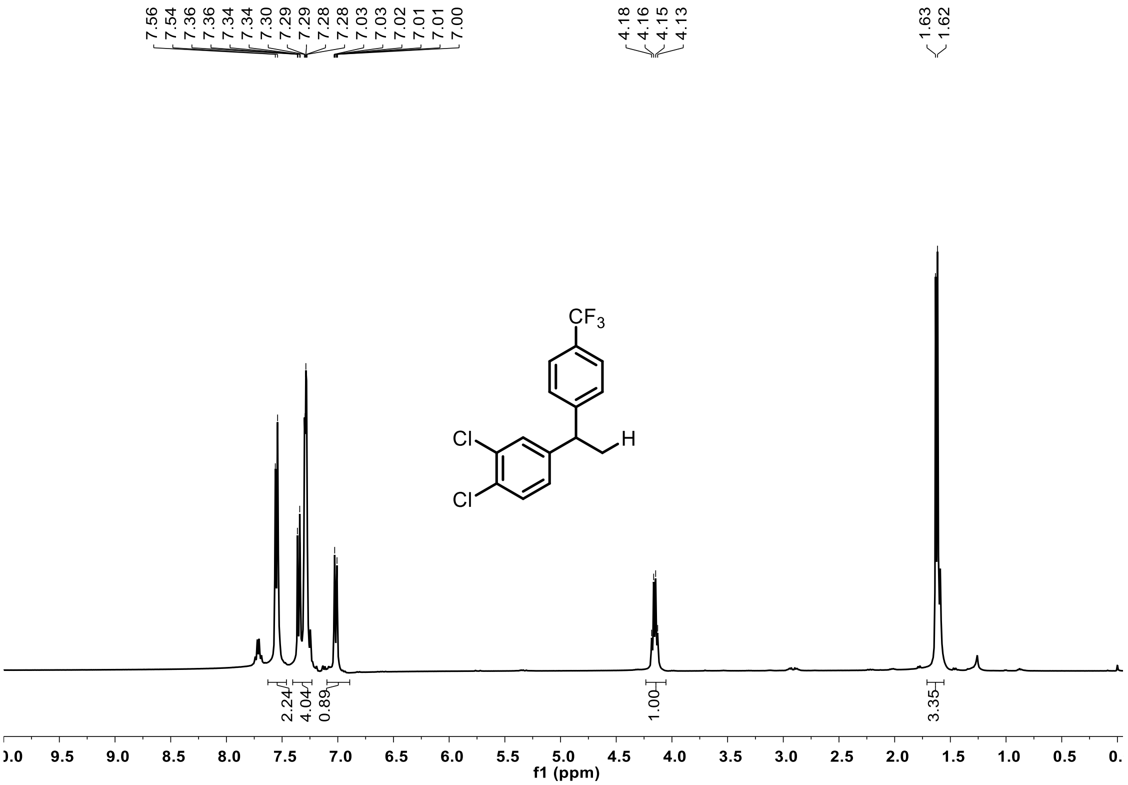

Supplementary Figure 109. ^{1}H NMR Spectrum of 3j

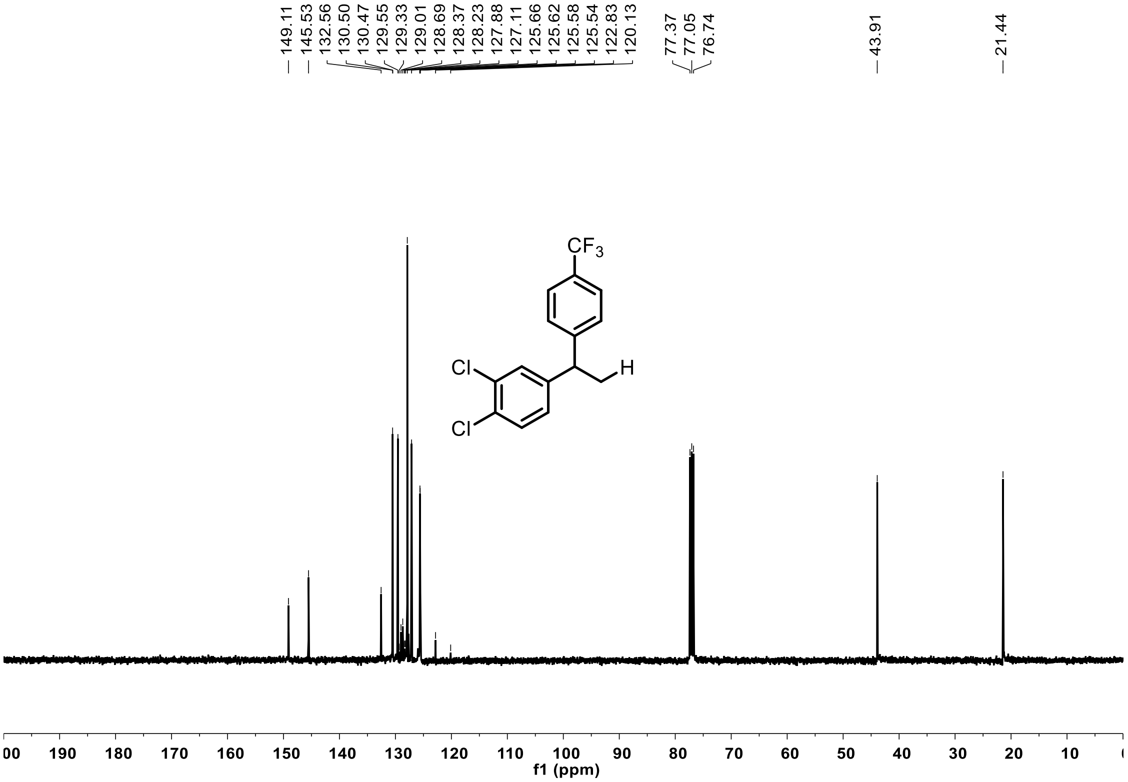

Supplementary Figure 110. ^{13}C NMR Spectrum of 3j

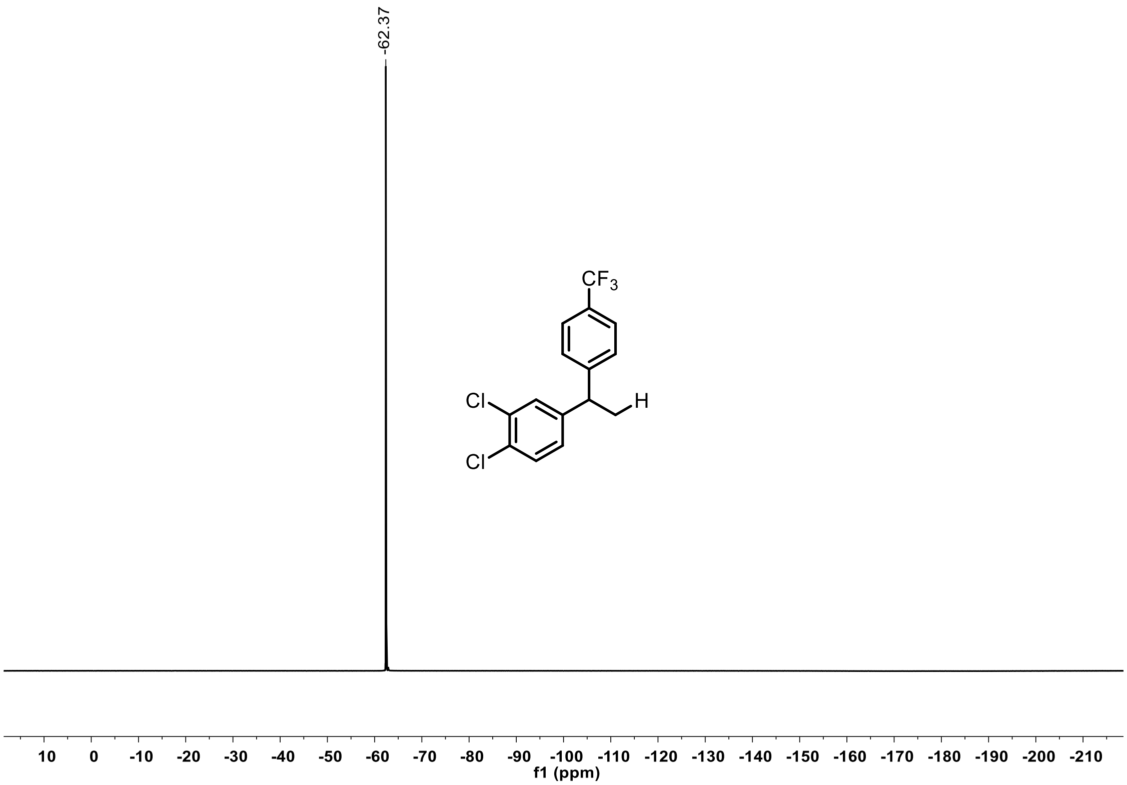

Supplementary Figure 111. ^{19}F NMR Spectrum of 3j

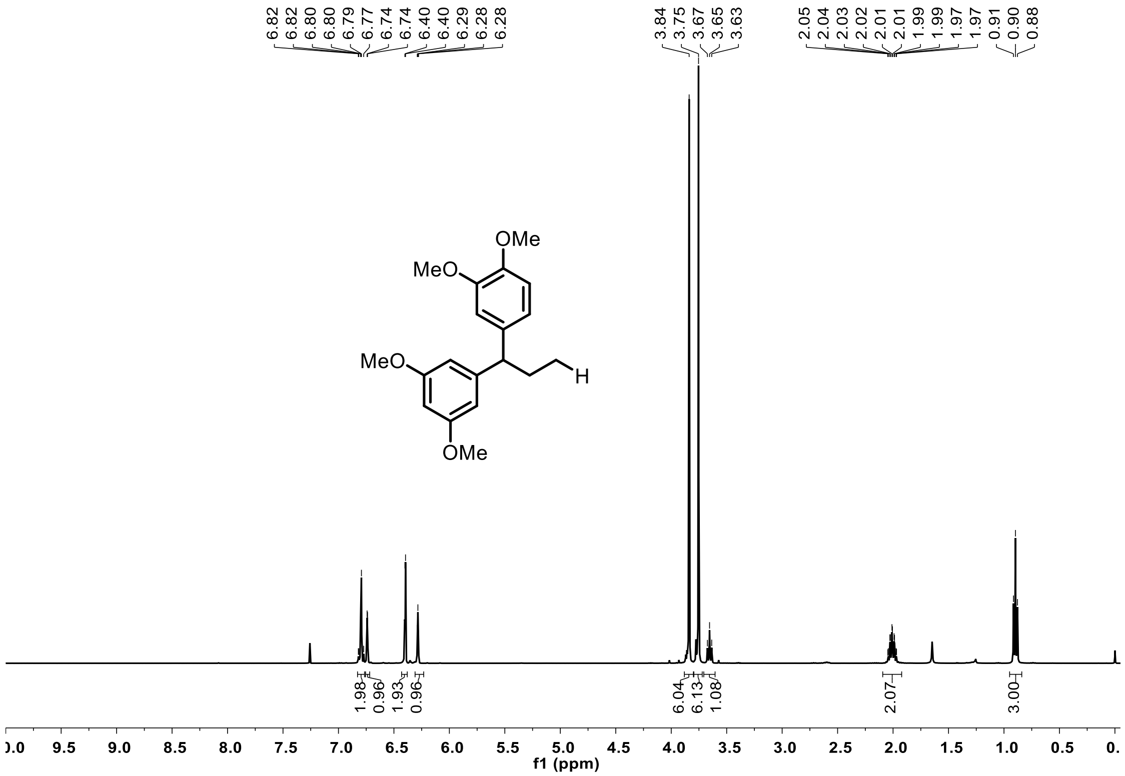

Supplementary Figure 112. ^{1}H NMR Spectrum of 3k

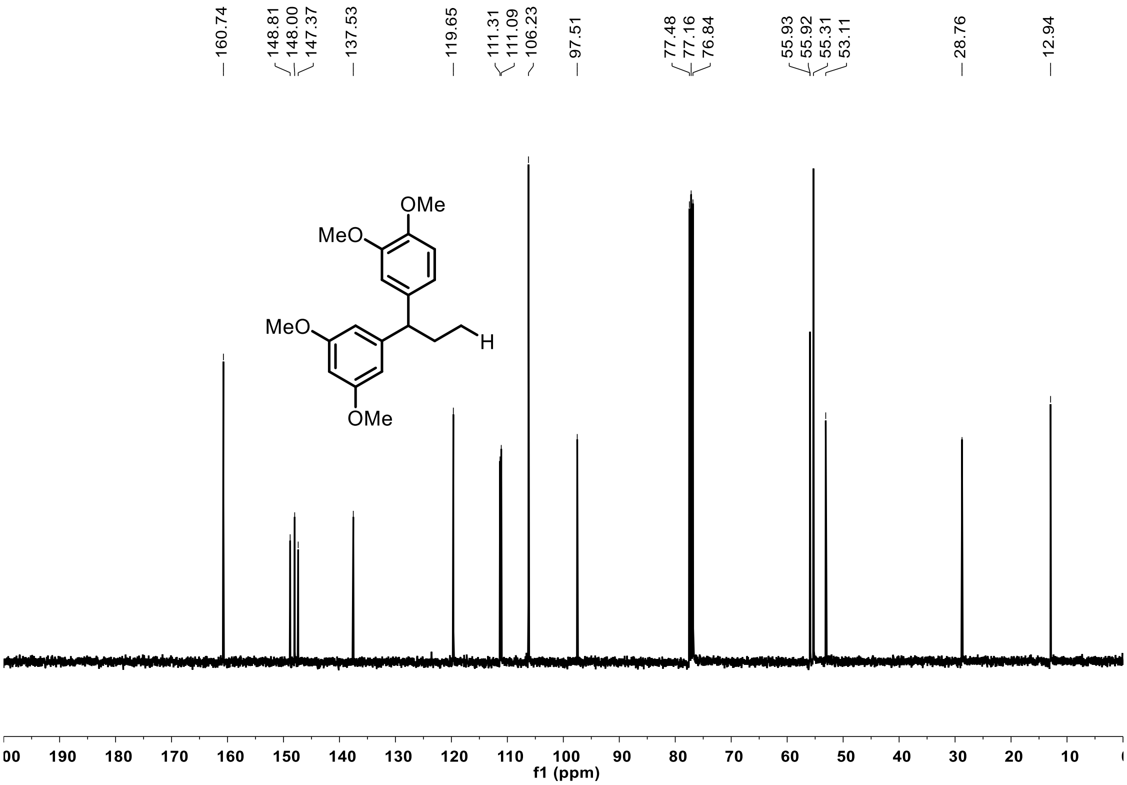

Supplementary Figure 113. ^{13}C NMR Spectrum of 3k

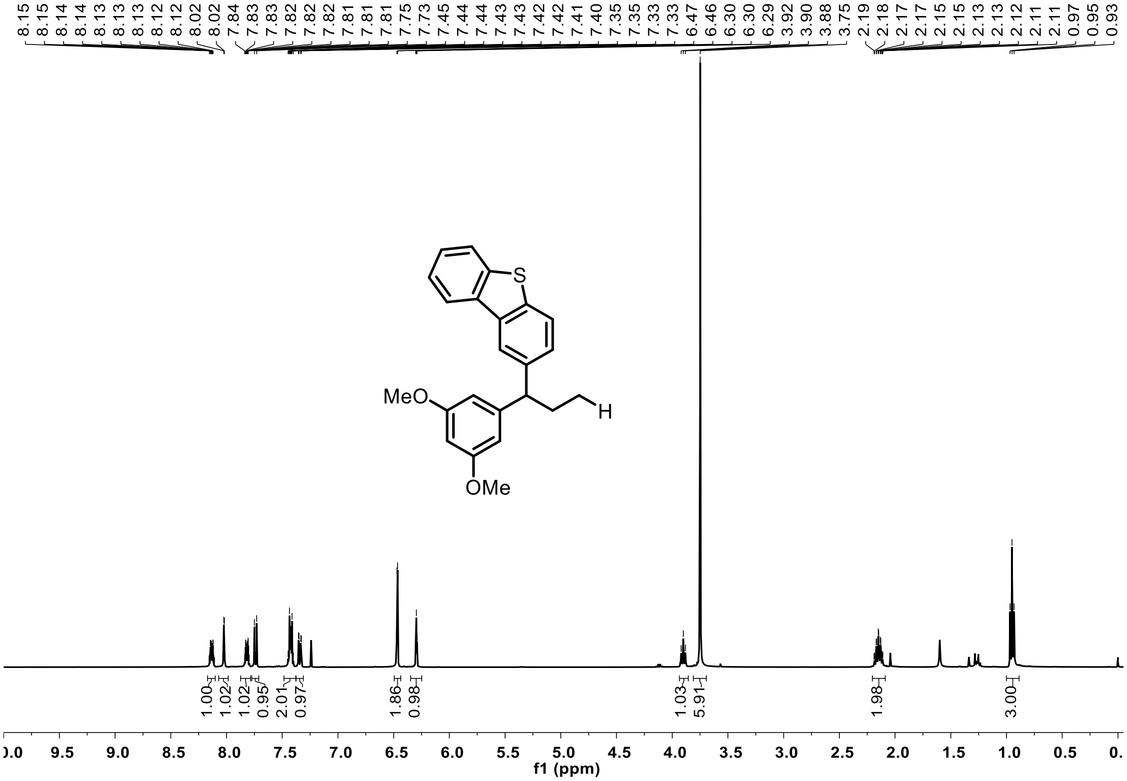

Supplementary Figure 114. ^{1}H NMR Spectrum of 3l

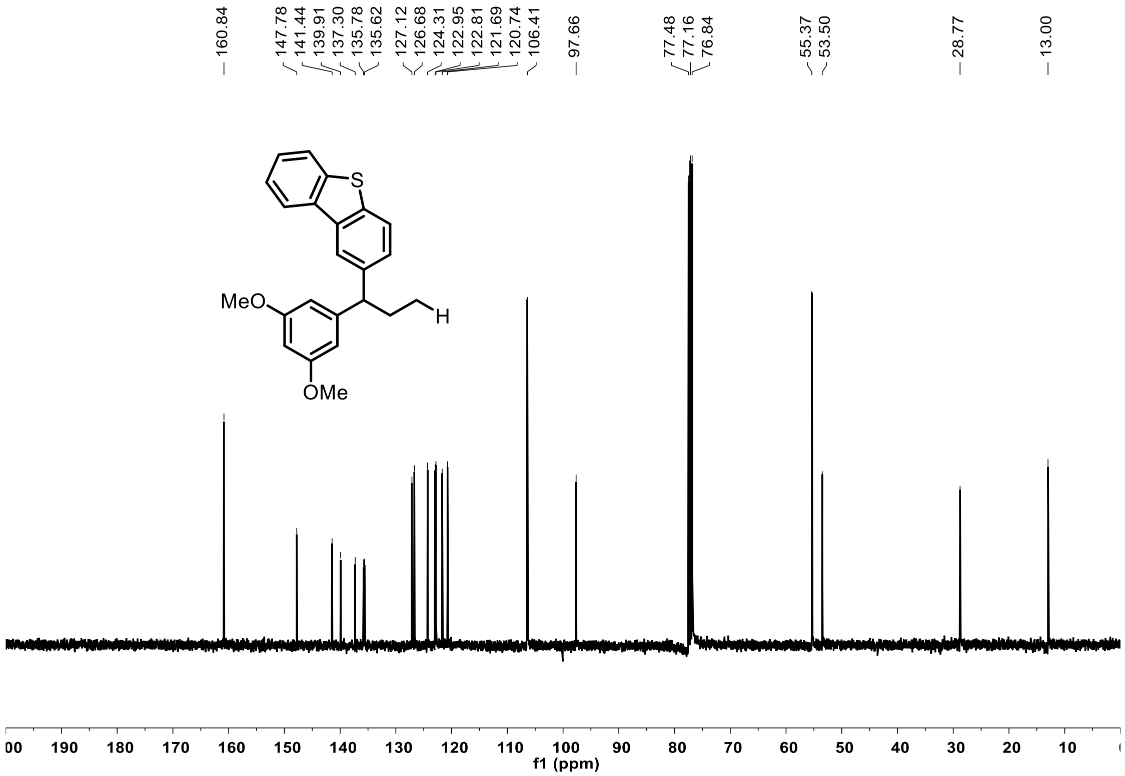

Supplementary Figure 115. ^{13}C NMR Spectrum of 3l

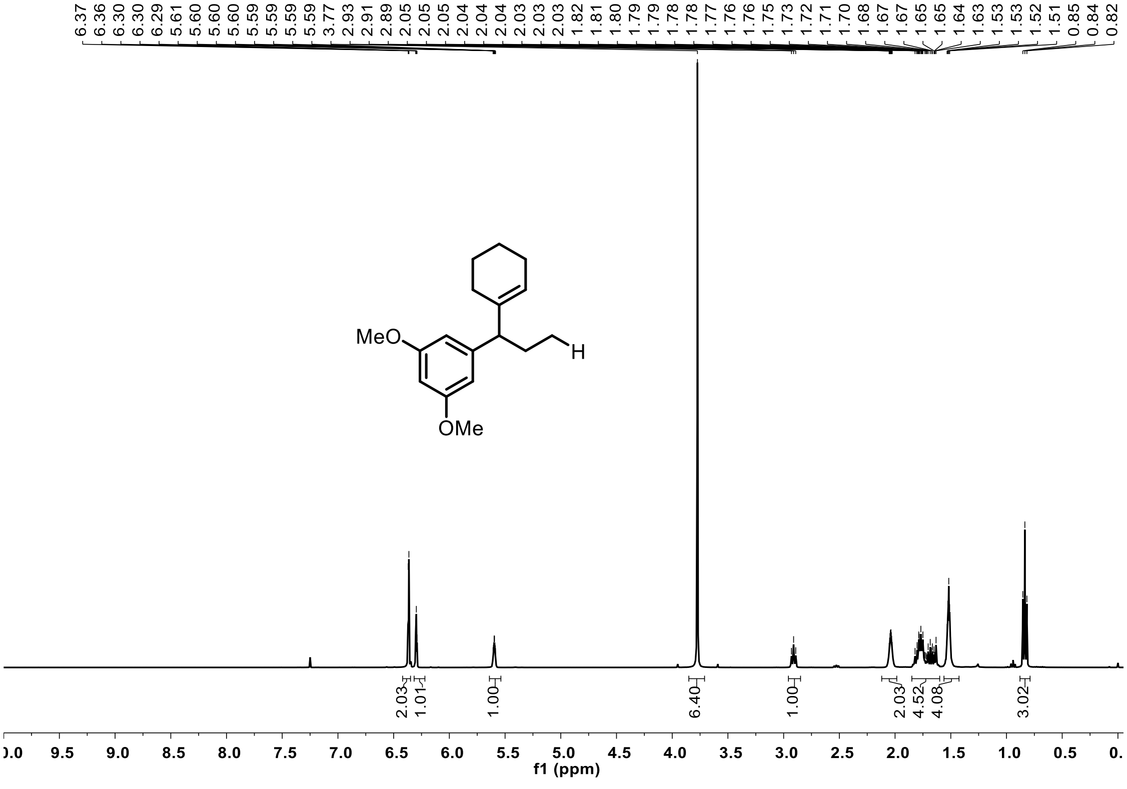

Supplementary Figure 116. ^{1}H NMR Spectrum of 3m

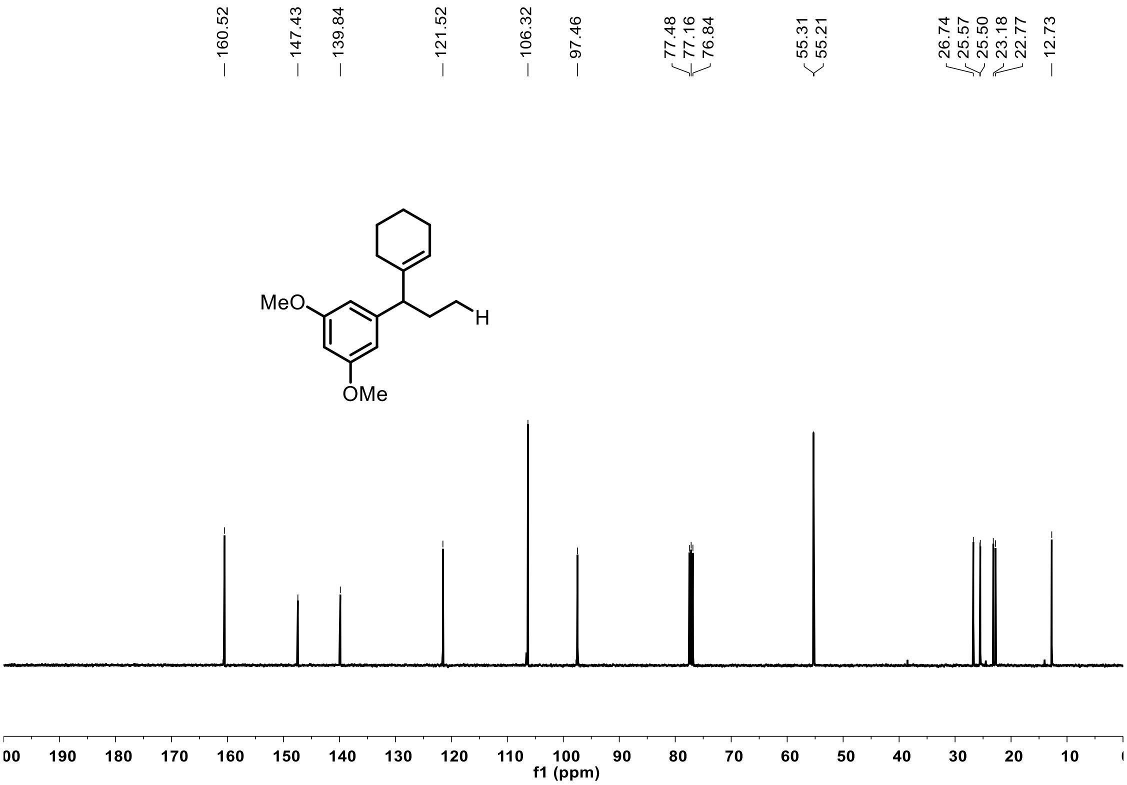

Supplementary Figure 117. ^{13}C NMR Spectrum of 3m

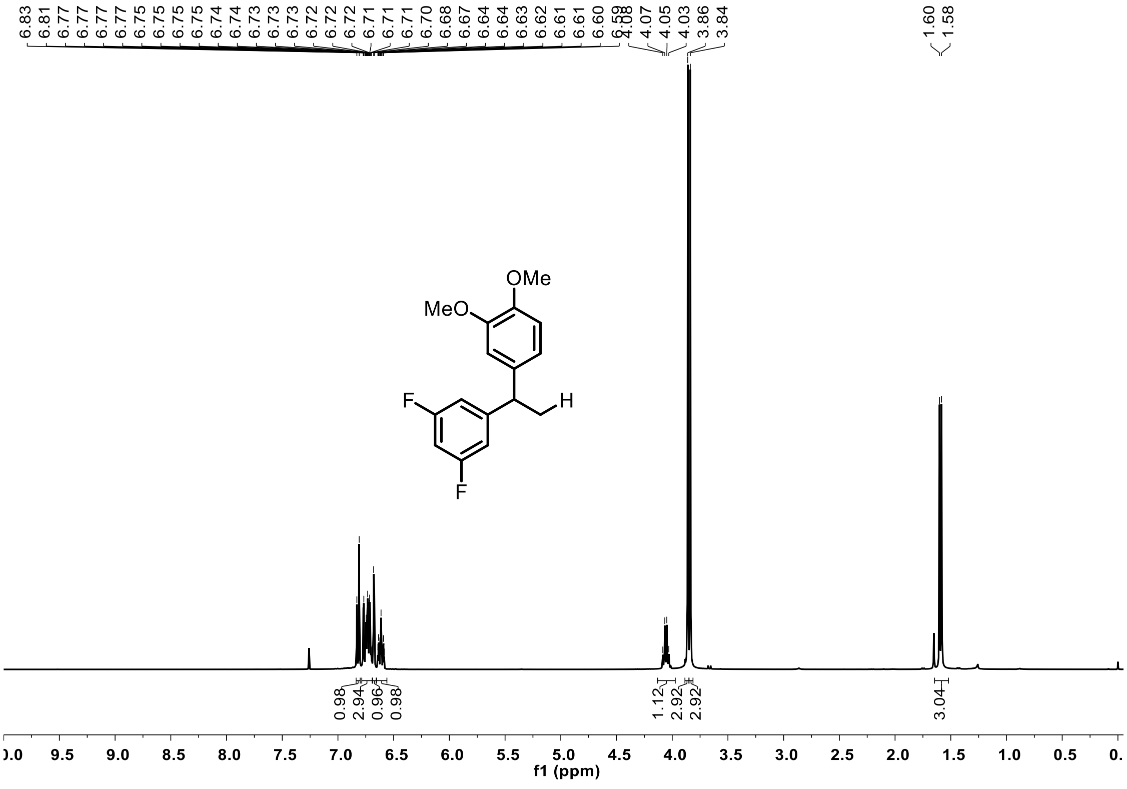

Supplementary Figure 118. ^{1}H NMR Spectrum of 3n

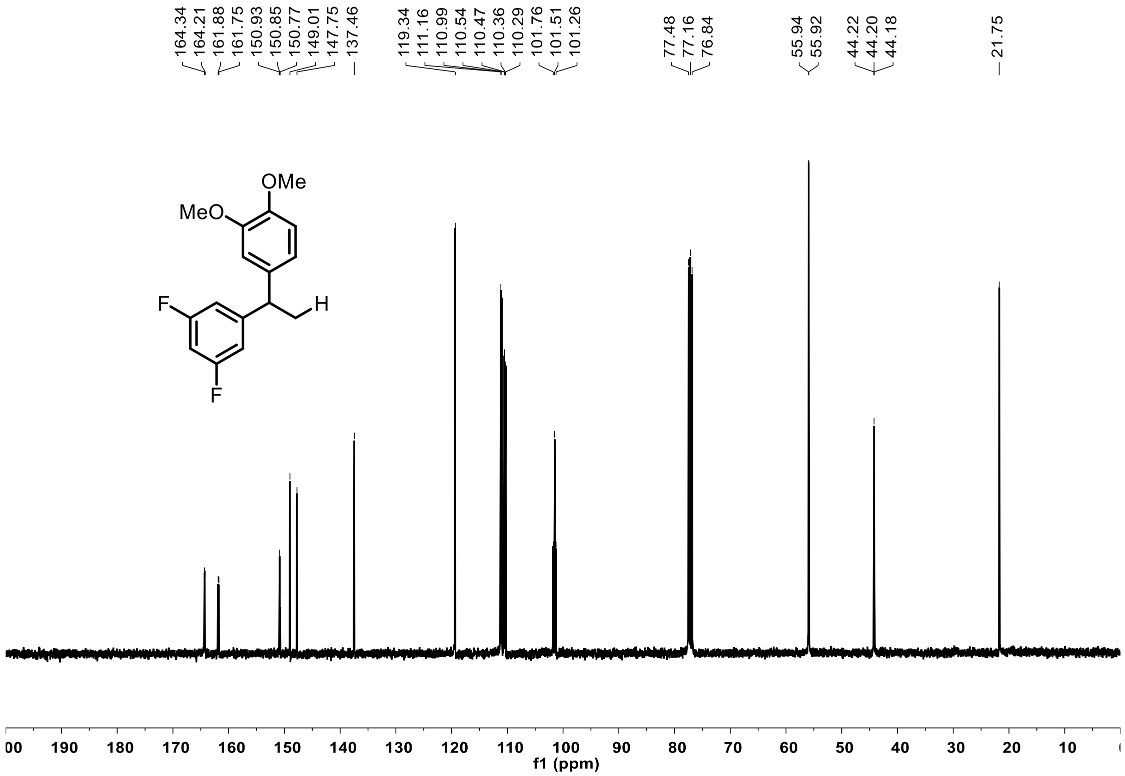

Supplementary Figure 119. ^{13}C NMR Spectrum of 3n

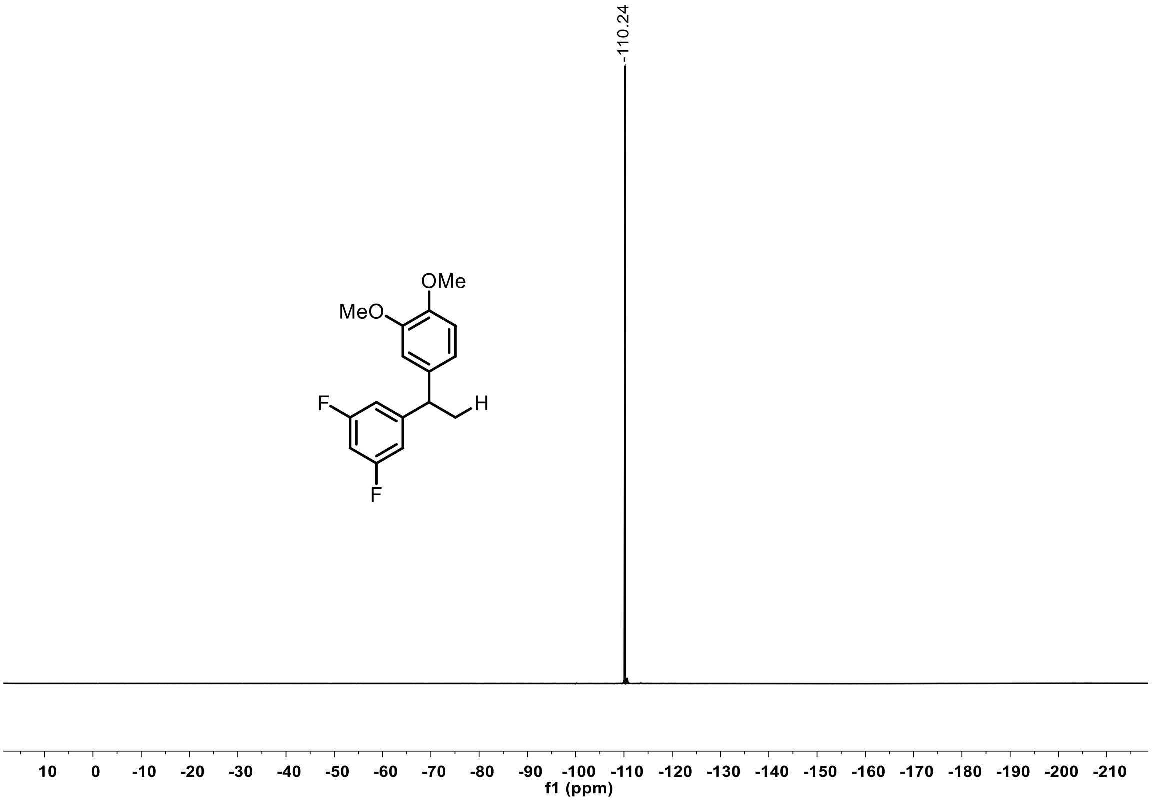

Supplementary Figure 120. ^{19}F NMR Spectrum of 3n

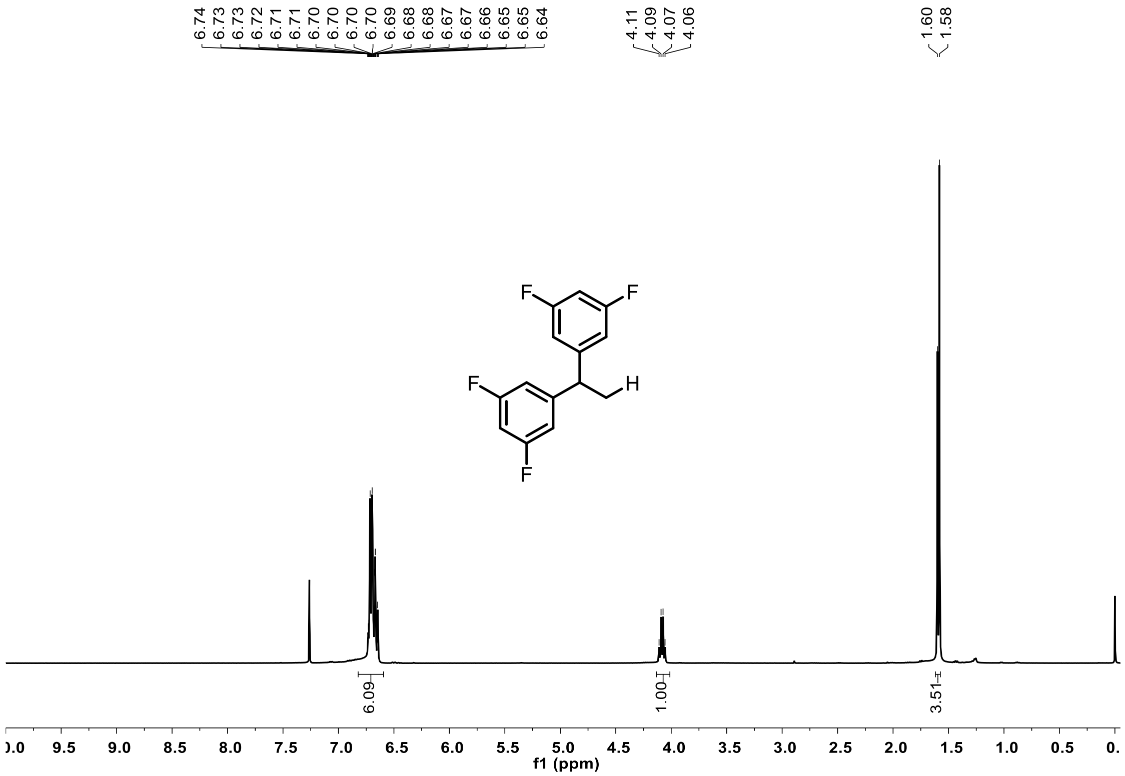

Supplementary Figure 121. ^{1}H NMR Spectrum of 3o

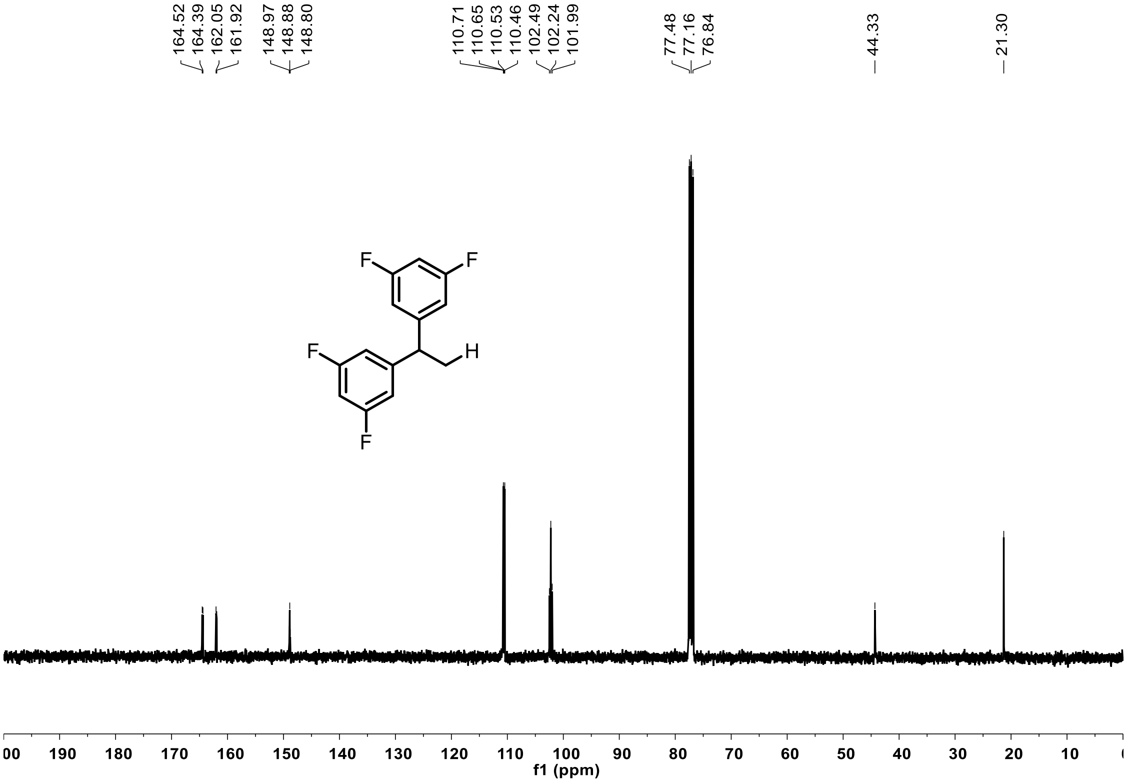

Supplementary Figure 122. ^{13}C NMR Spectrum of 3o

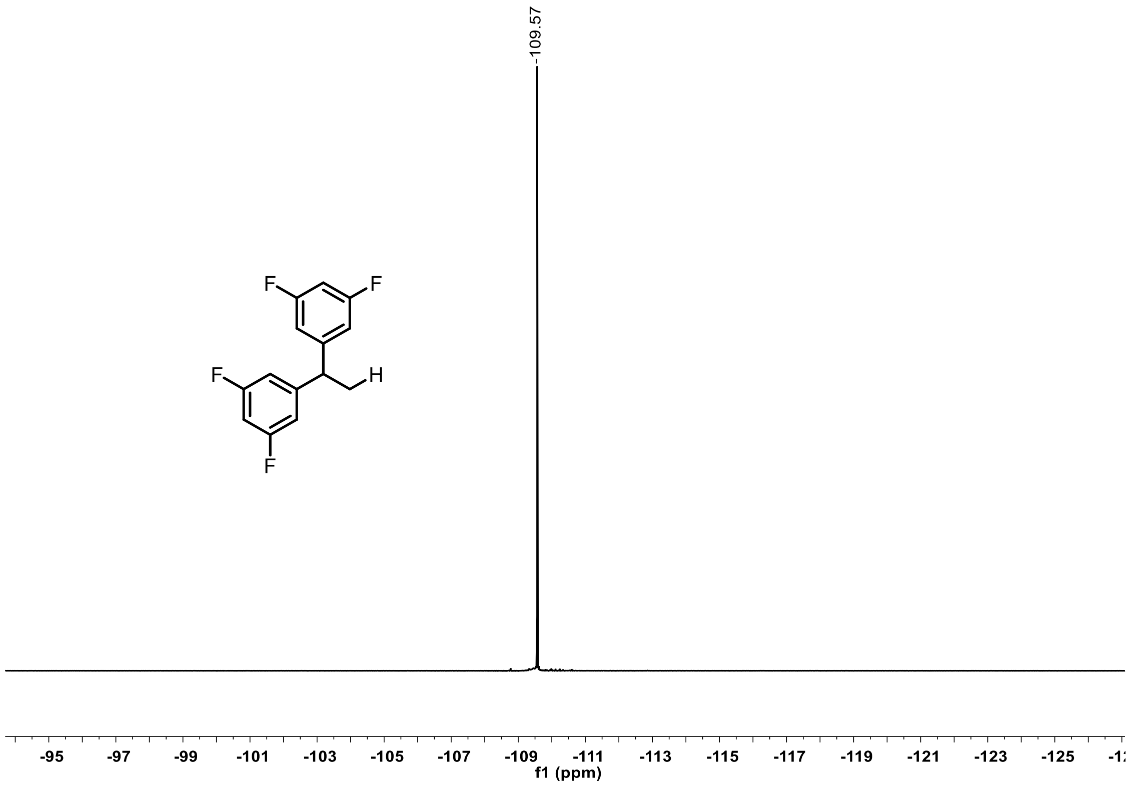

Supplementary Figure 123. ^{19}F NMR Spectrum of 3o

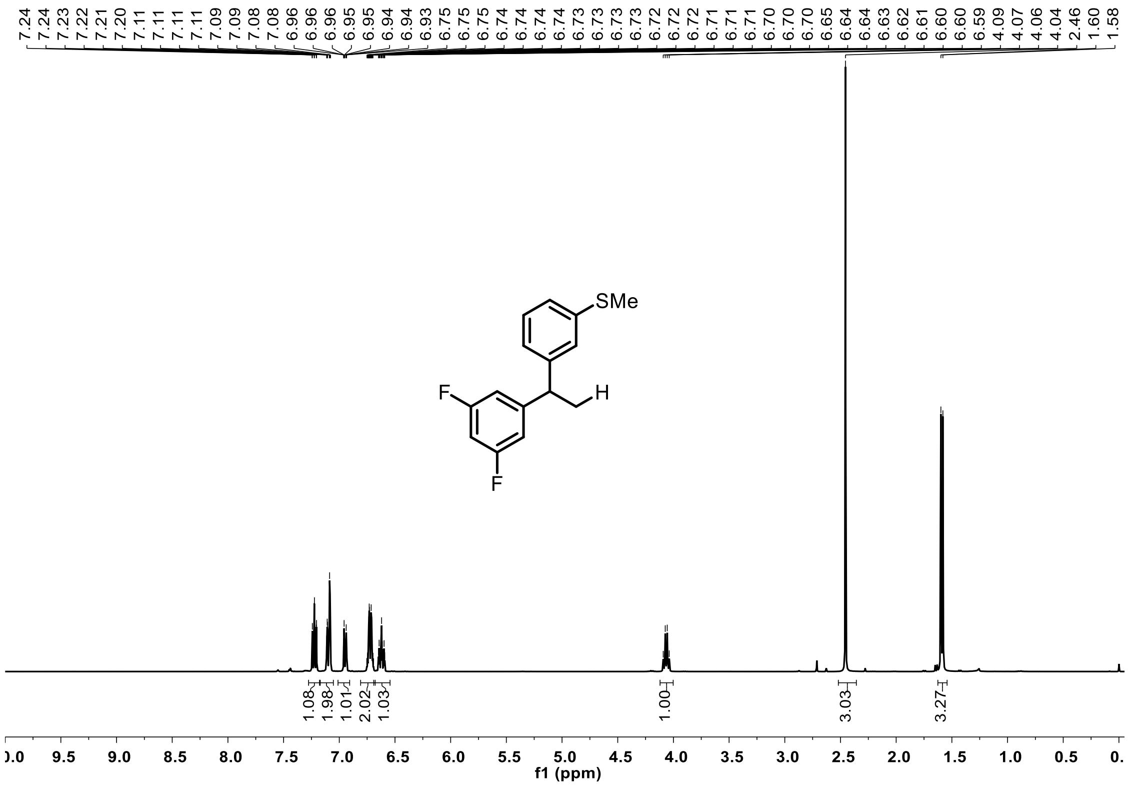

Supplementary Figure 124. ^{1}H NMR Spectrum of 3p

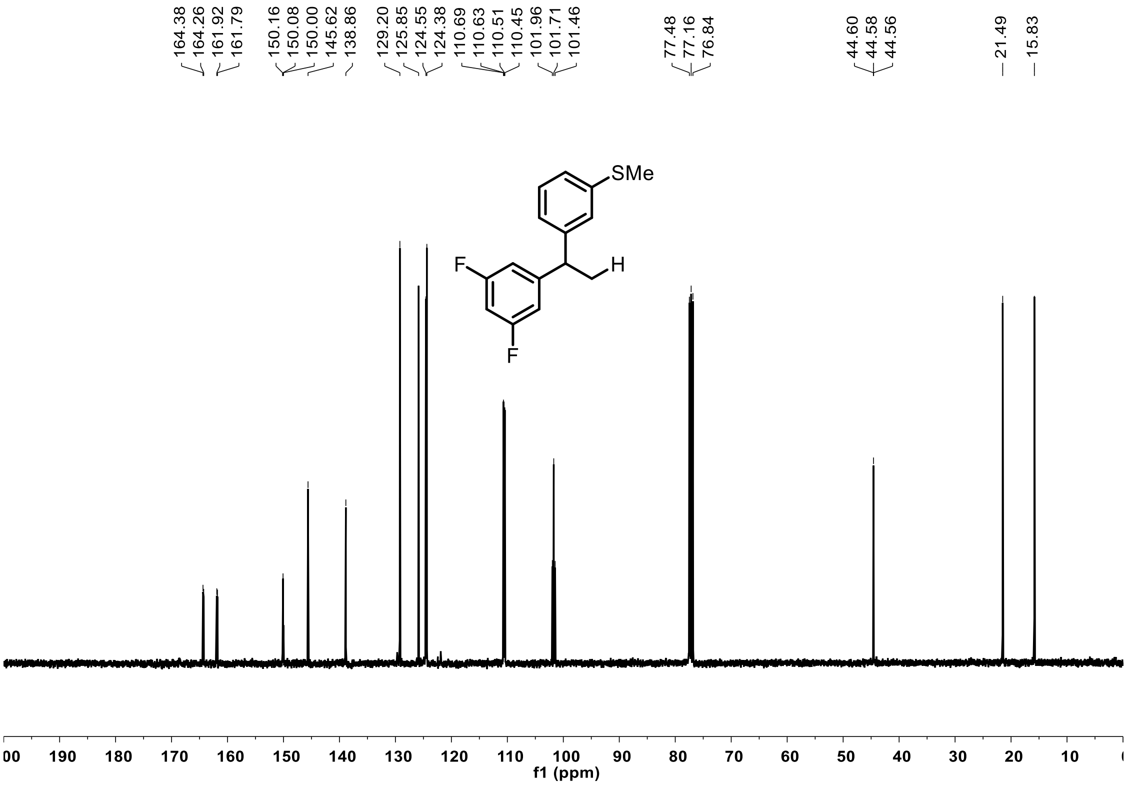

Supplementary Figure 125. ^{13}C NMR Spectrum of 3p

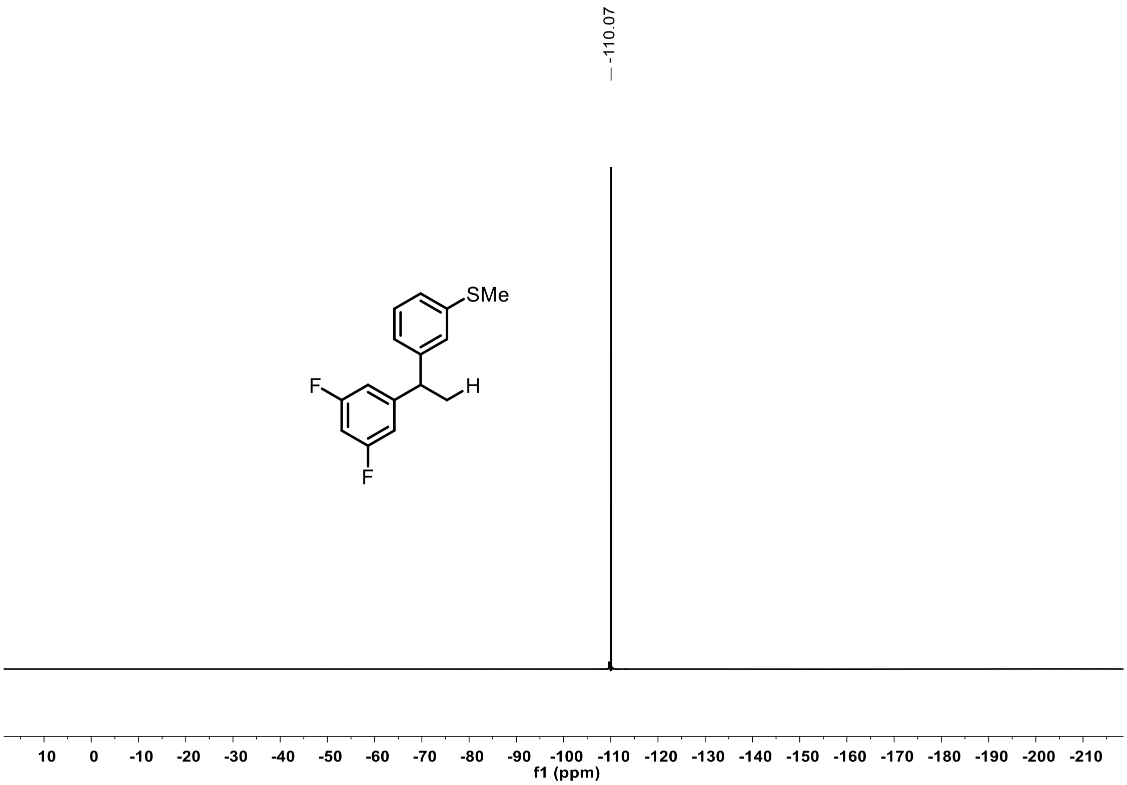

Supplementary Figure 126. ^{19}F NMR Spectrum of 3p

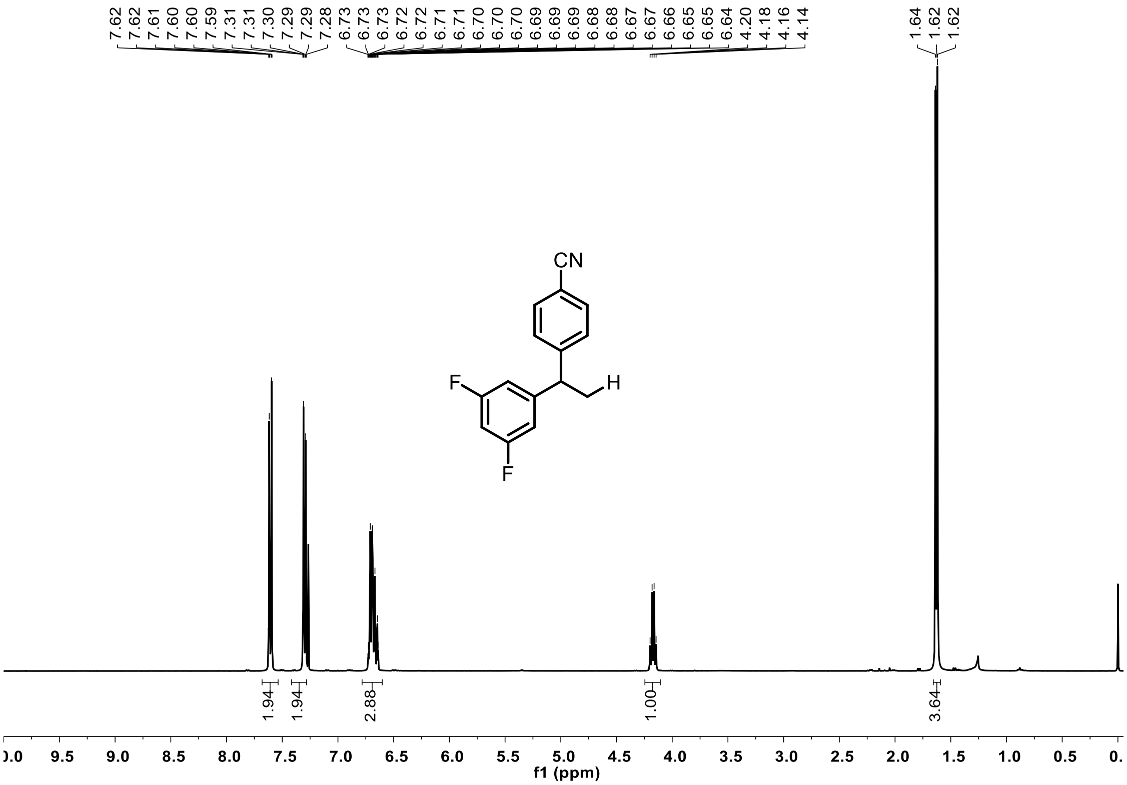

Supplementary Figure 127. ^{1}H NMR Spectrum of 3q

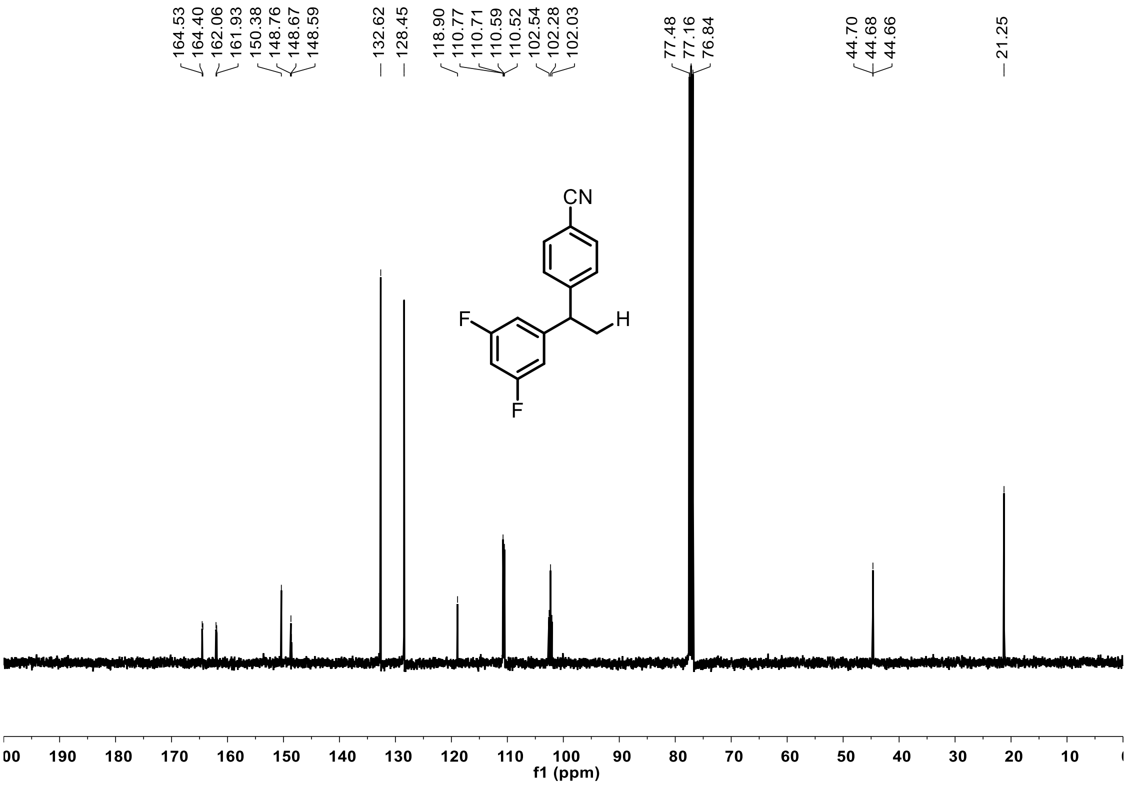

Supplementary Figure 128. ^{13}C NMR Spectrum of 3q

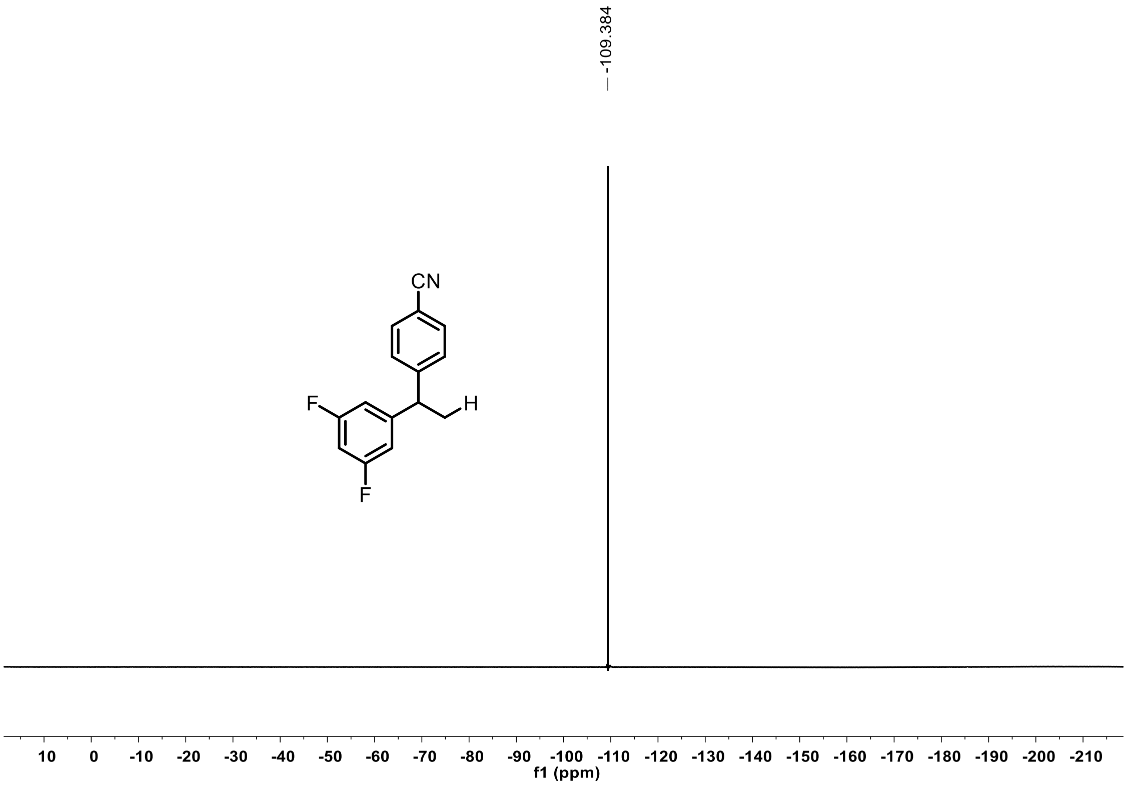

Supplementary Figure 129. ^{19}F NMR Spectrum of 3q

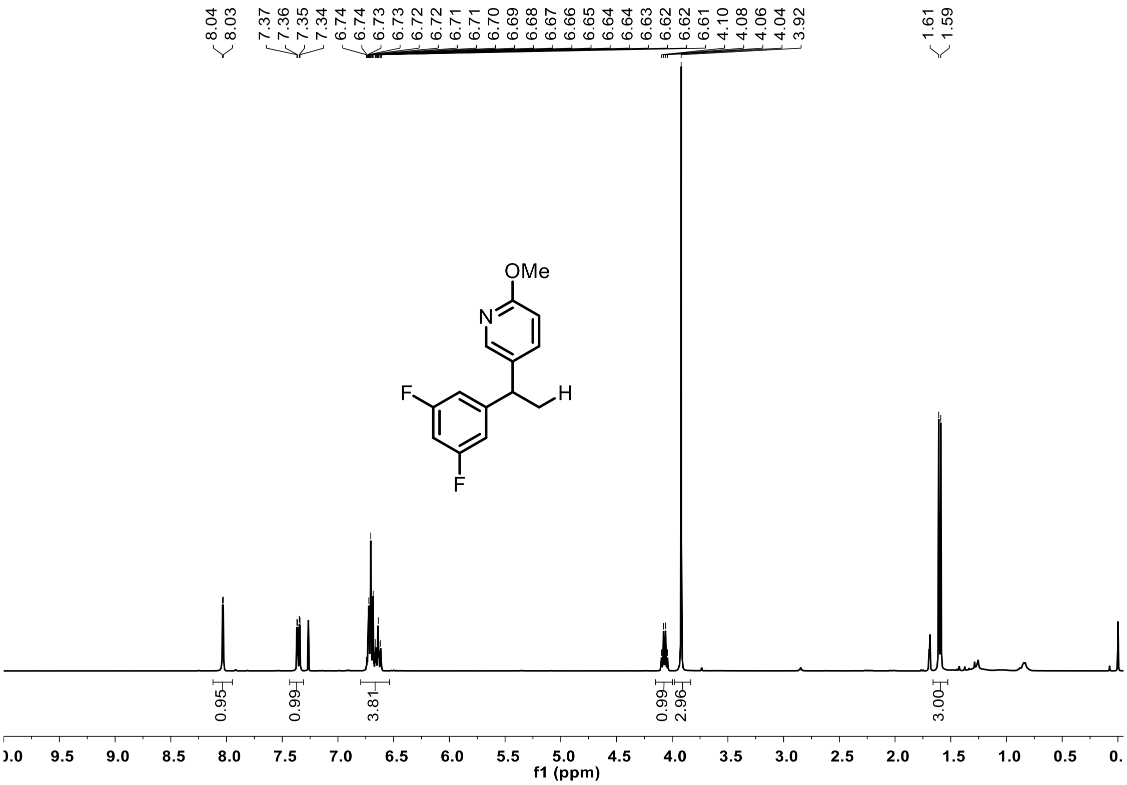

Supplementary Figure 130. ^{1}H NMR Spectrum of 3r

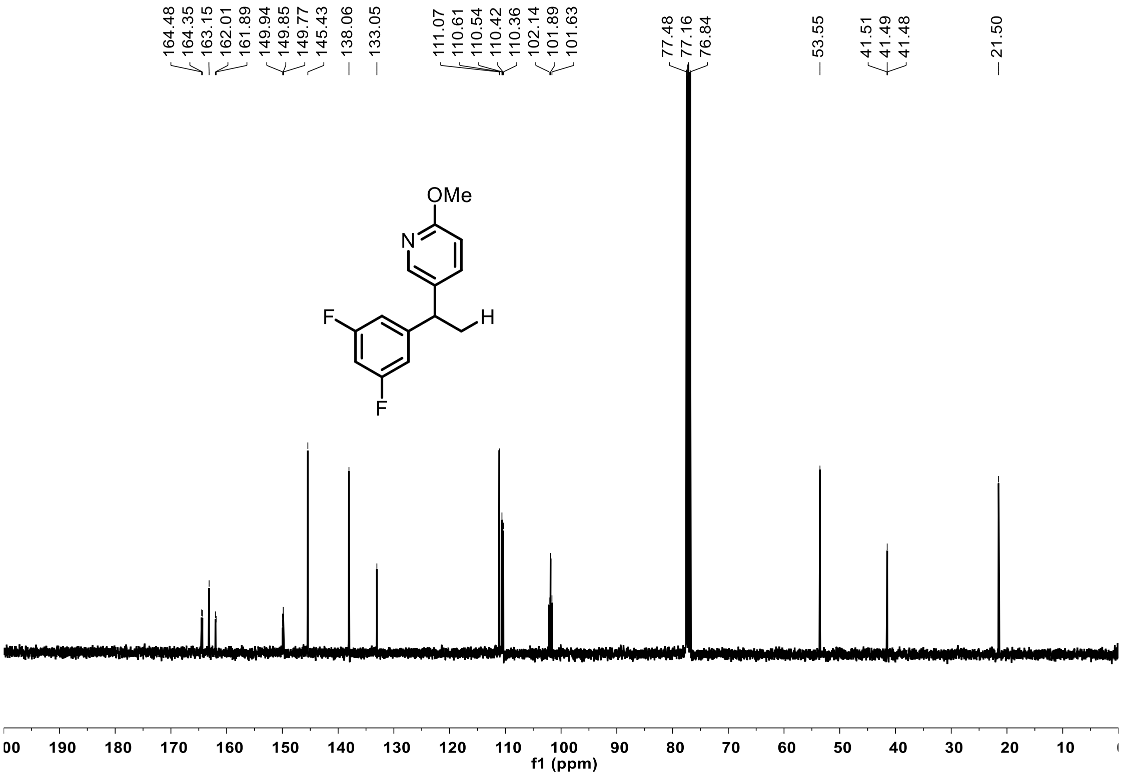

Supplementary Figure 131. ^{13}C NMR Spectrum of 3**r**

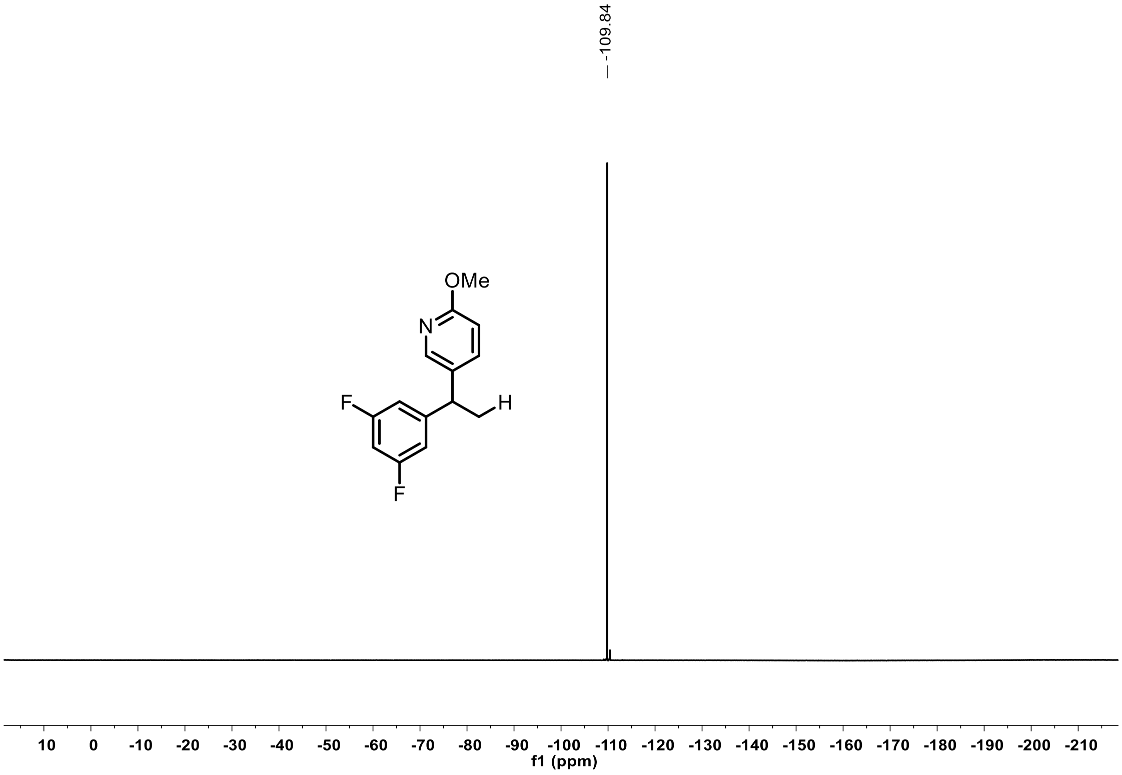

Supplementary Figure 132. ^{19}F NMR Spectrum of 3r

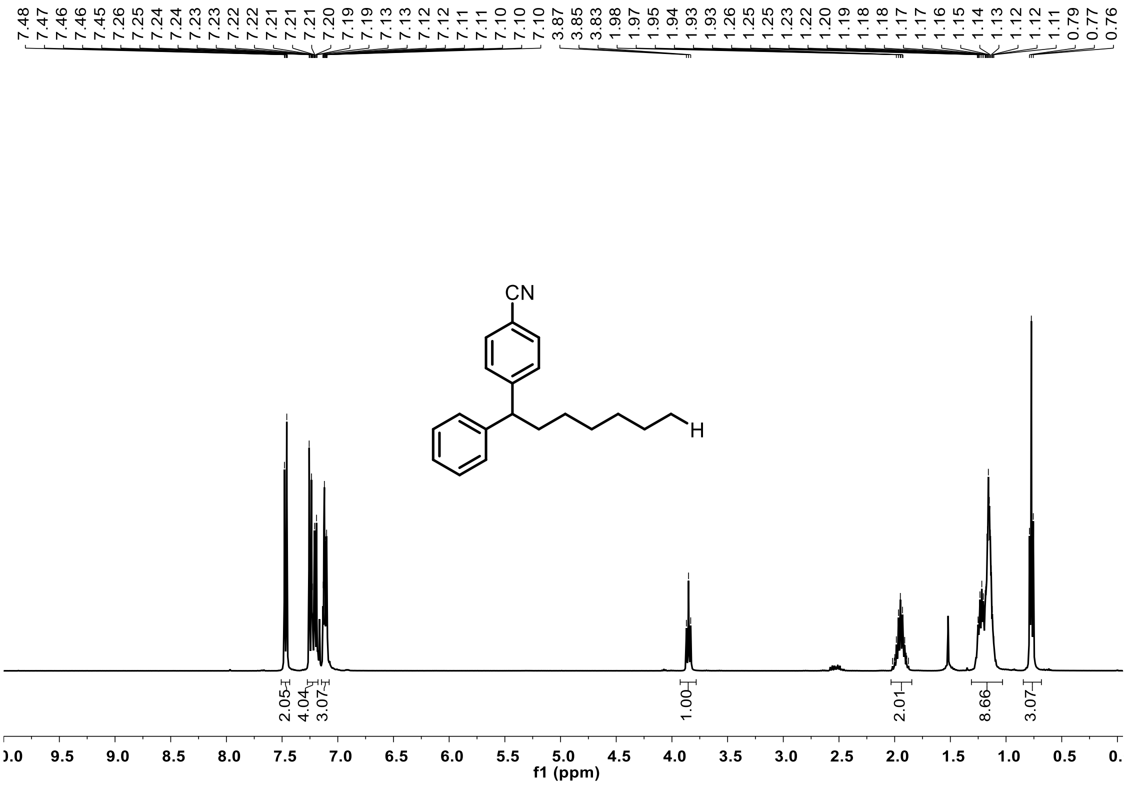

Supplementary Figure 133. ^{1}H NMR Spectrum of 3s

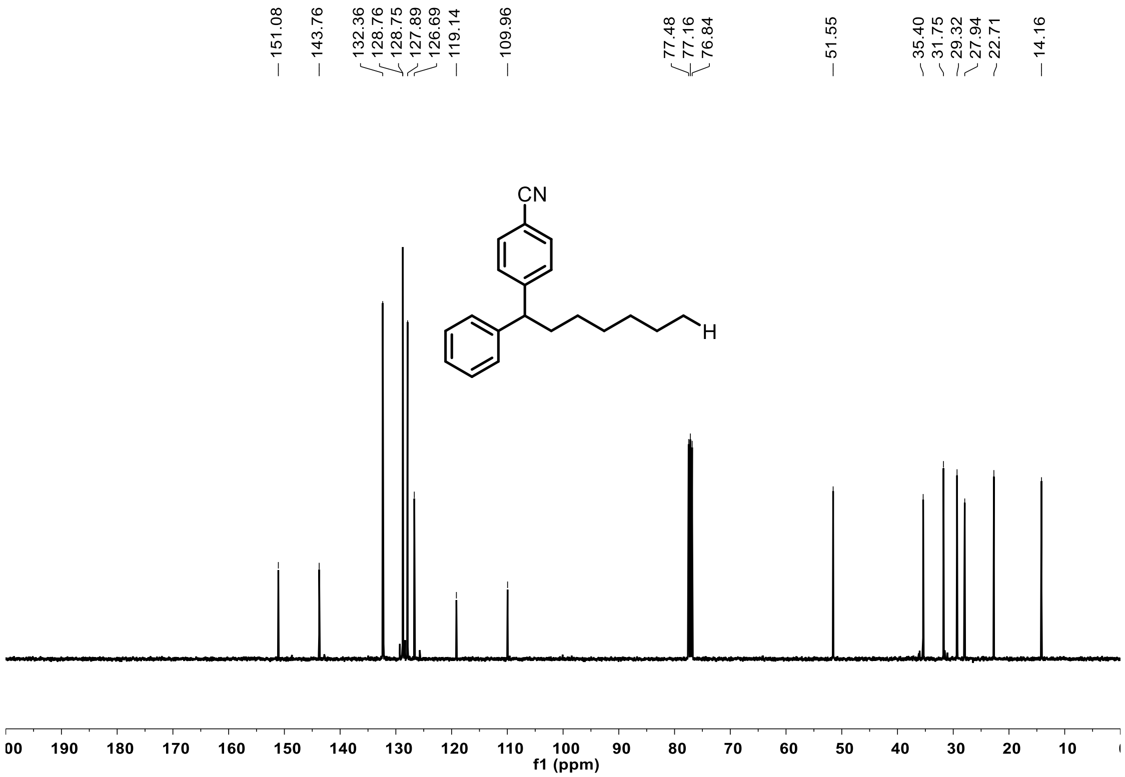

Supplementary Figure 134. ^{13}C NMR Spectrum of 3s

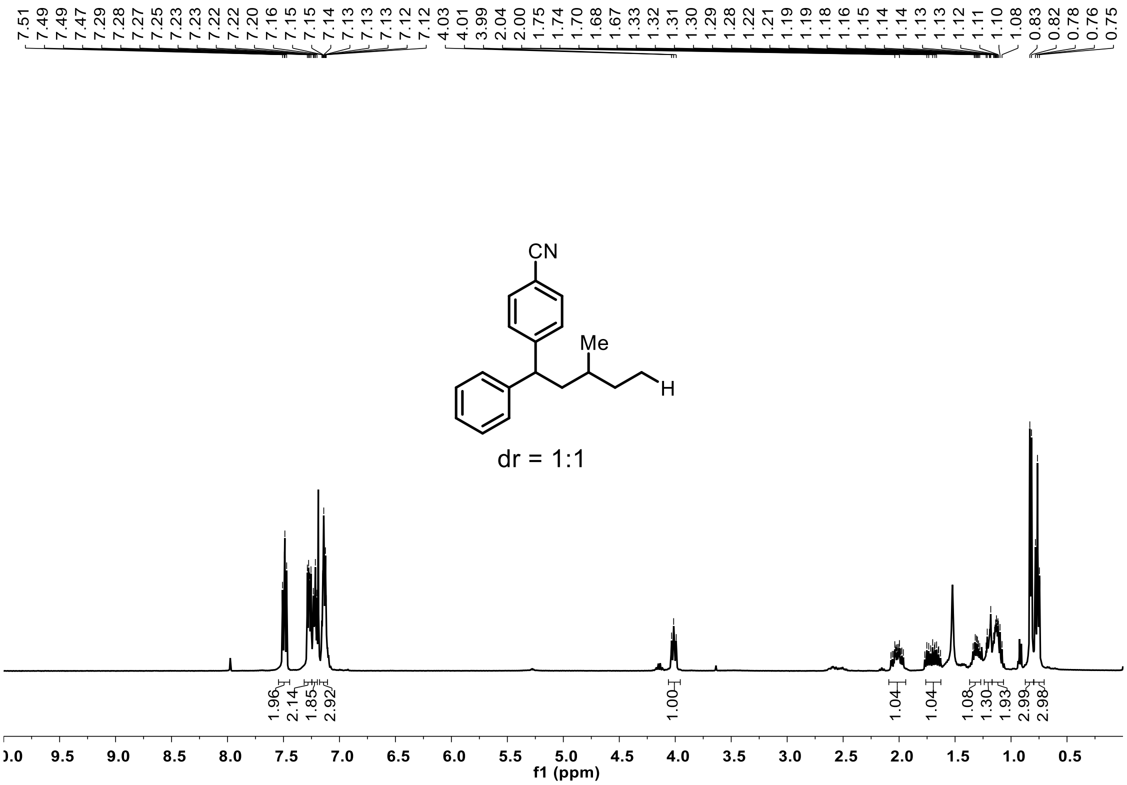

Supplementary Figure 135. ^{1}H NMR Spectrum of 3t

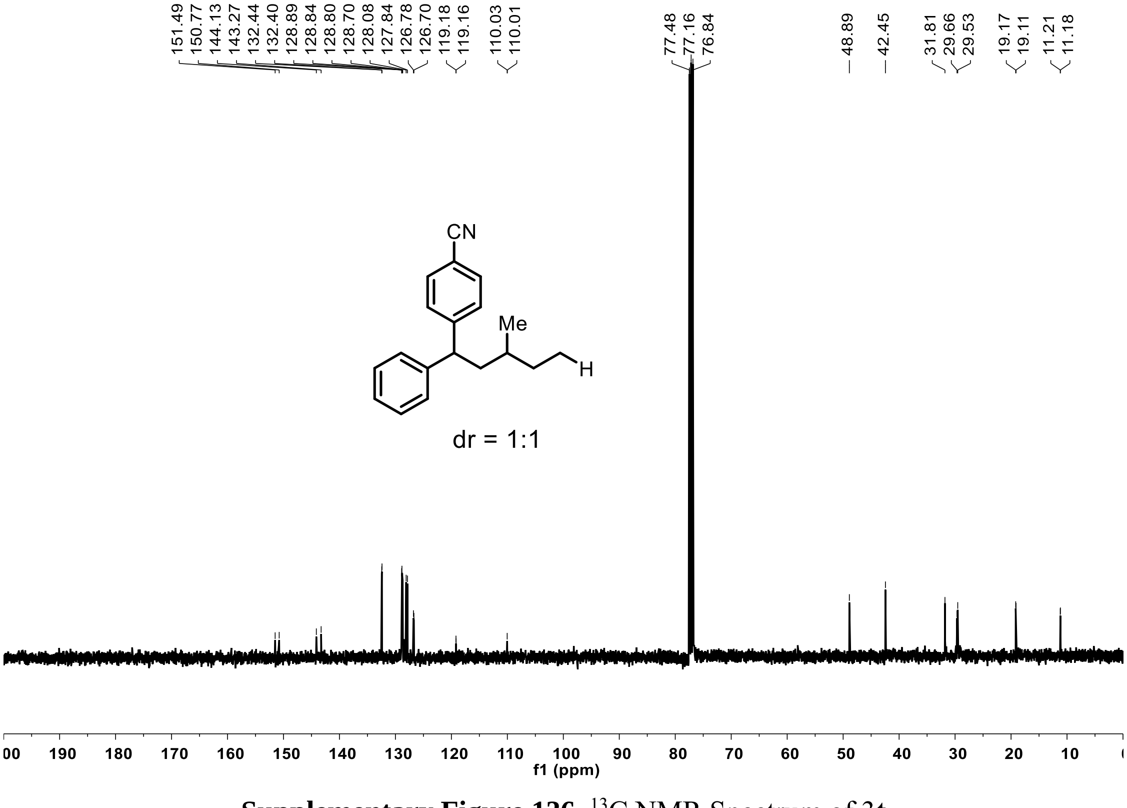

Supplementary Figure 136. ^{13}C NMR Spectrum of 3**t**

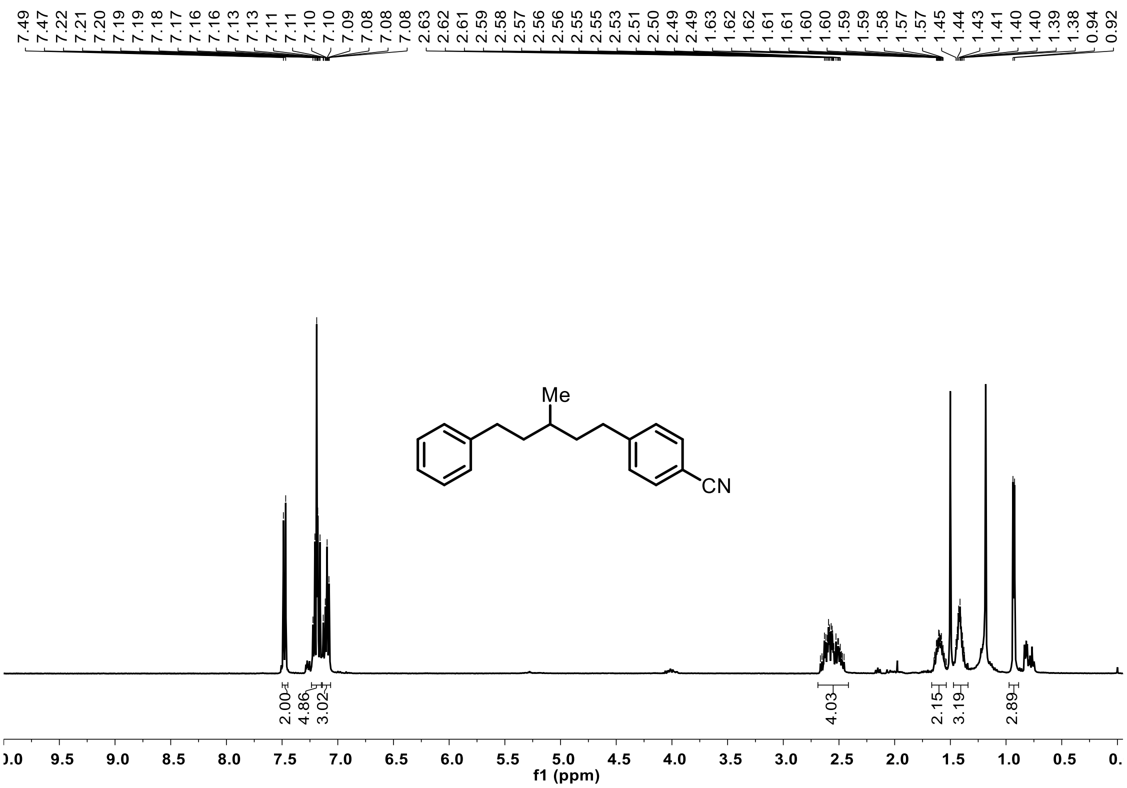

Supplementary Figure 137. ^{1}H NMR Spectrum of 4t

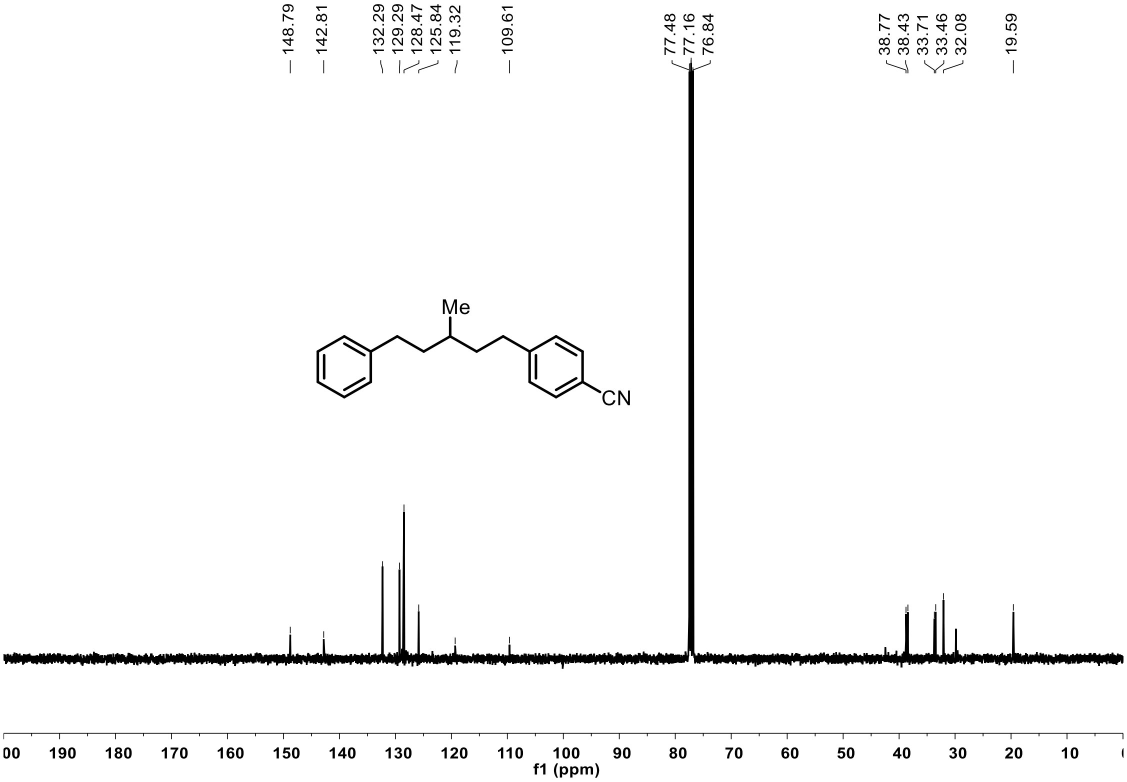

Supplementary Figure 138. ^{13}C NMR Spectrum of 4**t**

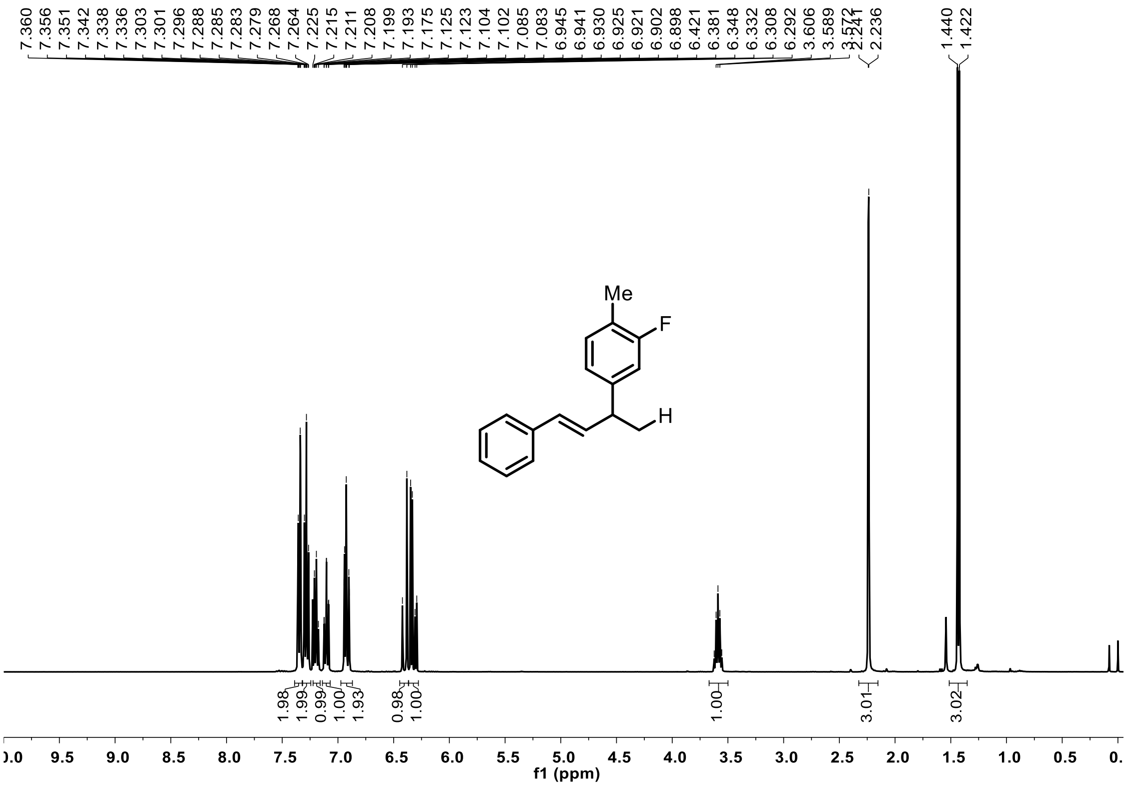

Supplementary Figure 139. ^{1}H NMR Spectrum of 3u

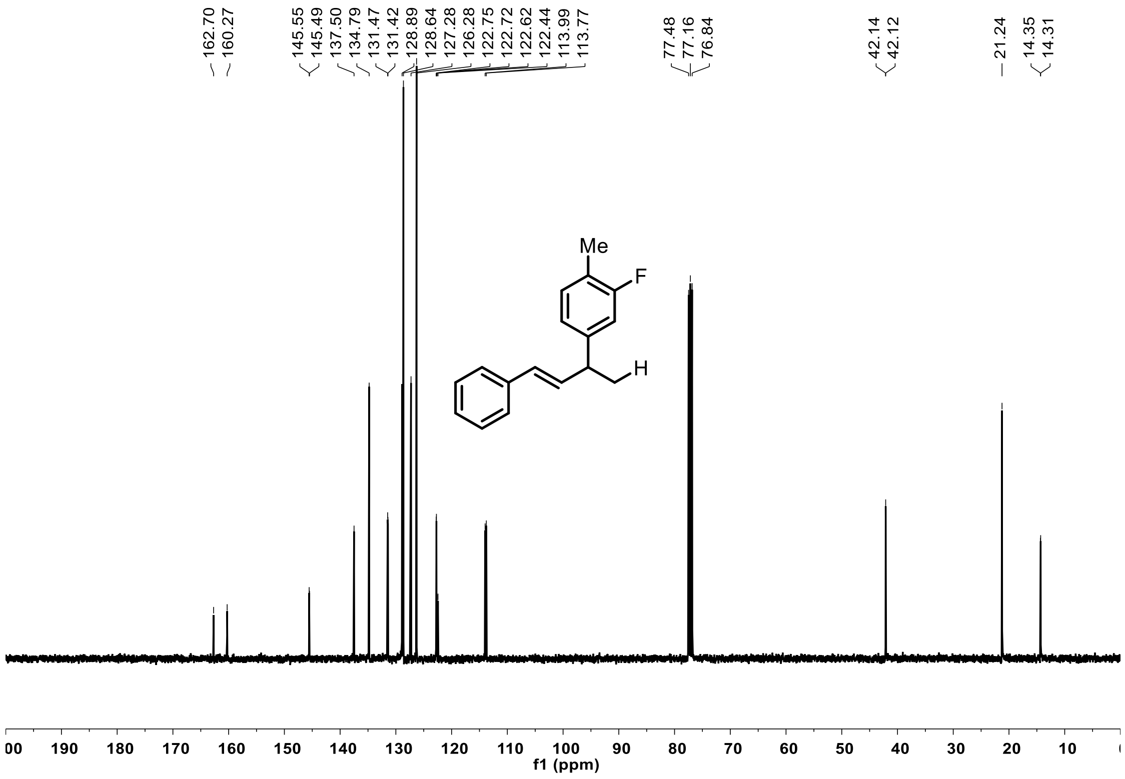

Supplementary Figure 140. ^{13}C NMR Spectrum of 3u

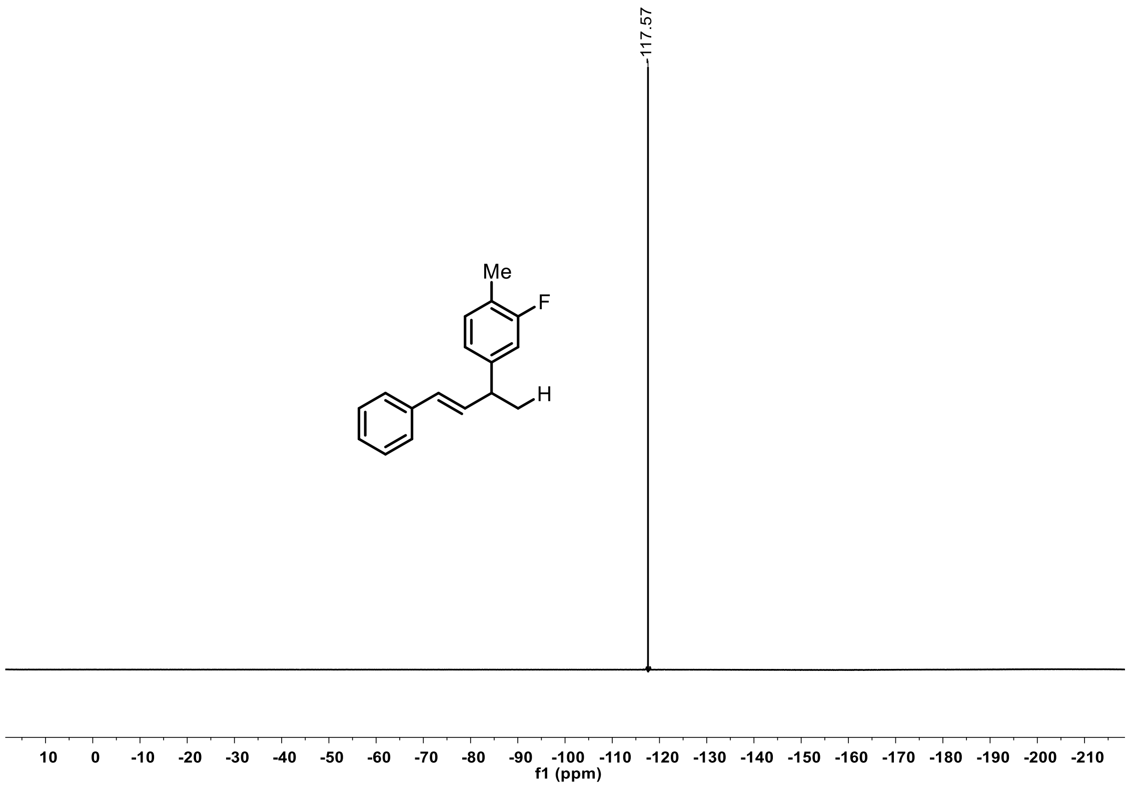

Supplementary Figure 141. ^{19}F NMR Spectrum of 3u

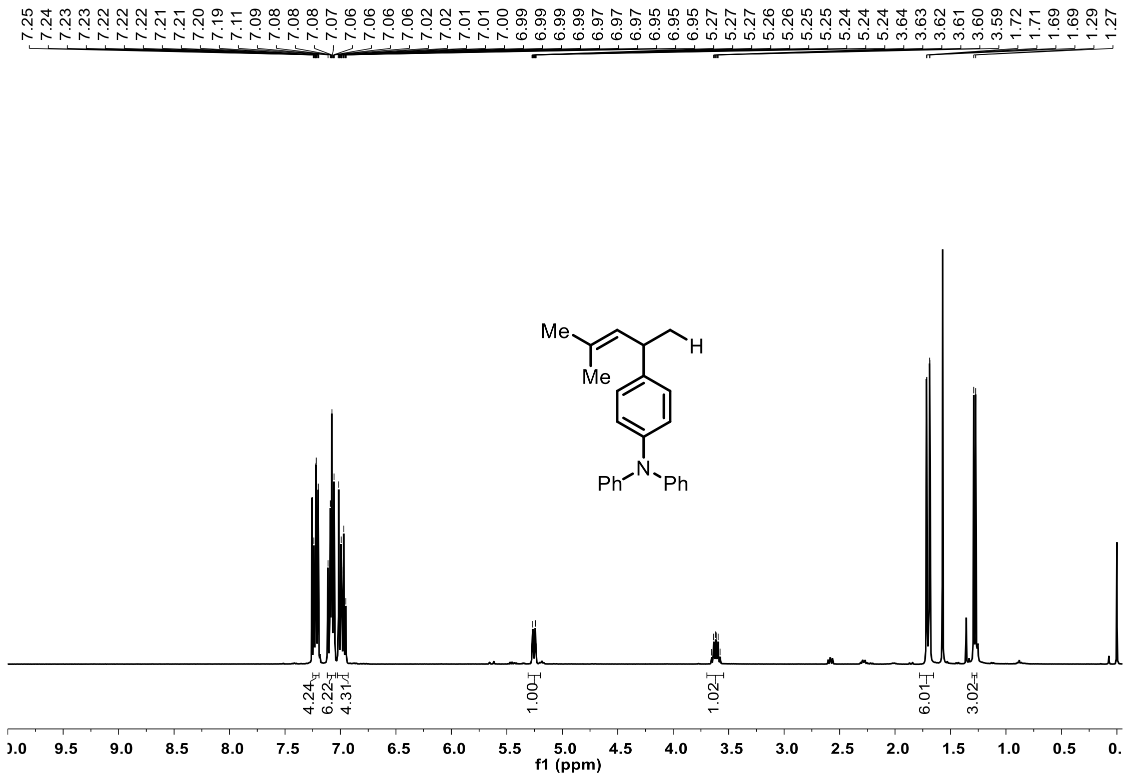

Supplementary Figure 142. ^{1}H NMR Spectrum of 3v

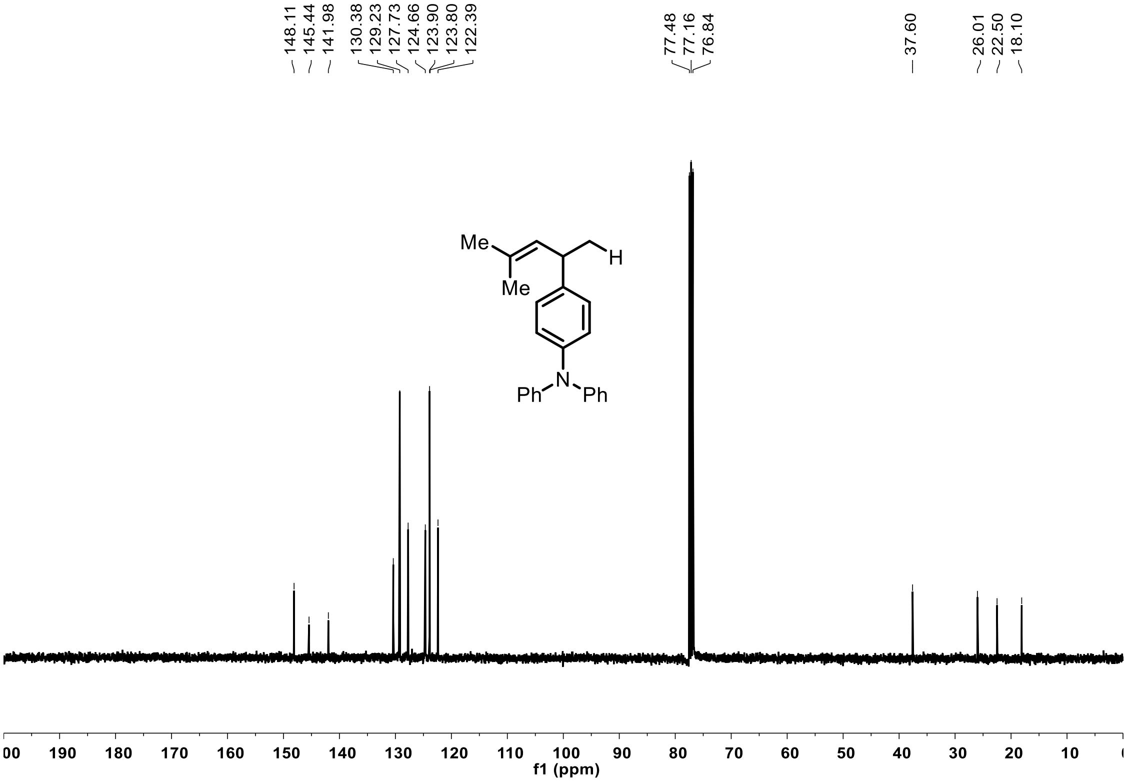

Supplementary Figure 143. ^{13}C NMR Spectrum of 3v

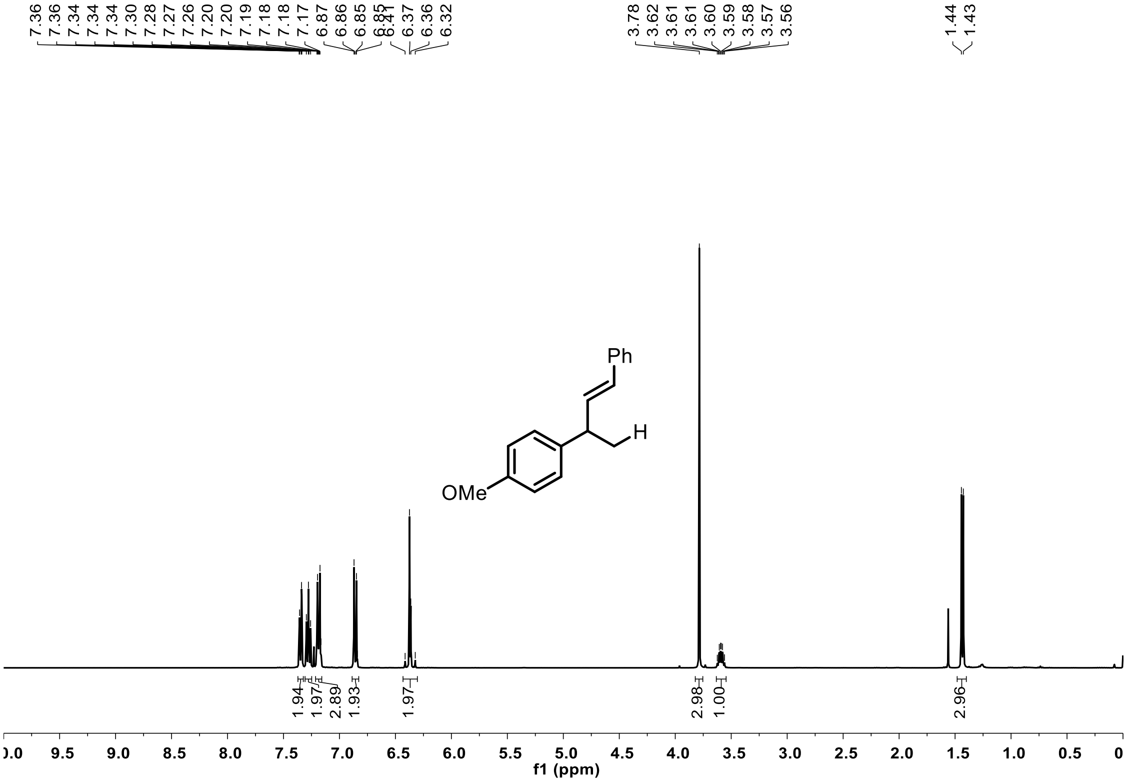

Supplementary Figure 144. ^{1}H NMR Spectrum of 3w

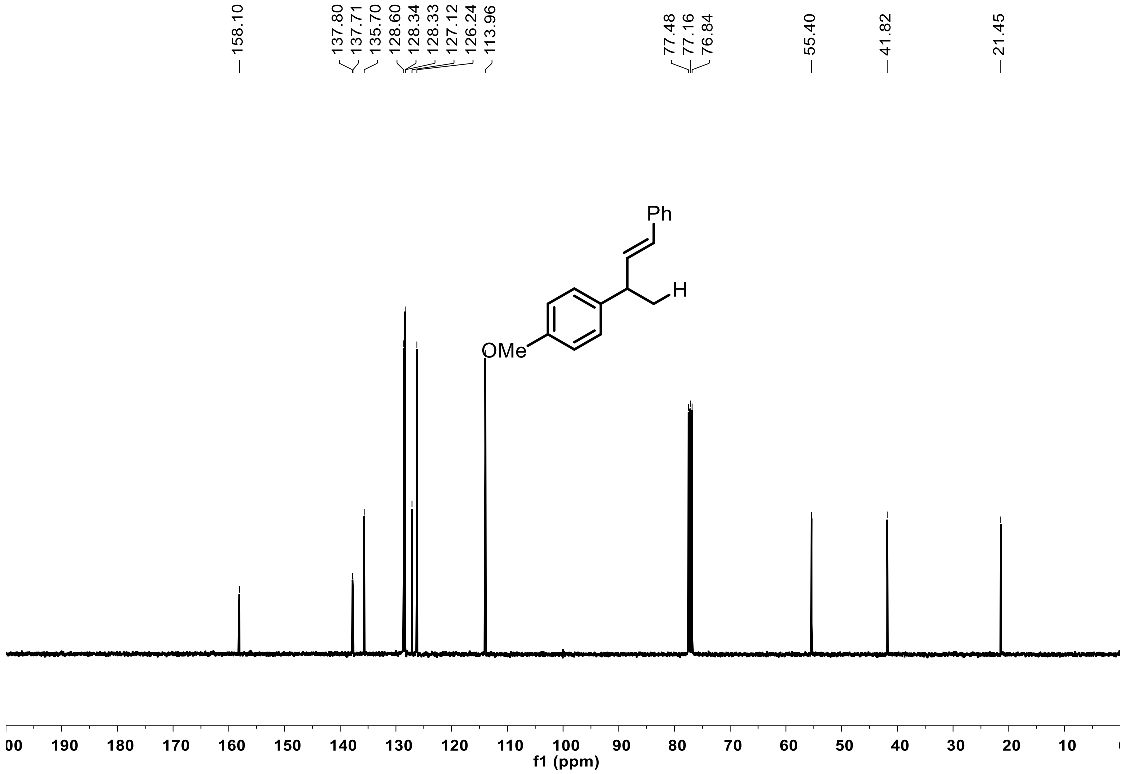

Supplementary Figure 145. ^{13}C NMR Spectrum of 3w

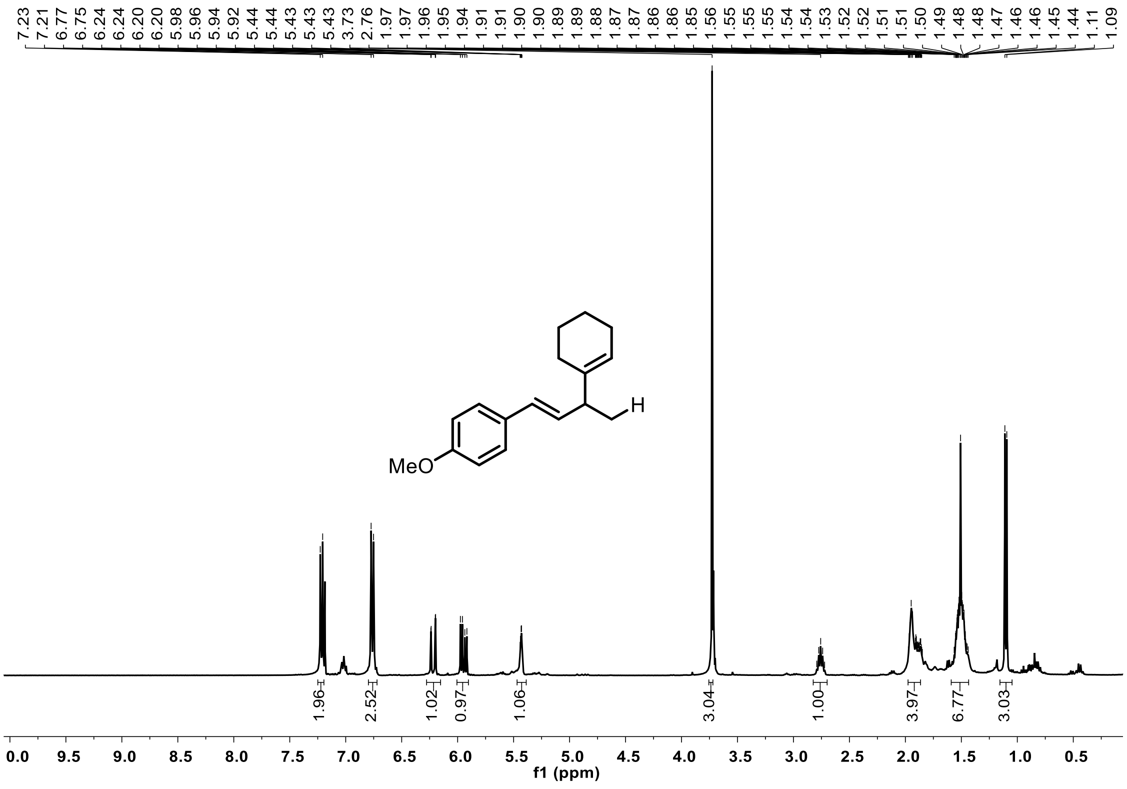

Supplementary Figure 146. ^{1}H NMR Spectrum of 3x

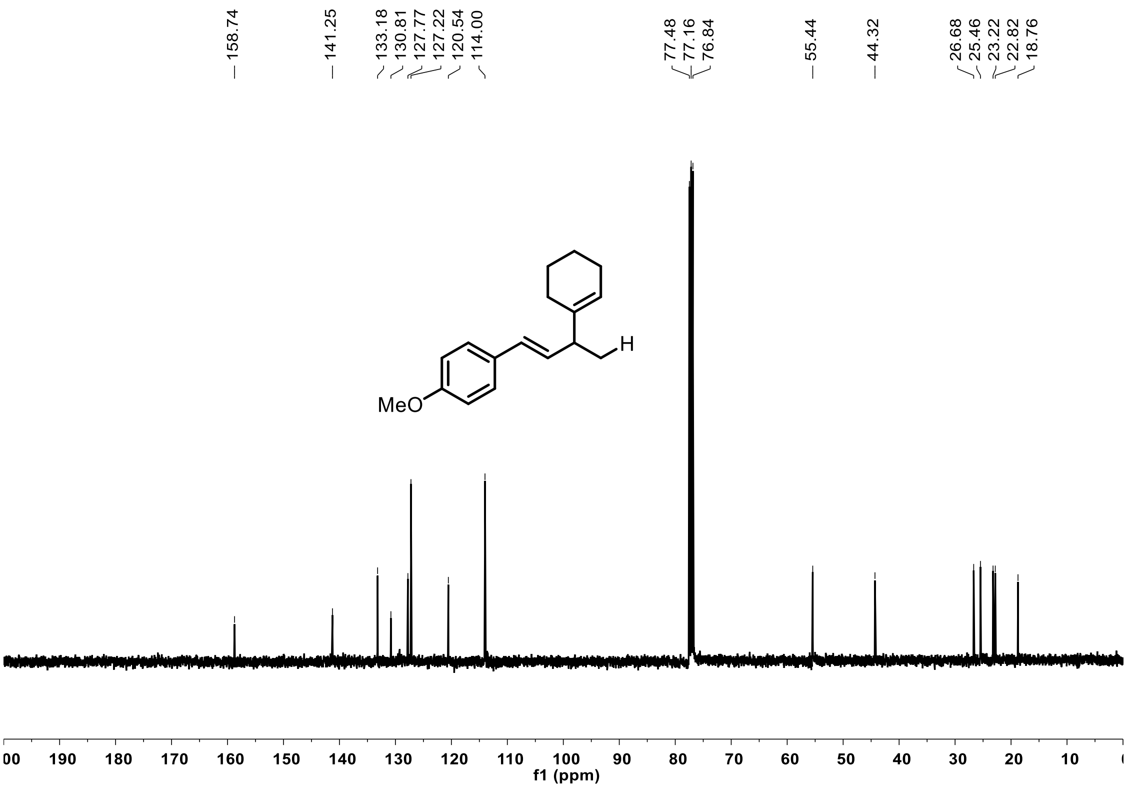

Supplementary Figure 147. ^{13}C NMR Spectrum of 3x

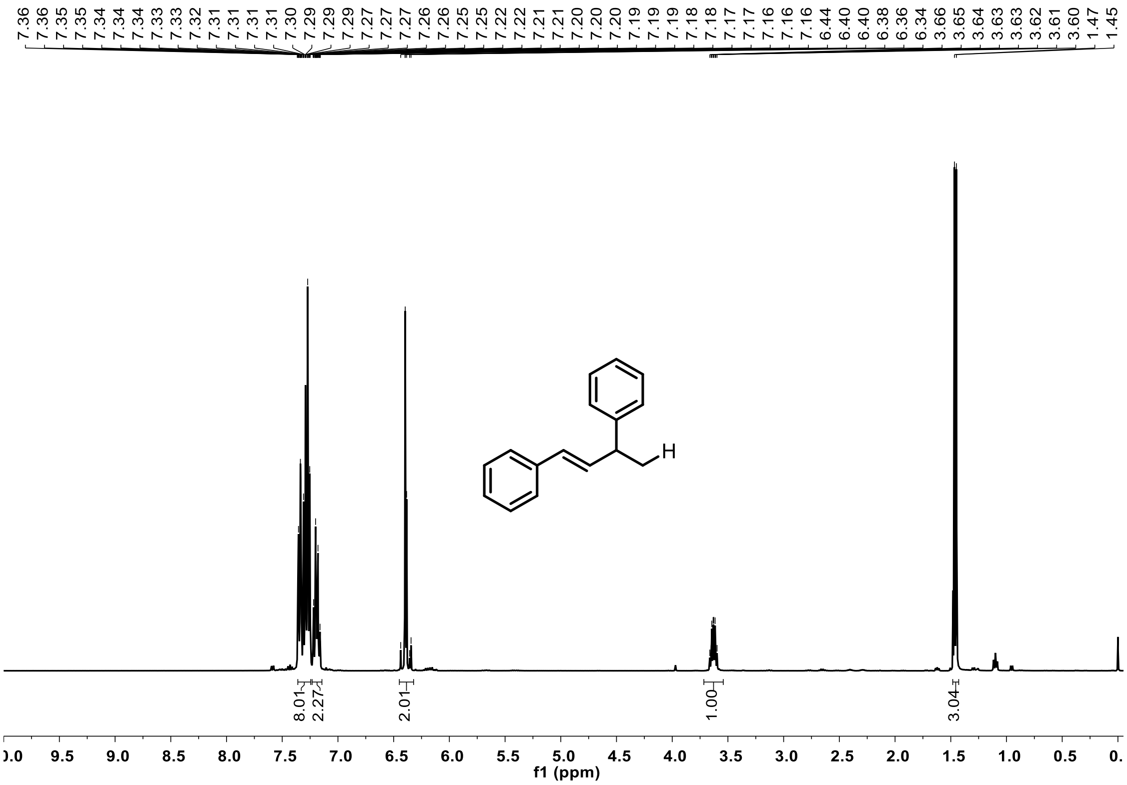

Supplementary Figure 148. ^{1}H NMR Spectrum of 3y

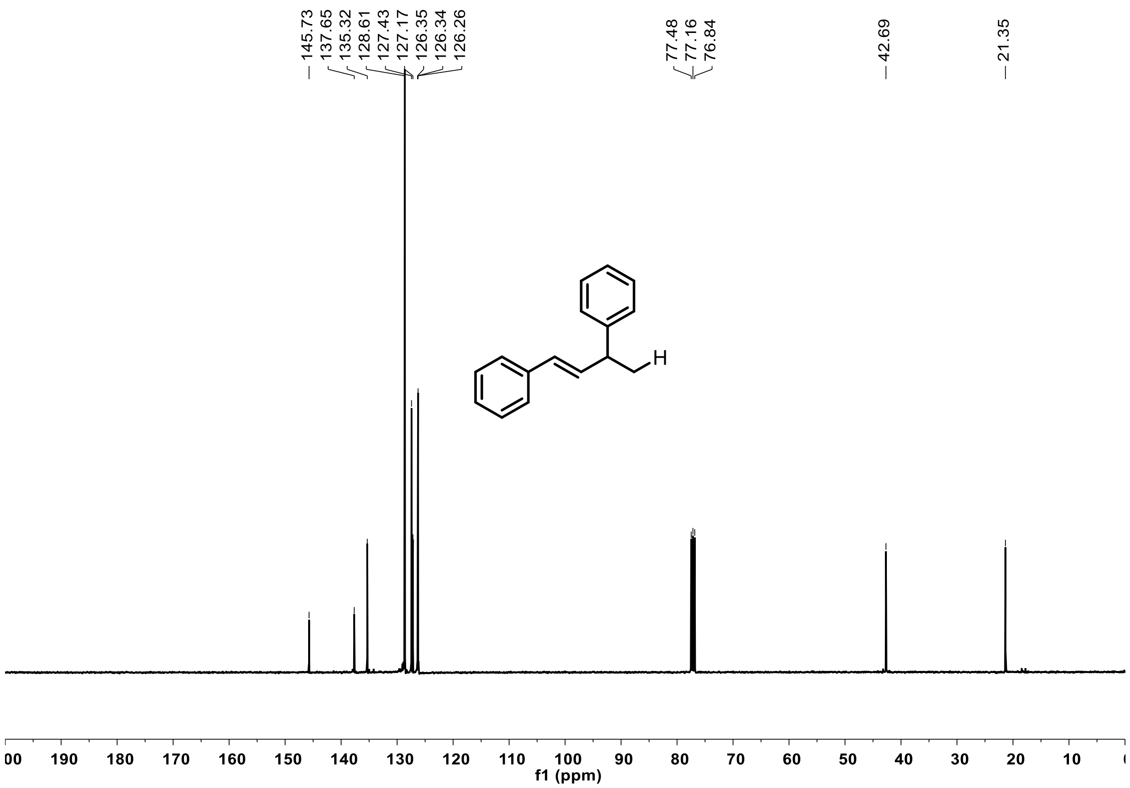

Supplementary Figure 149. ^{13}C NMR Spectrum of 3y

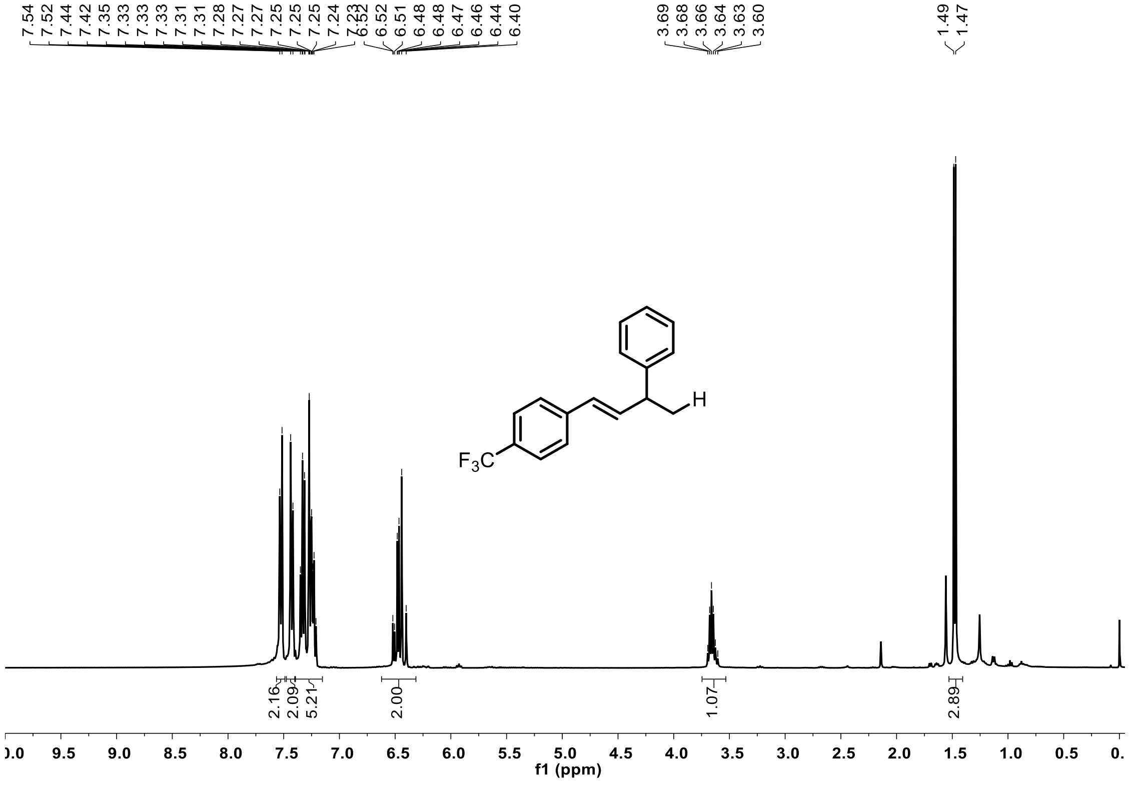

Supplementary Figure 150. ^{1}H NMR Spectrum of 3z

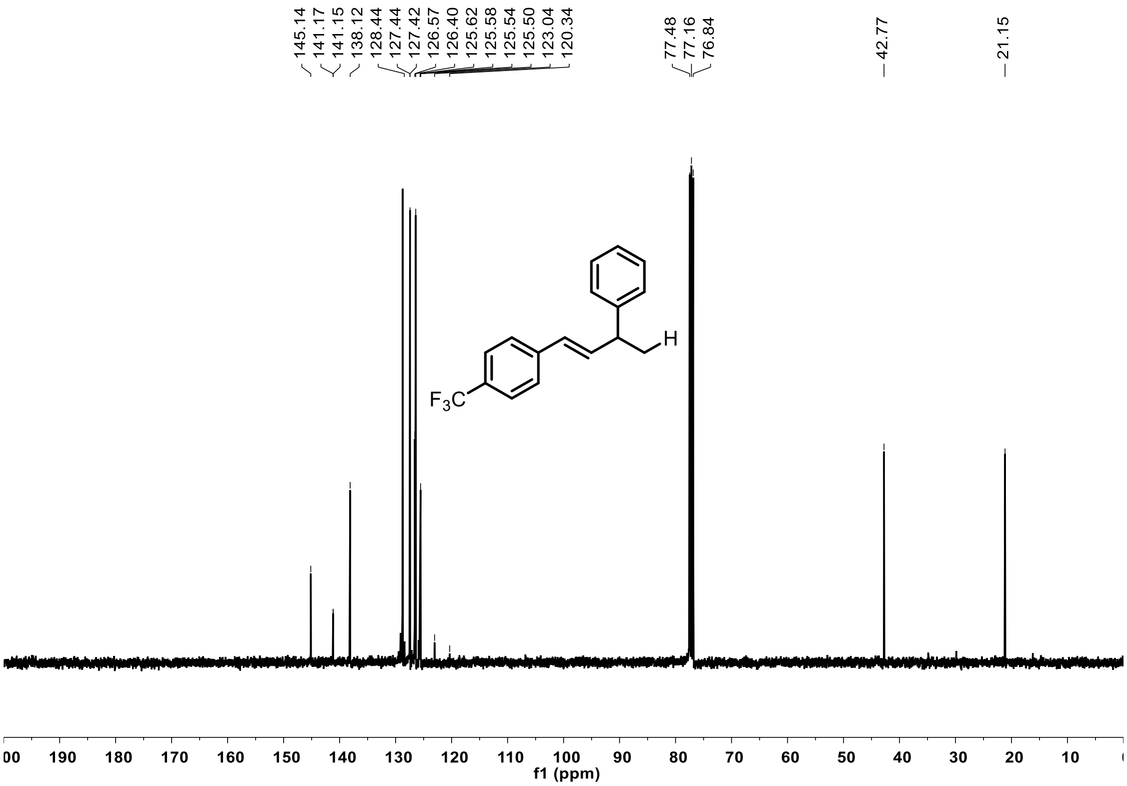

Supplementary Figure 151. ^{13}C NMR Spectrum of 3z

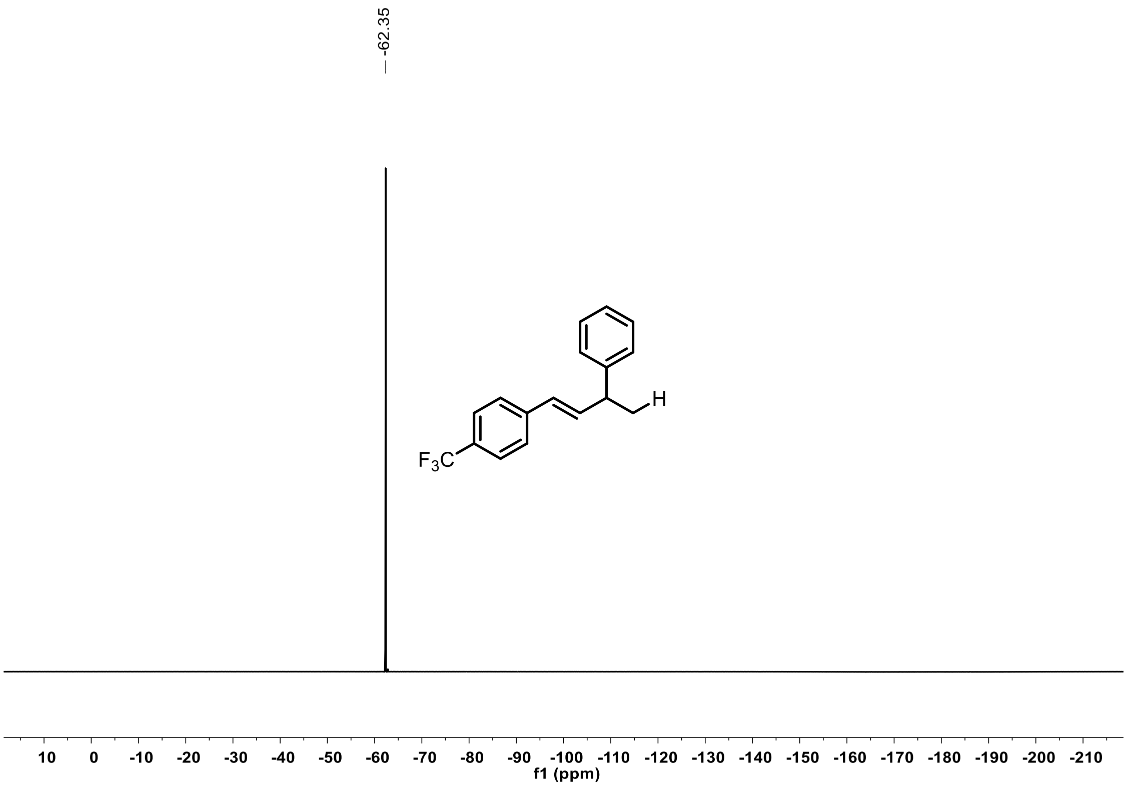

Supplementary Figure 152. ^{19}F NMR Spectrum of 3z

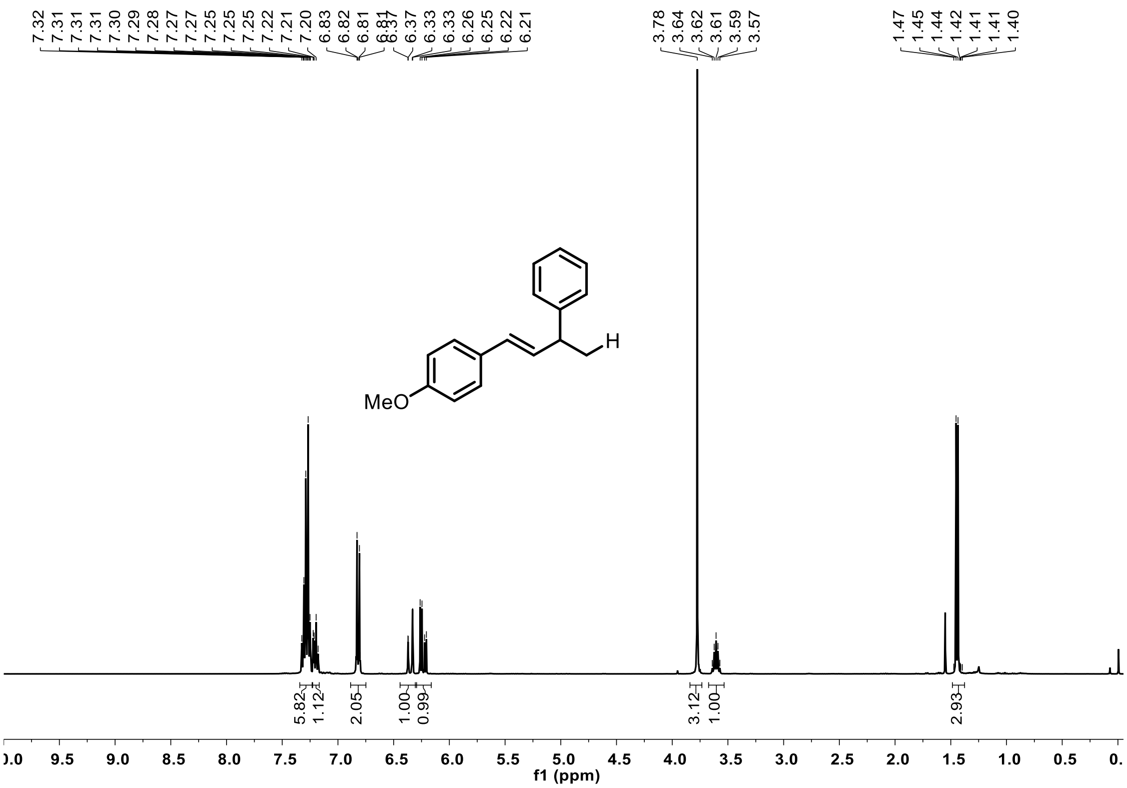

Supplementary Figure 153. ^{1}H NMR Spectrum of 3aa

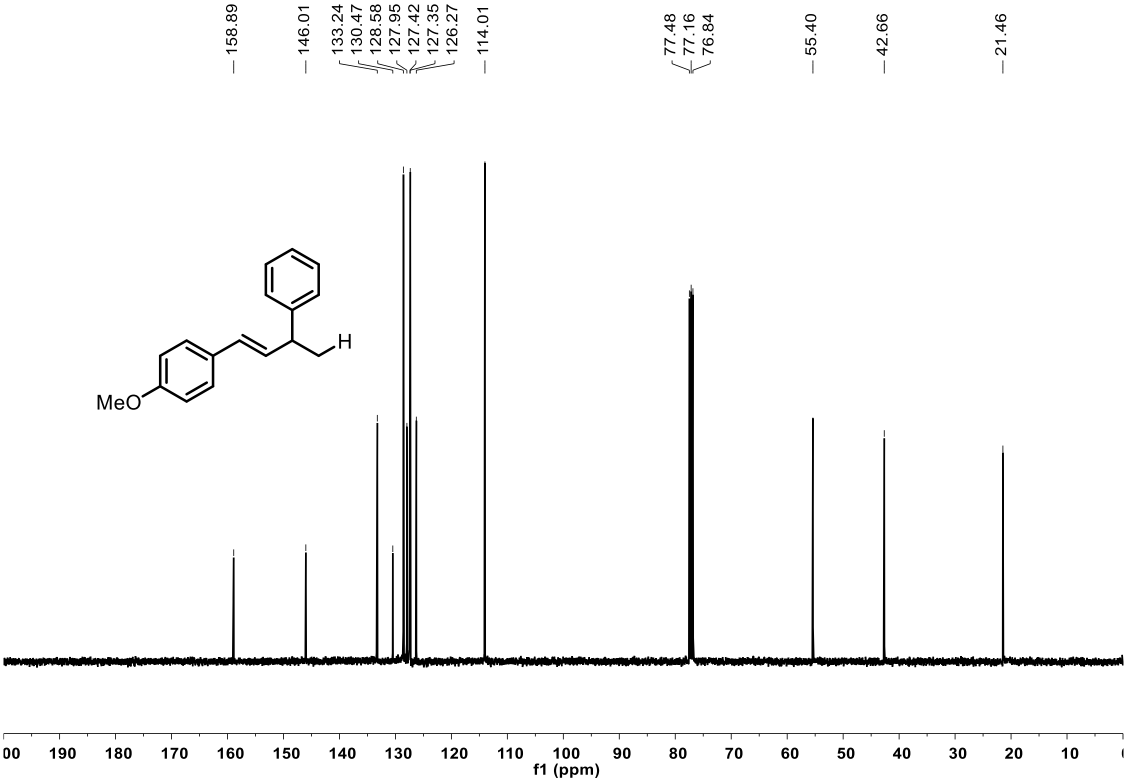

Supplementary Figure 154. ^{13}C NMR Spectrum of 3aa

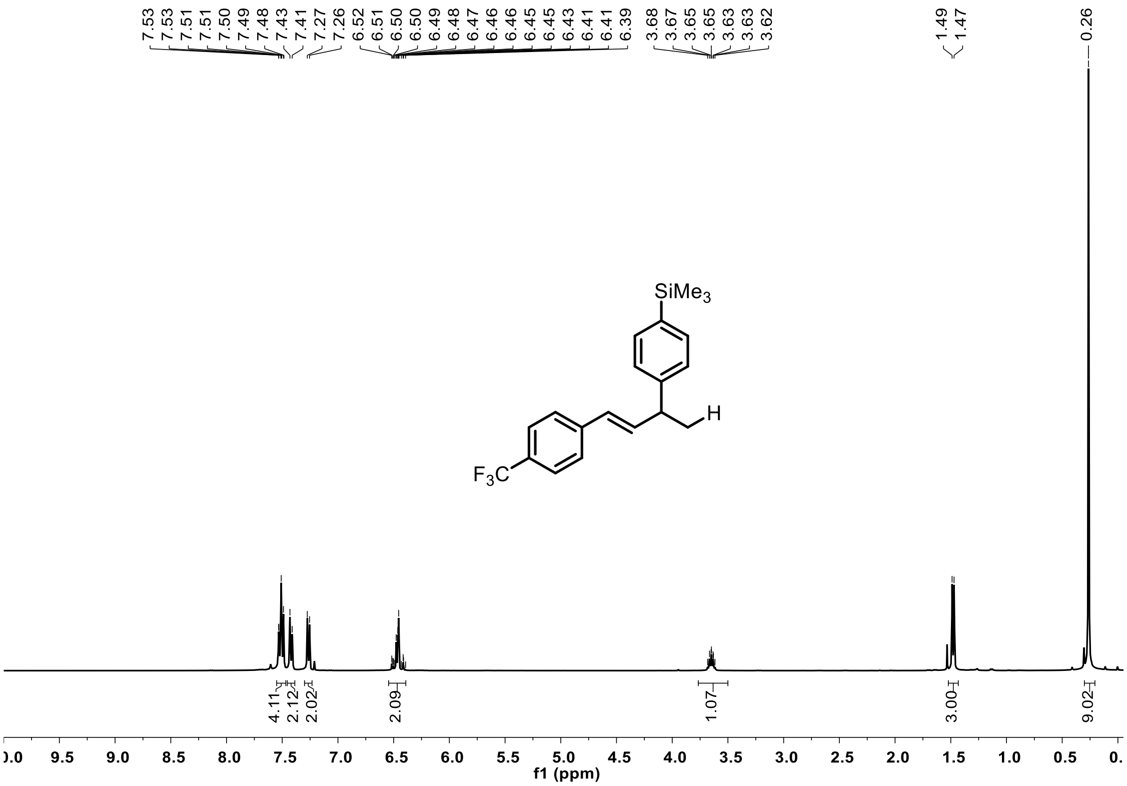

Supplementary Figure 155. ^{1}H NMR Spectrum of 3ab

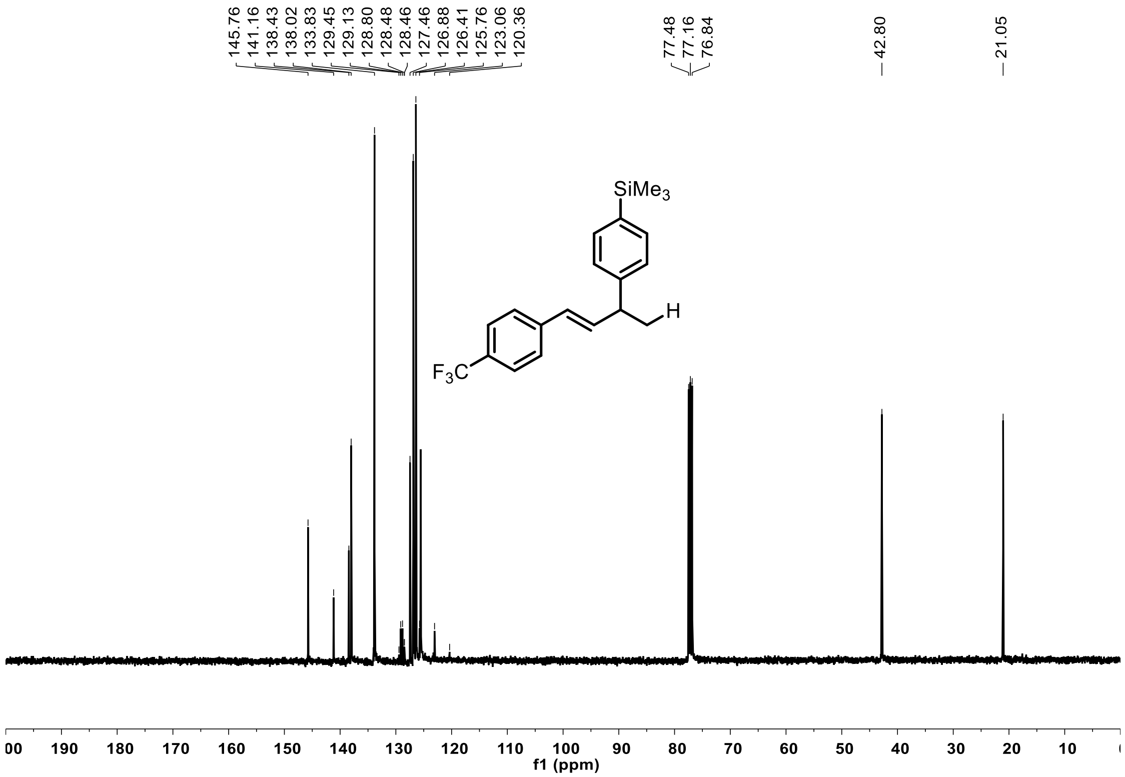

Supplementary Figure 156. ^{13}C NMR Spectrum of 3ab

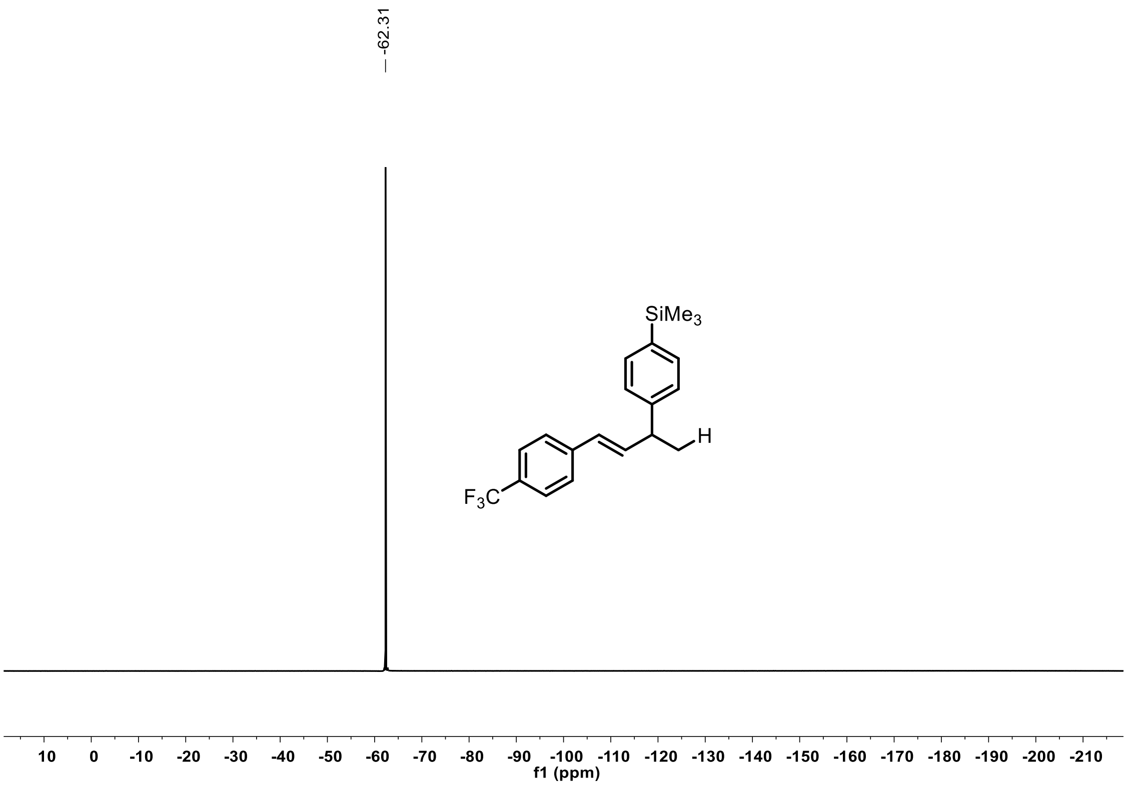

Supplementary Figure 157. ^{19}F NMR Spectrum of 3ab

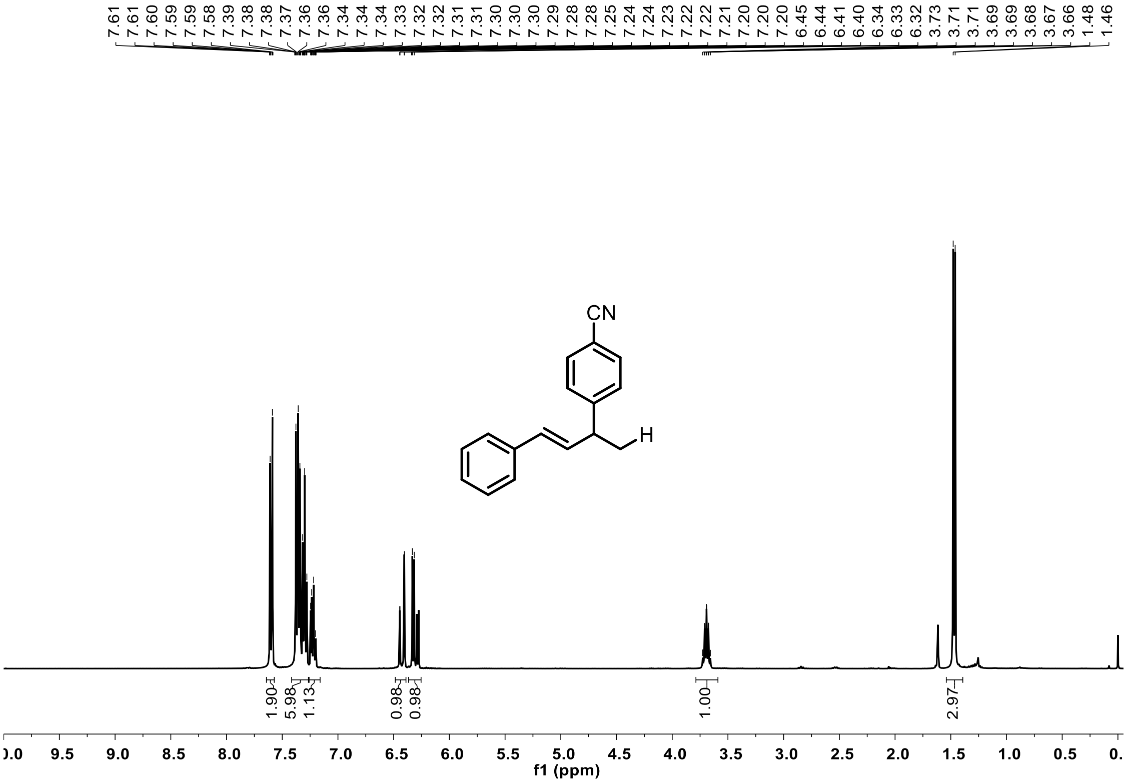

Supplementary Figure 158. ^{1}H NMR Spectrum of 3ac

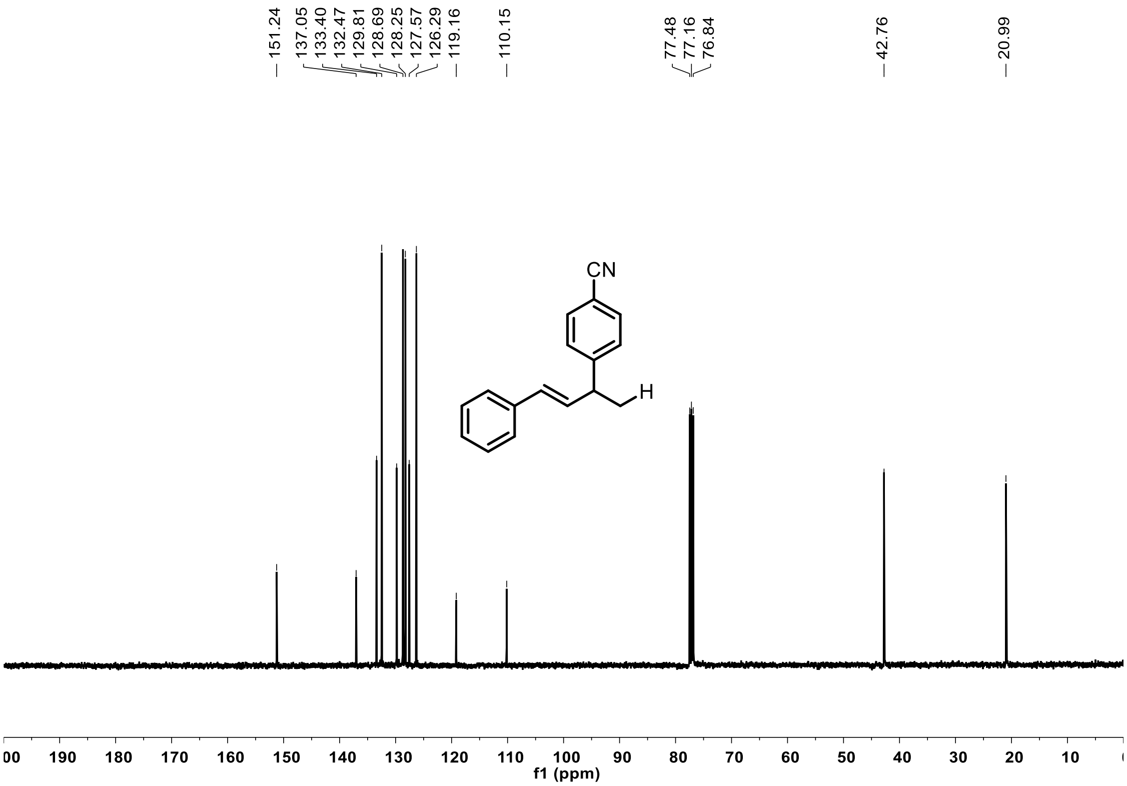

Supplementary Figure 159. ^{13}C NMR Spectrum of 3ac

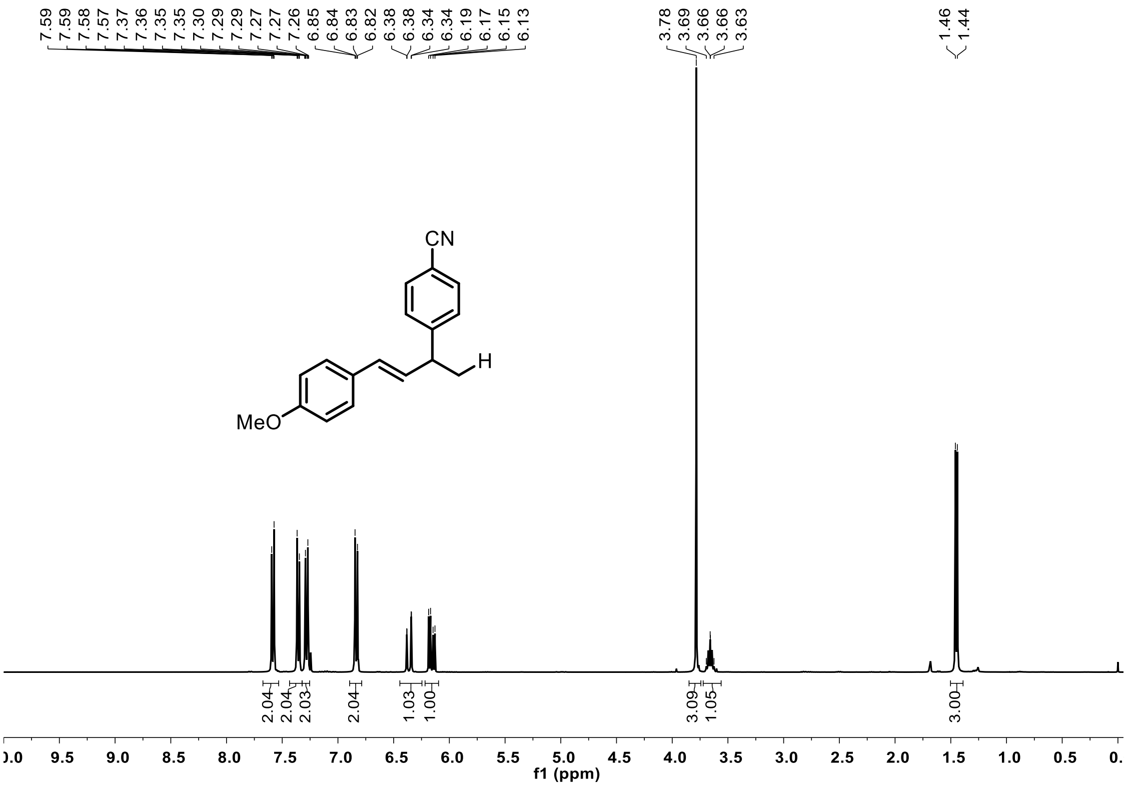

Supplementary Figure 160. ^{1}H NMR Spectrum of 3ad

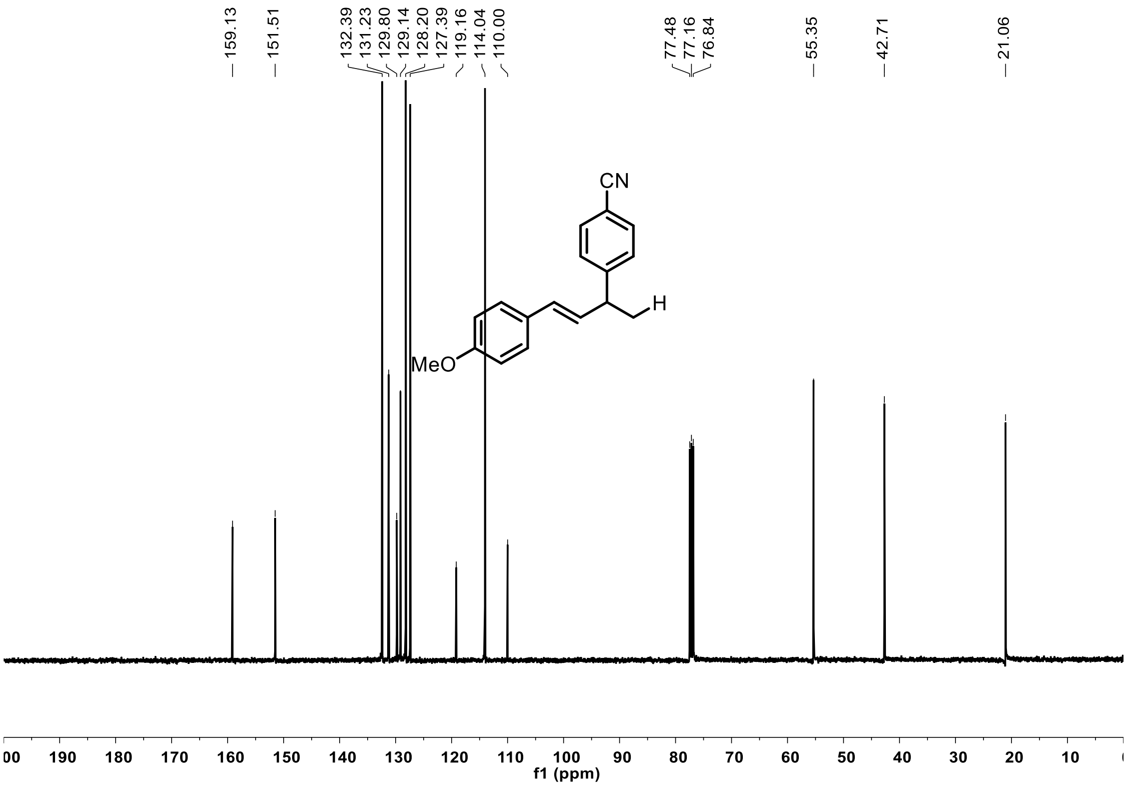

Supplementary Figure 161. ^{13}C NMR Spectrum of 3ad

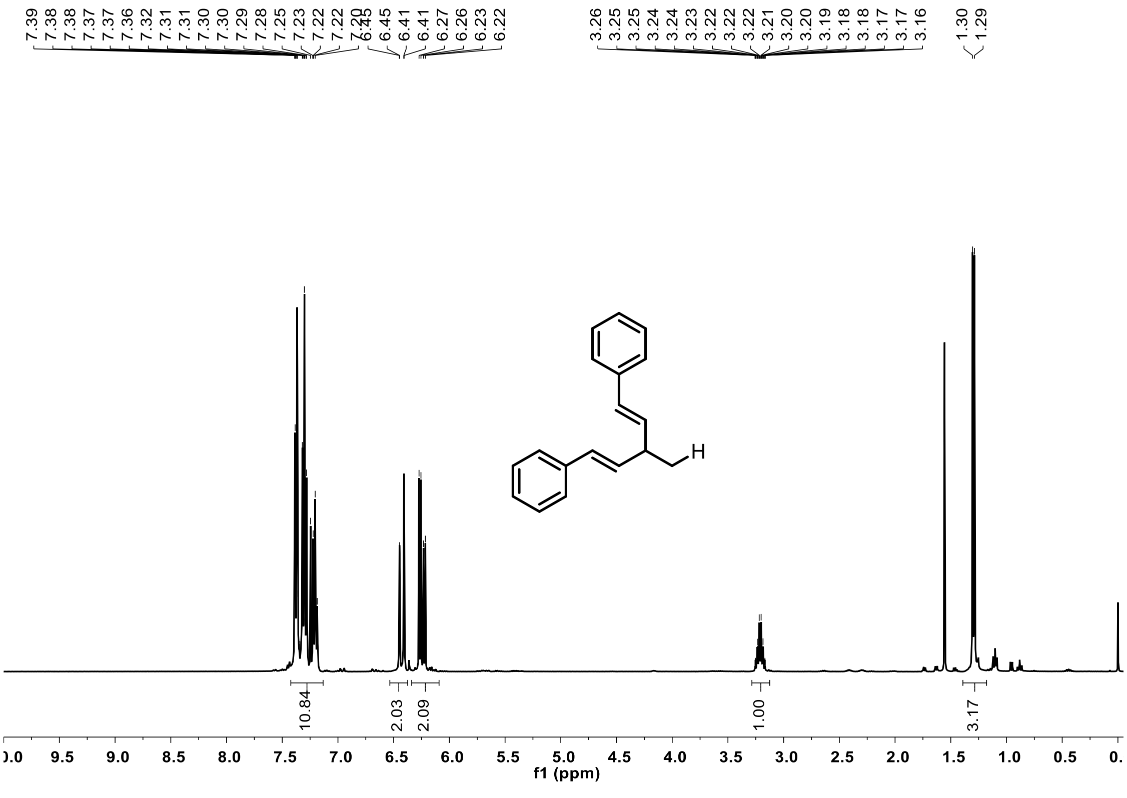

Supplementary Figure 162. ^{1}H NMR Spectrum of 3ae

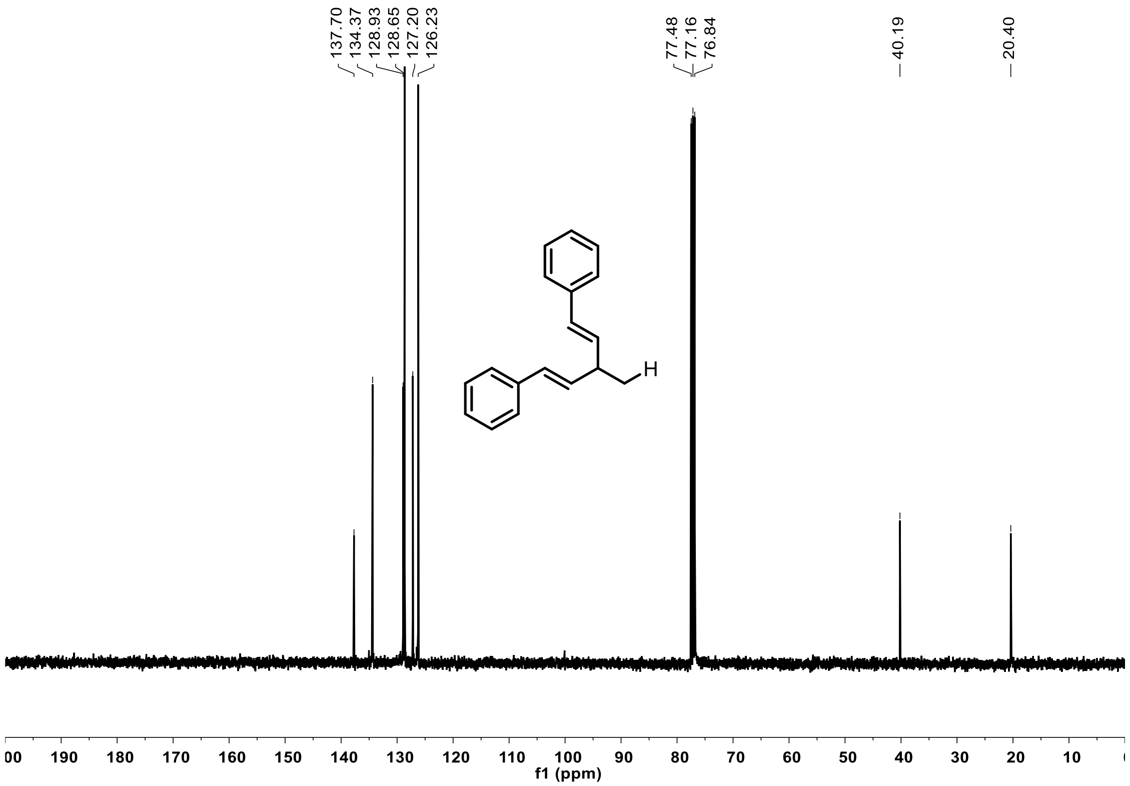

Supplementary Figure 163. ^{13}C NMR Spectrum of 3**ae**

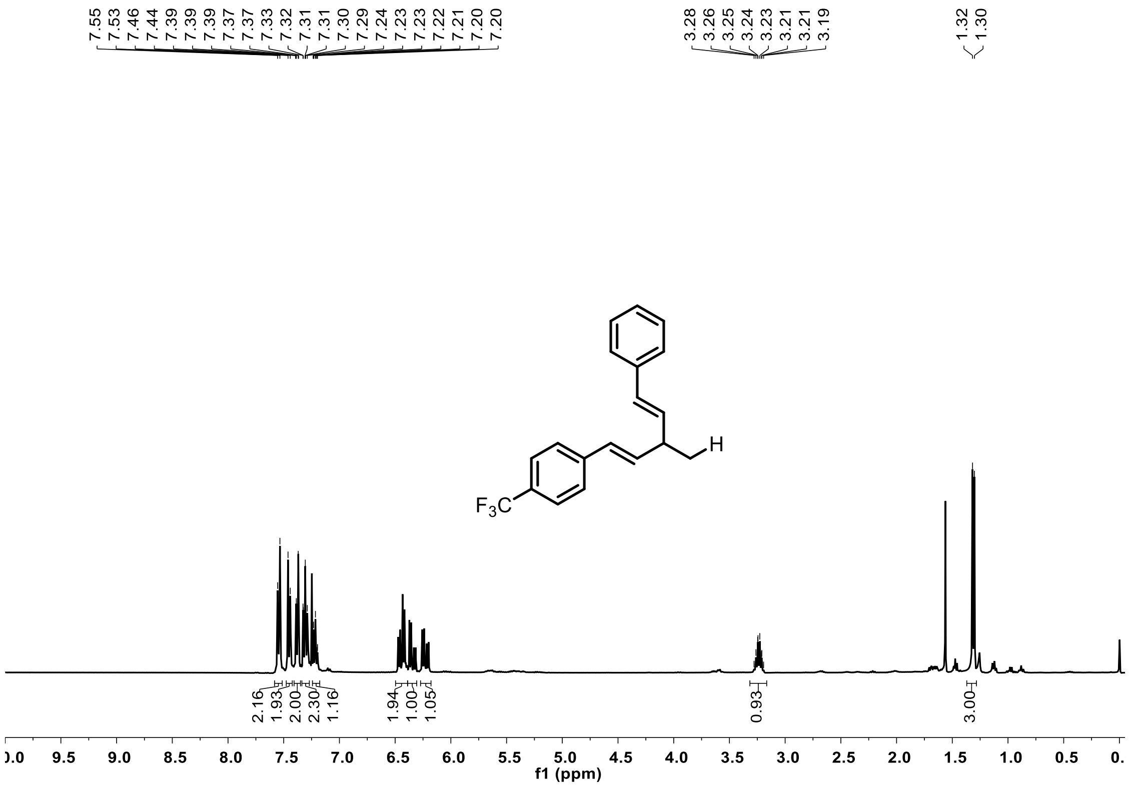

Supplementary Figure 164. ^{1}H NMR Spectrum of 3af

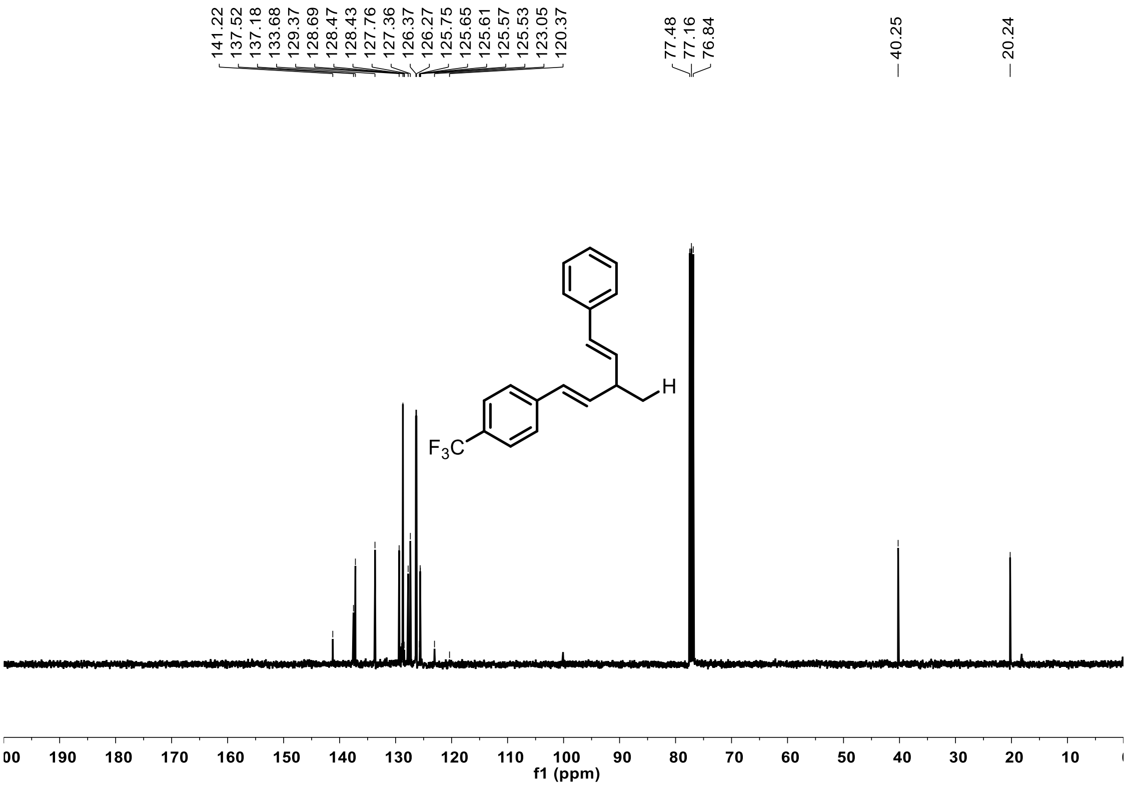

Supplementary Figure 165. ^{13}C NMR Spectrum of 3af

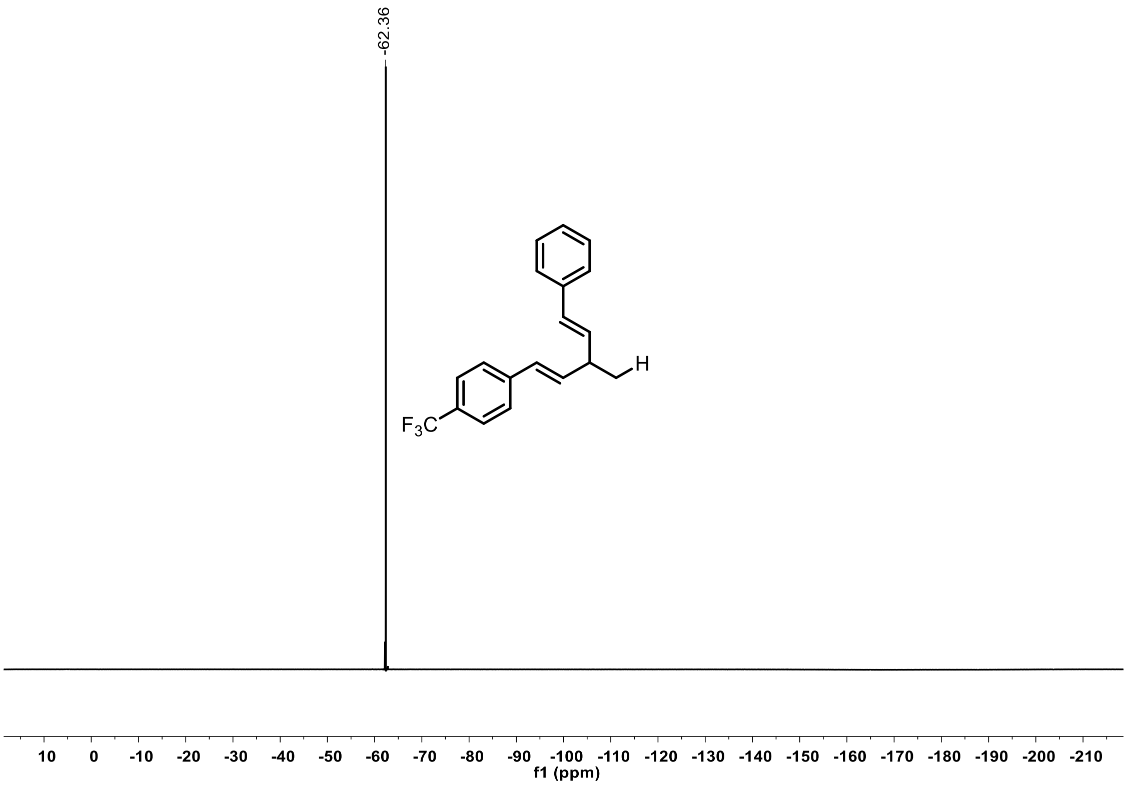

Supplementary Figure 166. ^{19}F NMR Spectrum of 3af

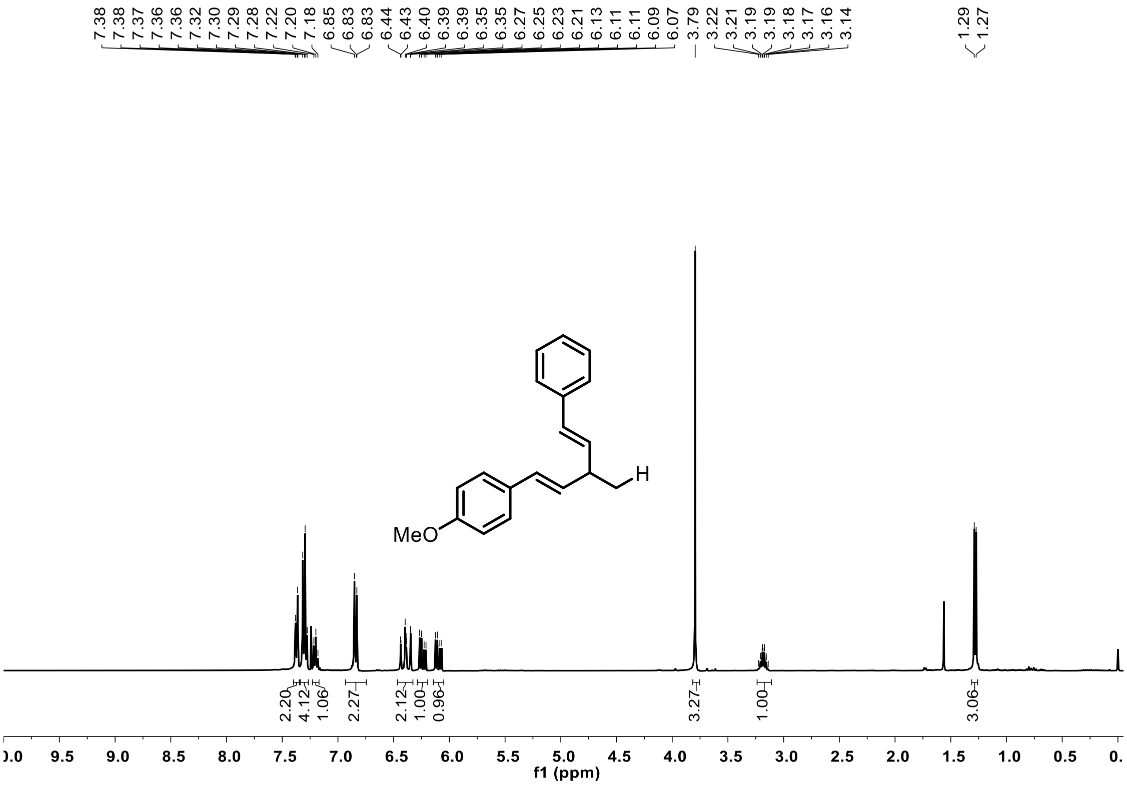

Supplementary Figure 167. ^{1}H NMR Spectrum of 3ag

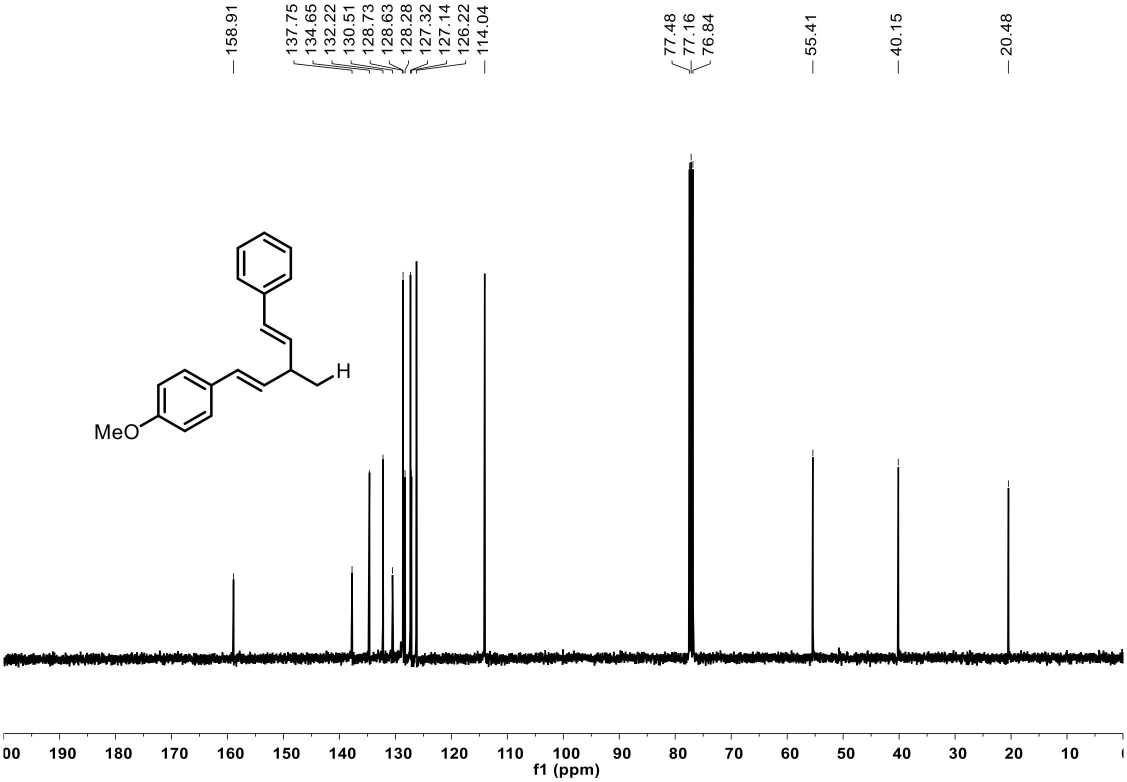

Supplementary Figure 168. ^{13}C NMR Spectrum of 3ag

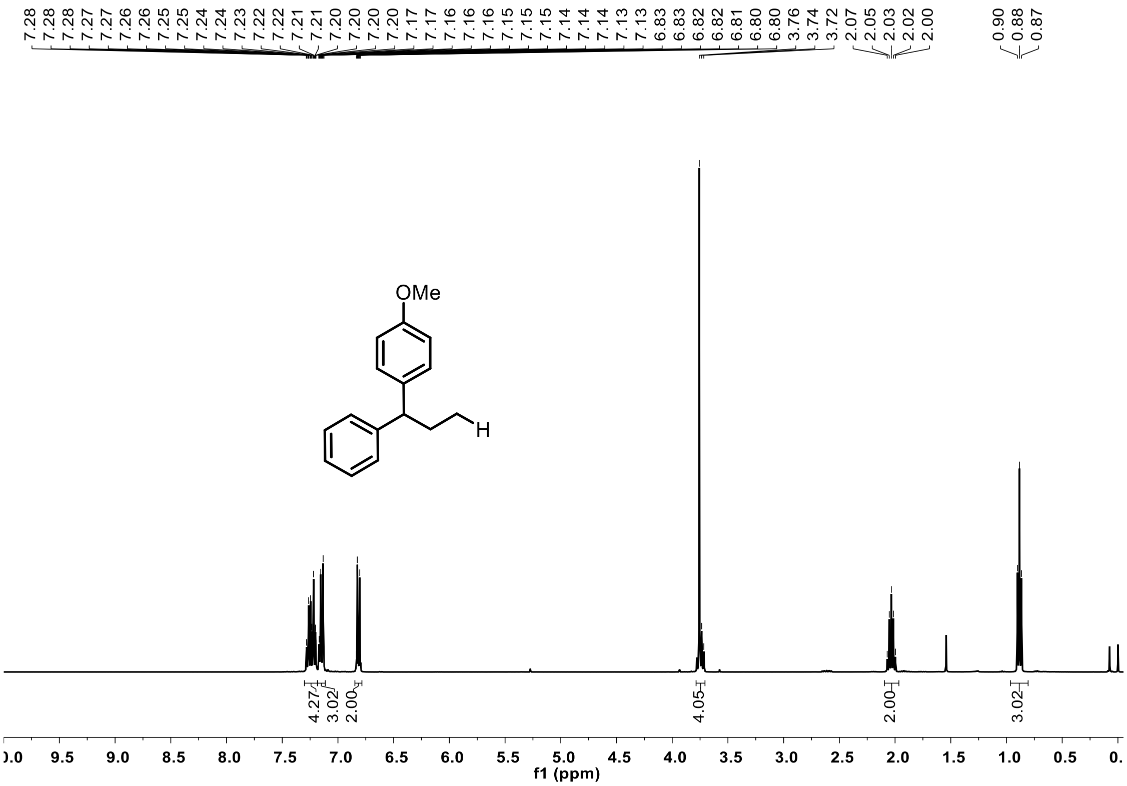

Supplementary Figure 169. ^{1}H NMR Spectrum of 3ah

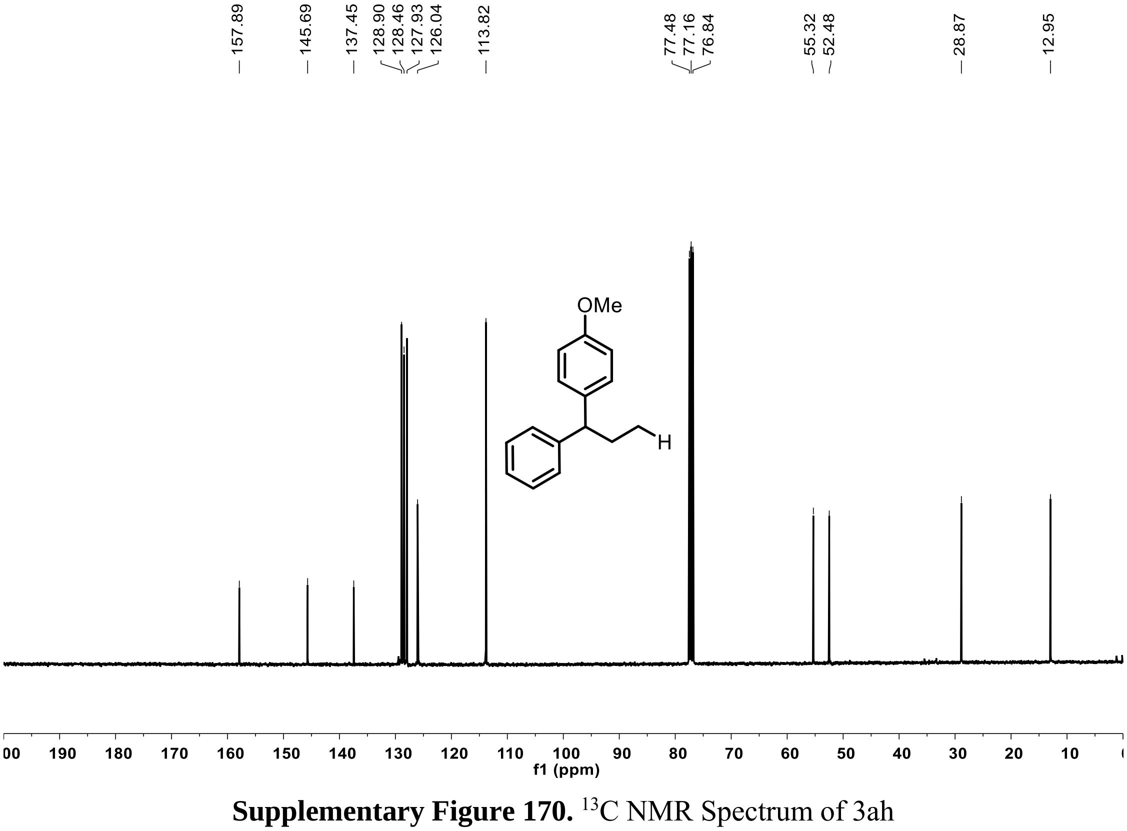

Supplementary Figure 170. ^{13}C NMR Spectrum of 3ah

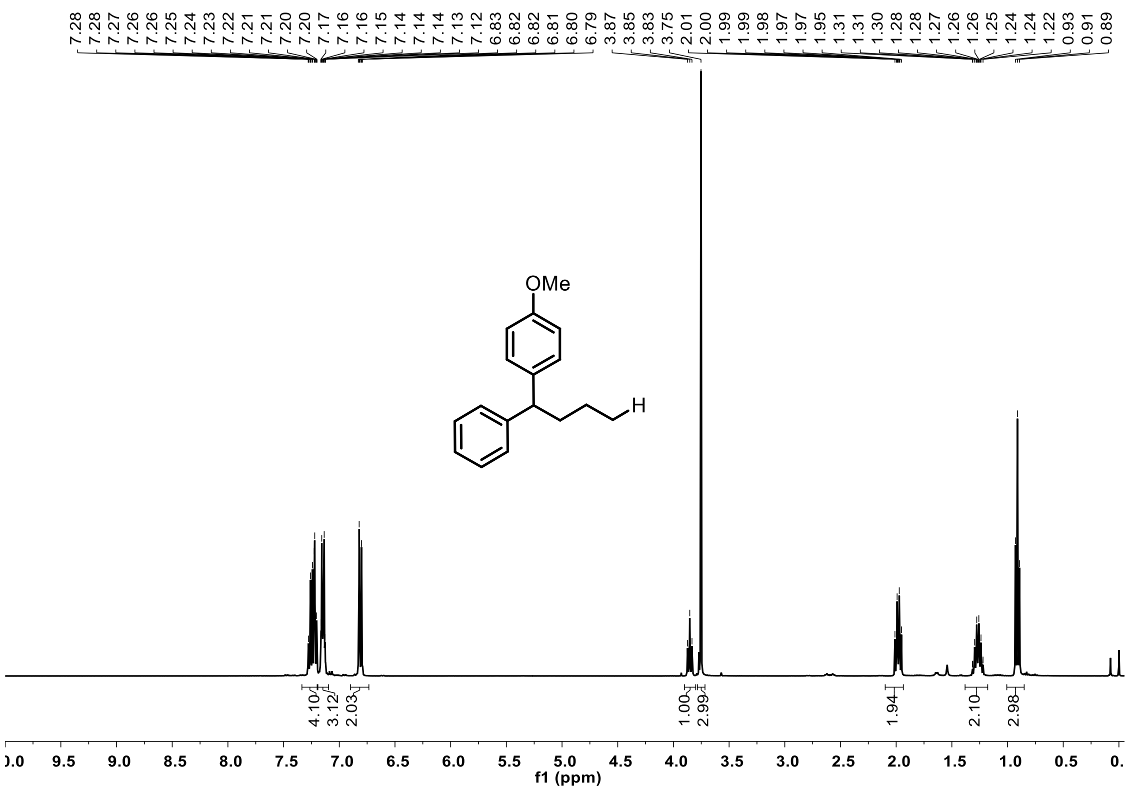

Supplementary Figure 171. ^{1}H NMR Spectrum of 3ai

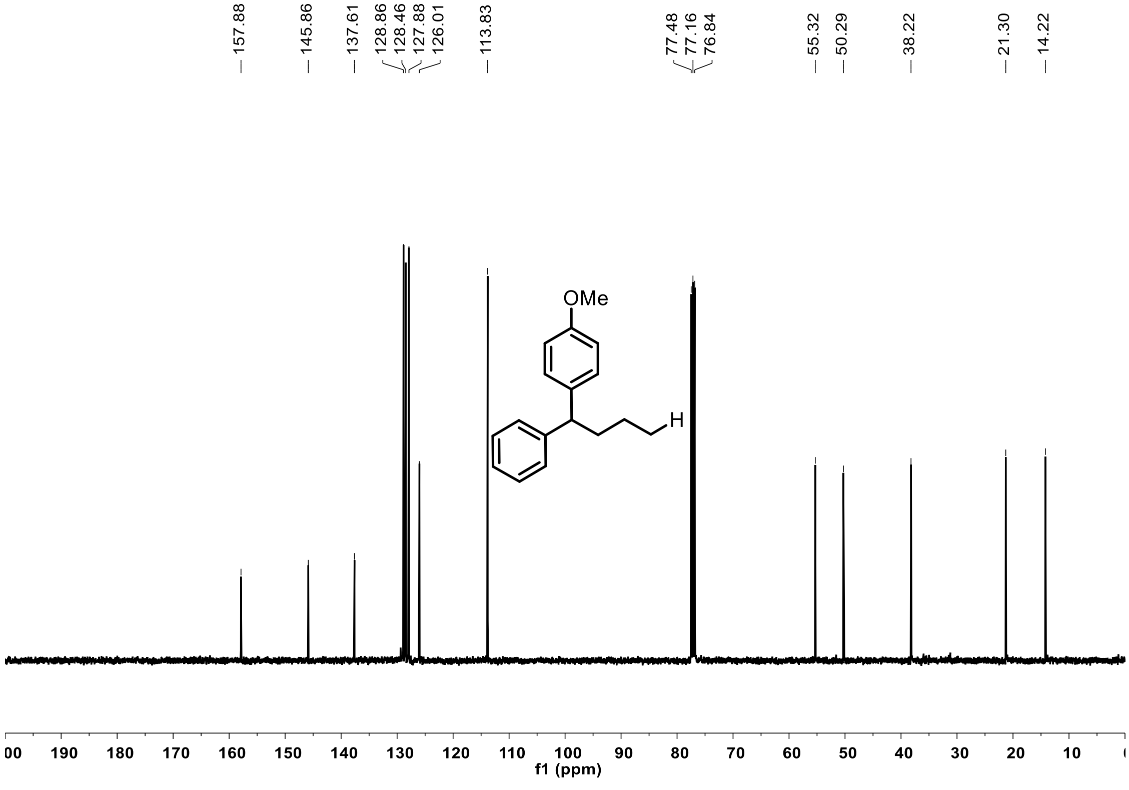

Supplementary Figure 172. ^{13}C NMR Spectrum of 3ai

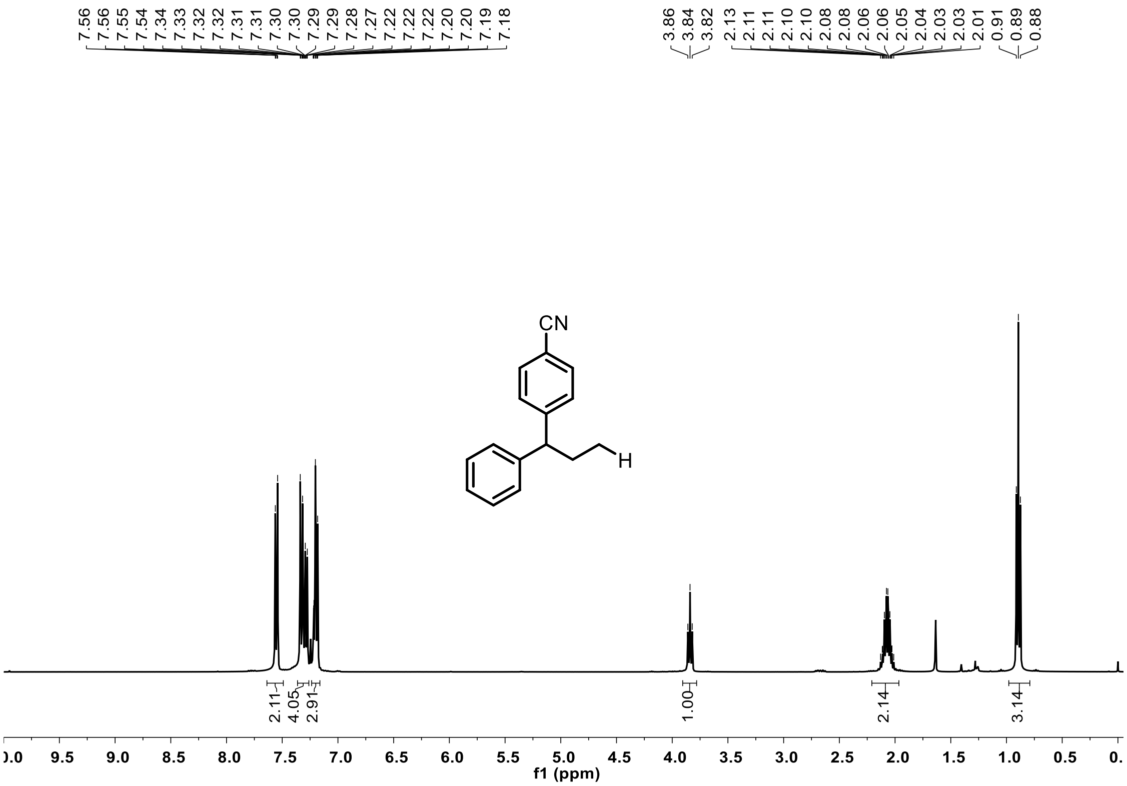

Supplementary Figure 173. ^{1}H NMR Spectrum of 3aj

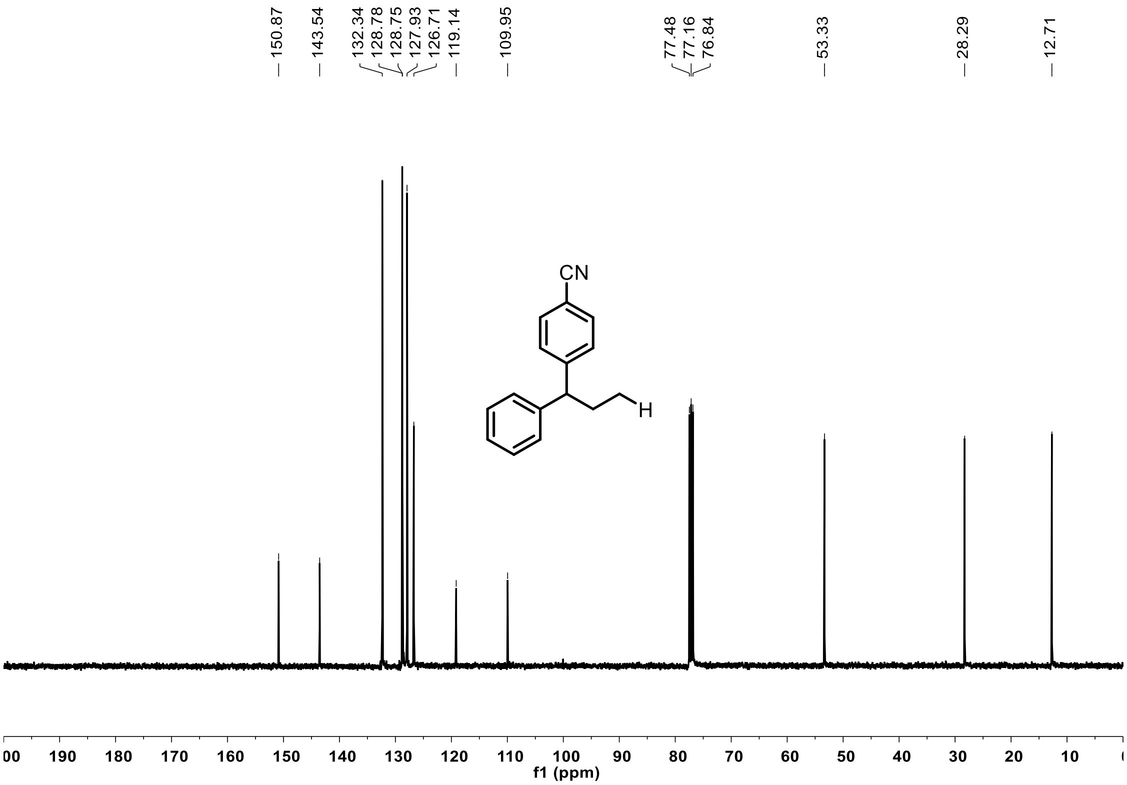

Supplementary Figure 174. ^{13}C NMR Spectrum of 3aj

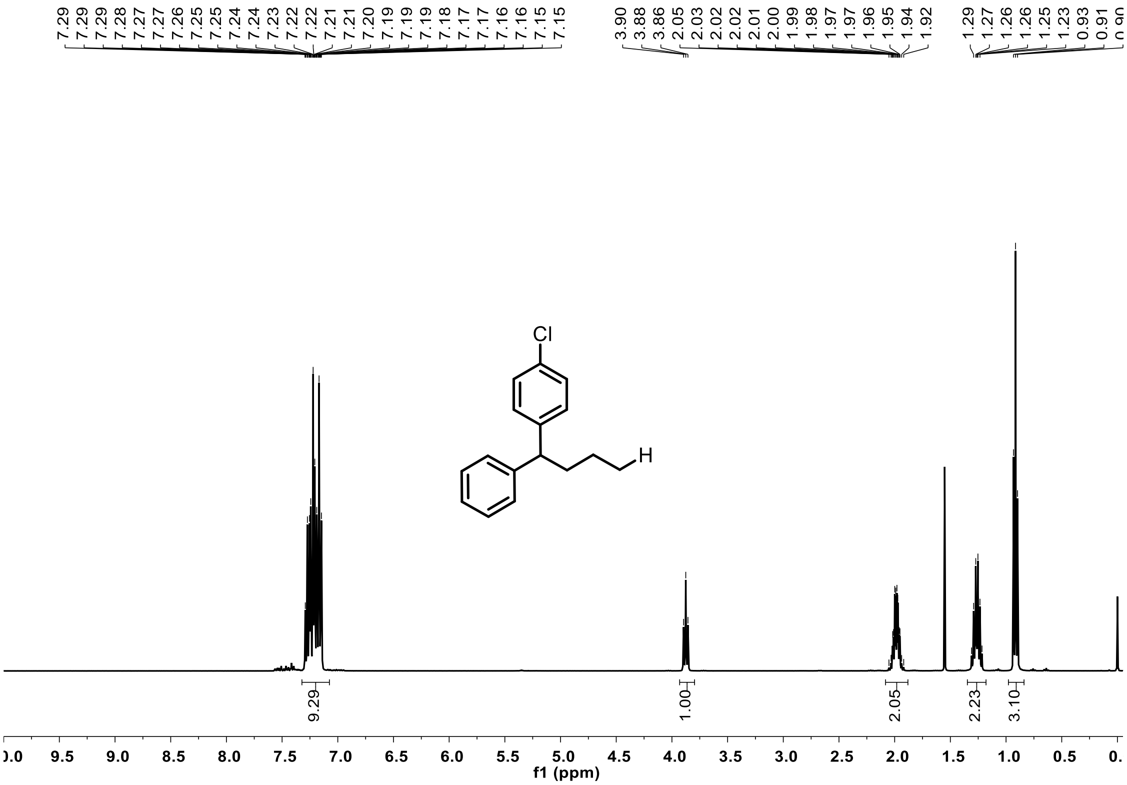

Supplementary Figure 175. ^{1}H NMR Spectrum of 3ak

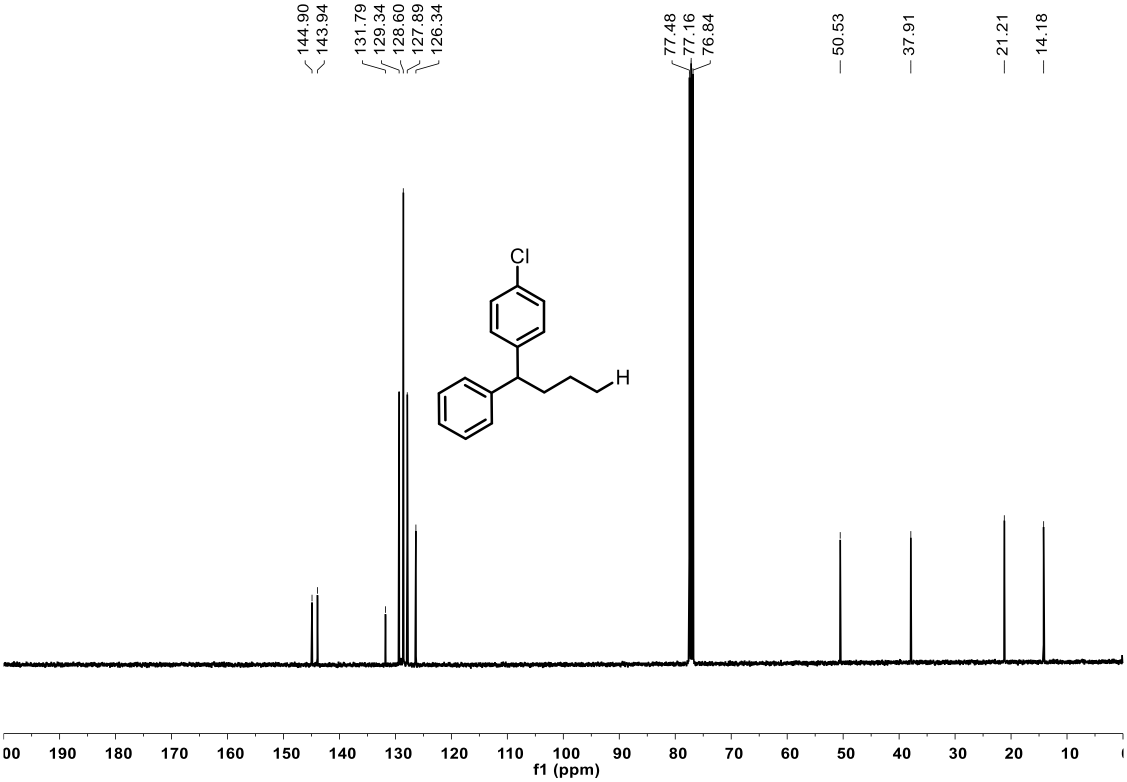

Supplementary Figure 176. ^{13}C NMR Spectrum of 3ak

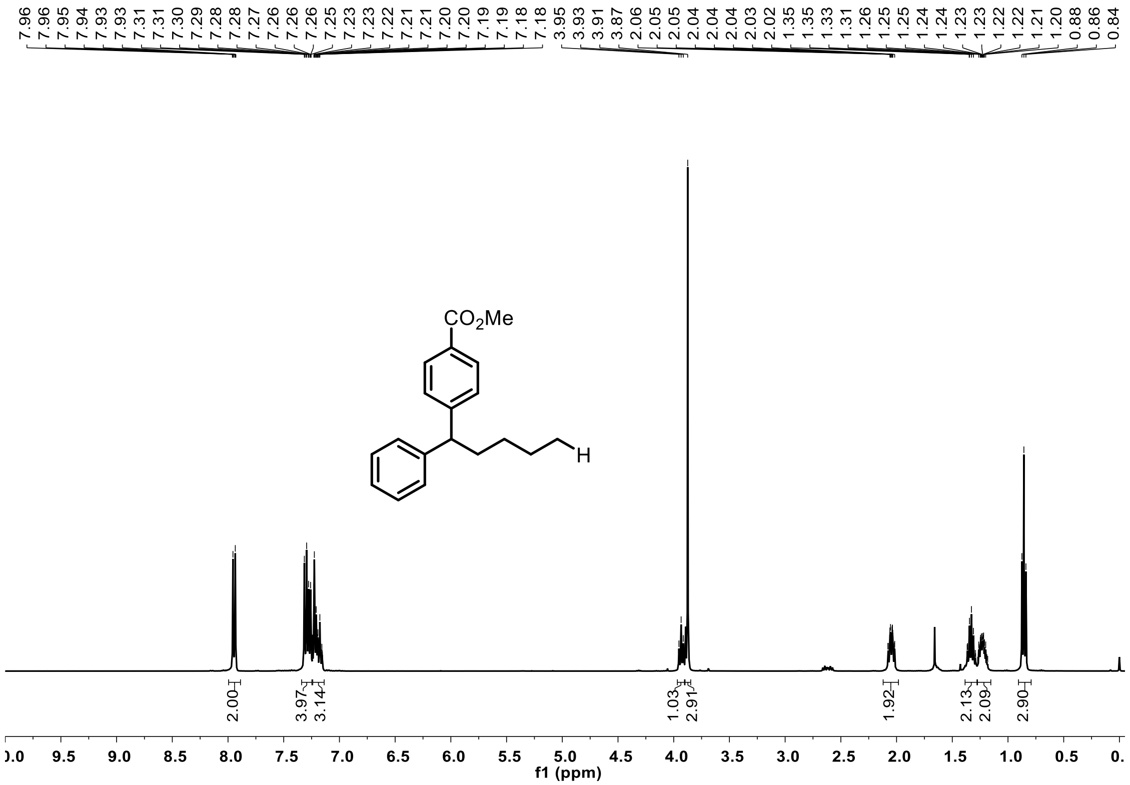

Supplementary Figure 177. ^{1}H NMR Spectrum of 3al

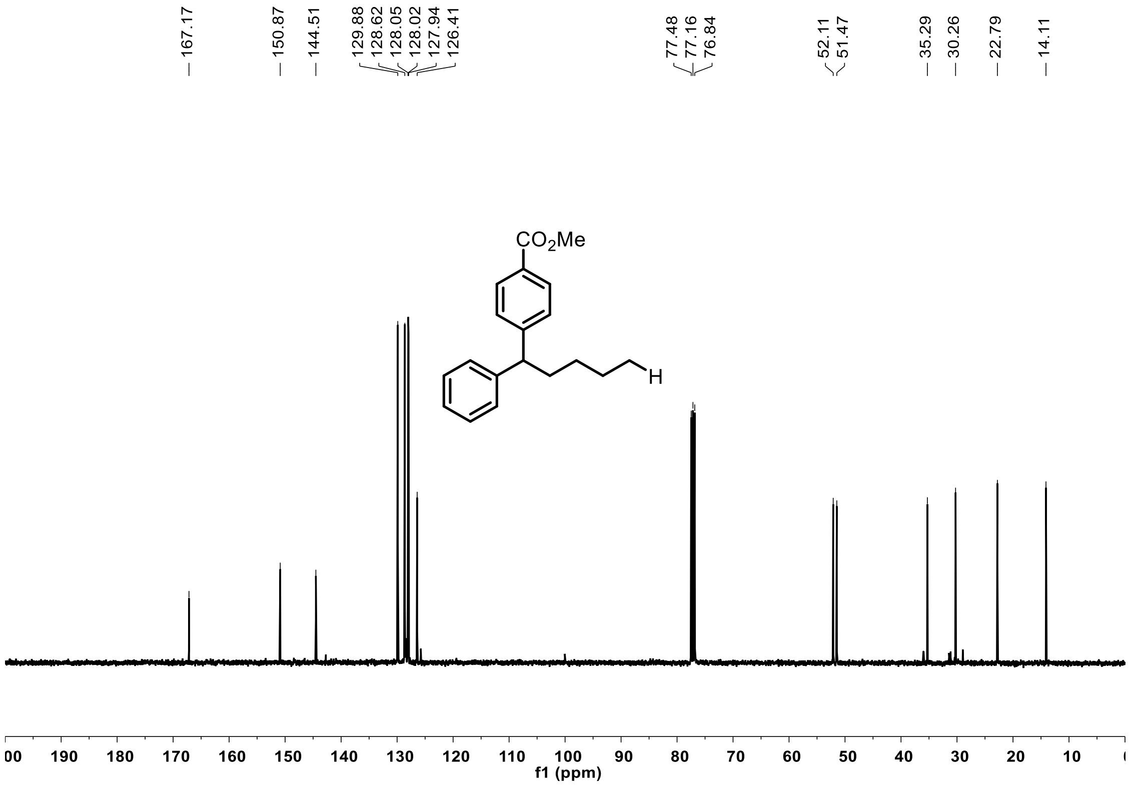

Supplementary Figure 178. ^{13}C NMR Spectrum of 3al

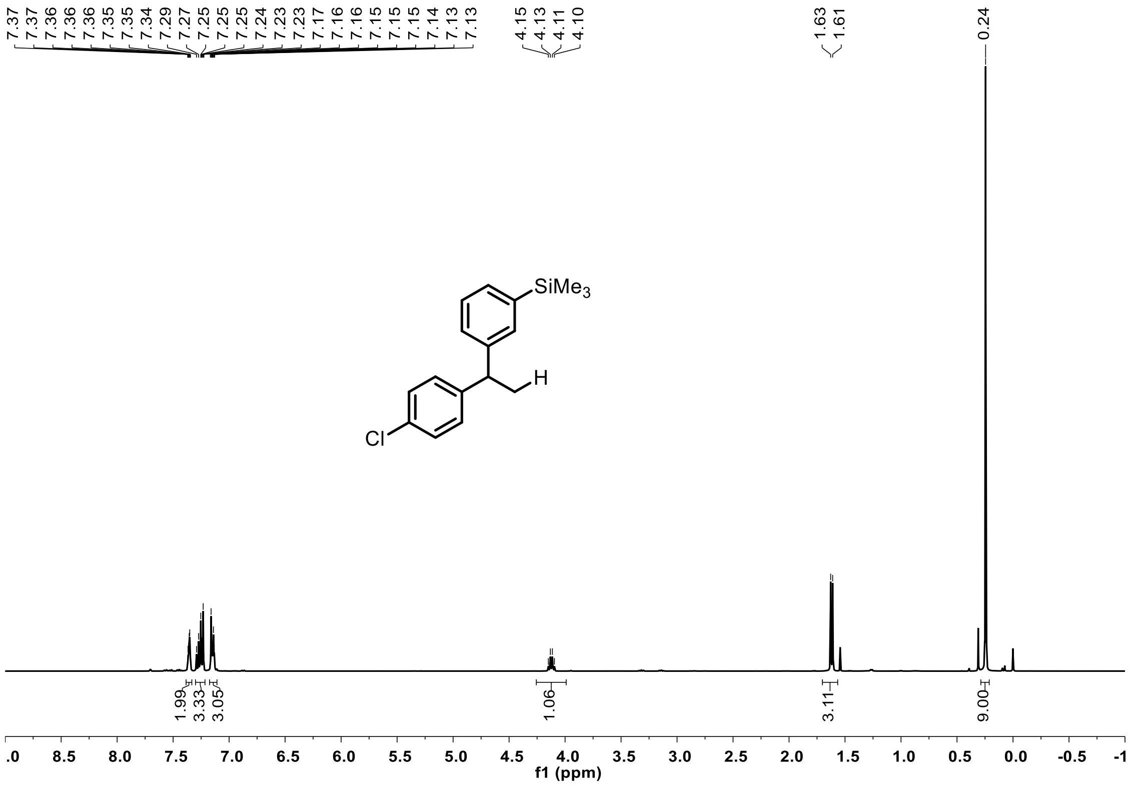

Supplementary Figure 179. ^{1}H NMR Spectrum of 3am

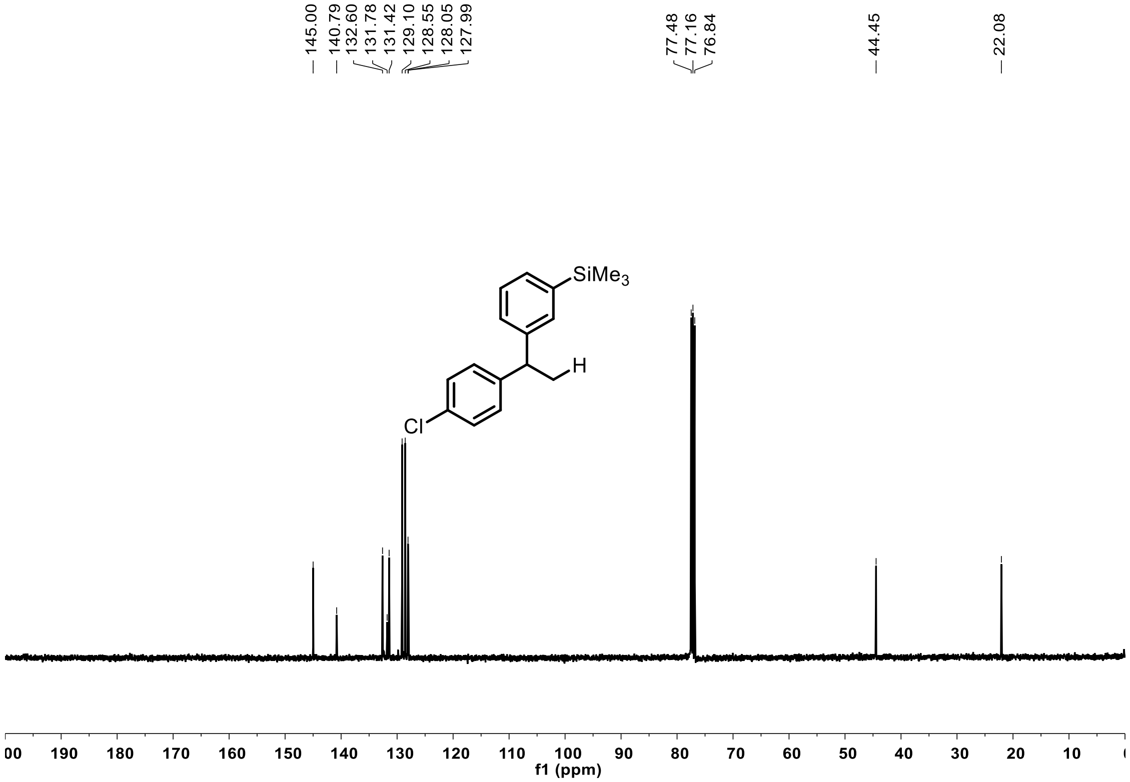

Supplementary Figure 180. ^{13}C NMR Spectrum of 3am

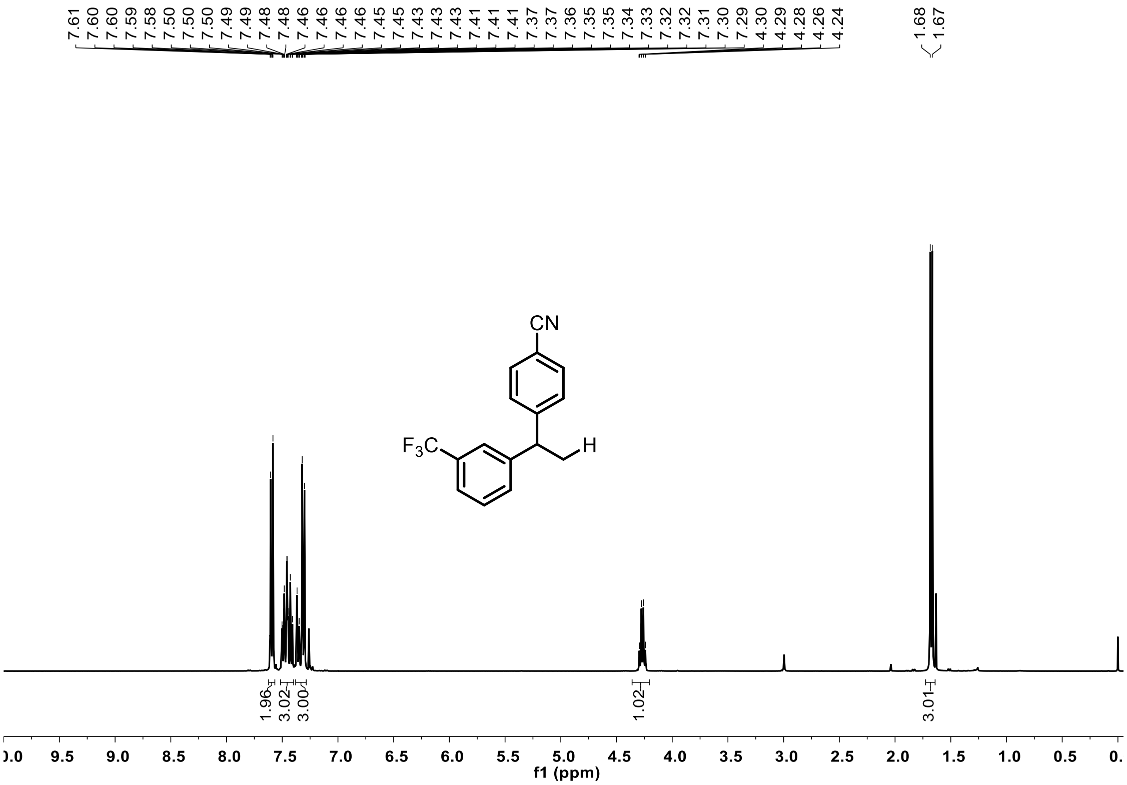

Supplementary Figure 181. ^{1}H NMR Spectrum of 3an

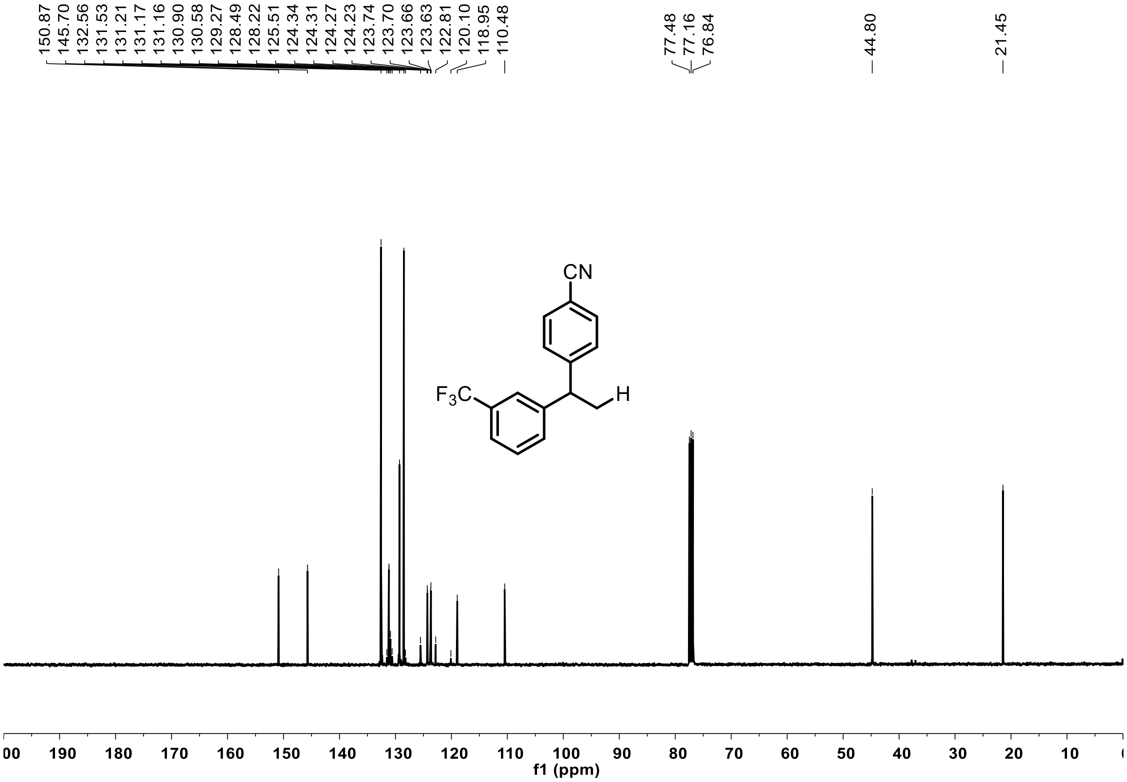

Supplementary Figure 182. ^{13}C NMR Spectrum of 3an

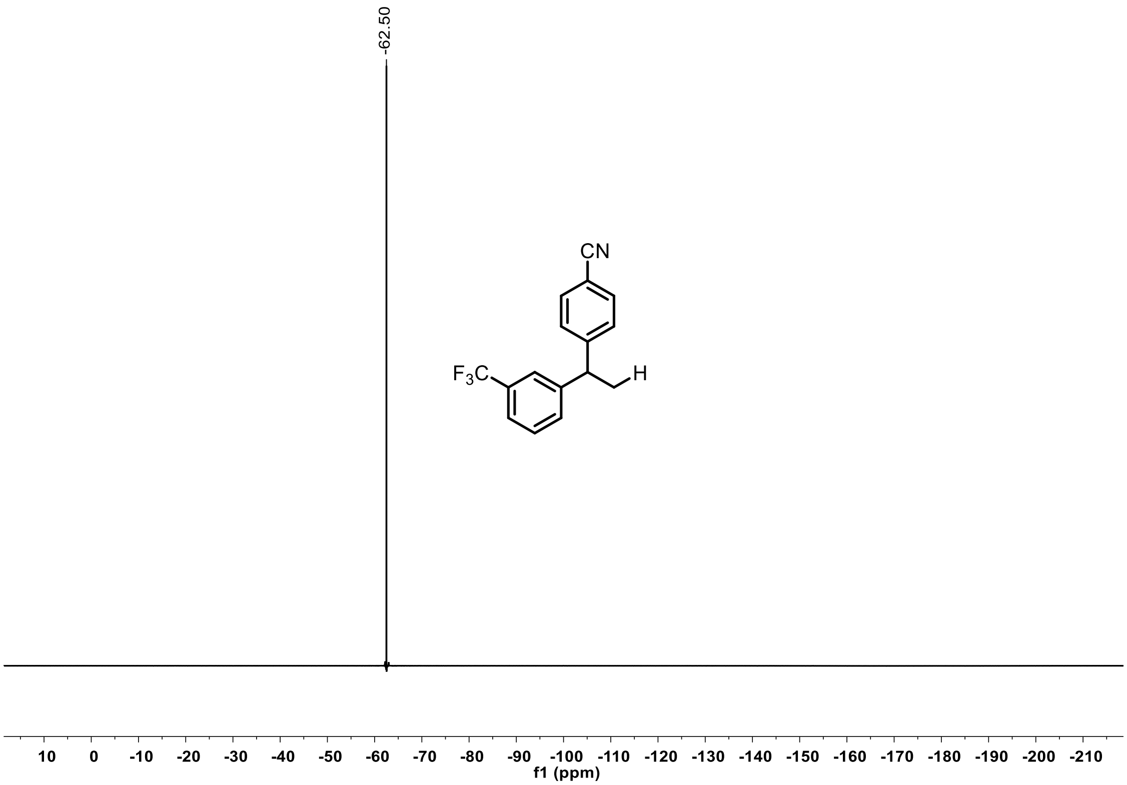

Supplementary Figure 183. ^{19}F NMR Spectrum of 3an

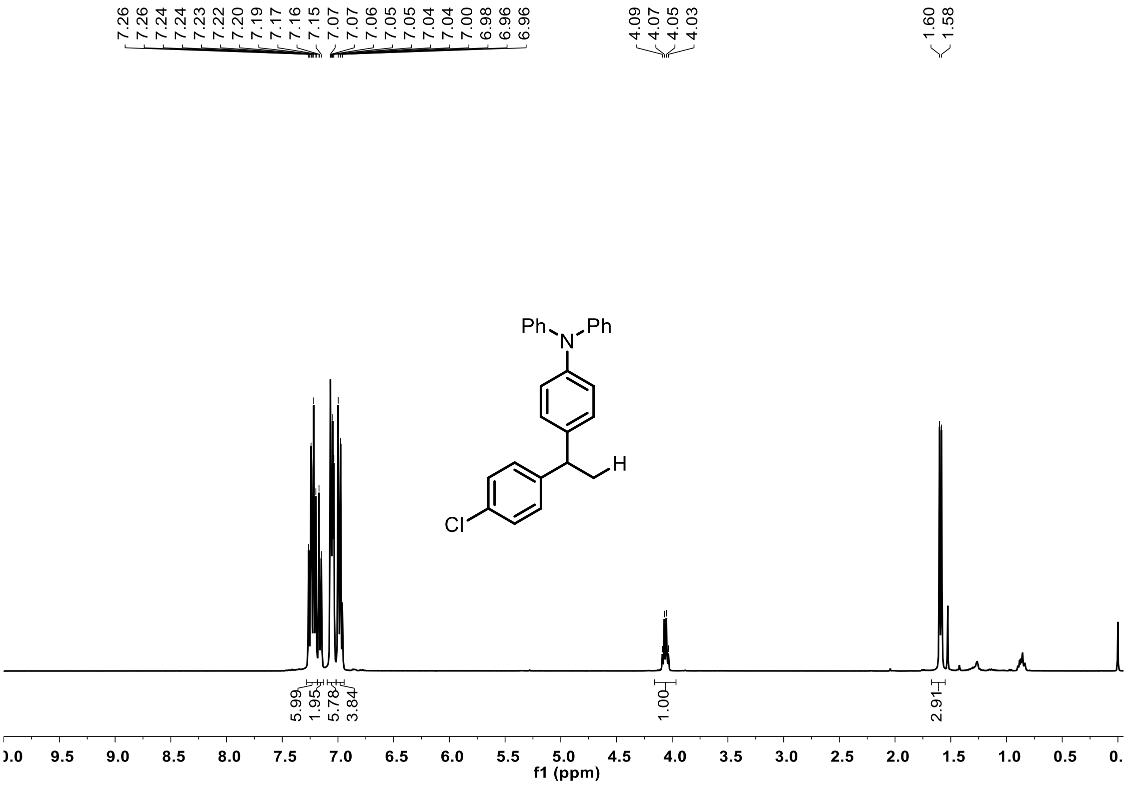

Supplementary Figure 184. ^{1}H NMR Spectrum of 3ao

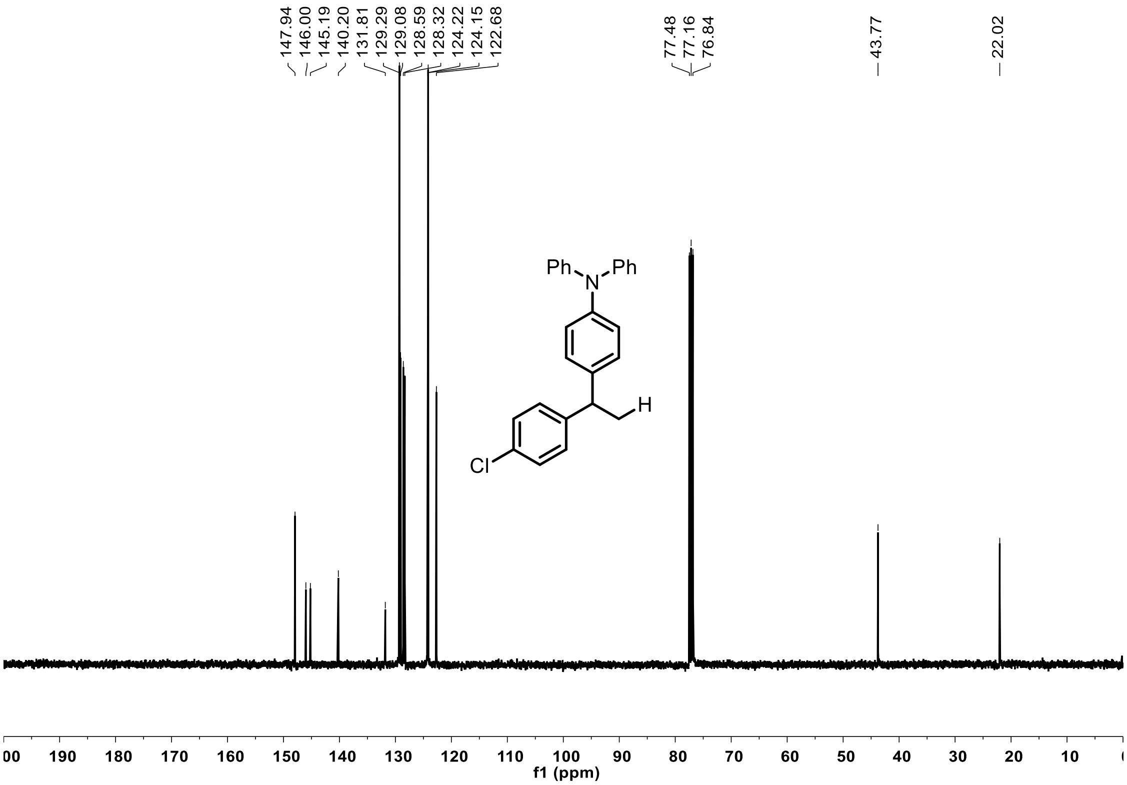

Supplementary Figure 185. ^{13}C NMR Spectrum of 3ao

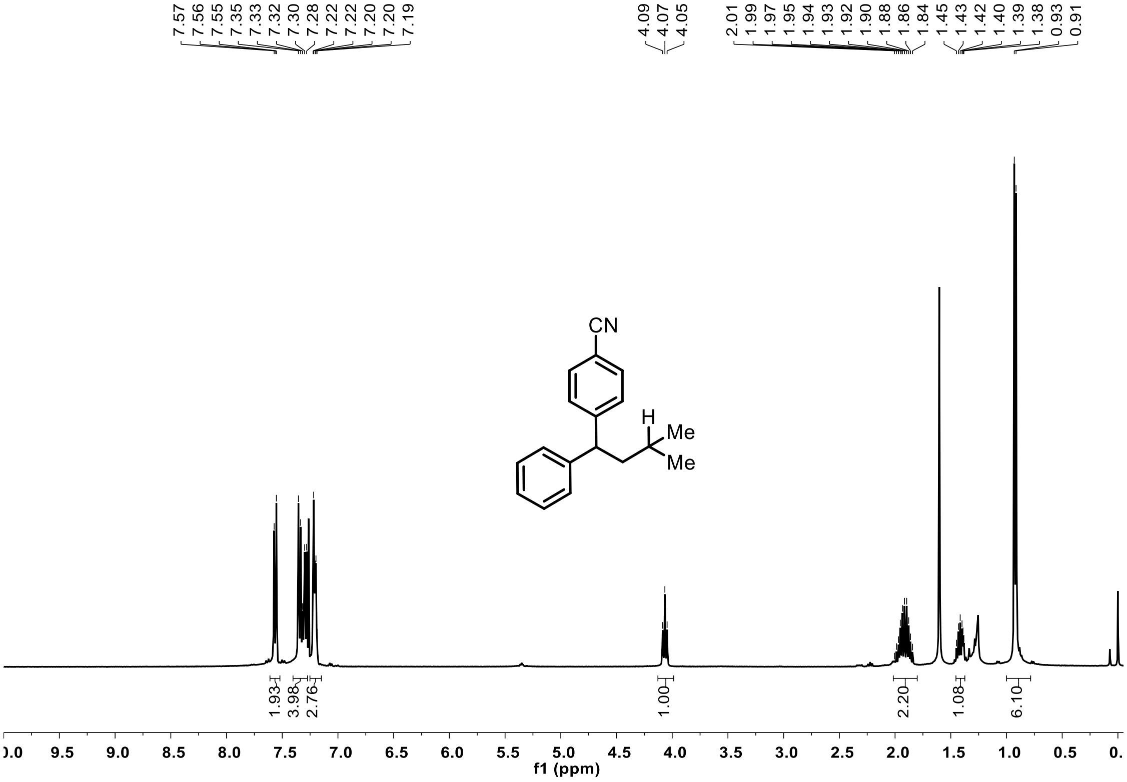

Supplementary Figure 186. ^{1}H NMR Spectrum of 3ap

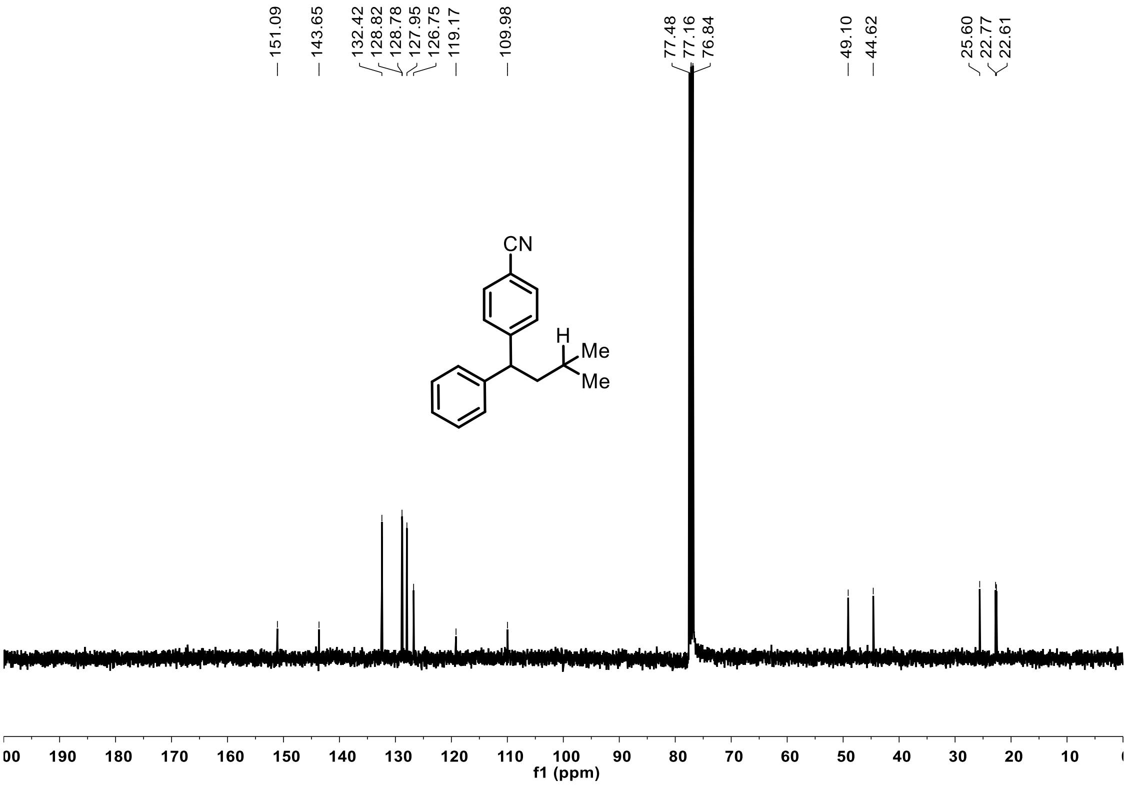

Supplementary Figure 187. ^{13}C NMR Spectrum of 3ap

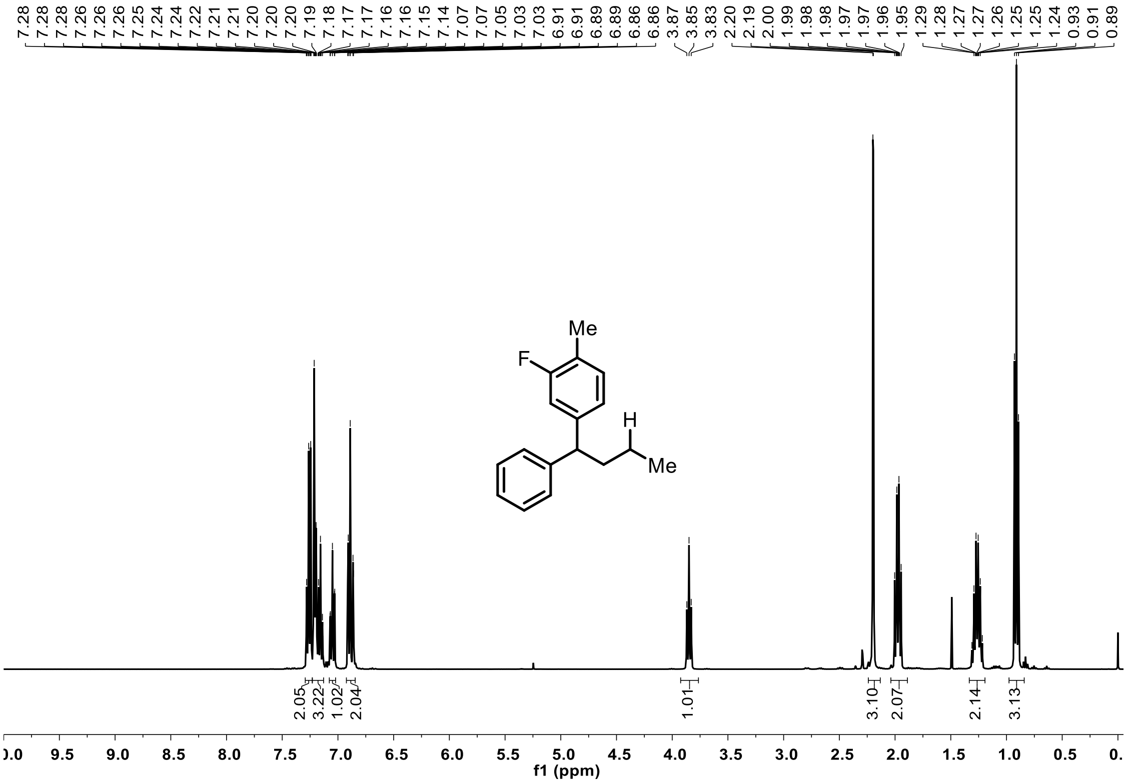

Supplementary Figure 188. ^{1}H NMR Spectrum of 3aq

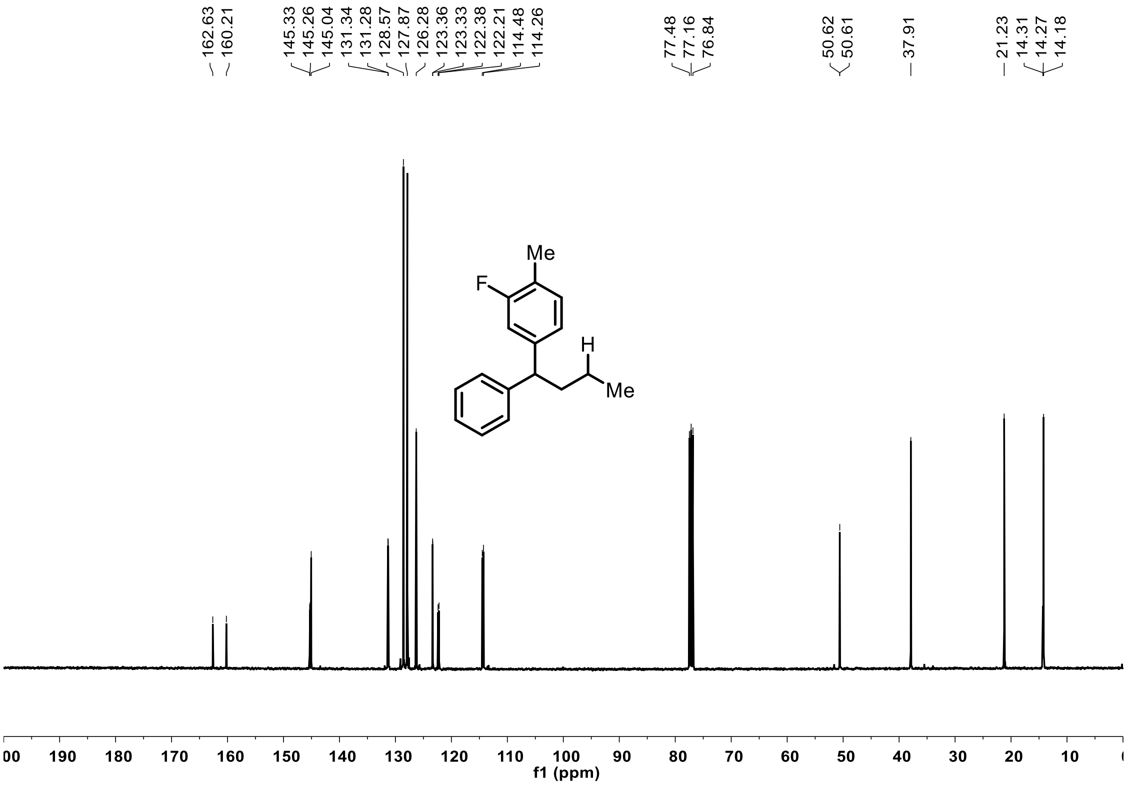

Supplementary Figure 189. ^{13}C NMR Spectrum of 3aq

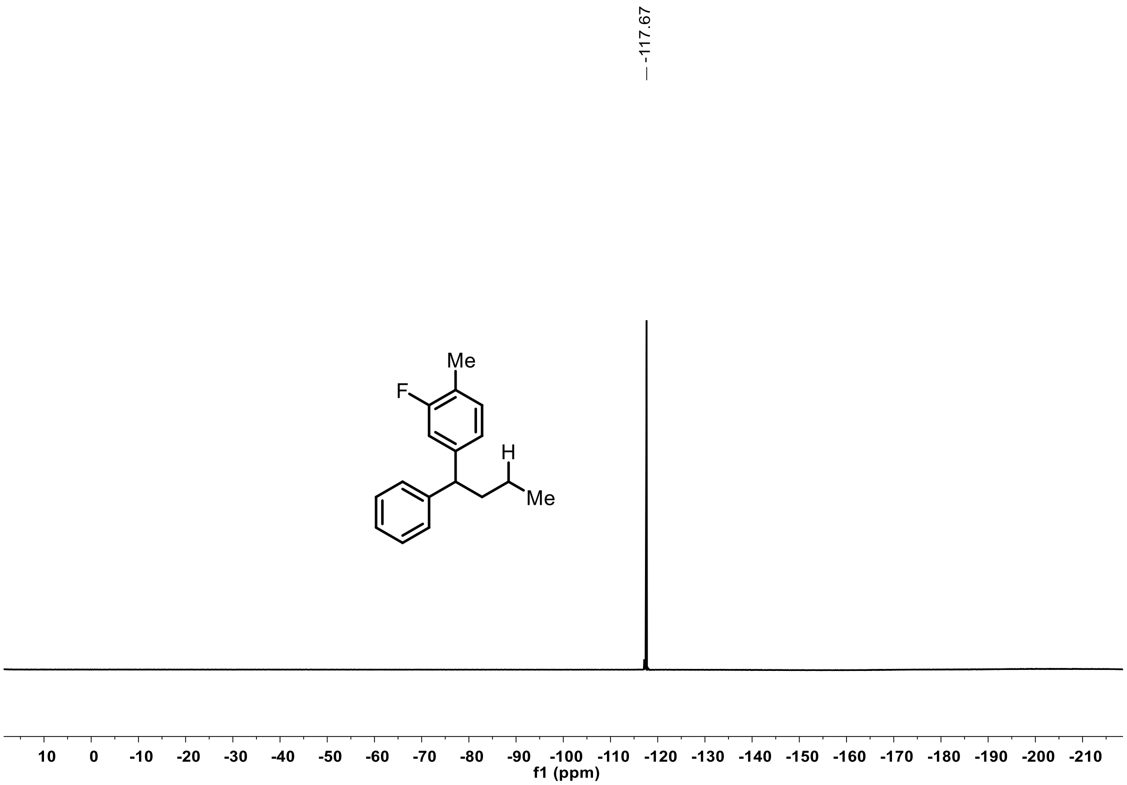

Supplementary Figure 190. ^{19}F NMR Spectrum of 3aq

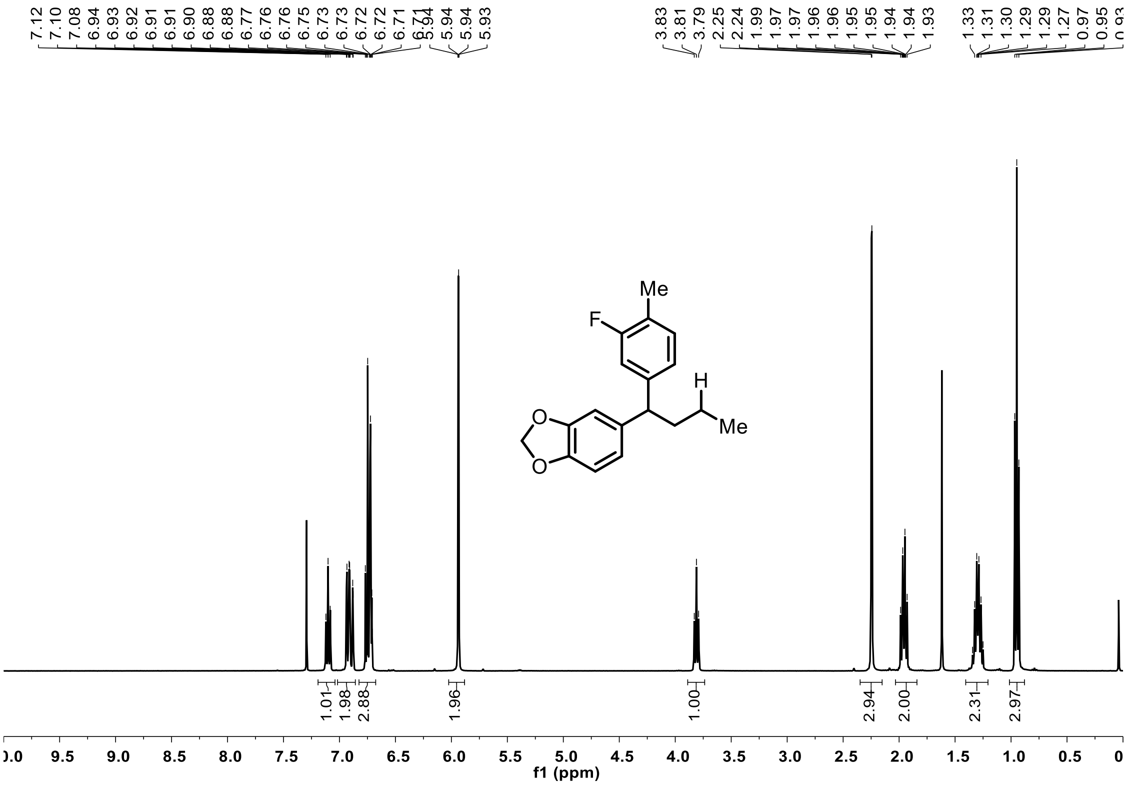

Supplementary Figure 191. ^{1}H NMR Spectrum of 3ar

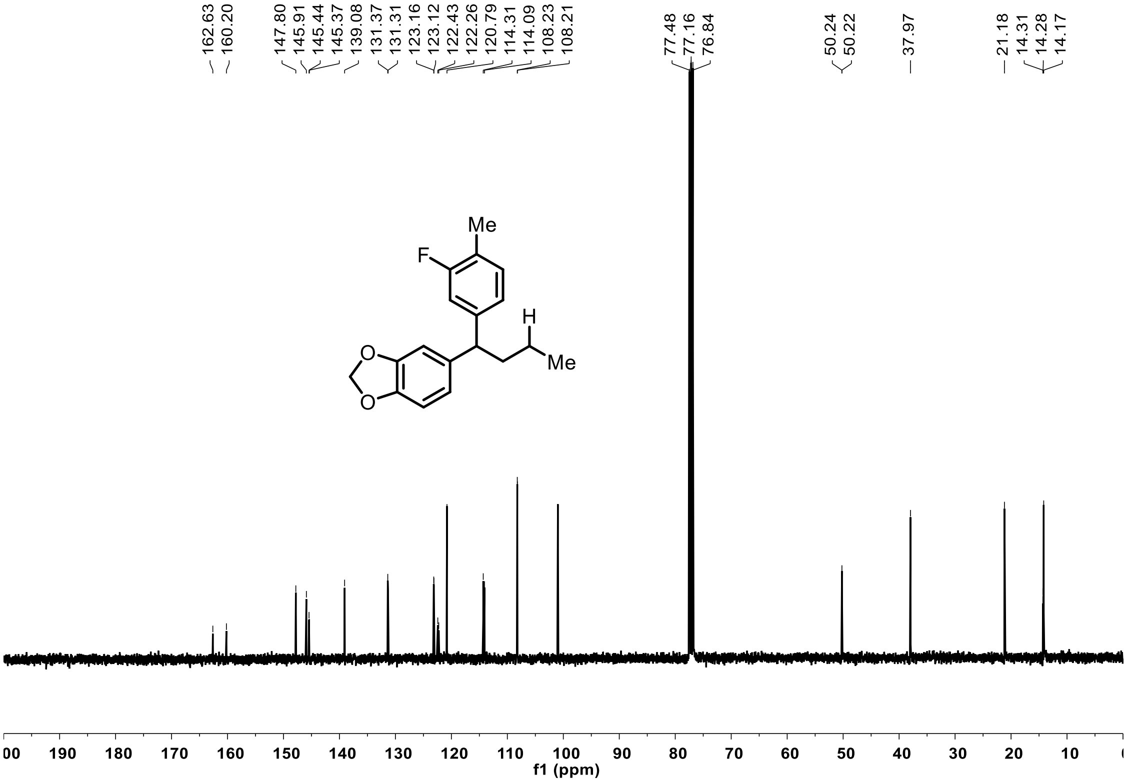

Supplementary Figure 192. ^{13}C NMR Spectrum of 3ar

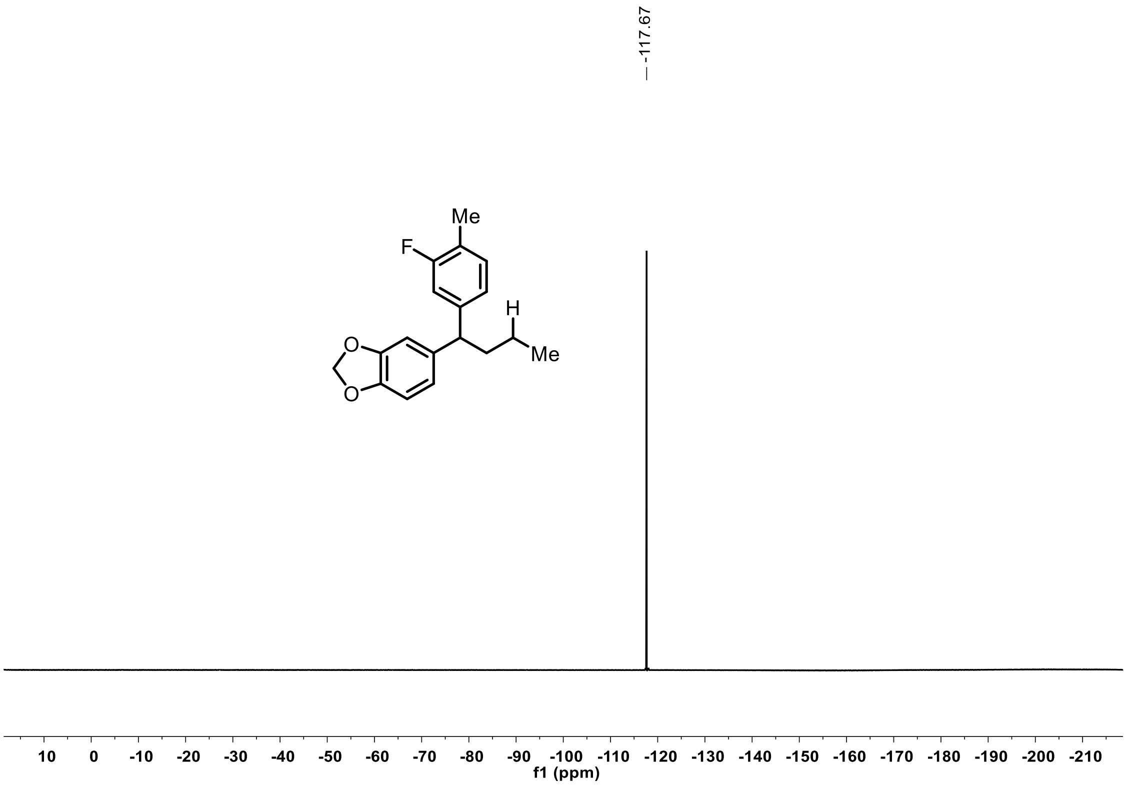

Supplementary Figure 193. ^{19}F NMR Spectrum of 3ar

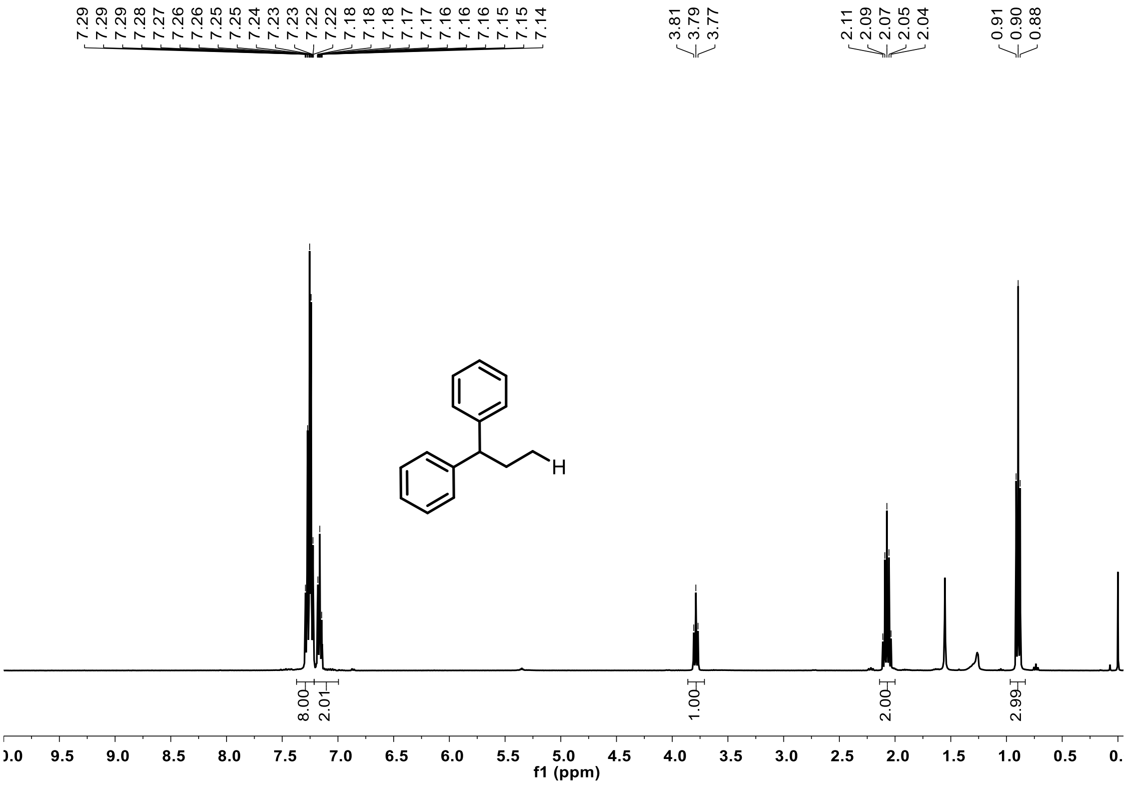

Supplementary Figure 194. ^{1}H NMR Spectrum of 3as

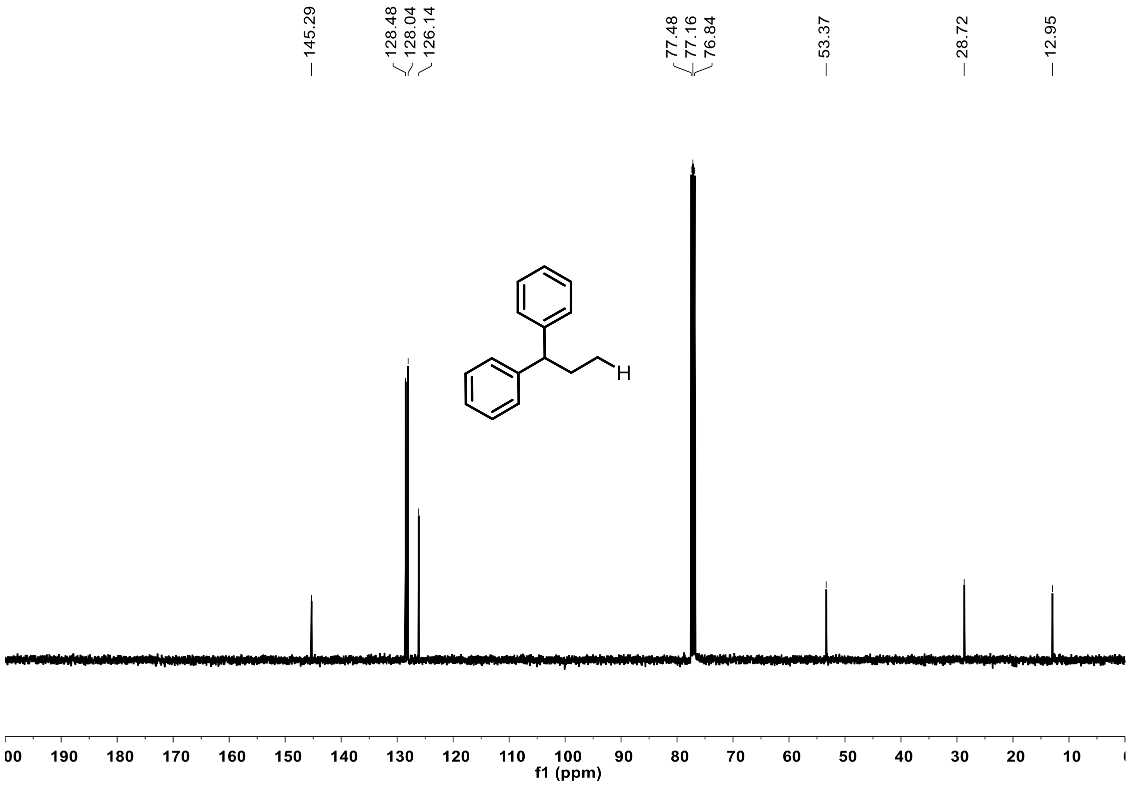

Supplementary Figure 195. ^{13}C NMR Spectrum of 3as

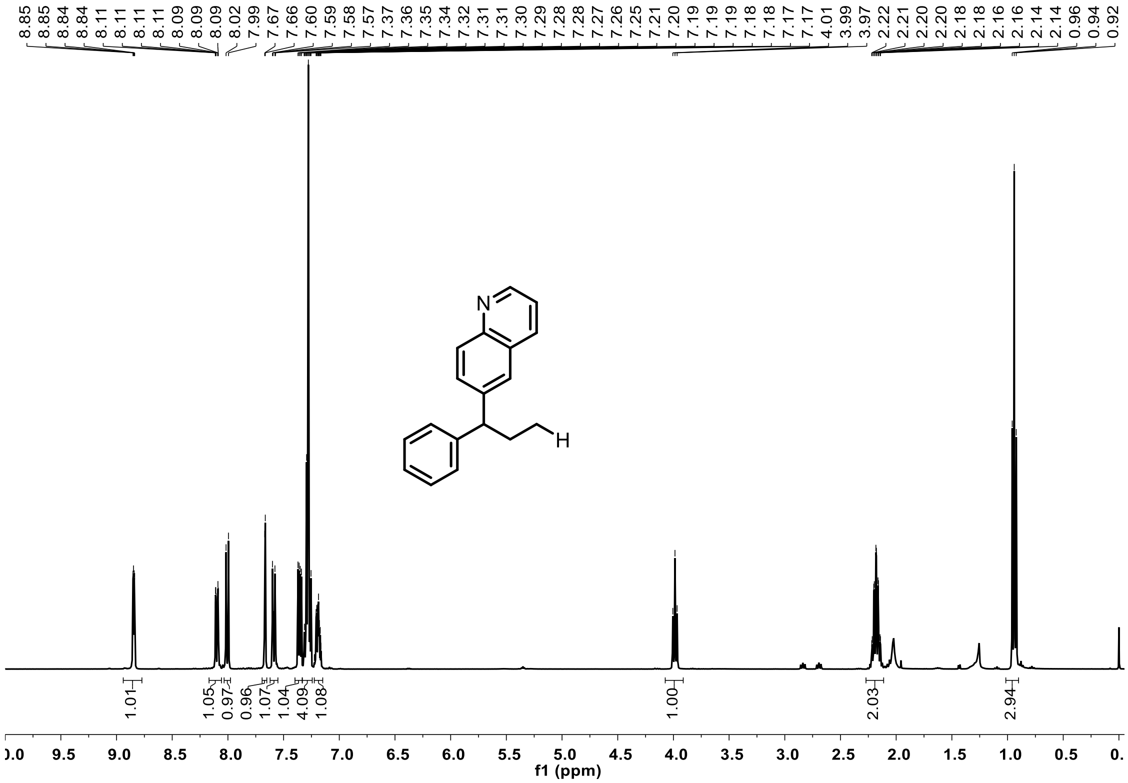

Supplementary Figure 196. ^{1}H NMR Spectrum of 3at

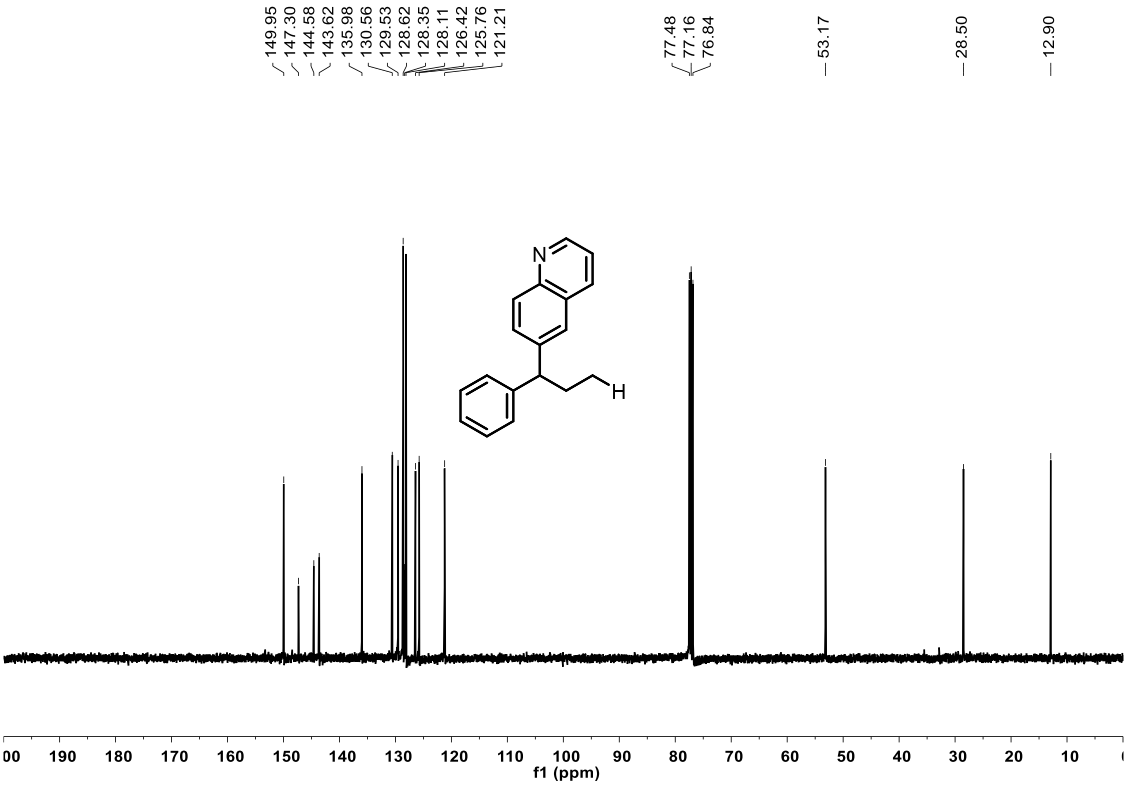

Supplementary Figure 197. ^{13}C NMR Spectrum of 3at

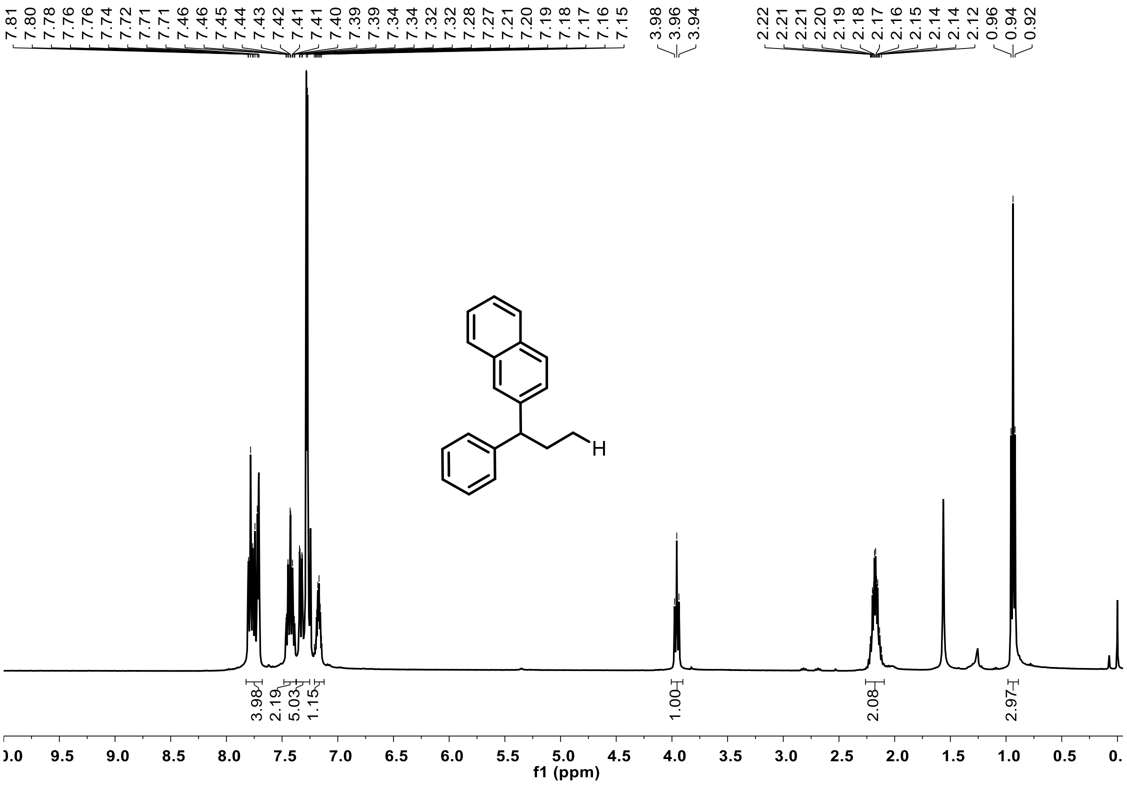

Supplementary Figure 198. ^{1}H NMR Spectrum of 3au

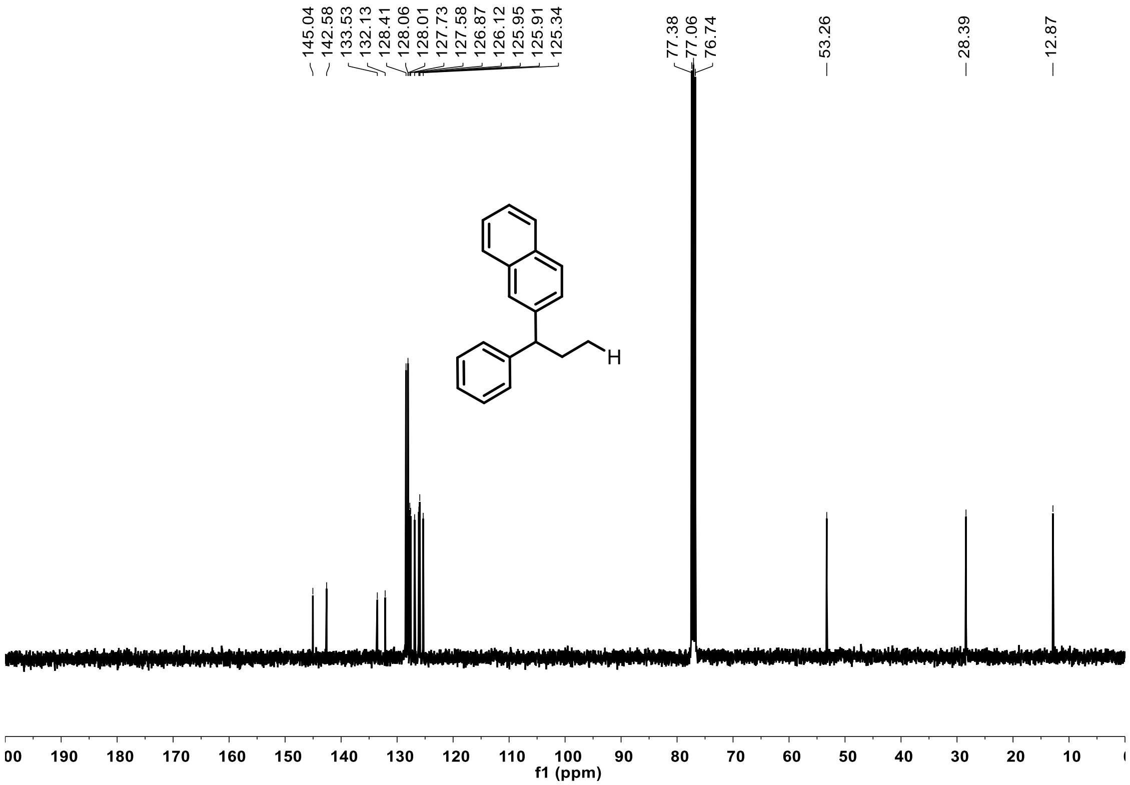

Supplementary Figure 199. ^{13}C NMR Spectrum of 3au

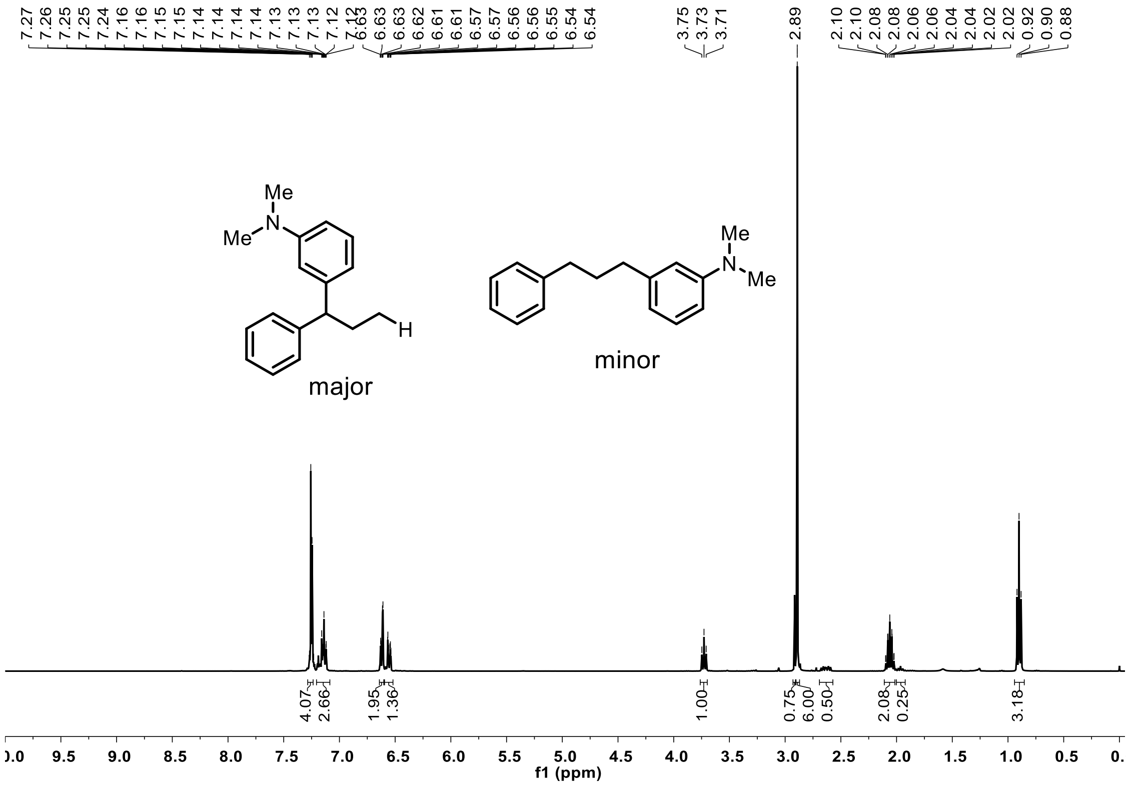

Supplementary Figure 200. ^{1}H NMR Spectrum of 3av + 4av

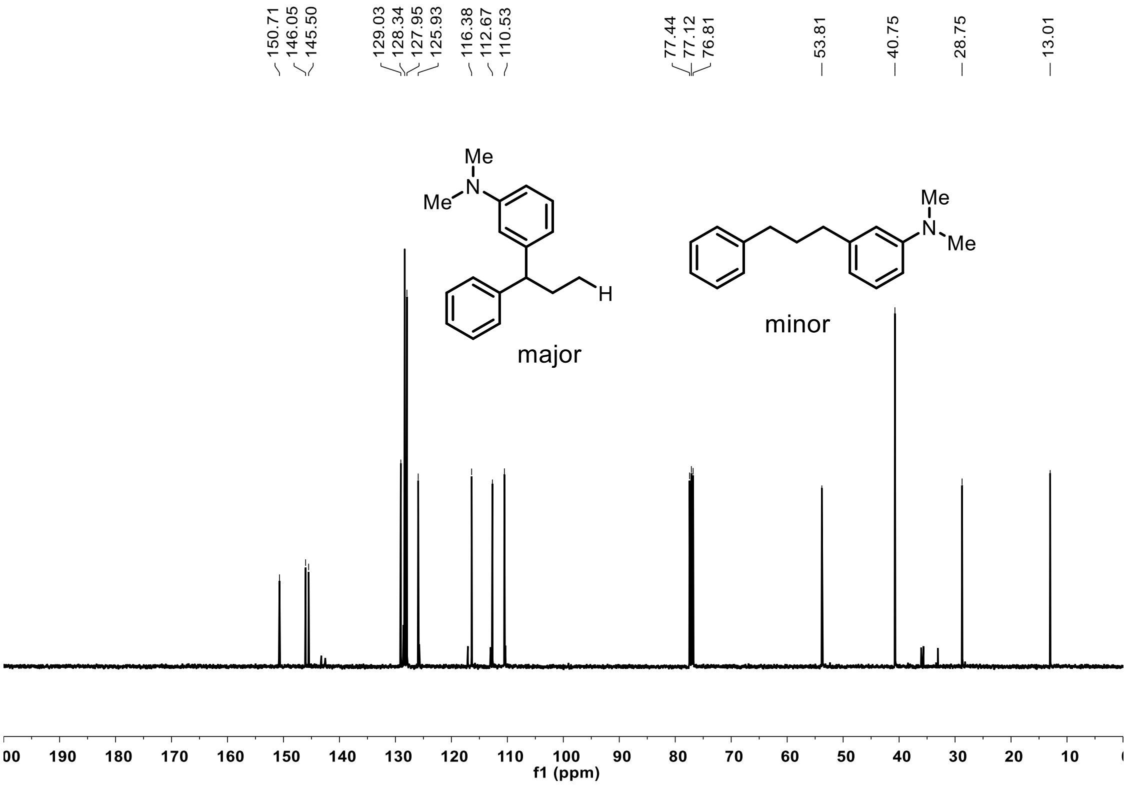

Supplementary Figure 201. ^{13}C NMR Spectrum of 3av + 4av

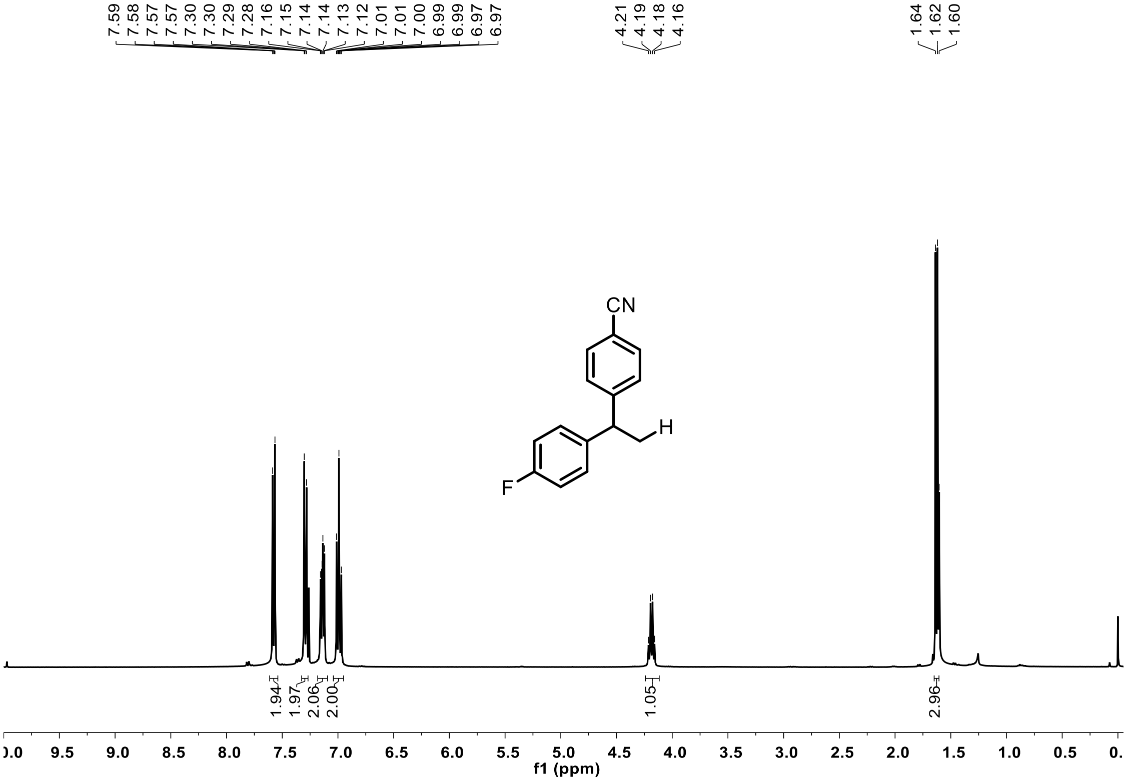

Supplementary Figure 202. ^{1}H NMR Spectrum of 3aw

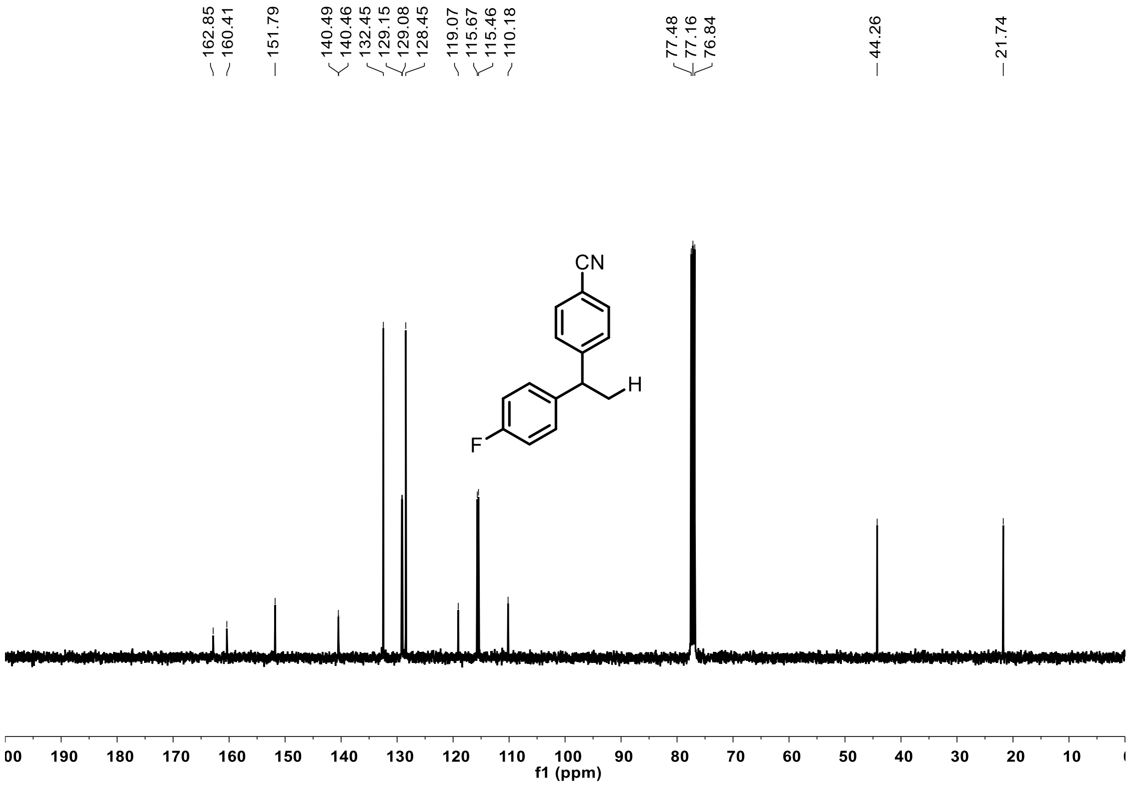

Supplementary Figure 203. ^{13}C NMR Spectrum of 3aw

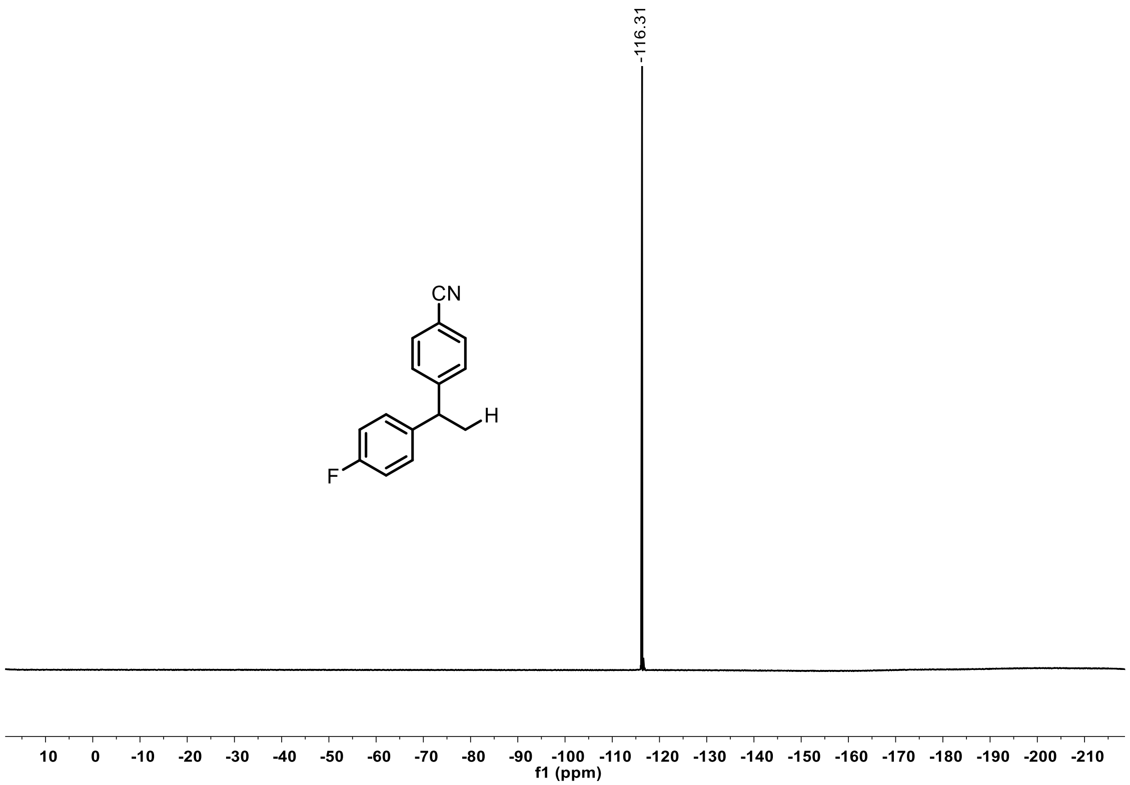

Supplementary Figure 204. ^{19}F NMR Spectrum of 3aw

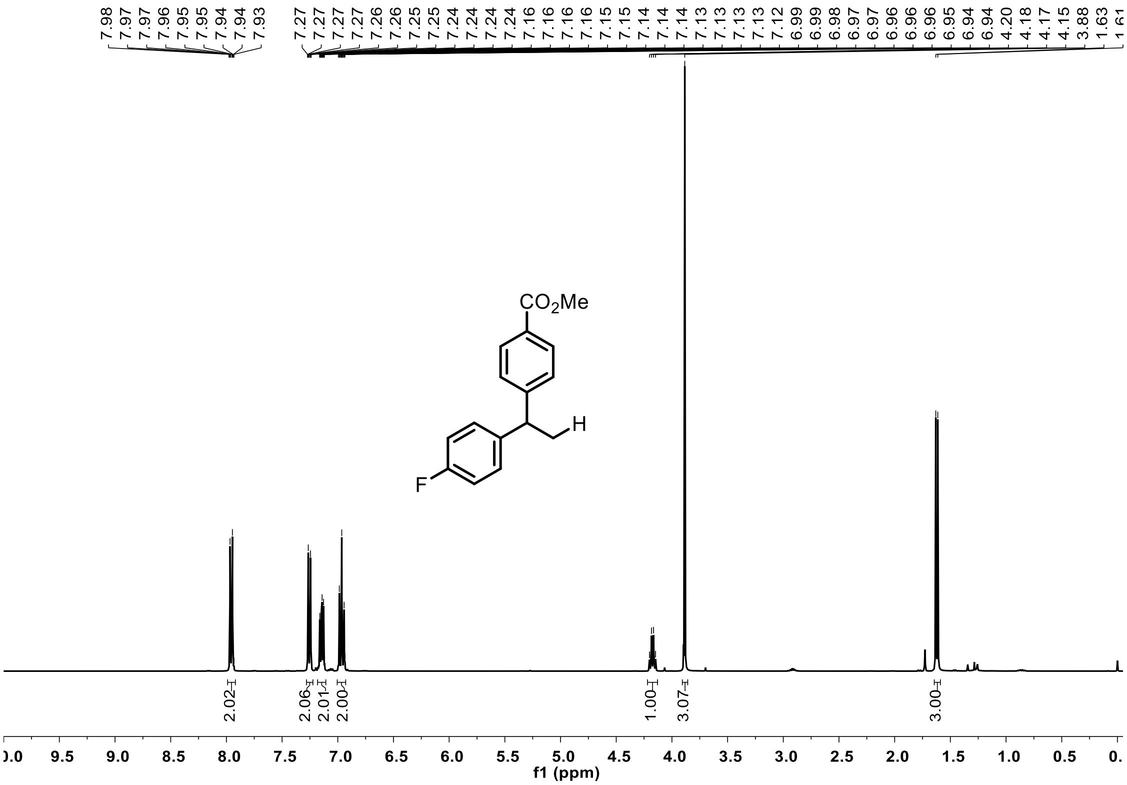

Supplementary Figure 205. ^{1}H NMR Spectrum of 3ax

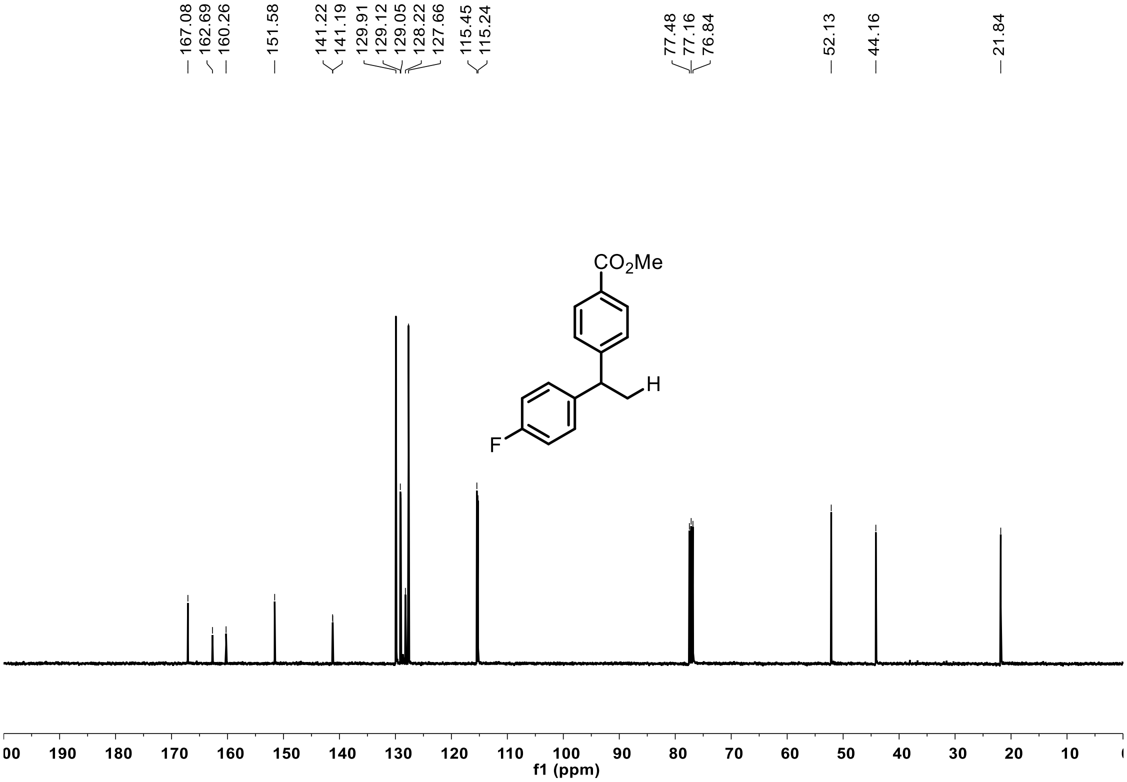

Supplementary Figure 206. ^{13}C NMR Spectrum of 3ax

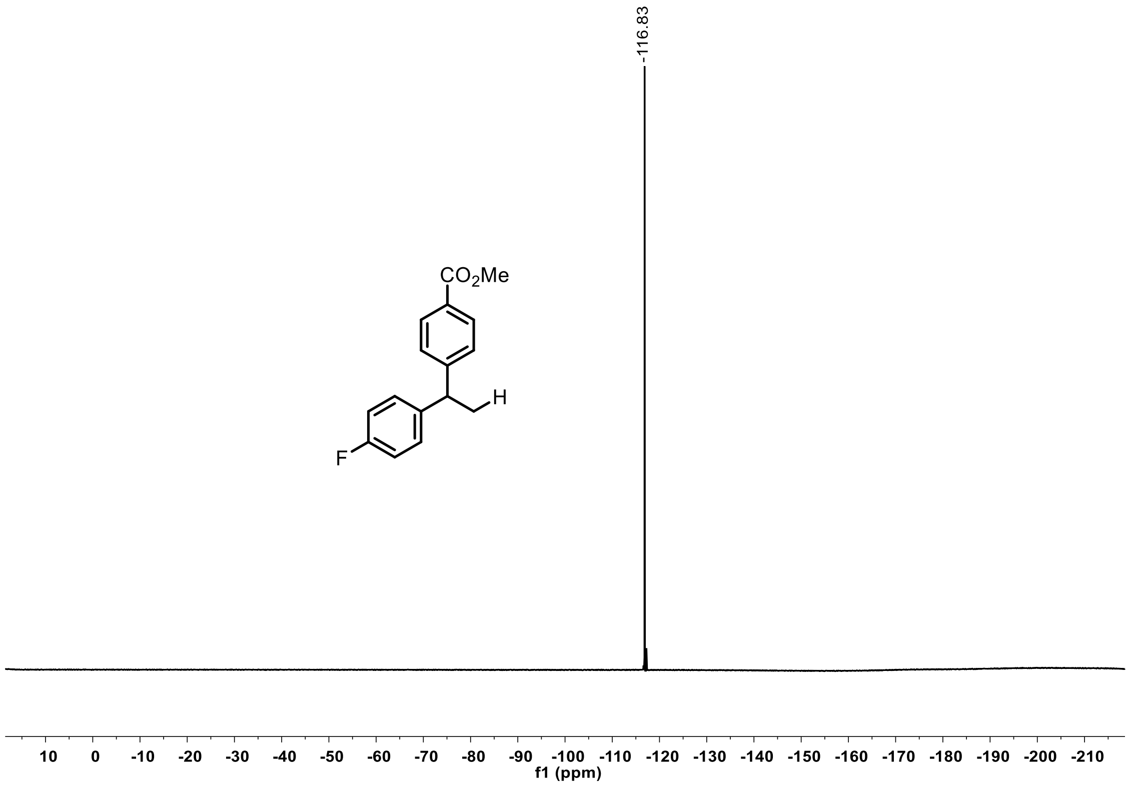

Supplementary Figure 207. ^{19}F NMR Spectrum of 3ax

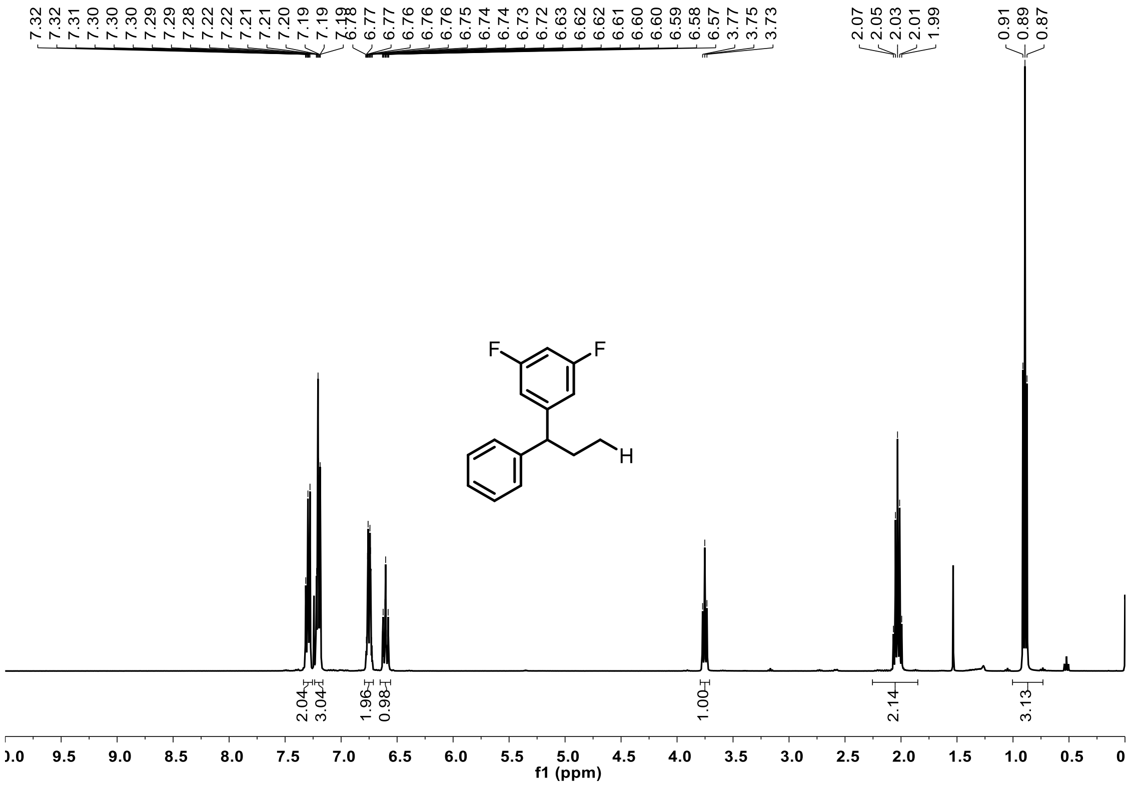

Supplementary Figure 208. ^{1}H NMR Spectrum of 3ay

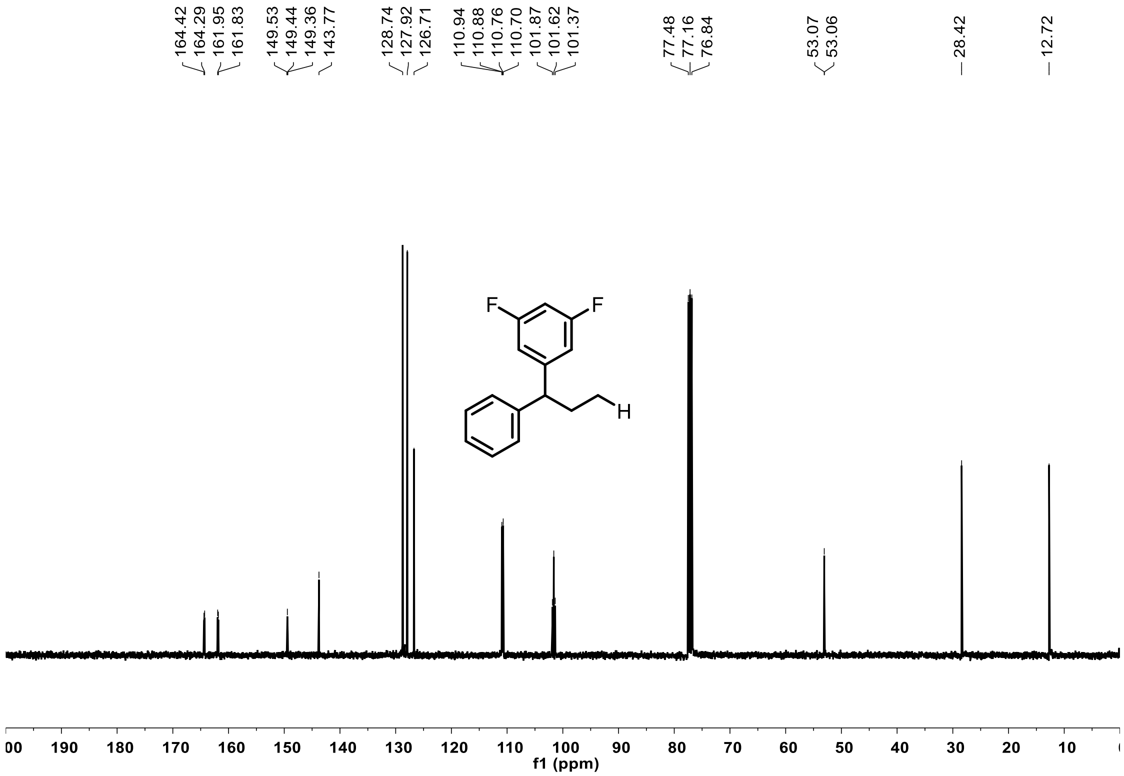

Supplementary Figure 209. ^{13}C NMR Spectrum of 3ay

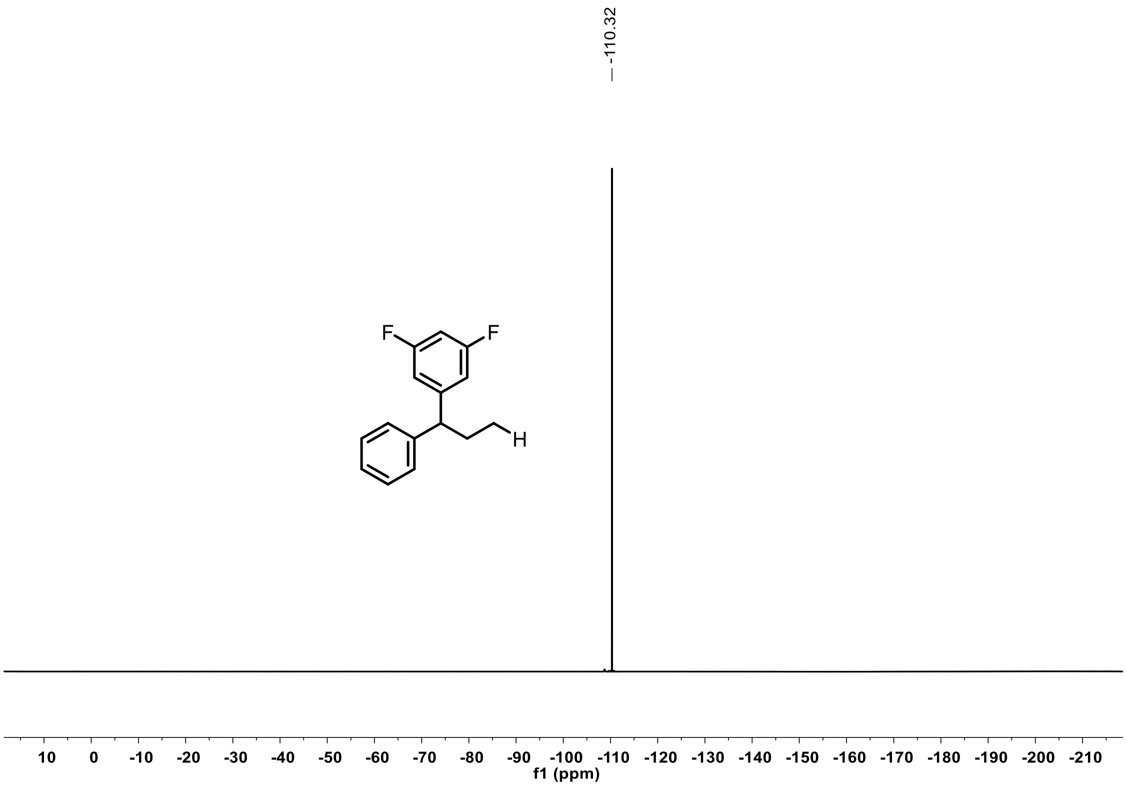

Supplementary Figure 210. ^{19}F NMR Spectrum of 3ay

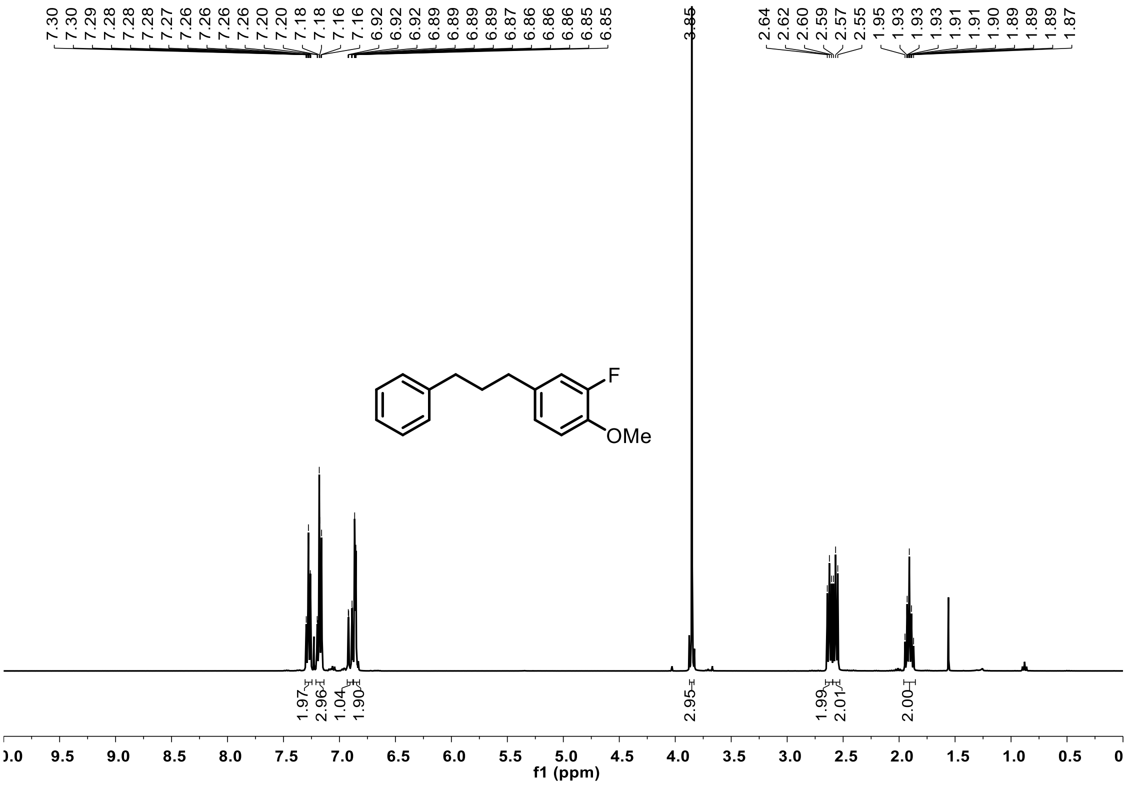

Supplementary Figure 211. ^{1}H NMR Spectrum of 4a

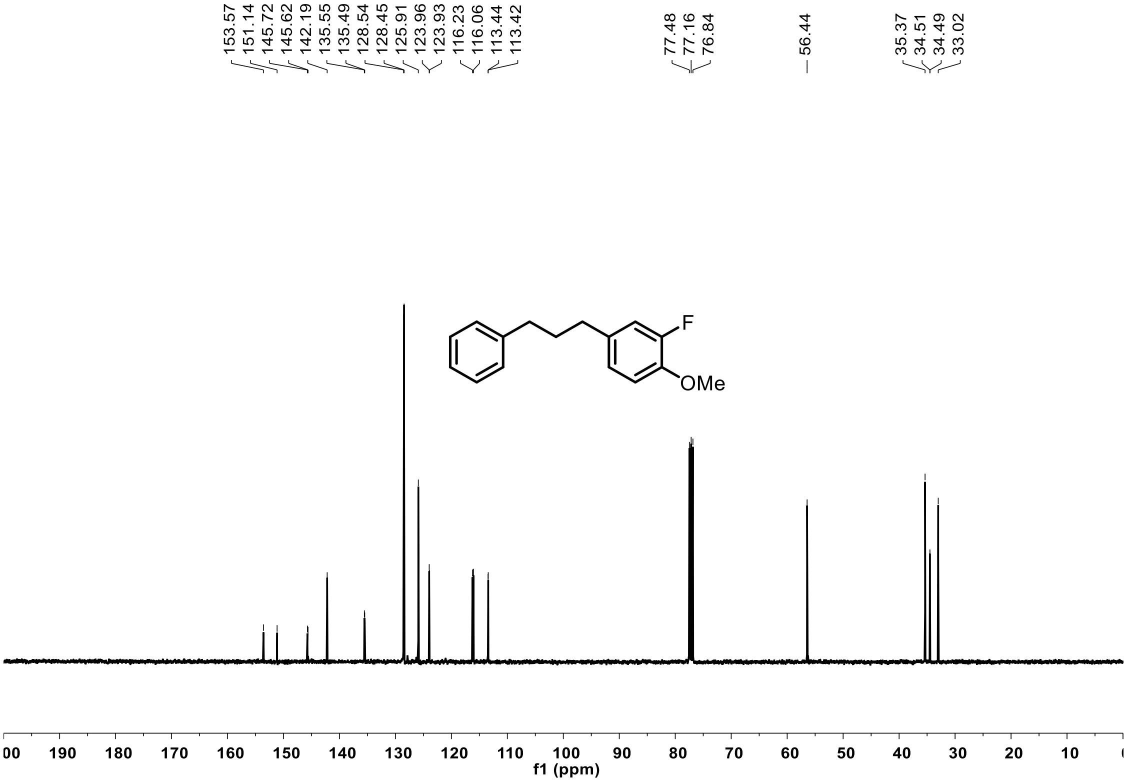

Supplementary Figure 212. ^{13}C NMR Spectrum of 4a

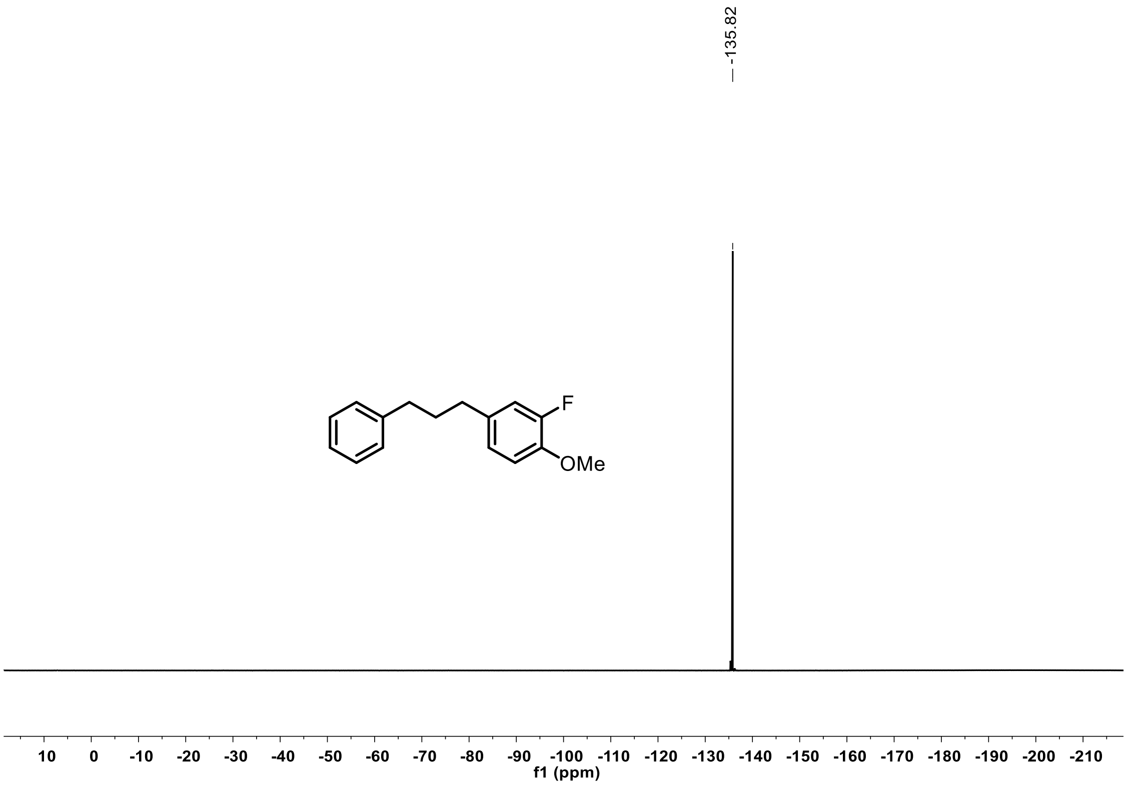

Supplementary Figure 213. ^{19}F NMR Spectrum of 4a

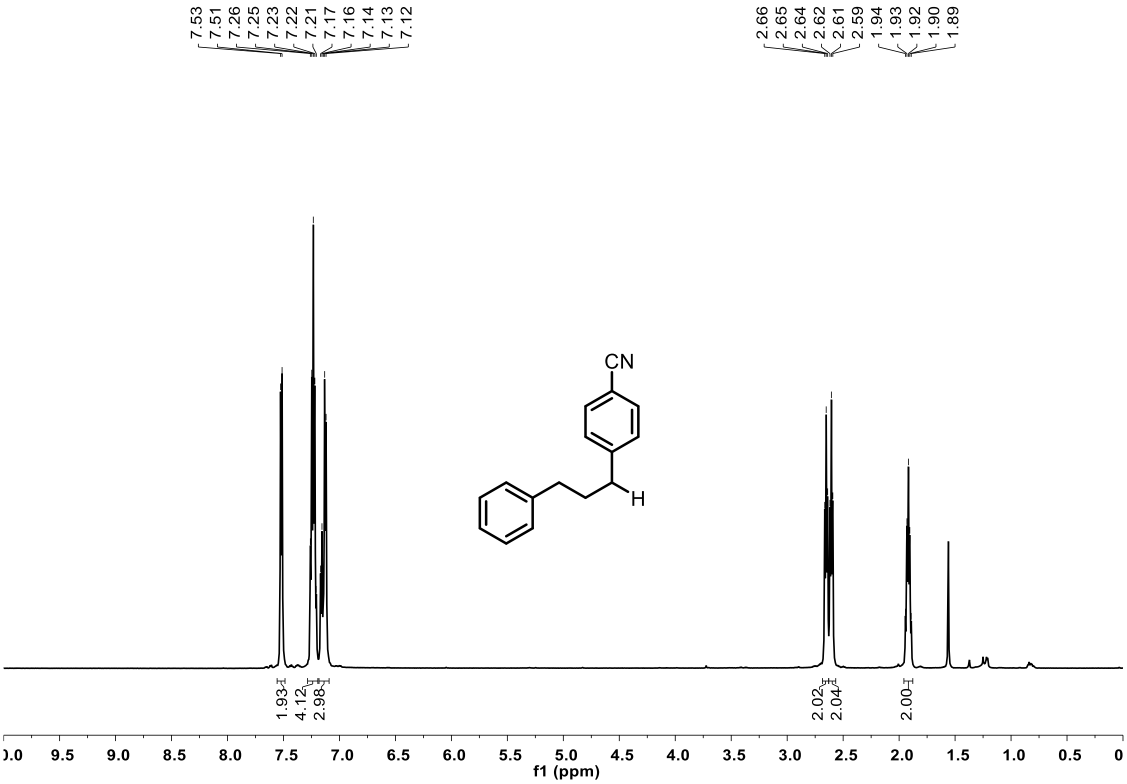

Supplementary Figure 214. ^{1}H NMR Spectrum of 4aj

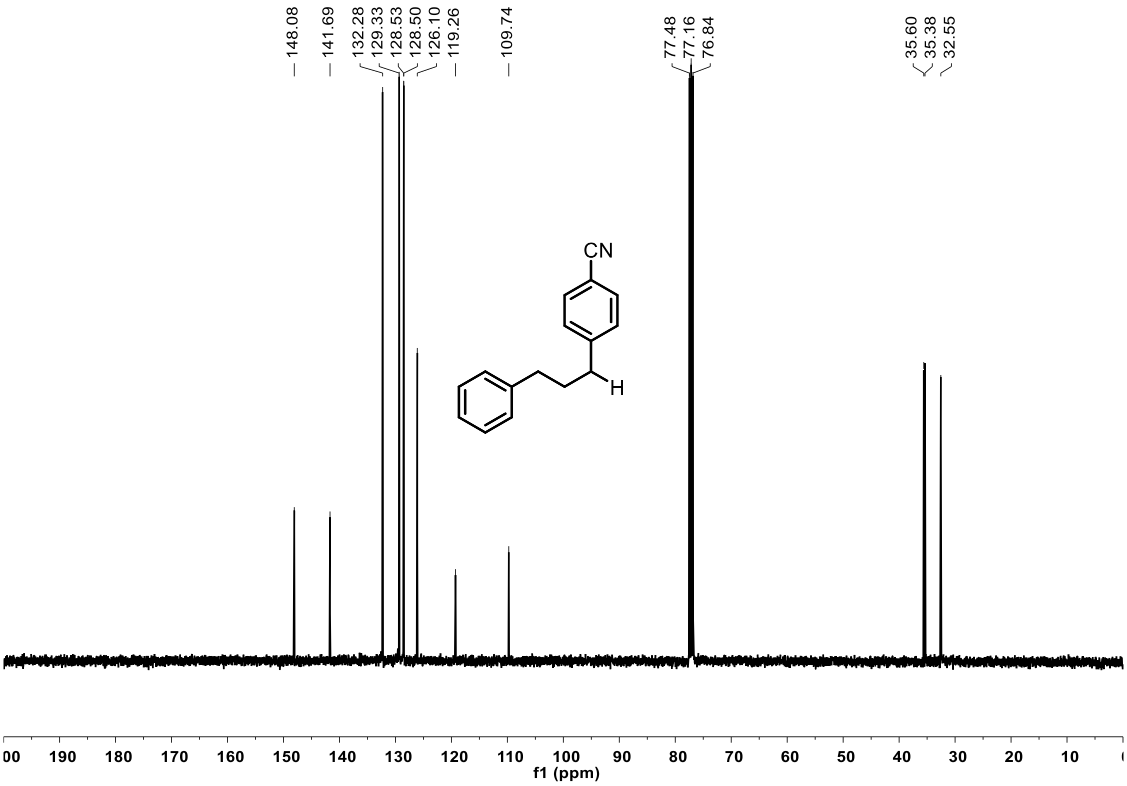

Supplementary Figure 215. ^{13}C NMR Spectrum of 4aj

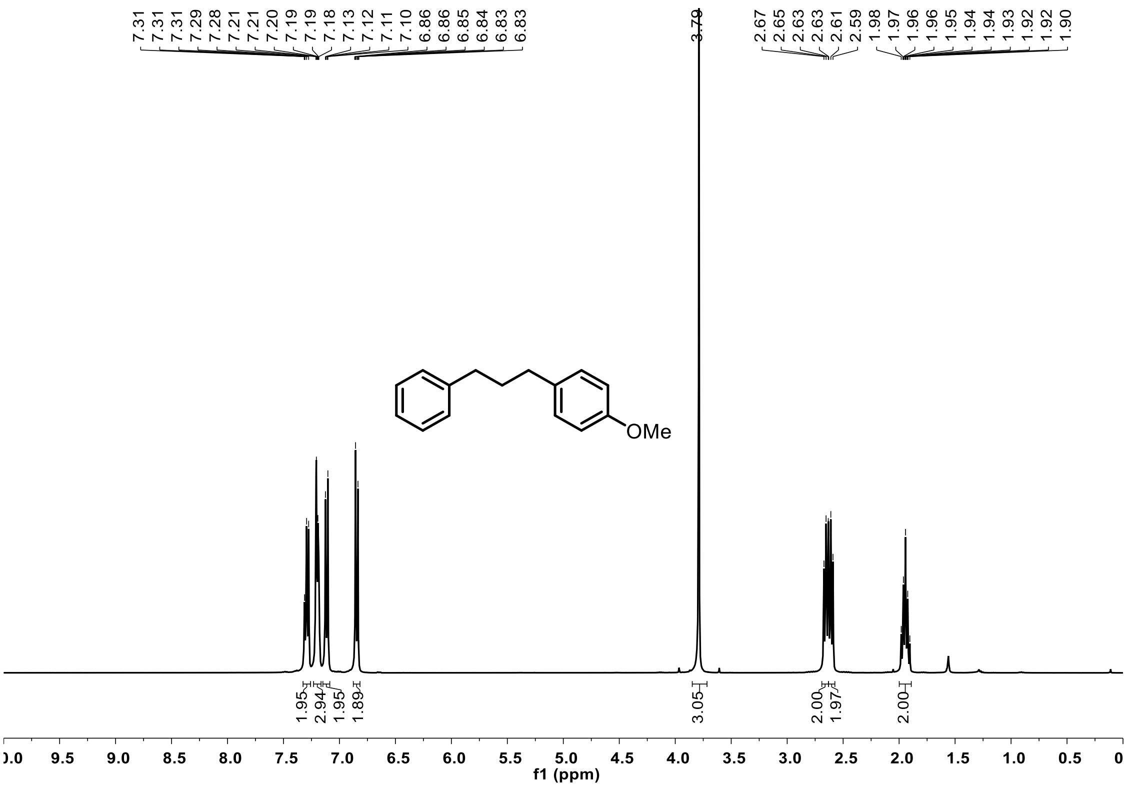

Supplementary Figure 216. ^{1}H NMR Spectrum of 4ah

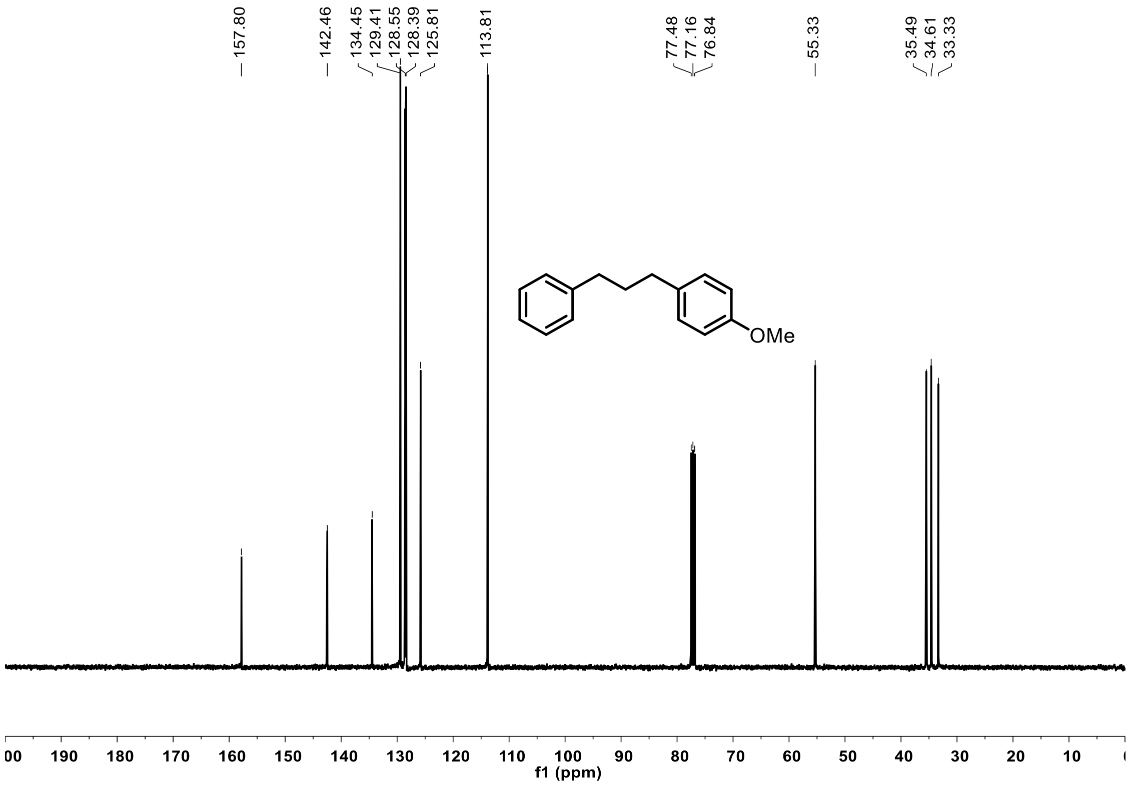

Supplementary Figure 217. ^{13}C NMR Spectrum of 4ah

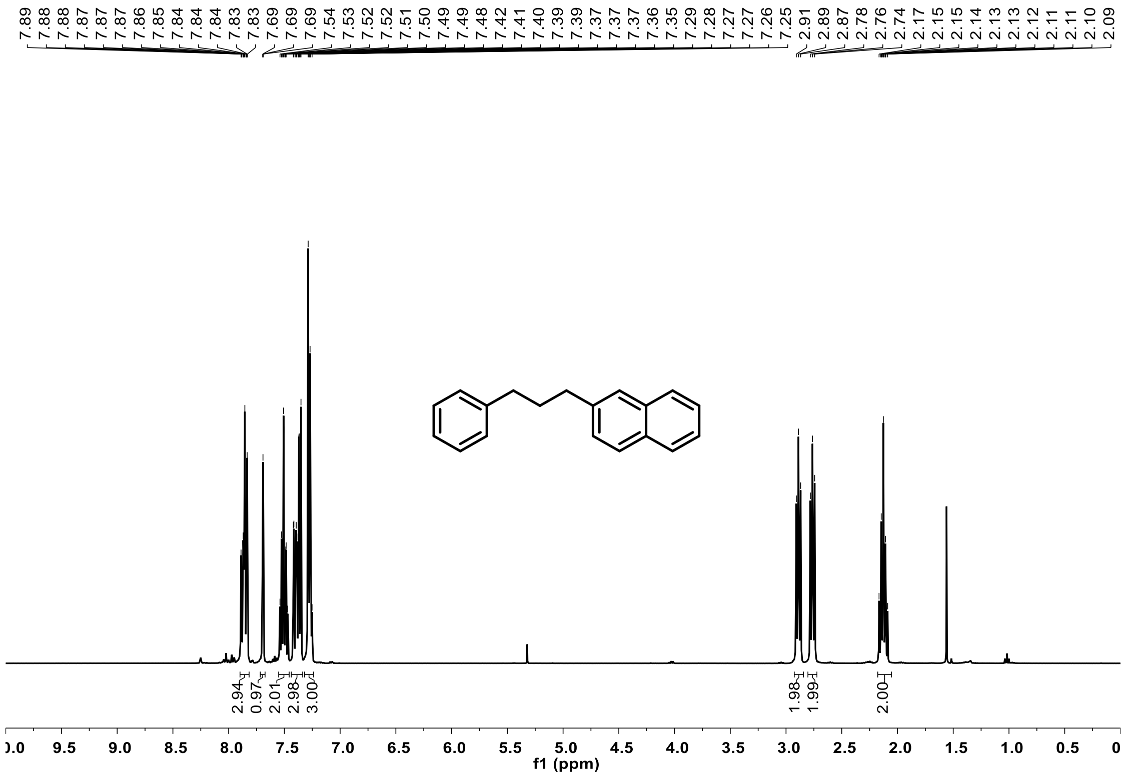

Supplementary Figure 218. ^{1}H NMR Spectrum of 4au

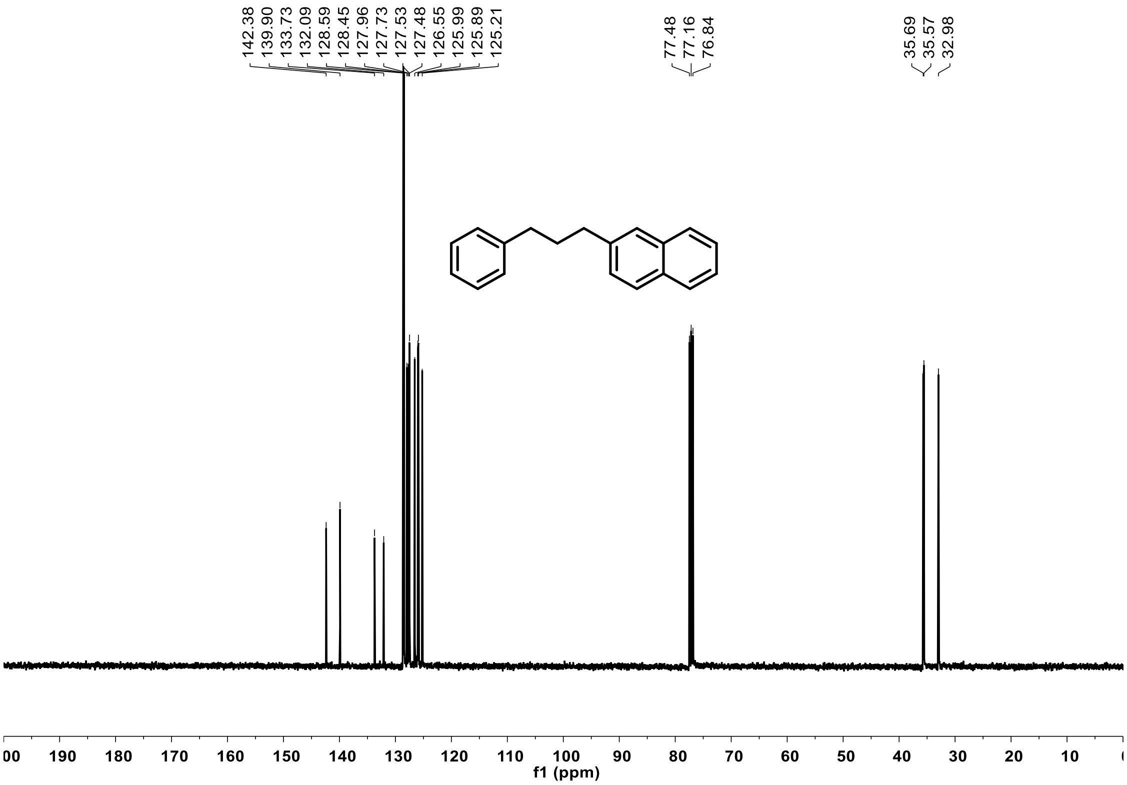

Supplementary Figure 219. ^{13}C NMR Spectrum of 4au

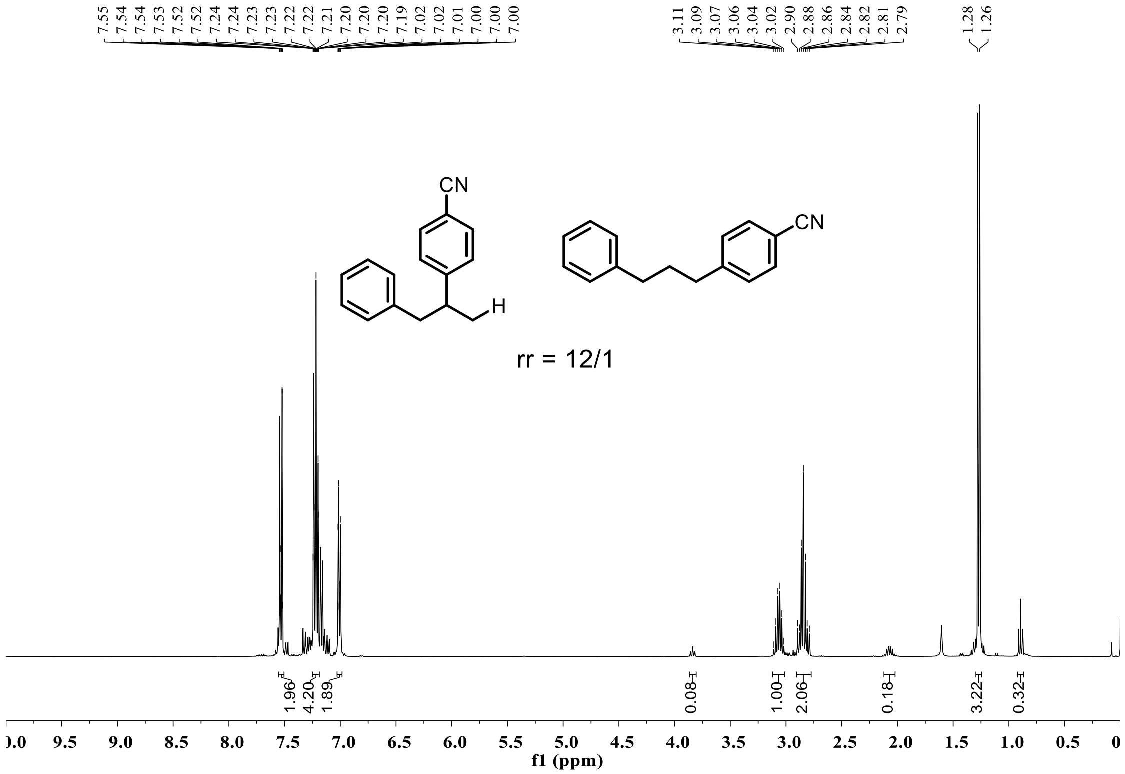

Supplementary Figure 220. ^{1}H NMR Spectrum of 4az

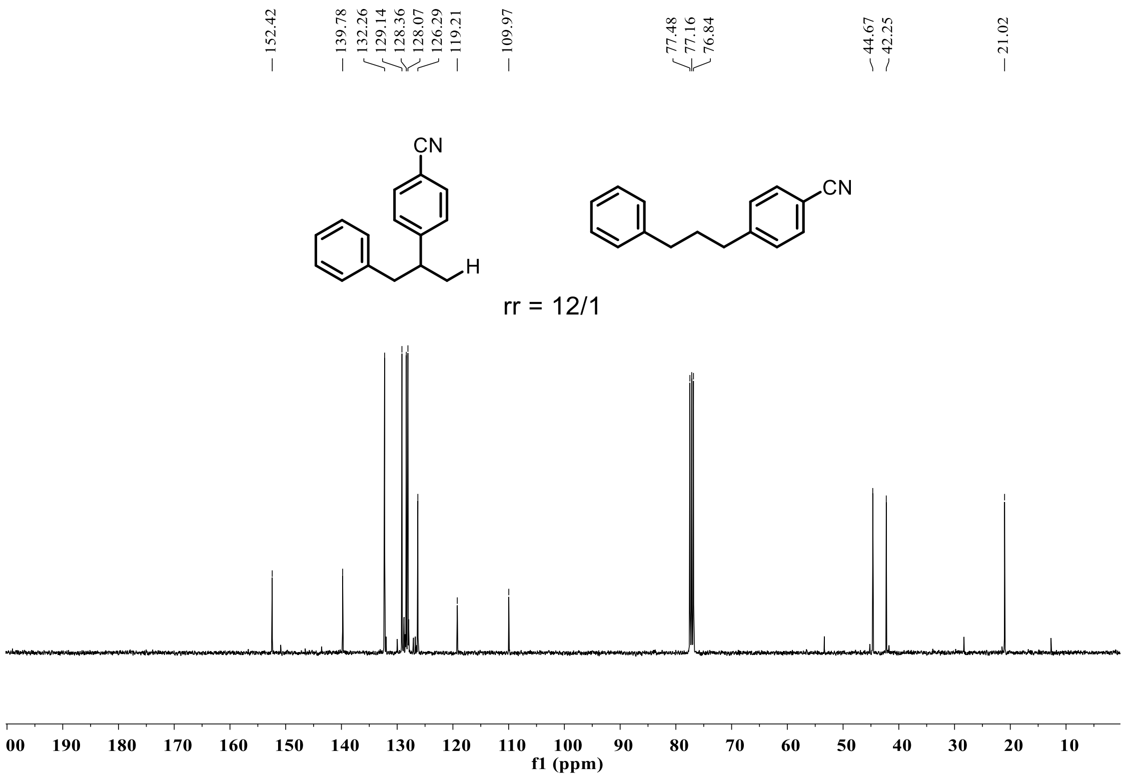

Supplementary Figure 221. ^{13}C NMR Spectrum of 4az

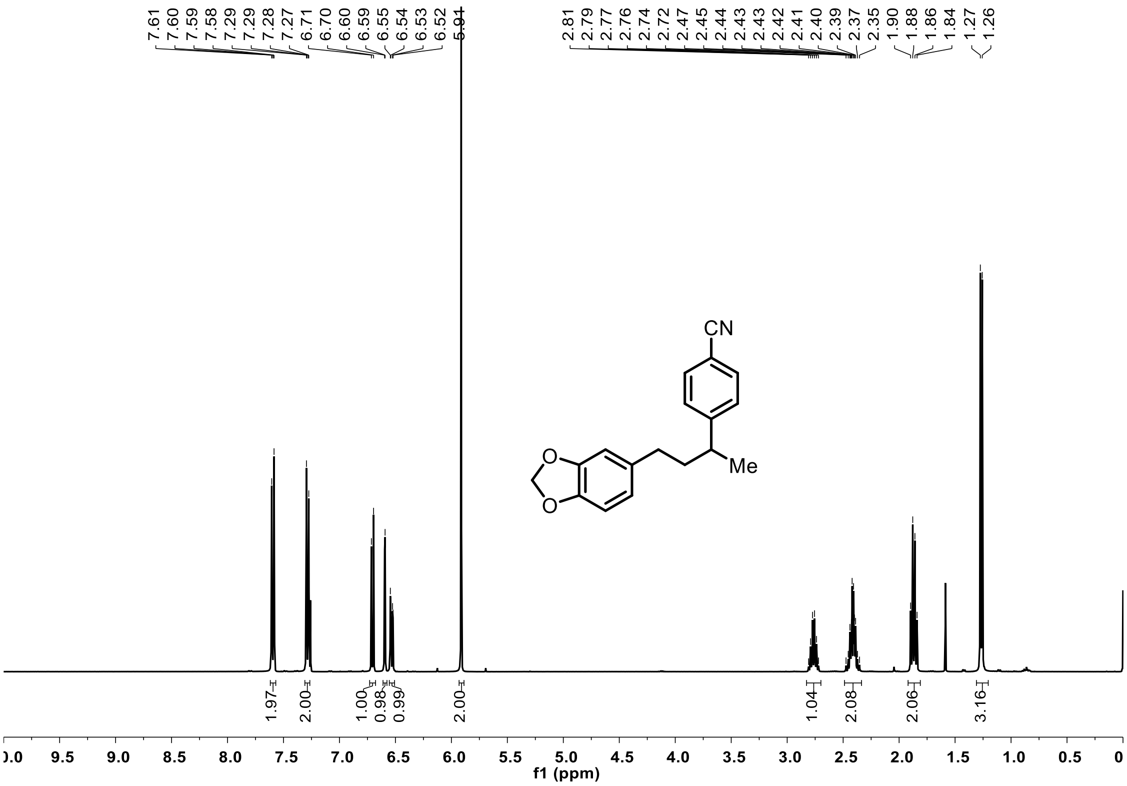

Supplementary Figure 222. ^{1}H NMR Spectrum of 4ba

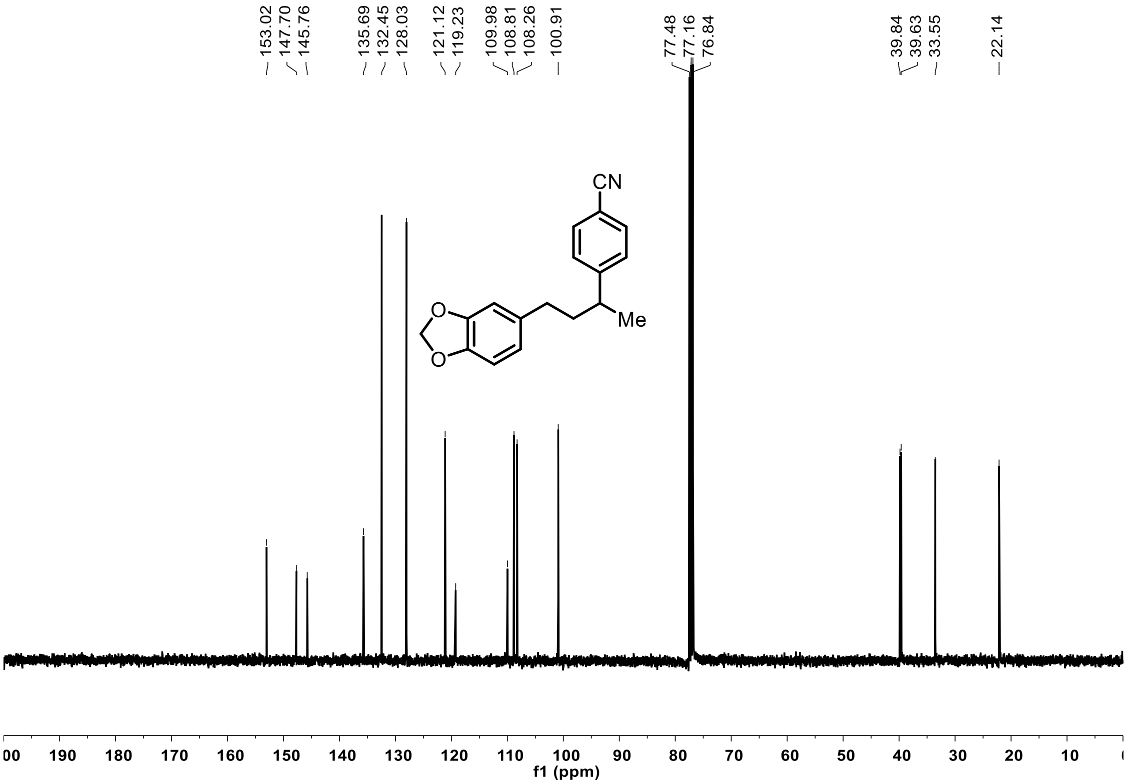

Supplementary Figure 223. ^{13}C NMR Spectrum of 4ba

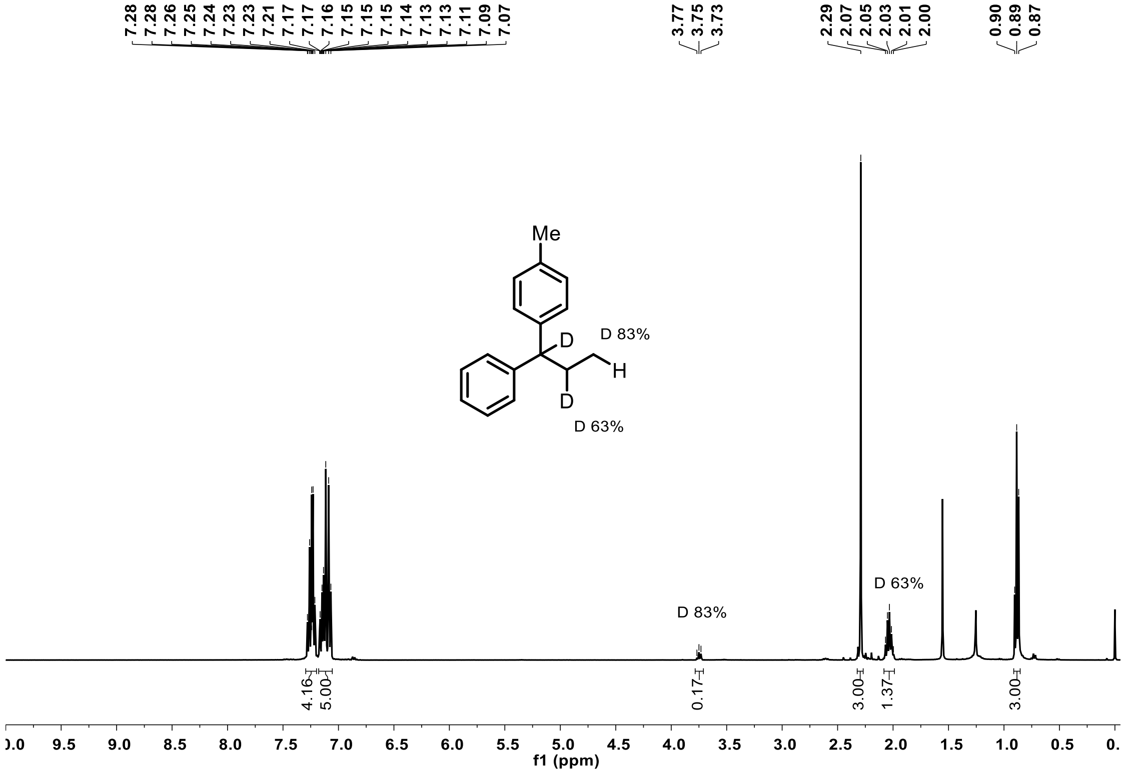

Supplementary Figure 224. ^{1}H NMR Spectrum of 3bb-D_2

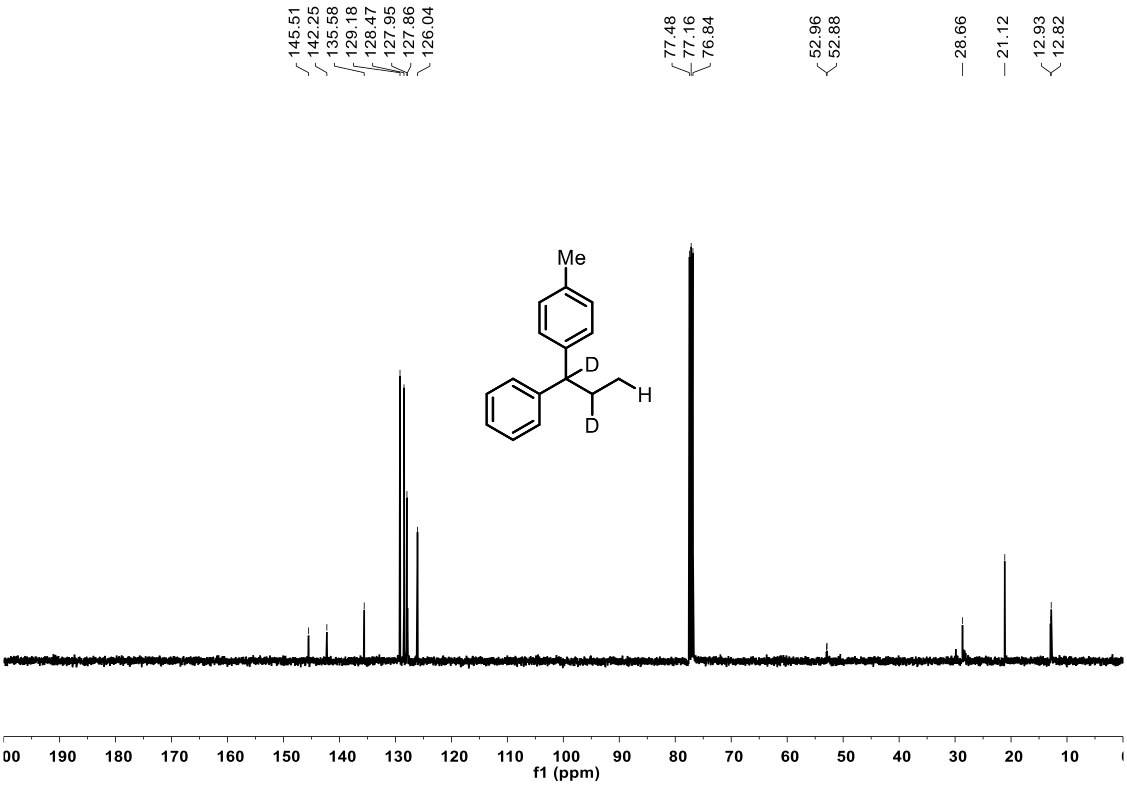

Supplementary Figure 225. ^{13}C NMR Spectrum of 3bb-D_2

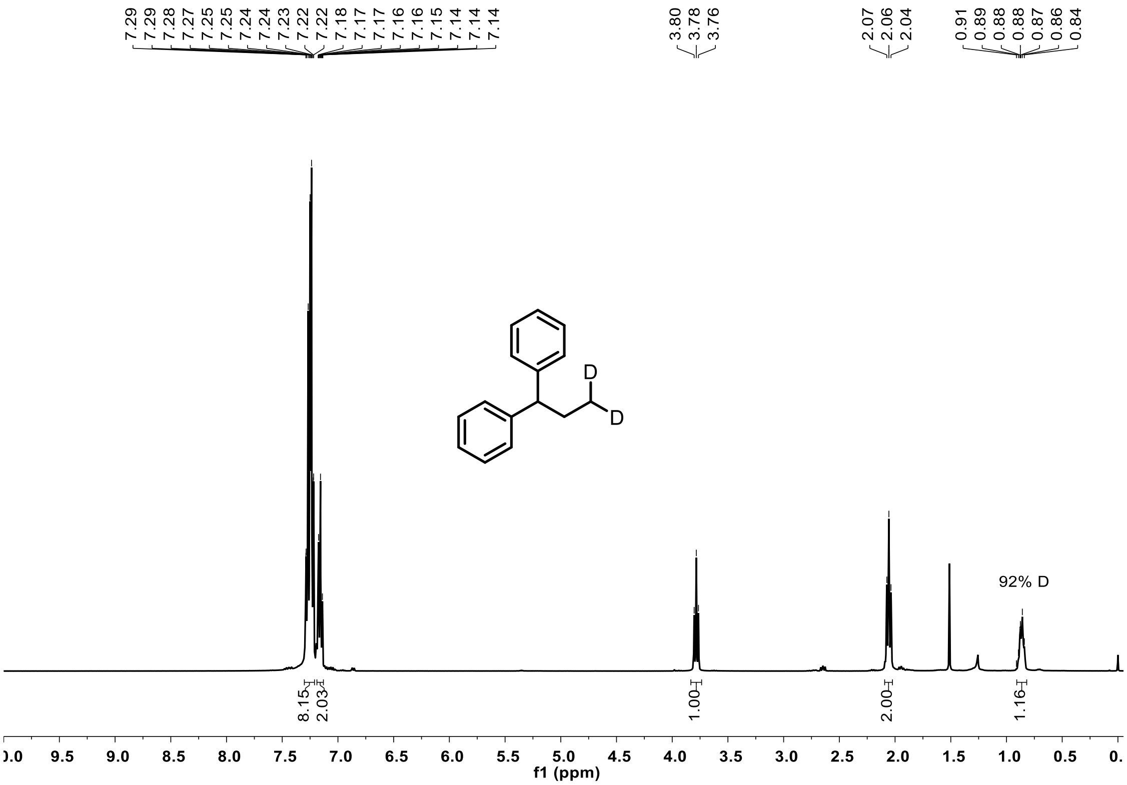

Supplementary Figure 226. ^{1}H NMR Spectrum of 3as-D_2’

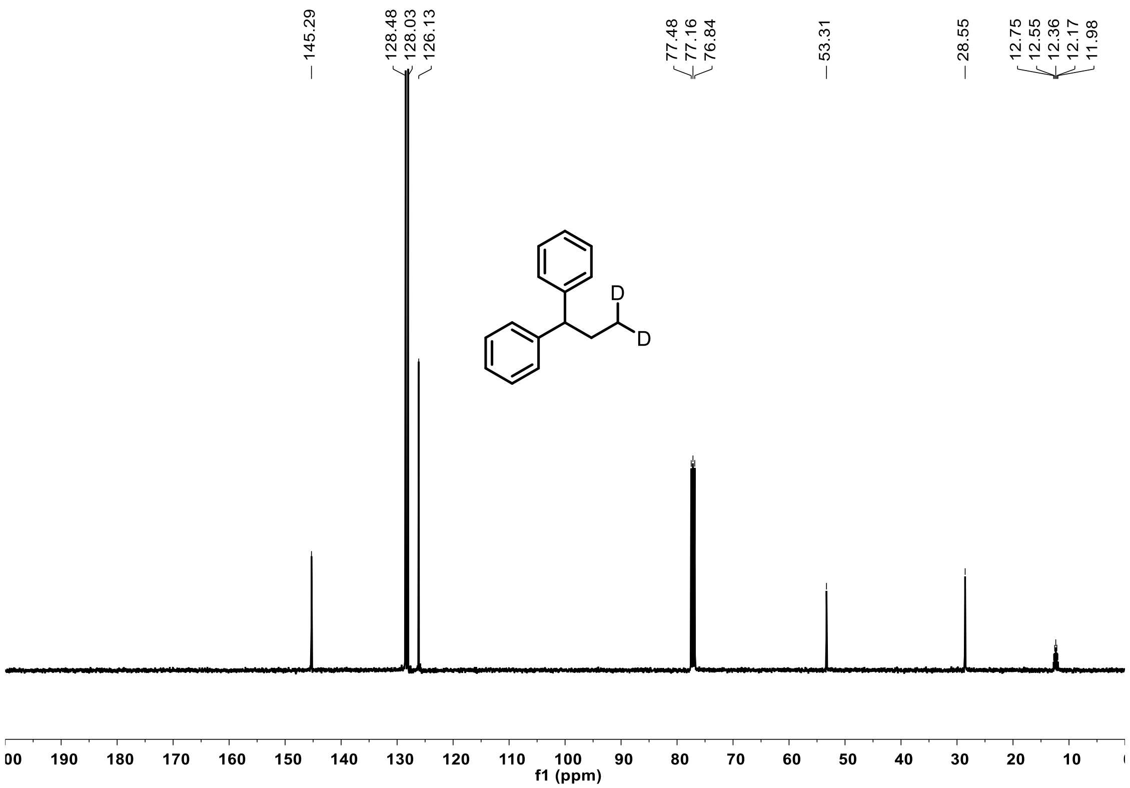

Supplementary Figure 227. ^{13}C NMR Spectrum of 3as-D_2’

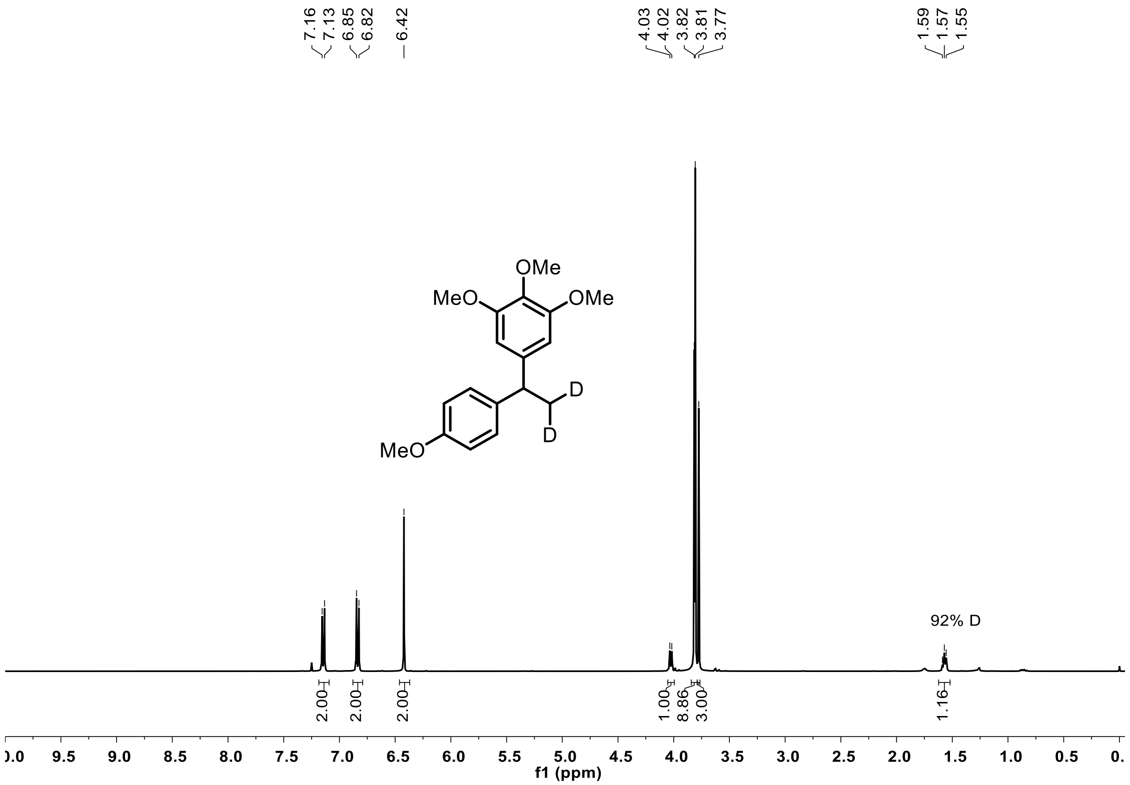

Supplementary Figure 228. ^{1}H NMR Spectrum of 3bc-D_2’

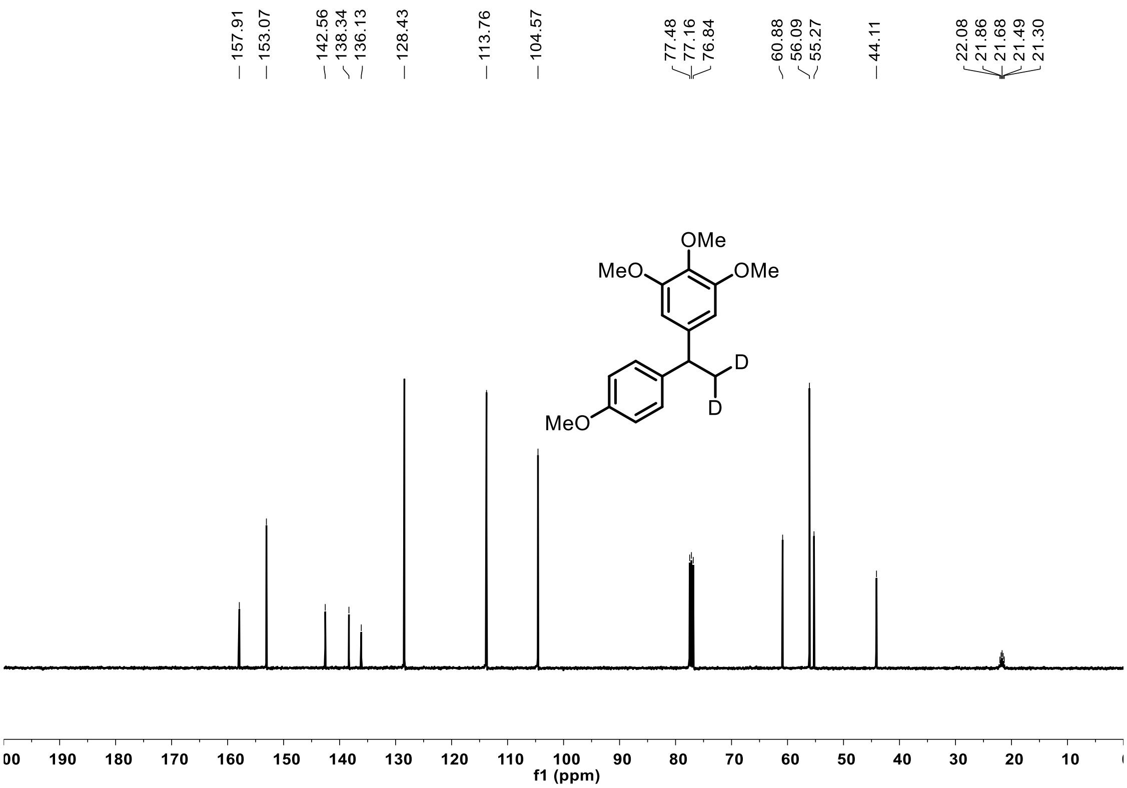

Supplementary Figure 229. ^{13}C NMR Spectrum of 3bc-D_2'

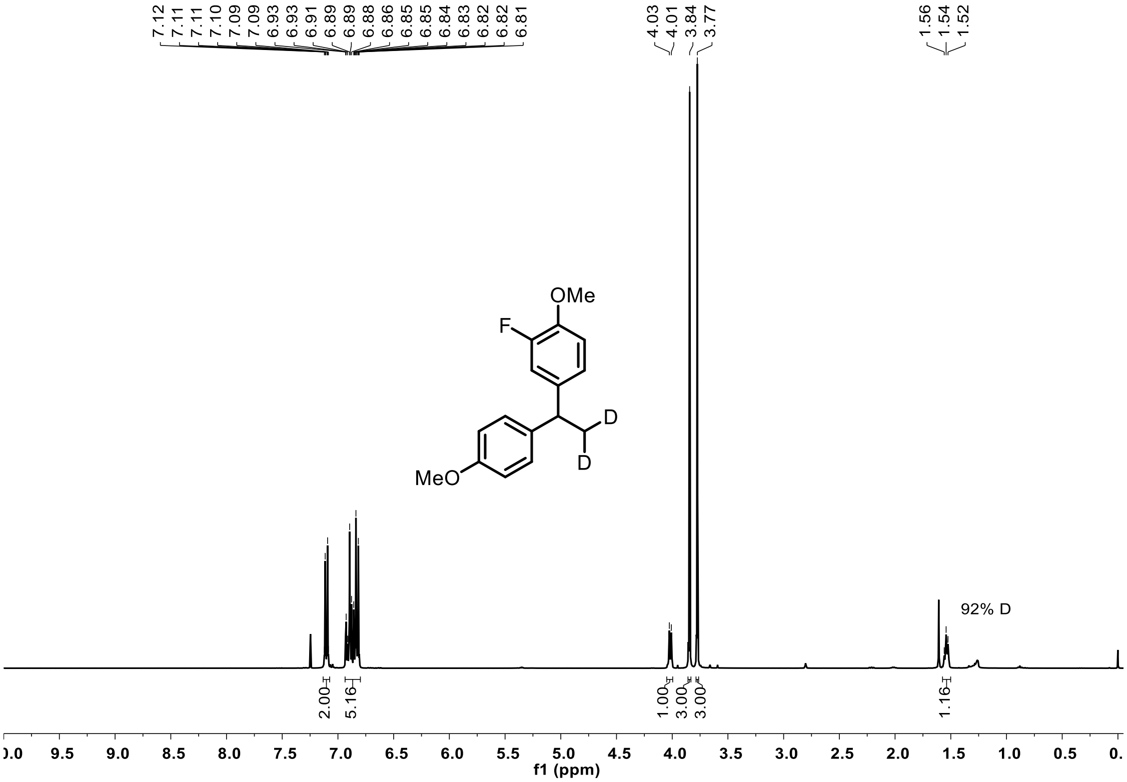

Supplementary Figure 230. ^{1}H NMR Spectrum of 3bf-D_2'

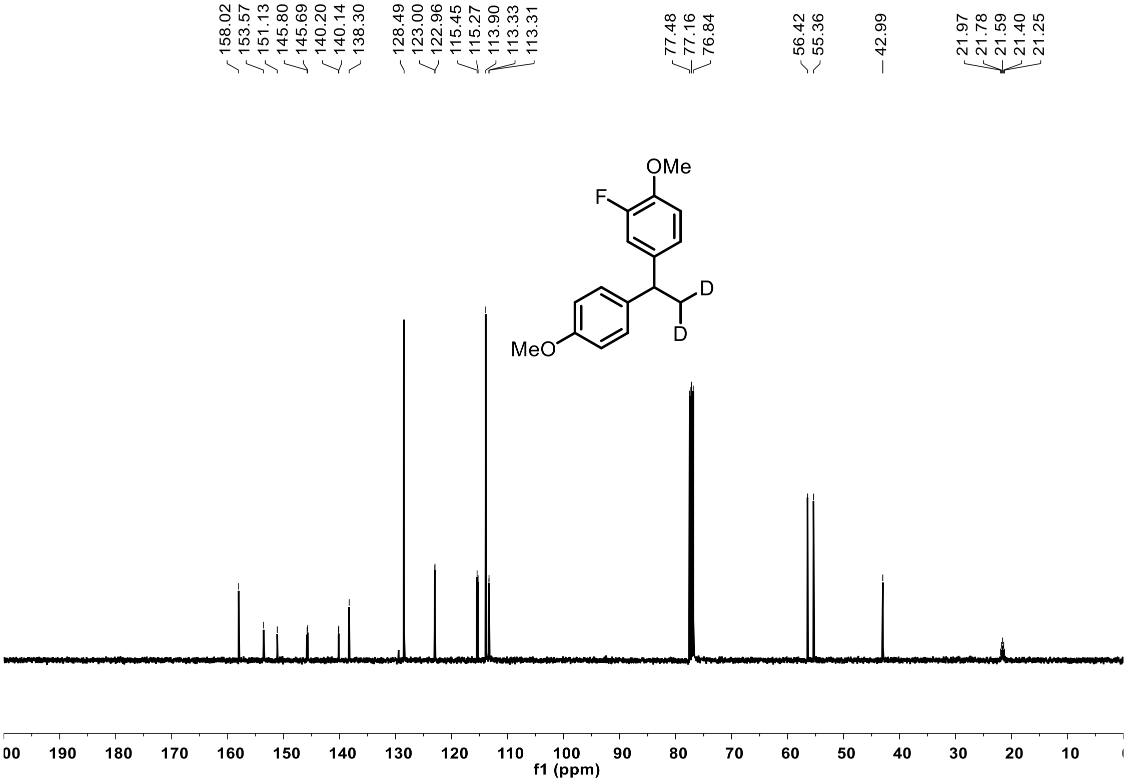

Supplementary Figure 231. ^{13}C NMR Spectrum of 3bf-D_2'

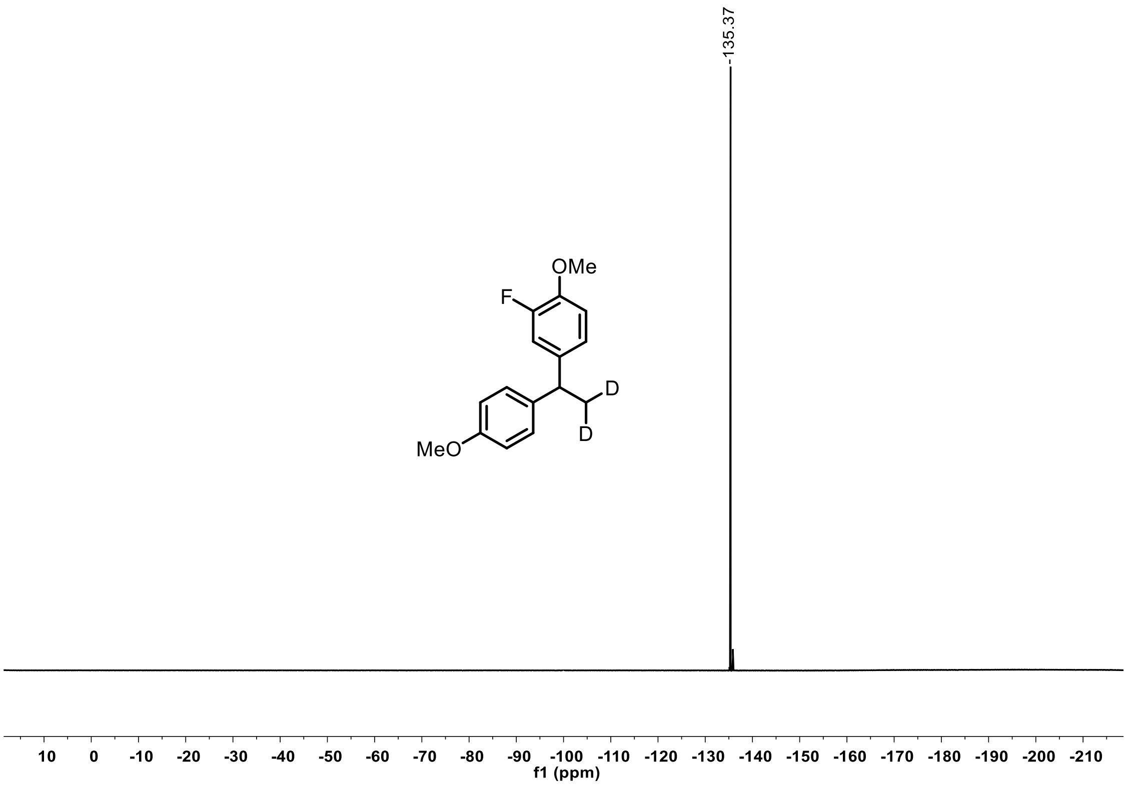

Supplementary Figure 232. ^{19}F NMR Spectrum of 3bf-D_2'

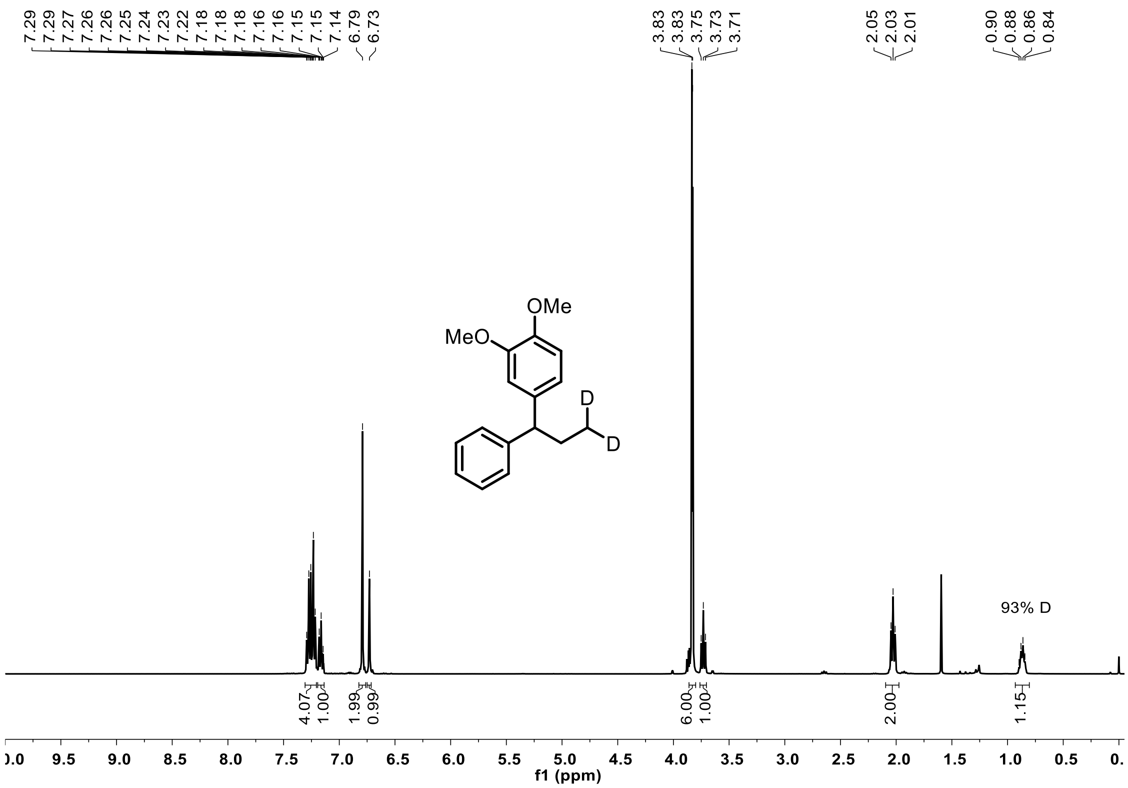

Supplementary Figure 233. ^{1}H NMR Spectrum of 3b-D_2’

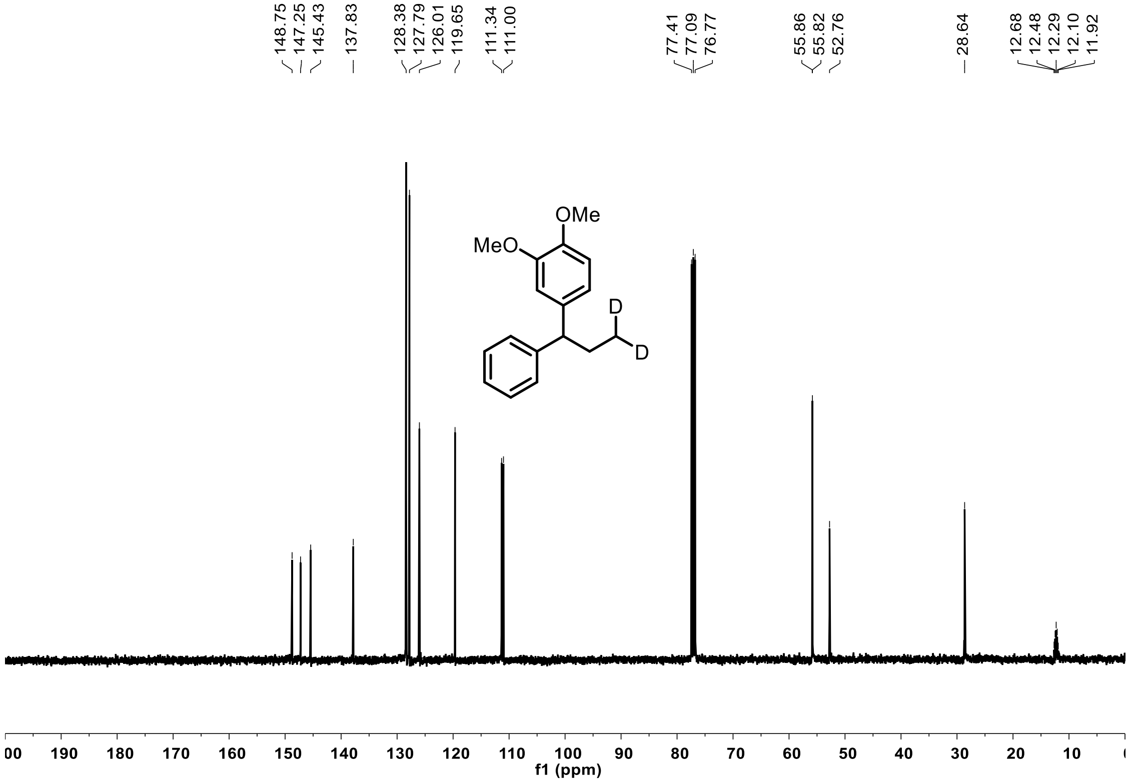

Supplementary Figure 234. ^{13}C NMR Spectrum of 3b-D_2’

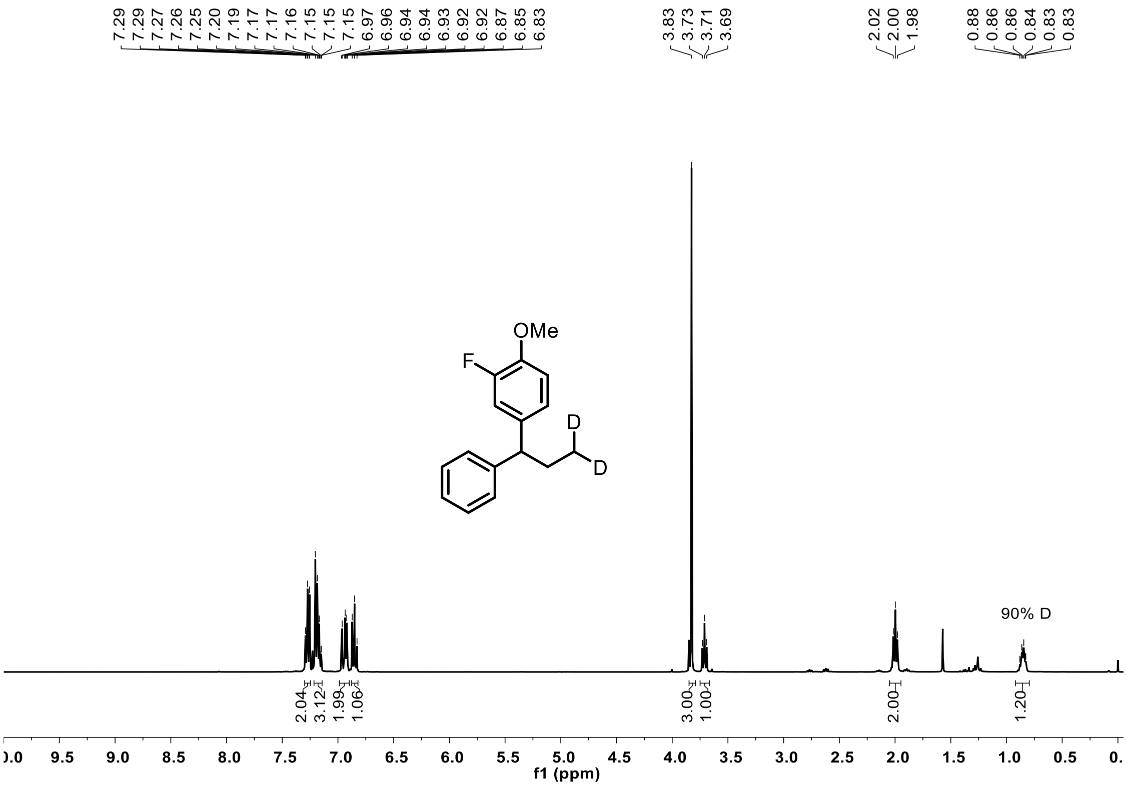

Supplementary Figure 235. ^{1}H NMR Spectrum of 3a-D_2’

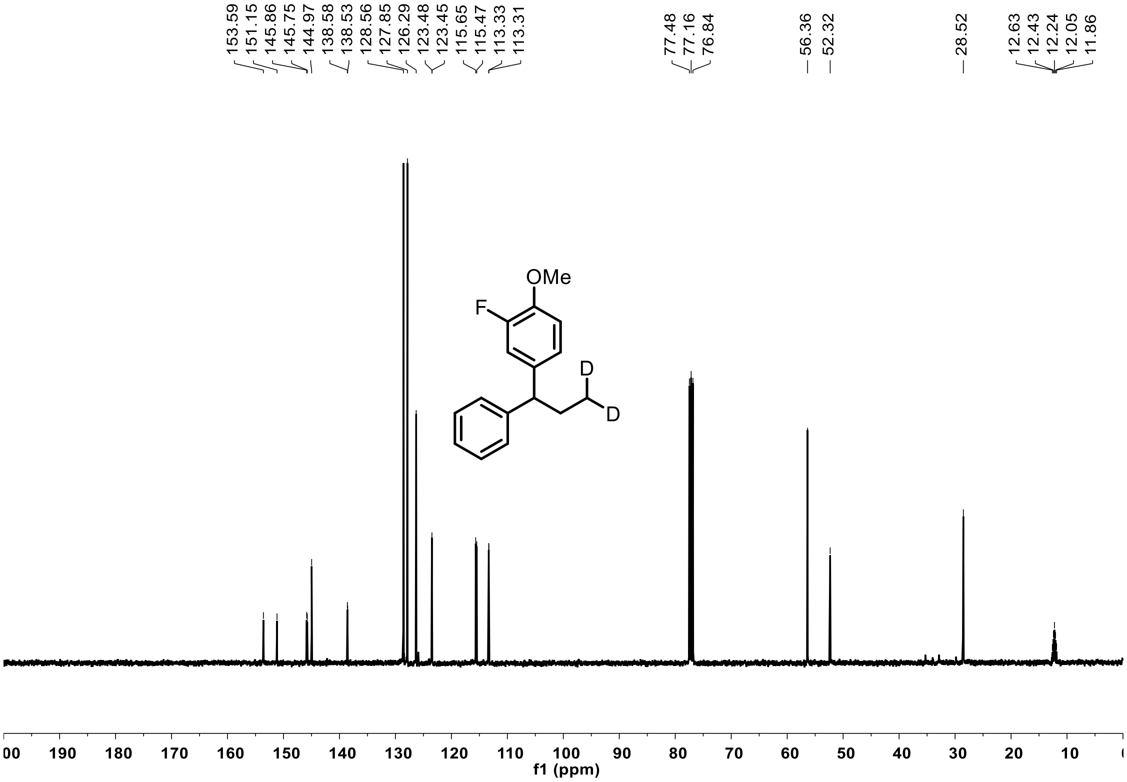

Supplementary Figure 236. ^{13}C NMR Spectrum of 3a-D_2’

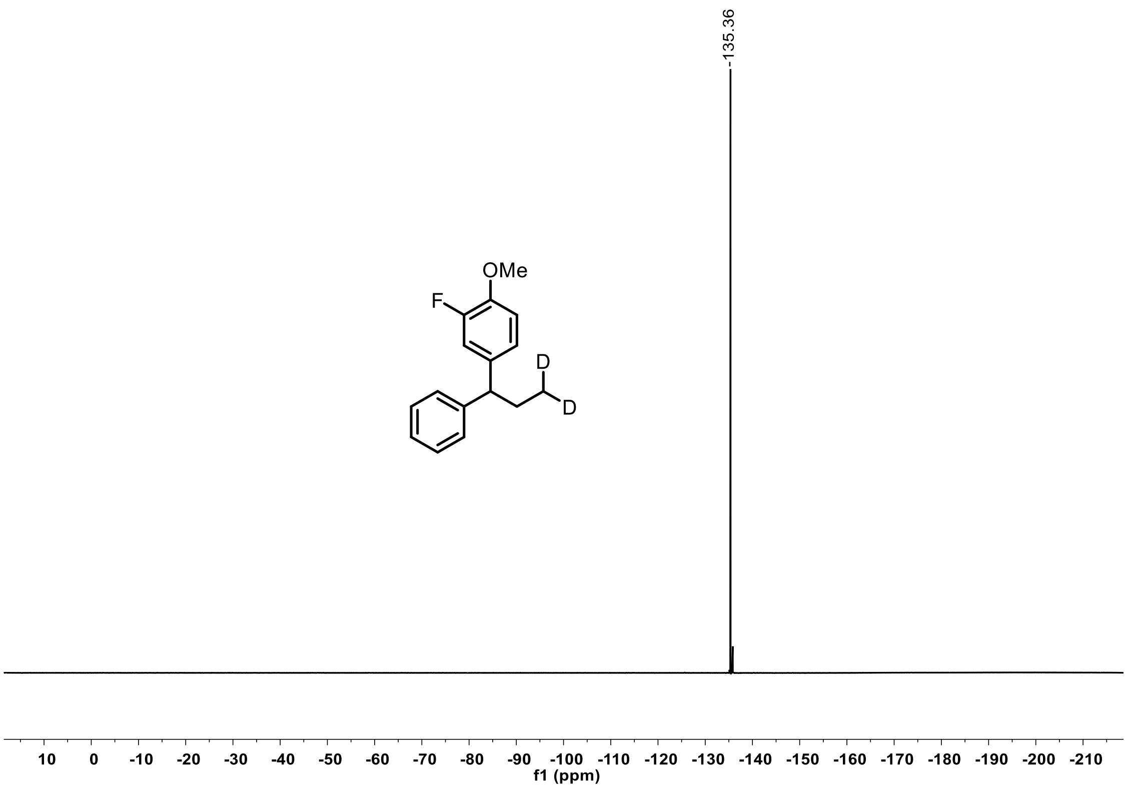

Supplementary Figure 237. ^{19}F NMR Spectrum of 3a-D_2'

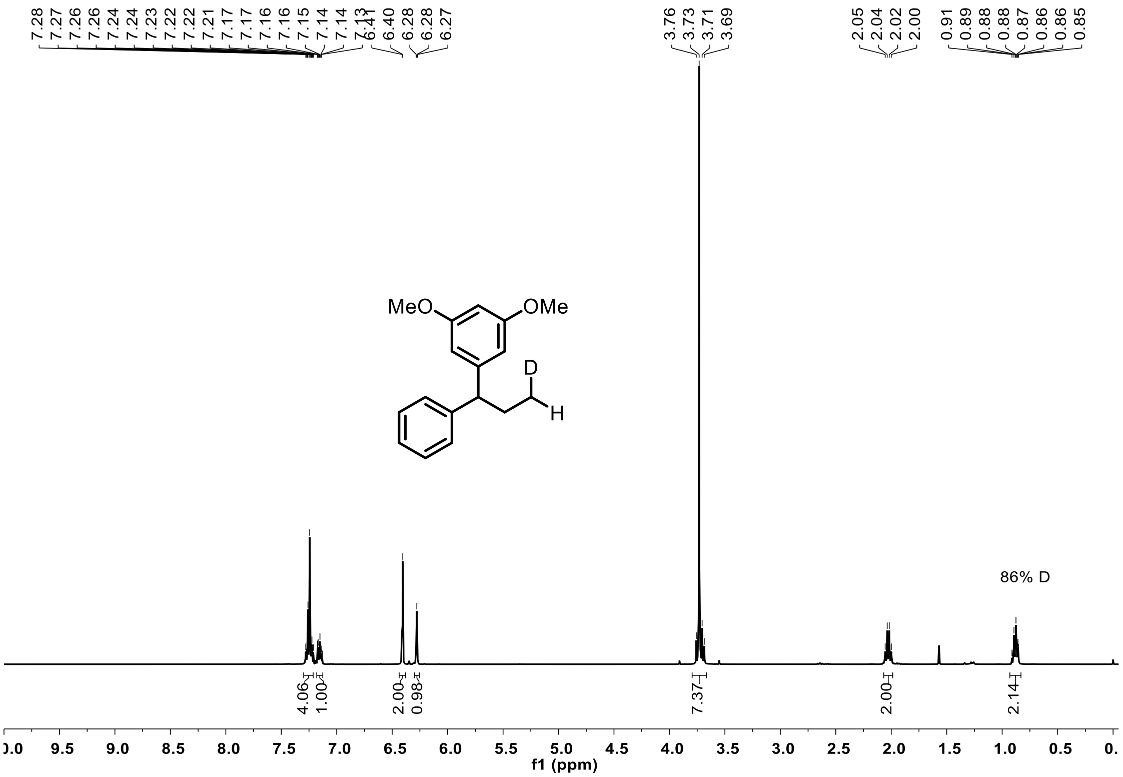

Supplementary Figure 238. ^{1}H NMR Spectrum of 3d-D_1'

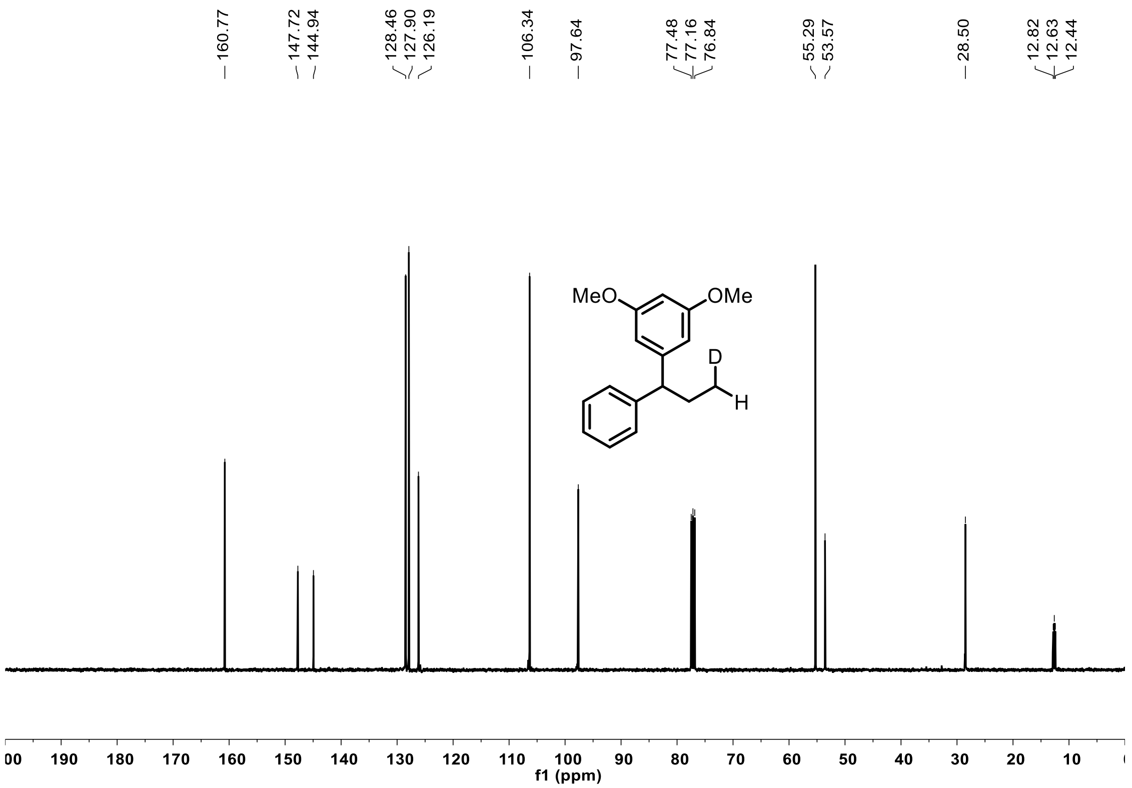

Supplementary Figure 239. ^{13}C NMR Spectrum of 3d-D$_1$'

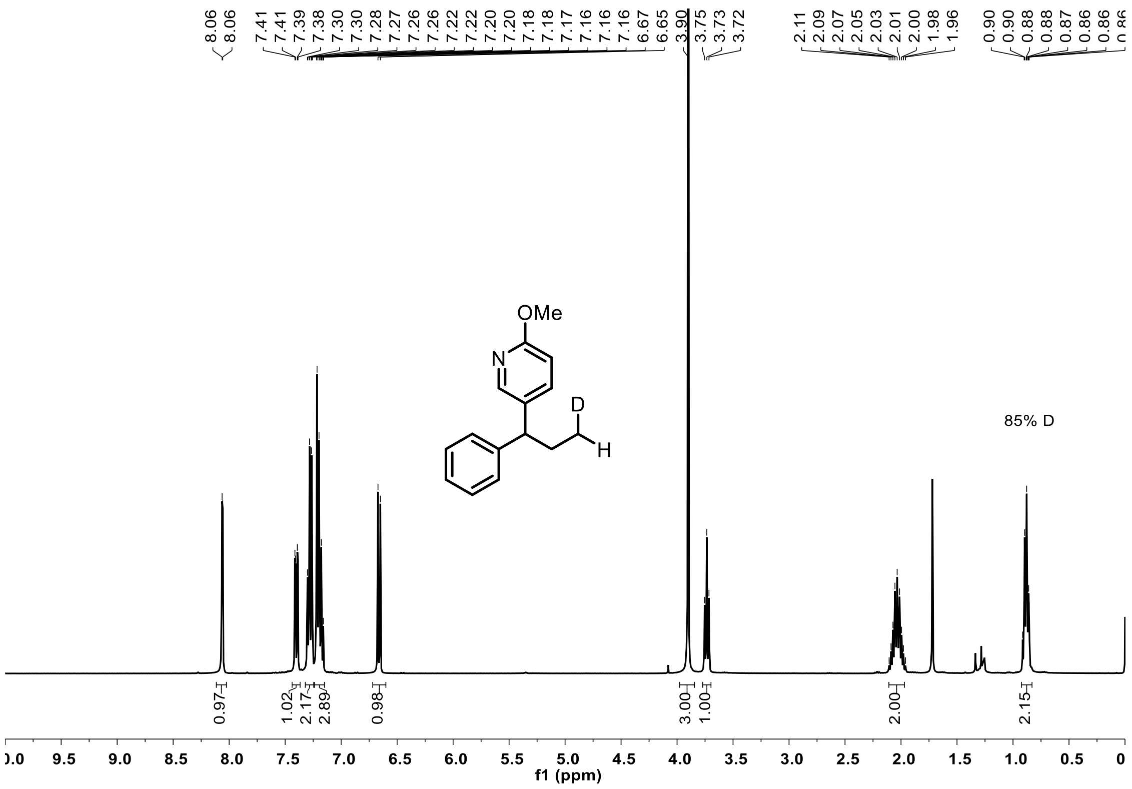

Supplementary Figure 240. ^{1}H NMR Spectrum of 3c-D$_1$'

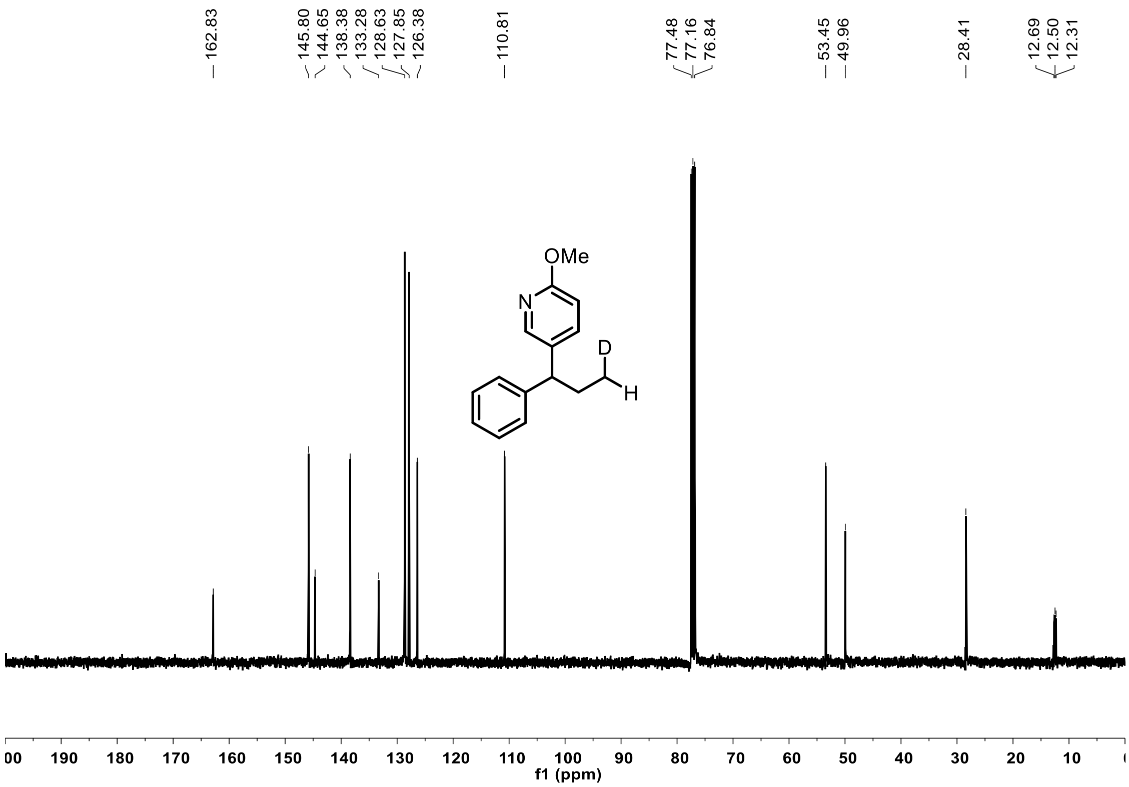

Supplementary Figure 241. ^{13}C NMR Spectrum of 3c-D$_1$’

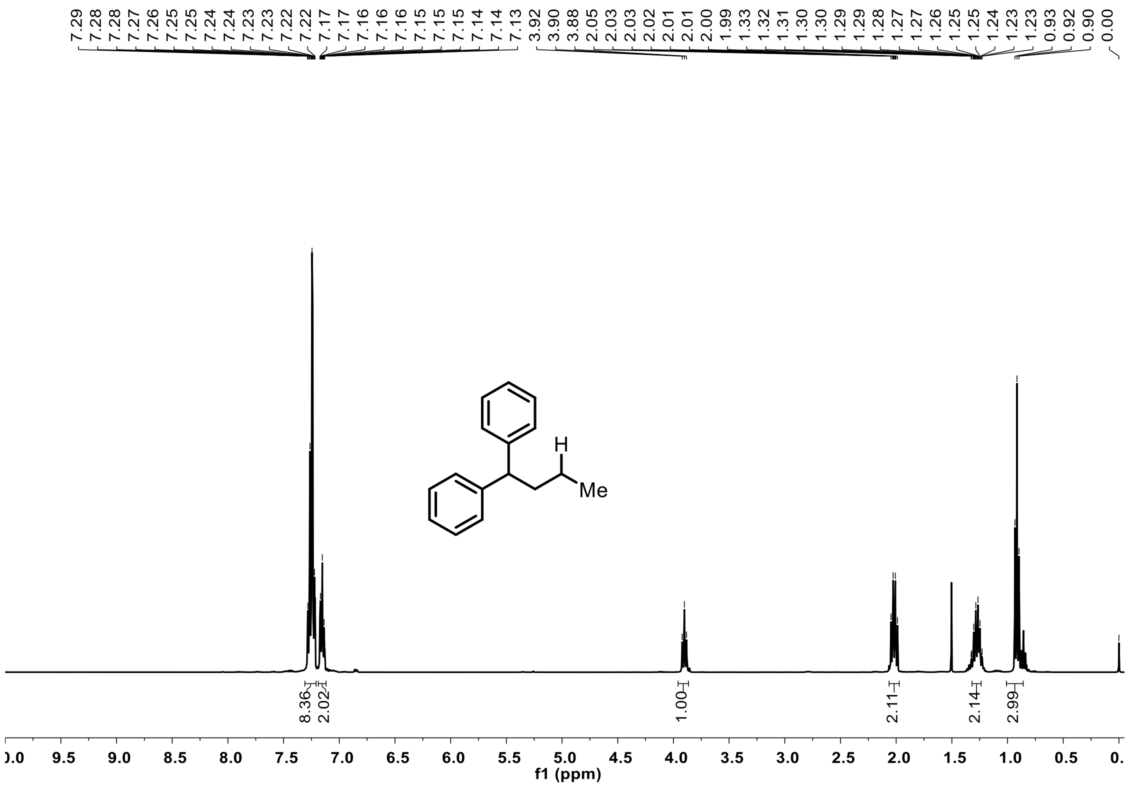

Supplementary Figure 242. ^{1}H NMR Spectrum of 3be

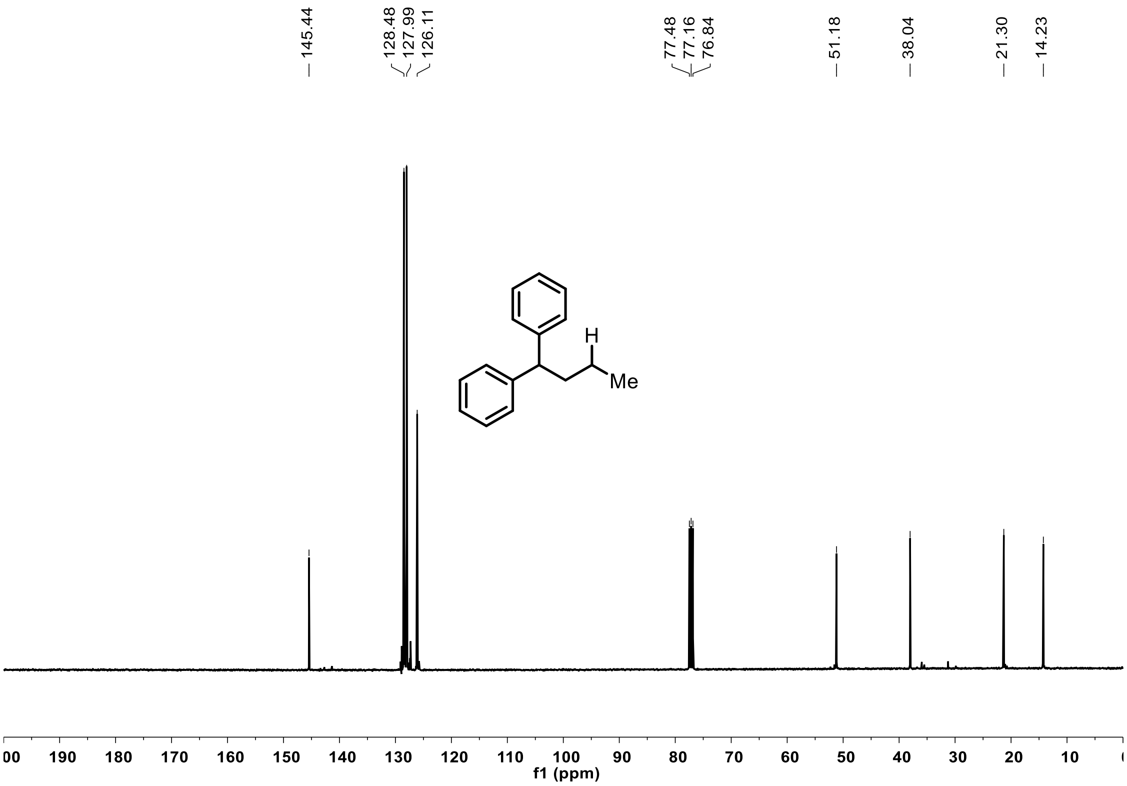

Supplementary Figure 243. ^{13}C NMR Spectrum of 3be

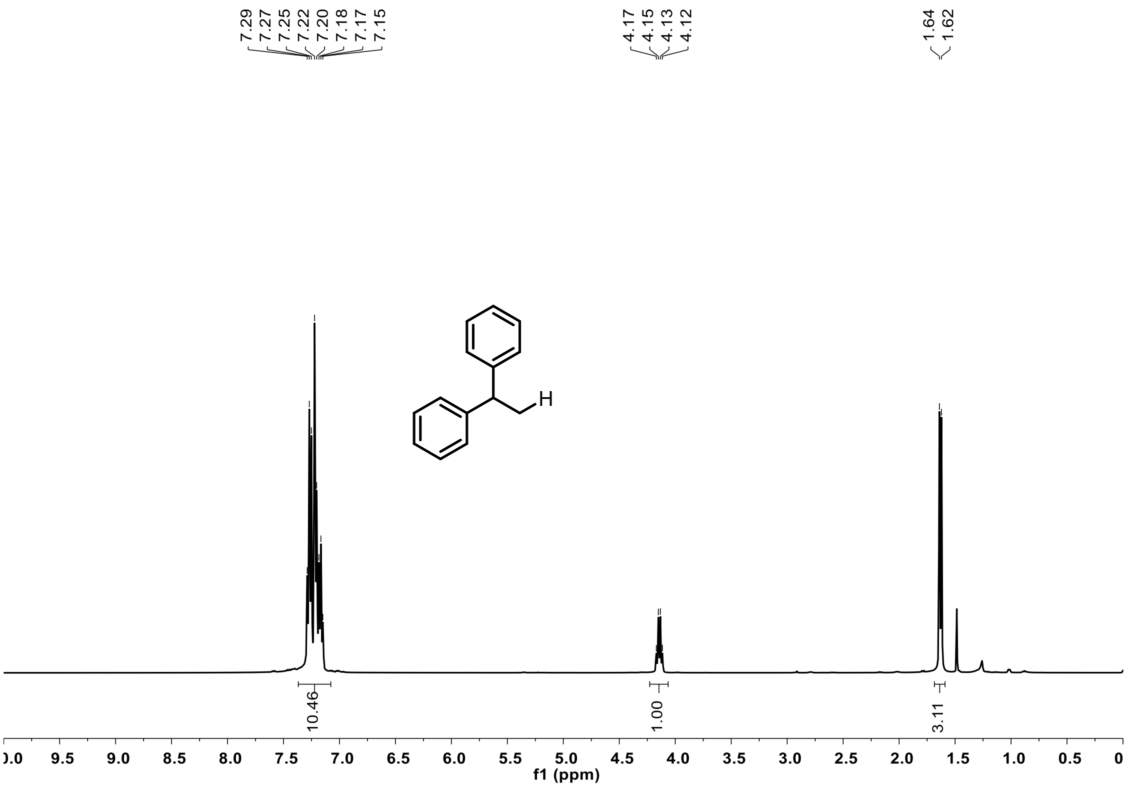

Supplementary Figure 244. ^{1}H NMR Spectrum of 3bf

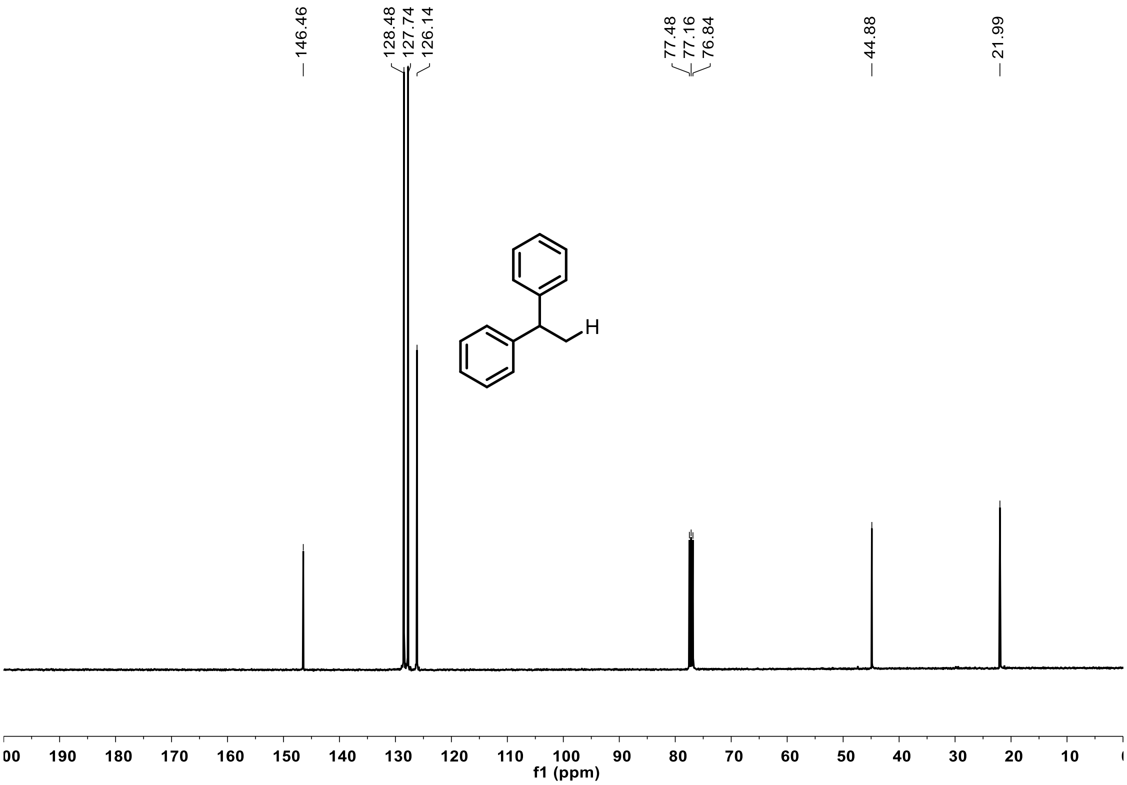

Supplementary Figure 245. ^{13}C NMR Spectrum of 3bf

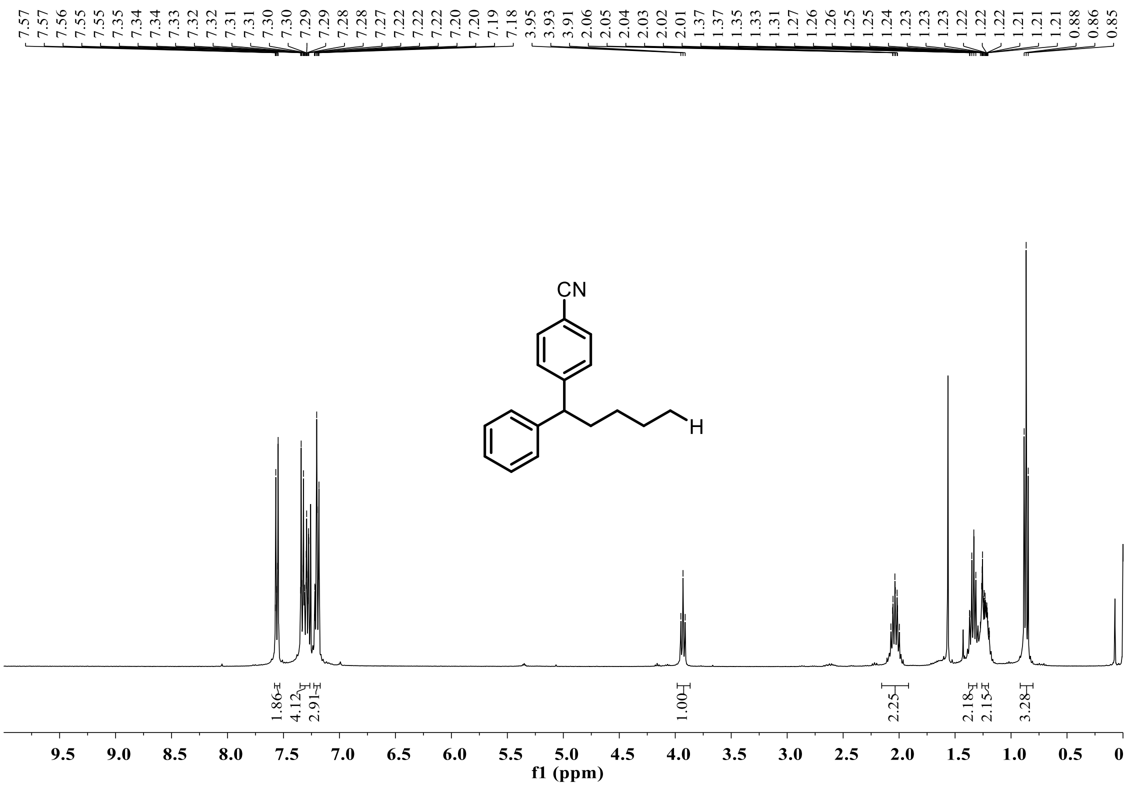

Supplementary Figure 246. ^{1}H NMR Spectrum of 3bg

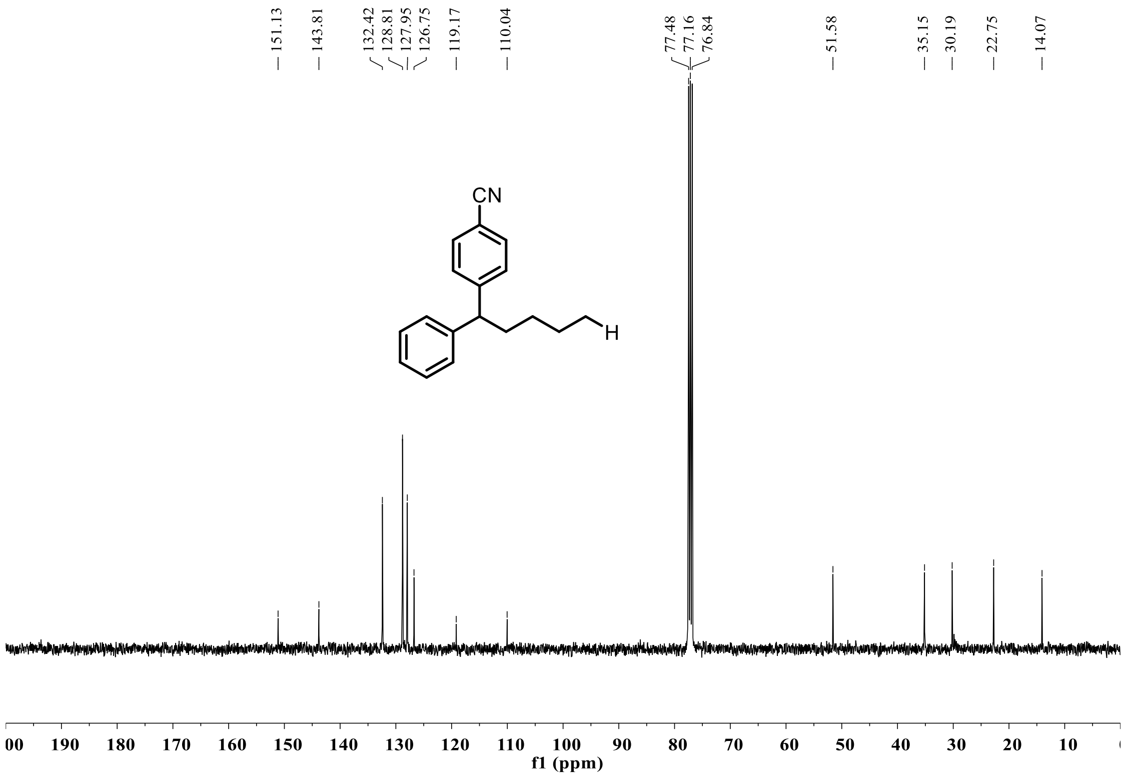

Supplementary Figure 247. ^{13}C NMR Spectrum of 3bg

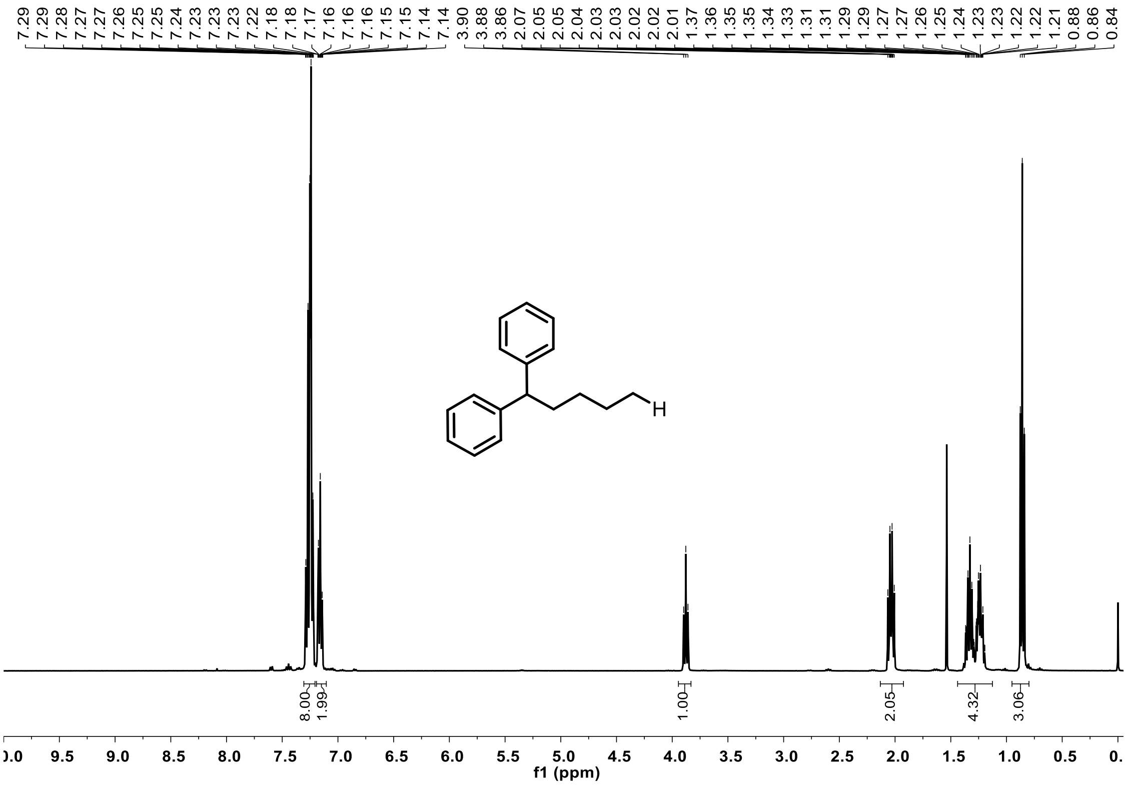

Supplementary Figure 248. ^{1}H NMR Spectrum of 3bh

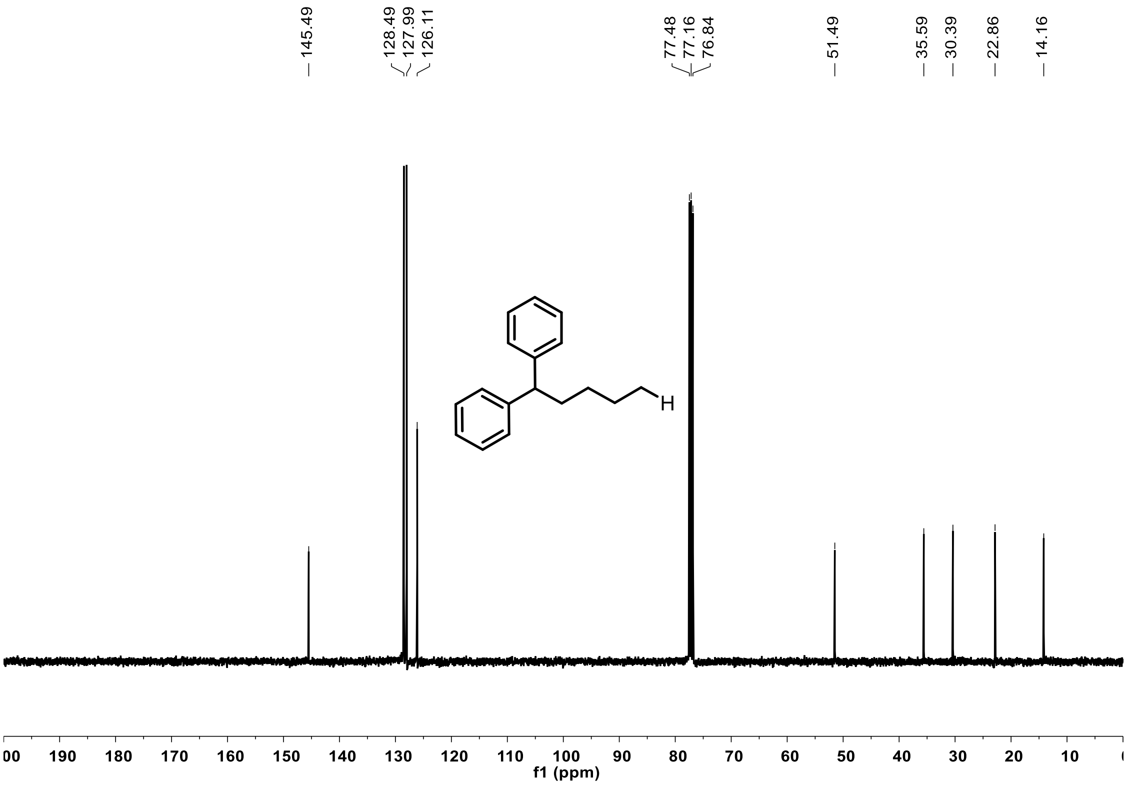

Supplementary Figure 249. ^{13}C NMR Spectrum of 3bh

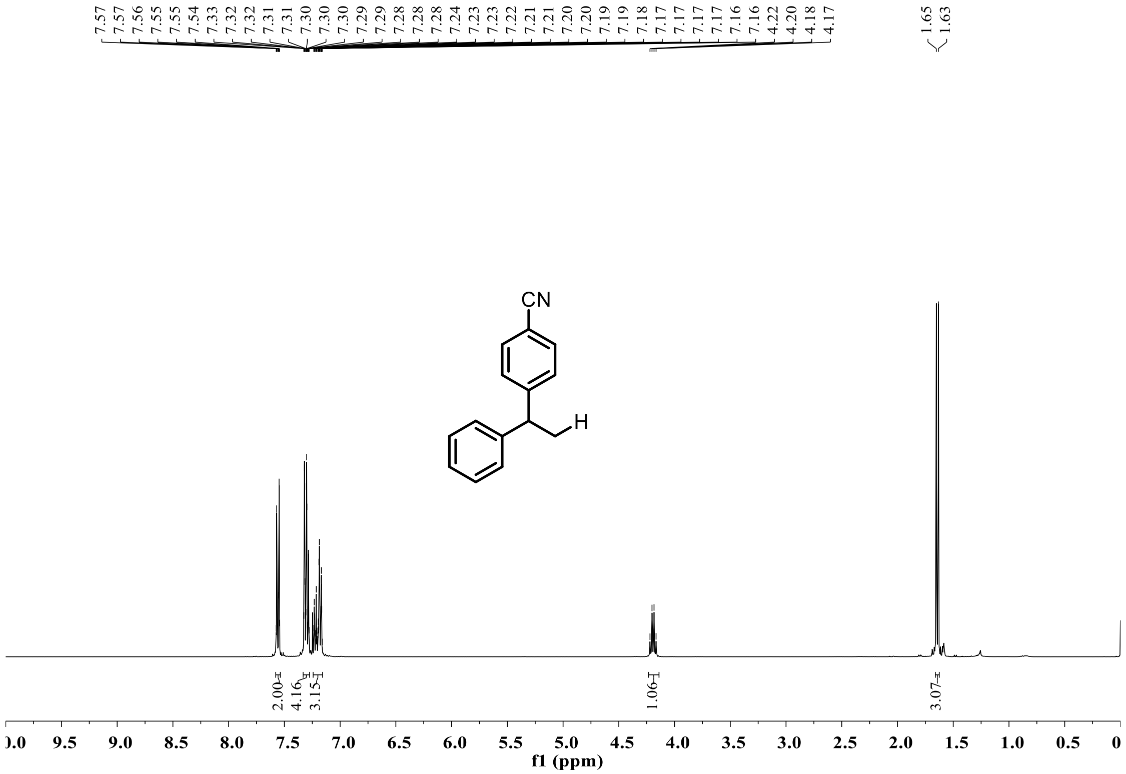

Supplementary Figure 250. ^{1}H NMR Spectrum of 3bl

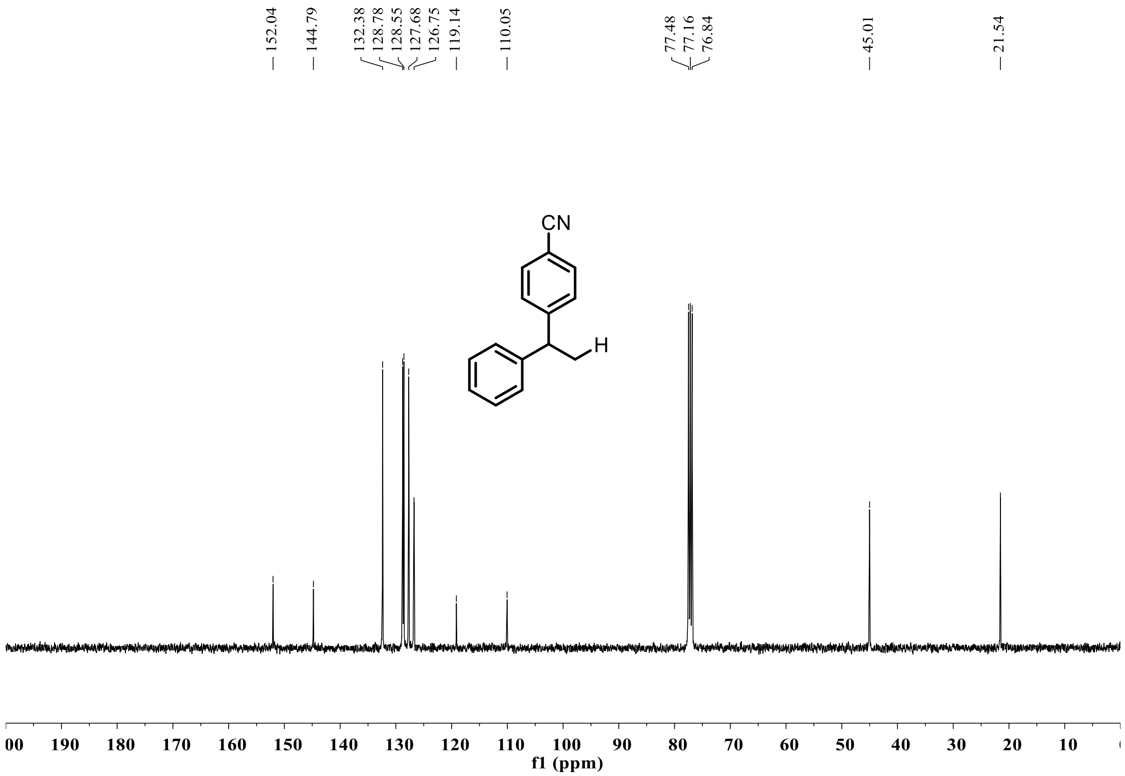

Supplementary Figure 251. ^{13}C NMR Spectrum of 3bl

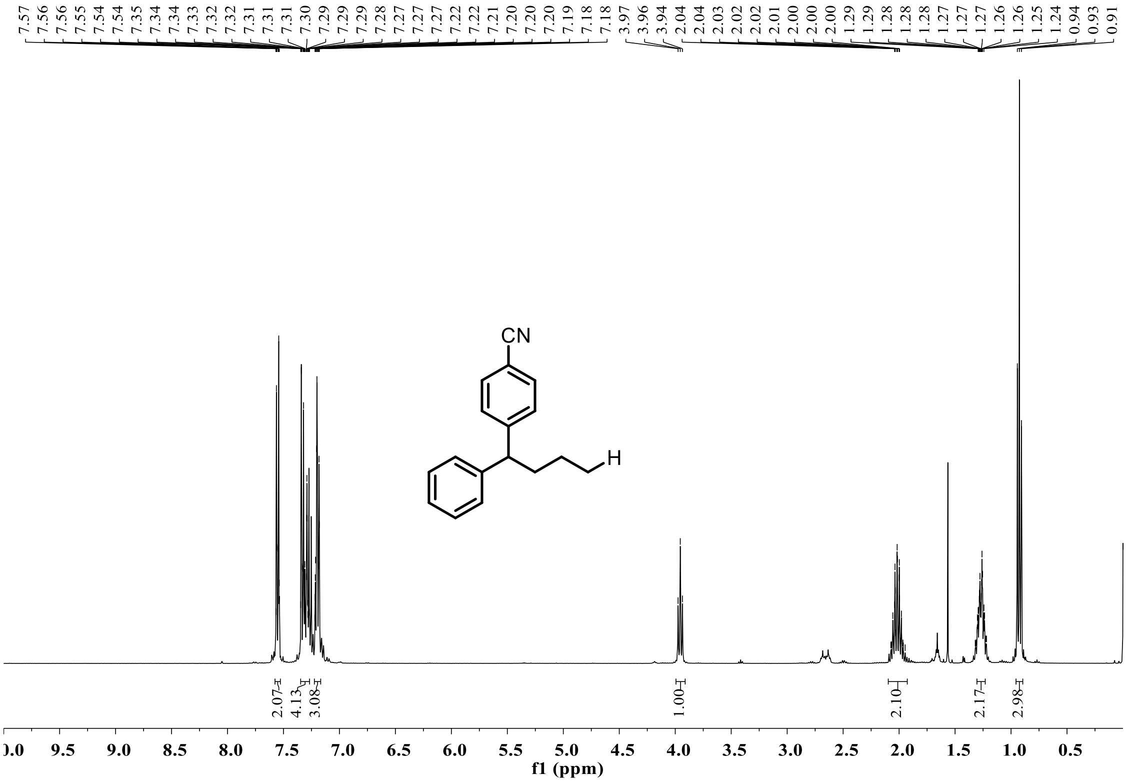

Supplementary Figure 252. ^{1}H NMR Spectrum of 3bm

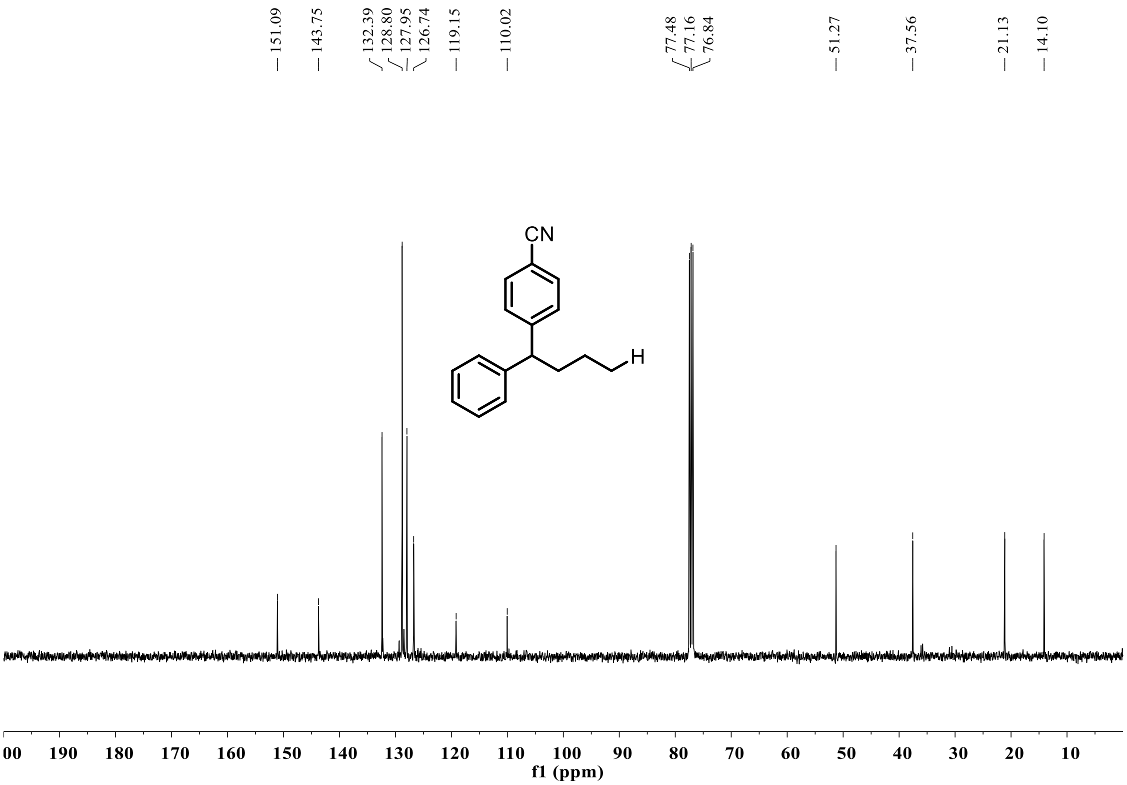

Supplementary Figure 253. ^{13}C NMR Spectrum of 3bm

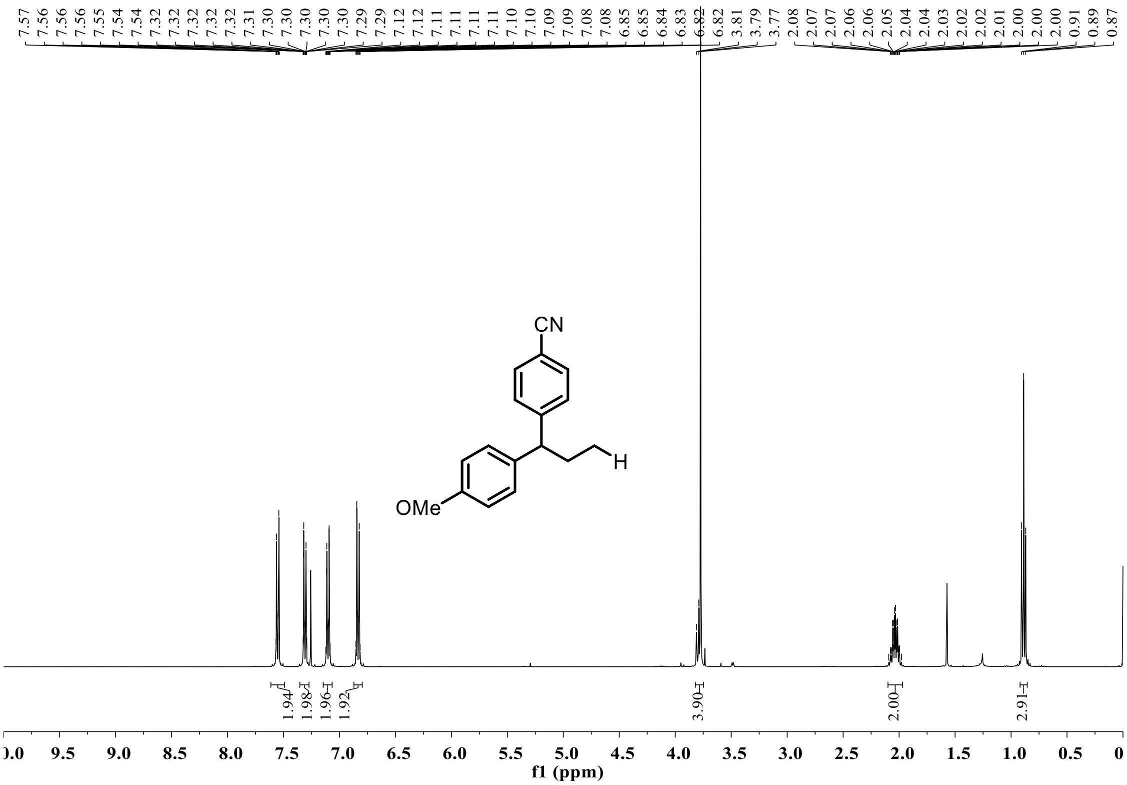

Supplementary Figure 254. ^{1}H NMR Spectrum of 3bn

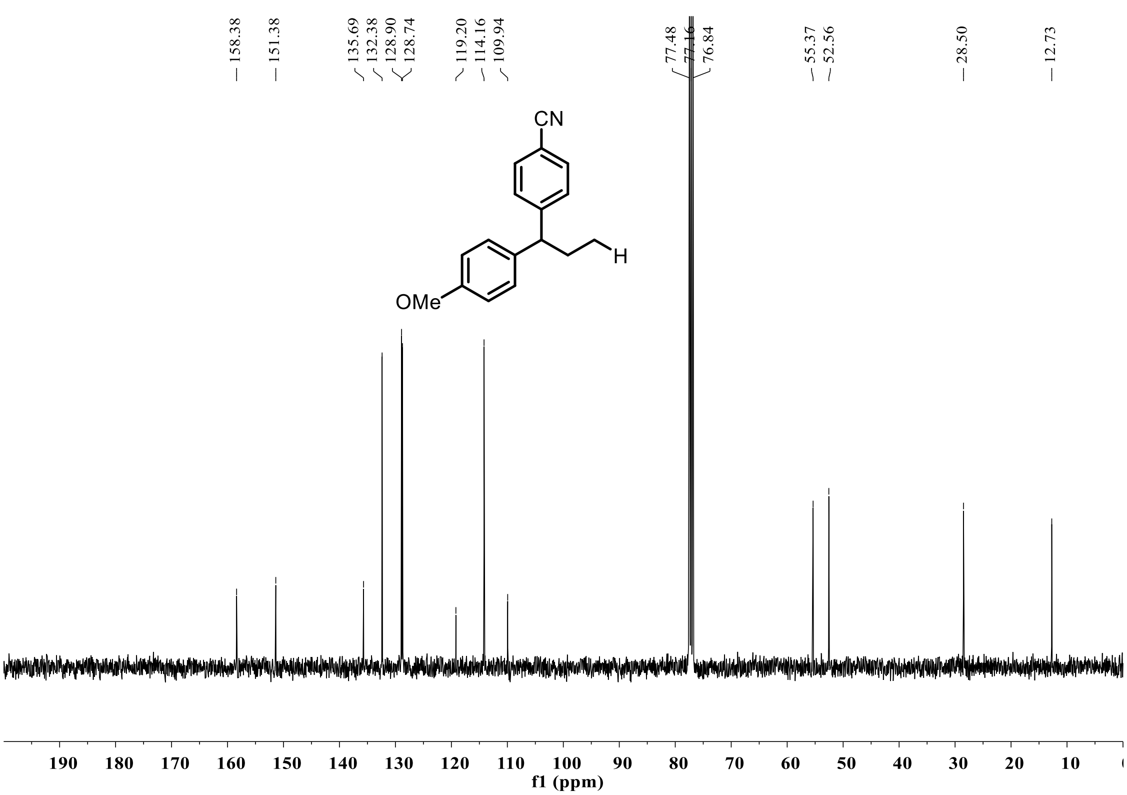

Supplementary Figure 255. ^{13}C NMR Spectrum of 3bn

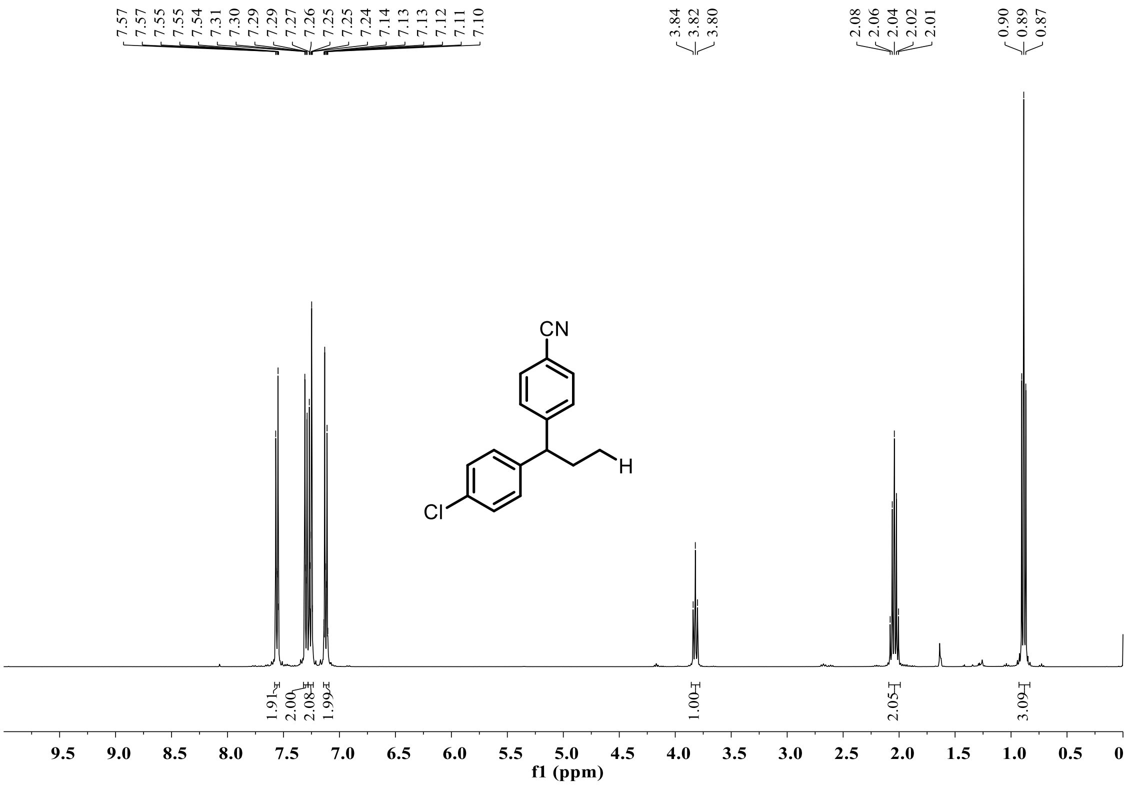

Supplementary Figure 256. ^{1}H NMR Spectrum of 3bo

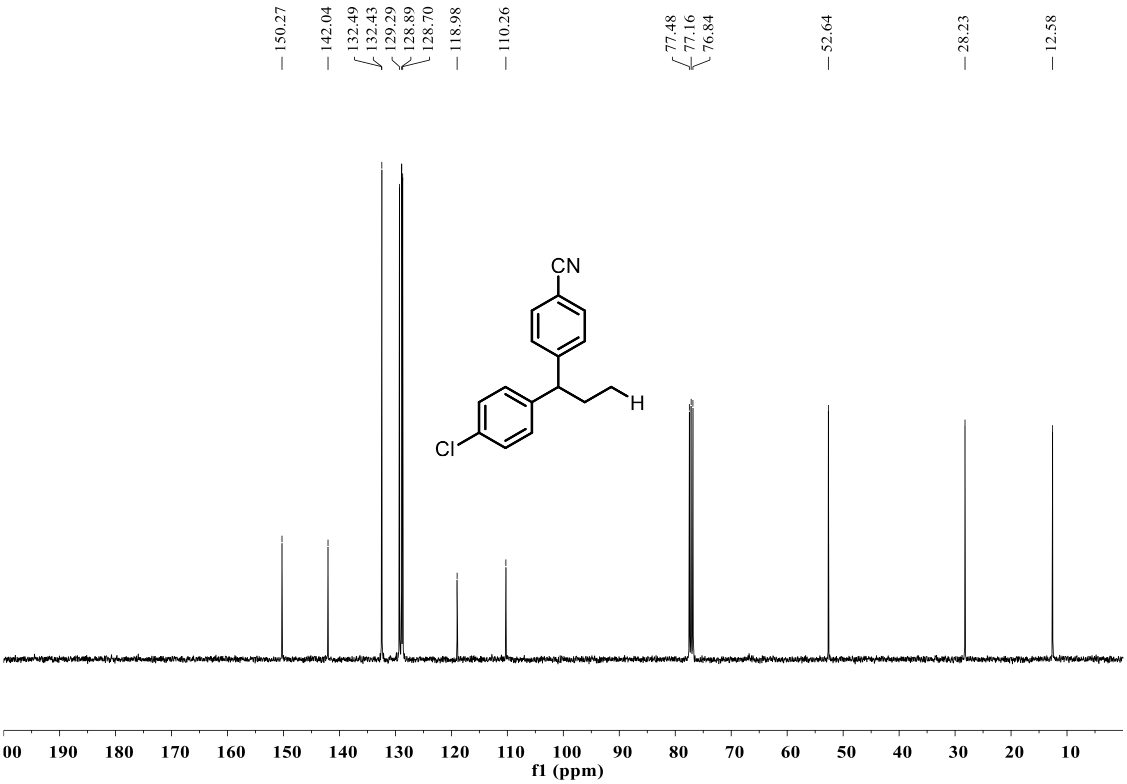

Supplementary Figure 257. ^{13}C NMR Spectrum of 3bo

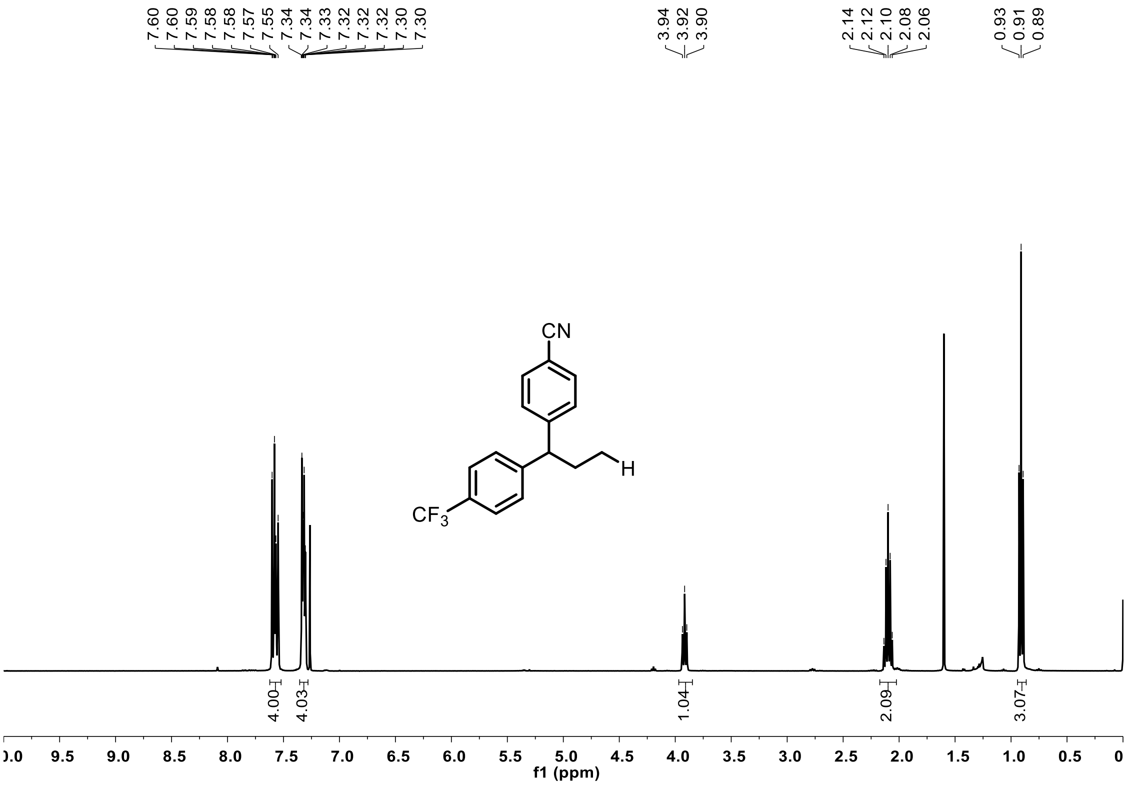

Supplementary Figure 258. ^{1}H NMR Spectrum of 3bp

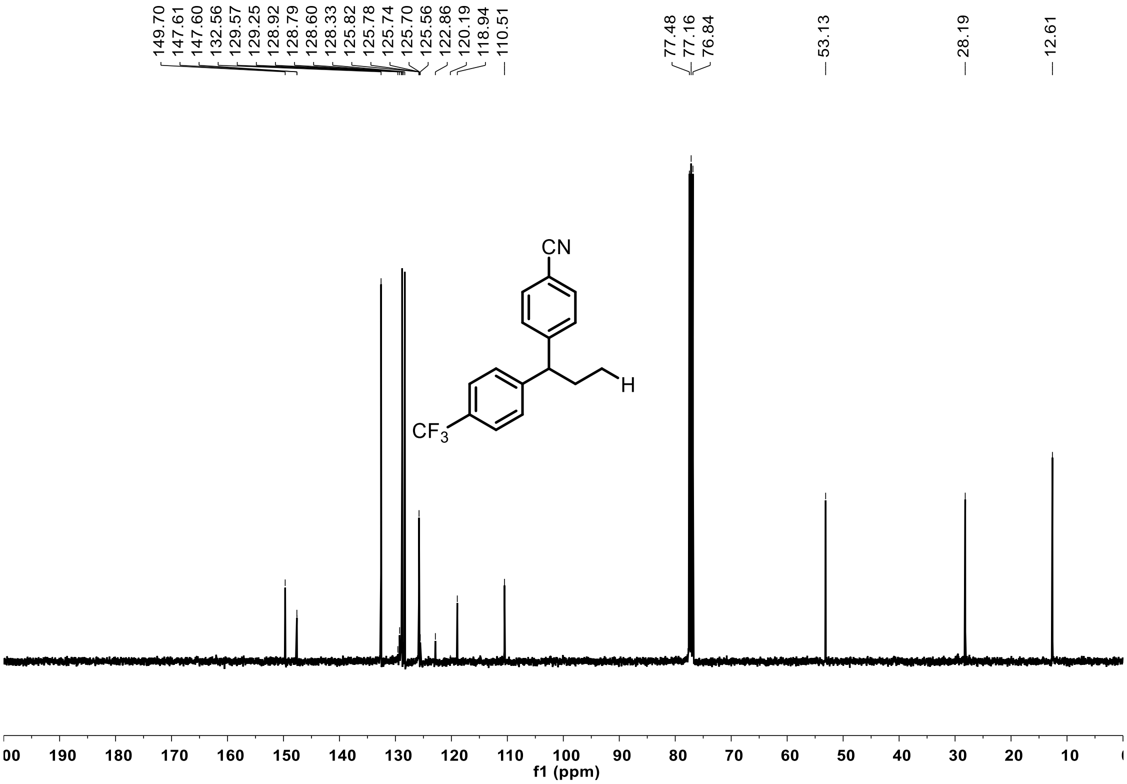

Supplementary Figure 259. ^{13}C NMR Spectrum of 3bp

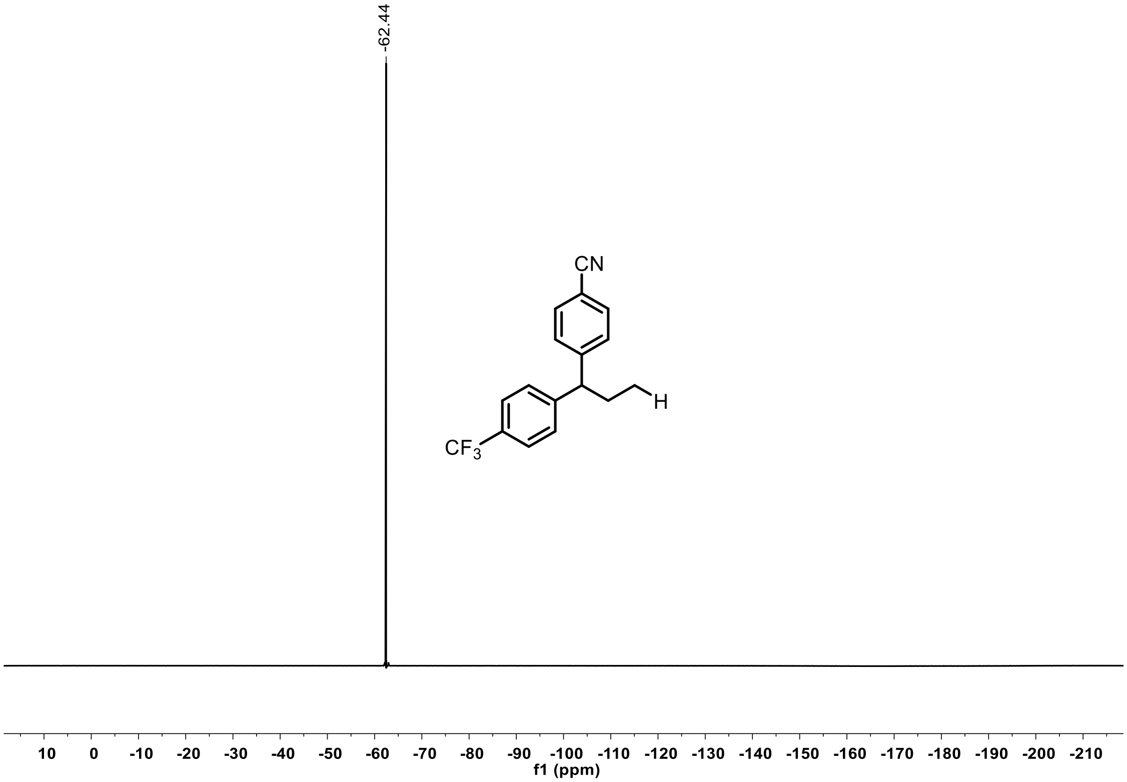

Supplementary Figure 260. ^{19}F NMR Spectrum of 3bp

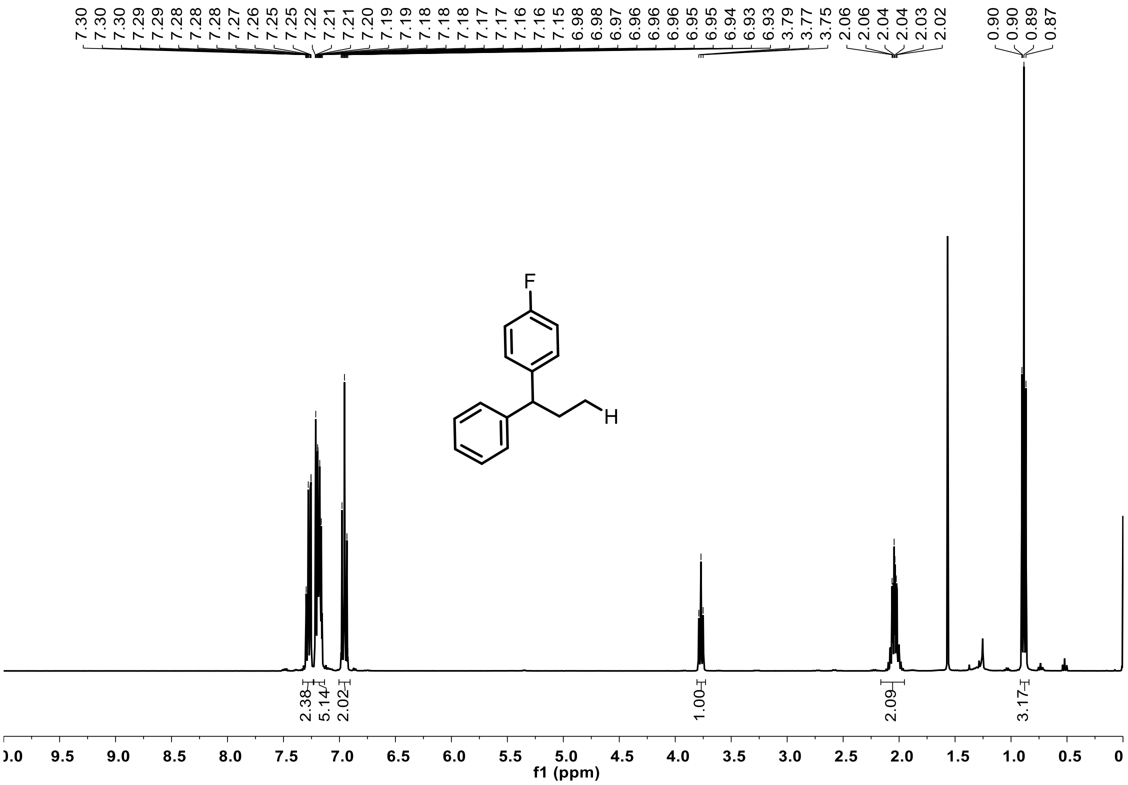

Supplementary Figure 261. ^{1}H NMR Spectrum of 3bq

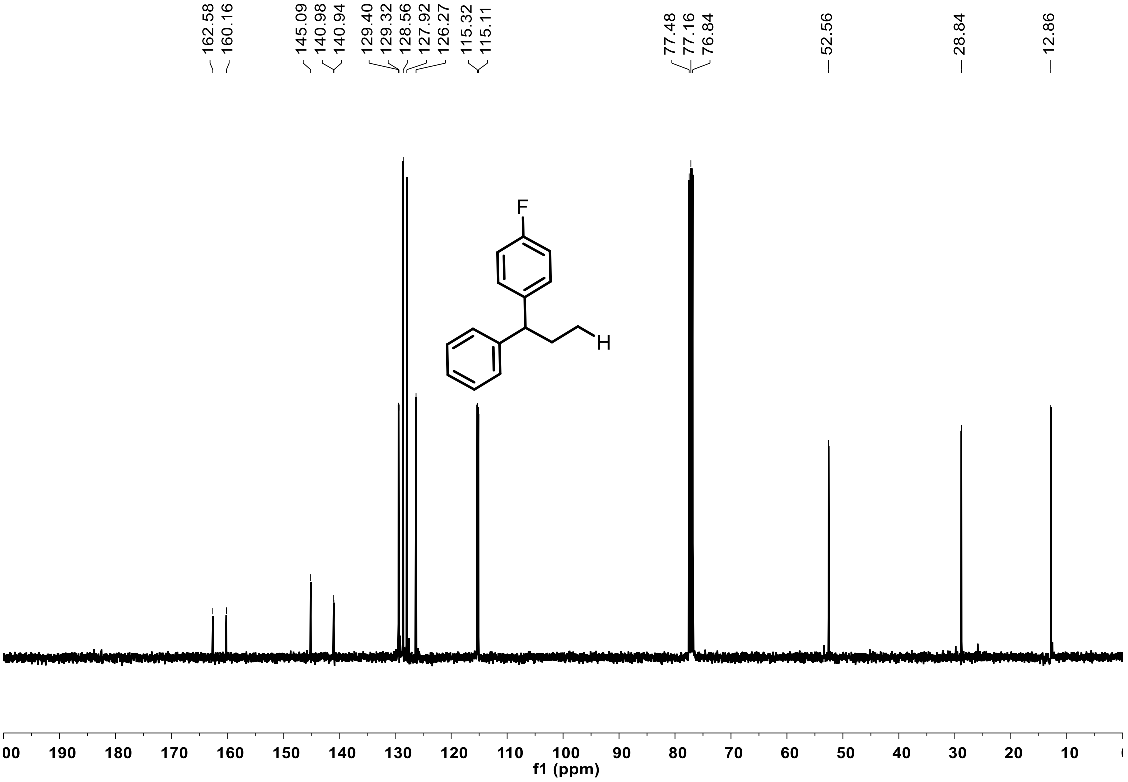

Supplementary Figure 262. ^{13}C NMR Spectrum of 3bq

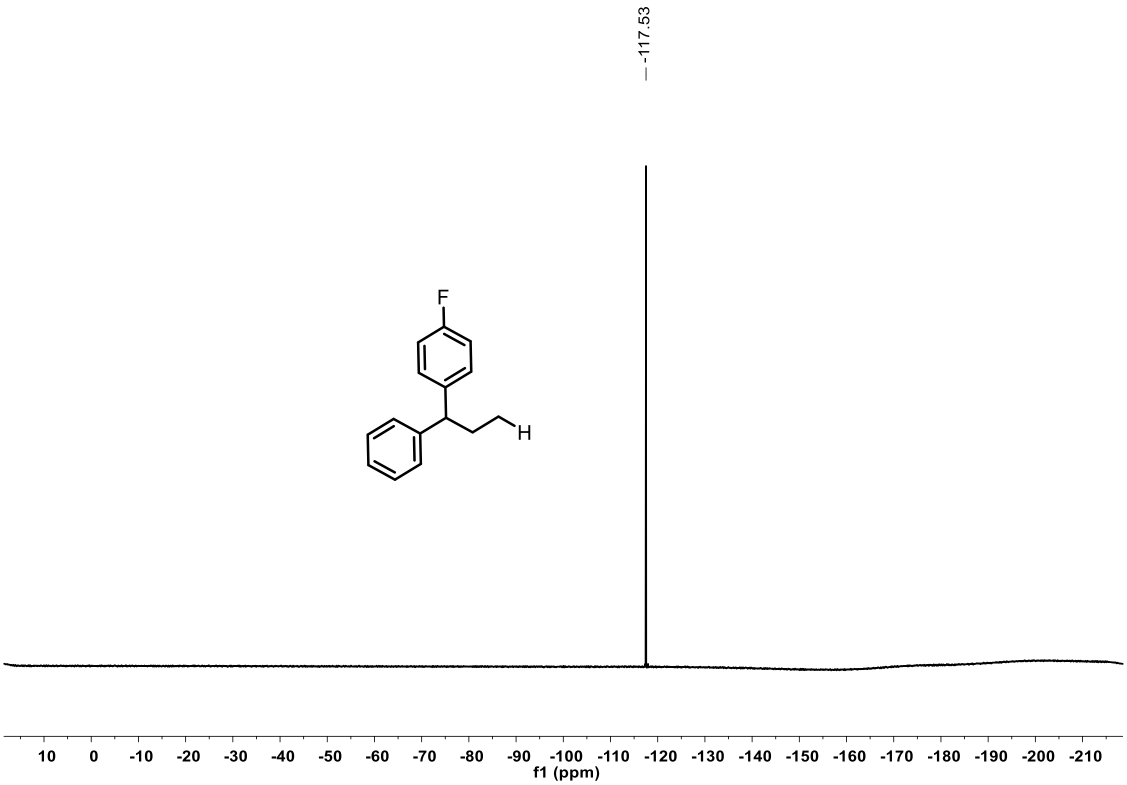

Supplementary Figure 263. ^{19}F NMR Spectrum of 3bq

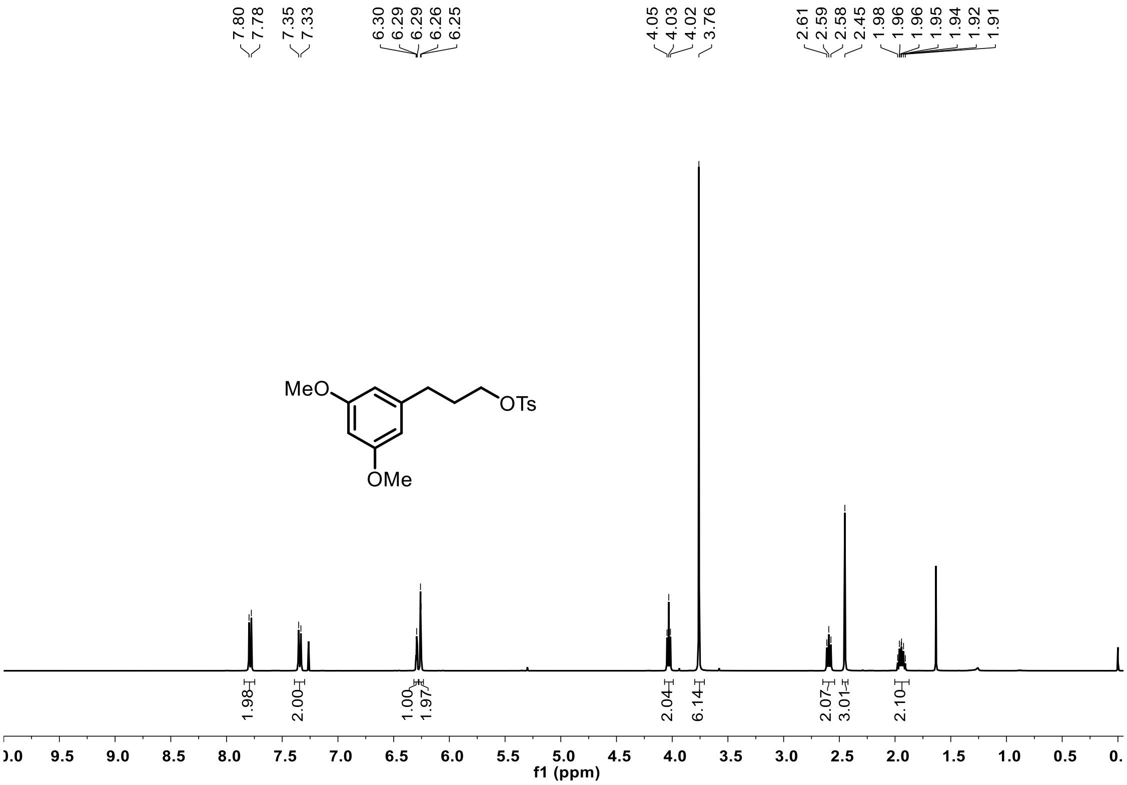

Supplementary Figure 264. ^{1}H NMR Spectrum of 1j

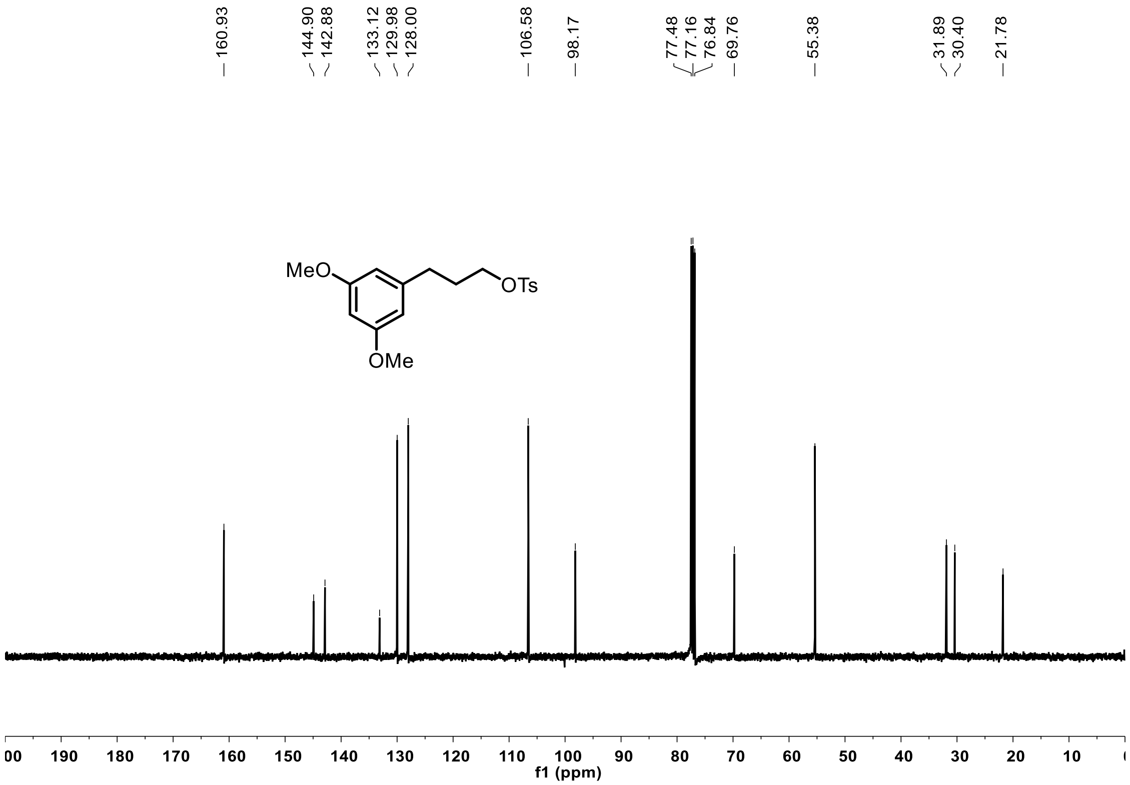

Supplementary Figure 265. ^{13}C NMR Spectrum of 1j

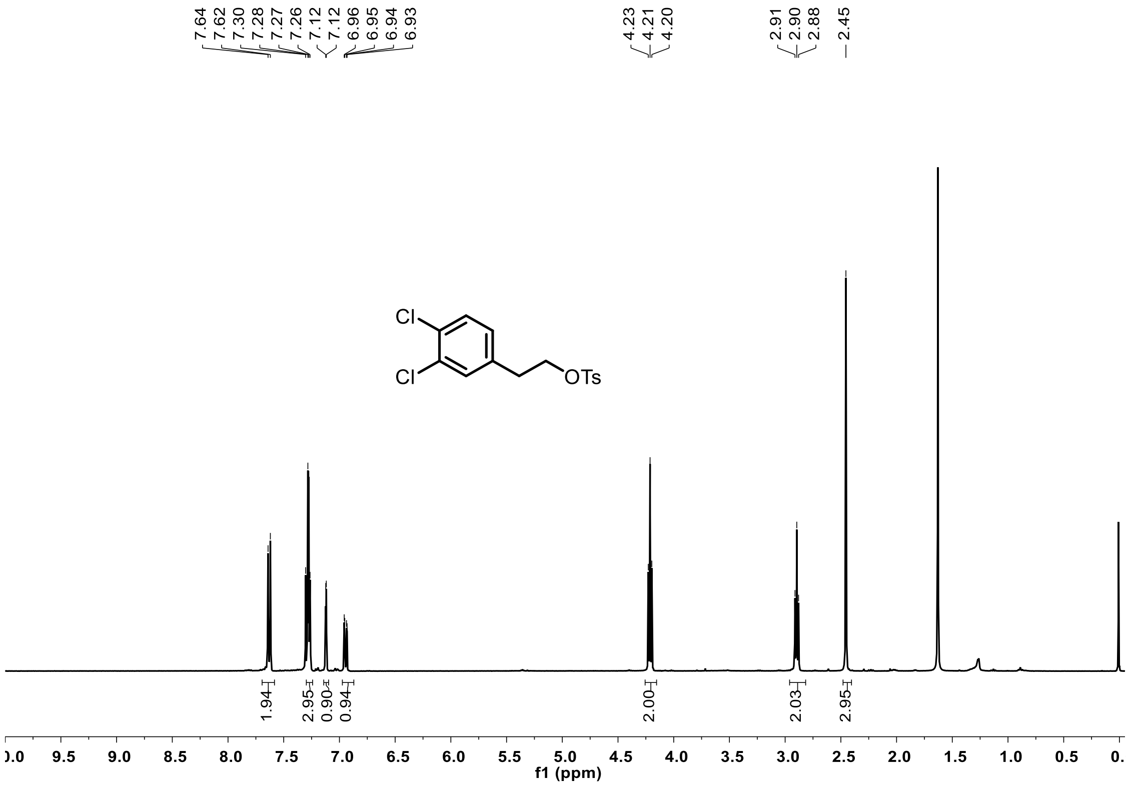

Supplementary Figure 266. ^{1}H NMR Spectrum of 1k

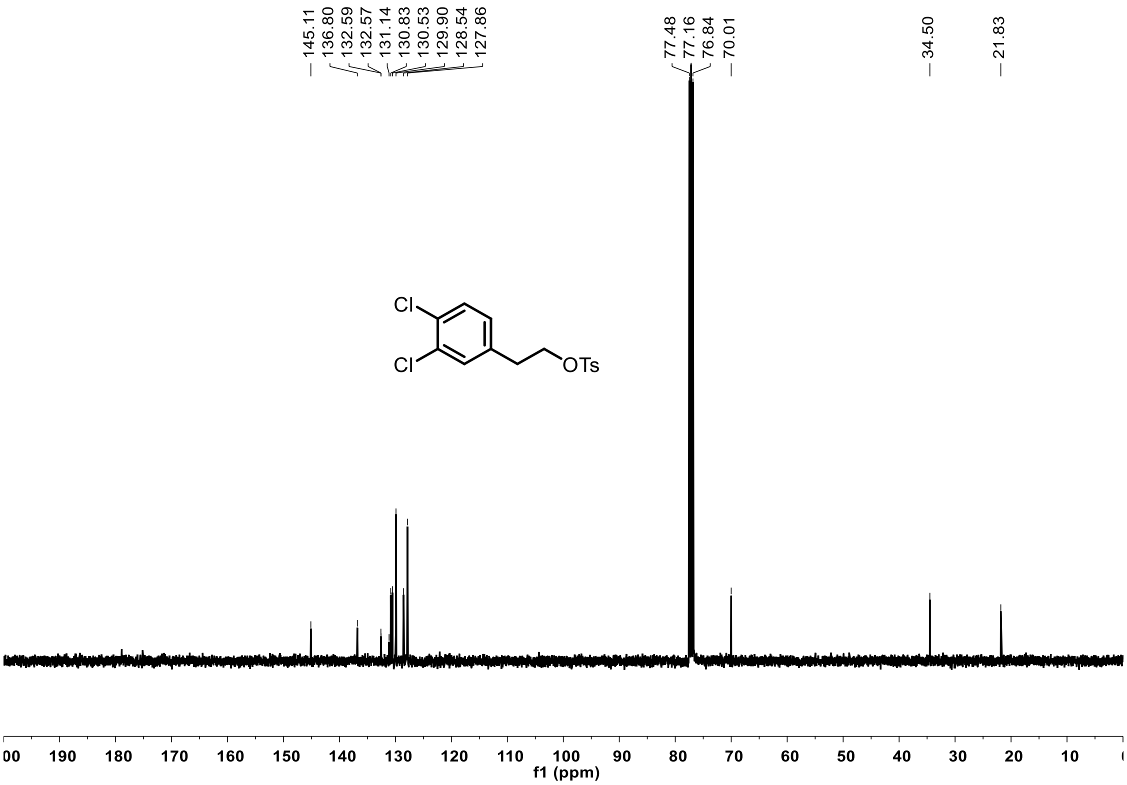

Supplementary Figure 267. ^{13}C NMR Spectrum of 1k

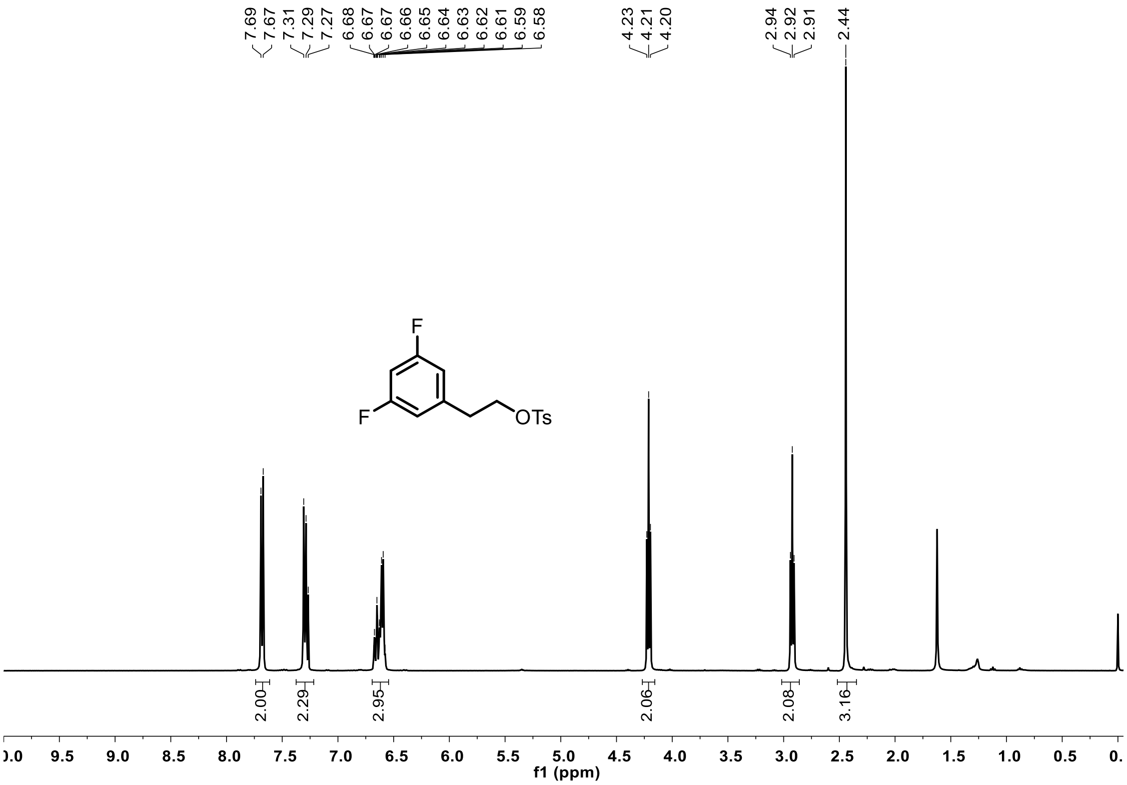

Supplementary Figure 268. ^{1}H NMR Spectrum of 1l

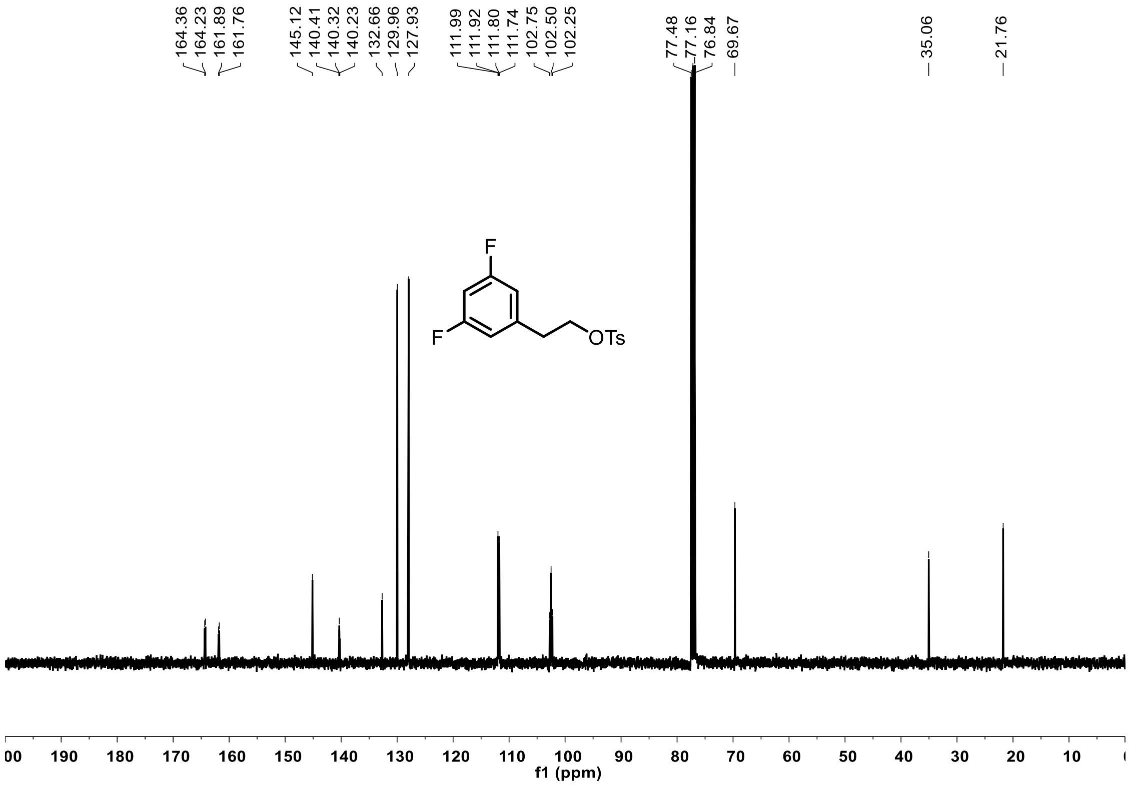

Supplementary Figure 269. ^{13}C NMR Spectrum of 1l

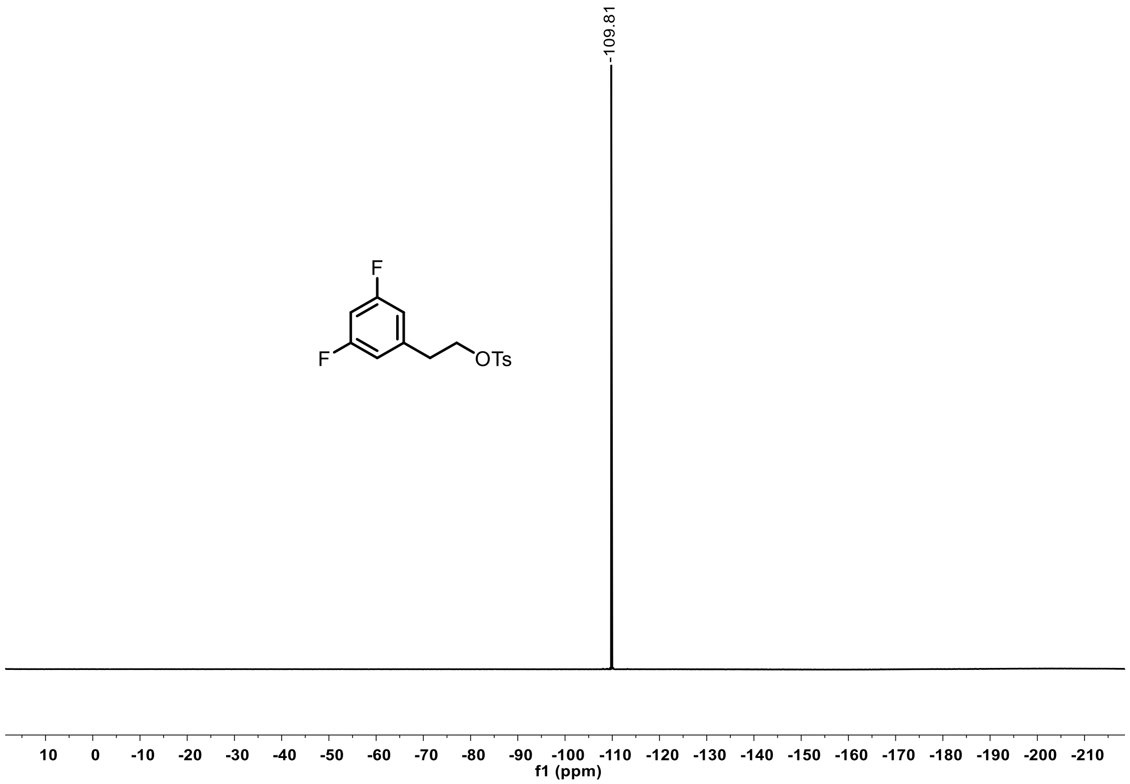

Supplementary Figure 270. ^{19}F NMR Spectrum of 1l

Supplementary References

1. Bejot, R., Tisserand, S., Li, D. R., Falck, J. R. & Mioskowski, C. A mechanistic study of the chromium(ii)-mediated transformations of trichloromethyl alkyls and carbinols: Evidence for carbene, carbyne, and carbenoid intermediates. *Tetrahedron Lett.* **48**, 3855-3858 (2007).
2. Stokes, B. J., Bischoff, A. J. & Sigman, M. S. Pd(quinox)-catalyzed allylic relay suzuki reactions of secondary homostyrenyl tosylates via alkene-assisted oxidative addition. *Chem. Sci.* **5**, 2336-2339 (2014).
3. Liu, J. H., *et al.* Copper-catalyzed reductive cross-coupling of nonactivated alkyl tosylates and mesylates with alkyl and aryl bromides. *Chem. Eur. J.* **20**, 15334-15338 (2014).
4. Kurita, T., *et al.* Efficient and convenient heterogeneous palladium-catalyzed regioselective deuteration at the benzylic position. *Chem. Eur. J.* **14**, 664-673 (2008).
5. Tzeng, Y. L., Yang, P. F., Mei, N. W., Yuan, T. M., Yu, C. C. & Luh, T. Y. Transition metal promoted reactions. 38. Nicl2(dppe)-catalyzed geminal dialkylation of dithioacetals and trimethylation of ortho thioesters. *J. Org. Chem.* **56**, 5289-5293 (1991).
6. Bedford, R. B., *et al.* Expedient iron-catalyzed coupling of alkyl, benzyl and allyl halides with arylboronic esters. *Chem. Eur. J.* **20**, 7935-7938 (2014).
7. Yan, M. Q., *et al.* Pd-indenyl-diphosphine: An effective catalyst for the preparation of triarylamines. *Org. Biomol. Chem.* **14**, 451-454 (2016).
8. Shteingarts, V., Haufe, G. & Panteleeva, E. Short access to 4-alkenylbenzonitriles: Reaction of anionic reduced forms of terephthalonitrile with alkenyl bromides. *Synlett* **2007**, 1616-1618 (2007).
9. Peng, L., Li, Y., Li, Y., Wang, W., Pang, H. & Yin, G. Ligand-controlled nickel-catalyzed reductive relay cross-coupling of alkyl bromides and aryl bromides. *ACS Catal.* **8**, 310-313 (2017).
10. Stokes, B. J., Opra, S. M. & Sigman, M. S. Palladium-catalyzed allylic cross-coupling reactions of primary and secondary homoallylic electrophiles. *J. Am. Chem. Soc.* **134**, 11408-11411 (2012).
11. Liao, L. & Sigman, M. S. Palladium-catalyzed hydroarylation of 1,3-dienes with boronic esters via reductive formation of pi-allyl palladium intermediates under oxidative conditions. *J. Am. Chem. Soc.* **132**, 10209-10211 (2010).
12. Peng, L., Li, Z. & Yin, G. Photochemical nickel-catalyzed reductive migratory cross-coupling of alkyl bromides with aryl bromides. *Org. Lett.* **20**, 1880-1883 (2018).
13. Masson-Makdissi, J., Vandavasi, J. K. & Newman, S. G. Switchable selectivity in the pd-catalyzed alkylative cross-coupling of esters. *Org. Lett.* **20**, 4094-4098 (2018).
14. Gong, H., Qian, Q., Zhang, Q. & Wang, X. Nickel-catalyzed reductive cross-coupling of benzyl halides with aryl halides. *Synthesis* **48**, 2829-2836 (2016).
15. Gaussian 09, Revision D.01, Frisch, M. J.; Trucks, G. W.; Schlegel, H. B.; Scuseria, G. E.; Robb, M. A.; Cheeseman, J. R.; Scalmani, G.; Barone, V.; Mennucci, B.; Petersson, G. A.; Nakatsuji, H.; Caricato, M.; Li,

X.; Hratchian, H. P.; Izmaylov, A. F.; Bloino, J.; Zheng, G.; Sonnenberg, J. L.; Hada, M.; Ehara, M.; Toyota, K.; Fukuda, R.; Hasegawa, J.; Ishida, M.; Nakajima, T.; Honda, Y.; Kitao, O.; Nakai, H.; Vreven, T.; Montgomery, Jr., J. A.; Peralta, J. E.; Ogliaro, F.; Bearpark, M.; Heyd, J. J.; Brothers, E.; Kudin, K. N.; Staroverov, V. N.; Kobayashi, R.; Normand, J.; Raghavachari, K.; Rendell, A.; Burant, J. C.; Iyengar, S. S.; Tomasi, J.; Cossi, M.; Rega, N.; Millam, N. J.; Klene, M.; Knox, J. E.; Cross, J. B.; Bakken, V.; Adamo, C.; Jaramillo, J.; Gomperts, R.; Stratmann, R. E.; Yazyev, O.; Austin, A. J.; Cammi, R.; Pomelli, C.; Ochterski, J. W.; Martin, R. L.; Morokuma, K.; Zakrzewski, V. G.; Voth, G. A.; Salvador, P.; Dannenberg, J. J.; Dapprich, S.; Daniels, A. D.; Farkas, Ö.; Foresman, J. B.; Ortiz, J. V.; Cioslowski, J.; Fox, D. J. Gaussian, Inc., Wallingford CT, **2009**.

16. Lee, C.; Yang, W. & Parr, R. G. Development of the Colle-Salvetti correlation-energy formula into a functional of the electron density. *Phys. Rev. B* ***37***, 785-789 (1998).
17. Becke, A. D. Density-functional thermochemistry. III. The role of exact exchange. *J. Chem. Phys.* ***98***, 5648-5652 (1993).
18. Zhao, Y. & Truhlar, D. G. Density functionals with broad applicability in chemistry. *Acc. Chem. Res.* ***41***, 157-167 (2008).
19. Zhao, Y. & Truhlar, D. G. The M06 suite of density functionals for main group thermochemistry, thermochemical kinetics, noncovalent interactions, excited states, and transition elements: two new functionals and systematic testing of four M06-class functionals and 12 other functionals. *Theor. Chem. Acc.* ***120***, 215-241 (2007).
20. Marenich, A. V.; Cramer, C. J. & Truhlar, D. G. Universal solvation model based on solute electron density and on a continuum model of the solvent defined by the bulk dielectric constant and atomic surface tensions. *J. Phys. Chem. B* ***113***, 6378-6396 (2009).
