# Supplementary material for: Reaction scope and mechanistic insights of nickel-catalyzed migratory Suzuki–Miyaura cross-coupling
Source: Nat Commun. 2020 Jan 21;11:417. doi: 10.1038/s41467-019-14016-1 (PMC6972863; doi:10.1038/s41467-019-14016-1)
Supplement: Supplementary file 3 — Source Data [file 41467_2019_14016_MOESM3_ESM.pdf]

Absolute Calculation Energies, Enthalpies, and Free Energies

Geometry	$E_{(elec\text{-}B3LYP)}$[1]	$E_{(solv,\ M06)}$[2]	$G_{(corr\text{-}B3LYP)}$[3]	$H_{(corr\text{-}B3LYP)}$[4]	IF[5]
**CP1**	-1569.483234	-1568.856797	0.452682	0.554803	-
**1b**	-2920.990064	-2923.422866	0.138057	0.187655	-
**DMA**	-287.824913	-287.725480	0.099007	0.139646	-
**CP2**	-4490.509279	-4492.316971	0.606354	0.744306	-
**TS1**	-4490.497275	-4492.295373	0.607924	0.742141	-263.40
**CP3**	-3853.174246	-3855.261833	0.330168	0.418166	-
**CP4**	-349.514582	-349.326063	0.135681	0.181003	-
**CP5**	-4202.704007	-4204.614283	0.495404	0.603862	-
**CP6**	-4202.647007	-4204.555600	0.489559	0.602909	-
**TS2**	-4202.625583	-4204.539165	0.490122	0.601051	-237.64
**TS3**	-4202.664914	-4204.589476	0.493642	0.598781	-259.60
**CP7**	-3853.728278	-3855.819293	0.341584	0.426924	-
**CP8**	-348.949838	-348.764192	0.128208	0.171251	-
**TS4**	-4202.662977	-4204.592031	0.491933	0.598087	-610.85
**CP9**	-4202.693680	-4204.610502	0.494461	0.602287	-
**TS5**	-4202.674780	-4204.596594	0.491626	0.598117	-375.16
**CP10**	-3853.728278	-3855.819292	0.341582	0.426925	-
**CP11**	-348.960880	-348.773751	0.128064	0.171291	-
**TS6**	-4202.678196	-4204.600901	0.492157	0.598531	-67.71
**CP12**	-4202.702160	-4204.616270	0.494974	0.603381	-

**CP13**	-4202.673833	-4204.598073	0.494333	0.600298	-
**CP14**	-4202.679481	-4204.598768	0.494629	0.600182	-
**Br^-**	-2571.464366	-2574.182809	-0.016176	0.002360	-
**CP15**	-1631.080043	-1630.41209	0.500985	0.600784	-
**TS7**	-1631.055394	-1630.390103	0.498526	0.597139	-35.36
**CP16**	-1631.055263	-1630.385955	0.497741	0.598762	-
**TS8**	-1631.051404	-1630.385682	0.497427	0.597293	-131.22
**CP17**	-1631.076604	-1630.412663	0.500054	0.600722	-
**TS9**	-1631.059541	-1630.391962	0.497267	0.597017	-238.58
**CP18**	-1631.061307	-1630.391935	0.497986	0.598597	-
**TS10**	-1631.046434	-1630.379885	0.499025	0.596838	-132.14
**CP19**	-1631.085281	-1630.419378	0.500523	0.600911	-
**CP20**	-1067.402471	-1067.063803	0.342352	0.436427	-
**CP21**	-5270.132712	-5271.704433	0.856528	1.041772	-
**TS11**	-5270.126284	-5271.694065	0.860218	1.040587	-47.43
**CP22**	-5270.128607	-5271.697400	0.862234	1.042014	-
**TS12**	-5270.117356	-5271.685259	0.859915	1.040685	-76.19
**CP23**	-3154.746980	-3157.150430	0.215482	0.289129	-
**CP24**	-2115.356593	-2114.522501	0.620923	0.751099	-
**TS13**	-2115.319626	-2114.500796	0.622715	0.749371	-250.59
**$B(OH)_3$**	-252.470851	-252.474183	0.022275	0.053998	-
**CP25**	-1862.876731	-1862.057462	0.580517	0.695951	-
**TS14**	-1862.843223	-1862.033412	0.579111	0.693995	-341.97

**3b**	-581.232331	-580.926415	0.224341	0.281669	-
**TS15**	-2115.29099	-2114.478157	0.621196	0.746748	-77.97
**CP26**	-2115.301967	-2114.484665	0.622491	0.74828	-
**CP27**	-5270.137053	-5271.711533	0.863018	1.042375	-
**TS16**	-5270.124853	-5271.695483	0.861488	1.041143	-25.34
**CP28**	-5270.126897	-5271.698386	0.859717	1.042089	-
**TS17**	-5270.115842	-5271.689006	0.864254	1.041435	-57.29
**CP29**	-2115.360836	-2114.529268	0.625378	0.751492	-
**TS18**	-2115.320451	-2114.507901	0.625171	0.749632	-255.34
**CP30**	-1862.87121	-1862.055982	0.582041	0.695807	-
**TS19**	-1862.832804	-1862.031689	0.580329	0.693689	-291.59
**4b**	-581.227968	-580.924387	0.225959	0.281422	-
**CP0**	-1281.618680	-1281.094085	0.333678	0.413340	-
**CP1’**	-1857.306126	-1856.584038	0.571035	0.696475	-
**Ph-B(OH)$_2$**	-408.242565	-408.120427	0.092964	0.132733	-
**LiOH•3DMA**	-946.933217	-946.623846	0.347827	0.441744	-

[1]The electronic energy calculated by B3LYP in gas phase. [2]The electronic energy calculated by M06 in DMA solvent. [3]The thermal correction to Gibbs free energy calculated by B3LYP in gas phase. [4]The thermal correction to enthalpy calculated by B3LYP in gas phase. [5]The B3LYP calculated imaginary frequencies for the transition states.

B3LYP Geometries for All the Optimized Compounds and Transition States

CP1

C	1.85770800	2.47877300	-0.72458400
C	0.56226400	2.81291500	-0.30057400
C	-0.36769400	1.74458300	-0.17283100
C	0.07367600	0.43417700	-0.50371500
C	2.20797000	1.17447400	-1.07893800
C	-1.71974700	1.88961700	0.29335700
C	-0.80621400	-0.67992500	-0.35459300
C	-2.17983900	-0.48882400	-0.04738600
C	-2.58919300	0.83770200	0.32222600
C	-3.02656200	-1.63005800	-0.04849900
C	-2.42905000	-2.86708700	-0.32325200

C	-1.05362800	-2.99457100	-0.54013300
H	-2.05283100	2.86472300	0.63112800
H	2.58251900	3.27325300	-0.87785500
H	-3.60356800	0.99115300	0.67370900
H	-3.04581900	-3.75984200	-0.35481100
N	1.33092400	0.14736700	-0.99566700
N	-0.23122400	-1.92522400	-0.49366800
C	3.57045300	0.86228300	-1.63771200
H	4.09505800	1.77276700	-1.94412200
H	4.19207300	0.34518600	-0.89539400
H	3.47986900	0.19067800	-2.49812200
C	-0.41999000	-4.33166900	-0.80346900
H	-0.05713800	-4.39237700	-1.83688100
H	0.45678400	-4.46064300	-0.15502000
H	-1.12482500	-5.15273200	-0.63860700
C	0.23012300	4.23286800	-0.03350700
C	1.09598600	5.02695700	0.74165400
C	-0.91033300	4.84766400	-0.58466800
C	0.82824900	6.37656200	0.96707700
H	1.97632800	4.56929700	1.18496200
C	-1.17816200	6.19738000	-0.35864100
H	-1.57363000	4.26690300	-1.21863300
C	-0.31217400	6.96863300	0.41997800
H	1.50886300	6.96541000	1.57690100
H	-2.06086400	6.65060900	-0.80266400
H	-0.52234000	8.02021500	0.59570600
C	-4.48585600	-1.56490900	0.21192900
C	-5.31462100	-0.64323700	-0.45432900
C	-5.08733600	-2.46975100	1.10574100
C	-6.69041200	-0.62148000	-0.22596200
H	-4.87672200	0.04197900	-1.17410600
C	-6.46248200	-2.44733200	1.33401800
H	-4.46214200	-3.18181900	1.63762700
C	-7.27078500	-1.52157800	0.67017300
H	-7.31148400	0.09457600	-0.75805000
H	-6.90276900	-3.15047300	2.03641200
H	-8.34275200	-1.50367300	0.84754900
Ni	1.67832200	-1.82124300	-0.63614500
N	4.61428400	-1.63947300	1.46500800
C	3.41238200	-1.24737500	2.19327500
H	3.51281000	-0.21390100	2.54728100
H	2.53799500	-1.32664400	1.54142200
H	3.26559300	-1.90051500	3.06421200
C	5.84427400	-1.53805000	2.23609000
H	6.72246300	-1.68570700	1.60945900
H	5.91099600	-0.53972400	2.68547900
H	5.86610800	-2.27655500	3.05083200
C	4.55021700	-2.19923500	0.21822400
O	3.48829300	-2.30939100	-0.42662500
C	5.82369400	-2.72648700	-0.41527400
H	5.54920500	-3.18075800	-1.36794800
H	6.54969900	-1.92731300	-0.60877600
H	6.31574900	-3.48094100	0.20962300

1b

C	-2.60216900	-1.20504100	0.21007400
C	-1.96608100	-0.00346700	0.55025600
C	-2.59983400	1.20258400	0.22168400
C	-3.83432700	1.20962000	-0.42955300
C	-4.45697800	0.00527500	-0.76352400
C	-3.83669600	-1.20343500	-0.44115200
H	-2.12742000	-2.15106200	0.46266200
H	-2.12329000	2.14522300	0.48337900
H	-4.31140700	2.15568300	-0.67203000
H	-5.41963600	0.00865200	-1.26763000
H	-4.31561900	-2.14619300	-0.69268800
C	-0.60876200	-0.00806400	1.22270300
C	0.55203300	-0.00129300	0.20123800
H	-0.51789100	-0.89290900	1.86650000
H	-0.51771400	0.86802900	1.87835600
C	1.90416000	-0.00644700	0.89871600

H	0.47101400	-0.87619200	-0.45361400
H	0.47141600	0.88260900	-0.44145600
H	2.05707600	0.87892100	1.51788200
H	2.05677000	-0.90057500	1.50525200
Br	3.39910300	0.00255500	-0.40597900

DMA

N	0.59657500	-0.08374700	0.00077900
C	1.62391300	0.94636000	0.00003600
H	2.26021900	0.85701900	-0.89110600
H	1.13273800	1.91835600	0.00181800
H	2.26316300	0.85500200	0.88881700
C	1.08400000	-1.45043800	-0.00033000
H	0.26196600	-2.16459500	0.00123600
H	1.70247800	-1.64129500	-0.88909600
H	1.70578200	-1.64131300	0.88609000
C	-0.72901500	0.29340900	0.00002500
O	-1.06732500	1.47278200	-0.00032500
C	-1.77919900	-0.81327600	0.00009200
H	-2.75540600	-0.32745700	-0.00017200
H	-1.70317400	-1.45431000	-0.88571000
H	-1.70338500	-1.45376300	0.88633100

CP2

C	-0.93278300	-2.89168000	-1.64734800
C	-2.22477500	-2.47846700	-1.23889900
C	-2.38663800	-1.12323400	-0.87225800
C	-1.24008800	-0.26872100	-0.89064400
C	0.14281800	-2.00878200	-1.65224900
C	-3.64818800	-0.51348100	-0.54751800
C	-1.34406000	1.09568600	-0.52316100
C	-2.59513300	1.65887000	-0.12301900
C	-3.74656300	0.79929800	-0.19421200
C	-2.63794600	3.02462800	0.23993500
C	-1.43403900	3.76478100	0.13262700
C	-0.24860300	3.16390900	-0.27024400
H	-4.54875600	-1.11341000	-0.61755100
H	-0.76738200	-3.92714100	-1.92616100
H	-4.72400700	1.22199900	0.00995200
H	-1.42589600	4.81335100	0.41088400
N	0.01590300	-0.72057800	-1.27424800
N	-0.18038600	1.85135100	-0.58268400
C	1.51678800	-2.46651300	-2.07007700
H	1.51580000	-3.52531100	-2.34496400
H	1.87373100	-1.88468700	-2.92862300
H	2.24118800	-2.31650200	-1.26078000
C	1.03296200	3.94981600	-0.37059400
H	1.80723300	3.53353600	0.28684900
H	1.42473400	3.91328300	-1.39657000
H	0.88130400	4.99863100	-0.09923500
C	-3.32593100	-3.46945900	-1.22873700
C	-3.51286500	-4.33166400	-2.32656600
C	-4.17846100	-3.62589700	-0.11864700
C	-4.51863900	-5.29663800	-2.32269800
H	-2.87209700	-4.22287800	-3.19748000
C	-5.18405600	-4.59205000	-0.11476100
H	-4.03006500	-3.00049200	0.75661700
C	-5.36183200	-5.43132800	-1.21697300
H	-4.64815700	-5.94163100	-3.18832700
H	-5.82343000	-4.69678800	0.75825000
H	-6.14551400	-6.18419000	-1.21277000
C	-3.86397200	3.71163200	0.70712700
C	-4.68922700	3.16697400	1.71047700
C	-4.20878200	4.97548000	0.18946600
C	-5.81805400	3.84950100	2.16267100
H	-4.42394900	2.21259600	2.15556900
C	-5.33702600	5.65735200	0.64168600
H	-3.59212000	5.41092500	-0.59212200
C	-6.15025900	5.09722500	1.62999900
H	-6.43347400	3.40938200	2.94343100
H	-5.58562800	6.62658300	0.21638400
H	-7.02986200	5.62845700	1.98362300

Ni	1.35088600	0.77015500	-1.16672100
Br	3.89417500	2.02942100	2.19726500
C	2.27018300	0.90630700	2.48559800
C	2.60448200	-0.57575400	2.49645300
H	1.59591500	1.17105300	1.66948700
H	1.86338500	1.25410900	3.43631900
C	1.34125500	-1.41345500	2.80533400
H	3.00913300	-0.86633000	1.52068400
H	3.37883500	-0.78030000	3.24464700
H	0.92842500	-1.08969600	3.77102600
H	0.57818900	-1.20269800	2.04674700
C	1.62322500	-2.90191700	2.84524000
C	2.31524000	-3.47031200	3.92510700
C	1.21212800	-3.74112400	1.80137700
C	2.59494600	-4.83651200	3.95927200
H	2.63229600	-2.83664700	4.75130200
C	1.48887400	-5.11012700	1.83299800
H	0.65883600	-3.32335900	0.96318700
C	2.18295800	-5.66237100	2.91030500
H	3.12890200	-5.25769000	4.80748000
H	1.15433200	-5.74481800	1.01629800
H	2.39601100	-6.72785100	2.93705200
N	4.21595100	1.10896400	-3.50872800
C	2.96270600	0.97130200	-4.24855600
H	2.95285100	0.03611000	-4.82286100
H	2.11652200	0.97402600	-3.55804200
H	2.86427900	1.81114500	-4.94481000
C	5.37582300	1.43504300	-4.33116800
H	6.29205100	1.43301700	-3.74462300
H	5.47942100	0.69617600	-5.13545900
H	5.25193300	2.42434500	-4.78893400
C	4.27110300	0.87006600	-2.17622000
O	3.27596400	0.49842300	-1.52171000
C	5.58720700	1.04136300	-1.44043300
H	5.37405400	0.96576400	-0.37369200
H	6.30179900	0.25775000	-1.71879100
H	6.05158300	2.01210000	-1.64094500

TS1

C	1.43140000	-2.09029300	1.75864500
C	2.41755000	-1.09475900	1.65444700
C	2.02358100	0.15863000	1.11924700
C	0.67391000	0.31359100	0.68317200
C	0.12542700	-1.87425200	1.31303800
C	2.88142900	1.30812600	1.04983800
C	0.22944500	1.54828500	0.11791300
C	1.13038500	2.64669700	-0.01219800
C	2.45724200	2.48720700	0.51426700
C	0.64688200	3.85000100	-0.58672400
C	-0.71155100	3.88976100	-0.94375500
C	-1.54242800	2.77939300	-0.78636800
H	3.88200200	1.23741900	1.46150000
H	1.69846200	-3.06925500	2.14269900
H	3.12537100	3.34104800	0.50683400
H	-1.12104800	4.79221300	-1.38590000
N	-0.25725400	-0.69904700	0.77852400
N	-1.09073900	1.61558100	-0.27884700
C	-0.90046600	-2.97446600	1.37101700
H	-0.50623000	-3.85631000	1.88451600
H	-1.80549800	-2.63047600	1.87957600
H	-1.19776100	-3.27043100	0.35683500
C	-2.99214000	2.84929900	-1.18507400
H	-3.22639200	2.09674000	-1.94472900
H	-3.63743900	2.63358500	-0.32368800
H	-3.24788100	3.83979800	-1.57290500
C	3.80103000	-1.39993200	2.09434000
C	4.02747400	-2.02568800	3.33457000
C	4.91516200	-1.13790800	1.27392600
C	5.31613300	-2.36337900	3.74512700
H	3.18154700	-2.22897000	3.98558200
C	6.20410900	-1.47835600	1.68443300

H	4.76309200	-0.68477200	0.29869100
C	6.41167900	-2.09030500	2.92249500
H	5.46566000	-2.83682500	4.71217600
H	7.04721300	-1.27318000	1.02972600
H	7.41621500	-2.35469100	3.24155100
C	1.49132600	5.04764300	-0.81452900
C	2.73395200	4.96677900	-1.47113600
C	1.03384900	6.31966400	-0.42262400
C	3.49291200	6.11139400	-1.71344100
H	3.09147300	4.00080900	-1.81555600
C	1.79349900	7.46330700	-0.66369900
H	0.08132200	6.40228400	0.09373800
C	3.02840300	7.36500400	-1.30920800
H	4.44557000	6.02391200	-2.22965000
H	1.42243800	8.43306500	-0.34180000
H	3.62076000	8.25616500	-1.49836200
Ni	-2.05793700	-0.15674900	0.01760800
Br	-3.59689500	-0.78217300	-1.81622500
C	-2.07566000	-1.35417400	-3.42702400
C	-1.07979200	-2.29705400	-2.82411100
H	-1.70406100	-0.36090100	-3.67162700
H	-2.74488100	-1.75705700	-4.18457100
C	0.15336800	-2.52362700	-3.74344900
H	-0.72935100	-1.89136600	-1.86445900
H	-1.55299100	-3.26332000	-2.61044800
H	-0.18057400	-2.91124300	-4.71396000
H	0.62733900	-1.55243500	-3.93756400
C	1.16066900	-3.47590600	-3.13261300
C	1.24130200	-4.80938300	-3.55468300
C	2.01533500	-3.04996500	-2.10473200
C	2.15063800	-5.69418000	-2.97105000
H	0.58756800	-5.15666000	-4.35231000
C	2.92408200	-3.93026300	-1.51657300
H	1.96834800	-2.01874100	-1.76135900
C	2.99522900	-5.25694000	-1.94936700
H	2.20009600	-6.72367700	-3.31711700
H	3.57641600	-3.57794400	-0.72172500
H	3.70503500	-5.94304700	-1.49459400
N	-4.47798800	-0.25892900	3.59999300
C	-3.28264300	-0.36336000	4.42920700
H	-3.33324300	-1.25036200	5.07522600
H	-2.40971600	-0.43664900	3.78310300
H	-3.19732900	0.52497400	5.06615500
C	-5.71215400	0.01201200	4.31988900
H	-6.56046000	0.07454400	3.64083800
H	-5.91241800	-0.78335900	5.05094200
H	-5.63341000	0.96121800	4.86554600
C	-4.39304800	-0.49663800	2.25835200
O	-3.31073800	-0.81772100	1.74220900
C	-5.64535800	-0.35412500	1.41159000
H	-5.35560600	-0.49529400	0.36951500
H	-6.39333700	-1.11105800	1.67466900
H	-6.10796700	0.63278900	1.52347300

CP3

C	-0.30318100	3.42842300	0.10918500
C	0.97986000	2.89850800	0.07303900
C	1.10612600	1.47583400	0.08249100
C	-0.08931100	0.71786000	0.08488700
C	-1.44793700	2.60562800	0.12489700
C	2.35492900	0.77127000	0.13995300
C	-0.03472100	-0.72378200	0.08392600
C	1.21453100	-1.38959700	0.08100300
C	2.40643200	-0.59285900	0.13948000
C	1.19603900	-2.81772200	0.07033100
C	-0.04354500	-3.44308000	0.10497100
C	-1.24670700	-2.70905400	0.12054500
H	3.27331200	1.34308400	0.20872100
H	-0.43658300	4.50540300	0.09459900
H	3.36508300	-1.09423800	0.20813400
H	-0.09533700	-4.52699000	0.08925000

N	-1.33262700	1.27326800	0.09833000
N	-1.23221200	-1.37152900	0.09630900
C	-2.83099400	3.19255900	0.18146200
H	-2.82690100	4.24470400	-0.11796900
H	-3.52153000	2.63100400	-0.45513700
H	-3.22671900	3.12969200	1.20337200
C	-2.58260100	-3.39690500	0.17123000
H	-3.01401000	-3.31320200	1.17676900
H	-3.29352500	-2.92139500	-0.51168800
H	-2.49130600	-4.45864000	-0.07541200
C	2.15102700	3.81292700	0.02478300
C	3.12711600	3.69631300	-0.97975700
C	2.27285100	4.85088500	0.96353700
C	4.19512500	4.59133600	-1.03973800
H	3.03283700	2.91707600	-1.73072500
C	3.34524800	5.74073400	0.90624800
H	1.53032400	4.94603400	1.75110200
C	4.30953500	5.61396400	-0.09560000
H	4.93463900	4.49338100	-1.82979800
H	3.42800100	6.53179000	1.64648800
H	5.14351800	6.30872400	-0.14179000
C	2.43248300	-3.64185800	0.02372000
C	2.62672900	-4.67037200	0.96080300
C	3.40184500	-3.45083400	-0.97592800
C	3.76237600	-5.47801100	0.90707700
H	1.88975400	-4.82257600	1.74460400
C	4.53345700	-4.26425800	-1.03257700
H	3.25346700	-2.67893800	-1.72571800
C	4.71916500	-5.27782000	-0.08994800
H	3.90020200	-6.26246100	1.64614800
H	5.26724600	-4.10978900	-1.81894700
H	5.60265500	-5.90860300	-0.13372400
Ni	-2.81194600	-0.10986000	0.01247900
Br	-5.09605700	-0.18373400	-0.27943700

CP4

C	3.77353700	0.00034700	-0.28565000
C	2.31292200	0.00009700	-0.58306200
H	4.29971200	0.92720500	-0.07612000
H	4.30347300	-0.92741000	-0.09025800
C	1.42056400	-0.00054000	0.70022200
H	2.04230100	0.88106100	-1.18060300
H	2.04277200	-0.88009300	-1.18176100
H	1.67482300	-0.88111400	1.30261100
H	1.67498000	0.87940800	1.30348700
C	-0.05751400	-0.00023900	0.37789300
C	-0.75612100	-1.20341600	0.20457200
C	-0.75558800	1.20320500	0.20429400
C	-2.11073300	-1.20617900	-0.13173700
H	-0.23301200	-2.14791000	0.34085500
C	-2.11019100	1.20649100	-0.13202400
H	-0.23207200	2.14750300	0.34037200
C	-2.79298900	0.00029000	-0.30201100
H	-2.63437300	-2.15069000	-0.25618300
H	-2.63340900	2.15120500	-0.25669700
H	-3.84851500	0.00049200	-0.56069200

CP5

C	0.16726500	3.30275100	-0.54926200
C	1.48320900	3.16695400	-0.13833600
C	1.99438600	1.83885800	-0.01288400
C	1.10296800	0.77122900	-0.27287300
C	-0.67035800	2.18722900	-0.76473500
C	3.34037800	1.51995000	0.36739300
C	1.59818600	-0.58240000	-0.33703300
C	2.96942300	-0.84498100	-0.10490400
C	3.80436800	0.23693100	0.32859500
C	3.41986800	-2.18358700	-0.31646100
C	2.48912200	-3.10407500	-0.76938900
C	1.13294300	-2.76176100	-0.97596100
H	3.99528500	2.32166900	0.68845500
H	-0.23530900	4.29346400	-0.73671600

H	4.82630100	0.02389100	0.62084400
H	2.80831700	-4.11501800	-1.00255300
N	-0.22597300	0.94370000	-0.55218400
N	0.69340700	-1.52949800	-0.70874700
C	-2.05574700	2.40254700	-1.30967100
H	-1.99798900	3.04278300	-2.19846800
H	-2.51537400	1.45459600	-1.58659000
H	-2.70599200	2.90715500	-0.58639700
C	0.18269700	-3.78070900	-1.53455100
H	-0.31748900	-4.31837000	-0.72280100
H	-0.60315300	-3.29431000	-2.11622600
H	0.72056400	-4.49508000	-2.16673500
C	2.30498800	4.38209600	0.10474800
C	3.52617700	4.58982200	-0.55897600
C	1.83462600	5.37707800	0.97772000
C	4.25747300	5.75882000	-0.34923600
H	3.88963800	3.84378600	-1.25968500
C	2.57141200	6.54159500	1.19229700
H	0.89573400	5.22387000	1.50265400
C	3.78490100	6.73591300	0.52954000
H	5.19456000	5.90860600	-0.87841500
H	2.19766500	7.29581800	1.87923900
H	4.35783500	7.64408500	0.69471000
C	4.83125200	-2.60559700	-0.10981400
C	5.11547000	-3.70872300	0.71182400
C	5.89508000	-1.95627300	-0.75880100
C	6.42892900	-4.14276900	0.89021300
H	4.30141000	-4.21359400	1.22450100
C	7.20715600	-2.39689800	-0.58540600
H	5.68949800	-1.11970500	-1.42060200
C	7.47847900	-3.48849400	0.24222500
H	6.63151100	-4.99160500	1.53744200
H	8.01724000	-1.89017700	-1.10287000
H	8.50128400	-3.82841400	0.37909900
Ni	-1.16153100	-0.75785000	-0.03487700

Br	-1.86610700	-2.78006900	0.93796700
C	-2.65211800	0.11663700	0.85867500
C	-4.02388500	-0.14912200	0.24204100
H	-2.43288400	1.19112000	0.89447800
H	-2.62065800	-0.25825700	1.88599400
C	-5.16729400	0.57438800	1.00149600
H	-4.06243700	0.16387100	-0.81131300
H	-4.22077500	-1.22570900	0.24647800
H	-5.15458600	0.23863100	2.04689000
H	-4.96481400	1.65433800	1.01818000
C	-6.53208900	0.32440100	0.39758800
C	-7.24078800	-0.85140100	0.68735900
C	-7.10793400	1.23850800	-0.49560100
C	-8.48276800	-1.10494300	0.10438600
H	-6.81166400	-1.57332900	1.37896200
C	-8.35047400	0.99050500	-1.08247700
H	-6.57645300	2.15922500	-0.72936500
C	-9.04338500	-0.18393900	-0.78400300
H	-9.01519600	-2.02154300	0.34634700
H	-8.77875100	1.71682600	-1.76918900
H	-10.01225400	-0.37906500	-1.23651300

CP6

C	-2.26333800	-3.43787400	-0.14200600
C	-3.45296600	-2.72528200	-0.03936500
C	-3.36045500	-1.30170400	-0.04729600
C	-2.06677800	-0.72570300	-0.11297100
C	-1.01487000	-2.79683000	-0.22448700
C	-4.49387800	-0.42172300	-0.05828300
C	-1.91164000	0.70748300	-0.10599100
C	-3.05172500	1.54984300	-0.06376500
C	-4.34770300	0.93460000	-0.07124700
C	-2.83318500	2.95973600	-0.06763200
C	-1.51698800	3.39957300	-0.15812300
C	-0.43734700	2.49970900	-0.19797800
H	-5.48776400	-0.85391200	-0.08677700

H	-2.29181000	-4.52302500	-0.13510000
H	-5.22628800	1.56863600	-0.11004800
H	-1.31059200	4.46541100	-0.15628300
N	-0.91280700	-1.45653900	-0.20399500
N	-0.62770100	1.17282900	-0.15192200
C	0.25401400	-3.59657900	-0.32618000
H	0.04280000	-4.66735500	-0.40993400
H	0.83767600	-3.27344900	-1.19503300
H	0.88533400	-3.42739200	0.55343600
C	0.98186900	2.99241200	-0.27233100
H	1.56437300	2.59463300	0.56604300
H	1.46160400	2.62978400	-1.18870900
H	1.02881400	4.08577400	-0.25691100
C	-4.74077500	-3.45794500	0.07078100
C	-5.04678000	-4.48617700	-0.83741500
C	-5.65590400	-3.18462300	1.10286200
C	-6.23280600	-5.21096000	-0.72429800
H	-4.35475900	-4.70183200	-1.64688600
C	-6.84011400	-3.91259400	1.21742700
H	-5.42364000	-2.41354800	1.83179300
C	-7.13453600	-4.92649400	0.30327600
H	-6.45426100	-5.99591700	-1.44254000
H	-7.52988700	-3.69210700	2.02780700
H	-8.05828600	-5.49163700	0.39236800
C	-3.93498300	3.95298200	0.02130200
C	-4.88919000	3.89787600	1.05254300
C	-4.01286800	5.00682100	-0.90511000
C	-5.89117700	4.86319600	1.14924800
H	-4.82864700	3.10671300	1.79438200
C	-5.01778500	5.96936300	-0.81006300
H	-3.28913300	5.05633300	-1.71411100
C	-5.96113700	5.90080900	0.21712300
H	-6.61382200	4.80824000	1.95909000
H	-5.06576600	6.77107900	-1.54216800
H	-6.74362700	6.65097600	0.29184600
Ni	0.70386500	-0.32973400	-0.17618800
Br	2.86912200	-0.85038500	-0.13177800
C	4.34281100	0.69166400	0.07902100
C	5.72429100	0.07796600	0.06970000
H	4.06885800	1.16645200	1.02217100
H	4.13673100	1.35119800	-0.76504700
C	6.84586200	1.13687500	0.23431800
H	5.80884900	-0.65937600	0.87804100
H	5.88345000	-0.46806000	-0.86872300
H	6.76552000	1.87094400	-0.57777700
H	6.68269100	1.68498000	1.17126900
C	8.22941600	0.52142600	0.23519100
C	8.94314600	0.35230200	-0.95942600
C	8.81224500	0.06835600	1.42703800
C	10.20119700	-0.25235300	-0.96506600
H	8.50927300	0.70248800	-1.89393500
C	10.06977200	-0.53694800	1.42721300
H	8.27529200	0.19581100	2.36493200
C	10.76905600	-0.69995400	0.22948200
H	10.73957300	-0.36987600	-1.90215500
H	10.50520100	-0.87732100	2.36332700
H	11.74980800	-1.16814700	0.22757300

TS2

C	0.12441900	3.19276000	0.13026900
C	1.51833100	3.15639900	0.08374100
C	2.13598900	1.87331000	0.11427500
C	1.29508400	0.72932600	0.14667900
C	-0.64391700	2.02232300	0.11254000
C	3.55564200	1.66576800	0.15090200
C	1.86173700	-0.58840500	0.25468500
C	3.26956100	-0.76180300	0.18258100
C	4.09389400	0.41283000	0.15636000
C	3.78130000	-2.09077800	0.23326600
C	2.85943100	-3.11852800	0.42581200
C	1.48600800	-2.86427000	0.55428500

H	4.20893500	2.53018000	0.18462600
H	-0.38400200	4.15162700	0.10229500
H	5.17110500	0.29291700	0.18755800
H	3.20959400	-4.14474100	0.48539500
N	-0.07483900	0.80194600	0.06741400
N	0.98062500	-1.62063500	0.45685500
C	-2.14491300	2.07848200	0.08642200
H	-2.50942100	3.10199600	-0.04310000
H	-2.52529900	1.44965800	-0.72839700
H	-2.56450300	1.68474600	1.02249300
C	0.51981600	-3.98580400	0.81490900
H	-0.16941200	-3.71063600	1.62107400
H	-0.09727900	-4.18340500	-0.07037200
H	1.04224600	-4.90722900	1.08940100
C	2.27890900	4.42965700	0.00501700
C	3.24537800	4.65007700	-0.99280000
C	2.00194400	5.47385000	0.90469900
C	3.91547600	5.87006100	-1.08116000
H	3.45201800	3.86756500	-1.71716500
C	2.67425600	6.69264000	0.81737200
H	1.26601700	5.31643000	1.68862900
C	3.63488300	6.89553100	-0.17551200
H	4.65218600	6.02223700	-1.86562300
H	2.45033800	7.48273400	1.52934400
H	4.15879600	7.84497700	-0.24452500
C	5.22541600	-2.41197200	0.10610300
C	5.84551200	-3.26752300	1.03386700
C	5.99682300	-1.91702800	-0.96088500
C	7.19128000	-3.60912200	0.90547800
H	5.26757600	-3.64953800	1.87100700
C	7.34221100	-2.26119000	-1.09013400
H	5.52897700	-1.27851200	-1.70466900
C	7.94588600	-3.10679300	-0.15687400
H	7.65222300	-4.26483000	1.63957800
H	7.91733300	-1.87435300	-1.92733600
H	8.99442200	-3.37344900	-0.25781800
Ni	-0.89477300	-0.96022200	-0.12053500
Br	-2.29904800	-2.54884800	-1.07469900
C	-3.36362500	-1.58934700	0.56256400
C	-4.66632600	-1.06062200	0.01178300
H	-2.71986800	-0.82743300	1.01493700
H	-3.45668200	-2.45267500	1.21677200
C	-5.58030500	-0.52807400	1.14620300
H	-4.47011900	-0.25743700	-0.70633100
H	-5.19259900	-1.85140000	-0.53518800
H	-5.75936000	-1.33428500	1.86950200
H	-5.05647200	0.27017900	1.68739600
C	-6.90219600	-0.00853800	0.61994400
C	-7.99233000	-0.87170300	0.44280100
C	-7.05455500	1.33860400	0.26516200
C	-9.20064200	-0.40365000	-0.07538300
H	-7.89420300	-1.91972700	0.71879000
C	-8.26104700	1.81180300	-0.25328300
H	-6.22068300	2.02430000	0.40181200
C	-9.33874600	0.94103500	-0.42582300
H	-10.03549200	-1.08829100	-0.20097700
H	-8.36017900	2.86128900	-0.51830300
H	-10.27988000	1.30798400	-0.82642600

TS3

C	3.38005900	-1.95557500	0.15997500
C	3.61506900	-0.60016000	0.32373600
C	2.50333100	0.27853100	0.15314600
C	1.23093800	-0.30797900	-0.06136600
C	2.08655600	-2.47164300	-0.06793200
C	2.61713900	1.70621900	0.09848500
C	0.09252200	0.52829200	-0.34612100
C	0.24153100	1.93662300	-0.40989700
C	1.53880200	2.49698500	-0.17094600
C	-0.90063500	2.70338600	-0.79130900
C	-2.04339400	2.00535200	-1.15020400

C	-2.11983100	0.59934000	-1.07030300
H	3.59494400	2.15541300	0.22831600
H	4.20586100	-2.65583400	0.23640600
H	1.66625800	3.57026700	-0.25285900
H	-2.92425000	2.55088200	-1.47275400
N	1.02152400	-1.65933500	-0.10280500
N	-1.07935400	-0.11519600	-0.62312200
C	1.89933000	-3.94552900	-0.29686100
H	2.82664100	-4.38426700	-0.67832200
H	1.64537800	-4.47149900	0.63083200
H	1.09207900	-4.10754900	-1.01989300
C	-3.36992100	-0.11966500	-1.48670300
H	-3.11008200	-0.97944200	-2.11405200
H	-3.90679300	-0.49792300	-0.60882500
H	-4.03747500	0.55193400	-2.03495800
C	4.98821700	-0.11860000	0.62710600
C	5.23707800	0.72651500	1.72220700
C	6.07531600	-0.54568200	-0.15329000
C	6.53644300	1.13221200	2.02599700
H	4.41033300	1.04645200	2.35020600
C	7.37320700	-0.13325800	0.14761900
H	5.89550100	-1.18834300	-1.01066800
C	7.60813600	0.70623800	1.23836600
H	6.71170700	1.77718400	2.88276400
H	8.20058200	-0.46593700	-0.47315100
H	8.61952400	1.02567600	1.47383100
C	-0.89715000	4.18903600	-0.84379800
C	-1.33502900	4.85448500	-2.00100100
C	-0.50828800	4.95788300	0.26668600
C	-1.37473300	6.24771100	-2.04980000
H	-1.62632000	4.27257900	-2.87101100
C	-0.55443400	6.35107200	0.21857900
H	-0.19038000	4.45924700	1.17811000
C	-0.98504300	7.00062100	-0.94030300
H	-1.70703500	6.74467700	-2.95711300
H	-0.25923500	6.92917000	1.09007100
H	-1.01749800	8.08596100	-0.97786000
Ni	-0.94778500	-2.08329700	-0.24472400
Br	-1.08108800	-3.28033500	-2.49237100
C	-0.97145000	-3.66792900	1.08764600
C	-2.15050200	-3.00030800	1.39029200
H	-0.09661000	-3.50870000	1.71307500
H	-0.98928200	-4.56462800	0.47745300
C	-2.29690700	-2.06820700	2.57977800
H	-2.38321800	-2.20750900	-0.33720500
H	-3.08324200	-3.42277600	1.02241500
H	-2.56305100	-2.70061500	3.44333500
H	-1.32771300	-1.61561300	2.81528500
C	-3.35470500	-0.98995300	2.43943400
C	-4.71112200	-1.32956800	2.32440100
C	-3.00808500	0.36627500	2.44619200
C	-5.69044600	-0.34246200	2.20716900
H	-5.00598900	-2.37694500	2.33323200
C	-3.98492700	1.35880800	2.33383400
H	-1.96191800	0.64824800	2.54027600
C	-5.32994800	1.00756800	2.21145500
H	-6.73562000	-0.62746800	2.11917300
H	-3.69293600	2.40580800	2.34167200
H	-6.09201400	1.77748700	2.12532800

CP7

C	-2.86765700	1.63158600	-0.40043000
C	-1.71641900	2.34738800	-0.12007300
C	-0.49964000	1.61042600	-0.00927000
C	-0.57142800	0.20487600	-0.17751800
C	-2.86075800	0.22774100	-0.56028900
C	0.77126400	2.20252900	0.28787700
C	0.63927000	-0.58028300	-0.14995600
C	1.89422300	0.05403700	0.02183900
C	1.91521500	1.46096300	0.29776300
C	3.06318200	-0.75992300	-0.05606000

C	2.87912100	-2.11525900	-0.28072100
C	1.59909600	-2.68986700	-0.41243100
H	0.81240000	3.26178400	0.51378300
H	-3.80488300	2.16088100	-0.53745300
H	2.86324700	1.93202300	0.52987900
H	3.74527800	-2.76043500	-0.38340600
N	-1.73075900	-0.47497200	-0.41020400
N	0.49589200	-1.92968900	-0.32596400
C	-4.12845200	-0.47779700	-0.93943200
H	-4.90314800	0.24467200	-1.21207100
H	-3.95349600	-1.15628500	-1.77934400
H	-4.48057100	-1.10960700	-0.11777200
C	1.47560900	-4.16595700	-0.66988800
H	1.13679500	-4.68873800	0.23132800
H	0.73210500	-4.36503700	-1.44555700
H	2.44114200	-4.58122200	-0.97505600
C	-1.78793200	3.82611000	0.02424100
C	-0.99268800	4.67926900	-0.75970400
C	-2.70516900	4.39616400	0.92232100
C	-1.10981100	6.06427600	-0.64311100
H	-0.29936700	4.25445000	-1.48001200
C	-2.81529700	5.78130100	1.04306000
H	-3.32018300	3.74614900	1.53851100
C	-2.01817800	6.61928900	0.26078900
H	-0.49523600	6.70998900	-1.26443500
H	-3.52327400	6.20510900	1.74990300
H	-2.10603400	7.69828300	0.35276300
C	4.44151800	-0.21730700	0.06953200
C	5.34506300	-0.80131600	0.97283800
C	4.88758500	0.84280500	-0.73837700
C	6.65389900	-0.33049500	1.07501300
H	5.00985700	-1.61561500	1.60931400
C	6.19900100	1.30720000	-0.64016400
H	4.21061700	1.28659300	-1.46263400
C	7.08490400	0.72501200	0.26892300
H	7.33590500	-0.78751800	1.78668000
H	6.53026300	2.12068800	-1.27980000
H	8.10515400	1.09017400	0.34682400
Ni	-1.34348700	-2.50504100	-0.10554500
Br	-3.35727000	-3.42490900	0.54535700
H	-0.92351100	-3.85737200	0.17887800

CP13

C	3.20398400	1.52778100	-0.22213100
C	2.11064500	2.37188600	-0.31140400
C	0.82204800	1.76578800	-0.40378600
C	0.75049600	0.35098500	-0.31191800
C	3.05843200	0.12640000	-0.14420400
C	-0.38002500	2.50586100	-0.65126900
C	-0.52462100	-0.31179400	-0.47893800
C	-1.69613600	0.45549400	-0.70686700
C	-1.58281700	1.88081100	-0.79112300
C	-2.91883800	-0.24963700	-0.90950000
C	-2.85878400	-1.63271200	-0.95236600
C	-1.65197000	-2.32830100	-0.73267200
H	-0.31506000	3.58190700	-0.76382500
H	4.20447200	1.94676600	-0.18171800
H	-2.47118400	2.46201400	-1.01026700
H	-3.76547300	-2.20358400	-1.12489700
N	1.84766800	-0.44151000	-0.13296200
N	-0.52149800	-1.67151200	-0.45927400
C	4.28638800	-0.74068900	-0.08155800
H	5.13168000	-0.22632900	-0.54933800
H	4.57187000	-0.95537300	0.95585000
H	4.10526500	-1.68526200	-0.60213100
C	-1.62124000	-3.82989800	-0.80714300
H	-0.82945300	-4.15125900	-1.49164200
H	-1.39594100	-4.26469200	0.17311400
H	-2.58327400	-4.22228200	-1.14985200
C	2.31855600	3.84454600	-0.33505600
C	1.66107700	4.68798000	0.57647000

C	3.22560600	4.41292800	-1.24435400
C	1.90453200	6.06129200	0.57664600
H	0.97315000	4.26057400	1.30058000
C	3.46203800	5.78756200	-1.24823800
H	3.73149700	3.77282500	-1.96181500
C	2.80323000	6.61566600	-0.33738300
H	1.39542200	6.69764500	1.29538500
H	4.15974400	6.21128000	-1.96533200
H	2.98947800	7.68605100	-0.33879100
C	-4.22555200	0.43862200	-1.08230800
C	-5.03433500	0.15488800	-2.19449200
C	-4.69842400	1.34278500	-0.11606000
C	-6.27877300	0.76753600	-2.34306500
H	-4.67475700	-0.53569400	-2.95243400
C	-5.94597900	1.94899400	-0.26320200
H	-4.09382800	1.54575100	0.76360800
C	-6.73816500	1.66621800	-1.37828200
H	-6.88782700	0.54436400	-3.21480400
H	-6.30214600	2.63853900	0.49748500
H	-7.70824900	2.14189900	-1.49329800
Ni	1.33542200	-2.45420700	0.04900800
Br	2.25175400	-3.15770400	-2.12310300
C	2.50560100	-3.18162900	1.56206400
C	1.32119200	-2.72895800	2.14340500
H	3.41340800	-2.59257900	1.64903800
H	2.65246600	-4.23014200	1.32490100
C	1.25770600	-1.49146300	3.01978100
H	0.88517000	-3.80895200	-0.00337100
H	0.52790400	-3.45586900	2.30477100
H	1.61316600	-1.78744900	4.02226300
H	1.97571800	-0.74551300	2.66118000
C	-0.10618300	-0.84324700	3.18834500
C	-1.24643300	-1.60130200	3.49536700
C	-0.24439400	0.54844000	3.10540400
C	-2.48317600	-0.98946400	3.70221000
H	-1.16845400	-2.68185700	3.58516000
C	-1.47896400	1.16743000	3.31523000
H	0.62826200	1.15556300	2.87572400
C	-2.60554900	0.39954700	3.61305000
H	-3.35173300	-1.59814900	3.94055600
H	-1.55761600	2.24967500	3.24790900
H	-3.56711800	0.87666000	3.78411700

CP8

C	-3.47357600	-0.54772700	-0.11333500
C	-2.41777000	0.17011300	-0.49985600
H	-3.69799500	-0.71297900	0.93887900
H	-4.15732800	-0.99268700	-0.83138800
C	-1.42751000	0.82202100	0.43322800
H	-2.22439800	0.30858900	-1.56422400
H	-1.46704300	1.91264600	0.30224300
H	-1.72993400	0.62385800	1.47092800
C	0.00628000	0.35914300	0.21429400
C	1.03638100	1.28210800	-0.00199800
C	0.32627300	-1.00608900	0.23624300
C	2.35458700	0.85743900	-0.18504200
H	0.80499000	2.34498400	-0.02496500
C	1.64107800	-1.43464300	0.05492900
H	-0.46578200	-1.73475400	0.39048900
C	2.66128900	-0.50338400	-0.15638100
H	3.13964800	1.59066700	-0.35160900
H	1.86997800	-2.49717300	0.07655000
H	3.68566300	-0.83702900	-0.29940300

TS4

C	2.98528800	1.17931200	-0.74307900
C	1.92422900	2.06910700	-0.79961300
C	0.60922900	1.53428100	-0.66617600
C	0.48567800	0.14501400	-0.40519200
C	2.78486900	-0.19202700	-0.48999800
C	-0.58189100	2.31284800	-0.84398300
C	-0.83311300	-0.45154900	-0.31510900

C	-1.98954200	0.35993600	-0.46188100
C	-1.82121300	1.75531600	-0.74299500
C	-3.25898500	-0.28720000	-0.38606500
C	-3.27059100	-1.66773500	-0.24708400
C	-2.07139400	-2.40126600	-0.13457800
H	-0.48320800	3.36243800	-1.09570000
H	3.99994700	1.54501600	-0.86353200
H	-2.70127500	2.36482000	-0.91386100
H	-4.21868000	-2.19427600	-0.19657700
N	1.55865700	-0.68150200	-0.27871500
N	-0.88815300	-1.79161200	-0.13938600
C	3.94784100	-1.14510400	-0.48040700
H	4.88565600	-0.62377700	-0.69317300
H	4.04474000	-1.65290000	0.48500300
H	3.77665100	-1.92336000	-1.23382800
C	-2.08369400	-3.90135000	-0.00520000
H	-1.43249500	-4.34112000	-0.76793400
H	-1.69151100	-4.21052200	0.97092300
H	-3.09526300	-4.30316400	-0.11442700
C	2.19041000	3.51783700	-1.00416600
C	1.68167000	4.48037800	-0.11526100
C	3.00378800	3.94444000	-2.06650300
C	1.97744600	5.83238000	-0.28852800
H	1.07277300	4.15946900	0.72523100
C	3.29285500	5.29779500	-2.24184900
H	3.39323600	3.21052000	-2.76672700
C	2.78099100	6.24568100	-1.35353100
H	1.58388100	6.56330700	0.41279000
H	3.91553200	5.61134100	-3.07530300
H	3.00760900	7.29959400	-1.48943900
C	-4.54693200	0.45404600	-0.45458800
C	-5.54327000	0.05684000	-1.36130500
C	-4.81648700	1.52518100	0.41434400
C	-6.77119900	0.71712900	-1.40433700
H	-5.34265500	-0.76244200	-2.04615500

C	-6.04715900	2.18042600	0.37456000
H	-4.06402000	1.82850200	1.13690100
C	-7.02728900	1.78040700	-0.53637600
H	-7.52667500	0.40222200	-2.11903400
H	-6.24221300	3.00067600	1.06016800
H	-7.98444800	2.29356900	-0.56880400
Ni	1.14506700	-2.65258700	0.09835800
Br	1.54034200	-3.39572400	-2.17842400
C	1.90392000	-3.78659400	1.64155600
C	1.22708000	-2.64956200	2.14411800
H	2.98483500	-3.76057400	1.50850900
H	1.52215500	-4.78673800	1.83777200
C	1.96399400	-1.51760300	2.83895800
H	0.95943600	-4.08431900	0.20578200
H	0.21734100	-2.82903700	2.51245300
H	2.27400800	-1.89322700	3.83015000
H	2.89884900	-1.29769300	2.31252200
C	1.20387200	-0.22107600	3.06973900
C	-0.15442300	-0.20203800	3.41829700
C	1.88525500	1.00313200	3.01705700
C	-0.80975600	0.99901200	3.69634700
H	-0.71175200	-1.13232000	3.47878900
C	1.23695000	2.20672500	3.30064400
H	2.94035300	1.01351100	2.75314700
C	-0.11735000	2.21004000	3.63986400
H	-1.86329000	0.98569700	3.96464400
H	1.79276800	3.14026400	3.25802500
H	-0.62611000	3.14417100	3.86317200

CP9

C	0.26680900	3.26780800	-0.85088000
C	-0.97306700	3.06951700	-0.26634100
C	-1.41308100	1.72271700	-0.09558200
C	-0.52461000	0.67647300	-0.46399500
C	1.10160900	2.18823700	-1.19640200
C	-2.72894500	1.38689000	0.36469600

C	-0.96900000	-0.70603300	-0.36228400
C	-2.27369900	-0.99371400	0.12640700
C	-3.13979800	0.09264100	0.47452700
C	-2.67281400	-2.36470300	0.16977200
C	-1.79035800	-3.31067000	-0.33011300
C	-0.52172900	-2.93230900	-0.81842600
H	-3.41747600	2.19161400	0.59428600
H	0.62857400	4.27715600	-1.01662100
H	-4.15293100	-0.12537500	0.79252000
H	-2.06722400	-4.36060300	-0.32524100
N	0.72272100	0.92473400	-0.96141300
N	-0.12460200	-1.66484400	-0.79900200
C	2.43655500	2.43027700	-1.84161900
H	2.55420600	3.48481900	-2.10845300
H	3.25443500	2.15169200	-1.16845200
H	2.52695600	1.80806400	-2.73961300
C	0.44209200	-3.94476900	-1.37960300
H	0.91456800	-3.53711900	-2.27953200
H	1.23677100	-4.16711400	-0.65577600
H	-0.06097400	-4.88513800	-1.62409300
C	-1.78814600	4.24680500	0.13613700
C	-2.27556200	4.38327200	1.44716200
C	-2.03825200	5.27692700	-0.78542600
C	-2.99547800	5.51719200	1.82285400
H	-2.06906100	3.60709100	2.17864200
C	-2.76465100	6.40688500	-0.40996000
H	-1.67609800	5.17860300	-1.80506200
C	-3.24502400	6.53082600	0.89514100
H	-3.35632800	5.61100200	2.84348300
H	-2.95738000	7.18907200	-1.13907200
H	-3.80887700	7.41209700	1.18811700
C	-3.98953200	-2.80287000	0.70714800
C	-4.82191300	-3.63290900	-0.06168700
C	-4.40755200	-2.44346400	1.99979600
C	-6.04195900	-4.08268700	0.44295100
H	-4.51522000	-3.90913200	-1.06674100
C	-5.62503000	-2.89900700	2.50559600
H	-3.76436300	-1.82222800	2.61674000
C	-6.44738500	-3.71722200	1.72804800
H	-6.67711500	-4.71612700	-0.17022700
H	-5.92817900	-2.61887600	3.51081000
H	-7.39706800	-4.06859400	2.12191100
Ni	1.90837300	-0.59005400	-1.32019900
Br	1.83998900	-0.78285100	-3.73058800
C	3.52425100	-1.79245900	-0.35282900
C	2.67601900	-0.82101700	0.42182900
H	4.58334100	-1.52108200	-0.39794700
H	3.41748600	-2.83794000	-0.04779900
C	3.37922600	0.34431200	1.11429900
H	3.21365800	-1.81816600	-1.46116800
H	1.94773300	-1.30094300	1.08094000
H	4.09904300	0.80300500	0.42515000
H	2.63554300	1.11415600	1.35977600
C	4.10334500	-0.06819300	2.38699900
C	5.48860700	-0.27819000	2.39827100
C	3.39109500	-0.27947800	3.57697500
C	6.14445700	-0.69255500	3.55986800
H	6.06175500	-0.10757200	1.48927900
C	4.04080100	-0.69275200	4.74010000
H	2.31535500	-0.11413700	3.59040500
C	5.42222100	-0.90264900	4.73539000
H	7.22061400	-0.84604500	3.54623000
H	3.46983900	-0.84659400	5.65248000
H	5.93054100	-1.22228800	5.64121200

TS5

C	-2.21968500	-3.17102500	0.05391400
C	-3.12177500	-2.12370000	-0.06255400
C	-2.59768200	-0.79564000	-0.06615600
C	-1.18465300	-0.65112400	-0.01944200
C	-0.82947600	-2.93615900	0.10894500

C	-3.41274400	0.38275000	-0.04263400
C	-0.59322900	0.67374700	0.02785400
C	-1.43651900	1.81618200	0.03582800
C	-2.85781600	1.62671800	0.00727500
C	-0.81892800	3.09792300	0.14385400
C	0.55650700	3.13185300	0.30540900
C	1.33264700	1.95519900	0.29652800
H	-4.49111500	0.27352300	-0.02534600
H	-2.58383300	-4.19362500	0.07456300
H	-3.49839100	2.49909100	0.06493100
H	1.06177300	4.08630400	0.41133400
N	-0.33616600	-1.70267200	0.03808400
N	0.76390900	0.75725400	0.11755000
C	0.14888000	-4.07147800	0.25125900
H	-0.36386200	-5.01822400	0.44539300
H	0.74855600	-4.18432700	-0.65992000
H	0.84309000	-3.85405400	1.06961000
C	2.81884100	2.01868600	0.50132900
H	3.09997600	1.34557300	1.31953700
H	3.36400700	1.68231000	-0.38696600
H	3.13756800	3.03837100	0.73669800
C	-4.57722900	-2.41635100	-0.16114300
C	-5.35334700	-1.91163600	-1.21830500
C	-5.19303300	-3.25233200	0.78479400
C	-6.70676500	-2.23233500	-1.32311000
H	-4.88662600	-1.28355000	-1.97191400
C	-6.54799300	-3.56713900	0.68236700
H	-4.60676000	-3.63994900	1.61335200
C	-7.30912400	-3.05864500	-0.37191600
H	-7.28936700	-1.84069100	-2.15259600
H	-7.00930100	-4.20763700	1.42913100
H	-8.36416600	-3.30533100	-0.45273000
C	-1.58495200	4.37248400	0.10851500
C	-1.41390900	5.32437600	1.12710900
C	-2.44780700	4.67645000	-0.95828200
C	-2.09341700	6.54176200	1.08538900
H	-0.75847100	5.09696800	1.96328900
C	-3.12116100	5.89713900	-1.00209400
H	-2.57318000	3.96099400	-1.76618600
C	-2.94859700	6.83233400	0.02065800
H	-1.95608000	7.26192800	1.88734500
H	-3.77679700	6.11955800	-1.83963500
H	-3.47622400	7.78151600	-0.01279400
Ni	1.80469400	-0.98732100	0.01261900
Br	2.27852600	-1.21023300	2.37753200
C	2.14511700	-0.81940700	-2.01239400
C	3.24837400	-1.50740100	-1.47715100
H	2.55352800	-2.23432900	0.02417000
H	3.31423400	-2.57276800	-1.70304400
C	4.56103100	-0.87017600	-1.15871000
C	5.33648000	-1.30691900	-0.07166800
C	5.08112200	0.13509900	-1.99069300
C	6.58781000	-0.74767800	0.17786500
H	4.93012800	-2.05377500	0.60328100
C	6.33418100	0.69797700	-1.73683300
H	4.50917800	0.46292200	-2.85478600
C	7.09249500	0.25828100	-0.65167800
H	7.16634400	-1.09075900	1.03149700
H	6.71896600	1.47306300	-2.39498000
H	8.06832000	0.69356200	-0.45297200
H	2.24255100	0.25741100	-2.14258200
C	1.12816700	-1.48965000	-2.90569600
H	1.03154000	-2.55578000	-2.67369400
H	0.13742300	-1.03281200	-2.80802700
H	1.42210800	-1.40541100	-3.96292900

CP10

C	-2.87946300	-2.11460400	-0.28139800
C	-3.06325700	-0.75930600	-0.05627900
C	-1.89412300	0.05440200	0.02174400
C	-0.63930500	-0.58013800	-0.15025100

C	-1.59955800	-2.68939600	-0.41347700
C	-1.91483000	1.46125500	0.29800700
C	0.57155900	0.20479600	-0.17744900
C	0.50003600	1.61035600	-0.00908200
C	-0.77075900	2.20263700	0.28817200
C	1.71695700	2.34710400	-0.11981000
C	2.86811800	1.63107700	-0.39994000
C	2.86098100	0.22722100	-0.55975000
H	-2.86275100	1.93244700	0.53030400
H	-3.74574800	-2.75958500	-0.38420200
H	-0.81172200	3.26185600	0.51427800
H	3.80546300	2.16020000	-0.53683700
N	-0.49621000	-1.92949400	-0.32680300
N	1.73080300	-0.47524500	-0.40993900
C	-1.47644900	-4.16542100	-0.67154500
H	-2.44200700	-4.58022100	-0.97727900
H	-0.73270300	-4.36443000	-1.44698400
H	-1.13824000	-4.68872500	0.22960400
C	4.12863400	-0.47859900	-0.93858200
H	4.48034500	-1.11060800	-0.11691200
H	3.95374700	-1.15687100	-1.77868700
H	4.90358600	0.24372900	-1.21088300
C	-4.44147700	-0.21651000	0.06966000
C	-4.88742200	0.84410900	-0.73765000
C	-5.34506000	-0.80082600	0.97273200
C	-6.19875100	1.30868600	-0.63908700
H	-4.21044300	1.28814600	-1.46174300
C	-6.65379900	-0.32983500	1.07526200
H	-5.00994500	-1.61550500	1.60876500
C	-7.08468600	0.72617900	0.26976600
H	-6.52991300	2.12257300	-1.27826800
H	-7.33582800	-0.78710900	1.78674400
H	-8.10486200	1.09148900	0.34795100
C	1.78866900	3.82583000	0.02423700
C	2.70613500	4.39598200	0.92202100
C	0.99342200	4.67892400	-0.75979100
C	2.81647700	5.78113500	1.04239700
H	3.32116100	3.74604000	1.53827900
C	1.11075900	6.06393700	-0.64355500
H	0.29994200	4.25402800	-1.47990000
C	2.01935300	6.61904200	0.26005500
H	3.52464000	6.20500800	1.74901400
H	0.49617300	6.70957800	-1.26494000
H	2.10738000	7.69804500	0.35174100
Ni	1.34304100	-2.50538200	-0.10601100
Br	3.35625200	-3.42561700	0.54599600
H	0.92264400	-3.85783500	0.17713900

CP14

C	2.98482700	-2.58819300	-0.12851900
C	3.53014400	-1.32897900	0.06308500
C	2.63981600	-0.21209600	0.02924200
C	1.25386600	-0.48388200	-0.11775400
C	1.59352400	-2.77801800	-0.27745000
C	3.07460800	1.15221700	0.05337600
C	0.30959200	0.60604600	-0.24534200
C	0.78335100	1.94241700	-0.23503200
C	2.18708600	2.17983600	-0.07363300
C	-0.16632500	2.98342400	-0.47711400
C	-1.45027000	2.59933300	-0.81324800
C	-1.85033000	1.24191200	-0.81641100
H	4.13531500	1.36217600	0.12868000
H	3.63354700	-3.45851200	-0.13535500
H	2.54757200	3.20170500	-0.10078600
H	-2.19386000	3.35384200	-1.05131100
N	0.75394800	-1.74322100	-0.22612400
N	-1.00062700	0.27569800	-0.45797300
C	1.02711700	-4.15443500	-0.49421700
H	1.81097000	-4.85338700	-0.80187600
H	0.57022600	-4.53524600	0.42713600
H	0.24012100	-4.11789500	-1.25359900

C	-3.24680800	0.89517100	-1.24674600
H	-3.31960800	-0.16284800	-1.50302000
H	-3.97308400	1.13015700	-0.46238800
H	-3.51001600	1.49227400	-2.12804500
C	4.99651500	-1.18631500	0.26677800
C	5.51539300	-0.49262500	1.37335600
C	5.89410000	-1.79261400	-0.62761800
C	6.89262500	-0.40703200	1.57657400
H	4.83430300	-0.03908900	2.08795800
C	7.27119200	-1.70064500	-0.42660300
H	5.50641400	-2.32182400	-1.49376400
C	7.77489200	-1.00803600	0.67612500
H	7.27602600	0.12473300	2.44315600
H	7.95038400	-2.16795900	-1.13437000
H	8.84758000	-0.93798500	0.83370500
C	0.18361000	4.42757500	-0.41754000
C	-0.13805000	5.27694800	-1.48892700
C	0.79061600	4.98222100	0.72218800
C	0.14621400	6.64099400	-1.42609900
H	-0.59655000	4.85853300	-2.38061200
C	1.06770500	6.34763800	0.78639200
H	1.02511900	4.34270800	1.56859600
C	0.74908700	7.18073500	-0.28823800
H	-0.10119900	7.28130300	-2.26834400
H	1.52791800	6.76143300	1.67954800
H	0.96849600	8.24366300	-0.23854800
Ni	-1.35345000	-1.76408100	-0.03025300
Br	-2.20053600	-2.54326700	-2.18724900
C	-1.44713300	-1.55232400	2.04553400
C	-2.73750700	-1.85356300	1.59978300
H	-1.42203200	-3.17931200	0.15336500
H	-3.03152200	-2.90113000	1.60536200
C	-3.88040300	-0.92036900	1.52089200
C	-5.04343500	-1.31543100	0.83334000
C	-3.89140800	0.32148000	2.18130400
C	-6.16635400	-0.49320200	0.79044400
H	-5.04530200	-2.26844700	0.31114400
C	-5.01631100	1.14597400	2.13836400
H	-3.02344300	0.63376200	2.75492000
C	-6.15840900	0.74502400	1.44062500
H	-7.05004000	-0.81737000	0.24706000
H	-5.00509400	2.09817900	2.66322400
H	-7.03658900	1.38467200	1.41309600
C	-0.58047200	-2.55034300	2.77688200
H	-0.69404400	-2.42406200	3.86403200
H	-0.86235700	-3.57914700	2.53088100
H	0.48286000	-2.42186500	2.54715200
H	-1.20600300	-0.50683700	2.23972700

CP11

C	1.38391400	-0.49861000	0.00044100
C	2.40112400	0.37628500	-0.00041900
H	2.19568400	1.44678100	-0.00143300
H	1.62975800	-1.56177600	0.00130600
C	-0.05802200	-0.20498800	0.00025400
C	-0.96757000	-1.27769500	0.00000300
C	-0.58488300	1.10091000	0.00033700
C	-2.34521700	-1.06134700	-0.00025400
H	-0.58279500	-2.29523200	-0.00002600
C	-1.95962400	1.31901900	0.00008200
H	0.08651500	1.95479200	0.00067900
C	-2.84909800	0.23971200	-0.00023000
H	-3.02416900	-1.91020100	-0.00046700
H	-2.34098300	2.33700600	0.00016300
H	-3.92170500	0.41355200	-0.00041600
C	3.85180100	-0.00729500	-0.00014400
H	3.98096400	-1.09493500	0.00083500
H	4.37104400	0.39623700	-0.88065600
H	4.37113300	0.39782000	0.87958800

TS6

C	1.46089600	-2.95240000	-0.95374100

C	2.40456400	-1.95710100	-0.75304800
C	1.92695000	-0.63267200	-0.52202300
C	0.52081700	-0.44759200	-0.44291200
C	0.07890900	-2.68217900	-0.87379600
C	2.78007700	0.51515300	-0.43898100
C	-0.02397600	0.88195200	-0.25379300
C	0.85697400	1.99240100	-0.16253900
C	2.26883300	1.76688800	-0.26976100
C	0.28089600	3.29131200	-0.03672900
C	-1.10022800	3.38161700	-0.08183800
C	-1.91519100	2.23586300	-0.17745300
H	3.84984300	0.38077400	-0.55157800
H	1.78696900	-3.96960100	-1.14688700
H	2.93396500	2.62233700	-0.25082100
H	-1.57801900	4.35325400	-0.00830500
N	-0.36854800	-1.45948100	-0.58934300
N	-1.38310100	1.00893600	-0.22313600
C	-0.93095000	-3.77244900	-1.10825500
H	-1.45346300	-4.01694300	-0.17666800
H	-1.68516300	-3.43221200	-1.82469900
H	-0.45089300	-4.68073500	-1.48461900
C	-3.41055700	2.37985900	-0.21445800
H	-3.69296200	3.40210000	-0.48359100
H	-3.83377600	1.67789700	-0.94048600
H	-3.84892400	2.16329700	0.76759300
C	3.85211800	-2.29640200	-0.79972000
C	4.71006300	-1.97439800	0.26566200
C	4.37752800	-2.99637900	-1.89815700
C	6.05561300	-2.34012300	0.22999300
H	4.31243500	-1.45626200	1.13376800
C	5.72475800	-3.35591900	-1.93526800
H	3.72673300	-3.24202700	-2.73283400
C	6.56797200	-3.02923500	-0.87140700
H	6.70267600	-2.09172800	1.06695400
H	6.11573700	-3.88892500	-2.79767700
H	7.61695500	-3.31096900	-0.89944700
C	1.09222600	4.52792200	0.12116000
C	0.88008800	5.62245500	-0.73311400
C	2.04123800	4.65136600	1.15025100
C	1.60334900	6.80383100	-0.56949200
H	0.15720300	5.53568700	-1.53959300
C	2.75841000	5.83591300	1.31705500
H	2.20022100	3.82227000	1.83398900
C	2.54407600	6.91459700	0.45629400
H	1.43313200	7.63672600	-1.24617100
H	3.48131300	5.91752600	2.12421900
H	3.10582300	7.83562300	0.58508000
Ni	-2.40832400	-0.76464800	-0.27546500
Br	-3.35592200	-0.56299900	-2.50585400
C	-2.74434200	-0.75732900	1.83438800
C	-3.83545700	-1.39626300	1.24603000
H	-3.03974500	-2.05226700	-0.42624300
H	-3.89262700	-2.47896900	1.33437600
H	-2.83425300	0.31313100	2.01127700
C	-1.64168700	-1.40386900	2.57193500
C	-0.68455100	-0.59223200	3.20957300
C	-1.50083500	-2.79959300	2.69711800
C	0.37134700	-1.14556700	3.93230700
H	-0.77850200	0.48908400	3.13671500
C	-0.44547400	-3.35329300	3.41785800
H	-2.22827400	-3.46036800	2.23458600
C	0.49960300	-2.53152200	4.03932300
H	1.09203500	-0.49164800	4.41716300
H	-0.36379300	-4.43419200	3.50142700
H	1.31893600	-2.96706200	4.60466300
C	-5.16620900	-0.74419500	0.96748200
H	-5.10371900	0.34598100	1.00396200
H	-5.54422700	-1.02666100	-0.01972900
H	-5.90084300	-1.06869300	1.71918200

CP12

C	3.40502700	-1.90761300	-0.43883700
C	3.60400900	-0.58360500	-0.08530600
C	2.48787000	0.30049600	-0.20620100
C	1.24308300	-0.27053100	-0.57523300
C	2.14930200	-2.37946200	-0.88732400
C	2.57736700	1.72381200	-0.06145400
C	0.07886600	0.57961200	-0.68139800
C	0.20978500	1.98613100	-0.58524600
C	1.49718500	2.53127400	-0.26990900
C	-0.94697600	2.77738000	-0.87068200
C	-2.08856300	2.11439000	-1.28820100
C	-2.16194200	0.70540600	-1.30406300
H	3.54172300	2.16406500	0.16555700
H	4.23061700	-2.60939400	-0.37371300
H	1.61057500	3.60796800	-0.21876700
H	-2.97641000	2.68175000	-1.54772400
N	1.07937500	-1.57944900	-0.91487200
N	-1.11371300	-0.03451700	-0.93082800
C	2.00763900	-3.78962500	-1.38282100
H	1.93409200	-3.79202000	-2.47804500
H	2.87236900	-4.39615200	-1.09772100
H	1.08495600	-4.24768100	-1.01484600
C	-3.41287600	0.01688700	-1.77179200
H	-3.39054100	-1.04506300	-1.51786100
H	-4.30548100	0.47715300	-1.33971700
H	-3.48239400	0.10334400	-2.86485800
C	4.94188800	-0.13303900	0.38075400
C	5.09326400	0.56487800	1.59141000
C	6.09146400	-0.44348500	-0.36450500
C	6.35849500	0.94223900	2.04071300
H	4.21681000	0.78967100	2.19255900
C	7.35543000	-0.05941000	0.08276800
H	5.98743900	-0.97284900	-1.30759000
C	7.49298300	0.63414100	1.28680000
H	6.45807300	1.47153200	2.98444700

H	8.23264600	-0.30020400	-0.51146400
H	8.47782200	0.93111900	1.63653600
C	-0.95607500	4.25999000	-0.75951200
C	-1.38945300	5.04634200	-1.83938400
C	-0.58543800	4.90076500	0.43493900
C	-1.44176700	6.43595600	-1.73169000
H	-1.66886400	4.56370400	-2.77191800
C	-0.64618700	6.28989400	0.54353300
H	-0.27303200	4.30441700	1.28740300
C	-1.07099100	7.06185800	-0.53998400
H	-1.77042600	7.02930900	-2.58048000
H	-0.36683700	6.76867100	1.47806500
H	-1.11438900	8.14411000	-0.45522800
Ni	-1.15247400	-1.98520400	-0.61334700
Br	-1.54609900	-4.24292200	-1.08824300
C	-1.68035700	-2.86330100	3.45656700
C	-1.11221000	-2.47924900	2.07568200
H	-2.41142400	-3.67484400	3.36146100
H	-0.88099400	-3.20818800	4.12421200
C	-2.19906600	-2.02836300	1.08378800
H	-2.18151600	-2.01489400	3.93499500
H	-0.59750700	-3.34907000	1.65578600
H	-0.34884000	-1.69941900	2.20729700
H	-2.89167000	-2.85225600	0.90357400
C	-2.96607000	-0.81403900	1.48392500
C	-4.36682600	-0.78591300	1.32970100
C	-2.36436000	0.32049600	2.06658200
C	-5.12667100	0.31618600	1.72127500
H	-4.86170900	-1.65481100	0.90108400
C	-3.11947300	1.42368200	2.46161400
H	-1.28768400	0.33699300	2.21300800
C	-4.50644600	1.43221200	2.28835900
H	-6.20665600	0.29851400	1.59420700
H	-2.62416000	2.28108100	2.91187200
H	-5.09496600	2.29085600	2.60080100

Br^-

Br	0.00000000	0.00000000	0.00000000

CP15

C	2.91045300	-2.80327300	-0.32691500
C	3.46682200	-1.57889800	0.00645600
C	2.60298300	-0.44041200	-0.04911800
C	1.23685900	-0.66184100	-0.34203300
C	1.54173600	-2.95931300	-0.62412800
C	3.06319100	0.90872700	0.09671300
C	0.34254800	0.45994700	-0.46009500
C	0.82057100	1.78004000	-0.28740400
C	2.21369800	1.96989500	-0.01724000
C	-0.11192800	2.85118300	-0.46697700
C	-1.39469500	2.51416900	-0.87450800
C	-1.80036700	1.17506200	-1.03769400
H	4.11985800	1.07926300	0.26301900
H	3.53559700	-3.68935000	-0.32649800
H	2.59724800	2.98002900	0.06051100
H	-2.12451100	3.29909500	-1.04073500
N	0.70640300	-1.90807000	-0.58171900
N	-0.94902000	0.16964700	-0.79481700
C	1.03616000	-4.32628600	-0.98832100
H	1.87378500	-4.98376400	-1.23295400
H	0.48541300	-4.78183300	-0.15917200
H	0.36757000	-4.27820800	-1.85053100
C	-3.20700000	0.86089400	-1.46801300
H	-3.22013100	0.05506400	-2.20851000
H	-3.82191600	0.55172700	-0.61332800
H	-3.67973300	1.74048800	-1.91225100
C	4.90062100	-1.49333300	0.37254900
C	5.31190800	-0.84489200	1.55099800
C	5.86933400	-2.11393000	-0.43523100
C	6.65900800	-0.81957700	1.90889800
H	4.57233000	-0.38987600	2.20371200
C	7.21682100	-2.07366800	-0.08113500
H	5.56692700	-2.60487500	-1.35609300
C	7.61447200	-1.42862900	1.09209200
H	6.96152700	-0.32898600	2.82936700
H	7.95598400	-2.54463300	-0.72233600
H	8.66397900	-1.40247900	1.36994100
C	0.24312300	4.27537000	-0.25509700
C	-0.07479200	5.23091700	-1.23566400
C	0.84772500	4.70351300	0.94028600
C	0.21836400	6.57833700	-1.03182900
H	-0.52908600	4.91130800	-2.16948500
C	1.12647500	6.05394600	1.14611100
H	1.06880000	3.98247600	1.72215600
C	0.81761400	6.99322200	0.15957300
H	-0.01988300	7.30360400	-1.80422600
H	1.57962300	6.37304400	2.08010700
H	1.04083900	8.04389700	0.31958500
Ni	-1.23049000	-1.79379100	-0.64995900
C	-1.90774000	-3.55129000	-0.37560900
C	-3.17214900	-2.77305800	-0.32132700
H	-1.58783300	-4.01091600	0.56144800
H	-1.78831700	-4.21272600	-1.23283800
C	-3.93283100	-2.74586200	1.02263300
H	-2.92472800	-1.64147800	-0.53615300
H	-3.83966000	-2.97268100	-1.16654900
H	-4.34887900	-3.75041900	1.16823100
H	-3.21300100	-2.58228700	1.83297700
C	-5.03317500	-1.70779100	1.08447000
C	-6.23959500	-1.90296900	0.39612600
C	-4.86098100	-0.52244900	1.81301600
C	-7.24389900	-0.93497300	0.42717300
H	-6.40054900	-2.82598200	-0.15741000
C	-5.86572100	0.44848500	1.84787500
H	-3.94146400	-0.36665100	2.37436500
C	-7.05861900	0.24513300	1.15196500
H	-8.17508700	-1.10622800	-0.10552100

H	-5.72065900	1.35531700	2.42867200
H	-7.84397900	0.99479000	1.18352700

TS7

C	2.93004000	-2.63047600	-0.53729400
C	3.38334900	-1.42335100	-0.03189700
C	2.46112500	-0.33044500	-0.06280500
C	1.13255200	-0.60303700	-0.46663000
C	1.59824600	-2.82227800	-0.96023700
C	2.83615700	1.02722400	0.19831200
C	0.19346100	0.47859200	-0.59331500
C	0.59406500	1.81223400	-0.34568300
C	1.94758100	2.05220900	0.05504700
C	-0.36251800	2.84851200	-0.58719600
C	-1.58749700	2.46858500	-1.11524800
C	-1.93268700	1.11825900	-1.32064400
H	3.86460700	1.23828100	0.46549200
H	3.60979400	-3.47438800	-0.59200300
H	2.27279000	3.07428100	0.20652200
H	-2.33109400	3.22700400	-1.33405500
N	0.68495300	-1.84577000	-0.84342100
N	-1.05880900	0.14273600	-1.02835200
C	1.24018900	-4.14613400	-1.58204500
H	2.13173700	-4.59064200	-2.03264600
H	0.86219800	-4.86321600	-0.84526600
H	0.48960500	-4.02253900	-2.36435600
C	-3.29744300	0.77178800	-1.84674300
H	-3.23793100	-0.00800200	-2.61117800
H	-3.94068100	0.40179600	-1.03925300
H	-3.77436700	1.65415200	-2.28161100
C	4.77427300	-1.29868300	0.46441100
C	5.04570700	-0.72913600	1.72149000
C	5.84279600	-1.80205600	-0.29777400
C	6.35305400	-0.66813300	2.20157700
H	4.22817400	-0.36396700	2.33664400
C	7.15005500	-1.72662000	0.17978900
H	5.64961000	-2.22850000	-1.27826100
C	7.40811800	-1.16197400	1.43090900
H	6.54679800	-0.23980000	3.18060700
H	7.96708600	-2.10672800	-0.42628200
H	8.42645000	-1.10824700	1.80434400
C	-0.08838400	4.28105400	-0.31868900
C	-0.34771100	5.24243900	-1.31067500
C	0.37940500	4.70900100	0.93656600
C	-0.13022400	6.59605100	-1.05830000
H	-0.69572700	4.92392200	-2.28945000
C	0.58248400	6.06494100	1.18945400
H	0.55310100	3.98197600	1.72492500
C	0.33328900	7.01061100	0.19219300
H	-0.32142500	7.32631500	-1.83903300
H	0.92992000	6.38310200	2.16803000
H	0.49752800	8.06580400	0.38956700
Ni	-1.36103100	-1.75830500	-0.82562900
C	-1.68452300	-3.79184000	-0.60902400
C	-2.93530600	-3.29883600	-0.26266500
H	-0.99723700	-4.10894200	0.17095700
H	-1.53301400	-4.20214900	-1.60232100
C	-3.42379800	-3.14557600	1.16270900
H	-2.76767500	-1.44851000	-0.72648200
H	-3.71259000	-3.28672000	-1.02467100
H	-3.90688400	-4.10380200	1.41062300
H	-2.56826700	-3.04610900	1.83922400
C	-4.41112700	-2.01479100	1.37616600
C	-5.71570700	-2.10153200	0.87086400
C	-4.03275800	-0.85977600	2.07190200
C	-6.61757800	-1.05058900	1.04419800
H	-6.03594000	-3.00168900	0.34970600
C	-4.93432800	0.19210400	2.25099000
H	-3.02968000	-0.78789800	2.48711900
C	-6.22798200	0.10061000	1.73357800
H	-7.62710900	-1.13577800	0.65215400

H	-4.62932700	1.07665600	2.80344800
H	-6.93282400	0.91440100	1.87779700

CP16

C	-0.50698800	3.07051600	-1.08876000
C	0.69282900	3.00993400	-0.39550200
C	1.23866300	1.71091000	-0.15719200
C	0.51087800	0.59274900	-0.63404700
C	-1.18262200	1.91400100	-1.52355200
C	2.45979700	1.48068000	0.55656900
C	1.07820700	-0.72771900	-0.51660200
C	2.35655800	-0.90372600	0.06365400
C	2.99633700	0.23171500	0.65924700
C	2.91918600	-2.21807200	0.05019900
C	2.15536000	-3.22887500	-0.51535600
C	0.87224200	-2.99772400	-1.04675800
H	2.95623900	2.32308700	1.02259200
H	-0.92545600	4.03868300	-1.34090300
H	3.91930200	0.08140300	1.20608000
H	2.56537700	-4.23044800	-0.58047100
N	-0.70316500	0.68836700	-1.26101200
N	0.34147800	-1.76375800	-1.02648100
C	-2.43086400	2.07276600	-2.35197100
H	-2.37455500	1.45774600	-3.25511500
H	-3.33543400	1.79385900	-1.80183800
H	-2.54904000	3.11370400	-2.66134600
C	0.11418900	-4.14185700	-1.66116600
H	-0.68136100	-4.48628800	-0.99007800
H	-0.35536000	-3.84423700	-2.60207600
H	0.78539000	-4.98351100	-1.85142400
C	1.35721900	4.26624300	0.02973600
C	2.69655600	4.53761700	-0.30240800
C	0.62145900	5.23728900	0.73093300
C	3.28319800	5.74809300	0.06433900
H	3.26830700	3.81524800	-0.87777700
C	1.21591200	6.44036900	1.10794700
H	-0.41161600	5.03616100	1.00114200
C	2.54729700	6.69907100	0.77479800
H	4.31378900	5.95098400	-0.21169300
H	0.63933100	7.17520600	1.66197300
H	3.00807700	7.63898700	1.06386100
C	4.26718600	-2.53009600	0.58375400
C	4.43442100	-3.61252200	1.46489800
C	5.39996700	-1.80040400	0.18077000
C	5.69998800	-3.94508000	1.94501400
H	3.56580300	-4.17760600	1.79172000
C	6.66588400	-2.14557200	0.65191900
H	5.29355400	-0.98518600	-0.52907600
C	6.81839700	-3.21368500	1.53885600
H	5.81224600	-4.77499600	2.63636600
H	7.53422800	-1.58382500	0.32039100
H	7.80484200	-3.47717300	1.90875300
Ni	-1.49285000	-1.21615200	-1.34762200
C	-3.52707300	-1.10583400	-1.94399900
C	-3.47705400	-0.77381500	-0.61092700
H	-3.84055900	-2.09721500	-2.25573300
H	-3.50285700	-0.34883300	-2.72041500
C	-3.84266000	-1.69820800	0.52767700
H	-1.86969900	-2.61032900	-1.29908000
H	-3.36435800	0.27129100	-0.32866400
H	-3.94488000	-2.72280900	0.15362000
H	-3.03799800	-1.70264800	1.27316400
C	-5.13329100	-1.22450800	1.18426400
C	-5.10819500	-0.55733300	2.41514000
C	-6.36625400	-1.43123000	0.54997700
C	-6.29210100	-0.11060500	3.00544700
H	-4.15914200	-0.39871500	2.92307500
C	-7.55013500	-0.98696000	1.13852200
H	-6.40194800	-1.95130800	-0.40485100
C	-7.51537900	-0.32401400	2.36788900
H	-6.25840000	0.39700000	3.96535600

H	-8.49950000	-1.16216900	0.64040600
H	-8.43740300	0.01923900	2.82806100

TS8

C	-1.12128300	2.65145000	-1.25340000
C	0.04553500	2.87759200	-0.53932700
C	0.85171400	1.73583000	-0.23156300
C	0.37919700	0.47025600	-0.64923600
C	-1.53574800	1.35602300	-1.62602700
C	2.09131200	1.79469100	0.48554100
C	1.21150100	-0.69443200	-0.49770500
C	2.49310300	-0.57910100	0.08869000
C	2.87463100	0.68803700	0.63961300
C	3.31606200	-1.74826800	0.11898800
C	2.78509500	-2.91116200	-0.42131500
C	1.48956300	-2.96494900	-0.97224400
H	2.40206500	2.74100200	0.91163800
H	-1.72231400	3.49658800	-1.57279600
H	3.80543900	0.75668500	1.18965500
H	3.39470200	-3.80735000	-0.45336400
N	-0.82797100	0.27702500	-1.26796800
N	0.71286900	-1.87073400	-0.99170000
C	-2.75241600	1.20913400	-2.50284200
H	-2.79451700	0.22288400	-2.96875000
H	-3.68273300	1.38563800	-1.94885600
H	-2.71621000	1.95780900	-3.30135600
C	0.98709000	-4.25389600	-1.56156300
H	0.28757200	-4.74774000	-0.87635300
H	0.45975400	-4.07639400	-2.50250700
H	1.81763600	-4.94021200	-1.74608600
C	0.42337800	4.26424300	-0.17040800
C	1.67708200	4.79943900	-0.51518000
C	-0.50800600	5.08541500	0.48800000
C	1.98949900	6.12200100	-0.20300700
H	2.39412600	4.19099400	-1.05869100
C	-0.18648400	6.40284400	0.81098600
H	-1.47706800	4.68049900	0.76674900
C	1.06204300	6.92438100	0.46533300
H	2.95575900	6.52750900	-0.48828200
H	-0.91095800	7.02146100	1.33237600
H	1.30985900	7.95248100	0.71218000
C	4.69113600	-1.75899300	0.67474600
C	5.06140600	-2.75299800	1.59710200
C	5.65649400	-0.82557300	0.25701500
C	6.35924100	-2.80034000	2.10338900
H	4.32170000	-3.47361600	1.93490700
C	6.95737700	-0.88528100	0.75495000
H	5.39749200	-0.07503200	-0.48419800
C	7.31015800	-1.86790500	1.68273800
H	6.62736900	-3.56498900	2.82641300
H	7.69737700	-0.16783200	0.41265100
H	8.32266400	-1.90897500	2.07326200
Ni	-1.21040900	-1.70525600	-1.25181800
C	-3.14360900	-2.64220500	-1.47448200
C	-3.22865100	-1.53926000	-0.64567600
H	-3.22527700	-3.64923000	-1.07652300
H	-3.31220900	-2.55157100	-2.54569500
C	-3.31214800	-1.62973700	0.86516300
H	-1.32367400	-3.15492500	-1.28016500
H	-3.53620400	-0.59394100	-1.07385100
H	-3.01085000	-2.63194000	1.19239700
H	-2.61079000	-0.91763100	1.31830500
C	-4.72272700	-1.31194300	1.34492500
C	-5.04258800	-0.03601000	1.82505900
C	-5.73049600	-2.28393300	1.28405400
C	-6.34224500	0.26426300	2.23789400
H	-4.26659100	0.72416700	1.89169100
C	-7.02912700	-1.98736000	1.69795200
H	-5.49702400	-3.28212900	0.91984500
C	-7.33867100	-0.71105100	2.17430200
H	-6.57341000	1.25606000	2.61642600

H	-7.79795900	-2.75352700	1.65329400
H	-8.34907800	-0.48086400	2.49947300

CP17

C	0.39952800	3.10820400	0.78228200
C	-0.86919500	2.99910500	0.23282600
C	-1.39036400	1.67900800	0.06000400
C	-0.57199100	0.59206800	0.44694200
C	1.17354000	1.98355700	1.12933700
C	-2.67692100	1.39571400	-0.50560900
C	-1.09746000	-0.74919900	0.40522000
C	-2.41876400	-0.98358500	-0.04328600
C	-3.16822000	0.12437000	-0.55720600
C	-2.90944300	-2.32617700	0.01246200
C	-2.04025900	-3.30190600	0.48239100
C	-0.73024500	-2.99678900	0.89750100
H	-3.25946700	2.21716900	-0.90476800
H	0.80637900	4.09252300	0.98628400
H	-4.13925100	-0.06270400	-0.99952200
H	-2.38707900	-4.32586700	0.56876500
N	0.70975800	0.74168200	0.91618400
N	-0.26962900	-1.73982400	0.85023800
C	2.50636000	2.19504000	1.79247300
H	2.54260200	3.18768300	2.24866600
H	2.67833900	1.44740800	2.56966300
H	3.32870300	2.13460800	1.07180100
C	0.16987900	-4.08446900	1.42032800
H	0.91950600	-4.36832700	0.67056600
H	0.69584300	-3.75932500	2.32352700
H	-0.40398800	-4.98181900	1.66391000
C	-1.62740400	4.22658900	-0.11114900
C	-2.92507800	4.44869000	0.38312200
C	-1.02119400	5.21943600	-0.90004300
C	-3.59830800	5.63367800	0.08914000
H	-3.39303900	3.70898800	1.02628400
C	-1.70357400	6.39636900	-1.20376300
H	-0.02246800	5.05507400	-1.29520900
C	-2.99255600	6.60671800	-0.70904300
H	-4.59409700	5.79958800	0.48961900
H	-1.22851200	7.14844900	-1.82665900
H	-3.52136500	7.52638100	-0.94125000
C	-4.28668800	-2.70296900	-0.38881300
C	-4.48663100	-3.78218900	-1.26698800
C	-5.40874800	-2.03646700	0.13508400
C	-5.77550400	-4.17326200	-1.62587800
H	-3.62791900	-4.29818400	-1.68776700
C	-6.69648200	-2.43966300	-0.21552900
H	-5.27299400	-1.22459000	0.84376400
C	-6.88303200	-3.50424800	-1.10019700
H	-5.91442700	-4.99968800	-2.31658900
H	-7.55444700	-1.92596100	0.20812500
H	-7.88697300	-3.81301600	-1.37605500
Ni	1.58115100	-0.99007000	0.99849300
C	3.41221200	-2.16895500	0.97571500
C	3.45308500	-0.69370600	0.74797000
H	3.76828900	-2.77332000	0.13635000
H	3.86339800	-2.49984200	1.91376700
C	3.83829600	-0.20852700	-0.65050700
H	2.31486100	-2.54303700	1.07813100
H	3.94349700	-0.14134900	1.54854000
H	3.22431800	-0.71761100	-1.40486500
H	3.61261200	0.86124600	-0.73852500
C	5.30942300	-0.44971400	-0.96003100
C	5.70246700	-1.49888400	-1.80055600
C	6.30164400	0.35628700	-0.38365700
C	7.05411100	-1.74181000	-2.05710300
H	4.94556600	-2.12138300	-2.27407300
C	7.65199500	0.11705000	-0.63751400
H	6.01685700	1.18429100	0.26298900
C	8.03230100	-0.93555700	-1.47406000
H	7.34027200	-2.55555800	-2.71768600

H	8.40753700	0.75496200	-0.18732700
H	9.08362100	-1.12065800	-1.67441100

TS9

C	0.60025900	3.41159500	-0.87156300
C	1.70308800	2.74861300	-0.35304200
C	1.60070000	1.33132600	-0.19933600
C	0.39580200	0.70900700	-0.60610900
C	-0.58485400	2.73721500	-1.22199700
C	2.62900000	0.51518900	0.37281000
C	0.27318200	-0.72693500	-0.58086600
C	1.36512600	-1.50886600	-0.13044800
C	2.51305200	-0.84251000	0.40946300
C	1.24949300	-2.93061000	-0.21816200
C	0.07520100	-3.42998200	-0.75533600
C	-0.97668500	-2.59254800	-1.17963200
H	3.50599800	0.99655700	0.78840100
H	0.65402600	4.48152000	-1.03949900
H	3.29823200	-1.44099200	0.85521900
H	-0.03216300	-4.50016700	-0.89691700
N	-0.68241100	1.40745000	-1.07244700
N	-0.90263600	-1.25870700	-1.05310800
C	-1.74065700	3.51708600	-1.78611600
H	-1.41216300	4.50793800	-2.11083000
H	-2.18847200	2.99851500	-2.63796600
H	-2.52253500	3.65367600	-1.02896300
C	-2.16830700	-3.23827800	-1.83310700
H	-2.91251800	-3.56130800	-1.09460900
H	-2.64882500	-2.56343100	-2.53940900
H	-1.85102200	-4.13432000	-2.37439300
C	2.92407400	3.51661300	-0.00605000
C	4.18487500	3.15401100	-0.51235500
C	2.82003700	4.66445600	0.79845100
C	5.31143400	3.92028400	-0.21626400
H	4.27697700	2.29109100	-1.16560100
C	3.95100800	5.42011000	1.10349300
H	1.85308700	4.94924900	1.20409200
C	5.19865500	5.05084900	0.59602700
H	6.27659000	3.63752800	-0.62622200
H	3.85762100	6.29669000	1.73777700
H	6.07842400	5.64322000	0.82944500
C	2.32350200	-3.86649700	0.19793000
C	2.01814600	-4.93682500	1.05596600
C	3.63466200	-3.74371600	-0.29488100
C	3.00367000	-5.85018700	1.42737600
H	1.01029300	-5.03797700	1.44934000
C	4.61426000	-4.66724700	0.06791400
H	3.87872600	-2.94445000	-0.98870600
C	4.30331200	-5.71827100	0.93356900
H	2.75628300	-6.66441600	2.10211400
H	5.61937600	-4.56921400	-0.33165900
H	5.06935500	-6.43327400	1.21871200
Ni	-2.26384000	0.26398500	-1.12934500
C	-4.07056000	-0.71256500	-1.09251900
C	-4.27646800	0.39540700	-0.25832200
H	-3.08115900	1.47533800	-1.07417400
H	-4.91808400	1.18812600	-0.64122200
C	-4.08413300	0.44609000	1.20808400
C	-4.62617200	1.53503100	1.91172300
C	-3.41780500	-0.55915100	1.93323900
C	-4.51506100	1.61765100	3.29874500
H	-5.14772100	2.31769200	1.36556300
C	-3.30564700	-0.47551700	3.31830700
H	-2.98679200	-1.41100300	1.41561600
C	-3.85328500	0.61240100	4.00584900
H	-4.94770000	2.46278700	3.82583000
H	-2.79404600	-1.26201300	3.86550700
H	-3.76631900	0.67255200	5.08669000
H	-3.86387000	-1.66572200	-0.61734600
C	-4.71860100	-0.75514100	-2.46008400
H	-4.10236400	-1.25916600	-3.21103500

H	-5.67186400	-1.29967500	-2.41108900
H	-4.93032700	0.25334800	-2.83066000

CP18

C	-3.04829000	-1.91416900	-0.83049700
C	-3.20803700	-0.62599400	-0.34009600
C	-2.03336200	0.18109500	-0.22987200
C	-0.80388200	-0.38723200	-0.63904600
C	-1.78967500	-2.43330500	-1.19165900
C	-2.02422700	1.51619000	0.29121200
C	0.39063900	0.41881500	-0.66822200
C	0.32613100	1.78335600	-0.29855300
C	-0.89641200	2.28232500	0.25833100
C	1.49856000	2.57909600	-0.49234500
C	2.59900900	1.95200300	-1.05569200
C	2.59366800	0.58131500	-1.38671900
H	-2.93714700	1.91440500	0.71733100
H	-3.92226300	-2.54049600	-0.97116700
H	-0.91436000	3.28957800	0.65668700
H	3.48227200	2.53650800	-1.29186300
N	-0.68509700	-1.68013200	-1.07428200
N	1.52688400	-0.18768500	-1.13474900
C	-1.69538600	-3.83261400	-1.73427300
H	-2.68259600	-4.19166300	-2.03704300
H	-1.02329700	-3.87654700	-2.59512700
H	-1.30302700	-4.51908500	-0.97459700
C	3.78796600	0.00965400	-2.10592400
H	4.63381800	-0.16344700	-1.42882700
H	3.53958000	-0.92465000	-2.61020000
H	4.13342800	0.72354100	-2.86088200
C	-4.56358000	-0.14030200	0.01698200
C	-5.07301600	1.06039000	-0.50885900
C	-5.38425300	-0.91735900	0.85270200
C	-6.36877100	1.47250200	-0.20053300
H	-4.46627200	1.65409400	-1.18646800
C	-6.67423300	-0.49494500	1.16973600
H	-4.99920400	-1.84252300	1.27279100
C	-7.16989300	0.69990900	0.64324100
H	-6.75487000	2.39434200	-0.62560900
H	-7.29181300	-1.09885000	1.82801700
H	-8.17705100	1.02557600	0.88604000
C	1.56337600	4.02645600	-0.16984900
C	2.62792600	4.52002400	0.60346000
C	0.61201200	4.93198800	-0.67199300
C	2.72770500	5.88188700	0.88428700
H	3.36586700	3.82936100	1.00240000
C	0.72289600	6.29515400	-0.40064300
H	-0.19583200	4.57291000	-1.30298900
C	1.77635000	6.77253600	0.38221100
H	3.54851100	6.24713200	1.49449100
H	-0.01057500	6.98529000	-0.80717800
H	1.85781400	7.83407100	0.59636500
Ni	1.18899100	-2.22160800	-1.02823800
C	3.16644300	-2.92778900	-0.72662300
C	2.43784600	-3.34345900	0.37613200
H	0.75070800	-3.59581900	-1.09667900
H	2.07085000	-4.36698400	0.37577500
C	2.26563200	-2.64515500	1.66111600
C	1.45087900	-3.24863200	2.63815700
C	2.90240300	-1.42888600	1.97684900
C	1.27036500	-2.65696700	3.88618500
H	0.96276600	-4.19351800	2.41169000
C	2.72342900	-0.83995400	3.22483100
H	3.55518300	-0.95093400	1.25308000
C	1.90536800	-1.44898500	4.18238900
H	0.64266200	-3.14085800	4.62852800
H	3.23182100	0.09088600	3.45916000
H	1.77356200	-0.98835500	5.15704900
H	3.75347800	-2.02115700	-0.63039000
C	3.57113900	-3.86050500	-1.84188400
H	3.52519300	-3.37524300	-2.82328700

H	4.60996600	-4.18772100	-1.69602100
H	2.93621700	-4.75076600	-1.87440700

TS10

C	-3.25371600	-2.30317600	-0.37261700
C	-3.53834500	-0.99785300	0.00432700
C	-2.44676500	-0.07576600	0.04021700
C	-1.16692300	-0.56318800	-0.31297400
C	-1.95175200	-2.72647900	-0.70579800
C	-2.56350900	1.29968300	0.42812600
C	-0.04936800	0.33863300	-0.39163600
C	-0.23586500	1.72131300	-0.16905400
C	-1.50880900	2.16006300	0.32275000
C	0.87228200	2.58716100	-0.44067100
C	2.02577100	1.99980800	-0.93686800
C	2.14234100	0.60409300	-1.11206800
H	-3.51504900	1.65438000	0.80585800
H	-4.06348800	-3.02018200	-0.45118900
H	-1.62585700	3.19694000	0.61427500
H	2.85990400	2.62765600	-1.23279500
N	-0.92294900	-1.86644000	-0.65835500
N	1.14388400	-0.21467800	-0.76682100
C	-1.72264600	-4.14712200	-1.14316800
H	-2.66915500	-4.62343300	-1.41208300
H	-1.05030300	-4.18653900	-2.00444700
H	-1.26637800	-4.73436100	-0.33744200
C	3.36011700	0.04284500	-1.78683400
H	4.28036900	0.33251200	-1.27205900
H	3.31346800	-1.04470900	-1.85083300
H	3.41356000	0.44090900	-2.80820200
C	-4.93550600	-0.61473500	0.32381700
C	-5.55990900	0.47099300	-0.31537100
C	-5.67563400	-1.37989500	1.24144200
C	-6.89049800	0.78246800	-0.03878200
H	-5.01341400	1.04994700	-1.05427100
C	-7.00129200	-1.05617300	1.52617800
H	-5.20129600	-2.21523300	1.74930500
C	-7.61214400	0.02433700	0.88597600
H	-7.36543200	1.61401600	-0.55116900
H	-7.55663200	-1.64778300	2.24800200
H	-8.64674200	0.27233700	1.10389800
C	0.81812600	4.05891700	-0.25649600
C	1.83604800	4.70381200	0.46679800
C	-0.20025700	4.83662000	-0.83518000
C	1.82569200	6.08885700	0.62411200
H	2.62442600	4.11199000	0.92394000
C	-0.20015200	6.22315600	-0.68679100
H	-0.97351800	4.35946800	-1.43029500
C	0.80812000	6.85176300	0.04727700
H	2.61185500	6.57189900	1.19695500
H	-0.98477100	6.81320100	-1.15132300
H	0.80295100	7.93129200	0.16548600
Ni	1.00934900	-2.21853600	-0.55264600
C	2.58157500	-3.75641900	-0.10058300
C	2.65730600	-2.67974200	0.77960500
H	0.73546900	-3.63322000	-0.61171400
H	2.04380700	-2.76069000	1.68143500
C	3.76581200	-1.71216200	0.89733100
C	3.60711900	-0.57451200	1.71028400
C	5.02917900	-1.95595200	0.32731300
C	4.66361000	0.30882100	1.91830700
H	2.64590000	-0.38866500	2.18362100
C	6.08663800	-1.07185200	0.53499500
H	5.19614000	-2.85593200	-0.25734200
C	5.90677500	0.06669700	1.32533300
H	4.52333000	1.17864400	2.55376400
H	7.05747800	-1.28218900	0.09569000
H	6.73564200	0.74701700	1.49784700
H	3.21860900	-3.72607300	-0.98629100
C	2.16265100	-5.14911300	0.32186700
H	1.73341400	-5.72453700	-0.50265400

H	3.06085500	-5.68084800	0.66566700
H	1.45007700	-5.12639100	1.15166600

CP19

C	2.63516500	-2.72000700	-0.39626300
C	3.16980200	-1.48614800	-0.05172900
C	2.29829900	-0.35311600	-0.11929700
C	0.94358100	-0.58738500	-0.45433200
C	1.27707500	-2.87617500	-0.73354800
C	2.73125700	1.00050900	0.05810200
C	0.02458400	0.51347800	-0.58363100
C	0.48221800	1.83764500	-0.39341700
C	1.86372700	2.04512900	-0.07461200
C	-0.44798200	2.90229800	-0.61594400
C	-1.69532600	2.55156600	-1.10641000
C	-2.08694900	1.20896000	-1.28313900
H	3.77804500	1.19074400	0.26170400
H	3.26852100	-3.60033100	-0.37436400
H	2.22514300	3.06175800	0.02275500
H	-2.41901900	3.32828300	-1.32910200
N	0.44347600	-1.82836500	-0.72894000
N	-1.25919300	0.20497900	-0.96054100
C	0.73842500	-4.23759000	-1.08452500
H	0.06390000	-4.18262100	-1.94469500
H	1.55097000	-4.92599900	-1.32955000
H	0.18452200	-4.67119900	-0.24188900
C	-3.45086900	0.91501700	-1.83668400
H	-3.42982600	0.03292500	-2.48007300
H	-4.16530100	0.73553200	-1.02712100
H	-3.81318000	1.76652400	-2.41877800
C	4.59475200	-1.38604900	0.34700300
C	4.97138300	-0.74629000	1.54148500
C	5.58940600	-1.98257400	-0.44690800
C	6.31014400	-0.70523700	1.92835900
H	4.21138500	-0.30974200	2.18332000
C	6.92847500	-1.92706000	-0.06358900
H	5.31343600	-2.46740800	-1.37928200
C	7.29172500	-1.29028800	1.12517800
H	6.58571800	-0.22105900	2.86064500
H	7.68792200	-2.37970700	-0.69424200
H	8.33463700	-1.25201600	1.42557600
C	-0.12038400	4.32808300	-0.37629800
C	-0.39580700	5.28904900	-1.36443500
C	0.41478500	4.75171100	0.85335100
C	-0.13014500	6.63773300	-1.13326300
H	-0.79443100	4.97344900	-2.32467500
C	0.66583900	6.10322600	1.08570900
H	0.60289400	4.02550100	1.63909200
C	0.39881200	7.04823200	0.09242600
H	-0.33478700	7.36742000	-1.91111700
H	1.06461000	6.41865000	2.04540400
H	0.60036300	8.09987300	0.27352500
Ni	-1.55388600	-1.71467200	-0.77314700
C	-3.43513200	-4.24133300	-1.66432700
C	-2.90261000	-3.41515800	-0.48799200
H	-3.37349200	-3.68157300	-2.60319100
H	-2.87840400	-5.17602400	-1.78534800
C	-3.42883900	-2.02012700	-0.36822800
H	-4.48509800	-4.49477700	-1.48736000
H	-1.74066900	-3.39137800	-0.63822600
H	-2.95458500	-3.96464300	0.45770300
H	-4.11423300	-1.74547100	-1.16988200
C	-3.81643400	-1.48062700	0.95586500
C	-4.97425700	-0.68983900	1.08647100
C	-3.06123700	-1.73746700	2.11902900
C	-5.35577000	-0.16592900	2.32100100
H	-5.59953200	-0.51308800	0.21423000
C	-3.43970100	-1.21134400	3.35216400
H	-2.16936200	-2.35778100	2.05971400
C	-4.58708700	-0.42000400	3.45866400
H	-6.26033400	0.43088100	2.39638900

H	-2.84217900	-1.42397200	4.23425900
H	-4.88436200	-0.01558500	4.42165500

CP20

B	0.64166100	1.24987400	0.14667000
O	0.72004400	0.38459100	-1.09196500
H	0.16805700	0.73876500	-1.80021900
O	0.11780600	0.28152600	1.17561000
H	0.54953600	0.47178700	2.01918600
O	1.97543200	1.63882200	0.61799800
H	2.26995600	2.41619100	0.12437200
C	-0.36161500	2.51806700	-0.09303600
C	-0.10895900	3.77298200	0.48883000
C	-1.54089100	2.41356800	-0.85559900
C	-0.96669500	4.86258200	0.31582700
H	0.78363400	3.89510000	1.10040000
C	-2.41003600	3.49162300	-1.04053700
H	-1.79735900	1.45790800	-1.31526000
C	-2.12351300	4.72657800	-0.45414600
H	-0.73372100	5.81833100	0.78147200
H	-3.30971600	3.37059200	-1.64120800
H	-2.79424900	5.57113300	-0.59447700
Li	0.34493400	-1.21799100	-0.03283700
C	3.01155000	-1.55746700	0.53422000
O	1.97610800	-2.22090700	0.32659000
C	-2.36573100	-2.71246500	-0.27446900
O	-1.21811100	-2.25402400	-0.36897000
N	-3.34134200	-2.08483200	0.43077300
N	3.83550000	-1.20154300	-0.48229900
C	-2.69498200	-4.02553200	-0.97100700
H	-3.05908900	-4.78253800	-0.26814300
H	-3.45427900	-3.89308400	-1.74997400
H	-1.77616200	-4.38630300	-1.43372300
C	3.37599300	-1.13080800	1.94243900
H	4.41305900	-1.36703500	2.20263900
H	3.22646700	-0.04888400	2.03257100
H	2.70751200	-1.64842800	2.63206000
C	4.84133100	-0.15388500	-0.33250100
H	5.57791400	-0.25250000	-1.13481800
H	4.37617800	0.83793300	-0.38191700
H	5.36551200	-0.24862600	0.61966600
C	3.40964100	-1.50116000	-1.84980700
H	2.90509500	-2.46739900	-1.86130100
H	2.71138100	-0.73687200	-2.20922200
H	4.29290500	-1.53986300	-2.49424000
C	-4.71899300	-2.54737600	0.52013000
H	-4.86636900	-3.47688000	-0.02638700
H	-4.99509000	-2.71261900	1.56945500
H	-5.39876600	-1.79062600	0.10698500
C	-3.07645600	-0.81937100	1.11685200
H	-3.43597700	-0.89057400	2.15052300
H	-2.01114400	-0.59061900	1.11955200
H	-3.61159000	0.00025900	0.62058200

CP21

C	1.47802300	2.89523900	0.59532300
C	2.46338000	2.72002600	-0.35929500
C	2.68112600	1.39967300	-0.85096900
C	1.90457800	0.34110000	-0.30280300
C	0.73114400	1.80771000	1.08529000
C	3.61250900	1.11396800	-1.90222200
C	2.13691100	-1.02172300	-0.76877400
C	3.12364700	-1.26461500	-1.76569400
C	3.82391900	-0.15697100	-2.34240900
C	3.33095900	-2.61860300	-2.17331200
C	2.53987100	-3.59694400	-1.59600200
C	1.57996400	-3.26465900	-0.61312400
H	4.14101200	1.94042700	-2.36309900
H	1.28288300	3.88510900	0.99333900
H	4.51897500	-0.33941400	-3.15411000
H	2.67829600	-4.63756700	-1.87560000
N	0.95467900	0.56306900	0.64917400

N	1.39656700	-2.01077000	-0.22147500
C	-0.32677200	2.02696800	2.13223400
H	-0.38639900	3.08584200	2.40319500
H	-1.30545700	1.70171700	1.76492500
H	-0.10406000	1.44100900	3.02953300
C	0.75896200	-4.34061000	0.05114900
H	-0.11391100	-3.90855800	0.54680700
H	0.43650000	-5.09675400	-0.67426100
H	1.35573600	-4.85762800	0.81487900
C	3.24421300	3.89832000	-0.82547800
C	2.57523800	5.02519500	-1.33029400
C	4.64482100	3.93354200	-0.72119200
C	3.29106500	6.15452700	-1.72905400
H	1.49333800	4.99877400	-1.42735400
C	5.35721500	5.06670000	-1.11401900
H	5.17225600	3.07697600	-0.31082600
C	4.68297600	6.17963600	-1.62142100
H	2.75971600	7.01525600	-2.12666600
H	6.43956800	5.08158600	-1.01745800
H	5.23918700	7.06088900	-1.92959200
C	4.35888500	-3.00919600	-3.17787700
C	5.71356600	-2.67995400	-3.00255500
C	3.99318100	-3.76966200	-4.30031200
C	6.67235300	-3.09730100	-3.92578600
H	6.01634500	-2.11208900	-2.12733700
C	4.95187300	-4.18158300	-5.22633100
H	2.94751900	-4.02406900	-4.45067700
C	6.29474000	-3.84697800	-5.04197100
H	7.71678000	-2.84125600	-3.76881000
H	4.64824800	-4.76246300	-6.09318500
H	7.04218000	-4.16937200	-5.76177300
C	0.71867700	-1.25124500	2.89599700
C	2.15038500	-0.75183700	3.06468900
H	0.69793600	-2.34721500	2.94436800
H	0.07669000	-0.88124800	3.70805900
C	2.80886600	-1.25289700	4.37771800
H	2.77559200	-1.08212600	2.22390300
H	2.19588900	0.34612000	3.06495700
H	2.19754900	-0.92081000	5.22746500
H	2.78415700	-2.35066300	4.38599400
C	4.23234500	-0.77110900	4.55146300
C	4.51116100	0.44604900	5.19071100
C	5.31168300	-1.50768100	4.04117000
C	5.82041700	0.91389500	5.31511100
H	3.68930300	1.02981400	5.60083000
C	6.62334700	-1.04584100	4.16212600
H	5.11853500	-2.45875800	3.54862400
C	6.88354000	0.16883800	4.80032100
H	6.01098200	1.85813300	5.81983500
H	7.44355300	-1.63817600	3.76320800
H	7.90453400	0.52844600	4.90022900
B	-2.49370900	0.36001300	-0.70809500
O	-3.43685700	-0.29512200	-1.69371600
H	-3.35404800	0.09817500	-2.57222000
O	-3.29553100	0.47662400	0.53812500
H	-3.01574800	-0.22750900	1.15651800
O	-1.32978000	-0.56715400	-0.52329600
H	-1.68187100	-1.46151900	-0.66371700
C	-2.00206200	1.80129700	-1.27819500
C	-1.16187600	1.87879700	-2.40535700
C	-2.43720200	3.02242500	-0.73361700
C	-0.78843800	3.10087800	-2.97146800
H	-0.78248400	0.95657100	-2.84277000
C	-2.06718400	4.25415400	-1.28327900
H	-3.07399200	2.99815800	0.14744400
C	-1.24528800	4.29809800	-2.41319400
H	-0.13997000	3.12151900	-3.84503500
H	-2.41489800	5.18194500	-0.83080700
H	-0.96575600	5.25269100	-2.85426300
Ni	-0.25208100	-0.89162300	1.24301600

Br	-2.07148600	-2.31831400	1.99684100
Li	-4.91355400	-0.12200400	-0.47162700
C	-6.22745500	-2.86441900	-0.19077300
O	-6.04491100	-1.63736100	-0.16699300
C	-6.10770900	2.33035300	0.27037200
O	-6.11313200	1.42453400	-0.58120100
N	-6.15736500	3.63642900	-0.10554900
N	-7.42521900	-3.37513900	-0.59594300
C	-8.50188300	-2.47790500	-0.99889800
H	-9.38075300	-2.63096000	-0.35934900
H	-8.15739300	-1.44948100	-0.90759100
H	-8.79131100	-2.67731200	-2.03853600
C	-7.74514300	-4.79260800	-0.66076000
H	-8.06774800	-5.05887000	-1.67568600
H	-6.88318900	-5.40461900	-0.40339800
H	-8.56445500	-5.03449900	0.02970600
C	-6.16885200	3.96796500	-1.52847800
H	-6.52671600	3.10752000	-2.09081100
H	-6.83284800	4.82383100	-1.69257400
H	-5.15884900	4.22335700	-1.87296700
C	-5.98024600	4.76993000	0.78998600
H	-5.06470500	5.31683700	0.52886300
H	-6.82774300	5.46187600	0.70220200
H	-5.89873700	4.44843700	1.82638200
C	-6.04294700	1.98173500	1.74652600
H	-6.33974100	0.93677700	1.84981900
H	-5.00897600	2.06718500	2.09839400
H	-6.69155900	2.59977200	2.37313200
C	-5.12939000	-3.82136700	0.22913700
H	-4.27981100	-3.24327000	0.59803300
H	-5.46032900	-4.49684100	1.02534300
H	-4.79783900	-4.43446200	-0.61733000

TS11

C	1.64115000	3.21051600	0.41214300
C	2.66607500	2.85800300	-0.44407500
C	2.81906000	1.47608300	-0.75985300
C	1.91073900	0.54979400	-0.17872800
C	0.77712300	2.24242200	0.96195000
C	3.81529700	1.00446400	-1.67528700
C	2.04853200	-0.86671400	-0.49748100
C	3.09969100	-1.29708700	-1.35678900
C	3.95503300	-0.31969300	-1.95917900
C	3.20950400	-2.69774100	-1.61564800
C	2.26698800	-3.53844700	-1.04635500
C	1.24910300	-3.02299200	-0.21442200
H	4.45666700	1.73180300	-2.15948400
H	1.49570300	4.25161000	0.67964400
H	4.70691200	-0.64399800	-2.66954300
H	2.32824800	-4.60988700	-1.21474400
N	0.91421200	0.94559100	0.66416200
N	1.15608200	-1.72465700	0.04388200
C	-0.33391300	2.66832500	1.87933300
H	-0.18478900	3.69996100	2.21371600
H	-1.29467900	2.60026600	1.35305100
H	-0.38994400	2.00981400	2.75026300
C	0.25266100	-3.94136500	0.44702700
H	-0.68575500	-3.41444400	0.64090200
H	0.06463400	-4.83111100	-0.16500700
H	0.63813300	-4.28627500	1.41590300
C	3.54688500	3.91962500	-1.00513300
C	2.98433300	4.96141900	-1.76022200
C	4.92907300	3.93031200	-0.75648200
C	3.78973600	5.98585400	-2.25955700
H	1.91842700	4.94970500	-1.97233300
C	5.72995700	4.95981600	-1.25148100
H	5.37133600	3.13920300	-0.15698300
C	5.16280600	5.98928300	-2.00591100
H	3.34248000	6.78123700	-2.84978700
H	6.79642900	4.96001700	-1.04233700
H	5.78775500	6.78960500	-2.39307400

C	4.28917300	-3.27843300	-2.46097300
C	5.64527100	-3.04979400	-2.17242400
C	3.96521600	-4.11786300	-3.53907700
C	6.64603300	-3.64142600	-2.94321900
H	5.91310700	-2.42137000	-1.32783900
C	4.96662300	-4.70434100	-4.31325900
H	2.92008500	-4.29541900	-3.77769600
C	6.31051800	-4.46812400	-4.01768100
H	7.68951800	-3.46034100	-2.69972700
H	4.69599900	-5.34352000	-5.14944800
H	7.09112600	-4.92567800	-4.61928700
C	0.05319200	-0.71102500	2.93232000
C	1.49167900	-0.38598800	3.32075300
H	-0.12469100	-1.79072900	3.02302300
H	-0.66002500	-0.21145800	3.60399400
C	1.86402700	-0.89506800	4.73872800
H	2.19242700	-0.83433400	2.60326800
H	1.67720700	0.69650600	3.29155000
H	1.17401700	-0.44896900	5.46725700
H	1.69881900	-1.98025500	4.77638700
C	3.29187800	-0.58177400	5.12827800
C	3.61278000	0.59869800	5.81427200
C	4.34122200	-1.44458700	4.77783100
C	4.93457100	0.90961800	6.13855500
H	2.81325100	1.27868400	6.10194900
C	5.66491100	-1.13974800	5.09865000
H	4.11360000	-2.37015300	4.25233100
C	5.96748700	0.04049300	5.78139500
H	5.15705100	1.82871600	6.67547100
H	6.46024000	-1.82753200	4.82098400
H	6.99708500	0.27790100	6.03676100
B	-2.30761400	1.05895400	-1.10711500
O	-3.31457500	0.22934900	-1.89656700
H	-3.65331300	0.74577500	-2.64055400
O	-2.93328200	1.54124300	0.13036900
H	-2.83925600	0.85922700	0.82041100
O	-1.21887100	0.05605000	-0.78982900
H	-1.54455900	-0.80112400	-1.10217500
C	-1.74837400	2.29577700	-1.99628600
C	-0.86748000	2.07503100	-3.07159500
C	-2.12600200	3.62817400	-1.75150300
C	-0.38934100	3.12093300	-3.86431000
H	-0.53975000	1.05842000	-3.28087900
C	-1.65728500	4.68610100	-2.53644600
H	-2.79455700	3.83581700	-0.91943700
C	-0.78543500	4.43560900	-3.59933700
H	0.29289100	2.91462600	-4.68685500
H	-1.97120900	5.70586100	-2.32051000
H	-0.42243900	5.25390800	-4.21793600
Ni	-0.54999800	-0.33418600	1.12143600
Br	-2.68379400	-1.48458100	1.56126100
Li	-4.59489100	-0.33082500	-0.55864600
C	-4.79474000	-3.27105600	-0.75629000
O	-5.19536800	-2.12344100	-1.00896400
C	-6.72031300	1.55878200	0.49579200
O	-6.21751300	0.57482000	-0.07043200
N	-8.06964300	1.63027300	0.67785500
N	-3.95173000	-3.91956000	-1.60205500
C	-5.84990100	2.69650300	0.99390200
H	-4.81462000	2.46693200	0.72814000
H	-6.13813100	3.65004500	0.53669100
H	-5.92838100	2.80822300	2.08177100
C	-8.76188800	2.73035400	1.33016000
H	-8.06219200	3.49243700	1.66709600
H	-9.47718000	3.19511400	0.63843200
H	-9.32179800	2.36361900	2.20064600
C	-8.92718100	0.54751200	0.21020300
H	-8.30730300	-0.21673000	-0.25490000
H	-9.47888600	0.11158100	1.05290700
H	-9.65338500	0.92795100	-0.51960400

C	-5.24433900	-3.97087500	0.51387000
H	-5.94499900	-3.30655600	1.01960200
H	-5.73851000	-4.92742500	0.31178400
H	-4.39186800	-4.14195000	1.17910600
C	-3.50088900	-5.29171300	-1.43047600
H	-2.40530000	-5.33251900	-1.46554100
H	-3.82373300	-5.69519200	-0.47276400
H	-3.89256100	-5.93380200	-2.23172500
C	-3.49738700	-3.24727600	-2.81736200
H	-4.09849700	-3.55349100	-3.68470800
H	-3.57026000	-2.16745700	-2.68870700
H	-2.45383600	-3.52151900	-3.00575300

CP22

C	1.79071400	2.82285100	1.33645100
C	2.76338000	2.71935500	0.36131100
C	2.82641900	1.50633700	-0.38514200
C	1.88339500	0.48466200	-0.08912100
C	0.88727300	1.77093300	1.58783500
C	3.76749600	1.30926500	-1.44747800
C	1.93424300	-0.76227700	-0.84468700
C	2.93743100	-0.94175500	-1.83999100
C	3.82594300	0.13951100	-2.14124700
C	2.96856600	-2.19259600	-2.52958000
C	1.99905900	-3.13040200	-2.21463500
C	1.03173400	-2.86260000	-1.22154300
H	4.43348800	2.12457100	-1.70550500
H	1.71642000	3.72700200	1.93106800
H	4.53710800	0.02598700	-2.95134800
H	1.99971400	-4.09545400	-2.71325900
N	0.93465500	0.63672100	0.87962500
N	1.01314800	-1.71022700	-0.56348200
C	-0.16416800	1.92912800	2.64902900
H	0.05659400	2.79284900	3.28428100
H	-1.14407000	2.07154800	2.17495800
H	-0.22641800	1.03089700	3.26947000
C	0.00851400	-3.90337300	-0.84282400
H	-0.89462000	-3.42775600	-0.45072000
H	-0.24442000	-4.54104900	-1.69757600
H	0.40383100	-4.55545900	-0.05227700
C	3.68403100	3.86542800	0.12330200
C	3.15745300	5.11476300	-0.24241700
C	5.07127400	3.73848900	0.30086500
C	4.00242800	6.20920900	-0.43148000
H	2.08689800	5.21583200	-0.40052900
C	5.91225600	4.83643400	0.11757600
H	5.48695500	2.78073100	0.60204700
C	5.38038500	6.07423300	-0.25065100
H	3.58213500	7.16804100	-0.72302100
H	6.98280300	4.72540200	0.26816300
H	6.03652200	6.92831800	-0.39543500
C	3.99555300	-2.52184700	-3.55636600
C	5.36846700	-2.44466200	-3.26697600
C	3.60209200	-2.97395300	-4.82638500
C	6.31827700	-2.80575500	-4.22251500
H	5.68932500	-2.11922800	-2.28152200
C	4.55282000	-3.32934100	-5.78344200
H	2.54352900	-3.02974300	-5.06534100
C	5.91406800	-3.24646000	-5.48456400
H	7.37548800	-2.74814100	-3.97778500
H	4.22915900	-3.66818900	-6.76395400
H	6.65495800	-3.52453100	-6.22914100
C	-0.00616200	-1.60459900	2.52364200
C	1.44635600	-1.49316400	2.97457400
H	-0.23856900	-2.64584700	2.26294600
H	-0.69362900	-1.31378200	3.33195800
C	1.78269900	-2.43128700	4.16385500
H	2.12414200	-1.73594900	2.14484300
H	1.69134700	-0.46558600	3.27586400
H	1.11049500	-2.19579200	4.99972100
H	1.56284300	-3.46605000	3.86865200

C	3.22156100	-2.32415100	4.61891600
C	3.60031800	-1.40590200	5.60924400
C	4.22433400	-3.11122200	4.03338900
C	4.93373400	-1.27507800	6.00100700
H	2.83699000	-0.79069800	6.08173800
C	5.55948900	-2.98542100	4.42033500
H	3.95084600	-3.83563200	3.26852400
C	5.92012400	-2.06526900	5.40706700
H	5.20164600	-0.55939200	6.77464000
H	6.31802800	-3.61072700	3.95532200
H	6.95852600	-1.96846100	5.71358000
B	-2.31842400	1.47857000	-0.60889600
O	-3.48143500	0.99863100	-1.46730000
H	-3.29324200	1.10743100	-2.40986000
O	-2.80419800	1.61284400	0.75399000
H	-2.87000100	0.72517400	1.14814600
O	-1.27285000	0.36599700	-0.72433600
H	-1.71929500	-0.36656000	-1.17598600
C	-1.70903600	2.87894400	-1.15591200
C	-0.87512100	2.93187100	-2.28736200
C	-2.00604500	4.10517800	-0.53368500
C	-0.37449900	4.13840300	-2.78461300
H	-0.59314100	2.00152800	-2.77808600
C	-1.51078000	5.32013900	-1.01582800
H	-2.62551300	4.09572300	0.36019100
C	-0.69358500	5.34184800	-2.14975900
H	0.26935300	4.14133300	-3.66209500
H	-1.75870800	6.25071700	-0.50801800
H	-0.31034600	6.28471700	-2.53428700
Ni	-0.59485800	-0.63323100	0.94508100
Br	-2.79426300	-1.79175200	1.00706200
Li	-4.60158200	-0.31313000	-0.63903600
C	-5.06126100	-3.03240000	-1.52511500
O	-5.20760500	-1.80574700	-1.68021100
C	-5.99937200	1.66890300	0.80878400
O	-6.03202500	0.50472200	0.35974400
N	-6.27607800	2.72915200	0.01260000
N	-5.63150100	-3.69806500	-0.49230200
C	-6.38240700	-2.96049900	0.52321900
H	-5.75297600	-2.77486200	1.40145700
H	-6.70767800	-2.00369900	0.11950600
H	-7.25367800	-3.55485500	0.81997100
C	-5.30118100	-5.07055600	-0.12971300
H	-6.21391900	-5.59836300	0.16874500
H	-4.85388800	-5.60569200	-0.96575400
H	-4.59817700	-5.08458800	0.71357600
C	-6.54856400	2.49178600	-1.40395800
H	-7.07363000	1.54307700	-1.51589200
H	-7.17312700	3.30645400	-1.78324600
H	-5.61297200	2.44619100	-1.97215300
C	-5.99107900	4.11374900	0.36810500
H	-5.08643100	4.46045500	-0.14735900
H	-6.83183400	4.75223300	0.07232300
H	-5.83713200	4.21930600	1.44042200
C	-5.66793400	1.90578800	2.26819600
H	-5.71252700	0.94187600	2.77704600
H	-4.64431100	2.28782400	2.33933200
H	-6.35311400	2.60551400	2.75676100
C	-4.23326800	-3.81331800	-2.53126600
H	-3.28936400	-4.13654300	-2.07741100
H	-4.75255400	-4.69739000	-2.91476200
H	-4.00896000	-3.14296700	-3.36164400

TS12

C	2.21420900	3.10275100	0.87587800
C	3.10407300	2.80037700	-0.14045200
C	3.04581700	1.49287100	-0.70895500
C	2.07281300	0.59403500	-0.19472900
C	1.27763200	2.15249100	1.33324600
C	3.88564200	1.06707300	-1.78904300
C	2.00765100	-0.76734600	-0.71441700

C	2.93552500	-1.17672700	-1.71169800
C	3.83631400	-0.20817200	-2.26482500
C	2.89119700	-2.53649100	-2.14160500
C	1.93529900	-3.36155600	-1.57172100
C	1.03678500	-2.87849200	-0.59827400
H	4.56452500	1.78426600	-2.23628400
H	2.24157100	4.08267400	1.34119700
H	4.47627000	-0.50224000	-3.08885500
H	1.89067100	-4.40857200	-1.85457300
N	1.20846800	0.93885700	0.78854800
N	1.06981700	-1.60514700	-0.20113200
C	0.35408600	2.46756400	2.47804200
H	0.36998200	3.53589500	2.71248900
H	-0.66665100	2.16012000	2.23375900
H	0.66657800	1.91474900	3.37316800
C	0.04517100	-3.80524600	0.04894600
H	-0.95507100	-3.36103800	0.07650000
H	0.00465500	-4.76522100	-0.47506400
H	0.33476100	-3.99730600	1.08955100
C	4.07369900	3.83545200	-0.59318400
C	3.60408400	5.08672300	-1.02492000
C	5.45962800	3.61001300	-0.55356900
C	4.49952500	6.08324100	-1.41462800
H	2.53269000	5.26275000	-1.07156100
C	6.35275800	4.61059700	-0.93752200
H	5.83646100	2.65394600	-0.20067600
C	5.87543800	5.84935900	-1.37131000
H	4.12050300	7.04336300	-1.75475900
H	7.42237100	4.42361000	-0.89174700
H	6.57183700	6.62734100	-1.67244800
C	3.83068200	-3.10004400	-3.14938600
C	5.22300200	-3.01504100	-2.97946000
C	3.33135300	-3.78572500	-4.26875000
C	6.08897700	-3.59690900	-3.90529400
H	5.62484600	-2.50940900	-2.10612900

C	4.19846100	-4.36127300	-5.19760700
H	2.25667900	-3.85140600	-4.41531800
C	5.58002300	-4.26918000	-5.01856500
H	7.16276500	-3.52984400	-3.75255200
H	3.79370700	-4.87989600	-6.06253100
H	6.25572200	-4.71949500	-5.74054400
C	-0.18906100	-1.15467900	2.61082600
C	1.22571100	-1.37379800	3.12660300
H	-0.77267300	-2.08572400	2.63560400
H	-0.72162700	-0.40405700	3.21802800
C	1.27189900	-1.87932700	4.59302900
H	1.75346100	-2.10158000	2.49518800
H	1.80651000	-0.44453800	3.06207500
H	0.75057100	-1.15363600	5.23167900
H	0.70509600	-2.81727500	4.66124100
C	2.67914300	-2.09254600	5.10603400
C	3.39514900	-1.04520700	5.70445400
C	3.32143100	-3.33117800	4.96257400
C	4.70764700	-1.22587900	6.14339700
H	2.91269100	-0.07808800	5.83231900
C	4.63385100	-3.51877700	5.39953400
H	2.78122800	-4.15976800	4.50859300
C	5.33316800	-2.46536900	5.99225400
H	5.24025600	-0.40000600	6.60890400
H	5.10865300	-4.49002300	5.28231600
H	6.35375400	-2.61020200	6.33719200
B	-2.07300800	1.37658200	-0.49712100
O	-3.38291200	1.15102300	-1.20502300
H	-3.30871900	1.40774300	-2.13499200
O	-2.30577500	1.26172100	0.94933000
H	-2.80132900	0.43854400	1.12351200
O	-1.13562900	0.25077900	-0.93595300
H	-1.71787600	-0.48591600	-1.19427400
C	-1.44234500	2.81451800	-0.89406300
C	-0.67255500	2.98666800	-2.05818900

C	-1.71920400	3.97174100	-0.14228500
C	-0.21376200	4.24434300	-2.46286400
H	-0.41976900	2.11147200	-2.65337300
C	-1.26189400	5.23406600	-0.52890900
H	-2.29795700	3.87000500	0.77287800
C	-0.50960100	5.37635900	-1.69889400
H	0.37499800	4.34218700	-3.37294400
H	-1.49154400	6.10822300	0.07796300
H	-0.16262300	6.35852500	-2.01335800
Ni	-0.46752300	-0.49618100	0.83713500
Br	-3.17674200	-1.92262700	0.58705300
Li	-4.68857200	-0.16582700	-0.69453300
C	-5.28883500	-2.46416000	-2.12650400
O	-5.45267400	-1.22762200	-2.11905300
C	-5.76866700	1.77211600	1.07712400
O	-5.98325200	0.73048700	0.42754500
N	-5.96182200	2.99429700	0.52221400
N	-6.01204100	-3.27813000	-1.32053400
C	-6.93352000	-2.67533600	-0.35744500
H	-6.41933300	-2.45830700	0.58671000
H	-7.32466700	-1.74371600	-0.76375400
H	-7.75525600	-3.37463100	-0.17303000
C	-5.64234500	-4.66090700	-1.04005100
H	-6.54989700	-5.25443600	-0.88716900
H	-5.08520900	-5.09580700	-1.86878700
H	-5.02415800	-4.71256100	-0.13424200
C	-6.35454000	3.08450400	-0.88112500
H	-6.87233200	2.17022400	-1.16737700
H	-7.01889900	3.94522700	-1.01225600
H	-5.47187900	3.20867200	-1.51987500
C	-5.54689900	4.25154300	1.12968400
H	-4.68030900	4.66259600	0.59629200
H	-6.36621200	4.97905100	1.07749700
H	-5.27302800	4.11209400	2.17365500
C	-5.28800300	1.67906100	2.51257500
H	-5.38338300	0.63778300	2.82267700
H	-4.22881900	1.95607400	2.55939500
H	-5.85662700	2.31528500	3.19796700
C	-4.26751400	-3.07822000	-3.06517900
H	-3.38553300	-3.38258200	-2.48994300
H	-4.64919300	-3.94681700	-3.61041100
H	-3.96947700	-2.30826700	-3.77804200

CP23

Li	-0.00000100	0.91979400	0.55426800
C	2.65887000	0.98970100	0.27828400
O	1.74307100	1.46924800	0.98540600
N	3.25309400	-0.17925300	0.60173300
C	-2.65882200	0.98973100	0.27820700
O	-1.74307000	1.46925900	0.98541100
N	-3.25312800	-0.17917500	0.60168100
Br	-0.00001600	-0.90465900	-1.00992100
C	-4.06486500	-0.94669700	-0.33832600
H	-4.83897600	-1.48984000	0.21313000
H	-4.55315100	-0.29326400	-1.05983400
H	-3.43633800	-1.66517200	-0.87965800
C	-2.71513200	-0.94430400	1.72674500
H	-1.86238200	-1.54955500	1.39372800
H	-2.38762600	-0.25750400	2.50697800
H	-3.50214200	-1.59578500	2.11745300
C	2.71495500	-0.94448000	1.72665900
H	2.38713600	-0.25772700	2.50680400
H	1.86238300	-1.54985400	1.39341500
H	3.50197500	-1.59581900	2.11758100
C	4.06486200	-0.94668600	-0.33831600
H	4.55376100	-0.29312200	-1.05929600
H	4.83849500	-1.49043700	0.21320300
H	3.43624700	-1.66463900	-0.88024400
C	-3.11656700	1.73789400	-0.95724900
H	-2.72195800	1.23681300	-1.84886900
H	-4.20530700	1.80335100	-1.04314200

H	-2.69855100	2.74438600	-0.90957300
C	3.11679100	1.73788500	-0.95710000
H	4.20554900	1.80350900	-1.04269800
H	2.72252900	1.23671300	-1.84882200
H	2.69860200	2.74431200	-0.90957000

CP24

C	-1.54541900	2.84659200	1.03457000
C	-0.37267600	3.24464000	0.41639000
C	0.38903300	2.24957400	-0.27153000
C	-0.09570800	0.91271300	-0.23680100
C	-1.96282200	1.49558000	1.00271300
C	1.57146700	2.54511500	-1.02221000
C	0.65901700	-0.14344900	-0.89998900
C	1.86660300	0.18790300	-1.57747700
C	2.27528200	1.56016200	-1.64574900
C	2.60055600	-0.86150400	-2.20424500
C	2.06144100	-2.13634100	-2.16010500
C	0.85755600	-2.39940700	-1.48310700
H	1.89430300	3.57640900	-1.10901400
H	-2.14362700	3.57464100	1.57467300
H	3.15488000	1.81000300	-2.22722200
H	2.59216000	-2.96170000	-2.62279100
N	-1.24187000	0.56657500	0.38762900
N	0.18546400	-1.42515000	-0.85476000
C	-3.24951800	1.07408500	1.66041400
H	-4.10157900	1.29861000	1.00656800
H	-3.25350400	-0.00269800	1.83725700
H	-3.40323000	1.60774000	2.60521600
C	0.31655800	-3.80397400	-1.43224500
H	-0.70870600	-3.84639100	-1.81389400
H	0.93988100	-4.48134800	-2.02395700
H	0.28359100	-4.16550600	-0.39934800
C	0.04887500	4.67055600	0.49296500
C	-0.83729600	5.68687700	0.10051600
C	1.30622500	5.03484900	1.00355300
C	-0.47344200	7.02941600	0.20595100
H	-1.80969000	5.41748700	-0.30260000
C	1.66641400	6.37792500	1.11444700
H	1.99346300	4.26152900	1.33522900
C	0.77936000	7.37922100	0.71334500
H	-1.16917600	7.80180300	-0.11050200
H	2.63903500	6.64179300	1.52097900
H	1.06203600	8.42493700	0.79772300
C	3.90371000	-0.64580300	-2.88875900
C	4.98917100	-0.05593900	-2.21889500
C	4.08609700	-1.08840600	-4.20901900
C	6.22131700	0.08944700	-2.85596700
H	4.87095100	0.26690400	-1.18852900
C	5.31686200	-0.93457300	-4.84722900
H	3.25176100	-1.53836400	-4.74020300
C	6.38806600	-0.34588400	-4.17246800
H	7.05340500	0.53781900	-2.32008000
H	5.43751800	-1.27329900	-5.87256400
H	7.34759500	-0.22886400	-4.66850100
C	-0.75893900	-2.46668700	1.70040100
C	0.65772400	-2.07386200	2.10238300
H	-0.86665600	-3.56478000	1.69727400
H	-1.48319300	-2.07263500	2.42691300
C	1.04233100	-2.54374800	3.52983100
H	1.39614100	-2.48641200	1.40051700
H	0.77475300	-0.98240400	2.05917700
H	0.31731600	-2.13025900	4.24307100
H	0.94220500	-3.63632100	3.58091400
C	2.44373800	-2.13831500	3.93121800
C	2.68365800	-0.91987400	4.58319300
C	3.54605000	-2.95007600	3.62523400
C	3.97953800	-0.52373100	4.91855400
H	1.84140200	-0.27879100	4.83604100
C	4.84440200	-2.55954100	3.95710900
H	3.38118200	-3.90362600	3.12717500

C	5.06644500	-1.34291700	4.60588700
H	4.13982300	0.42225700	5.43026300
H	5.68246700	-3.20887500	3.71542100
H	6.07594600	-1.03924300	4.87088500
B	-3.94170500	-2.09091200	-0.85423600
O	-4.28542300	-3.37456900	-1.42440200
H	-5.21099100	-3.37761300	-1.69931700
O	-3.31351800	-2.35335900	0.53497300
H	-3.35294200	-3.31221300	0.68036900
O	-2.73698200	-1.52249500	-1.59231500
H	-2.55900600	-2.14664400	-2.31230000
C	-5.14963900	-1.02909900	-0.75444900
C	-5.21271600	0.11448900	-1.56919800
C	-6.22401500	-1.24181700	0.12971900
C	-6.28895000	1.00498700	-1.50576900
H	-4.39419300	0.30866500	-2.25820600
C	-7.30599700	-0.36180700	0.20227300
H	-6.20804100	-2.11112000	0.78578700
C	-7.34132500	0.76878500	-0.61823900
H	-6.30821900	1.88244000	-2.14937800
H	-8.11907700	-0.55324600	0.89956400
H	-8.18082700	1.45841100	-0.56570100
Ni	-1.51795000	-1.84850400	0.02160200

TS13

C	-0.56657900	-2.88020200	-1.78615400
C	-1.79953300	-2.25339700	-1.74672600
C	-1.82789400	-0.87974500	-1.36658300
C	-0.60055500	-0.25435900	-1.00391800
C	0.60878900	-2.20395100	-1.41128700
C	-3.03482400	-0.10700000	-1.37653700
C	-0.61101900	1.15167900	-0.61304900
C	-1.85080800	1.85138200	-0.55219400
C	-3.04894500	1.19476300	-0.98015200
C	-1.82212800	3.20988000	-0.11531600
C	-0.58791500	3.76564900	0.17881800
C	0.59741500	3.00852900	0.05798300
H	-3.94771700	-0.57708700	-1.72282900
H	-0.50236900	-3.92546400	-2.06988200
H	-3.97394700	1.75914200	-1.01011600
H	-0.52935300	4.79160600	0.52909800
N	0.58517300	-0.92828000	-1.00555600
N	0.57414900	1.73643200	-0.33235400
C	1.92955900	-2.92297200	-1.45136100
H	1.78941900	-3.98987200	-1.65164400
H	2.56142200	-2.49978600	-2.24041200
H	2.46454000	-2.79726800	-0.50527800
C	1.93300700	3.61195300	0.39815200
H	2.31796700	3.16347800	1.32244200
H	2.65857800	3.39827700	-0.39314100
H	1.85496900	4.69322400	0.54884500
C	-3.02290600	-3.02644200	-2.09379500
C	-3.09807200	-3.71007000	-3.31803300
C	-4.09437800	-3.13831800	-1.19156900
C	-4.21958700	-4.47546500	-3.63797100
H	-2.27843200	-3.62513500	-4.02641800
C	-5.21127300	-3.91067100	-1.50989200
H	-4.03910000	-2.63675900	-0.22958200
C	-5.27919400	-4.57842600	-2.73469000
H	-4.26520800	-4.98988100	-4.59398700
H	-6.02643300	-3.99550500	-0.79646700
H	-6.15148200	-5.17701300	-2.98226100
C	-3.05133500	4.03504200	0.04008700
C	-4.11897300	3.61049800	0.84921200
C	-3.13820200	5.29096600	-0.58248700
C	-5.24173000	4.41905100	1.02607600
H	-4.05658200	2.65305000	1.35867400
C	-4.26450600	6.09565900	-0.41019500
H	-2.32221600	5.62747700	-1.21621900
C	-5.31969900	5.66243700	0.39494200
H	-6.05351200	4.07929400	1.66357600

H	-4.31751900	7.06059600	-0.90702500
H	-6.19596600	6.29010100	0.53168100
Ni	2.39261800	-0.11755700	-0.49286700
C	2.29483800	-0.56097800	1.40627500
C	0.98470700	-1.09308500	1.98513300
H	2.55501900	0.39066200	1.89257100
H	3.11264200	-1.25933000	1.62944500
C	1.04140500	-1.30159400	3.52158700
H	0.15362600	-0.40835000	1.76951800
H	0.71166700	-2.05418300	1.52632200
H	1.85411800	-2.00274000	3.75287300
H	1.30773700	-0.34781000	3.99603800
C	-0.26025900	-1.81435900	4.09745500
C	-0.52196400	-3.18988600	4.18242800
C	-1.26131600	-0.92712700	4.52022000
C	-1.74075900	-3.66457400	4.67013600
H	0.24400000	-3.89579600	3.86714900
C	-2.48216500	-1.39500600	5.00916700
H	-1.07523900	0.14405000	4.47042800
C	-2.72737400	-2.76775000	5.08571500
H	-1.91765200	-4.73576000	4.73170100
H	-3.23980300	-0.68727900	5.33753200
H	-3.67495500	-3.13506900	5.47151600
C	6.24872700	-1.34160800	0.42013200
C	5.12765400	-1.06678700	-0.36209100
C	4.44042100	0.17059700	-0.30863200
C	4.97050500	1.12442200	0.58575100
C	6.09412500	0.87107500	1.37483800
C	6.73454800	-0.36763500	1.29813000
H	6.74457300	-2.30779400	0.35064100
H	4.77602900	-1.84473600	-1.04094100
H	4.49574800	2.09983600	0.65207600
H	6.47263300	1.63624700	2.04976000
H	7.60932700	-0.57142600	1.91139600
B	4.03402300	1.00769300	-2.10704100
O	5.13346500	0.61638000	-2.88929200
H	5.19341200	-0.34441700	-2.96152700
O	3.91792600	2.40444700	-1.94124400
H	4.78364700	2.80230300	-2.11170800
O	2.73695800	0.38715500	-2.40500000
H	2.11010400	1.09829700	-2.61633800

$B(OH)_3$

B	0.00002800	-0.00005300	-0.00064500
O	1.36484300	0.14081900	0.00001800
H	1.80031000	-0.72328500	0.00025100
O	-0.56048500	-1.25229700	0.00017700
H	-1.52652100	-1.19747400	0.00044900
O	-0.80436100	1.11151000	0.00009000
H	-0.27390500	1.92077300	0.00024500

CP25

C	-0.59163400	3.04275100	-2.12429400
C	-1.58170800	2.94760200	-1.16028700
C	-1.86339200	1.64611100	-0.63794300
C	-1.03064200	0.58282000	-1.06136400
C	0.19367800	1.92888100	-2.49760800
C	-2.97537700	1.34273900	0.21335200
C	-1.33123900	-0.77537400	-0.67741700
C	-2.44531000	-1.04126100	0.15448700
C	-3.24943100	0.06071200	0.59440400
C	-2.74648200	-2.41197900	0.42929400
C	-1.99061200	-3.36756300	-0.22756000
C	-0.90582300	-3.02191400	-1.06613600
H	-3.63234700	2.14767500	0.52225000
H	-0.38071800	4.00333100	-2.58490700
H	-4.12133000	-0.14275900	1.20565300
H	-2.21204500	-4.42039500	-0.08050100
N	0.02941000	0.74422400	-1.90674100
N	-0.54291100	-1.74610800	-1.23074700
C	1.19975300	2.06581900	-3.61034000
H	0.69143400	2.42430200	-4.51479700

H	1.98119400	2.79439200	-3.36703000
H	1.66895500	1.10536200	-3.82540600
C	-0.15980200	-4.10837700	-1.78972300
H	0.37763500	-3.70351800	-2.64721100
H	0.58111900	-4.57970000	-1.13497300
H	-0.86127800	-4.88096400	-2.12540000
C	-2.32498600	4.16347000	-0.73817900
C	-2.44859600	4.50267700	0.62024600
C	-2.87621600	5.02654000	-1.69961300
C	-3.10590800	5.67160600	1.00323600
H	-2.00527600	3.85971300	1.37526300
C	-3.53921000	6.19153600	-1.31451100
H	-2.79901900	4.76994100	-2.75263700
C	-3.65570700	6.51805000	0.03803800
H	-3.18219700	5.92407100	2.05729800
H	-3.96744300	6.84233600	-2.07193300
H	-4.17013400	7.42669000	0.33820900
C	-3.83806000	-2.83007000	1.34724600
C	-4.79182400	-3.77314600	0.92987900
C	-3.91211500	-2.33459400	2.66053500
C	-5.79585300	-4.20269800	1.79737600
H	-4.75315700	-4.15553200	-0.08642000
C	-4.91295500	-2.77005300	3.52884100
H	-3.16611900	-1.62532500	3.00775400
C	-5.85951000	-3.70283300	3.09955700
H	-6.53029800	-4.92621700	1.45415800
H	-4.94817000	-2.38498800	4.54429300
H	-6.64022400	-4.03919900	3.77613800
Ni	1.29973600	-0.83807400	-1.60273300
C	2.91279100	0.23239000	-1.35452600
C	3.15922500	0.54736700	0.12422400
H	2.76176400	1.17579000	-1.89477100
H	3.79673600	-0.24983800	-1.78780900
C	4.40246900	1.44537100	0.35942100
H	2.28589800	1.05351700	0.56320300
H	3.29341000	-0.38014500	0.69320900
H	5.28041500	0.93621400	-0.06031400
H	4.28265300	2.37986700	-0.20566000
C	4.64372200	1.76105400	1.81917300
C	5.30968000	0.84732900	2.65041400
C	4.17549000	2.95183300	2.39265900
C	5.50072100	1.11283700	4.00680700
H	5.68424500	-0.08116000	2.22437700
C	4.36372900	3.22382300	3.74936800
H	3.66203700	3.67716100	1.76412800
C	5.02783100	2.30400600	4.56290100
H	6.02373900	0.39108300	4.62968300
H	3.99625700	4.15721100	4.16957100
H	5.17958500	2.51452700	5.61852900
C	2.40662500	-2.32654500	-1.21401200
C	2.43239900	-2.92647400	0.05992300
C	3.18449700	-2.94015900	-2.21529400
C	3.16812100	-4.09027600	0.31613900
H	1.86211500	-2.48563600	0.87730800
C	3.91221900	-4.11014300	-1.97400500
H	3.22543400	-2.49959100	-3.21089300
C	3.90644800	-4.69422100	-0.70446100
H	3.16445600	-4.52514400	1.31455800
H	4.49282600	-4.56239500	-2.77653800
H	4.47771900	-5.59927700	-0.51092700

TS14

C	1.45808600	2.42566400	-2.07628800
C	0.48583100	3.03561700	-1.29645800
C	-0.57894000	2.21311900	-0.81554900
C	-0.50953100	0.82244400	-1.08870800
C	1.43683900	1.04024300	-2.33096300
C	-1.73640800	2.71857600	-0.13863800
C	-1.57356600	-0.05684100	-0.65687300
C	-2.69789200	0.47782700	0.02245400
C	-2.74723400	1.89145800	0.25591400

C	-3.74724800	-0.42419300	0.37589300
C	-3.61854900	-1.73948300	-0.04627500
C	-2.47455300	-2.19646100	-0.72976000
H	-1.81862100	3.78625300	0.03051800
H	2.26900100	3.02136500	-2.48525400
H	-3.62552900	2.30917700	0.73477800
H	-4.40599300	-2.45263400	0.17810100
N	0.50259400	0.24373900	-1.79801800
N	-1.44056300	-1.37876800	-0.97944800
C	2.48607300	0.41628800	-3.21312900
H	2.86199700	1.13942500	-3.94394600
H	3.34531900	0.06458500	-2.62701300
H	2.07289300	-0.44517200	-3.74507300
C	-2.37958000	-3.62733800	-1.17368100
H	-1.88422700	-3.69469400	-2.14489500
H	-1.77864700	-4.21426200	-0.47012000
H	-3.37471900	-4.07866600	-1.24056700
C	0.57520000	4.49174600	-1.01268800
C	0.51628600	4.98207900	0.30352500
C	0.77348800	5.40718300	-2.06002600
C	0.64958100	6.34615600	0.56184200
H	0.38844800	4.28592200	1.12754500
C	0.90105400	6.77169600	-1.80087700
H	0.80850400	5.04439300	-3.08369100
C	0.83985300	7.24606700	-0.48907600
H	0.61108900	6.70499000	1.58685200
H	1.04447700	7.46477200	-2.62548800
H	0.94070500	8.30883500	-0.28694200
C	-4.95246000	-0.00921800	1.13999100
C	-6.23508100	-0.33137700	0.66482400
C	-4.84411400	0.66274800	2.37015100
C	-7.37398200	0.01480500	1.39147800
H	-6.33511600	-0.84157800	-0.28930400
C	-5.98359100	1.00457300	3.09820300
H	-3.85996100	0.89510600	2.76702200

C	-7.25257800	0.68440000	2.61075700
H	-8.35716900	-0.23599900	1.00235400
H	-5.87857000	1.51502500	4.05182700
H	-8.13967900	0.95303900	3.17772600
Ni	0.51386000	-1.79229100	-1.30140700
C	2.46385900	-2.41780500	-0.83582400
C	2.89095000	-1.63618700	0.40943800
H	2.59491000	-1.81362200	-1.74940600
H	3.14402800	-3.25862000	-0.98237700
C	4.39468300	-1.26899900	0.39948200
H	2.30229300	-0.71706200	0.51699500
H	2.69001800	-2.23591000	1.30550600
H	4.98208500	-2.19107000	0.29547900
H	4.61260900	-0.65859400	-0.48755600
C	4.82593500	-0.52441300	1.64487900
C	5.25952800	-1.21776200	2.78394400
C	4.76334900	0.87498600	1.70553900
C	5.61852300	-0.53610600	3.94778800
H	5.31950200	-2.30384000	2.75505300
C	5.12108400	1.56219400	2.86670100
H	4.43430300	1.43156600	0.82993900
C	5.55038900	0.85797200	3.99349500
H	5.95597700	-1.09395500	4.81777100
H	5.06948700	2.64799400	2.88940100
H	5.83326900	1.39076200	4.89759000
C	1.03239100	-3.50741700	-0.69100000
C	0.69604600	-3.93031500	0.62951100
C	1.00820800	-4.52844900	-1.69538000
C	0.32698800	-5.24662500	0.90676400
H	0.70870300	-3.20745500	1.44158900
C	0.62687300	-5.83304000	-1.40939500
H	1.31014700	-4.27579500	-2.71089300
C	0.28177400	-6.21352200	-0.10357400
H	0.06358000	-5.51925900	1.92766100
H	0.60705600	-6.57067000	-2.21006100

H	0.00016000	-7.23874200	0.12006600

3b

C	-2.54746300	0.00087300	-0.51714500
C	-3.13374900	1.20407000	-0.10024900
C	-3.14115000	-1.20209200	-0.11029400
C	-4.27696200	1.20738700	0.70045800
H	-2.69107200	2.14838500	-0.41107100
C	-4.28443900	-1.20505500	0.69035300
H	-2.70426800	-2.14647900	-0.42897800
C	-4.85649700	0.00124000	1.09947600
H	-4.71754000	2.15211400	1.00893500
H	-4.73085100	-2.14959500	0.99090000
H	-5.74858000	0.00141100	1.72015400
C	-1.28396600	0.00058700	-1.35137600
C	-0.00000300	-0.00018100	-0.49732700
H	-1.27411600	0.88052600	-2.00877600
H	-1.27495700	-0.87895100	-2.00928400
C	1.28396100	-0.00126100	-1.35137300
H	0.00066600	0.87722500	0.16258400
H	-0.00067400	-0.87733200	0.16292100
H	1.27415500	-0.88146900	-2.00841000
H	1.27492000	0.87800600	-2.00964400
C	2.54746200	-0.00113000	-0.51713800
C	3.14102000	1.20204300	-0.11073000
C	3.13388000	-1.20411600	-0.09981000
C	4.28431800	1.20541400	0.68992200
H	2.70404800	2.14627800	-0.42974800
C	4.27708800	-1.20702700	0.70088500
H	2.69129200	-2.14858000	-0.41030000
C	4.85650000	-0.00067500	1.09947300
H	4.73063100	2.15011300	0.99012500
H	4.71777000	-2.15159100	1.00970200
H	5.74858500	-0.00052400	1.72014800

TS15

C	0.17745800	3.52889100	-1.28885400
C	1.42848900	3.37970100	-0.72691300
C	1.74369000	2.09871800	-0.18332300
C	0.78551700	1.06252000	-0.33205700
C	-0.74043100	2.45833000	-1.40118100
C	2.94113600	1.84290000	0.55823400
C	1.04703600	-0.22745200	0.26402400
C	2.25869100	-0.45489700	0.97041100
C	3.19097500	0.62195300	1.10773200
C	2.45115300	-1.73551200	1.56842200
C	1.40829400	-2.64126600	1.47827300
C	0.22309500	-2.34641100	0.77537000
H	3.64163700	2.65612600	0.70797600
H	-0.11680600	4.49237500	-1.69178500
H	4.08932900	0.46423800	1.69283900
H	1.50993200	-3.62129500	1.93232100
N	-0.41602000	1.22864900	-0.97372900
N	0.06749000	-1.17246300	0.15010200
C	-2.06469800	2.75604500	-2.04460800
H	-2.33204100	3.79546400	-1.83149800
H	-1.99133000	2.66143800	-3.13719500
H	-2.86903600	2.12113200	-1.66529700
C	-0.87095000	-3.37657400	0.72090500
H	-1.82342300	-2.93789900	1.03091700
H	-0.99281900	-3.75907900	-0.29824300
H	-0.63814900	-4.21741600	1.38074300
C	2.36517900	4.53413200	-0.68841300
C	3.66545500	4.43751000	-1.21259700
C	1.93814100	5.76740200	-0.16929800
C	4.51456800	5.54403700	-1.21432600
H	4.00213100	3.49729600	-1.64043600
C	2.79124100	6.87089800	-0.16520300
H	0.93851300	5.85170700	0.24784000
C	4.08144500	6.76295600	-0.68795900
H	5.51333400	5.45476000	-1.63297400
H	2.44741000	7.81460300	0.24916400

H	4.74486200	7.62336600	-0.68678700
C	3.69759300	-2.11901800	2.28282200
C	3.62883600	-2.65228400	3.58055700
C	4.95572800	-2.01052000	1.66582600
C	4.78580500	-3.05643700	4.24645600
H	2.66371100	-2.73073700	4.07336200
C	6.11092200	-2.42187700	2.33030100
H	5.02409000	-1.62207700	0.65356000
C	6.03018500	-2.94321800	3.62341500
H	4.71430700	-3.45697500	5.25390800
H	7.07433900	-2.33983800	1.83451500
H	6.93113100	-3.25992300	4.14142400
Ni	-1.43813800	-0.54849600	-1.02169000
C	-2.44584800	-0.26117200	-2.82288400
C	-1.99653000	-1.55874500	-2.96597000
H	-1.89281800	0.53222700	-3.31395200
H	-3.44589300	-0.02617500	-2.46692200
C	-0.77883100	-1.94472500	-3.78427300
H	-1.97296500	-1.88696500	-0.98613400
H	-2.68696000	-2.37597700	-2.76913100
H	-1.13917000	-2.15057600	-4.80579600
H	-0.09902600	-1.08935600	-3.85989600
C	-0.03113100	-3.16702000	-3.28568500
C	-0.60776700	-4.44252700	-3.37634900
C	1.24954500	-3.05356500	-2.73165200
C	0.07099800	-5.57105100	-2.91393100
H	-1.59474100	-4.55518200	-3.82077100
C	1.93528000	-4.18041500	-2.27190600
H	1.71333500	-2.07251800	-2.66036900
C	1.34682800	-5.44318500	-2.35882000
H	-0.39259800	-6.55078200	-2.99533500
H	2.92982500	-4.07053900	-1.84730100
H	1.87950200	-6.32153200	-2.00430300
B	-3.85923700	1.03226100	0.58812100
O	-3.06292300	2.22905700	0.84295300
H	-2.59267900	2.09261600	1.67727400
O	-4.48005900	1.10779100	-0.75139300
H	-5.21813200	1.73194400	-0.70726500
O	-2.89823000	-0.15228200	0.54830000
H	-3.43484800	-0.95364800	0.62858000
C	-4.99205300	0.72712200	1.74330100
C	-5.94029600	-0.30131200	1.57667900
C	-5.07159500	1.45732100	2.94134600
C	-6.89396700	-0.60354100	2.55216100
H	-5.94388800	-0.86663400	0.64382400
C	-6.01978000	1.17185100	3.92776300
H	-4.37950900	2.28388400	3.09576500
C	-6.93441800	0.13318500	3.73880400
H	-7.61100100	-1.40607100	2.38628600
H	-6.05036100	1.76175300	4.84248600
H	-7.67567900	-0.09348500	4.50224300

CP26

C	-1.98536400	3.50324400	-0.43778800
C	-3.09133200	2.74081400	-0.09803200
C	-2.94925800	1.32178300	-0.12283000
C	-1.69922700	0.78856700	-0.54420300
C	-0.76863500	2.90406000	-0.81993100
C	-3.97756900	0.42309800	0.30558300
C	-1.50570700	-0.64815500	-0.60847100
C	-2.57674200	-1.50086600	-0.22968300
C	-3.79427600	-0.92516600	0.25744300
C	-2.36770000	-2.90938300	-0.30739300
C	-1.12299600	-3.34100100	-0.71942700
C	-0.09796800	-2.43787400	-1.07487600
H	-4.90141100	0.83196400	0.69755100
H	-2.05863100	4.58592500	-0.43418700
H	-4.57398900	-1.58852200	0.61298900
H	-0.92313500	-4.40410700	-0.80024000
N	-0.64377600	1.57587200	-0.88358600
N	-0.29238500	-1.11464900	-1.02653300

C	0.41362500	3.76899400	-1.16942700
H	0.15336800	4.82930900	-1.10250400
H	1.24875200	3.56581800	-0.49122200
H	0.76475400	3.55605000	-2.18424500
C	1.21762800	-2.98281200	-1.54347900
H	2.03356400	-2.53814700	-0.95511300
H	1.24134800	-4.07009700	-1.42816100
H	1.37205400	-2.75977200	-2.60630400
C	-4.36056200	3.42125900	0.27494500
C	-4.35879800	4.41487400	1.26763500
C	-5.56648100	3.13039100	-0.38529200
C	-5.53249400	5.09168100	1.59905900
H	-3.43496600	4.64138500	1.79274900
C	-6.73742400	3.81391400	-0.05831000
H	-5.57923400	2.38141200	-1.17196800
C	-6.72493700	4.79393900	0.93663100
H	-5.51488400	5.84981500	2.37713800
H	-7.65920800	3.58374800	-0.58553300
H	-7.63867500	5.32276700	1.19310300
C	-3.41728700	-3.90896700	0.02863000
C	-4.66951600	-3.89950800	-0.60867500
C	-3.14270500	-4.92281400	0.96085500
C	-5.62116000	-4.87698900	-0.31812800
H	-4.88787500	-3.13638300	-1.35059600
C	-4.09853200	-5.89498700	1.25573000
H	-2.18097200	-4.93319900	1.46607500
C	-5.33997100	-5.87552100	0.61693000
H	-6.58090800	-4.86142300	-0.82750800
H	-3.87288000	-6.66650200	1.98675100
H	-6.08329800	-6.63448300	0.84483800
C	1.22990400	0.22821300	-3.32865100
C	2.50073700	-0.07908100	-2.83187200
H	1.06099000	1.17758900	-3.83396800
H	0.51132600	-0.55556200	-3.55168700
C	3.71656100	0.79285300	-3.10940200
H	1.91495800	1.61196800	-1.45522100
H	2.73759700	-1.10793800	-2.58104300
H	4.06735800	0.51923700	-4.11651400
H	3.40578300	1.84325400	-3.17254900
C	4.88909900	0.64122500	-2.15500900
C	4.80646500	1.05093900	-0.81464100
C	6.09466800	0.09245200	-2.60985000
C	5.89957800	0.90951800	0.04337300
H	3.87600600	1.45714000	-0.42999400
C	7.19203500	-0.04684400	-1.75600600
H	6.17754700	-0.22785900	-3.64675500
C	7.09698400	0.36112000	-0.42568800
H	5.80505600	1.21627800	1.08115300
H	8.11724800	-0.47632300	-2.13221400
H	7.94569300	0.25003600	0.24398800
B	2.29519300	-0.73070100	1.35382400
O	1.12067700	-1.49269400	1.78209500
H	1.36273500	-2.01771700	2.55675100
O	3.08502900	-1.49124600	0.38723400
H	3.93185800	-1.04845100	0.23419200
O	1.68758500	0.49782400	0.62825600
H	0.88418900	0.70340900	1.13059500
C	3.22068000	-0.21905100	2.60717000
C	3.16326600	1.07735800	3.14969600
C	4.11524800	-1.11486200	3.22600300
C	3.94083700	1.46173600	4.24630900
H	2.49971100	1.80912300	2.69184100
C	4.89540700	-0.74945900	4.32610300
H	4.21043800	-2.12119200	2.81990800
C	4.81036400	0.54568000	4.84310300
H	3.87070500	2.47616400	4.63560700
H	5.57544000	-1.47048100	4.77659100
H	5.41733500	0.83874500	5.69705200
Ni	1.12344400	0.43623800	-1.32032600

CP27

C	-1.60855800	-2.94595300	1.03297300	H	-6.75323900	-5.29337600	1.25218000
C	-2.85875200	-2.83059100	0.46430300	H	-5.86058400	-7.28458900	0.05733000
C	-3.25145800	-1.54165200	-0.01004800	C	-5.60994300	2.64778700	-2.13134500
C	-2.35538400	-0.45249600	0.17418500	C	-6.81699900	2.41929200	-1.44919500
C	-0.76598500	-1.82618900	1.19882400	C	-5.65983400	3.24556000	-3.40113200
C	-4.48162700	-1.33317700	-0.71202000	C	-8.03716000	2.77647400	-2.02304300
C	-2.71110600	0.85102200	-0.37555700	H	-6.79439200	1.97898600	-0.45631600
C	-3.97489000	1.02372100	-1.01045800	C	-6.88098200	3.59673600	-3.97694800
C	-4.83650900	-0.10481300	-1.18000300	H	-4.73401800	3.42003100	-3.94255000
C	-4.29638900	2.32491800	-1.50873300	C	-8.07365500	3.36391200	-3.28959400
C	-3.33637100	3.31369400	-1.39724900	H	-8.95988700	2.60113900	-1.47642600
C	-2.09032500	3.04539700	-0.78580800	H	-6.89979900	4.05040400	-4.96426600
H	-5.12824700	-2.18517700	-0.88662100	H	-9.02486200	3.63952800	-3.73667600
H	-1.25706300	-3.91200400	1.38048300	C	-0.60251000	1.40083400	2.89609500
H	-5.76663400	0.02010900	-1.72250900	H	-0.00529300	2.30136200	3.05555200
H	-3.54770500	4.31607200	-1.75896000	B	1.95912800	-0.37545500	-1.30171600
N	-1.15778200	-0.60496800	0.82033800	O	2.57305500	0.30398300	-2.51182900
N	-1.80450300	1.85225500	-0.28226600	H	2.32684900	-0.15859800	-3.32391600
C	0.59665900	-2.04687000	1.79400100	O	3.05080000	-0.37768000	-0.29291900
H	0.51790400	-2.33733200	2.84672200	H	2.89032500	0.34917400	0.33800700
H	1.09597800	-2.85300400	1.24639400	O	0.81128500	0.48027500	-0.85716400
H	1.21497900	-1.15212500	1.72369700	H	1.01243500	1.37623000	-1.17022500
C	-1.05042500	4.13250900	-0.68205900	C	1.44922700	-1.86603100	-1.69491000
H	-0.20325200	3.79875200	-0.07888100	C	0.28157500	-2.04560100	-2.46032000
H	-0.68527400	4.41406700	-1.67911600	C	2.16411100	-3.02840900	-1.35371200
H	-1.47576700	5.03738700	-0.23050700	C	-0.14913800	-3.31062900	-2.87101300
C	-3.71510600	-4.04108600	0.33140500	H	-0.30966900	-1.17135800	-2.72697700
C	-3.21607300	-5.17437700	-0.33125900	C	1.74492800	-4.30060200	-1.75354100
C	-4.99953000	-4.09917200	0.89690400	H	3.06115800	-2.92739100	-0.74763700
C	-3.98668100	-6.33312100	-0.43190400	C	0.58450800	-4.44679000	-2.51878200
H	-2.22845300	-5.13199100	-0.78297500	H	-1.05594900	-3.41131000	-3.46439200
C	-5.76518500	-5.26132900	0.80080500	H	2.32148000	-5.17933700	-1.46976800
H	-5.38923200	-3.23620400	1.42988300	H	0.25901200	-5.43371800	-2.84109300
C	-5.26219900	-6.38079100	0.13411900	Ni	0.08710600	0.92927500	1.06635200
H	-3.58992300	-7.19885400	-0.95547400	Br	1.97109800	2.50743000	1.25979600

C	-2.08258300	1.79585500	2.92097500
C	-2.52194700	2.44662800	4.24679700
H	-2.73409600	0.93671100	2.71055300
H	-2.26465500	2.51204400	2.11385000
H	-3.57720500	2.74763800	4.20939300
H	-2.39427200	1.76493900	5.09437900
H	-1.92497900	3.34384700	4.45317500
C	-0.18353500	0.36952200	3.89180800
C	1.14346100	0.35705000	4.37563400
C	-1.05598900	-0.61473700	4.39974700
C	1.57693000	-0.59318900	5.29560000
H	1.83654200	1.10559000	4.00009200
C	-0.62486700	-1.56660700	5.32690100
H	-2.09139300	-0.63529200	4.07163200
C	0.69619200	-1.56844500	5.77806800
H	2.60722900	-0.57187100	5.64454200
H	-1.32858700	-2.30738100	5.70133700
H	1.03195700	-2.30754800	6.50117200
Li	4.31866000	0.27920300	-1.67332400
C	4.75533600	3.09528200	-1.00722500
O	5.16469800	1.93444100	-1.19435600
C	6.33389000	-1.82090400	-1.59101500
O	5.69035900	-0.96843300	-2.22409800
N	7.35911100	-2.49600400	-2.18284300
N	5.16782900	3.81868600	0.06412400
C	5.99543000	-2.12531500	-0.14168500
H	5.85226400	-3.19711300	0.03000100
H	6.79298400	-1.78014400	0.52726500
H	5.06941800	-1.60246600	0.10727800
C	7.68365000	-2.24479100	-3.58232600
H	7.62153700	-3.17828700	-4.15566000
H	6.97622100	-1.52146600	-3.98271600
H	8.70328700	-1.84867200	-3.67158300
C	8.19268000	-3.48871500	-1.52308700
H	7.96197300	-3.55800200	-0.46186000
H	8.05163000	-4.47843500	-1.97827200
H	9.25064700	-3.21553500	-1.62697000
C	3.79589200	3.74026600	-1.98909200
H	4.19865800	4.66894000	-2.40884500
H	3.60825400	3.02932900	-2.79393200
H	2.84802100	3.96498400	-1.48911400
C	4.56425300	5.09121400	0.44697500
H	5.31105100	5.69209500	0.97584600
H	4.24165400	5.65015900	-0.43134900
H	3.70048300	4.92855100	1.10387300
C	5.93439000	3.14890700	1.11124200
H	5.26140700	2.77080000	1.89175500
H	6.47870300	2.31358300	0.67422500
H	6.63871300	3.86037800	1.55444300

TS16

C	-2.33726400	-3.17828600	0.18240900
C	-3.57245200	-2.64439800	-0.12642900
C	-3.67072400	-1.22363600	-0.21247400
C	-2.51694200	-0.44752300	0.09290600
C	-1.23215200	-2.35819400	0.49159200
C	-4.86890000	-0.56879700	-0.64409300
C	-2.58530900	1.00091300	-0.06907500
C	-3.82207300	1.61160200	-0.42414900
C	-4.95001800	0.78708100	-0.73054200
C	-3.85690300	3.03778900	-0.52116600
C	-2.67387600	3.72668000	-0.32398300
C	-1.47787800	3.03626700	-0.02547400
H	-5.71908500	-1.17927800	-0.92506600
H	-2.21129000	-4.25440900	0.23210400
H	-5.86589000	1.25428800	-1.07362100
H	-2.66423000	4.81171000	-0.37745100
N	-1.34751300	-1.02292800	0.50532900
N	-1.44750500	1.71588900	0.10172400
C	0.09416000	-2.99742000	0.78423900
H	0.49715100	-2.66100800	1.74118500

H	-0.00677000	-4.08646700	0.81326000
H	0.82335800	-2.73116100	0.01076500
C	-0.19451600	3.80115100	0.17789900
H	0.61671500	3.12496000	0.45824700
H	0.08314500	4.34100000	-0.73729000
H	-0.31471400	4.55257000	0.96879100
C	-4.72283100	-3.55591700	-0.37297600
C	-4.60875700	-4.58885600	-1.31750300
C	-5.91507800	-3.44354300	0.36202600
C	-5.66187500	-5.47909600	-1.52851200
H	-3.69503600	-4.67982400	-1.89832500
C	-6.96414000	-4.33927800	0.15516800
H	-6.00949500	-2.66315000	1.11202000
C	-6.84224700	-5.35760300	-0.79282700
H	-5.55960900	-6.26666700	-2.27016700
H	-7.87532400	-4.24423300	0.73967200
H	-7.66139700	-6.05271000	-0.95556400
C	-5.10422900	3.79520500	-0.81601600
C	-6.25809800	3.63564600	-0.02985700
C	-5.12876700	4.73267400	-1.86143500
C	-7.40292500	4.39014300	-0.28590700
H	-6.24799100	2.93149200	0.79723200
C	-6.27618400	5.48261700	-2.12036200
H	-4.24613400	4.85883600	-2.48251500
C	-7.41685600	5.31397600	-1.33321300
H	-8.28291000	4.26014000	0.33827400
H	-6.27919200	6.19662200	-2.93955000
H	-8.31026400	5.89911600	-1.53331400
C	-0.21589100	0.22949100	2.92922200
H	0.55078400	0.95369200	3.21404500
B	1.88936100	-0.71012800	-1.74997800
O	2.71855100	0.46858100	-2.25066600
H	3.04524300	0.25785900	-3.13708900
O	2.64125900	-1.48742300	-0.76589900
H	2.54358600	-1.06543700	0.10618000
O	0.71019100	-0.07096800	-1.05303900
H	0.65108900	0.84406800	-1.36189800
C	1.44495300	-1.63369200	-3.01326700
C	0.78152500	-1.07244000	-4.12154800
C	1.70058000	-3.01494100	-3.06763200
C	0.39075200	-1.83958200	-5.22128000
H	0.56034000	-0.00510700	-4.12267500
C	1.31675100	-3.79652500	-4.16162100
H	2.21323500	-3.48104400	-2.22978600
C	0.65857000	-3.21077000	-5.24489800
H	-0.12330700	-1.37123600	-6.05863600
H	1.53243600	-4.86358000	-4.17052100
H	0.35804800	-3.81445600	-6.09857000
Ni	0.25005700	0.08095000	0.97322300
Br	2.45979800	1.12144800	1.35209200
C	-1.58700900	0.85404900	3.21185900
C	-1.79257300	1.23358300	4.69106800
H	-2.40586300	0.19365000	2.89592000
H	-1.68706000	1.76315600	2.61100200
H	-2.76993900	1.70932500	4.84621400
H	-1.73489100	0.35661100	5.34441200
H	-1.02004300	1.94014800	5.01959400
C	0.06348400	-1.06159500	3.62816300
C	1.38981200	-1.40644300	3.97212100
C	-0.94462600	-1.98508600	3.97499500
C	1.69042600	-2.60846200	4.60835700
H	2.18745500	-0.71544200	3.71307300
C	-0.64486000	-3.18941900	4.61516000
H	-1.98219900	-1.75851900	3.74597000
C	0.67463200	-3.51378200	4.93376900
H	2.72417900	-2.83914500	4.85695600
H	-1.45016300	-3.87516300	4.87053800
H	0.90791500	-4.45048600	5.43363300
Li	4.12917000	0.52897400	-0.90786300
C	4.41417000	3.44115200	-0.81185200

O	4.94584100	2.32073300	-0.84634600
C	6.36410400	-1.56049000	-0.73613700
O	5.79014700	-0.46738300	-0.85261500
N	7.72767500	-1.62010000	-0.71303500
N	4.79152300	4.36750500	0.11151900
C	5.57076900	-2.84770500	-0.61736600
H	5.81367000	-3.54245000	-1.42971400
H	5.77609300	-3.35590800	0.33220000
H	4.51079600	-2.58775700	-0.66985700
C	8.51066900	-0.39523900	-0.82268400
H	9.15475300	-0.43452800	-1.71084600
H	7.82971900	0.45042900	-0.90154900
H	9.14902200	-0.27409100	0.06210000
C	8.50341700	-2.84273200	-0.58502600
H	7.85627000	-3.71383200	-0.50523400
H	9.15663100	-2.97395200	-1.45828400
H	9.13933700	-2.79902300	0.30938200
C	3.34331000	3.81183900	-1.82285600
H	3.74183000	4.50154200	-2.57755600
H	3.02238800	2.88917500	-2.30896200
H	2.47574200	4.28363200	-1.35319900
C	4.22473100	5.70289500	0.21692700
H	5.02656700	6.41930300	0.43107400
H	3.74654700	6.00357200	-0.71451700
H	3.48803500	5.76137900	1.03034300
C	5.71723200	3.99367000	1.17486500
H	5.23308900	4.10931000	2.15229400
H	6.00398300	2.95341200	1.03617100
H	6.60907200	4.63279300	1.14536500

CP28

C	-2.86365100	-2.91938800	-0.55578500
C	-3.96919400	-2.10071600	-0.68356100
C	-3.78566000	-0.70616200	-0.44293400
C	-2.49943700	-0.26184500	-0.02976500
C	-1.62272000	-2.42594400	-0.09949200
C	-4.82901700	0.25457600	-0.64747900
C	-2.26227700	1.17042500	0.10723700
C	-3.35232100	2.07829200	-0.01319500
C	-4.63301800	1.58226100	-0.41695200
C	-3.08652700	3.46380300	0.21321700
C	-1.77526300	3.83923600	0.45646100
C	-0.73996200	2.87938500	0.47334100
H	-5.79020600	-0.09447200	-1.00650500
H	-2.95264100	-3.98132100	-0.75838000
H	-5.44075400	2.28569000	-0.58388100
H	-1.53953200	4.88220900	0.64706200
N	-1.47528600	-1.13140200	0.21525000
N	-0.99042400	1.58407500	0.31363400
C	-0.46017900	-3.36660400	0.03454700
H	-0.20441400	-3.52795900	1.08532400
H	-0.71053000	-4.33588200	-0.40780500
H	0.42843300	-2.96923000	-0.46716600
C	0.69612700	3.29594100	0.66244300
H	1.27439700	2.49021300	1.12252900
H	1.15881700	3.52081600	-0.30848400
H	0.76826300	4.19963100	1.27745100
C	-5.27589400	-2.69200000	-1.08139200
C	-5.36534200	-3.47779700	-2.24175700
C	-6.42364800	-2.52930600	-0.28745900
C	-6.57206600	-4.07668100	-2.60399800
H	-4.48616100	-3.60348500	-2.86763900
C	-7.62757100	-3.13503700	-0.64712900
H	-6.36383600	-1.94362100	0.62565600
C	-7.70650400	-3.90766200	-1.80790100
H	-6.62506900	-4.67424200	-3.51001300
H	-8.50305900	-3.00759600	-0.01607900
H	-8.64570600	-4.37646500	-2.08860200
C	-4.15495900	4.49984800	0.20189900
C	-5.29679300	4.37960700	1.01245200
C	-4.01178100	5.65255000	-0.58777100

C	-6.26640500	5.38205400	1.02888800
H	-5.41162900	3.50565200	1.64723500
C	-4.98496100	6.65179600	-0.57521100
H	-3.13893200	5.75304100	-1.22710500
C	-6.11543100	6.52004200	0.23348600
H	-7.13787300	5.27604100	1.66932000
H	-4.86102200	7.53194600	-1.20045900
H	-6.87297300	7.29887900	0.24495800
C	-0.31911900	-0.65706700	2.87655900
H	0.57122400	-0.24647200	3.35697800
B	1.96827100	-1.01760300	-1.84176400
O	2.98116800	0.11966300	-1.99540100
H	3.58009600	-0.13457200	-2.71379300
O	2.50162200	-2.14243500	-1.09758300
H	2.59875000	-1.86244700	-0.17030400
O	0.86025600	-0.41152800	-1.02009500
H	0.61429000	0.46780600	-1.33829500
C	1.46409600	-1.48910900	-3.31714700
C	1.10519100	-0.55482100	-4.30689500
C	1.34515700	-2.84707100	-3.66292900
C	0.64212500	-0.94467300	-5.56624500
H	1.19645900	0.50947700	-4.08893800
C	0.88786300	-3.25343700	-4.91953100
H	1.62611300	-3.59457200	-2.92531000
C	0.53084300	-2.30176800	-5.87778700
H	0.37115200	-0.19294400	-6.30567800
H	0.81197700	-4.31386300	-5.15426000
H	0.17403900	-2.61331700	-6.85725800
Ni	0.27646900	-0.51337200	0.95834500
Br	2.59194400	-0.05358600	1.67595400
C	-1.50743900	0.23547100	3.25113000
C	-1.74998500	0.32272900	4.77026100
H	-2.43363100	-0.09431700	2.76220400
H	-1.31541900	1.24742500	2.87868200
H	-2.58311300	0.99979100	5.00060800

H	-1.98266300	-0.65855900	5.19729500
H	-0.85774400	0.70268000	5.28321400
C	-0.44035000	-2.09771300	3.25228400
C	0.71899100	-2.84510500	3.55754400
C	-1.67361800	-2.77781100	3.32102600
C	0.65016800	-4.19498000	3.89361400
H	1.68325300	-2.34585600	3.50857300
C	-1.74472900	-4.13066400	3.66019900
H	-2.59472000	-2.24175200	3.10954800
C	-0.58420300	-4.85187900	3.94496500
H	1.56477500	-4.73855800	4.12062600
H	-2.71483700	-4.62126600	3.70627500
H	-0.63923400	-5.90465300	4.21000100
Li	3.99954600	0.52641400	-0.40813100
C	4.34065100	3.43966200	-1.05313300
O	4.50826200	2.42094100	-0.36775200
C	6.31381900	-1.45136300	-0.15760700
O	5.71251100	-0.37548700	-0.30383100
N	7.53019300	-1.48133400	0.45948200
N	4.77447100	4.65504300	-0.60937800
C	5.72219300	-2.75152200	-0.66818400
H	6.30787900	-3.14181300	-1.50983600
H	5.70200500	-3.52118400	0.11070200
H	4.69747600	-2.56459500	-1.00118300
C	8.09584600	-0.25720500	1.01317600
H	9.03558300	-0.00542500	0.50412900
H	7.38035200	0.55192900	0.87971400
H	8.30520200	-0.39109200	2.08194800
C	8.33649600	-2.67685600	0.64878600
H	7.91795800	-3.52500000	0.11068700
H	9.35359900	-2.50149700	0.27520100
H	8.40625400	-2.93621600	1.71405300
C	3.64854100	3.35689100	-2.40353500
H	4.35340500	3.55718300	-3.21969000
H	3.25548900	2.34329500	-2.51066000

H	2.82506600	4.07389000	-2.49066300
C	4.69177400	5.89478400	-1.36439900
H	5.69231800	6.33120900	-1.48349600
H	4.27365600	5.72755900	-2.35520800
H	4.06417100	6.62680400	-0.83836800
C	5.41217600	4.76362600	0.69775900
H	4.90942800	5.53452600	1.29458800
H	5.34078000	3.80336500	1.20492600
H	6.46844300	5.04321000	0.58851900

TS17

C	-2.70171600	-3.20058900	0.10337600
C	-3.80401200	-2.44302600	-0.23990600
C	-3.74181700	-1.04013900	0.00568400
C	-2.56176900	-0.51407000	0.60254200
C	-1.55297200	-2.61073300	0.66864200
C	-4.79899000	-0.15132300	-0.37462200
C	-2.50892100	0.91153400	0.92017900
C	-3.62702000	1.73842700	0.60646400
C	-4.74338600	1.17831100	-0.09340800
C	-3.56312800	3.11284300	0.98257300
C	-2.41987700	3.55250300	1.62721700
C	-1.35788300	2.66723700	1.90765200
H	-5.64843100	-0.55647000	-0.91242500
H	-2.71255900	-4.27332800	-0.05769500
H	-5.54761000	1.83325700	-0.40826100
H	-2.34934000	4.58472900	1.95610700
N	-1.48812900	-1.29628100	0.90356400
N	-1.40200100	1.38816900	1.53703000
C	-0.38237100	-3.47764900	1.03336100
H	-0.28572800	-3.54592400	2.12140400
H	-0.51626600	-4.48960400	0.63957500
H	0.54255100	-3.05206500	0.63421800
C	-0.16031200	3.14732000	2.68237200
H	0.76389900	2.91821700	2.14316700
H	-0.21443600	4.22532600	2.86285100
H	-0.10973300	2.63981000	3.65320500
C	-4.99144500	-3.11210300	-0.83922200
C	-4.84354700	-3.88773900	-2.00071900
C	-6.25772200	-3.03109800	-0.23632200
C	-5.93834000	-4.55827700	-2.54759200
H	-3.87151200	-3.94122900	-2.48342600
C	-7.34871200	-3.70804900	-0.78173000
H	-6.38039000	-2.45006600	0.67361500
C	-7.19268700	-4.47223400	-1.94024300
H	-5.81010600	-5.14706900	-3.45207500
H	-8.31962000	-3.64258400	-0.29768200
H	-8.04345700	-4.99747300	-2.36613900
C	-4.67220000	4.07507400	0.73214400
C	-5.97073300	3.83995600	1.21470700
C	-4.41798500	5.27577500	0.04973700
C	-6.98474500	4.77690500	1.01531100
H	-6.17886800	2.92670400	1.76485200
C	-5.43419200	6.20945200	-0.15500500
H	-3.41908400	5.46653500	-0.33305400
C	-6.72104800	5.96318700	0.32714100
H	-7.98085000	4.58158000	1.40352100
H	-5.22001200	7.12882500	-0.69349600
H	-7.51252700	6.69088200	0.16972600
C	0.82346300	-0.48871700	2.97126700
H	1.12044000	0.55773500	3.12593300
B	0.90816900	-0.83093800	-1.60756700
O	1.90919400	-0.05501600	-2.43175600
H	2.08782500	-0.54129600	-3.24876700
O	1.60482700	-1.63206600	-0.57375000
H	2.51626400	-1.31750900	-0.46151800
O	0.07938500	0.21264400	-0.89441400
H	0.57071700	1.05347100	-0.93479600
C	-0.01948600	-1.76770100	-2.55183000
C	-1.12611000	-1.24091200	-3.24361300
C	0.28968100	-3.11950600	-2.78650400

C	-1.88343700	-2.01599400	-4.12516200
H	-1.39953000	-0.20160500	-3.07565500
C	-0.45867600	-3.90864400	-3.66527500
H	1.13250500	-3.55977200	-2.25830400
C	-1.54972300	-3.35695200	-4.34167100
H	-2.73262400	-1.57741900	-4.64594900
H	-0.19109200	-4.95165500	-3.82512600
H	-2.13027100	-3.96254100	-5.03486300
Ni	0.36336600	-0.21478500	1.07678500
Br	2.47952400	1.80357300	0.49918700
C	-0.37441100	-0.80790600	3.87125000
C	-0.10295100	-0.60222700	5.37355400
H	-0.72524100	-1.83557100	3.70817100
H	-1.20756400	-0.16334500	3.57326000
H	-0.99789100	-0.81710500	5.97199800
H	0.70588000	-1.24942300	5.73047800
H	0.19360700	0.43482100	5.57758000
C	2.03197400	-1.35083400	3.15500100
C	3.32131900	-0.82013800	2.92495800
C	1.97107400	-2.68500300	3.61103000
C	4.47296600	-1.57326300	3.14534600
H	3.40571700	0.20166500	2.56872400
C	3.12179500	-3.44499300	3.82248200
H	1.00778000	-3.13100500	3.83619100
C	4.38562200	-2.89523000	3.59528600
H	5.44723600	-1.11532800	2.98343900
H	3.02970200	-4.46615700	4.18613700
H	5.28361300	-3.47809100	3.78798500
Li	3.46985300	0.62929500	-1.50619500
C	4.08971700	3.03327900	-2.87041700
O	4.51459600	1.91554800	-2.52432200
C	5.26989500	-1.74362900	-0.47280800
O	4.50365800	-0.88747200	-0.94486100
N	6.54919300	-1.43124700	-0.14016600
N	4.93110500	4.09956800	-2.95603700
C	4.77707800	-3.16463700	-0.27155600
H	5.40661800	-3.89358700	-0.79324100
H	4.74968200	-3.42004000	0.79324900
H	3.76058300	-3.22012500	-0.66252000
C	7.03415100	-0.06373500	-0.30161900
H	8.01223900	-0.08107900	-0.79707200
H	6.32720300	0.50470900	-0.90325300
H	7.14505600	0.42285400	0.67604700
C	7.48191100	-2.34782100	0.49850900
H	7.05206800	-3.34236900	0.59750900
H	8.40358000	-2.42132600	-0.09275900
H	7.74655600	-1.98455400	1.50016700
C	2.62443100	3.22922500	-3.21325400
H	2.49130600	3.68951000	-4.19818300
H	2.13978800	2.25159900	-3.20137800
H	2.13498500	3.86012500	-2.46325600
C	4.51804400	5.46002400	-3.26624700
H	5.06422200	5.83284800	-4.14253200
H	3.45144200	5.51170000	-3.47372300
H	4.73676400	6.12375200	-2.41951800
C	6.34054800	3.94519700	-2.61710500
H	6.57318100	4.47651100	-1.68513000
H	6.55886900	2.88683900	-2.49212500
H	6.96235300	4.35960600	-3.41992900

CP29

C	0.63740000	3.55331400	0.56985800
C	1.96176000	3.15257100	0.50630300
C	2.22842500	1.79539100	0.14782500
C	1.11021500	0.93858000	-0.05426900
C	-0.41260400	2.64194100	0.31552400
C	3.54708800	1.28866800	-0.08289900
C	1.33010200	-0.45578400	-0.41369600
C	2.65945000	-0.91950200	-0.61920300
C	3.75111400	-0.00460400	-0.45837800
C	2.84550300	-2.27253000	-1.03269100

C	1.71135000	-3.02730200	-1.27859700
C	0.42191200	-2.51246700	-1.04668400
H	4.39094900	1.96319200	0.00747700
H	0.39737600	4.57663900	0.84231300
H	4.75509700	-0.35306000	-0.66995200
H	1.80960200	-4.05478100	-1.61299900
N	-0.16699900	1.36703000	0.03152900
N	0.24475500	-1.27159900	-0.57374700
C	-1.84905000	3.08758100	0.34772700
H	-2.20151200	3.30734800	-0.66745700
H	-2.49052500	2.29266400	0.73415200
H	-1.97385800	3.99018400	0.95440700
C	-0.78435800	-3.36187500	-1.34767600
H	-1.56276000	-3.23345800	-0.59264400
H	-1.21150100	-3.07804800	-2.31830800
H	-0.51307900	-4.42018400	-1.40408600
C	3.04064600	4.13352500	0.80447500
C	3.06662200	5.37280500	0.14395800
C	4.01794700	3.87107100	1.77962900
C	4.04724000	6.31882600	0.44219700
H	2.32179300	5.58484800	-0.61818200
C	4.99441700	4.82034700	2.08127600
H	3.99609300	2.92787700	2.31834000
C	5.01410800	6.04583100	1.41180900
H	4.05654000	7.26872800	-0.08538000
H	5.73609400	4.60433900	2.84552400
H	5.77655000	6.78365700	1.64589800
C	4.18633900	-2.88976700	-1.21684100
C	5.13691100	-2.89023400	-0.18174100
C	4.50195300	-3.53945500	-2.42136600
C	6.36919100	-3.52124100	-0.35031100
H	4.89821800	-2.41350900	0.76467400
C	5.73803000	-4.16307200	-2.59101900
H	3.77911300	-3.53799000	-3.23264200
C	6.67493600	-4.15650400	-1.55592700

H	7.08857300	-3.52134500	0.46398000
H	5.96911200	-4.65214800	-3.53332400
H	7.63669300	-4.64457700	-1.68694200
C	-1.30784700	-1.17686800	1.85724000
H	-0.23108200	-1.36776500	1.94866400
B	-3.71432200	-0.09148000	-1.39498100
O	-4.48765200	-1.21631100	-1.87114200
H	-5.29735200	-0.90688500	-2.29611000
O	-3.46538000	-0.29308000	0.11849300
H	-3.90820700	-1.12374700	0.36323700
O	-2.29029100	-0.22171100	-1.91784000
H	-2.30284800	-0.99803500	-2.49806200
C	-4.34872500	1.35876600	-1.69523700
C	-3.93210700	2.13756500	-2.78934500
C	-5.40030500	1.87003800	-0.91266500
C	-4.53125900	3.36413400	-3.09094800
H	-3.11299900	1.77506500	-3.40649500
C	-6.00843900	3.09370400	-1.20292300
H	-5.74112400	1.30201700	-0.04865000
C	-5.57455400	3.84650700	-2.29694800
H	-4.18496600	3.94430300	-3.94410300
H	-6.81715600	3.46299000	-0.57551200
H	-6.04358000	4.80051100	-2.52706500
Ni	-1.57784000	-0.67968000	-0.05305800
C	-2.04747600	-2.44825800	2.12185200
C	-1.38184600	-3.69197100	2.07030800
C	-3.42604100	-2.48169000	2.43090800
C	-2.05265100	-4.89594100	2.27718400
H	-0.31183800	-3.70350200	1.87053800
C	-4.10040600	-3.68587500	2.63785800
H	-3.97295900	-1.54841800	2.53373200
C	-3.42218400	-4.90365800	2.55566200
H	-1.50184200	-5.83298200	2.23214600
H	-5.16183100	-3.67004400	2.87435400
H	-3.94729100	-5.84059600	2.72024400

C	-1.68450800	0.00731200	2.75851600
C	-1.41240200	-0.24251200	4.25343600
H	-2.73791300	0.27598200	2.61851500
H	-1.11007800	0.88428600	2.44168200
H	-1.67096800	0.63848400	4.85476600
H	-1.99450500	-1.09062400	4.63034300
H	-0.35203000	-0.46635300	4.42782600

TS18

C	0.52126600	3.08912800	-1.17212600
C	1.81816800	2.72169300	-0.86908800
C	2.06682400	1.34092300	-0.60918600
C	0.96145800	0.44128700	-0.61158600
C	-0.53459600	2.16021000	-1.10883100
C	3.39013500	0.83363000	-0.40253700
C	1.21672400	-0.98907100	-0.46819600
C	2.54142100	-1.43344300	-0.18601700
C	3.61597200	-0.48892100	-0.17644900
C	2.74897000	-2.83488400	-0.00347300
C	1.66249000	-3.67168700	-0.17958200
C	0.39261800	-3.15298400	-0.51737300
H	4.22341200	1.52486300	-0.44692900
H	0.29124700	4.12430100	-1.40043200
H	4.62830500	-0.84841400	-0.03245000
H	1.77803800	-4.74261300	-0.04217100
N	-0.32256100	0.87962300	-0.77747400
N	0.18308200	-1.84576300	-0.65411900
C	-1.93744200	2.61459900	-1.40649000
H	-1.93851700	3.64836100	-1.76563100
H	-2.38043000	1.96986900	-2.17102200
H	-2.55979600	2.56081200	-0.50753800
C	-0.77473000	-4.08943300	-0.68229600
H	-1.28836700	-4.21026200	0.28173000
H	-1.50429600	-3.70160600	-1.39744500
H	-0.43992200	-5.08290300	-0.99944100
C	2.88495000	3.75900200	-0.84422000
C	3.07885800	4.59467600	-1.95583200
C	3.67471500	3.96472700	0.29957700
C	4.04422800	5.60133300	-1.92968300
H	2.47901700	4.44051300	-2.84865200
C	4.63376000	4.97704700	0.32708000
H	3.51992200	3.34148700	1.17579500
C	4.82400100	5.79603500	-0.78811600
H	4.18722500	6.23240300	-2.80256700
H	5.22884300	5.12920200	1.22340900
H	5.57372300	6.58207500	-0.76640600
C	4.07159100	-3.41447800	0.35753300
C	4.77110300	-2.98323500	1.49743500
C	4.61989300	-4.45054900	-0.41587100
C	5.98626300	-3.57044800	1.84976000
H	4.34796300	-2.19965700	2.11963400
C	5.83865900	-5.03212400	-0.06642800
H	4.09201300	-4.78755100	-1.30378200
C	6.52550300	-4.59406400	1.06744000
H	6.50868200	-3.23162500	2.74028900
H	6.25203600	-5.82662300	-0.68175600
H	7.47355600	-5.04868700	1.34134400
Ni	-2.01033500	-0.23040200	-0.49227400
C	-2.10350400	0.01277900	1.50287300
C	-6.03469600	-0.16075600	0.44460400
C	-4.91122300	-0.00068900	-0.36568300
C	-3.94401900	-1.01885900	-0.54062200
C	-4.19066100	-2.22521500	0.14936300
C	-5.30736000	-2.40563000	0.96824500
C	-6.23095900	-1.36876500	1.12234000
H	-6.75634600	0.64652900	0.55153100
H	-4.78309900	0.95270100	-0.87929300
H	-3.49269800	-3.04895300	0.02750000
H	-5.46222400	-3.35096100	1.48443700
H	-7.10293500	-1.50253400	1.75834400
B	-3.33821400	-1.32631200	-2.43072200

O	-4.40795800	-0.84893600	-3.20892500
H	-4.50617600	0.10826700	-3.12963500
O	-3.08511100	-2.70795800	-2.57099500
H	-3.89716000	-3.14509000	-2.86350300
O	-2.09765800	-0.54557600	-2.47066900
C	-1.97889900	1.44101000	1.93382000
C	-0.74830400	2.06022200	2.23564700
C	-3.13657400	2.23888100	2.07117300
C	-0.67657200	3.39690900	2.63118800
H	0.17013800	1.48362300	2.16773300
C	-3.06901800	3.57536300	2.46189200
H	-4.10506600	1.78553800	1.87329500
C	-1.83486800	4.16887500	2.74242000
H	0.29231800	3.83675400	2.85880900
H	-3.98551600	4.15333500	2.56020200
H	-1.77874400	5.20875900	3.05368200
C	-1.14434700	-0.96860500	2.19376100
C	-1.37091500	-1.08130700	3.71300000
H	-0.09622400	-0.69980900	2.00971700
H	-1.27741400	-1.96161300	1.74792600
H	-0.68532800	-1.80970500	4.16581500
H	-1.21857000	-0.11786800	4.21217200
H	-2.39616100	-1.40668300	3.92852900
H	-3.12284400	-0.30972500	1.72403000
H	-1.35125100	-1.15002900	-2.62094300

CP30

C	1.62963500	-3.42949800	-0.64642700
C	2.62741900	-2.56517900	-0.21061500
C	2.34013500	-1.16852300	-0.25825600
C	1.02329400	-0.78014900	-0.61934200
C	0.36869500	-2.96077100	-1.05973900
C	3.32308000	-0.14303800	-0.05509900
C	0.69166400	0.62303400	-0.70591400
C	1.69432300	1.61159600	-0.53120100
C	3.02172400	1.17942600	-0.20303900
C	1.32400500	2.97092800	-0.75805500
C	0.02794500	3.21566100	-1.19698400
C	-0.92133200	2.18275800	-1.31147900
H	4.34158800	-0.43774100	0.17186800
H	1.82205300	-4.49794700	-0.67089900
H	3.80330800	1.92336700	-0.09888300
H	-0.28269800	4.23472000	-1.40585700
N	0.03779600	-1.66160100	-0.97229700
N	-0.60634100	0.91721300	-1.00792900
C	-0.62524700	-3.89968300	-1.68372800
H	-0.74368000	-3.65338400	-2.74699500
H	-0.29173100	-4.93916600	-1.60824400
H	-1.61372800	-3.80368900	-1.22909700
C	-2.32962100	2.47350000	-1.75158800
H	-2.99624200	2.57440100	-0.88809200
H	-2.37464000	3.40568000	-2.32412200
H	-2.70552200	1.65430100	-2.37200000
C	3.93276300	-3.10939100	0.24353400
C	4.49418200	-2.72563100	1.47436000
C	4.61411400	-4.06298600	-0.53230200
C	5.69853900	-3.27619700	1.91202800
H	3.96924800	-2.00884600	2.09941000
C	5.82040400	-4.60997900	-0.09574500
H	4.20092500	-4.35997400	-1.49234700
C	6.36735100	-4.21877300	1.12806600
H	6.11084900	-2.97332000	2.87084500
H	6.33558400	-5.33938000	-0.71509900
H	7.30660100	-4.64579300	1.46874500
C	2.26153500	4.10886300	-0.56722400
C	2.43551000	5.05978400	-1.58689900
C	2.95184000	4.29224300	0.64359200
C	3.28012700	6.15480400	-1.40603700
H	1.91692400	4.92540700	-2.53225600
C	3.79232600	5.39036500	0.82536600
H	2.80912100	3.58194100	1.45298600

C	3.96175800	6.32408000	-0.19924300
H	3.40812900	6.87411000	-2.21050000
H	4.30996500	5.51985100	1.77207900
H	4.61873500	7.17780200	-0.05749400
Ni	-1.78780400	-0.65174300	-0.56669800
C	-1.81670100	-1.04763100	3.73456200
C	-1.60437300	-1.12342000	2.21210700
H	-2.67495600	-1.65888800	4.04049600
H	-0.93475100	-1.41616900	4.27253200
C	-2.83372100	-0.64774500	1.41334400
H	-2.00765000	-0.02006000	4.06219100
H	-1.38551200	-2.16222600	1.93958900
H	-0.70817600	-0.55391200	1.94127400
H	-3.69097000	-1.15986500	1.85597500
C	-3.16630200	0.81937300	1.48797600
C	-4.50697000	1.23211000	1.33238900
C	-2.22129000	1.81723600	1.80178700
C	-4.87617700	2.57272800	1.44267600
H	-5.26776000	0.48306400	1.12889300
C	-2.59246300	3.15464400	1.93144800
H	-1.18068000	1.54584900	1.94987600
C	-3.92118200	3.54700300	1.74291800
H	-5.91868900	2.85355400	1.31188800
H	-1.83712200	3.89531200	2.18370000
H	-4.20852500	4.59012800	1.84404500
C	-3.28020300	-1.66805400	-0.15241100
C	-3.43007500	-3.05533500	0.11944400
C	-4.22239000	-1.07761900	-1.03914700
C	-4.39008700	-3.81633500	-0.53623400
H	-2.78257600	-3.53274600	0.85261600
C	-5.15532300	-1.86373600	-1.72821600
H	-4.24843500	0.00193600	-1.16190200
C	-5.24831700	-3.23273700	-1.48317100
H	-4.47166500	-4.87941500	-0.31736900
H	-5.83730900	-1.38990600	-2.43177400
H	-5.99526000	-3.83584100	-1.99196700

TS19

C	-0.11408400	3.54957000	-0.63595800
C	-1.39726400	3.13185100	-0.31794600
C	-1.64888800	1.72690600	-0.35048700
C	-0.54697100	0.86520700	-0.59030500
C	-2.95826300	1.15478600	-0.23833700
C	-0.76480800	-0.56107400	-0.69361600
C	-2.07811900	-1.09098600	-0.59181200
C	-3.16525400	-0.18772100	-0.35934500
C	-2.24281500	-2.49348700	-0.80038800
C	-1.12014900	-3.21973200	-1.16881800
C	0.15193400	-2.61803700	-1.23878100
H	-3.80454200	1.81724500	-0.09572400
H	0.11229700	4.61143600	-0.66583500
H	-4.17375600	-0.58273700	-0.31438400
H	-1.20985100	-4.28370400	-1.36826100
N	0.72901400	1.30912200	-0.80371800
N	0.33298700	-1.32785500	-0.94814200
C	1.34992000	-3.43946400	-1.63415800
H	1.66495500	-4.10578600	-0.82130300
H	1.11665100	-4.07261600	-2.49817900
H	2.18738100	-2.78586900	-1.88565600
C	-2.44423900	4.13651900	0.00352000
C	-3.21100300	4.03987400	1.17794600
C	-2.65284000	5.23875000	-0.84256800
C	-4.15772900	5.01442200	1.49296500
H	-3.04524400	3.20761600	1.85614800
C	-3.60307700	6.21032900	-0.52900300
H	-2.07633200	5.32060900	-1.75994900
C	-4.35904400	6.10177800	0.63994200
H	-4.73385700	4.92722100	2.41030500
H	-3.75531300	7.05067500	-1.20104000
H	-5.09850200	6.85919500	0.88514700
C	-3.55210600	-3.18396900	-0.65931200

C	-4.03263900	-4.00897000	-1.68971100
C	-4.30974600	-3.07247400	0.51936500
C	-5.23889900	-4.69532600	-1.55045200
H	-3.46257400	-4.09549200	-2.61074200
C	-5.51291900	-3.76390100	0.65977100
H	-3.93964800	-2.45816600	1.33525100
C	-5.98311300	-4.57531300	-0.37515600
H	-5.59874800	-5.32138600	-2.36252900
H	-6.08041000	-3.67311500	1.58208200
H	-6.92205700	-5.11101600	-0.26574400
Ni	2.03844400	-0.23939500	-0.29657300
C	3.44386500	-1.04194100	1.16314000
C	3.89819900	0.13915200	-0.24275800
C	4.56422200	1.30479400	0.22790800
C	4.39659800	-0.41689400	-1.45285400
C	5.57323900	1.92037700	-0.50145400
H	4.27567000	1.72973800	1.18836100
C	5.38750100	0.22617000	-2.20291900
H	4.00563400	-1.36580300	-1.81185600
C	5.98306700	1.39881400	-1.73810300
H	6.04873100	2.81702700	-0.10793300
H	5.71304900	-0.21310600	-3.14409600
H	6.77505000	1.88020900	-2.30537700
C	2.10706700	-0.98846800	1.87565700
C	1.24743500	-2.10253700	2.01620700
C	1.73619300	0.19220300	2.56799000
C	0.06752700	-2.02275600	2.75323800
H	1.50661000	-3.04336500	1.54158100
C	0.55111000	0.27284000	3.29420300
H	2.40264800	1.05068600	2.53670400
C	-0.29961600	-0.83319700	3.38881100
H	-0.56580500	-2.90299400	2.83932600
H	0.29838700	1.19990000	3.80389200
H	-1.21784200	-0.77618900	3.96715400
C	4.04384300	-2.43059100	0.92331100
C	4.50431300	-3.12021000	2.21778300
H	4.90647000	-2.32150600	0.25628400
H	3.33651800	-3.07680700	0.39267500
H	4.94730400	-4.10046400	2.00333600
H	5.26481700	-2.51759100	2.72959100
H	3.67417600	-3.26981700	2.91659000
H	4.13712200	-0.49666400	1.80578100
C	0.92275100	2.63186500	-0.89853400
C	2.28065900	3.13509700	-1.29828100
H	2.92045000	3.29882600	-0.42482900
H	2.79191200	2.40596500	-1.92986800
H	2.19307000	4.08649800	-1.83405900

4b

C	3.63153300	-0.32302800	0.80955500
C	2.42080400	0.33729400	1.02626400
C	1.33501100	0.16455500	0.15668800
C	1.49392200	-0.69500900	-0.93902100
C	2.70280700	-1.35633100	-1.16055800
C	3.77729600	-1.17316700	-0.28761300
H	4.45783800	-0.17597500	1.50050000
H	2.31575100	0.99786700	1.88478600
H	0.66109600	-0.86127600	-1.61635300
H	2.80302800	-2.02068000	-2.01522300
H	4.71694700	-1.69177900	-0.45848900
C	0.04183900	0.95018200	0.39324200
C	0.04076800	2.24531700	-0.45726300
H	0.06269900	1.27178500	1.44388300
C	-1.10511200	3.20915400	-0.13015400
H	1.00043600	2.74949300	-0.28794600
H	0.02917200	1.98833300	-1.52440900
H	-1.02894000	4.12249000	-0.73125100
H	-2.08348200	2.75790800	-0.32500900
H	-1.08065100	3.50364800	0.92654400
C	-1.20517300	0.08203300	0.23126600
C	-1.66655700	-0.66015300	1.32873600

C	-1.90190000	-0.03560400	-0.97997500
C	-2.77888700	-1.49453900	1.22381800
H	-1.14043000	-0.58357100	2.27793500
C	-3.01712100	-0.87036500	-1.09116300
H	-1.58204000	0.53307300	-1.84860400
C	-3.46016700	-1.60392100	0.00944600
H	-3.11533400	-2.05700500	2.09104100
H	-3.54118700	-0.94217400	-2.04089500
H	-4.32928600	-2.25064800	-0.0761220

CP0

C	3.44718300	1.01435500	-0.03623900
C	2.85772800	-0.25208400	0.02136300
C	1.43354700	-0.30668300	0.03063100
C	0.71462300	0.91374100	-0.06982100
C	2.68449000	2.17646200	-0.19073900
C	0.67822700	-1.51816900	0.17783500
C	-0.71809400	0.92024200	0.03999500
C	-1.43914200	-0.30316400	0.04950100
C	-0.68553100	-1.52026700	0.15206400
C	-2.86266100	-0.24699400	0.04087700
C	-3.45265500	1.01920700	0.05827200
C	-2.68704200	2.18896000	0.11985500
H	1.21235600	-2.45114800	0.31684600
H	4.52976100	1.09487100	-0.05711000
H	-1.22252600	-2.45581400	0.26063100
H	-4.53508000	1.10241000	0.04694900
N	1.33821400	2.13151400	-0.28053300
N	-1.33963200	2.15070700	0.16223600
C	3.32677900	3.52886800	-0.33472600
H	4.38915500	3.45163300	-0.58715600
H	2.80743500	4.10192700	-1.11378500
H	3.24453800	4.10306700	0.59731000
C	-3.32868400	3.54857400	0.17407800
H	-2.78592700	4.18366700	0.88570100
H	-3.28005700	4.04444700	-0.80406600
H	-4.38176000	3.49096800	0.46742500
C	3.72345900	-1.45612100	0.07401400
C	3.54795800	-2.53262600	-0.81499600
C	4.78825500	-1.52037800	0.99083400
C	4.40142000	-3.63499000	-0.78111800
H	2.75288400	-2.48988000	-1.55377400
C	5.64033500	-2.62371800	1.02516500
H	4.93137000	-0.70395800	1.69352000
C	5.44986700	-3.68699600	0.14026000
H	4.25228000	-4.45106900	-1.48337700
H	6.45074500	-2.65493400	1.74880100
H	6.11333400	-4.54713000	0.16670800
C	-3.72556800	-1.45450000	0.00926800
C	-4.78976000	-1.58702200	0.91922500
C	-3.54271400	-2.46650000	-0.95075100
C	-5.63471700	-2.69530000	0.87817500
H	-4.93868600	-0.82053000	1.67497900
C	-4.38976600	-3.57387800	-0.99259900
H	-2.74596200	-2.36931900	-1.68259200
C	-5.43787200	-3.69445300	-0.07768600
H	-6.44488700	-2.78098300	1.59763700
H	-4.23518500	-4.33975100	-1.74819100
H	-6.09642500	-4.55813700	-0.11049400
Ni	-0.00019400	3.48766800	-0.12158200

CP1’

C	0.87831500	-3.41736900	0.55215400
C	2.16097100	-2.86039200	0.40351300
C	2.22229700	-1.46820300	0.13921800
C	1.00250900	-0.73366900	0.07505900
C	-0.27403400	-2.63662200	0.52543700
C	3.44014400	-0.74851800	-0.10847700
C	1.00766400	0.65181000	-0.23816100
C	2.23485800	1.36737300	-0.34291600
C	3.44872800	0.60028600	-0.30718000
C	2.18815100	2.76568300	-0.57931400

C	0.91345200	3.32986700	-0.75970400
C	-0.24890300	2.56558000	-0.70664300
H	4.37371600	-1.29879800	-0.14595200
H	0.78654800	-4.47195900	0.79582800
H	4.38937400	1.10649200	-0.49449800
H	0.82877600	4.39300000	-0.96582700
N	-0.24116700	-1.29437900	0.32905700
N	-0.23431100	1.22971000	-0.46486800
C	-1.61392900	-3.26116000	0.80506700
H	-1.50421900	-4.24351100	1.27621800
H	-2.19581900	-2.60597800	1.46462800
H	-2.19335400	-3.39373500	-0.11698700
C	-1.58427500	3.20622400	-0.97212000
H	-2.20599600	2.52788400	-1.56887000
H	-2.12537100	3.41083300	-0.03982100
H	-1.46990700	4.15651700	-1.50450300
C	3.34960400	-3.73288100	0.54297700
C	4.44047800	-3.38700800	1.36454600
C	3.38990900	-4.98246500	-0.10581000
C	5.52771600	-4.24659200	1.51749500
H	4.41775700	-2.44729600	1.90812700
C	4.47590500	-5.84275700	0.04888900
H	2.56475500	-5.26567900	-0.75378300
C	5.55339500	-5.47878400	0.85978500
H	6.35281500	-3.95722000	2.16381000
H	4.48397900	-6.79731500	-0.47180100
H	6.40122500	-6.14797800	0.98026500
C	3.38793900	3.63014400	-0.65399800
C	3.51797300	4.58545800	-1.68114500
C	4.40720900	3.57096800	0.31699800
C	4.62108500	5.43514800	-1.74194300
H	2.75144800	4.64066600	-2.44947800
C	5.51201200	4.41954800	0.25459200
H	4.31441500	2.86835700	1.13983600
C	5.62676300	5.35623000	-0.77508500

H	4.70003800	6.15589500	-2.55228900
H	6.28063000	4.35614500	1.02108100
H	6.48769900	6.01779500	-0.82269200
Ni	-1.71248600	-0.00342100	-0.02508200
N	-4.62608400	-2.56877700	-1.92347000
C	-3.89029100	-2.59966400	-3.18241800
H	-4.54684600	-2.31030300	-4.01333000
H	-3.05403100	-1.90594000	-3.11793800
H	-3.51273500	-3.61194100	-3.37648800
C	-5.77078500	-3.46094800	-1.85088300
H	-6.29215200	-3.36034700	-0.90052000
H	-6.47993700	-3.23526900	-2.65872000
H	-5.45391900	-4.50696400	-1.96327800
C	-4.20283600	-1.75359500	-0.90896200
O	-3.21380800	-1.01960300	-1.06449500
C	-4.96643100	-1.76332700	0.40323200
H	-4.45521700	-1.07915300	1.08067500
H	-6.00388100	-1.43289400	0.27347700
H	-4.98631200	-2.76193400	0.85365600
N	-4.11779200	2.80191900	2.17005600
C	-3.19734800	2.75148800	3.30047600
H	-3.72531400	2.44785700	4.21453100
H	-2.40941500	2.03295900	3.08165300
H	-2.75758800	3.74237300	3.46766900
C	-5.17788200	3.79025500	2.27435800
H	-5.83456800	3.75972000	1.40654000
H	-5.78413300	3.60839700	3.17260400
H	-4.75417100	4.80037200	2.35279200
C	-3.97333100	1.91474000	1.13863000
O	-3.07725400	1.05594800	1.16747200
C	-4.92505500	2.00020700	-0.04162400
H	-4.57813200	1.28242700	-0.78554400
H	-5.95349200	1.75079400	0.24648100
H	-4.93493200	2.99913600	-0.49075600

Ph-B(OH)$_2$

C	-1.95010600	-1.20679200	0.00981700
C	-0.55405400	-1.20271400	0.00970200
C	0.17171800	-0.00003500	-0.00000300
C	-0.55404500	1.20266000	-0.00969800
C	-1.95007500	1.20677500	-0.00981500
C	-2.65202100	0.00000600	-0.00000300
H	-2.48929300	-2.15088000	0.01751300
H	-0.02412900	-2.15303300	0.01730200
H	-0.02409000	2.15296900	-0.01729300
H	-2.48923700	2.15086500	-0.01751200
H	-3.73914500	0.00001500	-0.00000400
B	1.74347800	0.00002600	-0.00000200
O	2.39142800	-0.01985100	-1.20975800
H	3.35859300	-0.02045500	-1.14735900
O	2.39142400	0.02020600	1.20975300
H	3.35859000	0.01814500	1.14740300

LiOH 3DMA

O	1.25547100	0.94827600	1.76373500
H	0.90144000	1.23302400	2.61999800
O	0.02504600	-1.62317600	0.33094500
Li	-0.03087800	0.37665700	0.62604400
C	1.59505000	1.82813600	-0.85053700
O	0.67663400	1.09590300	-1.26812600
C	-3.04542700	0.24129300	0.57269300
O	-1.95833200	0.80166000	0.76991800
N	-3.34354000	-0.35394100	-0.61289400
N	1.37397700	3.13300100	-0.51974400
C	-4.09263800	0.23411100	1.67750000
H	-5.05686000	0.63236800	1.34386100
H	-4.25806400	-0.77926000	2.06124700
H	-3.71163000	0.85420900	2.48932600
C	3.00267100	1.28921700	-0.71609400
H	3.77024600	1.98906600	-1.06122200
H	3.15215200	1.06752200	0.34698400
H	3.06799400	0.36344600	-1.29075300
C	2.25091800	3.79173600	0.44953600
H	1.98275400	4.85042100	0.50559100
H	2.13183500	3.31067800	1.42883100
H	3.29624600	3.72623800	0.13979800
C	-0.01193900	3.59195600	-0.51846200
H	-0.52971600	3.17886300	-1.38379700
H	-0.53921700	3.26654600	0.38854200
H	-0.02204000	4.68504300	-0.57297900
C	-4.51884700	-1.17861500	-0.84597500
H	-5.23600900	-1.08854300	-0.03168200
H	-5.01278000	-0.87191600	-1.77627600
H	-4.23696900	-2.23628500	-0.94362600
C	-2.34084000	-0.37753200	-1.67837400
H	-1.65725300	0.46287600	-1.56647300
H	-1.75224500	-1.30086100	-1.63659500
H	-2.85446900	-0.31248500	-2.64387500
C	1.08260100	-2.18708200	0.67561700
C	1.55736300	-2.04392300	2.09821800
H	0.77984300	-2.41308600	2.77509300
H	2.50790200	-2.52192400	2.33940600
H	1.62972400	-0.93805700	2.23042000
N	1.82902600	-2.83769800	-0.26350300
C	1.42275400	-2.72328900	-1.66239600
H	0.99617500	-1.73447400	-1.83391300
H	2.30547300	-2.85622600	-2.29603900
H	0.68065600	-3.48714700	-1.93347500
C	2.94076600	-3.73753300	0.00252900
H	3.88219100	-3.33434600	-0.39404900
H	3.05699400	-3.90952400	1.07064500
H	2.75687700	-4.70781400	-0.477439
